# An Integrated System for the Automated Recording and Analysis of Insect Behavior in T-maze Arrays

**DOI:** 10.3389/fpls.2019.00020

**Published:** 2019-01-29

**Authors:** Maarten A. Jongsma, Manus P. M. Thoen, Leo M. Poleij, Gerrie L. Wiegers, Paul W. Goedhart, Marcel Dicke, Lucas P. J. J. Noldus, Johannes W. Kruisselbrink

**Affiliations:** ^1^Wageningen Plant Research, Wageningen University and Research, Wageningen, Netherlands; ^2^Laboratory of Entomology, Wageningen University and Research, Wageningen, Netherlands; ^3^Noldus Information Technology BV, Wageningen, Netherlands

**Keywords:** video tracking, insect, plant, EntoLab, EthoAnalysis, *Frankliniella occidentalis*, *Arabidopsis*

## Abstract

Host-plant resistance to insects like thrips and aphids is a complex trait that is difficult to phenotype quickly and reliably. Here, we introduce novel hardware and software to facilitate insect choice assays and automate the acquisition and analysis of movement tracks. The hardware consists of an array of individual T-mazes allowing simultaneous release of up to 90 insect individuals from their individual cage below each T-maze with choice of two leaf disks under a video camera. Insect movement tracks are acquired with computer vision software (EthoVision) and analyzed with EthoAnalysis, a novel software package that allows for automated reporting of highly detailed behavior parameters and statistical analysis. To validate the benefits of the system we contrasted two *Arabidopsis* accessions that were previously analyzed for differential resistance to western flower thrips. Results of two trials with 40 T-mazes are reported and we show how we arrived at optimized settings for the different filters and statistics. The statistics are reported in terms of frequency, duration, distance and speed of behavior events, both as sum totals and event averages, and both for the total trial period and in time bins of 1 h. Also included are higher level analyses with subcategories like short-medium-long events and slow-medium-fast events. The time bins showed how some behavior elements are more descriptive of differences between the genotypes during the first hours, whereas others are constant or become more relevant at the end of an 8 h recording. The three overarching behavior categories, i.e., choice, movement, and halting, were automatically corrected for the percentage of time thrips were detected and 24 out of 38 statistics of behavior parameters differed by a factor 2–6 between the accessions. The analysis resulted in much larger contrasts in behavior traits than reported previously. Compared to leaf damage assays on whole plants or detached leaves that take a week or more to complete, results were obtained in 8 h, with more detail, fewer individuals and higher significance. The potential value of the new integrated system, named EntoLab, for discovery of genetic traits in plants and insects by high throughput screening of large populations is discussed.

## Introduction

Breeding for host-plant resistance has gained much interest in recent years due to the increasing bans on the use of chemical pesticides. A crucial element in the breeding process is the accurate estimation of the resistance level of large populations of plant accessions (Eigenbrode and Trumble, 1994). This requires robust phenotyping systems that can accurately screen many different plant lines in a high-throughput manner (Kloth et al., 2012; Goggin et al., 2015).

Current phenotyping methods mainly focus on costly, labor-intensive and time-consuming end-point measurements of feeding damage or insect performance (reproduction and mortality). Clip-on cage techniques are not effective with thrips as they easily escape. Conventional visual rating systems that score feeding damage often do not allow precise quantification and are sensitive to subjectivity and inconsistency of the scoring process, although recently a more objective method was reported (Visschers, 2018). Usually experiments are, therefore, done in greenhouses with an open choice situation that suffer from environmental effects. An alternative, automated high-throughput and controlled process to phenotype host-plant resistance to thrips would therefore greatly aid research and the breeding process for thrips-resistant cultivars.

We have recently demonstrated the value of automated video tracking of peach aphid (*Myzus persicae*) (Kloth et al., 2015) and Western flower thrips (*F. occidentalis*) to establish host-plant resistance levels in *Arabidopsis* (*Arabidopsis thaliana)* (Thoen et al., 2016) and locate associated genes in Genome Wide Association Study (GWAS) populations (Kloth et al., 2016, 2017; Thoen et al., 2017). Those studies were, however, done in microtiter plates that were not suitable for T-maze applications, nor for the study of whole leaves, and suffered from poor detection due to condensation of water droplets on the inside of the plastic cover after a few hours. In thrips choice experiments, movement and halting in a two-choice setting was only reported using the sum total time or speed of all movement or halting events, and not the event averages which would have given more detailed and differentiating information of the nature of halting or movement behavior (Thoen et al., 2016). All studies so far were further sensitive to differences in detection percentages (tracking efficiency). Extraction, of more detailed behavior categories, that could distinguish short and long or fast and slow moving or halting events, were only reported in the aphid studies, as this required a large amount of extra offline manual processing and *ad hoc* programming work. Not analyzing the more detailed behavior information contained in the thrips tracks, however, neglects important details of animal behavior that can be far more informative than sum totals (Benjamini et al., 2010; Kloth et al., 2017). The goal of the development of the EntoLab™ phenotyping system presented here was to overcome these shortcomings, and automate the data extraction and statistical analysis. Here, we validate its utility for the study of thrips in a T-maze setting. It involves a camera, camera stand, illumination, and EthoVision® software, plus newly developed T-maze array hardware, and EthoAnalysis™ software for automated data extraction and statistical analysis. This system is shown with fewer insects to deliver larger contrasts that are more accurate and significant compared to the previous report (Thoen et al., 2016). Furthermore, it provides more detailed insight in the behavior of insects like thrips on different plant genotypes. With two accessions the system is operated using the following workflow:

1. T-maze array experiment: video recording of thrips behavior in a fully controlled environment. Leaf discs of two accessions are placed into each of the parallel T-maze arenas. In each arena, one insect is placed and behavior is video-recorded over a time span of 8 h.
2. Extracting video tracks: EthoVision video-tracking software is used to determine the position, zone, and velocity of each insect in each video frame during the complete run of the experiment.
3. Analysis of behavior statistics: EthoAnalysis is used to convert raw tracking data exported from EthoVision into zone-specific movement and halting events, and to generate higher level behavior statistics, that are less sensitive to differences in tracking efficiency (detection) between genotypes. The statistics include zone preference, average velocity, total time moving/halting, short/medium/long moving and halting duration and slow/medium/fast moving events, mostly also per hourly time bin. For these choice assays, the differences between the behavior statistics in the two zones containing the genotypes are modeled in order to establish differences in insect preference between two genotypes.

For validation of the system, we used two wild *A. thaliana* accessions, Cur-3 and Rmx-A180. In the previous study Cur-3 was shown to be much more resistant to thrips in choice settings with Rmx-A180 as susceptible reference genotype (Thoen et al., 2016). In that study, the behavior of Western flower thrips (*F. occidentalis*) was recorded in 88 parallel arenas the size of a single 6 mm microtiter-plate well filled with two half leaf discs. The results were found to be consistent with choice assays with whole plants and detached leaves in which damage levels were assessed.

In this follow up study, we introduce a novel parallel arena plate with T-maze type designs. The two leaf discs each occupied their own 6 mm well and were separated by a central well from which simultaneous access by a single thrips individual was possible after shifting a gate plate. EthoAnalysis subsequently provides a number of filters to automatically remove entire records or specific events based on various quality criteria. Setting these filters and tuning their parameters generally allows navigating between data quality and data quantity. Additionally, for some behavior statistics, EthoAnalysis requires insect specific parameters, like time intervals for probing vs. feeding to be set. We discuss the potential contribution of the EntoLab system to studies in the field of plant-insect science and resistance breeding.

## Materials and Methods

### Plants

We used *Arabidopsis thaliana* as host plant species. Accessions Rmx-A180 (CS76220, collected by J. Bergelson, latitude 42,036, longitude −86,511, Michigan, USA) and Cur-3 (CS76115, collected by F. Roux, latitude 45,000, longitude 1,750, France) were used for this study. For insect assays, plants were grown from seeds in small plastic pots (5 cm diameter) on pasteurized soil (4 h at 80°C; Lentse potgrond, Lent, The Netherlands) in a climate room (21 ± 1°C, 50–70% relative humidity; 8 h:16 h L:D photoperiod; light intensity 200 μmol m^−2^ s^−1^). For both reported experiments, ten 5-weeks-old plants were used. From each plant the top 4 youngest leaves large enough to produce 4 leaf discs of 6 mm were harvested. Leaf discs of both genotypes were subsequently randomly combined but their leaf position was recorded in the software to allow analysis of such aspects if desired.

### Insects

The Western flower thrips [*Frankliniella occidentalis* (Pergande)] used in this study were collected from chrysanthemum flowers in a climate chamber (25 ± 1°C, L:D 16:8). In the experiments non-synchronized adult females were used, that were starved overnight in Perspex tubular cages closed on one side with gauze and on the other side with two layers of stretched sheets of Parafilm containing a droplet of water to enable drinking. Thrips were anesthetized with CO_2_ and placed on ice just prior to experiments.

### T-maze Array Plate

**Figure 1:**
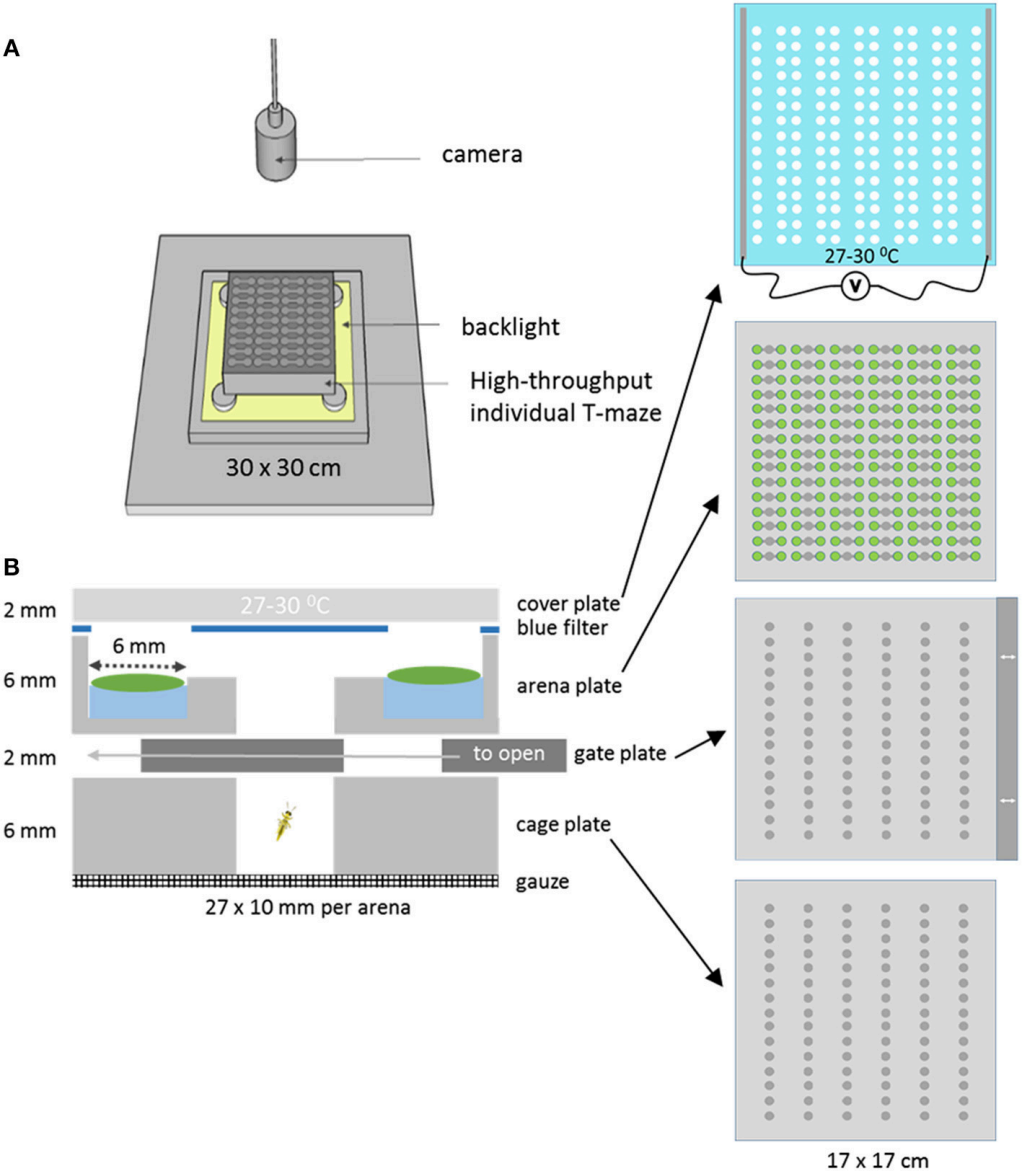
The T-maze array set up. **(A)** The T-maze array consisted of stacked layers of Perspex plates topped with a heated glass cover. It was lit from below with a backlight and monitored from above with a camera. **(B)** Cross section of one single T-maze arena of the complete array of 90. The bottom compartment of a cage plate closed with a gate plate, that could create access to the arena plate by sliding it to the left. The T-maze arena consisted of two leaf disk zones with disks placed on 20 μl water, a blue filter with holes in the position of the leaf discs, and a heated cover plate made of glass.

**Figure 2:**
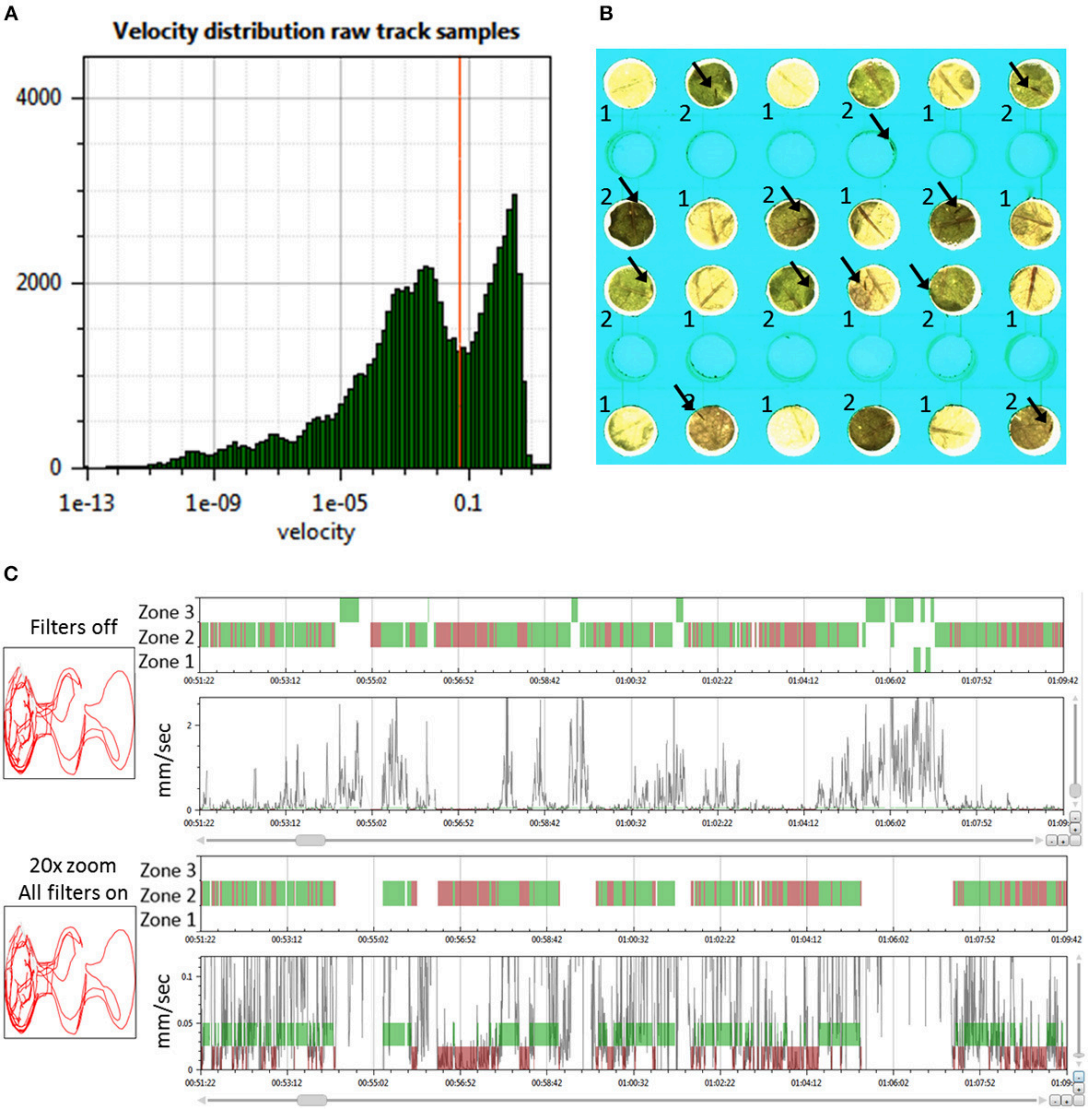
Determining the behavior state and zone position of thrips. **(A)** Histogram of the velocity distribution of one thrips in a selected arena to aid in selecting proper thresholds for the determination of movement and halting events (velocity in mm/s); **(B)** Zoom of 12 arenas of the T-maze array with the two *Arabidopsis* accessions Cur-3 (1) and Rmx-A180 (2) in alternating positions. Arrows indicate the position of thrips individuals. The central empty well provided the access route of thrips from the cage plate below; **(C)** View on the left of the 2D arena shape position trace (red line) and on the right the 1D thrips zone position (zones 1–3 for Cur-3, Rmx-A180 and other) and velocity traces (black line) and movement state (green band for moving and red for halting) for a selected time interval (ca 18 min): thrips per arena are assigned to either a movement or halting state based on the velocity threshold and look-ahead window settings. The second panel is a Y-zoom of the velocity trace. It shows the effects of the velocity threshold (0.05 mm/s) and filters. All movement events in zone 1 and 3 are removed by applying all filters because in those zones they do not start and end with halts within the zone.

A novel T-maze array plate was designed to allow easier and controlled high throughput T-maze behavior studies with insects using leaf discs. The T-maze array setup was created by stacking multiple layers of micro-machined and laser cut Perspex in a holder (Figure 1). In the bottom cage plate cold-anesthetized insects are placed row by row and retained there by covering the cages with a gate plate. The gate plate prevents the insects from entering the top arena plate until it is pushed into the stack by about ~1 cm at the start of the trial, so that the holes in the gate plate simultaneously create access to all 90 arenas of the arena plate. Each arena consists of three separate circular zones of 6 mm diameter, arranged in a row. The zones are connected through 2 mm wide and deep tunnels. The two outer zones are 4 mm deep, while the center zone cuts through the plate, and is used to release the insects into the arena from the cage below. Leaf discs are placed on 20 μl water in the two outer zones. To prevent condensation, the glass cover plate was coated with indium tin oxide and heated to 27–30°C by applying a voltage. The coating did not affect the transparency of the glass. The whole plate stack was placed in a holder and mounted for video recording. In this study only 40 of the 90 available parallel T-maze arenas were used, because for a simple comparison of just two genotypes 40 replications are more than enough. A blue color filter with excised holes in the positions of the leaf discs was placed on top of the arena plate below the cover plate to equalize the light intensities from areas with and without leaves (Figures 1B, 2B). This improved the tracking success when the insects were moving between zones with a high contrast of direct and leaf disc filtered backlight (data not shown).

### Video-Tracking Setup and Experiment

Thrips behavior was recorded with a digital video camera (Basler acA2040-25gc, 1″ CMOS sensor, Kowa LM35HC lens, 35 mm/F1.4). A backlight unit (LED panel 30 × 30 cm, 24 V and 18 W, 5,000 K) was used to illuminate the arenas. The arena plate with T-maze arenas was mounted ~1 cm above the backlight unit, with ventilation in between to prevent heating. Room temperature was kept constant at 21–22°C. Videos were recorded at the maximum resolution of 2,046 × 2,046 pixels at 10 video frames per second using Debut Video Capture software (version1.88, http://nchsoftware.com/capture/index.html). Eight-hour recordings of 40 parallel two-choice assays were run (Figure 2B). For each arena, leaf discs of 6 mm from both accessions (Cur-3 and Rmx-A180) were placed on 20 μl of water in alternating arms of the T-maze. A section of 4 × 10 arenas was filmed representing a size of 108 × 100 mm, implying a resolution of ~20 pixels/mm or 50 micron per pixel.

### EthoVision Video-Tracking Settings and Export

We tracked thrips behavior with EthoVision® XT 11.5 video tracking and analysis software (Noldus Information Technology BV, Wageningen, The Netherlands, www.noldus.com/ethovision) at 3.33 frames/s. Each leaf disc was assigned to a specific zone (Z1 and Z2); in addition, there was a neutral zone that did not contain leaf material (Z3). Dynamic subtraction and center-point detection were used as detection methods, with a dark contrast of 8–255. Subject size detection was limited to the range of 40–385 pixels. Pixel smoothing was set to medium. Moving thresholds were not set because EthoVision was only used for tracking and not for event analysis. Raw data files with genotypes in a “Genotype” column separated by a “$” sign were exported per subject for analysis in EthoAnalysis as .txt files for each arena (ca 1 Mb per arena per hour).

### EthoAnalysis Extraction of Behavior Events From Tracking Data

The video tracking software EthoVision produces series of track samples (set at 3.33 samples/s, 10800/h) for all insects/arenas as described before (Thoen et al., 2016). Each track sample contains an (x,y)-coordinate, a velocity, and an indication of the current zone in which the insect resides unless the tracking is not successful because EthoVision could not detect the insect, in which case the sample is recorded as not detected. These track files were imported by EthoAnalysis, a software package developed by Wageningen Plant Research.

Supplementary Data File 1 provides screenshots of how the software processes the data in steps based on consecutive tabs. In the “Project” tab with a new project a dialogue box is given where the experiment should be named and the location of the data indicated. A summary of genotypes and experiments (arenas) is given once data are added in the other tabs. In the “Input data” tab (Supplementary Data File 1) trials and experiments can be added or removed. Crucial are the import settings which require a value for the look-ahead window, and the velocity threshold. The look-ahead window exists to ignore minor drops below the velocity threshold within a movement event, or single spikes above the velocity threshold while halting and is an important tool to accurately follow the unique behavior of specific insects. Based on these settings EthoAnalysis software translates these raw track data into series of zone-specific behavior events of three types: halting, moving, and not-detected (events that do not contribute to the calculation of behavior statistics) using the following procedure:

- Start at the first track sample at the beginning of the trial with a *movement state unknown* and process the track file sample by sample in time.
- Iterate over the sample records using the following decision rules:

- Determine the *movement state* of the current sample based on these rules:
  1. Current state is *moving* if either of the following two conditions is met:
    - **Start moving:** The previous state is not *moving* **and** the current velocity is greater than or equal to the velocity threshold **and** any of the next *n* samples (the look-ahead window) has a velocity greater than or equal to the velocity threshold. This protects both the moving and halting states from short halting or movement spikes.
    - **Remain moving:** The previous state is *moving* **and** any velocity in the current or the next *n* samples is greater than or equal to the velocity threshold.
  2. Else, if the current state is not recognized as *moving*, the current state is *halting* if either of the following two conditions is met:
    - **Start halting:** The previous state is not halting and the current sample has a positive detection.
    - **Remain halting:** The previous state is halting and any sample in the current or the next *n* samples has a positive detection.
  3. Otherwise, if the current state is not recognized as *moving* or *halting*, the current state is set to *not-detected*.
- If the current *movement state* is not equal to the previous *movement state* **or** the current zone is not equal to the previous zone, then add a new event for the previous state with its start time, end-time, and, if moving, the distance moved during this event.

From these series of events, the various behavior statistics are extracted (see section below about the calculation of the behavior statistics). An optional feature is to check “Recover halting from non-detected events.” If checked, then halting events interrupted by a period of non-detection (due to tracking failure) are recovered and merged into a single halting event. More specifically, for each event-triplet of halting, non-detect, and halting, the start-position of the second halt event should lie within a given radius of the end-position of the first halt event for this merging to occur, otherwise merging cannot take place. The radius-size is defined by the total displacement (i.e., distance between start-point and end-point) within the first halting event. This option is checked by default in order to obtain more reliable data with less non-detect time [not detected (n.d.) was in the range of 10% in these experiments]. It is essential to check the “multiple zones” option to process the data in the format of the T-maze nature of the experiment in which zones can be contrasted against each other.

For various reasons, it may also be necessary to exclude certain types of events for analysis. Minor insect displacements on the zone boundary may cause artificial consecutive halting and moving events. Similarly, movement events containing extreme (unrealistic) velocities can be caused by video tracking hick ups, e.g., when a dirt particle or an optical reflection similar in size with the insect is confused with the insect. EthoAnalysis contains a number of “event filters” which change such events to non-detect events and therewith exclude them from analysis (Figure 2C). The effects of the filters are discussed below.

### EthoAnalysis Filtering of Records

To obtain high-quality data it is important to remove records with insects that are either dead or obviously less active compared to the rest. For this purpose “Record filters” are used to automatically exclude records based on inactivity, detection percentage, and event count (Supplementary Data File 1, Filters tab). When applying these criteria all records are immediately updated and the table shows which records are deleted on which grounds. In the case of this experiment, the following criteria for record removal were applied: >3,600 s inactivity, <50% detection and <1,000 events. Three records were removed based on these settings in trial 3, all on the basis of event count. In experiment 3 there were on average 1,161 halts and 1,569 moves. More moves than halts can occur when insects move from one zone to another: that splits one movement into two parts. Once records are imported it is possible to inspect their quality using a velocity histogram (Figure 1A). This can also serve to guide modifications to the velocity threshold setting. Changing settings requires recalculation which took about 30 s for this experiment with 120 records and 8 h of recording.

### EthoAnalysis Calculation of Behavior Statistics

The behavior statistics (Supplementary Data File 1, Behavior statistics tab) are extracted from the series of behavior events. The software contains a number of behavior statistics that can be extracted for all events of the complete trial, but some of these statistics are also of interest when looking at the statistics per zone, per hour, per zone/hour, or per type of behavior event, e.g., event duration category (short/medium/long) or movement velocity (slow/medium/fast). For the analysis, the robust zone-specified behavior events were selected.

### EthoAnalysis Statistical Analysis

In the experiments, the two genotypes Cur-3 and Rmx-A180 were assessed in multiple trials, where each trial contains multiple choice arenas. Each arena consists of a single leaf of a plant from Cur-3 in one zone, and a single leaf from a Rmx-A180 plant in the other zone. In each trial 4 leafs of 10 plants of either genotype were used and the positioning of leafs in arenas is completely at random.

Suppose that the underlying means of some behavior statistic, such as the duration detected per zone, are μ_*A*_ and μ_*B*_ for Cur-3 and Rmx-A180, respectively, and the observed values in an arena are *y*_*A*_ and *y*_*B*_. Choice experiments, like the one described here, are commonly analyzed by modeling the log ratio $\log\left(\frac{y_{A}}{y_{B}}\right)$ using normal errors, see, e.g., Elston et al. (1996). However, the disadvantage of the log ratio analysis is that it cannot properly handle zero observations. Moreover, observations very close to zero frequently popup as outliers in such analyses. Alternatively, a (conditional) logit model can be used, see, e.g., Hauber et al. (2016). In this approach, the log ratio is rewritten as logit(π_*A*_), where $\pi_{A}=\frac{\mu_{A}}{\left(\mu_{A}+\mu_{B}\right)}$, i.e., the mean for Cur-3 relative to the sum of the means. For behavior statistics, such as duration detected per zone, π_*A*_ can be interpreted as the probability of being in the zone with Cur-3. Information about this parameter is contained in the conditional distribution of *y*_*A*_ given the sum *y*_*A*_+*y*_*B*_, for which it is natural to assume a quasi-binomial distribution resulting in logistic regression.

Both the log ratio analysis model, which is a linear mixed model (LMM), and the logit analysis model, which is a generalized linear mixed model (GLMM), are included in EthoAnalysis. The logit model is implemented by means of an iterative re-weighted restricted maximum likelihood (IRREML) algorithm as proposed by Schall (1991). The LMM within this iterative procedure is fitted using the lmer function in the R package lme4 (Bates et al., 2015).

For the analyses in this paper, the logit model was applied to all behavior statistics to generate predictions of the ratios between means. Confidence intervals of these ratios and *p*-values were constructed using Satterthwaite's degrees of freedom approximation method [implemented in the R package lmerTest (Kuznetsova et al., 2017)]. To account for differences between plants, random effects for plants of Cur-3 and for plants of Rmx-A180 were added to the model. For analyses over multiple trials, a random effect for trial was included as well. The analysis was done using the EthoAnalysis software (version 1.3.0.6) which internally makes use of the lm function of a local installation of R (version 3.1.2) (Supplementary Data File 1, Analysis and Output tabs).

## Results

### Optimizing EthoAnalysis Input and Filter Settings

We performed a sensitivity analysis on various input and filter settings in EthoAnalysis to test how these settings affected the contrasts between both genotypes for the 38 different traits extracted from the tracks (Tables 1, 2). At the basis of each comparison was the optimized F1 analysis. The robustness of that analysis was explored by altering settings one by one and usually in steps of two.

**Table 1:**
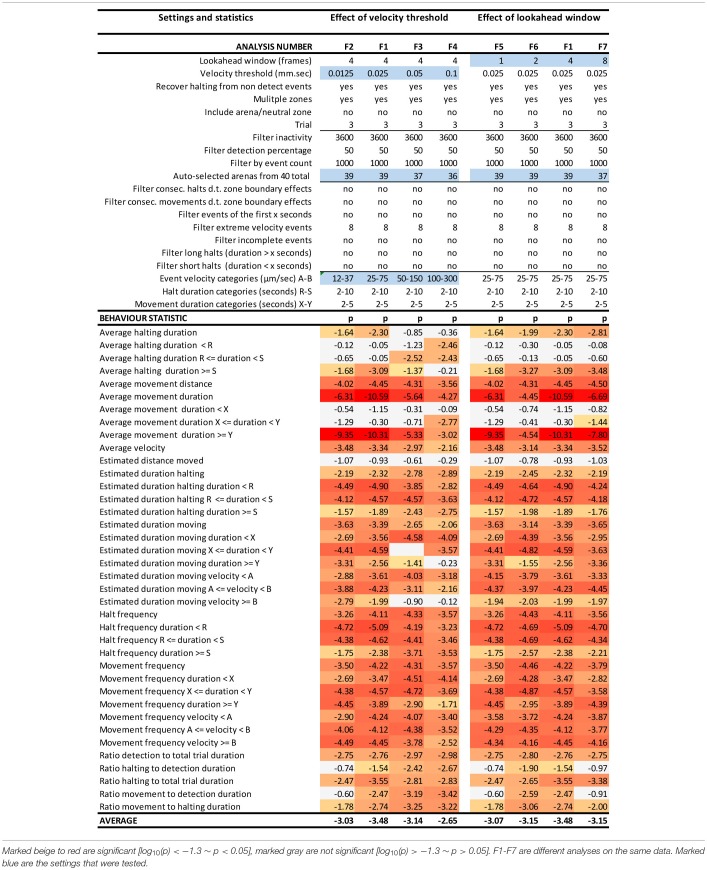
EthoAnalysis settings to evaluate the effect of velocity threshold and look-ahead window on the significance of the statistics [log_10_(*p*)].

*Marked beige to red are significant [log_10_(p) < −1.3 ~ p < 0.05], marked gray are not significant [log_10_(p) > −1.3 ~ p > 0.05]. F1-F7 are different analyses on the same data. Marked blue are the settings that were tested*.

**Table 2:**
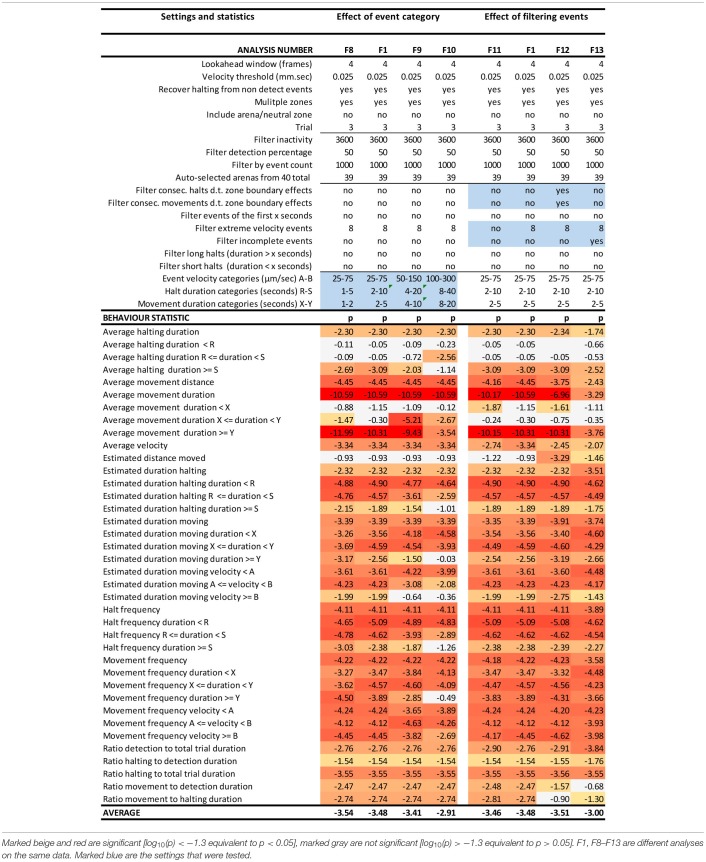
EthoAnalysis settings to evaluate the effect of event categories and event filtering on the significance of the statistics [log_10_(*p*)] in trial 3.

*Marked beige and red are significant [log_10_(p) < −1.3 equivalent to p < 0.05], marked gray are not significant [log_10_(p) > −1.3 equivalent to p > 0.05]. F1, F8–F13 are different analyses on the same data. Marked blue are the settings that were tested*.

#### Effects of the Velocity Threshold and Look-Ahead Window Settings

The look-ahead window and the velocity thresholds that are applied to the velocity trace of each arena jointly determine the classification into moving and halting events. For a first estimation EthoAnalysis provides frequency histograms of all measured velocities per individual insect (Figure 2A; Supplementary Data File 1). From the camel shape of most histograms the velocity separating halting and moving behavior can roughly be deduced (Figure 2A). Most commonly at the dip of the camel shape we observed a velocity of around 0.05 mm/s. To explore the sensitivity of the system we also evaluated thresholds that were both higher (0.1 mm/s) and lower (0.025 and 0.012 mm/s) in steps of 2. The velocity threshold setting was also affecting the number of selected records (Table 1). Inspection showed that at least three velocity tracks were indeed poor and correctly deleted from the analysis. The table contains 38 parameters related to the recorded movement and halting events. The results of the statistical analysis are given in the table with the red/yellow color indicating various degrees of significance [log_10_(*p*)]. The table shows how the velocity threshold affects the significance of certain parameters more or less strongly. Often less significance in one parameter is complemented with more significance in another one. The most significant *p*-values (~10^−10^) were found at velocity threshold 0.025 mm/s for average movement distance and duration (F1 vs. F2–4).

The setting of the look-ahead window around 4 was subsequently explored by also testing 1, 2 and 8 frames-ahead of an original one (time window of 0.3–2.4 s). Both the longer and shorter look-ahead windows yielded results quite similar to a look-ahead window of 4 frames but on average 4 frames was optimal (Table 1, F1 vs. F6, F7).

#### Effect of Event Categories on Movement and Halting Duration, Frequency and Velocity

Kloth et al. (2017) have shown with aphids that it is insightful to subdivide halt events into event categories of different duration to specifically investigate the more frequent test probing phase that is interrupted (<3 min) separate from the less frequent succesful probing phase that continues into sustained feeding (>25 min). In EthoAnalysis this type of detailed analysis of behavior is accessible for both halting and moving events. Short, medium and long intervals can be defined by setting two time thresholds that separate short and medium and medium and long and which can be evaluated in terms of average and total duration and frequency. In the case of Western flower thrips, which is a frequently moving species, 2 and 10 s for halts and 2 and 5 s for movements appeared optimal for creating the most significant behavior distinctions between both accessions. Velocity categories were tested and found to yield relevant differences at the thresholds of 0.025 mm/s and 0.075 mm/s (Table 2, F1 vs. F8-F10).

#### Effect of Event Filters

Due to the strict assignment of all events to specific zones in two-choice assays, every moving/halting event terminates when the subject leaves a designated zone even though the movement continues. This leads to about 30–40% more moves than stops in the F1 settings of this experiment. The first event filter we applied was a filter for extreme velocity events that result from tracking artifacts. This removed 10% of all moves in experiment 3. Table 2 (F11 vs. F1) shows that filtering out those events improved the statistics related to movements a little bit. Filtering out consecutive halts and movements due to zone boundary effects made little overall difference, but strongly reduced significance of the best statistic of average movement duration (F12 vs. F1). This can be explained by the fact that the highest speeds and longest distances and moves to other zones are generated on the most resistant genotype. Removing those zone transition data actually creates a bias toward “normal” behavior to accept the genotype for feeding and strongly reduces the number of events on the resistant genotype with resultant decreases in significance of the contrast. A similar and even larger effect is observed by the option to filter out “incomplete events” (F13 vs. F1), which are events that are interrupted by a non-detect state.

### Different Behavior Statistics in a Choice Arena

**Figure 3:**
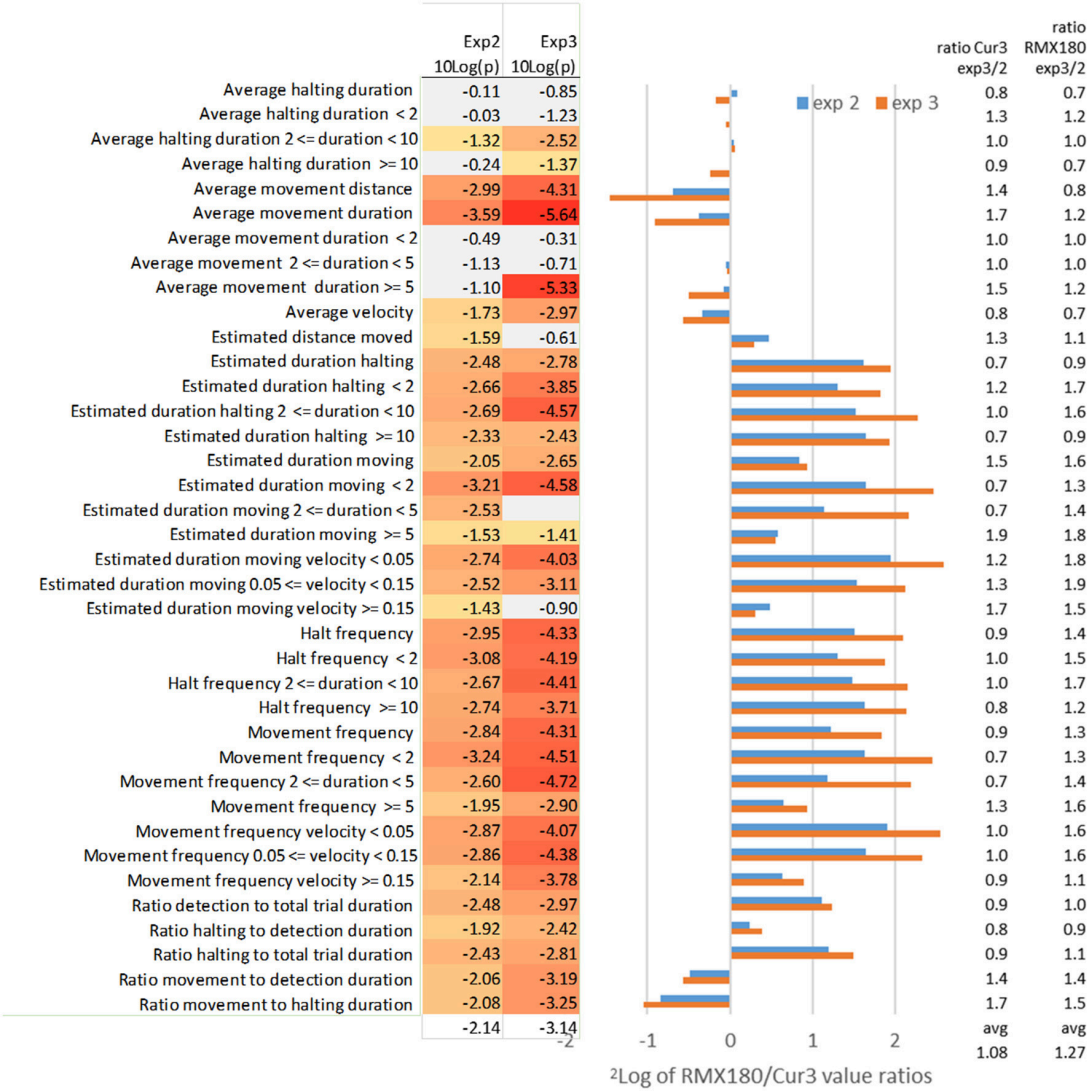
Effect of independent experimental replication (experiments 2 and 3) on the log2 of RMX180/Cur3 value ratios and the value ratios across experiments per genotype and on the significance of the statistics (*N* = 39–40). Marked in red are significant [log_10_(*p*) < −1.3 ~ *p* < 0.05], marked gray are not significant [log_10_(*p*) > −1.3 ~ *p* > 0.05]; the settings of analysis F3 (Tables 1, 2) were used to compare both experiments.

Using the optimized settings above we compared two independent experiments in Figure 3 for an impression of the variation between genotypes and between experiments in the values of different statistics and their significance.

#### Average Movement Duration, Velocity and Distance

The average duration of movement events is the most distinctive feature of thrips behavior between these two genotypes with highly significant scores of *P* = 10^−10^ in the best experiment 3 with 39 samples (Tables 1, 2). Comparing this with the independent experiment 2 (using the same F3 settings for both experiments) here also average movement duration yielded the best significance score. Interestingly the contrasts for this movement duration trait differed less than a factor 2: apparently the frequent measurement at every move creates a very reliable average for each arena (Figure 3). In experiment 3 the significant differences appeared to reside mostly in the >5 s moves which were dominant in the resistant Cur-3 genotype, but this relationship was not visible in experiment 2. This may be due to the fact that arena averages were based on a very high number of ca 200 movement events per hour which are obviously reduced when the data are split into the three subcategories (Figure 3). The average distance of movement events was also a highly significant feature of thrips behavior. Movement duration and distance are a function of velocity, but the average velocity differed less strongly between the genotypes (30–50%) than duration and distance. Yet, it is interesting to note that the insects were moving faster on average on the resistant genotype.

#### Estimated Duration Moving

By contrast the total estimated duration moving (corrected for detection) has less significant results for the aggregated results compared to some of the subcategories (frequency, velocity and distance) of this moving duration statistic (Figure 3). The aggregated total duration moving is less than a factor 2 different between both genotypes whereas the subcategories with the <2 and 2–5 s intervals are in the range of a factor 2–5 (Figure 3). The likely reason for this higher significance in these subcategories is that the durations are not averaged but totalled, and thus also reflect the choice between genotypes. Apparently, then, the moves of <5 s, which are associated with feeding on the preferred genotype, and not related to searching, are the most significant. This is also reflected in the statistic “estimated duration moving (at a certain) velocity (threshold).” Here the moving events are not totalled on the basis of their duration but their velocity instead. This yields almost a 6-fold difference between the genotypes for the slowest speeds of < 0.05. These speeds are clearly associated with movements between cells during feeding: 50 μm per second is exactly in the range of the size of an epidermal cell. Most events were < 5 s, based on the other statistics, so the range of movement was not more than 250 μm much less than the length of the insect (2 mm).

#### Estimated Distance Moved

Despite the fact that average movement distance differed significantly between both genotypes, the estimated distance moved did not. The *average* distance moved on the resistant Cur-3 genotype was much longer but more *total* distance was covered on the susceptible genotype simply because more time was spent there.

#### Movement Frequency Duration and Velocity

The movement frequency is a way of describing movement events not in terms of duration or velocity, but simply in terms of the number of events. It represents just a subtle variation on the other statistics but assumes neutrality to the values and provides similar or more significant values. Also here the subcategories of frequency in terms of the duration and velocity of the events are given in Figure 3. The frequency of movements of short duration (<5 s) and low speed (<0.15 mm/s) are most significantly different.

#### Average and Estimated Halting Duration and Frequency

In strong contrast to average movement duration, the average halting duration, also when split into duration categories, is hardly or not significantly different between both genotypes (Figure 3). In the T-maze the thrips can choose either genotype for halting/feeding. Feeding on the resistant Cur-3 accession apparently follows the same behavior pattern except that a lower proportion of the total time is spent on feeding there (3- to 4-fold less, Figure 3). This effect of choice and uniformity of halting behavior on both genotypes is visible as well in the halting frequency for different durations: 3- to 4-fold differences are seen with similar significances and ratios for all categories in both experimental replications (Figure 3).

#### Ratio Detection and Halting to Total Trial Duration

The ratio of detection of the thrips individual relative to total trial duration represents the choice for activities (moving and halting) on either accession and a conventional way of studying relative resistance looking at preference. The ratio of detection is 2-fold more on the susceptible accession and the significance is reasonable but not as good as reported by the more specific statistics (Figure 3). Taking out only the component of halting (excluding moving) does not improve the result (Figure 3).

#### Ratio Halting and Movement to Detection Duration

Halting and movement to detection duration split up the comparison of time detected inside a zone into either halting or moving. The statistics show that on the susceptible genotype Rmx-A180 a slightly higher proportion of time is spent on halting and a slightly lower proportion on moving (Figure 3).

#### Ratio Movement to Halting Duration

Potentially this statistic ratio of movement and halting amplifies the differences between two tested genotypes and indeed compared to movement to detection and halting to detection the differences are combined and larger (around a factor 2). However, the significance of the difference is not better compared to the individual statistics so that this ratio statistic is also not as useful as some of the others offered above (Figure 3).

### Western Flower Thrips Behavior in Time

**Figure 4:**
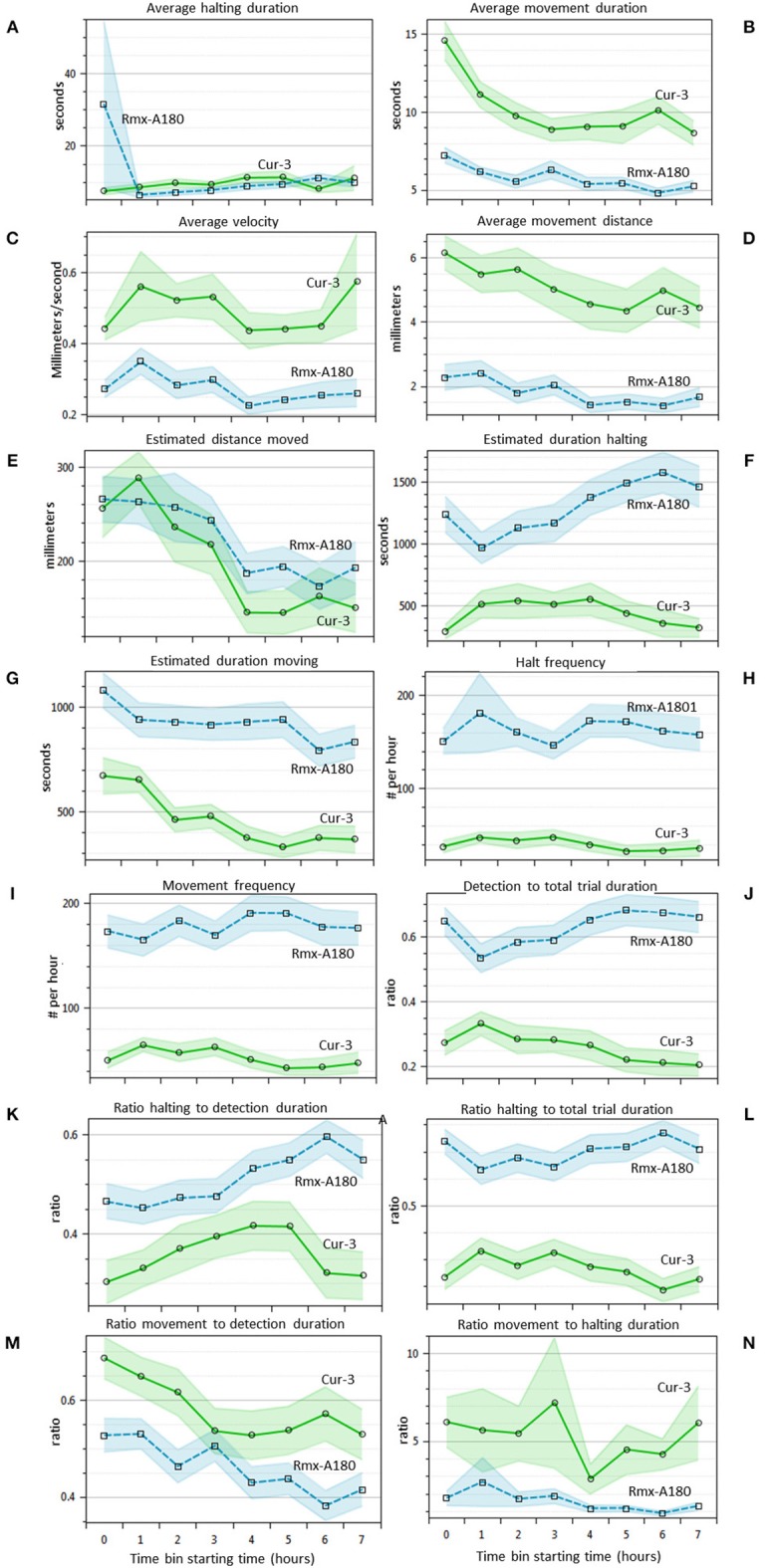
Thrips choice behavior statistics in time **(A–N)**. Overview of 14 different statistics that are automatically indexed per hour by EthoAnalysis. The 95% confidence intervals are indicated by blue and green shading (excluding effects of experimental design). The results are given for experiment 3 (analysis F1) and are based on 39 arenas. Estimated values are per hour, corrected for the percentage detection (except frequency), and log-transformed for averaging. The average detection level was ~90% in this experiment.

For 14 of the statistics above the EthoAnalysis software also offers a time course analysis in time bins of 1 h. This feature is very useful for a quick insight into how the trait values and significance change in time. Figure 4 shows that most of the 14 statistics have 95% confidence intervals (not taking experimental design into account) that do not overlap at some or all points in time during the experiment. Some obvious trends are that the average movement duration and distance decrease in time for both genotypes. The estimated duration spent halting and detection and halting to total trial duration on the other hand clearly diverge. Apparently, thrips develop a growing preference for Rmx-A180 over time.

### Correlation of Behavior Statistics Within Experiments

**Figure 5:**
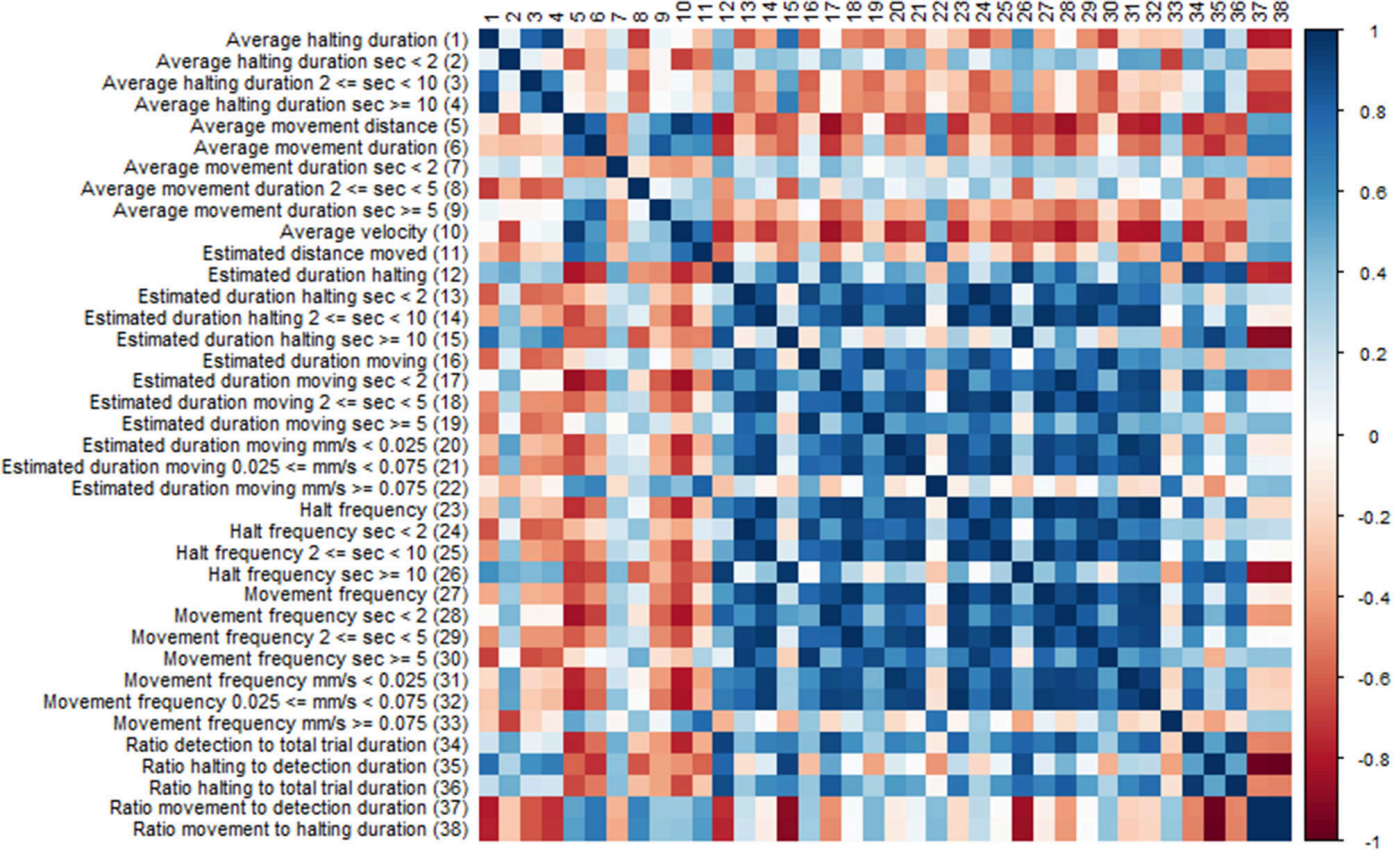
Correlation diagram of behavior statistics. Colors indicate spearman *Rho* correlation values, where blue boxes indicate positive correlations and red boxes negative correlations between traits.

Many of the EthoAnalysis behavior statistics are likely dependent on each other to some degree, and the purpose of looking at many is to determine which one across independent experiments reproducibly best describes the genotype differences. Both dependence and independence may have causes in genes (both plant and insect) or environment (assay quality, insect age) or assay set up (choice vs. no-choice). To test this, the software automatically generates Spearman correlation tests. We are showing the results for statistics obtained for Zone 2 (Rmx-A180) (Figure 5) in the EthoAnalysis software. This diagram of the correlation between the statistics of all reported traits for the entire 8 h of the recording shows how specific thrips behavior parameters correlate negatively (red boxes), positively (blue boxes), or not (white boxes), within zone 2.

Immediately obvious is the large blue square of positively correlated parameters of estimated movement and halt frequency duration and velocity. Exceptions are the estimated duration at high velocity which correlates only with long distance moved and the frequency of long halts which correlates with long duration and correlates negatively with the ratio movement to halting duration. Average velocity appears to correlate negatively with most traits except average movement distance and long fast moves.

The effect of assay set up on correlations found should be considered as well. The fact that the insects can choose their host has a strong effect on some of the ratios found depending on whether they represent average or cumulative/frequency data. The choice for the susceptible genotype will increase both the total movement and halting time on that genotype but not necessarily the average time of the events.

## Discussion

We have developed and evaluated a T-maze array in the novel EntoLab video tracking and behavior analysis system. The system makes use of dedicated hardware consisting of 90 parallel T-mazes to which individual insects like thrips can be admitted simultaneously by opening a gate plate. The heated cover plate was essential to allow prolonged recordings without the appearance of condensation droplets. We recorded the behavior of the insects on video and subsequently performed video tracking and computation of position and speed using EthoVision software. Exported tracks were analyzed by EthoAnalysis (Supplementary Data File 1). Both the hardware and software represent a large improvement relative to the use of microtiter plates and the previous time-consuming data extraction and manual statistical analysis (Thoen et al., 2016). In the new set up, with some experience, a powerful statistical analysis of multiple experiments including some optimization steps can now be done in 10–20 min whereas before *ad hoc* R scripts were written taking months to learn and carry out to obtain a similarly detailed output that we deem relevant to describe insect behavior (Table 3). With different insect species it will be easy to modify the size of the T-mazes to properly accommodate the size of the insects tested as was recently described for parasitic wasps for example (De Bruijn et al., 2018).

**Table 3:** Features of EthoAnalysis for analyzing insect behavior.

Event determination	Post-analysis • adjusting event thresholds is done on raw track data, this takes 10–30 s for an 8 h movie depending the total number of arenas
Behavior parameters	Choice (= detection/total trial duration) Velocity Halting duration + 3 subcategories duration Halting frequency + 3 subcategories duration Moving duration + 6 subcategories velocity and duration Moving frequency + 6 subcategories velocity and duration Moving distance Event duration halting + 3 subcategories duration Event duration moving + 3 subcategories duration Event distance moved Halting/total trial duration Halting/detection duration Movement/halting duration Movement/detection duration 38 statistics (14 in time bins)
Data filter	• Automatic track filtering on inactivity period, detection percentage, and event count with threshold settings • Broad variety of event filters. Allows event filters based on duration of events, consecutive events due to zone transitions and velocity.
Detection dependency	Robust statistics that are corrected for the percentage of detection per subject
Statistics	Built-in statistical package and default transformations on data
Graphical visualization of data	Summary tables, graphs per trait, visualization of statistical models used
Post-recording video/track interaction	Fully interactive with a 2D visual tracking trace corresponding to the arena shape for the selected time period and with a corresponding 1D velocity trace with velocity threshold and a color coded assignment of movement, halting events and zone position. All with immediate feedback of applied filtering and detection success (red or gray).

EthoAnalysis significantly extends the capabilities of EthoVision software for plant phenotyping studies (Table 3). Firstly, it also analyzes behavior statistics at the event level. These cannot be obtained directly from EthoVision, but are shown here to be the most reliable indicators of plant resistance to thrips. Secondly, filters can be applied in EthoAnalysis that automatically remove entire records or events with poor data, so that more reliable records remain. EthoVision only allows manual filtering of entire tracks based on visual inspection of deviant records. EthoAnalysis can also redefine input thresholds without the need to re-run the entire recording, as with EthoVision. This saves much time when settings must be adjusted to find the optimum. Post-recording 1D and 2D track and zone visualizations are available including the event assignments at any desired zoom level providing feedback on the effects of specific statistic settings on the assigned behavior in the recorded track (Table 3). We extracted 38 statistics in three overarching behavior categories of choice, halting behavior and movement behavior. In the given example these behavior parameters related to host-plant acceptance, and comprised of both dependent and independent statistics with different qualities to describe the difference between the genotypes. Eighteen of these statistics are available in time bins of 1 h as well (Table 3). EthoAnalysis also includes a built-in software package based on R, that properly transforms data of multiple experiments and applies the appropriate statistical models to evaluate behavior parameters, which are visualized in graphs (Supplementary Data File 2, Table 3).

A relevant question is whether one needs 38 partly dependent statistics to describe the behavior on these two plant genotypes. We think it is very helpful for two reasons: First of all, from these statistics a highly detailed time-resolved view emerges of how thrips is behaving in the T-maze on the combination of genotypes. Secondly, the most powerful statistics can be used to analyze large populations much more efficiently. In a high-throughput setting one would wish to reduce the number of replications and also the observation time (for example only 2 h and 10 insects per genotype). In such a setting it becomes critical to have access to the most informative statistic. In some cases such statistic information may be obtained in advance for crossing populations by pretesting the parents, but in the case of GWAS populations and also many crossing populations it will generally be a trial and error testing of all statistics to find which one best generates a particular QTL. This may require multiple testing corrections of the associated gene found, if all traits are given equal weight in quantitative genetic studies. One way to successfully overcome this with multivariate phenotypes like insect behavior, would be to map summary statistics or values generated through dimensional reduction methods, like principle component analyses or discriminant analyses (Horton et al., 2014).

We can evaluate the way in which the event-based analysis provides access to the detailed feeding behavior of thrips. A single feeding event of thrips can be divided in five consecutive steps: (1) Placing the tip of the mouth cone on the cell surface; (2) Thrusting the mandible through the plant surface layers; (3) Inserting the maxillary stylets into the cavity created by the mandible intrusion; (4) Sucking of the contents of punctured cells; (5) Retracting stylets and lifting the mouth cone (Kindt et al., 2003, 2006). Step 3, in which maxillary stylets are inserted into the created cavity is considered the start of a probing event, where cell contents are evaluated by the thrips. Only if this test probe is satisfactory, step 4 (the sustained sucking of contents) will follow. Feeding events where this last step does not follow, are thus likely not real feeding events, but just probing events. These “test probes” might occur more frequently when plant material is of suboptimal quality for thrips. Given that these probes were reported to generally take <10 s (Lewis, 1997), we thought that the relative duration of short and long probes could serve as a proxy for host plant suitability. However, in this choice assay the halting event durations were not very different and only the frequencies were strongly different due to the preference difference. Apparently, the free choice situation created “normal” feeding behavior (halting time) on both accessions. We expect, though, that this could be different in non-choice assays, because in that case the total time on each genotype is identical.

The selected settings were used to characterize two experiments with the *Arabidopsis* accessions Cur-3 and Rmx-A180. The value ratios of the two replicated experiments on different days delivered qualitatively very similar results, but quantitatively exhibited differences in both the absolute values and value ratios. Apparently, there are environmental batch effects between consecutive experimental days that can lead to such differences, and it will be crucial in complex experiments to deal with that. In an application of the EntoLab system for association genetics by screening large populations, proper assay design and mathematical treatment of these variations in values and ratios will be crucial for high quality results. High frequent alleles in a plant panel will to some extent compensate for experimental variation, but obviously it will always be important that the experimental design and analysis method should minimize these effects (Kloth et al., 2017). Future studies for application in plant genetics should, therefore, concentrate on best practices to minimize or correct for such variation. One approach could be to achieve complete block design on each plate. The current set up can contain 90 genotypes once and 10 plates could be tested to obtain 10 replications per accession for example. Yet with larger populations incomplete block design will be necessary. In choice assays such modeling approaches need to take into account genotype × genotype interactions, however, that can deal with a situation in which the reference genotype can both be relatively “susceptible” and “resistant” compared to the contrasted reference.

Heritability is essential in quantitative genetic studies. If biological variation arises from genetic or environmental effects, stochastic effects are classified as environmental because they are not passed on to offspring. But non-heritable effects can be subdivided into those which can be predicted from measurable variables, and those that cannot, e.g., stochastic effects (Honegger and De Bivort, 2018). Reducing the amount of stochasticity in spontaneous behavior might greatly increase data quality. Data output from EntoLab can be subsequently analyzed in dynamic models that can accurately capture stochasticity, non-linearity and non-stationary behavior transitions from active to non-active (Melanson et al., 2017).

Potentially, long recordings also contain information on changes in thrips preference over time, due to resistance mechanisms that take a few hours to establish their effects on thrips, or resistance mechanisms that require induction by herbivory before defense pathways are activated. An example of “slow-acting” defense compounds are protease inhibitors, which can take at least 4 h to result in a significant effect on thrips choice behavior (Outchkourov et al., 2004). Thrips induced defenses are mediated by jasmonic acid and trigger the metabolism of a wide array of defensive compounds, but these can take 24 h to fully establish (De Vos et al., 2005; Abe et al., 2008, 2009). If such time-resolved data will be mapped onto plant genomes one would expect the associated QTLs to change in the course of hours, providing additional insight into the resistance mechanisms at work. Alternatively in screenings this induction period could be mimicked with a prior treatment with jasmonic acid so that data relevant to induced plants are obtained.

## Concluding remarks

EntoLab is a promising new instrument for high-throughput phenotyping insect behavior on plants and other substrates. We have so far validated the setup in either choice or no-choice assays for different species of thrips, aphids and whitefly (*Frankliniella occidentalis, Thrips tabaci, Myzus persicae, Brevicoryne brassicae, Nasonovia ribisnigri, Aphis gossypii, and Bemisia tabaci)* in various combinations on pepper, tomato, water melon, chrysanthemum, white cabbage, lily, lettuce, and bitter gourd. A paper describing results with no-choice assays of *N. ribisnigri* on whole leaves of lettuce is in preparation. We expect the potential use of the EntoLab system to extend beyond the assay of plant material and herbivores. Different arena designs, insect sizes and test samples can be easily implemented to fit the universal arena plate holder as was recently shown in a case study on learning and memory retention in parasitic wasps (De Bruijn et al., 2018). We hope that the unlocking of behavioral details that now often go unnoticed will in the future lead to more insight into the environmental and genetic mechanisms that control it.

## Data Availability Statement

The raw and processed data supporting the conclusions of this manuscript will be made available by the authors, without undue reservation, to any qualified researcher. The EthoVision and EthoAnalysis software can be obtained with a time-limited trial license. The entire EntoLab set up can be obtained from Noldus Information Technology BV (www.noldus.com/entolab).

## Author Contributions

MJ and MT wrote the paper. MJ designed the hardware and supervised the research. LP and GW carried out the experiments. PG designed the method for the statistical analysis. MD and LN supervised the research and cooperation. JK wrote the EthoAnalysis software. All authors have read and approved the paper.

### Conflict of Interest Statement

The hardware and software of the EntoLab system will be marketed jointly by Noldus Information Technology BV and Wageningen Plant Research. This will promote the distribution and use of the system, but does present a potential commercial and financial conflict of interest.

## Supplementary Material

Supplementary Data File 1 EthoAnalysis screen capture of all tabs.

Supplementary Data File 2 Automatically generated statistical analysis by the EthoAnalysis software using the F1 settings. This represents the most extensive format. Optional are reports with only the main results.
