# Supplementary material for: An Integrated System for the Automated Recording and Analysis of Insect Behavior in T-maze Arrays
Source: Front Plant Sci. 2019 Jan 29;10:20. doi: 10.3389/fpls.2019.00020 (PMC6361829; doi:10.3389/fpls.2019.00020)
Supplement: Supplementary Data File 1 — EthoAnalysis screen capture of all tabs. [file Data_Sheet_1.PDF]

Supplementary Material

EntoVision™ hardware and software for the automated analysis of thrips behaviour recorded by videotracking

Maarten A. Jongsma, Manus P.M. Thoen, Leo M. Poleij, Gerrie L. Wiegers, Marcel Dicke, Lucas P.J.J. Noldu3, Johannes Kruisselbrink1

*** Correspondence: maarten.jongsma@wur.nl**

Supplementary data 1. EthoAnalysis screen capture of all tabs

EthoAnalysis: Project tab

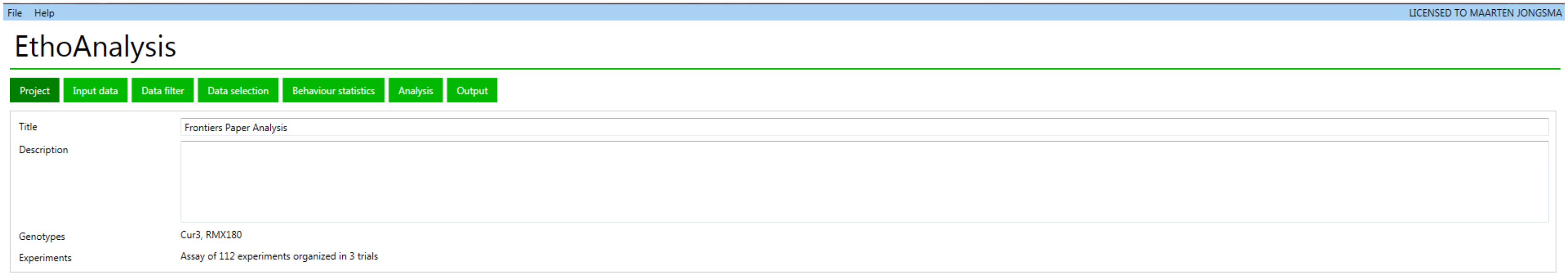

EthoAnalysis: Input data tab

File Help LICENSED TO MAARTEN JONGSMA

EthoAnalysis

Project | Input data | Data filter | Data selection | Behaviour statistics | Analysis | Output

Add/remove data files

Add | Remove selected

Import settings

Look-ahead window: 2 Frames

Velocity threshold: .025

- [x] Recover halting from non-detect events
- [x] Multiple zones
- [ ] Include arena / neutral zone

Open import dialog | Save

Zones

Zone 1
Zone 2
Zone 3

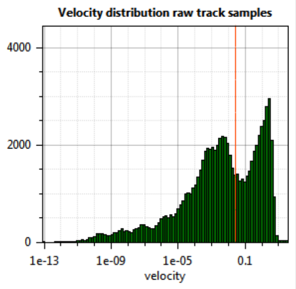

Select	Filename	Tracking	IsLoaded
[ ]	..\Track-Frankliniella-Arabidopsis-Trial 1-1-Subject 1.txt	3.33	True
[ ]	..\Track-Frankliniella-Arabidopsis-Trial 1-10-Subject 1.txt	3.33	True
[ ]	..\Track-Frankliniella-Arabidopsis-Trial 1-11-Subject 1.txt	3.33	True
[ ]	..\Track-Frankliniella-Arabidopsis-Trial 1-12-Subject 1.txt	3.33	True
[ ]	..\Track-Frankliniella-Arabidopsis-Trial 1-13-Subject 1.txt	3.33	True
[ ]	..\Track-Frankliniella-Arabidopsis-Trial 1-14-Subject 1.txt	3.33	True
[ ]	..\Track-Frankliniella-Arabidopsis-Trial 1-15-Subject 1.txt	3.33	True
[ ]	..\Track-Frankliniella-Arabidopsis-Trial 1-16-Subject 1.txt	3.33	True
[ ]	..\Track-Frankliniella-Arabidopsis-Trial 1-17-Subject 1.txt	3.33	True
[ ]	..\Track-Frankliniella-Arabidopsis-Trial 1-18-Subject 1.txt	3.33	True
[ ]	..\Track-Frankliniella-Arabidopsis-Trial 1-19-Subject 1.txt	3.33	True
[ ]	..\Track-Frankliniella-Arabidopsis-Trial 1-2-Subject 1.txt	3.33	True
[ ]	..\Track-Frankliniella-Arabidopsis-Trial 1-20-Subject 1.txt	3.33	True
[ ]	..\Track-Frankliniella-Arabidopsis-Trial 1-21-Subject 1.txt	3.33	True
[ ]	..\Track-Frankliniella-Arabidopsis-Trial 1-22-Subject 1.txt	3.33	True
[ ]	..\Track-Frankliniella-Arabidopsis-Trial 1-23-Subject 1.txt	3.33	True
[ ]	..\Track-Frankliniella-Arabidopsis-Trial 1-24-Subject 1.txt	3.33	True
[ ]	..\Track-Frankliniella-Arabidopsis-Trial 1-25-Subject 1.txt	3.33	True
[ ]	..\Track-Frankliniella-Arabidopsis-Trial 1-26-Subject 1.txt	3.33	True
[ ]	[illegible]	[illegible]	[illegible]

- [ ] Select all/none

Trial	Arena	Genotype Zone 1	Plant Zone 1	Leaf Zone 1	Genotype Zone 2	Plant Zone 2	Leaf Zone 2	Genotype Zone 3	Data file
Trial 1	2	Cur3	2	4	RMX180	9	4	Neutral	..\Track-Frankliniella-A
Trial 1	3	Cur3	9	4	RMX180	5	4	Neutral	..\Track-Frankliniella-A
Trial 1	4	Cur3	8	4	RMX180	1	4	Neutral	..\Track-Frankliniella-A
Trial 1	5	Cur3	3	4	RMX180	8	4	Neutral	..\Track-Frankliniella-A
Trial 1	6	Cur3	6	4	RMX180	7	4	Neutral	..\Track-Frankliniella-A
Trial 1	7	Cur3	7	4	RMX180	4	4	Neutral	..\Track-Frankliniella-A
Trial 1	8	Cur3	5	4	RMX180	3	4	Neutral	..\Track-Frankliniella-A
Trial 1	9	Cur3	10	4	RMX180	6	4	Neutral	..\Track-Frankliniella-A
Trial 1	10	Cur3	7	3	RMX180	8	3	Neutral	..\Track-Frankliniella-A
Trial 1	11	Cur3	5	3	RMX180	5	3	Neutral	..\Track-Frankliniella-A
Trial 1	12	Cur3	6	3	RMX180	6	3	Neutral	..\Track-Frankliniella-A
Trial 1	13	Cur3	9	3	RMX180	10	3	Neutral	..\Track-Frankliniella-A
Trial 1	14	Cur3	2	3	RMX180	2	3	Neutral	..\Track-Frankliniella-A
Trial 1	15	Cur3	1	3	RMX180	4	3	Neutral	..\Track-Frankliniella-A
Trial 1	16	Cur3	10	3	RMX180	9	3	Neutral	..\Track-Frankliniella-A
Trial 1	17	Cur3	3	3	RMX180	3	3	Neutral	..\Track-Frankliniella-A
Trial 1	18	Cur3	4	3	RMX180	7	3	Neutral	..\Track-Frankliniella-A
Trial 1	19	Cur3	8	3	RMX180	1	3	Neutral	..\Track-Frankliniella-A
Trial 1	20	Cur3	10	2	RMX180	10	2	Neutral	..\Track-Frankliniella-A
Trial 1	21	Cur3	9	2	RMX180	2	2	Neutral	..\Track-Frankliniella-A

EthoAnalysis: Data filter tab

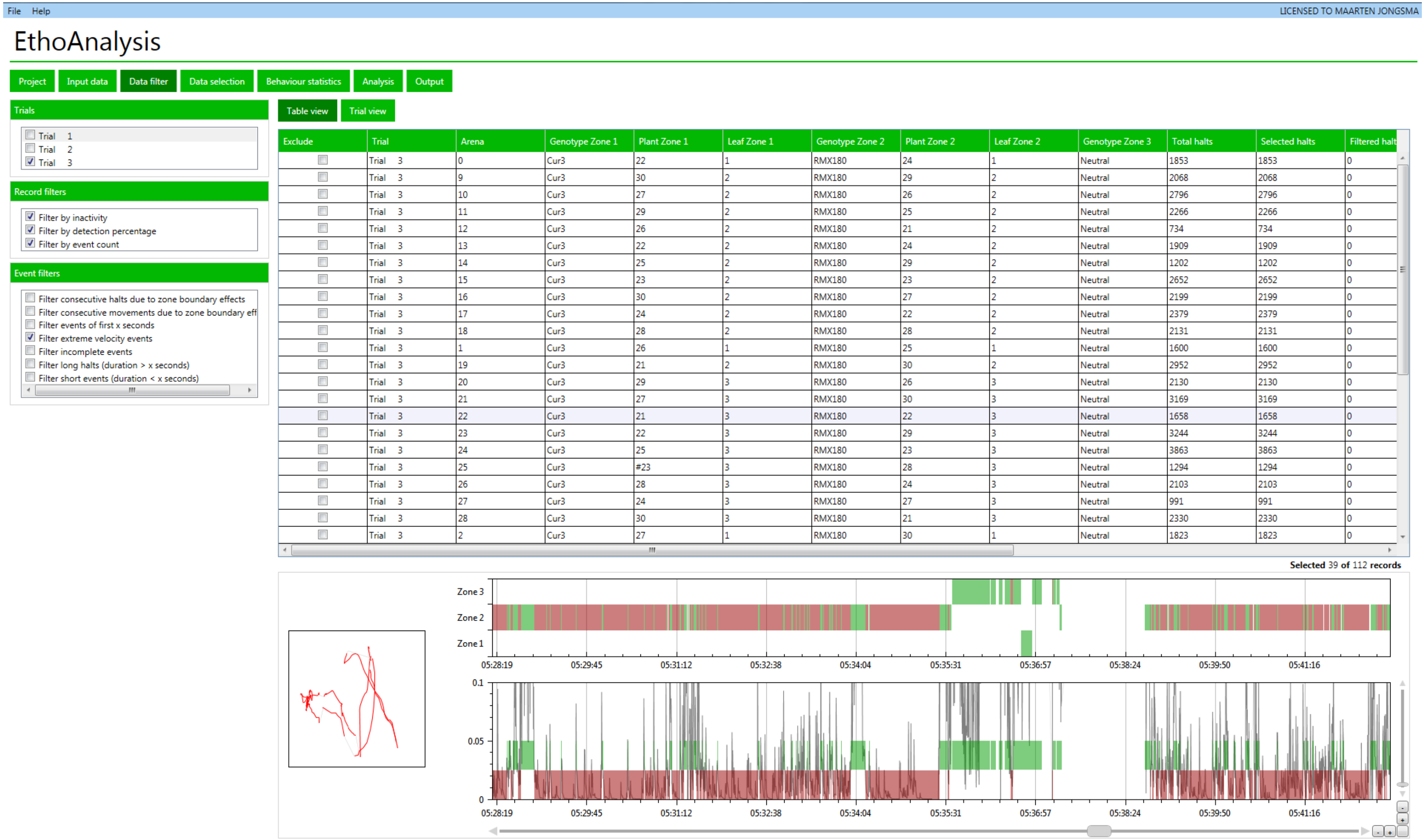

Exclude	Trial	Arena	Genotype Zone 1	Plant Zone 1	Leaf Zone 1	Genotype Zone 2	Plant Zone 2	Leaf Zone 2	Genotype Zone 3	Total halts	Selected halts	Filtered halt
	Trial 3	0	Cur3	22	1	RMX180	24	1	Neutral	1853	1853	0
	Trial 3	9	Cur3	30	2	RMX180	29	2	Neutral	2068	2068	0
	Trial 3	10	Cur3	27	2	RMX180	26	2	Neutral	2796	2796	0
	Trial 3	11	Cur3	29	2	RMX180	25	2	Neutral	2266	2266	0
	Trial 3	12	Cur3	26	2	RMX180	21	2	Neutral	734	734	0
	Trial 3	13	Cur3	22	2	RMX180	24	2	Neutral	1909	1909	0
	Trial 3	14	Cur3	25	2	RMX180	29	2	Neutral	1202	1202	0
	Trial 3	15	Cur3	23	2	RMX180	23	2	Neutral	2652	2652	0
	Trial 3	16	Cur3	30	2	RMX180	27	2	Neutral	2199	2199	0
	Trial 3	17	Cur3	24	2	RMX180	22	2	Neutral	2379	2379	0
	Trial 3	18	Cur3	28	2	RMX180	28	2	Neutral	2131	2131	0
	Trial 3	1	Cur3	26	1	RMX180	25	1	Neutral	1600	1600	0
	Trial 3	19	Cur3	21	2	RMX180	30	2	Neutral	2952	2952	0
	Trial 3	20	Cur3	29	3	RMX180	26	3	Neutral	2130	2130	0
	Trial 3	21	Cur3	27	3	RMX180	30	3	Neutral	3169	3169	0
	Trial 3	22	Cur3	21	3	RMX180	22	3	Neutral	1658	1658	0
	Trial 3	23	Cur3	22	3	RMX180	29	3	Neutral	3244	3244	0
	Trial 3	24	Cur3	25	3	RMX180	23	3	Neutral	3863	3863	0
	Trial 3	25	Cur3	#23	3	RMX180	28	3	Neutral	1294	1294	0
	Trial 3	26	Cur3	28	3	RMX180	24	3	Neutral	2103	2103	0
	Trial 3	27	Cur3	24	3	RMX180	27	3	Neutral	991	991	0
	Trial 3	28	Cur3	30	3	RMX180	21	3	Neutral	2330	2330	0
	Trial 3	2	Cur3	27	1	RMX180	30	1	Neutral	1823	1823	0

EthoAnalysis: Data selection tab

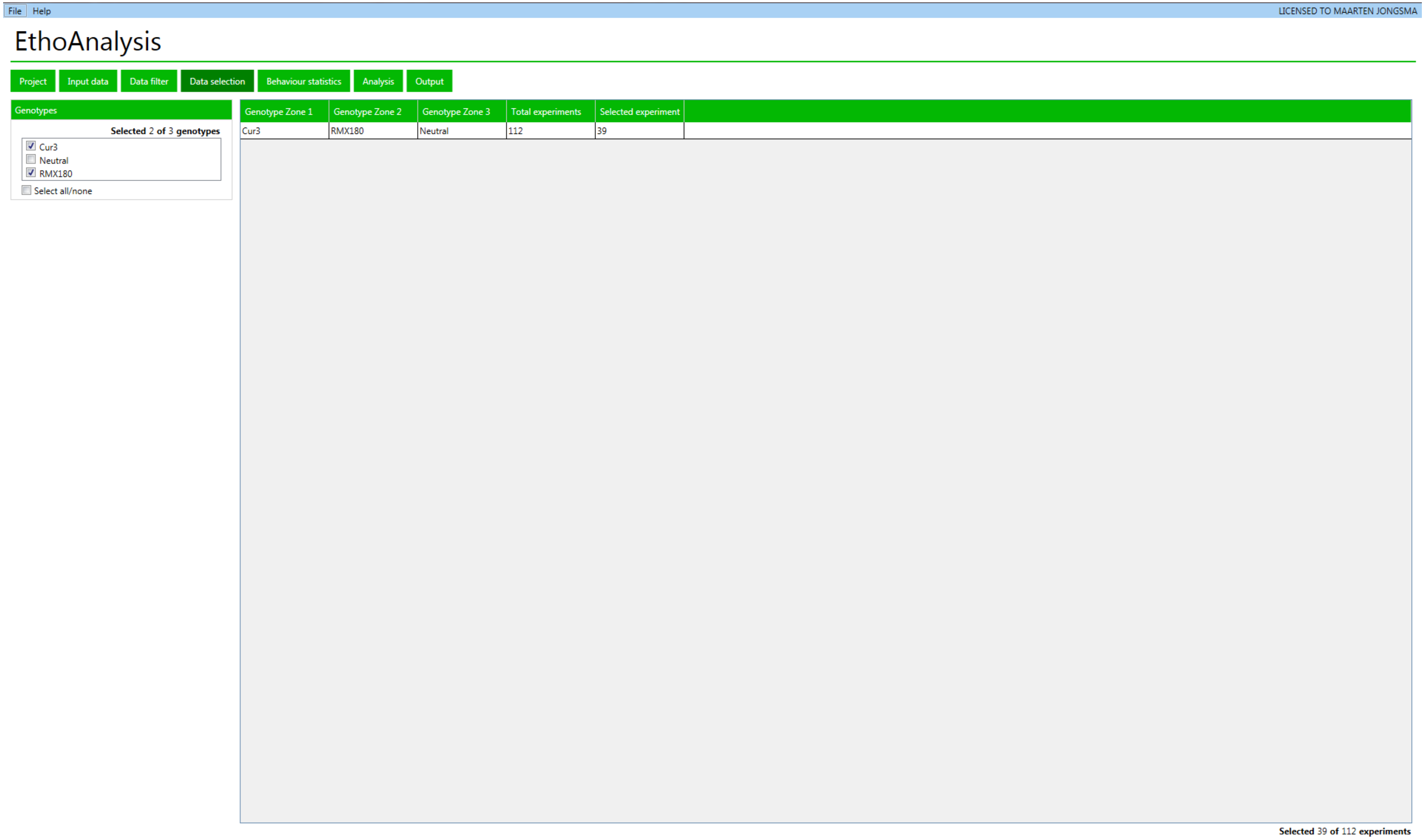

EthoAnalysis: Behaviour statistics tab

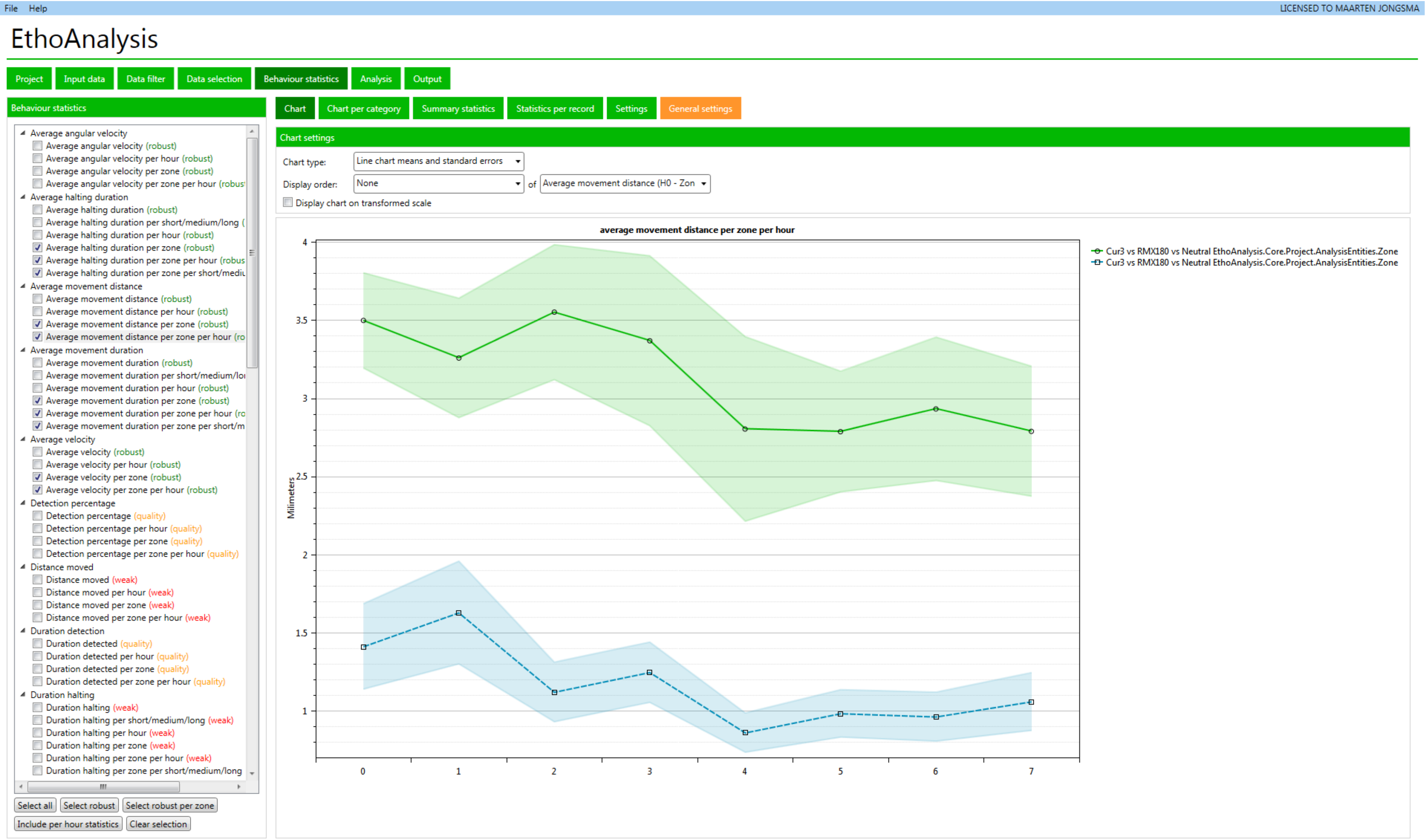

EthoAnalysis: Analysis tab

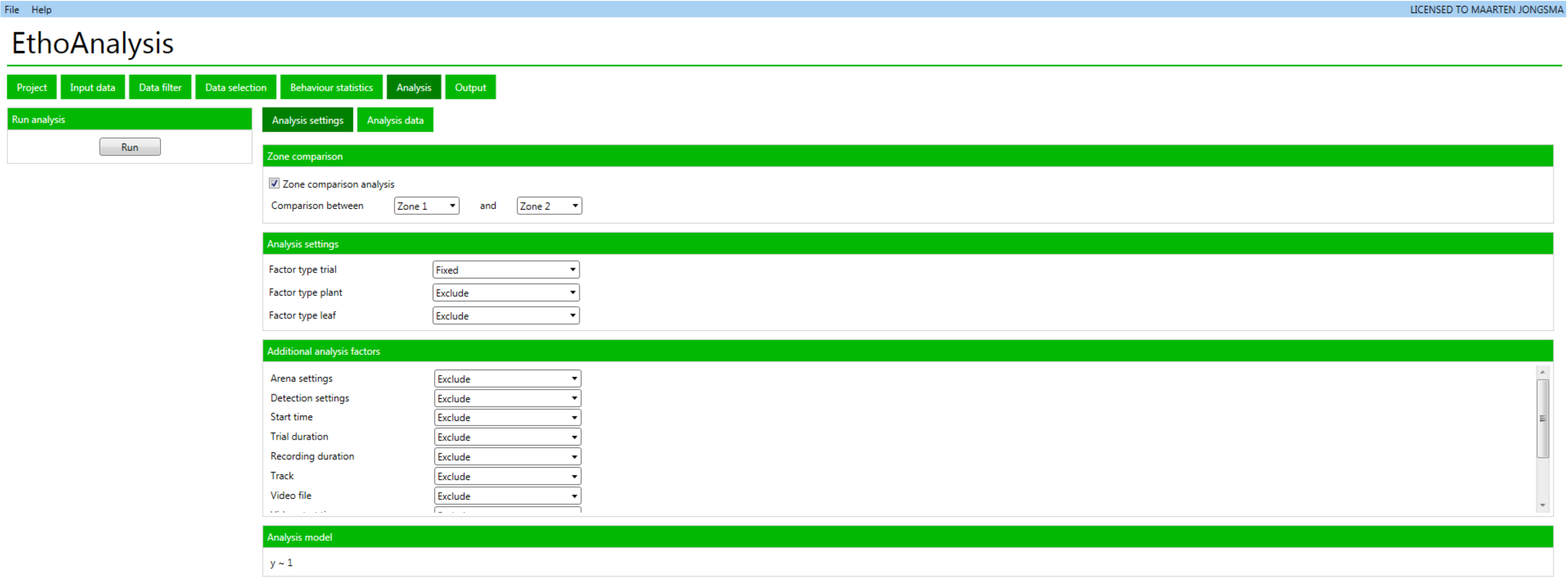

EthoAnalysis: Output tab

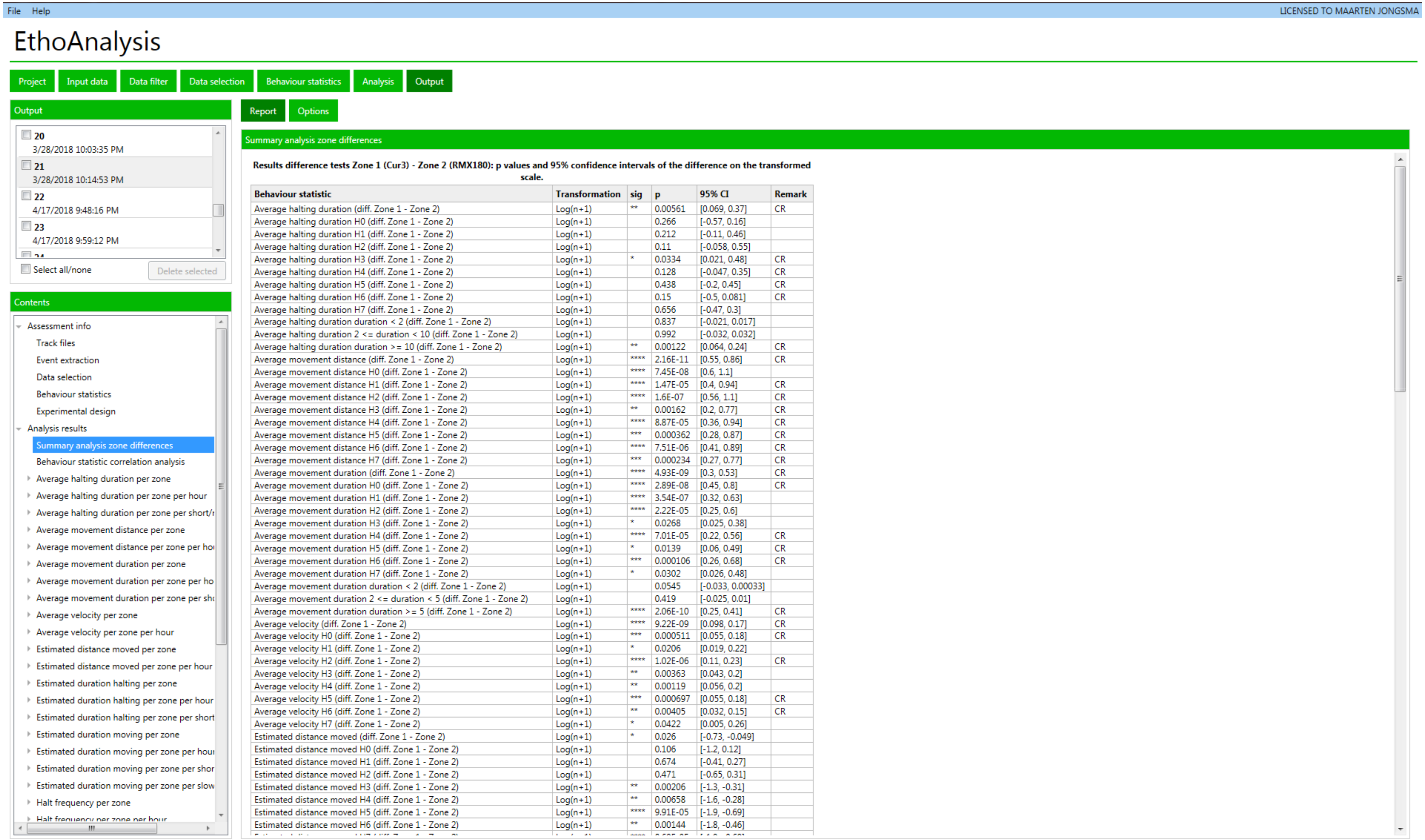

Results difference tests Zone 1 (Cur3) - Zone 2 (RMX180): p values and 95% confidence intervals of the difference on the transformed scale.

Behaviour statistic	Transformation	sig	p	95% CI	Remark
Average halting duration (diff. Zone 1 - Zone 2)	Log(n+1)	**	0.00561	[0.069, 0.37]	CR
Average halting duration H0 (diff. Zone 1 - Zone 2)	Log(n+1)		0.266	[-0.57, 0.16]	
Average halting duration H1 (diff. Zone 1 - Zone 2)	Log(n+1)		0.212	[-0.11, 0.46]	
Average halting duration H2 (diff. Zone 1 - Zone 2)	Log(n+1)		0.11	[-0.058, 0.55]	
Average halting duration H3 (diff. Zone 1 - Zone 2)	Log(n+1)	*	0.0334	[0.021, 0.48]	CR
Average halting duration H4 (diff. Zone 1 - Zone 2)	Log(n+1)		0.128	[-0.047, 0.35]	CR
Average halting duration H5 (diff. Zone 1 - Zone 2)	Log(n+1)		0.438	[-0.2, 0.45]	CR
Average halting duration H6 (diff. Zone 1 - Zone 2)	Log(n+1)		0.15	[-0.5, 0.081]	CR
Average halting duration H7 (diff. Zone 1 - Zone 2)	Log(n+1)		0.656	[-0.47, 0.3]	
Average halting duration duration < 2 (diff. Zone 1 - Zone 2)	Log(n+1)		0.837	[-0.021, 0.017]	
Average halting duration 2 <= duration < 10 (diff. Zone 1 - Zone 2)	Log(n+1)		0.992	[-0.032, 0.032]	
Average halting duration duration >= 10 (diff. Zone 1 - Zone 2)	Log(n+1)	**	0.00122	[0.064, 0.24]	CR
Average movement distance (diff. Zone 1 - Zone 2)	Log(n+1)	****	2.16E-11	[0.55, 0.86]	CR
Average movement distance H0 (diff. Zone 1 - Zone 2)	Log(n+1)	****	7.45E-08	[0.6, 1.1]	
Average movement distance H1 (diff. Zone 1 - Zone 2)	Log(n+1)	****	1.47E-05	[0.4, 0.94]	CR
Average movement distance H2 (diff. Zone 1 - Zone 2)	Log(n+1)	****	1.6E-07	[0.56, 1.1]	CR
Average movement distance H3 (diff. Zone 1 - Zone 2)	Log(n+1)	**	0.00162	[0.2, 0.77]	CR
Average movement distance H4 (diff. Zone 1 - Zone 2)	Log(n+1)	****	8.87E-05	[0.36, 0.94]	CR
Average movement distance H5 (diff. Zone 1 - Zone 2)	Log(n+1)	***	0.000362	[0.28, 0.87]	CR
Average movement distance H6 (diff. Zone 1 - Zone 2)	Log(n+1)	****	7.51E-06	[0.41, 0.89]	CR
Average movement distance H7 (diff. Zone 1 - Zone 2)	Log(n+1)	***	0.000234	[0.27, 0.77]	CR
Average movement duration (diff. Zone 1 - Zone 2)	Log(n+1)	****	4.93E-09	[0.3, 0.53]	CR
Average movement duration H0 (diff. Zone 1 - Zone 2)	Log(n+1)	****	2.89E-08	[0.45, 0.8]	CR
Average movement duration H1 (diff. Zone 1 - Zone 2)	Log(n+1)	****	3.54E-07	[0.32, 0.63]	
Average movement duration H2 (diff. Zone 1 - Zone 2)	Log(n+1)	****	2.22E-05	[0.25, 0.6]	
Average movement duration H3 (diff. Zone 1 - Zone 2)	Log(n+1)	*	0.0268	[0.025, 0.38]	
Average movement duration H4 (diff. Zone 1 - Zone 2)	Log(n+1)	****	7.01E-05	[0.22, 0.56]	CR
Average movement duration H5 (diff. Zone 1 - Zone 2)	Log(n+1)	*	0.0139	[0.06, 0.49]	CR
Average movement duration H6 (diff. Zone 1 - Zone 2)	Log(n+1)	***	0.000106	[0.26, 0.68]	CR
Average movement duration H7 (diff. Zone 1 - Zone 2)	Log(n+1)	*	0.0302	[0.026, 0.48]	
Average movement duration duration < 2 (diff. Zone 1 - Zone 2)	Log(n+1)		0.0545	[-0.033, 0.00033]	
Average movement duration 2 <= duration < 5 (diff. Zone 1 - Zone 2)	Log(n+1)		0.419	[-0.025, 0.01]	
Average movement duration duration >= 5 (diff. Zone 1 - Zone 2)	Log(n+1)	****	2.06E-10	[0.25, 0.41]	CR
Average velocity (diff. Zone 1 - Zone 2)	Log(n+1)	****	9.22E-09	[0.098, 0.17]	CR
Average velocity H0 (diff. Zone 1 - Zone 2)	Log(n+1)	***	0.000511	[0.055, 0.18]	CR
Average velocity H1 (diff. Zone 1 - Zone 2)	Log(n+1)	*	0.0206	[0.019, 0.22]	
Average velocity H2 (diff. Zone 1 - Zone 2)	Log(n+1)	****	1.02E-06	[0.11, 0.23]	CR
Average velocity H3 (diff. Zone 1 - Zone 2)	Log(n+1)	**	0.00363	[0.043, 0.2]	
Average velocity H4 (diff. Zone 1 - Zone 2)	Log(n+1)	**	0.00119	[0.056, 0.2]	
Average velocity H5 (diff. Zone 1 - Zone 2)	Log(n+1)	***	0.000697	[0.055, 0.18]	CR
Average velocity H6 (diff. Zone 1 - Zone 2)	Log(n+1)	**	0.00405	[0.032, 0.15]	CR
Average velocity H7 (diff. Zone 1 - Zone 2)	Log(n+1)	*	0.0422	[0.005, 0.26]	
Estimated distance moved (diff. Zone 1 - Zone 2)	Log(n+1)	*	0.026	[-0.73, -0.049]	
Estimated distance moved H0 (diff. Zone 1 - Zone 2)	Log(n+1)		0.106	[-1.2, 0.12]	
Estimated distance moved H1 (diff. Zone 1 - Zone 2)	Log(n+1)		0.674	[-0.41, 0.27]	
Estimated distance moved H2 (diff. Zone 1 - Zone 2)	Log(n+1)		0.471	[-0.65, 0.31]	
Estimated distance moved H3 (diff. Zone 1 - Zone 2)	Log(n+1)	**	0.00206	[-1.3, -0.31]	
Estimated distance moved H4 (diff. Zone 1 - Zone 2)	Log(n+1)	**	0.00658	[-1.6, -0.28]	
Estimated distance moved H5 (diff. Zone 1 - Zone 2)	Log(n+1)	****	9.91E-05	[-1.9, -0.69]	
Estimated distance moved H6 (diff. Zone 1 - Zone 2)	Log(n+1)	**	0.00144	[-1.8, -0.46]	
[illegible]	[illegible]	[illegible]	[illegible]	[illegible]	
