# Supplementary material for: An Integrated System for the Automated Recording and Analysis of Insect Behavior in T-maze Arrays
Source: Front Plant Sci. 2019 Jan 29;10:20. doi: 10.3389/fpls.2019.00020 (PMC6361829; doi:10.3389/fpls.2019.00020)
Supplement: Supplementary Data File 2 — Automatically generated statistical analysis by the EthoAnalysis software using the F1 settings. This represents the most extensive format. Optional are reports with only the main results. [file Data_Sheet_2.pdf]

Report

Assay info

**Project name**	F1
**Assessment type**	Multiple choice assessment
**Multiple zones**	Yes
**Include arena as zone**	No

**Number of trials**	3
**Number of genotypes**	3
**Number of experiments**	112

Track files

Total: 112 track files

Trial	Arena	Genotype Zone 1	Plant Zone 1	Leaf Zone 1	Genotype Zone 2	Plant Zone 2	Leaf Zone 2	Genotype Zone 3	Data file
Trial 1	0	Cur3	1	4	RMX180	10	4	Neutral	..\..\Frankliniella-Arabidopsis\Track-Frankliniella-Arabidopsis-Trial 1-1-Subject 1.txt
Trial 1	1	Cur3	4	4	RMX180	2	4	Neutral	..\..\Frankliniella-Arabidopsis\Track-Frankliniella-Arabidopsis-Trial 1-2-Subject 1.txt
Trial 1	2	Cur3	2	4	RMX180	9	4	Neutral	..\..\Frankliniella-Arabidopsis\Track-Frankliniella-Arabidopsis-Trial 1-3-Subject 1.txt
Trial 1	3	Cur3	9	4	RMX180	5	4	Neutral	..\..\Frankliniella-Arabidopsis\Track-Frankliniella-Arabidopsis-Trial 1-4-Subject 1.txt
Trial 1	4	Cur3	8	4	RMX180	1	4	Neutral	..\..\Frankliniella-Arabidopsis\Track-Frankliniella-Arabidopsis-Trial 1-5-Subject 1.txt
Trial 1	5	Cur3	3	4	RMX180	8	4	Neutral	..\..\Frankliniella-Arabidopsis\Track-Frankliniella-Arabidopsis-Trial 1-6-Subject 1.txt
Trial 1	6	Cur3	6	4	RMX180	7	4	Neutral	..\..\Frankliniella-Arabidopsis\Track-Frankliniella-Arabidopsis-Trial 1-7-Subject 1.txt
Trial 1	7	Cur3	7	4	RMX180	4	4	Neutral	..\..\Frankliniella-Arabidopsis\Track-Frankliniella-Arabidopsis-Trial 1-8-Subject 1.txt
Trial 1	8	Cur3	5	4	RMX180	3	4	Neutral	..\..\Frankliniella-Arabidopsis\Track-Frankliniella-Arabidopsis-Trial 1-9-Subject 1.txt
Trial 1	9	Cur3	10	4	RMX180	6	4	Neutral	..\..\Frankliniella-Arabidopsis\Track-Frankliniella-Arabidopsis-Trial 1-10-Subject 1.txt
Trial 1	10	Cur3	7	3	RMX180	8	3	Neutral	..\..\Frankliniella-Arabidopsis\Track-Frankliniella-Arabidopsis-Trial 1-11-Subject 1.txt
Trial 1	11	Cur3	5	3	RMX180	5	3	Neutral	..\..\Frankliniella-Arabidopsis\Track-Frankliniella-Arabidopsis-Trial 1-12-Subject 1.txt
Trial 1	12	Cur3	6	3	RMX180	6	3	Neutral	..\..\Frankliniella-Arabidopsis\Track-Frankliniella-Arabidopsis-Trial 1-13-Subject 1.txt
Trial 1	13	Cur3	9	3	RMX180	10	3	Neutral	..\..\Frankliniella-Arabidopsis\Track-Frankliniella-Arabidopsis-Trial 1-14-Subject 1.txt
Trial 1	14	Cur3	2	3	RMX180	2	3	Neutral	..\..\Frankliniella-Arabidopsis\Track-Frankliniella-Arabidopsis-Trial 1-15-Subject 1.txt
Trial 1	15	Cur3	1	3	RMX180	4	3	Neutral	..\..\Frankliniella-Arabidopsis\Track-Frankliniella-Arabidopsis-Trial 1-16-Subject 1.txt
Trial 1	16	Cur3	10	3	RMX180	9	3	Neutral	..\..\Frankliniella-Arabidopsis\Track-Frankliniella-Arabidopsis-Trial 1-17-Subject 1.txt
Trial 1	17	Cur3	3	3	RMX180	3	3	Neutral	..\..\Frankliniella-Arabidopsis\Track-Frankliniella-Arabidopsis-Trial 1-18-Subject 1.txt
Trial 1	18	Cur3	4	3	RMX180	7	3	Neutral	..\..\Frankliniella-Arabidopsis\Track-Frankliniella-Arabidopsis-Trial 1-19-Subject 1.txt
Trial 1	19	Cur3	8	3	RMX180	1	3	Neutral	..\..\Frankliniella-Arabidopsis\Track-Frankliniella-Arabidopsis-Trial 1-20-Subject 1.txt
Trial 1	20	Cur3	10	2	RMX180	10	2	Neutral	..\..\Frankliniella-Arabidopsis\Track-Frankliniella-Arabidopsis-Trial 1-21-Subject 1.txt
Trial 1	21	Cur3	9	2	RMX180	2	2	Neutral	..\..\Frankliniella-Arabidopsis\Track-Frankliniella-Arabidopsis-Trial 1-22-Subject 1.txt
Trial 1	22	Cur3	5	2	RMX180	8	2	Neutral	..\..\Frankliniella-Arabidopsis\Track-Frankliniella-Arabidopsis-Trial 1-23-Subject 1.txt
Trial 1	23	Cur3	1	2	RMX180	4	2	Neutral	..\..\Frankliniella-Arabidopsis\Track-Frankliniella-Arabidopsis-Trial 1-24-Subject 1.txt
Trial 1	24	Cur3	6	2	RMX180	1	2	Neutral	..\..\Frankliniella-Arabidopsis\Track-Frankliniella-Arabidopsis-Trial 1-25-Subject 1.txt
Trial 1	25	Cur3	3	2	RMX180	7	2	Neutral	..\..\Frankliniella-Arabidopsis\Track-Frankliniella-Arabidopsis-Trial 1-26-Subject 1.txt
Trial 1	26	Cur3	7	2	RMX180	5	2	Neutral	..\..\Frankliniella-Arabidopsis\Track-Frankliniella-Arabidopsis-Trial 1-27-Subject 1.txt
Trial 1	27	Cur3	4	2	RMX180	6	2	Neutral	..\..\Frankliniella-Arabidopsis\Track-Frankliniella-Arabidopsis-Trial 1-28-Subject 1.txt
Trial 1	28	Cur3	8	2	RMX180	9	2	Neutral	..\..\Frankliniella-Arabidopsis\Track-Frankliniella-Arabidopsis-Trial 1-29-Subject 1.txt
Trial 1	29	Cur3	2	2	RMX180	3	2	Neutral	..\..\Frankliniella-Arabidopsis\Track-Frankliniella-Arabidopsis-Trial 1-30-Subject 1.txt
Trial 1	30	Cur3	1	1	RMX180	2	1	Neutral	..\..\Frankliniella-Arabidopsis\Track-Frankliniella-Arabidopsis-Trial 1-31-Subject 1.txt
Trial 1	31	Cur3	4	1	RMX180	7	1	Neutral	..\..\Frankliniella-Arabidopsis\Track-Frankliniella-Arabidopsis-Trial 1-32-Subject 1.txt
Trial 2	0	Cur3	17	1	RMX180	14	1	Neutral	..\..\Frankliniella-Arabidopsis\Track-Frankliniella-Arabidopsis-Trial 2-1-Subject 1.txt
Trial 2	1	Cur3	20	1	RMX180	13	1	Neutral	..\..\Frankliniella-Arabidopsis\Track-Frankliniella-Arabidopsis-Trial 2-2-Subject 1.txt
Trial 2	2	Cur3	13	1	RMX180	16	1	Neutral	..\..\Frankliniella-Arabidopsis\Track-Frankliniella-Arabidopsis-Trial 2-3-Subject 1.txt
Trial 2	3	Cur3	16	1	RMX180	15	1	Neutral	..\..\Frankliniella-Arabidopsis\Track-Frankliniella-Arabidopsis-Trial 2-4-Subject 1.txt
Trial 2	4	Cur3	12	1	RMX180	11	1	Neutral	..\..\Frankliniella-Arabidopsis\Track-Frankliniella-Arabidopsis-Trial 2-5-Subject 1.txt
Trial 2	5	Cur3	14	1	RMX180	17	1	Neutral	..\..\Frankliniella-Arabidopsis\Track-Frankliniella-Arabidopsis-Trial 2-6-Subject 1.txt
Trial 2	6	Cur3	18	1	RMX180	18	1	Neutral	..\..\Frankliniella-Arabidopsis\Track-Frankliniella-Arabidopsis-Trial 2-7-Subject 1.txt
Trial 2	7	Cur3	11	1	RMX180	19	1	Neutral	..\..\Frankliniella-Arabidopsis\Track-Frankliniella-Arabidopsis-Trial 2-8-Subject 1.txt
Trial 2	8	Cur3	19	1	RMX180	20	1	Neutral	..\..\Frankliniella-Arabidopsis\Track-Frankliniella-Arabidopsis-Trial 2-9-Subject 1.txt
Trial 2	9	Cur3	15	2	RMX180	12	2	Neutral	..\..\Frankliniella-Arabidopsis\Track-Frankliniella-Arabidopsis-Trial 2-10-Subject 1.txt
Trial 2	10	Cur3	18	2	RMX180	19	2	Neutral	..\..\Frankliniella-Arabidopsis\Track-Frankliniella-Arabidopsis-Trial 2-11-Subject 1.txt
Trial 2	11	Cur3	12	2	RMX180	16	2	Neutral	..\..\Frankliniella-Arabidopsis\Track-Frankliniella-Arabidopsis-Trial 2-12-Subject 1.txt
Trial 2	12	Cur3	15	2	RMX180	18	2	Neutral	..\..\Frankliniella-Arabidopsis\Track-Frankliniella-Arabidopsis-Trial 2-13-Subject 1.txt
Trial 2	13	Cur3	19	2	RMX180	14	2	Neutral	..\..\Frankliniella-Arabidopsis\Track-Frankliniella-Arabidopsis-Trial 2-14-Subject 1.txt
Trial 2	14	Cur3	11	2	RMX180	20	2	Neutral	..\..\Frankliniella-Arabidopsis\Track-Frankliniella-Arabidopsis-Trial 2-15-Subject 1.txt
Trial 2	15	Cur3	16	2	RMX180	11	2	Neutral	..\..\Frankliniella-Arabidopsis\Track-Frankliniella-Arabidopsis-Trial 2-16-Subject 1.txt
Trial 2	16	Cur3	13	2	RMX180	17	2	Neutral	..\..\Frankliniella-Arabidopsis\Track-Frankliniella-Arabidopsis-Trial 2-17-Subject 1.txt
Trial 2	17	Cur3	20	2	RMX180	12	2	Neutral	..\..\Frankliniella-Arabidopsis\Track-Frankliniella-Arabidopsis-Trial 2-18-Subject 1.txt
Trial 2	18	Cur3	17	2	RMX180	13	2	Neutral	..\..\Frankliniella-Arabidopsis\Track-Frankliniella-Arabidopsis-Trial 2-19-Subject 1.txt
Trial 2	19	Cur3	14	2	RMX180	15	2	Neutral	..\..\Frankliniella-Arabidopsis\Track-Frankliniella-Arabidopsis-Trial 2-20-Subject 1.txt
Trial 2	20	Cur3	13	3	RMX180	14	3	Neutral	..\..\Frankliniella-Arabidopsis\Track-Frankliniella-Arabidopsis-Trial 2-21-Subject 1.txt
Trial 2	21	Cur3	19	3	RMX180	15	3	Neutral	..\..\Frankliniella-Arabidopsis\Track-Frankliniella-Arabidopsis-Trial 2-22-Subject 1.txt
Trial 2	22	Cur3	20	3	RMX180	17	3	Neutral	..\..\Frankliniella-Arabidopsis\Track-Frankliniella-Arabidopsis-Trial 2-23-Subject 1.txt
Trial 2	23	Cur3	18	3	RMX180	13	3	Neutral	..\..\Frankliniella-Arabidopsis\Track-Frankliniella-Arabidopsis-Trial 2-24-Subject 1.txt
Trial 2	24	Cur3	17	3	RMX180	12	3	Neutral	..\..\Frankliniella-Arabidopsis\Track-Frankliniella-Arabidopsis-Trial 2-25-Subject 1.txt
Trial 2	25	Cur3	14	3	RMX180	20	3	Neutral	..\..\Frankliniella-Arabidopsis\Track-Frankliniella-Arabidopsis-Trial 2-26-Subject 1.txt
Trial 2	26	Cur3	12	3	RMX180	18	3	Neutral	..\..\Frankliniella-Arabidopsis\Track-Frankliniella-Arabidopsis-Trial 2-27-Subject 1.txt
Trial 2	27	Cur3	16	3	RMX180	19	3	Neutral	..\..\Frankliniella-Arabidopsis\Track-Frankliniella-Arabidopsis-Trial 2-28-Subject 1.txt
Trial 2	28	Cur3	15	3	RMX180	11	3	Neutral	..\..\Frankliniella-Arabidopsis\Track-Frankliniella-Arabidopsis-Trial 2-29-Subject 1.txt

Trial	Arena	Genotype Zone 1	Plant Zone 1	Leaf Zone 1	Genotype Zone 2	Plant Zone 2	Leaf Zone 2	Genotype Zone 3	Data file
Trial 2	29	Cur3	11	3	RMX180	16	3	Neutral	..\..\Frankliniella-Arabidopsis\Track-Frankliniella-Arabidopsis-Trial 2-30-Subject 1.txt
Trial 2	30	Cur3	13	4	RMX180	14	4	Neutral	..\..\Frankliniella-Arabidopsis\Track-Frankliniella-Arabidopsis-Trial 2-31-Subject 1.txt
Trial 2	31	Cur3	14	4	RMX180	20	4	Neutral	..\..\Frankliniella-Arabidopsis\Track-Frankliniella-Arabidopsis-Trial 2-32-Subject 1.txt
Trial 2	32	Cur3	11	4	RMX180	17	4	Neutral	..\..\Frankliniella-Arabidopsis\Track-Frankliniella-Arabidopsis-Trial 2-33-Subject 1.txt
Trial 2	33	Cur3	19	4	RMX180	11	4	Neutral	..\..\Frankliniella-Arabidopsis\Track-Frankliniella-Arabidopsis-Trial 2-34-Subject 1.txt
Trial 2	34	Cur3	17	4	RMX180	13	4	Neutral	..\..\Frankliniella-Arabidopsis\Track-Frankliniella-Arabidopsis-Trial 2-35-Subject 1.txt
Trial 2	35	Cur3	16	4	RMX180	16	4	Neutral	..\..\Frankliniella-Arabidopsis\Track-Frankliniella-Arabidopsis-Trial 2-36-Subject 1.txt
Trial 2	36	Cur3	12	4	RMX180	19	4	Neutral	..\..\Frankliniella-Arabidopsis\Track-Frankliniella-Arabidopsis-Trial 2-37-Subject 1.txt
Trial 2	37	Cur3	20	4	RMX180	15	4	Neutral	..\..\Frankliniella-Arabidopsis\Track-Frankliniella-Arabidopsis-Trial 2-38-Subject 1.txt
Trial 2	38	Cur3	18	4	RMX180	18	4	Neutral	..\..\Frankliniella-Arabidopsis\Track-Frankliniella-Arabidopsis-Trial 2-39-Subject 1.txt
Trial 2	39	Cur3	15	4	RMX180	12	4	Neutral	..\..\Frankliniella-Arabidopsis\Track-Frankliniella-Arabidopsis-Trial 2-40-Subject 1.txt
Trial 3	0	Cur3	22	1	RMX180	24	1	Neutral	..\..\Frankliniella-Arabidopsis\Track-Frankliniella-Arabidopsis-Trial 3-1-Subject 1.txt
Trial 3	1	Cur3	26	1	RMX180	25	1	Neutral	..\Track-Frankliniella-Arabidopsis-Trial 3-2-Subject 1.txt
Trial 3	2	Cur3	27	1	RMX180	30	1	Neutral	..\..\Frankliniella-Arabidopsis\Track-Frankliniella-Arabidopsis-Trial 3-3-Subject 1.txt
Trial 3	3	Cur3	21	1	RMX180	28	1	Neutral	..\..\Frankliniella-Arabidopsis\Track-Frankliniella-Arabidopsis-Trial 3-4-Subject 1.txt
Trial 3	4	Cur3	23	1	RMX180	22	1	Neutral	..\..\Frankliniella-Arabidopsis\Track-Frankliniella-Arabidopsis-Trial 3-5-Subject 1.txt
Trial 3	5	Cur3	25	1	RMX180	27	1	Neutral	..\..\Frankliniella-Arabidopsis\Track-Frankliniella-Arabidopsis-Trial 3-6-Subject 1.txt
Trial 3	6	Cur3	24	1	RMX180	23	1	Neutral	..\..\Frankliniella-Arabidopsis\Track-Frankliniella-Arabidopsis-Trial 3-7-Subject 1.txt
Trial 3	7	Cur3	28	1	RMX180	26	1	Neutral	..\..\Frankliniella-Arabidopsis\Track-Frankliniella-Arabidopsis-Trial 3-8-Subject 1.txt
Trial 3	8	Cur3	29	1	RMX180	21	1	Neutral	..\..\Frankliniella-Arabidopsis\Track-Frankliniella-Arabidopsis-Trial 3-9-Subject 1.txt
Trial 3	9	Cur3	30	2	RMX180	29	2	Neutral	..\..\Frankliniella-Arabidopsis\Track-Frankliniella-Arabidopsis-Trial 3-10-Subject 1.txt
Trial 3	10	Cur3	27	2	RMX180	26	2	Neutral	..\..\Frankliniella-Arabidopsis\Track-Frankliniella-Arabidopsis-Trial 3-11-Subject 1.txt
Trial 3	11	Cur3	29	2	RMX180	25	2	Neutral	..\..\Frankliniella-Arabidopsis\Track-Frankliniella-Arabidopsis-Trial 3-12-Subject 1.txt
Trial 3	12	Cur3	26	2	RMX180	21	2	Neutral	..\..\Frankliniella-Arabidopsis\Track-Frankliniella-Arabidopsis-Trial 3-13-Subject 1.txt
Trial 3	13	Cur3	22	2	RMX180	24	2	Neutral	..\..\Frankliniella-Arabidopsis\Track-Frankliniella-Arabidopsis-Trial 3-14-Subject 1.txt
Trial 3	14	Cur3	25	2	RMX180	29	2	Neutral	..\..\Frankliniella-Arabidopsis\Track-Frankliniella-Arabidopsis-Trial 3-15-Subject 1.txt
Trial 3	15	Cur3	23	2	RMX180	23	2	Neutral	..\..\Frankliniella-Arabidopsis\Track-Frankliniella-Arabidopsis-Trial 3-16-Subject 1.txt
Trial 3	16	Cur3	30	2	RMX180	27	2	Neutral	..\..\Frankliniella-Arabidopsis\Track-Frankliniella-Arabidopsis-Trial 3-17-Subject 1.txt
Trial 3	17	Cur3	24	2	RMX180	22	2	Neutral	..\..\Frankliniella-Arabidopsis\Track-Frankliniella-Arabidopsis-Trial 3-18-Subject 1.txt
Trial 3	18	Cur3	28	2	RMX180	28	2	Neutral	..\Track-Frankliniella-Arabidopsis-Trial 3-19-Subject 1.txt
Trial 3	19	Cur3	21	2	RMX180	30	2	Neutral	..\Track-Frankliniella-Arabidopsis-Trial 3-20-Subject 1.txt
Trial 3	20	Cur3	29	3	RMX180	26	3	Neutral	..\Track-Frankliniella-Arabidopsis-Trial 3-21-Subject 1.txt
Trial 3	21	Cur3	27	3	RMX180	30	3	Neutral	..\Track-Frankliniella-Arabidopsis-Trial 3-22-Subject 1.txt
Trial 3	22	Cur3	21	3	RMX180	22	3	Neutral	..\Track-Frankliniella-Arabidopsis-Trial 3-23-Subject 1.txt
Trial 3	23	Cur3	22	3	RMX180	29	3	Neutral	..\Track-Frankliniella-Arabidopsis-Trial 3-24-Subject 1.txt
Trial 3	24	Cur3	25	3	RMX180	23	3	Neutral	..\Track-Frankliniella-Arabidopsis-Trial 3-25-Subject 1.txt
Trial 3	25	Cur3	23	3	RMX180	28	3	Neutral	..\Track-Frankliniella-Arabidopsis-Trial 3-26-Subject 1.txt
Trial 3	26	Cur3	28	3	RMX180	24	3	Neutral	..\Track-Frankliniella-Arabidopsis-Trial 3-27-Subject 1.txt
Trial 3	27	Cur3	24	3	RMX180	27	3	Neutral	..\..\Frankliniella-Arabidopsis\Track-Frankliniella-Arabidopsis-Trial 3-28-Subject 1.txt
Trial 3	28	Cur3	30	3	RMX180	21	3	Neutral	..\..\Frankliniella-Arabidopsis\Track-Frankliniella-Arabidopsis-Trial 3-29-Subject 1.txt
Trial 3	29	Cur3	26	3	RMX180	25	3	Neutral	..\..\Frankliniella-Arabidopsis\Track-Frankliniella-Arabidopsis-Trial 3-30-Subject 1.txt
Trial 3	30	Cur3	22	4	RMX180	24	4	Neutral	..\..\Frankliniella-Arabidopsis\Track-Frankliniella-Arabidopsis-Trial 3-31-Subject 1.txt
Trial 3	31	Cur3	25	4	RMX180	30	4	Neutral	..\..\Frankliniella-Arabidopsis\Track-Frankliniella-Arabidopsis-Trial 3-32-Subject 1.txt
Trial 3	32	Cur3	27	4	RMX180	23	4	Neutral	..\..\Frankliniella-Arabidopsis\Track-Frankliniella-Arabidopsis-Trial 3-33-Subject 1.txt
Trial 3	33	Cur3	30	4	RMX180	29	4	Neutral	..\..\Frankliniella-Arabidopsis\Track-Frankliniella-Arabidopsis-Trial 3-34-Subject 1.txt
Trial 3	34	Cur3	26	4	RMX180	25	4	Neutral	..\..\Frankliniella-Arabidopsis\Track-Frankliniella-Arabidopsis-Trial 3-35-Subject 1.txt
Trial 3	35	Cur3	21	4	RMX180	28	4	Neutral	..\..\Frankliniella-Arabidopsis\Track-Frankliniella-Arabidopsis-Trial 3-36-Subject 1.txt
Trial 3	36	Cur3	24	4	RMX180	26	4	Neutral	..\..\Frankliniella-Arabidopsis\Track-Frankliniella-Arabidopsis-Trial 3-37-Subject 1.txt
Trial 3	37	Cur3	29	4	RMX180	27	4	Neutral	..\..\Frankliniella-Arabidopsis\Track-Frankliniella-Arabidopsis-Trial 3-38-Subject 1.txt
Trial 3	38	Cur3	28	4	RMX180	22	4	Neutral	..\..\Frankliniella-Arabidopsis\Track-Frankliniella-Arabidopsis-Trial 3-39-Subject 1.txt
Trial 3	39	Cur3	23	4	RMX180	21	4	Neutral	..\..\Frankliniella-Arabidopsis\Track-Frankliniella-Arabidopsis-Trial 3-40-Subject 1.txt

Event extraction

Event extraction

Setting	Value
**Look-ahead window size**	4
**Is-moving velocity threshold**	0.025
**Halting from non-detect events recovery**	True

Event filters

Filter	Settings
**Filter extreme velocity events**	Velocity threshold = 8

Data selection

Record filters

Filter	Settings
**Filter by inactivity**	Halt duration threshold = 3600
**Filter by detection percentage**	Minimal detection percentage = 50
**Filter by event count**	Number of events = 1000

Selected trials

Trial	Total experiments	Selected experiments
**Trial 3**	40	39

Selected analysis levels

Genotype Zone 1	Genotype Zone 2	Genotype Zone 3	Total experiments	Selected experiments
Cur3	RMX180	Neutral	112	39

Behaviour statistics

Behaviour statistic	Setting	Value
**Average halting duration per zone**		
	Selected zones	Zone 1, Zone 2

Behaviour statistic	Setting	Value
	Data transformation	Natural logarithm
	Include in analysis	yes
**Average halting duration per zone per hour**		
	Selected hours	0, 1, 2, 3, 4, 5, 6, 7
	Selected zones	Zone 1, Zone 2
	Data transformation	Natural logarithm
	Include in analysis	yes
**Average halting duration per zone per short/medium/long**		
	Selected zones	Zone 1, Zone 2
	Event duration categories	duration < 2, 2 <= duration < 10, duration >= 10
	Data transformation	Natural logarithm
	Include in analysis	yes
**Average movement distance per zone**		
	Selected zones	Zone 1, Zone 2
	Data transformation	Natural logarithm
	Include in analysis	yes
**Average movement distance per zone per hour**		
	Selected hours	0, 1, 2, 3, 4, 5, 6, 7
	Selected zones	Zone 1, Zone 2
	Data transformation	Natural logarithm
	Include in analysis	yes
**Average movement duration per zone**		
	Selected zones	Zone 1, Zone 2
	Data transformation	Natural logarithm
	Include in analysis	yes
**Average movement duration per zone per hour**		
	Selected hours	0, 1, 2, 3, 4, 5, 6, 7
	Selected zones	Zone 1, Zone 2
	Data transformation	Natural logarithm
	Include in analysis	yes
**Average movement duration per zone per short/medium/long**		
	Selected zones	Zone 1, Zone 2
	Event duration categories	duration < 2, 2 <= duration < 5, duration >= 5
	Data transformation	Natural logarithm
	Include in analysis	yes
**Average velocity per zone**		
	Selected zones	Zone 1, Zone 2
	Data transformation	Natural logarithm
	Include in analysis	yes
**Average velocity per zone per hour**		
	Selected hours	0, 1, 2, 3, 4, 5, 6, 7
	Selected zones	Zone 1, Zone 2
	Data transformation	Natural logarithm
	Include in analysis	yes
**Estimated distance moved per zone**		
	Selected zones	Zone 1, Zone 2
	Data transformation	Natural logarithm
	Include in analysis	yes
**Estimated distance moved per zone per hour**		
	Selected hours	0, 1, 2, 3, 4, 5, 6, 7
	Selected zones	Zone 1, Zone 2
	Data transformation	Natural logarithm
	Include in analysis	yes
**Estimated duration halting per zone**		
	Selected zones	Zone 1, Zone 2
	Data transformation	Natural logarithm
	Include in analysis	yes
**Estimated duration halting per zone per hour**		
	Selected hours	0, 1, 2, 3, 4, 5, 6, 7
	Selected zones	Zone 1, Zone 2
	Data transformation	Natural logarithm
	Include in analysis	yes
**Estimated duration halting per zone per short/medium/long**		
	Selected zones	Zone 1, Zone 2
	Event duration categories	duration < 2, 2 <= duration < 10, duration >= 10
	Data transformation	Natural logarithm
	Include in analysis	yes
**Estimated duration moving per zone**		
	Selected zones	Zone 1, Zone 2
	Data transformation	Natural logarithm
	Include in analysis	yes
**Estimated duration moving per zone per hour**		
	Selected hours	0, 1, 2, 3, 4, 5, 6, 7
	Selected zones	Zone 1, Zone 2
	Data transformation	Natural logarithm
	Include in analysis	yes
**Estimated duration moving per zone per short/medium/long**		
	Selected zones	Zone 1, Zone 2
	Event duration categories	duration < 2, 2 <= duration < 5, duration >= 5
	Data transformation	Natural logarithm
	Include in analysis	yes
**Estimated duration moving per zone per slow/medium/fast**		
	Selected zones	Zone 1, Zone 2
	Velocity categories	velocity < 0.025, 0.025 <= velocity < 0.075, velocity >= 0.075
	Data transformation	Natural logarithm
	Include in analysis	yes
**Halt frequency per zone**		
	Selected zones	Zone 1, Zone 2
	Data transformation	Natural logarithm
	Include in analysis	yes
**Halt frequency per zone per hour**		
	Selected hours	0, 1, 2, 3, 4, 5, 6, 7
	Selected zones	Zone 1, Zone 2
	Data transformation	Natural logarithm
	Include in analysis	yes
**Halt frequency per zone per short/medium/long**		
	Selected zones	Zone 1, Zone 2
	Event duration categories	duration < 2, 2 <= duration < 10, duration >= 10
	Data transformation	Natural logarithm
	Include in analysis	yes
**Movement frequency per zone**		
	Selected zones	Zone 1, Zone 2
	Data transformation	Natural logarithm
	Include in analysis	yes
**Movement frequency per zone per hour**		
	Selected hours	0, 1, 2, 3, 4, 5, 6, 7
	Selected zones	Zone 1, Zone 2
	Data transformation	Natural logarithm
	Include in analysis	yes
**Movement frequency per zone per short/medium/long**		
	Selected zones	Zone 1, Zone 2
	Event duration categories	duration < 2, 2 <= duration < 5, duration >= 5
	Data transformation	Natural logarithm
	Include in analysis	yes
**Movement frequency per zone per slow/medium/fast**		
	Selected zones	Zone 1, Zone 2
	Velocity categories	velocity < 0.025, 0.025 <= velocity < 0.075, velocity >= 0.075
	Data transformation	Natural logarithm
	Include in analysis	yes
**Ratio detection to total trial duration per zone**		
	Selected zones	Zone 1, Zone 2
	Data transformation	Natural logarithm
	Include in analysis	yes
**Ratio detection to total trial duration per zone per hour**		
	Selected hours	0, 1, 2, 3, 4, 5, 6, 7
	Selected zones	Zone 1, Zone 2
	Data transformation	Natural logarithm
	Include in analysis	yes
**Ratio halting to detection duration per zone**		
	Selected zones	Zone 1, Zone 2
	Data transformation	Natural logarithm
	Include in analysis	yes
**Ratio halting to detection duration per zone per hour**		
	Selected hours	0, 1, 2, 3, 4, 5, 6, 7

Behaviour statistic	Setting	Value
	Selected zones	Zone 1, Zone 2
	Data transformation	Natural logarithm
	Include in analysis	yes
**Ratio halting to total trial duration per zone**		
	Selected zones	Zone 1, Zone 2
	Data transformation	Natural logarithm
	Include in analysis	yes
**Ratio halting to total trial duration per zone per hour**		
	Selected hours	0, 1, 2, 3, 4, 5, 6, 7
	Selected zones	Zone 1, Zone 2
	Data transformation	Natural logarithm
	Include in analysis	yes
**Ratio movement to detection duration per zone**		
	Selected zones	Zone 1, Zone 2
	Data transformation	Natural logarithm
	Include in analysis	yes
**Ratio movement to detection duration per zone per hour**		
	Selected hours	0, 1, 2, 3, 4, 5, 6, 7
	Selected zones	Zone 1, Zone 2
	Data transformation	Natural logarithm
	Include in analysis	yes
**Ratio movement to halting duration per zone**		
	Selected zones	Zone 1, Zone 2
	Data transformation	Natural logarithm
	Include in analysis	yes
**Ratio movement to halting duration per zone per hour**		
	Selected hours	0, 1, 2, 3, 4, 5, 6, 7
	Selected zones	Zone 1, Zone 2
	Data transformation	Natural logarithm
	Include in analysis	yes

Experimental design

Replicates Trial 3

Genotype Zone 1	Plant Zone 1	Leaf Zone 1	Genotype Zone 2	Plant Zone 2	Leaf Zone 2	Genotype Zone 3	Replicates
Cur3	21	3	RMX180	22	3	Neutral	1
Cur3	21	1	RMX180	28	1	Neutral	1
Cur3	21	4	RMX180	28	4	Neutral	1
Cur3	21	2	RMX180	30	2	Neutral	1
Cur3	22	1	RMX180	24	1	Neutral	1
Cur3	22	2	RMX180	24	2	Neutral	1
Cur3	22	4	RMX180	24	4	Neutral	1
Cur3	22	3	RMX180	29	3	Neutral	1
Cur3	23	1	RMX180	22	1	Neutral	1
Cur3	23	2	RMX180	23	2	Neutral	1
Cur3	24	2	RMX180	22	2	Neutral	1
Cur3	24	1	RMX180	23	1	Neutral	1
Cur3	24	4	RMX180	26	4	Neutral	1
Cur3	24	3	RMX180	27	3	Neutral	1
Cur3	25	3	RMX180	23	3	Neutral	1
Cur3	25	1	RMX180	27	1	Neutral	1
Cur3	25	2	RMX180	29	2	Neutral	1
Cur3	25	4	RMX180	30	4	Neutral	1
Cur3	26	2	RMX180	21	2	Neutral	1
Cur3	26	1	RMX180	25	1	Neutral	1
Cur3	26	3	RMX180	25	3	Neutral	1
Cur3	26	4	RMX180	25	4	Neutral	1
Cur3	27	4	RMX180	23	4	Neutral	1
Cur3	27	2	RMX180	26	2	Neutral	1
Cur3	27	1	RMX180	30	1	Neutral	1
Cur3	27	3	RMX180	30	3	Neutral	1
Cur3	28	4	RMX180	22	4	Neutral	1
Cur3	28	3	RMX180	24	3	Neutral	1
Cur3	28	1	RMX180	26	1	Neutral	1
Cur3	28	2	RMX180	28	2	Neutral	1
Cur3	29	1	RMX180	21	1	Neutral	1
Cur3	29	2	RMX180	25	2	Neutral	1
Cur3	29	3	RMX180	26	3	Neutral	1
Cur3	29	4	RMX180	27	4	Neutral	1
Cur3	30	3	RMX180	21	3	Neutral	1
Cur3	30	2	RMX180	27	2	Neutral	1
Cur3	30	2	RMX180	29	2	Neutral	1
Cur3	30	4	RMX180	29	4	Neutral	1
Cur3	23	3	RMX180	28	3	Neutral	1

Replicates Trial 1

Genotype Zone 1	Genotype Zone 2	Genotype Zone 3	Replicates

Replicates Trial 2

Genotype Zone 1	Genotype Zone 2	Genotype Zone 3	Replicates

Analysis results

**Date/time analysis**	01-17-2019 9:57
**Duration analysis run**	00:03:36
**EthoAnalysis version**	1.3.0.6

Summary analysis zone differences

Summary of zone difference tests: p values and 95% confidence intervals of the ratio between Zone 1 (Cur3) and Zone 2 (RMX180).

Behaviour statistic	Transformation	sig	p	95% CI	Remark
Average halting duration (diff. Zone 1 - Zone 2)	Logit	**	0.00501	[1.09, 1.55]	
Average halting duration H0 (diff. Zone 1 - Zone 2)	Logit		0.375	[0.299, 1.62]	
Average halting duration H1 (diff. Zone 1 - Zone 2)	Logit		0.152	[0.895, 1.87]	CR
Average halting duration H2 (diff. Zone 1 - Zone 2)	Logit		0.126	[0.893, 2.18]	CR
Average halting duration H3 (diff. Zone 1 - Zone 2)	Logit	*	0.0145	[1.09, 1.85]	
Average halting duration H4 (diff. Zone 1 - Zone 2)	Logit		0.23	[0.883, 1.56]	
Average halting duration H5 (diff. Zone 1 - Zone 2)	Logit		0.284	[0.788, 2.06]	
Average halting duration H6 (diff. Zone 1 - Zone 2)	Logit		0.269	[0.531, 1.23]	
Average halting duration H7 (diff. Zone 1 - Zone 2)	Logit		0.955	[0.525, 1.97]	
Average halting duration duration < 2 (diff. Zone 1 - Zone 2)	Logit		0.894	[0.967, 1.03]	CR
Average halting duration 2 <= duration < 10 (diff. Zone 1 - Zone 2)	Logit		0.9	[0.957, 1.05]	CR
Average halting duration duration >= 10 (diff. Zone 1 - Zone 2)	Logit	***	0.000809	[1.08, 1.3]	
Average movement distance (diff. Zone 1 - Zone 2)	Logit	****	3.54E-05	[2.04, 3.83]	
Average movement distance H0 (diff. Zone 1 - Zone 2)	Logit	**	0.00403	[1.54, 5.96]	CR
Average movement distance H1 (diff. Zone 1 - Zone 2)	Logit	*	0.0122	[1.24, 3.87]	CR
Average movement distance H2 (diff. Zone 1 - Zone 2)	Logit	****	2.8E-08	[2.24, 4.21]	CR
Average movement distance H3 (diff. Zone 1 - Zone 2)	Logit	*	0.0494	[1, 3.93]	
Average movement distance H4 (diff. Zone 1 - Zone 2)	Logit	**	0.006	[1.47, 5.61]	
Average movement distance H5 (diff. Zone 1 - Zone 2)	Logit	*	0.0165	[1.22, 4.36]	
Average movement distance H6 (diff. Zone 1 - Zone 2)	Logit	***	0.000116	[1.87, 3.81]	
Average movement distance H7 (diff. Zone 1 - Zone 2)	Logit	**	0.00788	[1.31, 3.28]	
Average movement duration (diff. Zone 1 - Zone 2)	Logit	****	2.58E-11	[1.52, 1.92]	
Average movement duration H0 (diff. Zone 1 - Zone 2)	Logit	***	0.000892	[1.52, 2.73]	
Average movement duration H1 (diff. Zone 1 - Zone 2)	Logit	***	0.000126	[1.42, 2.2]	
Average movement duration H2 (diff. Zone 1 - Zone 2)	Logit	*	0.0177	[1.11, 2.3]	
Average movement duration H3 (diff. Zone 1 - Zone 2)	Logit		0.082	[0.959, 1.75]	CR
Average movement duration H4 (diff. Zone 1 - Zone 2)	Logit	**	0.00331	[1.26, 2.17]	
Average movement duration H5 (diff. Zone 1 - Zone 2)	Logit	*	0.0488	[1, 2.18]	
Average movement duration H6 (diff. Zone 1 - Zone 2)	Logit	****	3.56E-06	[1.49, 2.29]	
Average movement duration H7 (diff. Zone 1 - Zone 2)	Logit	*	0.0149	[1.1, 1.94]	
Average movement duration duration < 2 (diff. Zone 1 - Zone 2)	Logit		0.07	[0.941, 1]	CR
Average movement duration 2 <= duration < 5 (diff. Zone 1 - Zone 2)	Logit		0.497	[0.971, 1.01]	CR
Average movement duration duration >= 5 (diff. Zone 1 - Zone 2)	Logit	****	4.86E-11	[1.33, 1.56]	CR
Average velocity (diff. Zone 1 - Zone 2)	Logit	***	0.000461	[1.37, 2.17]	
Average velocity H0 (diff. Zone 1 - Zone 2)	Logit	*	0.0213	[1.09, 2.35]	
Average velocity H1 (diff. Zone 1 - Zone 2)	Logit		0.102	[0.909, 2.45]	
Average velocity H2 (diff. Zone 1 - Zone 2)	Logit	****	2.46E-06	[1.46, 2.23]	
Average velocity H3 (diff. Zone 1 - Zone 2)	Logit	*	0.0362	[1.04, 2.22]	
Average velocity H4 (diff. Zone 1 - Zone 2)	Logit	*	0.0193	[1.12, 2.77]	
Average velocity H5 (diff. Zone 1 - Zone 2)	Logit	*	0.0151	[1.13, 2.35]	
Average velocity H6 (diff. Zone 1 - Zone 2)	Logit	*	0.0243	[1.05, 1.82]	
Average velocity H7 (diff. Zone 1 - Zone 2)	Logit		0.12	[0.845, 3.29]	
Estimated distance moved (diff. Zone 1 - Zone 2)	Logit		0.118	[0.769, 1.04]	
Estimated distance moved H0 (diff. Zone 1 - Zone 2)	Logit		0.771	[0.681, 1.34]	

Behaviour statistic	Transformation	sig	p	95% CI	Remark
Estimated distance moved H1 (diff. Zone 1 - Zone 2)	Logit		0.309	[0.915, 1.31]	
Estimated distance moved H2 (diff. Zone 1 - Zone 2)	Logit		0.422	[0.679, 1.2]	
Estimated distance moved H3 (diff. Zone 1 - Zone 2)	Logit		0.226	[0.744, 1.07]	
Estimated distance moved H4 (diff. Zone 1 - Zone 2)	Logit		0.076	[0.565, 1.04]	
Estimated distance moved H5 (diff. Zone 1 - Zone 2)	Logit		0.0637	[0.524, 1.02]	
Estimated distance moved H6 (diff. Zone 1 - Zone 2)	Logit		0.615	[0.734, 1.2]	
Estimated distance moved H7 (diff. Zone 1 - Zone 2)	Logit		0.121	[0.492, 1.11]	
Estimated duration halting (diff. Zone 1 - Zone 2)	Logit	**	0.00476	[0.177, 0.564]	
Estimated duration halting H0 (diff. Zone 1 - Zone 2)	Logit	****	8.57E-06	[0.133, 0.416]	CR
Estimated duration halting H1 (diff. Zone 1 - Zone 2)	Logit	*	0.0355	[0.295, 0.948]	CR
Estimated duration halting H2 (diff. Zone 1 - Zone 2)	Logit		0.0822	[0.12, 1.18]	
Estimated duration halting H3 (diff. Zone 1 - Zone 2)	Logit	*	0.0362	[0.174, 0.929]	CR
Estimated duration halting H4 (diff. Zone 1 - Zone 2)	Logit	*	0.0377	[0.17, 0.926]	CR
Estimated duration halting H5 (diff. Zone 1 - Zone 2)	Logit	**	0.00532	[0.143, 0.602]	CR
Estimated duration halting H6 (diff. Zone 1 - Zone 2)	Logit	**	0.00431	[0.0696, 0.504]	CR
Estimated duration halting H7 (diff. Zone 1 - Zone 2)	Logit	**	0.00316	[0.081, 0.487]	CR
Estimated duration halting duration < 2 (diff. Zone 1 - Zone 2)	Logit	****	1.25E-05	[0.151, 0.34]	
Estimated duration halting 2 <= duration < 10 (diff. Zone 1 - Zone 2)	Logit	****	2.7E-05	[0.152, 0.35]	
Estimated duration halting duration >= 10 (diff. Zone 1 - Zone 2)	Logit	*	0.0129	[0.181, 0.727]	
Estimated duration moving (diff. Zone 1 - Zone 2)	Logit	***	0.000403	[0.375, 0.665]	CR
Estimated duration moving H0 (diff. Zone 1 - Zone 2)	Logit	*	0.0451	[0.385, 0.987]	
Estimated duration moving H1 (diff. Zone 1 - Zone 2)	Logit	*	0.0157	[0.519, 0.93]	
Estimated duration moving H2 (diff. Zone 1 - Zone 2)	Logit	**	0.00426	[0.336, 0.736]	CR
Estimated duration moving H3 (diff. Zone 1 - Zone 2)	Logit	**	0.00289	[0.362, 0.756]	
Estimated duration moving H4 (diff. Zone 1 - Zone 2)	Logit	*	0.0127	[0.19, 0.768]	CR
Estimated duration moving H5 (diff. Zone 1 - Zone 2)	Logit	**	0.00138	[0.213, 0.602]	CR
Estimated duration moving H6 (diff. Zone 1 - Zone 2)	Logit	**	0.00339	[0.302, 0.737]	
Estimated duration moving H7 (diff. Zone 1 - Zone 2)	Logit	**	0.00891	[0.242, 0.756]	CR
Estimated duration moving duration < 2 (diff. Zone 1 - Zone 2)	Logit	***	0.000274	[0.151, 0.391]	
Estimated duration moving 2 <= duration < 5 (diff. Zone 1 - Zone 2)	Logit	****	2.58E-05	[0.134, 0.33]	CR
Estimated duration moving duration >= 5 (diff. Zone 1 - Zone 2)	Logit	**	0.00275	[0.454, 0.797]	
Estimated duration moving velocity < 0.025 (diff. Zone 1 - Zone 2)	Logit	***	0.000244	[0.123, 0.349]	
Estimated duration moving 0.025 <= velocity < 0.075 (diff. Zone 1 - Zone 2)	Logit	****	5.86E-05	[0.104, 0.333]	
Estimated duration moving velocity >= 0.075 (diff. Zone 1 - Zone 2)	Logit	*	0.0102	[0.62, 0.915]	
Halt frequency (diff. Zone 1 - Zone 2)	Logit	****	7.73E-05	[0.159, 0.38]	
Halt frequency H0 (diff. Zone 1 - Zone 2)	Logit	***	0.000622	[0.142, 0.463]	CR
Halt frequency H1 (diff. Zone 1 - Zone 2)	Logit	***	0.000185	[0.156, 0.467]	
Halt frequency H2 (diff. Zone 1 - Zone 2)	Logit	**	0.00314	[0.128, 0.56]	CR
Halt frequency H3 (diff. Zone 1 - Zone 2)	Logit	**	0.00358	[0.164, 0.617]	CR
Halt frequency H4 (diff. Zone 1 - Zone 2)	Logit	**	0.00279	[0.113, 0.497]	CR
Halt frequency H5 (diff. Zone 1 - Zone 2)	Logit	***	0.000343	[0.1, 0.379]	CR
Halt frequency H6 (diff. Zone 1 - Zone 2)	Logit	***	0.000276	[0.108, 0.398]	CR
Halt frequency H7 (diff. Zone 1 - Zone 2)	Logit	**	0.00422	[0.0984, 0.501]	CR
Halt frequency duration < 2 (diff. Zone 1 - Zone 2)	Logit	****	8.17E-06	[0.152, 0.345]	CR
Halt frequency 2 <= duration < 10 (diff. Zone 1 - Zone 2)	Logit	****	2.39E-05	[0.148, 0.348]	
Halt frequency duration >= 10 (diff. Zone 1 - Zone 2)	Logit	**	0.00413	[0.167, 0.58]	
Movement frequency (diff. Zone 1 - Zone 2)	Logit	****	6E-05	[0.198, 0.426]	
Movement frequency H0 (diff. Zone 1 - Zone 2)	Logit	**	0.00131	[0.166, 0.53]	
Movement frequency H1 (diff. Zone 1 - Zone 2)	Logit	****	7.34E-06	[0.276, 0.566]	
Movement frequency H2 (diff. Zone 1 - Zone 2)	Logit	**	0.00204	[0.177, 0.572]	CR
Movement frequency H3 (diff. Zone 1 - Zone 2)	Logit	**	0.00195	[0.221, 0.626]	
Movement frequency H4 (diff. Zone 1 - Zone 2)	Logit	**	0.00322	[0.134, 0.548]	CR
Movement frequency H5 (diff. Zone 1 - Zone 2)	Logit	***	0.000302	[0.128, 0.418]	CR
Movement frequency H6 (diff. Zone 1 - Zone 2)	Logit	****	2.16E-06	[0.151, 0.413]	CR
Movement frequency H7 (diff. Zone 1 - Zone 2)	Logit	**	0.00281	[0.119, 0.533]	CR
Movement frequency duration < 2 (diff. Zone 1 - Zone 2)	Logit	***	0.000342	[0.155, 0.403]	
Movement frequency 2 <= duration < 5 (diff. Zone 1 - Zone 2)	Logit	****	2.7E-05	[0.133, 0.33]	CR
Movement frequency duration >= 5 (diff. Zone 1 - Zone 2)	Logit	***	0.000128	[0.307, 0.583]	
Movement frequency velocity < 0.025 (diff. Zone 1 - Zone 2)	Logit	****	5.69E-05	[0.152, 0.384]	CR
Movement frequency 0.025 <= velocity < 0.075 (diff. Zone 1 - Zone 2)	Logit	****	7.51E-05	[0.122, 0.333]	
Movement frequency velocity >= 0.075 (diff. Zone 1 - Zone 2)	Logit	****	3.56E-05	[0.408, 0.626]	
Ratio detection to total trial duration (diff. Zone 1 - Zone 2)	Logit	**	0.00172	[0.27, 0.582]	
Ratio detection to total trial duration H0 (diff. Zone 1 - Zone 2)	Logit	***	0.000125	[0.278, 0.633]	CR
Ratio detection to total trial duration H1 (diff. Zone 1 - Zone 2)	Logit	*	0.0181	[0.422, 0.918]	
Ratio detection to total trial duration H2 (diff. Zone 1 - Zone 2)	Logit	*	0.0191	[0.267, 0.859]	CR
Ratio detection to total trial duration H3 (diff. Zone 1 - Zone 2)	Logit	*	0.013	[0.269, 0.817]	CR
Ratio detection to total trial duration H4 (diff. Zone 1 - Zone 2)	Logit	*	0.02	[0.199, 0.819]	CR
Ratio detection to total trial duration H5 (diff. Zone 1 - Zone 2)	Logit	****	3.09E-05	[0.198, 0.523]	CR
Ratio detection to total trial duration H6 (diff. Zone 1 - Zone 2)	Logit	****	3.4E-05	[0.19, 0.516]	CR
Ratio detection to total trial duration H7 (diff. Zone 1 - Zone 2)	Logit	**	0.00444	[0.156, 0.587]	CR
Ratio halting to detection duration (diff. Zone 1 - Zone 2)	Logit	*	0.0291	[0.711, 0.976]	
Ratio halting to detection duration H0 (diff. Zone 1 - Zone 2)	Logit	*	0.0322	[0.482, 0.961]	
Ratio halting to detection duration H1 (diff. Zone 1 - Zone 2)	Logit	*	0.0484	[0.573, 0.998]	
Ratio halting to detection duration H2 (diff. Zone 1 - Zone 2)	Logit		0.244	[0.52, 1.22]	
Ratio halting to detection duration H3 (diff. Zone 1 - Zone 2)	Logit		0.432	[0.586, 1.3]	
Ratio halting to detection duration H4 (diff. Zone 1 - Zone 2)	Logit		0.197	[0.601, 1.12]	
Ratio halting to detection duration H5 (diff. Zone 1 - Zone 2)	Logit		0.198	[0.558, 1.15]	
Ratio halting to detection duration H6 (diff. Zone 1 - Zone 2)	Logit	**	0.00164	[0.403, 0.789]	
Ratio halting to detection duration H7 (diff. Zone 1 - Zone 2)	Logit	*	0.0326	[0.349, 0.944]	
Ratio halting to total trial duration (diff. Zone 1 - Zone 2)	Logit	***	0.000283	[0.234, 0.532]	
Ratio halting to total trial duration H0 (diff. Zone 1 - Zone 2)	Logit	****	5.49E-05	[0.188, 0.527]	CR
Ratio halting to total trial duration H1 (diff. Zone 1 - Zone 2)	Logit	**	0.00827	[0.325, 0.837]	CR
Ratio halting to total trial duration H2 (diff. Zone 1 - Zone 2)	Logit	*	0.0124	[0.213, 0.778]	CR
Ratio halting to total trial duration H3 (diff. Zone 1 - Zone 2)	Logit	*	0.0315	[0.273, 0.926]	CR
Ratio halting to total trial duration H4 (diff. Zone 1 - Zone 2)	Logit	**	0.00968	[0.199, 0.742]	CR
Ratio halting to total trial duration H5 (diff. Zone 1 - Zone 2)	Logit	**	0.006	[0.179, 0.68]	CR
Ratio halting to total trial duration H6 (diff. Zone 1 - Zone 2)	Logit	***	0.000819	[0.121, 0.467]	CR
Ratio halting to total trial duration H7 (diff. Zone 1 - Zone 2)	Logit	**	0.00404	[0.156, 0.622]	CR
Ratio movement to detection duration (diff. Zone 1 - Zone 2)	Logit	**	0.00335	[1.07, 1.36]	
Ratio movement to detection duration H0 (diff. Zone 1 - Zone 2)	Logit	**	0.0076	[1.06, 1.45]	CR
Ratio movement to detection duration H1 (diff. Zone 1 - Zone 2)	Logit	*	0.0233	[1.03, 1.39]	
Ratio movement to detection duration H2 (diff. Zone 1 - Zone 2)	Logit	**	0.00569	[1.08, 1.54]	
Ratio movement to detection duration H3 (diff. Zone 1 - Zone 2)	Logit		0.844	[0.803, 1.2]	
Ratio movement to detection duration H4 (diff. Zone 1 - Zone 2)	Logit		0.166	[0.917, 1.54]	
Ratio movement to detection duration H5 (diff. Zone 1 - Zone 2)	Logit		0.367	[0.827, 1.59]	
Ratio movement to detection duration H6 (diff. Zone 1 - Zone 2)	Logit	*	0.0353	[1.02, 1.78]	
Ratio movement to detection duration H7 (diff. Zone 1 - Zone 2)	Logit		0.441	[0.778, 1.66]	
Ratio movement to halting duration (diff. Zone 1 - Zone 2)	Logit	**	0.00183	[1.31, 2.33]	
Ratio movement to halting duration H0 (diff. Zone 1 - Zone 2)	Logit	*	0.0143	[1.29, 6.37]	
Ratio movement to halting duration H1 (diff. Zone 1 - Zone 2)	Logit		0.142	[0.732, 6.21]	
Ratio movement to halting duration H2 (diff. Zone 1 - Zone 2)	Logit		0.0535	[0.984, 5.34]	
Ratio movement to halting duration H3 (diff. Zone 1 - Zone 2)	Logit		0.196	[0.678, 5.51]	
Ratio movement to halting duration H4 (diff. Zone 1 - Zone 2)	Logit		0.204	[0.672, 5.05]	
Ratio movement to halting duration H5 (diff. Zone 1 - Zone 2)	Logit		0.0525	[0.985, 6.87]	
Ratio movement to halting duration H6 (diff. Zone 1 - Zone 2)	Logit	****	1.31E-05	[2.37, 6.83]	
Ratio movement to halting duration H7 (diff. Zone 1 - Zone 2)	Logit		0.087	[0.84, 7.22]	

(p ≤ 0.05)*, (p ≤ 0.01)**, (p ≤ 0.001)***, (p ≤ 0.0001)****
CR = Check residuals

Behaviour statistic correlation analysis

Spearman rank correlation matrix

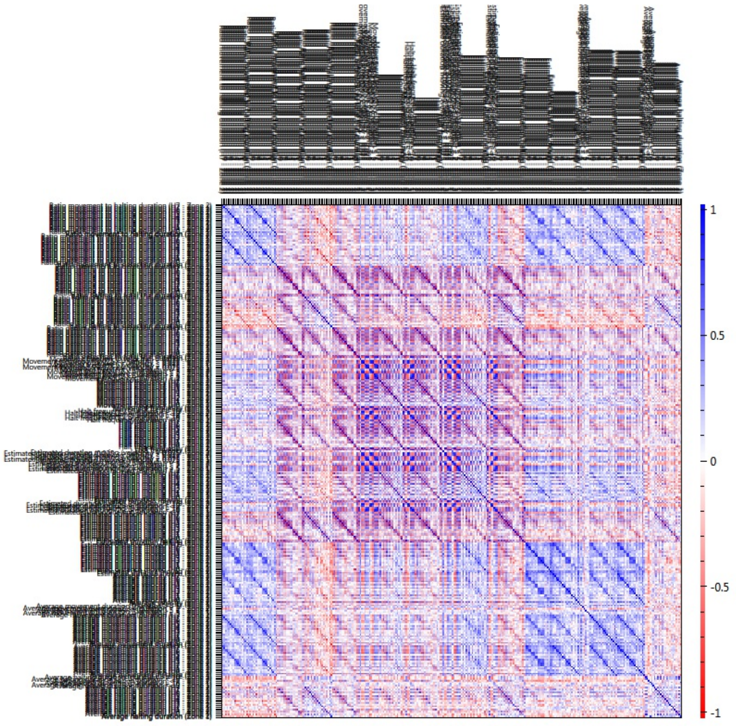

Average halting duration per zone

Selected zones	Zone 1, Zone 2
Data transformation	Natural logarithm
Analysis	Zone difference analysis

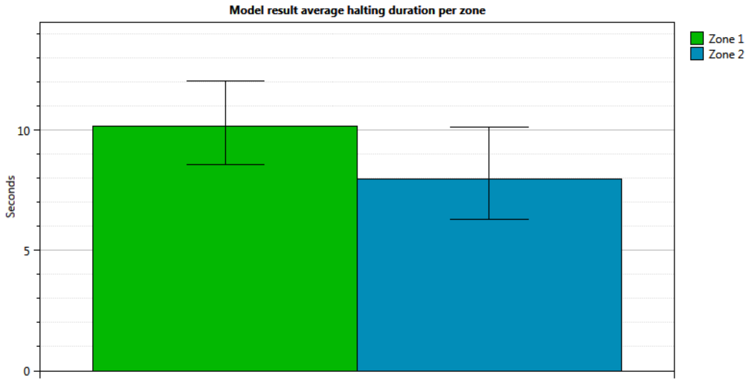

Results difference tests Zone 1 - Zone 2: p values and 95% confidence intervals of the difference on the transformed scale for each statistic.

Behaviour statistic	Cur3-RMX180
Average halting duration (diff. Zone 1 - Zone 2)	p=0.00501**
	[0.0842, 0.441]

The model predictions and 95% confidence intervals for each statistic.

Statistic	Cur3-RMX180	Remark
Average halting duration (Zone 1)	10.2	
	[8.59, 12.1]	
Average halting duration (Zone 2)	7.99	CR
	[6.3, 10.1]	

CR = Check residuals

Data summary

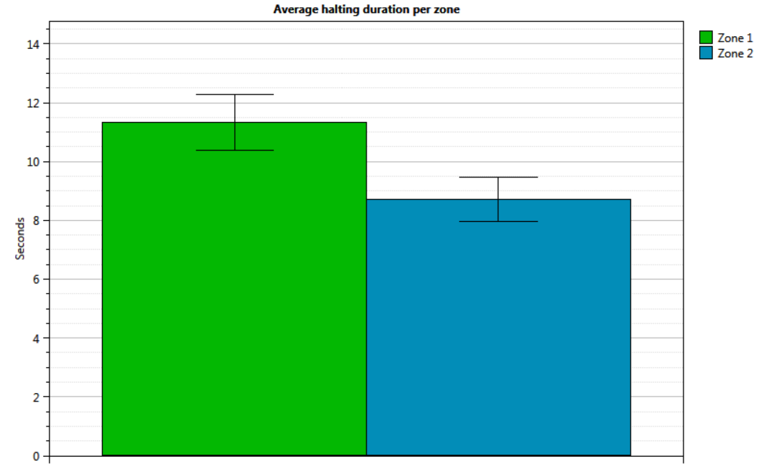

Genotype Zone 1	Genotype Zone 2	Genotype Zone 3	Mean Zone 1	StdErr Zone 1	Mean Zone 2	StdErr Zone 2
Cur3	RMX180	Neutral	11.34	0.96	8.72	0.76

Analysis average halting duration (Zone 1)

**Analysis model**	Linear mixed model fit by REML: Average_halting_duration_Zone_1 ~ 1 + (1|Genotype_Zone_1:Plant_Zone_1) + (1|Genotype_Zone_2:Plant_Zone_2)
**Transformation**	Natural logarithm

Confidence intervals average halting duration (Zone 1)

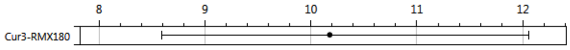

Confidence intervals average halting duration (Zone 1)

Genotype Zone 1	Genotype Zone 2	Mean	Lower 95% CL	Upper 95% CL	Group
Cur3	RMX180	10.18	8.589	12.06	a

Model summary

```
Linear mixed model fit by REML. t-tests use Satterthwaite's method ['lmerModLmerTest']
Formula: Average_halting_duration_Zone_1 ~ 1 + (1 | Genotype_Zone_1:Plant_Zone_1) +      (1 |
Genotype_Zone_2:Plant_Zone_2)
   Data: data

REML criterion at convergence: 51.4

Scaled residuals: 
    Min      1Q  Median      3Q     Max 
-2.2956 -0.6483 -0.1858  0.4395  2.3936 

Random effects:
 Groups                       Name        Variance Std.Dev.
 Genotype_Zone_1:Plant_Zone_1 (Intercept) 0.000000 0.00000 
 Genotype_Zone_2:Plant_Zone_2 (Intercept) 0.003947 0.06282 
 Residual                                 0.202193 0.44966 
Number of obs: 39, groups:  Genotype_Zone_1:Plant_Zone_1, 10; Genotype_Zone_2:Plant_Zone_2, 10

Fixed effects:
            Estimate Std. Error      df t value Pr(>|t|)    
(Intercept)  2.32015    0.07471 8.78456   31.06 2.75e-10 ***
---
Signif. codes:  0 '***' 0.001 '**' 0.01 '*' 0.05 '.' 0.1 ' ' 1
```

Model residuals

Statistic	Value
Sample skewness	0.5706
Sample excess kurtosis	0.5048
Passed Shapiro Wilk normality test	Yes (p-value = 0.07023 > 0.05)

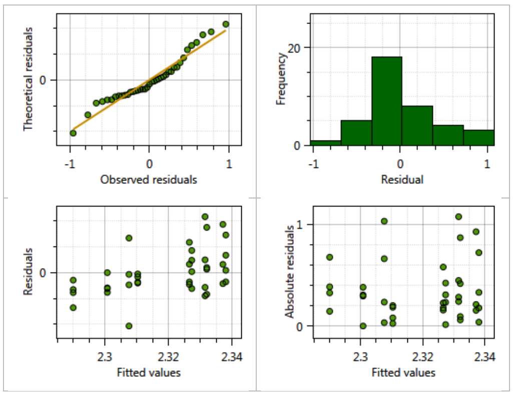

Analysis average halting duration (Zone 2)

**Analysis model**	Linear mixed model fit by REML: Average_halting_duration_Zone_2 ~ 1 + (1|Genotype_Zone_1:Plant_Zone_1) + (1|Genotype_Zone_2:Plant_Zone_2)
**Transformation**	Natural logarithm

Confidence intervals average halting duration (Zone 2)

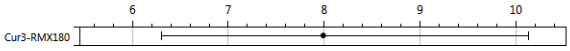

Confidence intervals average halting duration (Zone 2)

Genotype Zone 1	Genotype Zone 2	Mean	Lower 95% CL	Upper 95% CL	Group
Cur3	RMX180	7.991	6.299	10.14	a

Model summary

```
Linear mixed model fit by REML. t-tests use Satterthwaite's method ['lmerModLmerTest']
Formula: Average_halting_duration_Zone_2 ~ 1 + (1 | Genotype_Zone_1:Plant_Zone_1) +      (1 |
Genotype_Zone_2:Plant_Zone_2)
   Data: data

REML criterion at convergence: 40.5
```

```
Scaled residuals:
    Min      1Q  Median      3Q     Max
-1.4660 -0.6630 -0.1507  0.4058  3.2737

Random effects:
 Groups                       Name        Variance Std.Dev.
 Genotype_Zone_1:Plant_Zone_1 (Intercept) 0.013011 0.11407
 Genotype_Zone_2:Plant_Zone_2 (Intercept) 0.007342 0.08568
 Residual                                 0.137537 0.37086
Number of obs: 39, groups:  Genotype_Zone_1:Plant_Zone_1, 10; Genotype_Zone_2:Plant_Zone_2, 10

Fixed effects:
            Estimate Std. Error      df t value Pr(>|t|)
(Intercept)  2.07828    0.07464 2.99431   27.84 0.000103 ***
---
Signif. codes:  0 '***' 0.001 '**' 0.01 '*' 0.05 '.' 0.1 ' ' 1
```

Model residuals

Statistic	Value
Sample skewness	1.319
Sample excess kurtosis	2.428
Passed Shapiro Wilk normality test	No (p-value = 0.003259 < 0.05)

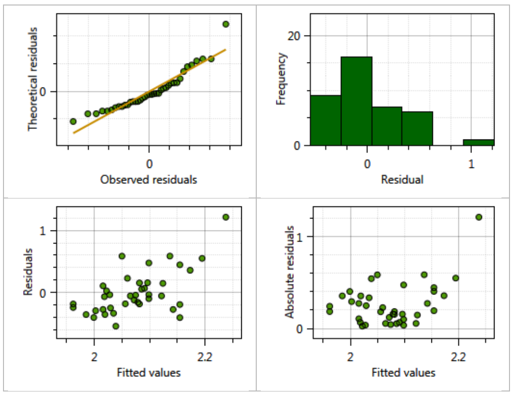

Data points with high residuals

Trial	Arena
Trial 3	12

Analysis average halting duration (diff. Zone 1 - Zone 2)

**Analysis model**	Generalized linear mixed model with dispersion factor, formula=cbind(Average_halting_duration_Zone_1,Average_halting_duration_Zone_2) ~ 1 + (1|Genotype_Zone_1:Plant_Zone_1) + (1|Genotype_Zone_2:Plant_Zone_2)
**Transformation**	Logit

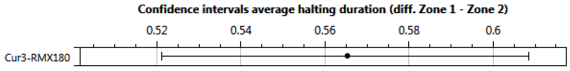

Confidence intervals average halting duration (diff. Zone 1 - Zone 2)

Genotype Zone 1	Genotype Zone 2	Mean	Lower 95% CL	Upper 95% CL	Group
Cur3	RMX180	0.5653	0.521	0.6085	a

Model summary

```
Linear mixed model fit by REML. t-tests use Satterthwaite's method ['lmerModLmerTest']
Formula: ziFormula
   Data: data
Weights: wi

REML criterion at convergence: 68.9

Scaled residuals:
     Min       1Q   Median       3Q      Max
-3.14144 -0.46288 -0.04596  0.53432  2.16217

Random effects:
 Groups                       Name        Variance Std.Dev.
 Genotype_Zone_1:Plant_Zone_1 (Intercept) 0.000    0.000
 Genotype_Zone_2:Plant_Zone_2 (Intercept) 0.000    0.000
 Residual                                 1.494    1.222
Number of obs: 39, groups:  Genotype_Zone_1:Plant_Zone_1, 10; Genotype_Zone_2:Plant_Zone_2, 10

Fixed effects:
            Estimate Std. Error       df t value Pr(>|t|)
(Intercept)  0.26270    0.08816 38.00000    2.98  0.00501 **
---
Signif. codes:  0 '***' 0.001 '**' 0.01 '*' 0.05 '.' 0.1 ' ' 1

Dispersion: 1.222
```

Model residuals

Statistic	Value
Sample skewness	-0.3915
Sample excess kurtosis	1.938
Passed Shapiro Wilk normality test	Yes (p-value = 0.1407 > 0.05)

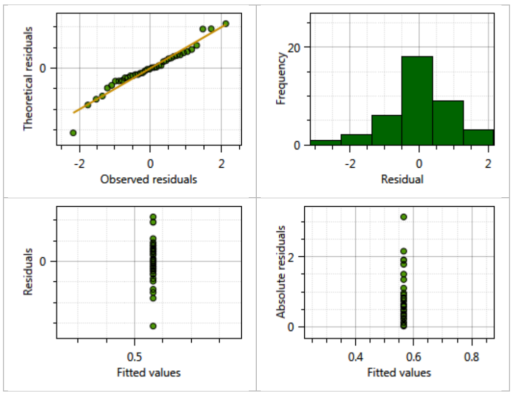

Average halting duration per zone per hour

Selected hours	0, 1, 2, 3, 4, 5, 6, 7
Selected zones	Zone 1, Zone 2
Data transformation	Natural logarithm
Analysis	Zone difference analysis

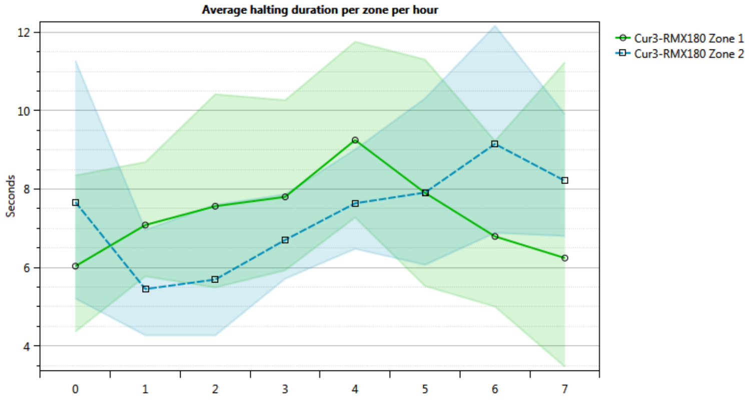

Results difference tests Zone 1 - Zone 2: p values and 95% confidence intervals of the difference on the transformed scale for each statistic.

Behaviour statistic	Cur3-RMX180	Remark
Average halting duration H0 (diff. Zone 1 - Zone 2)	p=0.375<br>[-1.21, 0.483]	
Average halting duration H1 (diff. Zone 1 - Zone 2)	p=0.152<br>[-0.111, 0.629]	CR
Average halting duration H2 (diff. Zone 1 - Zone 2)	p=0.126<br>[-0.113, 0.779]	CR
Average halting duration H3 (diff. Zone 1 - Zone 2)	p=0.0145*<br>[0.0848, 0.614]	
Average halting duration H4 (diff. Zone 1 - Zone 2)	p=0.23<br>[-0.125, 0.448]	
Average halting duration H5 (diff. Zone 1 - Zone 2)	p=0.284<br>[-0.238, 0.722]	
Average halting duration H6 (diff. Zone 1 - Zone 2)	p=0.269<br>[-0.634, 0.204]	
Average halting duration H7 (diff. Zone 1 - Zone 2)	p=0.955<br>[-0.644, 0.678]	

CR = Check residuals

The model predictions and 95% confidence intervals for each statistic.

Statistic	Cur3-RMX180	Remark
Average halting duration (H0 - Zone 1)	6.03<br>[4.36, 8.35]	
Average halting duration (H0 - Zone 2)	7.67<br>[5.21, 11.3]	CR
Average halting duration (H1 - Zone 1)	7.08<br>[5.77, 8.69]	
Average halting duration (H1 - Zone 2)	5.45<br>[4.27, 6.96]	CR
Average halting duration (H2 - Zone 1)	7.56<br>[5.49, 10.4]	
Average halting duration (H2 - Zone 2)	5.69<br>[4.27, 7.6]	CR
Average halting duration (H3 - Zone 1)	7.8<br>[5.93, 10.3]	CR
Average halting duration (H3 - Zone 2)	6.7<br>[5.71, 7.87]	CR
Average halting duration (H4 - Zone 1)	9.25<br>[7.28, 11.8]	
Average halting duration (H4 - Zone 2)	7.64<br>[6.48, 9.01]	CR
Average halting duration (H5 - Zone 1)	7.9<br>[5.53, 11.3]	CR
Average halting duration (H5 - Zone 2)	7.91<br>[6.07, 10.3]	
Average halting duration (H6 - Zone 1)	6.79<br>[5, 9.23]	
Average halting duration (H6 - Zone 2)	9.15<br>[6.88, 12.2]	
Average halting duration (H7 - Zone 1)	6.24<br>[3.47, 11.2]	
Average halting duration (H7 - Zone 2)	8.21<br>[6.8, 9.91]	

CR = Check residuals

Data summary

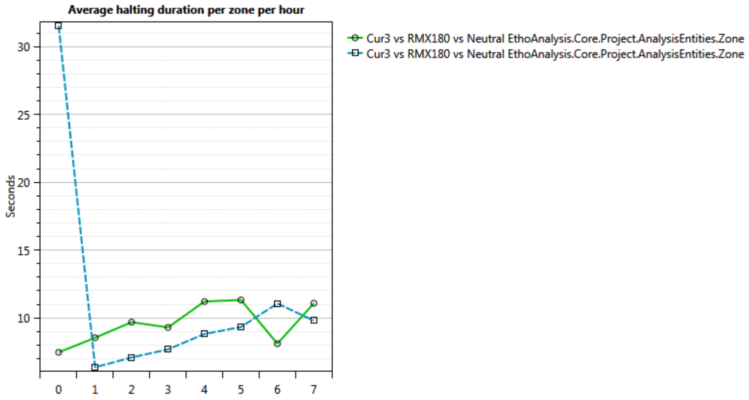

Genotype Zone 1	Genotype Zone 2	Genotype Zone 3	Mean H0 - Zone 1	StdErr H0 - Zone 1	Mean H0 - Zone 2	StdErr H0 - Zone 2	Mean H1 - Zone 1	StdErr H1 - Zone 1	Mean H1 - Zone 2	StdErr H1 - Zone 2	Mean H2 - Zone 1	StdErr H2 - Zone 1	Mean H2 - Zone 2	StdErr H2 - Zone 2	Mean H3 - Zone 1	StdErr H3 - Zone 1	Mean H3 - Zone 2	StdErr H3 - Zone 2	Mean H4 - Zone 1	StdErr H4 - Zone 1	Mean H4 - Zone 2	StdErr H4 - Zone 2	Mean H5 - Zone 1	StdErr H5 - Zone 1	Mean H5 - Zone 2	StdErr H5 - Zone 2	Mean H6 - Zone 1	StdErr H6 - Zone 1	Mean H6 - Zone 2	StdErr H6 - Zone 2
Cur3	RMX180	Neutral	7.47	0.99	31.53	23.15	8.54	1.01	6.36	0.65	9.69	1.27	7.09	0.76	9.3	0.83	7.68	0.78	11.21	1.41	8.82	0.98	11.33	1.72	9.33	0.98	8.1	1.03	11.06	1.39

Analysis average halting duration (H0 - Zone 1)

**Analysis model**	Linear mixed model fit by REML: Average_halting_duration_H0_Zone_1 ~ 1 + (1|Genotype_Zone_1:Plant_Zone_1) + (1|Genotype_Zone_2:Plant_Zone_2)
**Transformation**	Natural logarithm

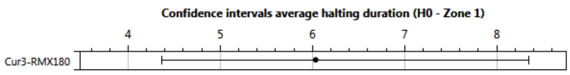

Confidence intervals average halting duration (H0 - Zone 1)

Genotype Zone 1	Genotype Zone 2	Mean	Lower 95% CL	Upper 95% CL	Group
Cur3	RMX180	6.035	4.362	8.35	a

Model summary

```
Linear mixed model fit by REML. t-tests use Satterthwaite's method ['lmerModLmerTest']
Formula: Average_halting_duration_H0_Zone_1 ~ 1 + (1 | Genotype_Zone_1:Plant_Zone_1) +      (1 |
Genotype_Zone_2:Plant_Zone_2)
   Data: data

REML criterion at convergence: 71.1

Scaled residuals:
     Min       1Q   Median       3Q      Max
-1.92583 -0.76676  0.05571  0.44182  1.94882

Random effects:
 Groups                       Name        Variance Std.Dev.
 Genotype_Zone_1:Plant_Zone_1 (Intercept) 0.0782   0.2796
 Genotype_Zone_2:Plant_Zone_2 (Intercept) 0.0000   0.0000
 Residual                                 0.3950   0.6285
Number of obs: 34, groups:  Genotype_Zone_1:Plant_Zone_1, 10; Genotype_Zone_2:Plant_Zone_2, 10

Fixed effects:
            Estimate Std. Error     df t value Pr(>|t|)
(Intercept)   1.7976     0.1401 7.7753   12.83 1.65e-06 ***
---
Signif. codes:  0 '***' 0.001 '**' 0.01 '*' 0.05 '.' 0.1 ' ' 1
```

Model residuals

Statistic	Value
Sample skewness	0.07507
Sample excess kurtosis	-0.2603
Passed Shapiro Wilk normality test	Yes (p-value = 0.743 > 0.05)

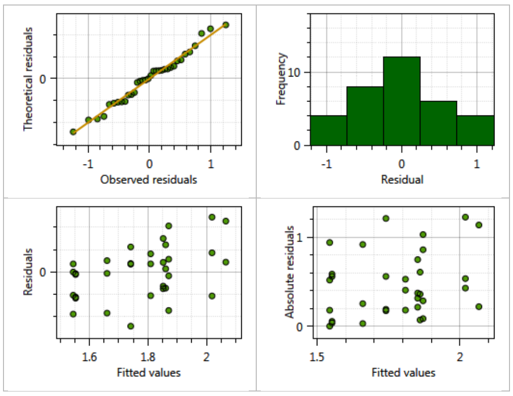

Analysis average halting duration (H0 - Zone 2)

**Analysis model**	Linear mixed model fit by REML: Average_halting_duration_H0_Zone_2 ~ 1 + (1|Genotype_Zone_1:Plant_Zone_1) + (1|Genotype_Zone_2:Plant_Zone_2)
**Transformation**	Natural logarithm

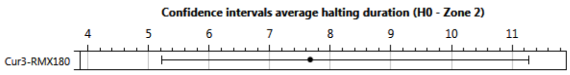

Confidence intervals average halting duration (H0 - Zone 2)

Genotype Zone 1	Genotype Zone 2	Mean	Lower 95% CL	Upper 95% CL	Group
Cur3	RMX180	7.668	5.212	11.28	a

Model summary

```
Linear mixed model fit by REML. t-tests use Satterthwaite's method ['lmerModLmerTest']
Formula: Average_halting_duration_H0_Zone_2 ~ 1 + (1 | Genotype_Zone_1:Plant_Zone_1) +      (1 |
Genotype_Zone_2:Plant_Zone_2)
   Data: data

REML criterion at convergence: 105.8

Scaled residuals:
    Min      1Q  Median      3Q     Max
-1.0101 -0.4218 -0.2981  0.1139  4.8864

Random effects:
 Groups                       Name        Variance  Std.Dev.
 Genotype_Zone_1:Plant_Zone_1 (Intercept) 2.175e-02 1.475e-01
 Genotype_Zone_2:Plant_Zone_2 (Intercept) 3.881e-15 6.230e-08
 Residual                                 9.057e-01 9.517e-01
Number of obs: 38, groups:  Genotype_Zone_1:Plant_Zone_1, 10; Genotype_Zone_2:Plant_Zone_2, 10

Fixed effects:
            Estimate Std. Error     df t value Pr(>|t|)
(Intercept)   2.0370     0.1613 6.6202   12.62 7.08e-06 ***
---
Signif. codes:  0 '***' 0.001 '**' 0.01 '*' 0.05 '.' 0.1 ' ' 1
```

Model residuals

Statistic	Value
Sample skewness	3.682
Sample excess kurtosis	16.46
Passed Shapiro Wilk normality test	No (p-value = 6.594E-09 < 0.05)

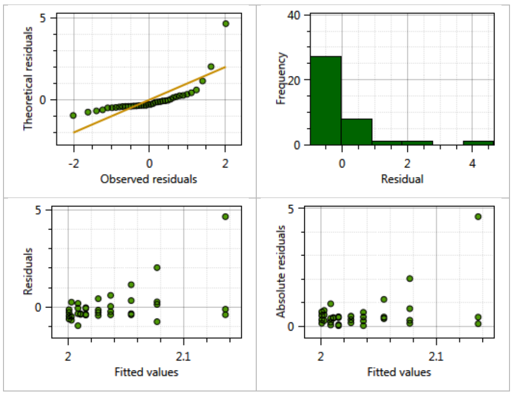

Data points with high residuals

Trial	Arena
Trial 3	25

Analysis average halting duration (H1 - Zone 1)

**Analysis model**	Linear mixed model fit by REML: Average_halting_duration_H1_Zone_1 ~ 1 + (1|Genotype_Zone_1:Plant_Zone_1) + (1|Genotype_Zone_2:Plant_Zone_2)
**Transformation**	Natural logarithm

Confidence intervals average halting duration (H1 - Zone 1)

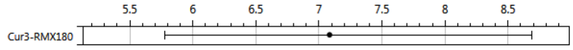

Confidence intervals average halting duration (H1 - Zone 1)

Genotype Zone 1	Genotype Zone 2	Mean	Lower 95% CL	Upper 95% CL	Group
Cur3	RMX180	7.084	5.775	8.69	a

Model summary

```
Linear mixed model fit by REML. t-tests use Satterthwaite's method ['lmerModLmerTest']
Formula: Average_halting_duration_H1_Zone_1 ~ 1 + (1 | Genotype_Zone_1:Plant_Zone_1) +      (1 |
Genotype_Zone_2:Plant_Zone_2)
   Data: data

REML criterion at convergence: 67.6

Scaled residuals:
     Min       1Q   Median       3Q      Max
-2.41261 -0.76989 -0.00741  0.49657  2.54776

Random effects:
 Groups                       Name        Variance Std.Dev.
 Genotype_Zone_1:Plant_Zone_1 (Intercept) 0.0000   0.0000
 Genotype_Zone_2:Plant_Zone_2 (Intercept) 0.0000   0.0000
 Residual                                 0.3647   0.6039
Number of obs: 36, groups:  Genotype_Zone_1:Plant_Zone_1, 10; Genotype_Zone_2:Plant_Zone_2, 10

Fixed effects:
            Estimate Std. Error      df t value Pr(>|t|)
(Intercept)   1.9578     0.1007 35.0000   19.45   <2e-16 ***
---
Signif. codes:  0 '***' 0.001 '**' 0.01 '*' 0.05 '.' 0.1 ' ' 1
```

Model residuals

Statistic	Value
Sample skewness	0.2652
Sample excess kurtosis	0.4891
Passed Shapiro Wilk normality test	Yes (p-value = 0.7121 > 0.05)

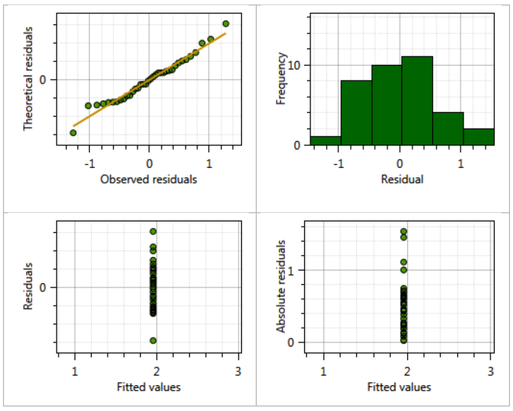

Analysis average halting duration (H1 - Zone 2)

**Analysis model**	Linear mixed model fit by REML: Average_halting_duration_H1_Zone_2 ~ 1 + (1|Genotype_Zone_1:Plant_Zone_1) + (1|Genotype_Zone_2:Plant_Zone_2)
**Transformation**	Natural logarithm

Confidence intervals average halting duration (H1 - Zone 2)

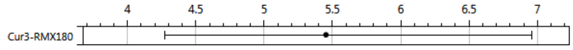

Confidence intervals average halting duration (H1 - Zone 2)

Genotype Zone 1	Genotype Zone 2	Mean	Lower 95% CL	Upper 95% CL	Group
Cur3	RMX180	5.452	4.27	6.961	a

Model summary

```
Linear mixed model fit by REML. t-tests use Satterthwaite's method ['lmerModLmerTest']
Formula: Average_halting_duration_H1_Zone_2 ~ 1 + (1 | Genotype_Zone_1:Plant_Zone_1) +      (1 |
Genotype_Zone_2:Plant_Zone_2)
   Data: data

REML criterion at convergence: 75.4

Scaled residuals:
    Min      1Q  Median      3Q     Max
```

```
-4.4738 -0.1726  0.0960  0.4564  2.4451

Random effects:
 Groups                       Name        Variance Std.Dev.
 Genotype_Zone_1:Plant_Zone_1 (Intercept) 0.0140   0.1183
 Genotype_Zone_2:Plant_Zone_2 (Intercept) 0.0000   0.0000
 Residual                                 0.3952   0.6287
Number of obs: 38, groups:  Genotype_Zone_1:Plant_Zone_1, 10; Genotype_Zone_2:Plant_Zone_2, 10

Fixed effects:
            Estimate Std. Error     df t value Pr(>|t|)
(Intercept)   1.6960     0.1087 9.3867    15.6 4.99e-08 ***
---
Signif. codes:  0 '***' 0.001 '**' 0.01 '*' 0.05 '.' 0.1 ' ' 1
```

Model residuals

Statistic	Value
Sample skewness	-2.325
Sample excess kurtosis	11.79
Passed Shapiro Wilk normality test	No (p-value = 1.133E-06 < 0.05)

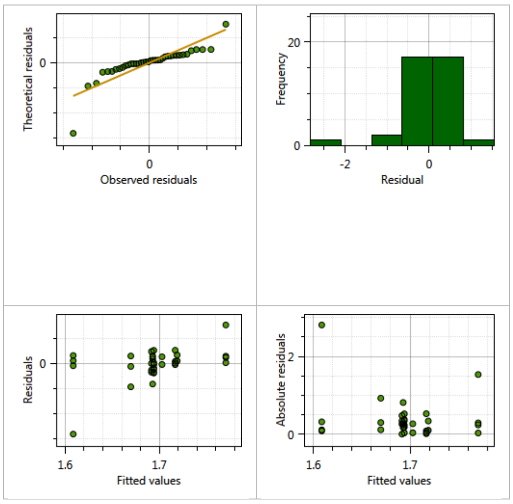

Analysis average halting duration (H2 - Zone 1)

**Analysis model**	Linear mixed model fit by REML: Average_halting_duration_H2_Zone_1 ~ 1 + (1|Genotype_Zone_1:Plant_Zone_1) + (1|Genotype_Zone_2:Plant_Zone_2)
**Transformation**	Natural logarithm

Confidence intervals average halting duration (H2 - Zone 1)

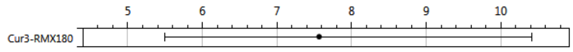

Confidence intervals average halting duration (H2 - Zone 1)

Genotype Zone 1	Genotype Zone 2	Mean	Lower 95% CL	Upper 95% CL	Group
Cur3	RMX180	7.564	5.491	10.42	a

Model summary

```
Linear mixed model fit by REML. t-tests use Satterthwaite's method ['lmerModLmerTest']
Formula: Average_halting_duration_H2_Zone_1 ~ 1 + (1 | Genotype_Zone_1:Plant_Zone_1) +      (1 |
Genotype_Zone_2:Plant_Zone_2)
   Data: data

REML criterion at convergence: 77.8

Scaled residuals:
    Min      1Q  Median      3Q     Max
-1.4858 -0.7845 -0.1022  0.6327  1.8255

Random effects:
 Groups                       Name        Variance Std.Dev.
 Genotype_Zone_1:Plant_Zone_1 (Intercept) 0.066548 0.25797
 Genotype_Zone_2:Plant_Zone_2 (Intercept) 0.001158 0.03404
 Residual                                 0.435447 0.65988
Number of obs: 36, groups:  Genotype_Zone_1:Plant_Zone_1, 10; Genotype_Zone_2:Plant_Zone_2, 10

Fixed effects:
            Estimate Std. Error    df t value Pr(>|t|)
(Intercept)    2.023      0.138 7.705   14.67 6.63e-07 ***
---
Signif. codes:  0 '***' 0.001 '**' 0.01 '*' 0.05 '.' 0.1 ' ' 1
```

Model residuals

Statistic	Value
Sample skewness	0.4355
Sample excess kurtosis	-0.7754
Passed Shapiro Wilk normality test	Yes (p-value = 0.07148 > 0.05)

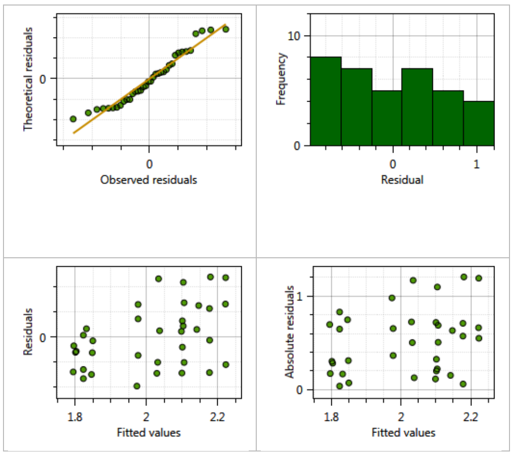

Analysis average halting duration (H2 - Zone 2)

**Analysis model**	Linear mixed model fit by REML: Average_halting_duration_H2_Zone_2 ~ 1 + (1|Genotype_Zone_1:Plant_Zone_1) + (1|Genotype_Zone_2:Plant_Zone_2)
**Transformation**	Natural logarithm

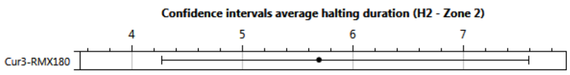

Confidence intervals average halting duration (H2 - Zone 2)

Genotype Zone 1	Genotype Zone 2	Mean	Lower 95% CL	Upper 95% CL	Group
Cur3	RMX180	5.694	4.269	7.596	a

Model summary

```
Linear mixed model fit by REML. t-tests use Satterthwaite's method ['lmerModLmerTest']
Formula: Average_halting_duration_H2_Zone_2 ~ 1 + (1 | Genotype_Zone_1:Plant_Zone_1) +      (1 |
Genotype_Zone_2:Plant_Zone_2)
   Data: data

REML criterion at convergence: 89.8

Scaled residuals: 
    Min      1Q  Median      3Q     Max 
-3.7883 -0.1618  0.0192  0.5143  1.8772 

Random effects:
 Groups                       Name        Variance Std.Dev.
 Genotype_Zone_1:Plant_Zone_1 (Intercept) 0.000000 0.00000 
 Genotype_Zone_2:Plant_Zone_2 (Intercept) 0.005714 0.07559 
 Residual                                 0.595878 0.77193 
Number of obs: 38, groups:  Genotype_Zone_1:Plant_Zone_1, 10; Genotype_Zone_2:Plant_Zone_2, 10

Fixed effects:
            Estimate Std. Error     df t value Pr(>|t|)    
(Intercept)   1.7395     0.1275 9.0543   13.64 2.41e-07 ***
---
Signif. codes:  0 '***' 0.001 '**' 0.01 '*' 0.05 '.' 0.1 ' ' 1
```

Model residuals

Statistic	Value
Sample skewness	-1.761
Sample excess kurtosis	5.815
Passed Shapiro Wilk normality test	No (p-value = 3.917E-05 < 0.05)

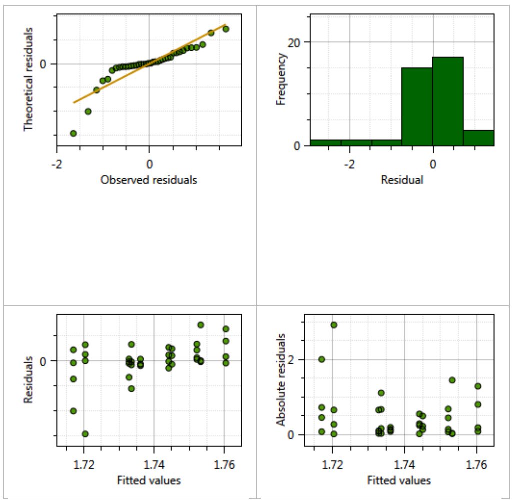

Analysis average halting duration (H3 - Zone 1)

**Analysis model**	Linear mixed model fit by REML: Average_halting_duration_H3_Zone_1 ~ 1 + (1|Genotype_Zone_1:Plant_Zone_1) + (1|Genotype_Zone_2:Plant_Zone_2)
**Transformation**	Natural logarithm

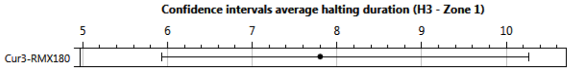

Confidence intervals average halting duration (H3 - Zone 1)

Genotype Zone 1	Genotype Zone 2	Mean	Lower 95% CL	Upper 95% CL	Group
Cur3	RMX180	7.802	5.928	10.27	a

Model summary

```
Linear mixed model fit by REML. t-tests use Satterthwaite's method ['lmerModLmerTest']
Formula: Average_halting_duration_H3_Zone_1 ~ 1 + (1 | Genotype_Zone_1:Plant_Zone_1) +      (1 |
Genotype_Zone_2:Plant_Zone_2)
   Data: data

REML criterion at convergence: 69.9

Scaled residuals: 
    Min      1Q  Median      3Q     Max 
-3.1427 -0.3646  0.1673  0.7421  1.2457 

Random effects:
 Groups                       Name        Variance Std.Dev.
 Genotype_Zone_1:Plant_Zone_1 (Intercept) 0.000000 0.00000 
 Genotype_Zone_2:Plant_Zone_2 (Intercept) 0.003903 0.06247 
 Residual                                 0.462812 0.68030 
Number of obs: 33, groups:  Genotype_Zone_1:Plant_Zone_1, 10; Genotype_Zone_2:Plant_Zone_2, 10

Fixed effects:
            Estimate Std. Error     df t value Pr(>|t|)    
(Intercept)   2.0544     0.1202 8.4241    17.1 7.78e-08 ***
---
Signif. codes:  0 '***' 0.001 '**' 0.01 '*' 0.05 '.' 0.1 ' ' 1
```

Model residuals

Statistic	Value
Sample skewness	-1.351
Sample excess kurtosis	2.345
Passed Shapiro Wilk normality test	No (p-value = 0.002548 < 0.05)

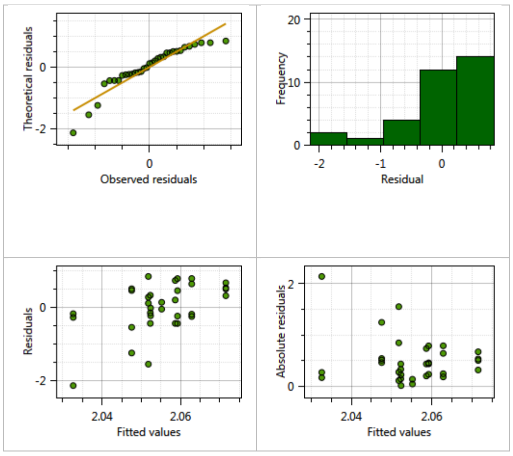

Analysis average halting duration (H3 - Zone 2)

**Analysis model**	Linear mixed model fit by REML: Average_halting_duration_H3_Zone_2 ~ 1 + (1|Genotype_Zone_1:Plant_Zone_1) + (1|Genotype_Zone_2:Plant_Zone_2)
**Transformation**	Natural logarithm

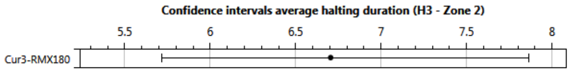

Confidence intervals average halting duration (H3 - Zone 2)

Genotype Zone 1	Genotype Zone 2	Mean	Lower 95% CL	Upper 95% CL	Group
Cur3	RMX180	6.704	5.715	7.865	a

Model summary

```
Linear mixed model fit by REML. t-tests use Satterthwaite's method ['lmerModLmerTest']
Formula: Average_halting_duration_H3_Zone_2 ~ 1 + (1 | Genotype_Zone_1:Plant_Zone_1) +      (1 |
Genotype_Zone_2:Plant_Zone_2)
   Data: data

REML criterion at convergence: 57.7

Scaled residuals:
    Min      1Q  Median      3Q     Max
-1.7503 -0.5595 -0.3436  0.6613  2.6563

Random effects:
 Groups                       Name        Variance  Std.Dev.
 Genotype_Zone_1:Plant_Zone_1 (Intercept) 0.000e+00 0.000e+00
 Genotype_Zone_2:Plant_Zone_2 (Intercept) 3.843e-15 6.199e-08
 Residual                                 2.428e-01 4.928e-01
Number of obs: 39, groups:  Genotype_Zone_1:Plant_Zone_1, 10; Genotype_Zone_2:Plant_Zone_2, 10

Fixed effects:
            Estimate Std. Error      df t value Pr(>|t|)
(Intercept)   1.9027     0.0789 38.0000   24.11   <2e-16 ***
---
Signif. codes:  0 '***' 0.001 '**' 0.01 '*' 0.05 '.' 0.1 ' ' 1
```

Model residuals

Statistic	Value
Sample skewness	0.9489
Sample excess kurtosis	0.7666
Passed Shapiro Wilk normality test	No (p-value = 0.005579 < 0.05)

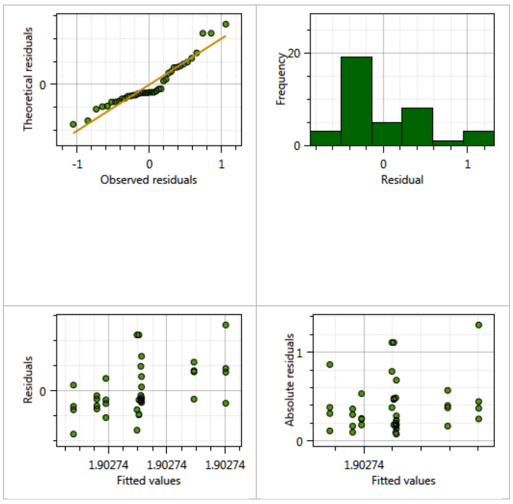

Analysis average halting duration (H4 - Zone 1)

**Analysis model**	Linear mixed model fit by REML: Average_halting_duration_H4_Zone_1 ~ 1 + (1|Genotype_Zone_1:Plant_Zone_1) + (1|Genotype_Zone_2:Plant_Zone_2)
**Transformation**	Natural logarithm

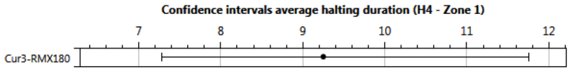

Confidence intervals average halting duration (H4 - Zone 1)

Genotype Zone 1	Genotype Zone 2	Mean	Lower 95% CL	Upper 95% CL	Group
Cur3	RMX180	9.25	7.276	11.76	a

Model summary

```
Linear mixed model fit by REML. t-tests use Satterthwaite's method ['lmerModLmerTest']
Formula: Average_halting_duration_H4_Zone_1 ~ 1 + (1 | Genotype_Zone_1:Plant_Zone_1) +      (1 |
Genotype_Zone_2:Plant_Zone_2)
   Data: data
```

```
REML criterion at convergence: 57

Scaled residuals:
    Min      1Q  Median      3Q     Max
-2.4115 -0.6861 -0.1794  0.5850  2.1743

Random effects:
 Groups                       Name        Variance Std.Dev.
 Genotype_Zone_1:Plant_Zone_1 (Intercept) 0.0000   0.000
 Genotype_Zone_2:Plant_Zone_2 (Intercept) 0.0000   0.000
 Residual                                 0.3981   0.631
Number of obs: 29, groups:  Genotype_Zone_1:Plant_Zone_1, 10; Genotype_Zone_2:Plant_Zone_2, 10

Fixed effects:
            Estimate Std. Error      df t value Pr(>|t|)
(Intercept)   2.2246     0.1172 28.0000   18.99   <2e-16 ***
---
Signif. codes:  0 '***' 0.001 '**' 0.01 '*' 0.05 '.' 0.1 ' ' 1
```

Model residuals

Statistic	Value
Sample skewness	0.02392
Sample excess kurtosis	0.1875
Passed Shapiro Wilk normality test	Yes (p-value = 0.9624 > 0.05)

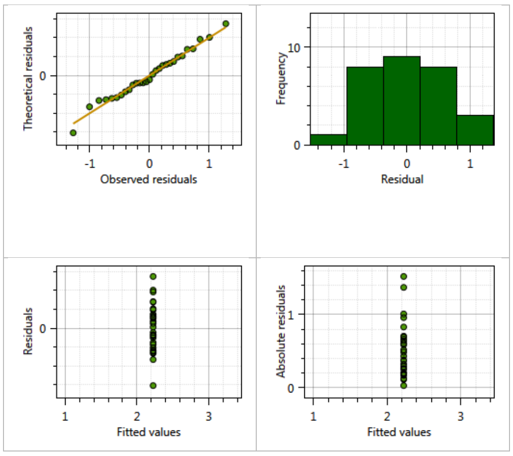

Analysis average halting duration (H4 - Zone 2)

**Analysis model**	Linear mixed model fit by REML: Average_halting_duration_H4_Zone_2 ~ 1 + (1|Genotype_Zone_1:Plant_Zone_1) + (1|Genotype_Zone_2:Plant_Zone_2)
**Transformation**	Natural logarithm

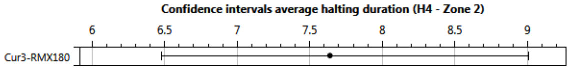

Confidence intervals average halting duration (H4 - Zone 2)

Genotype Zone 1	Genotype Zone 2	Mean	Lower 95% CL	Upper 95% CL	Group
Cur3	RMX180	7.638	6.476	9.01	a

Model summary

```
Linear mixed model fit by REML. t-tests use Satterthwaite's method ['lmerModLmerTest']
Formula: Average_halting_duration_H4_Zone_2 ~ 1 + (1 | Genotype_Zone_1:Plant_Zone_1) +      (1 |
Genotype_Zone_2:Plant_Zone_2)
   Data: data

REML criterion at convergence: 55.2

Scaled residuals:
    Min      1Q  Median      3Q     Max
-1.1936 -0.6855 -0.3604  0.3866  3.0120

Random effects:
 Groups                       Name        Variance  Std.Dev.
 Genotype_Zone_1:Plant_Zone_1 (Intercept) 7.258e-17 8.520e-09
 Genotype_Zone_2:Plant_Zone_2 (Intercept) 1.758e-19 4.192e-10
 Residual                                 2.452e-01 4.952e-01
Number of obs: 37, groups:  Genotype_Zone_1:Plant_Zone_1, 10; Genotype_Zone_2:Plant_Zone_2, 10

Fixed effects:
            Estimate Std. Error      df t value Pr(>|t|)
(Intercept)   2.0332     0.0814 36.0000   24.98   <2e-16 ***
---
Signif. codes:  0 '***' 0.001 '**' 0.01 '*' 0.05 '.' 0.1 ' ' 1
```

Model residuals

Statistic	Value
Sample skewness	1.233
Sample excess kurtosis	1.235
Passed Shapiro Wilk normality test	No (p-value = 0.00169 < 0.05)

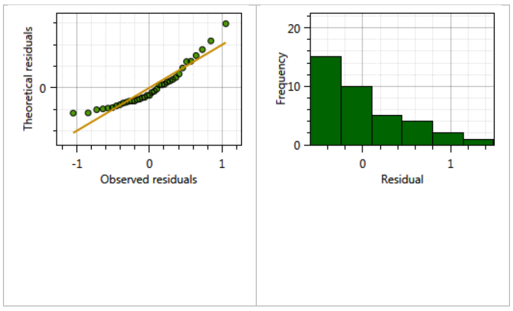

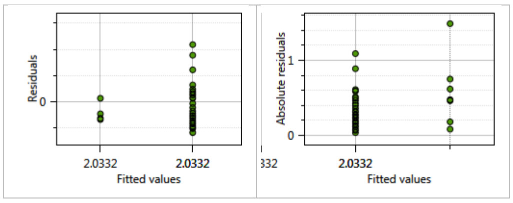

Data points with high residuals

Trial	Arena
Trial 3	12

Analysis average halting duration (H5 - Zone 1)

**Analysis model**	Linear mixed model fit by REML: Average_halting_duration_H5_Zone_1 ~ 1 + (1|Genotype_Zone_1:Plant_Zone_1) + (1|Genotype_Zone_2:Plant_Zone_2)
**Transformation**	Natural logarithm

Confidence intervals average halting duration (H5 - Zone 1)

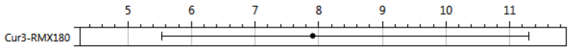

Confidence intervals average halting duration (H5 - Zone 1)

Genotype Zone 1	Genotype Zone 2	Mean	Lower 95% CL	Upper 95% CL	Group
Cur3	RMX180	7.904	5.527	11.3	a

Model summary

```
Linear mixed model fit by REML. t-tests use Satterthwaite's method ['lmerModLmerTest']
Formula: Average_halting_duration_H5_Zone_1 ~ 1 + (1 | Genotype_Zone_1:Plant_Zone_1) +      (1 |
Genotype_Zone_2:Plant_Zone_2)
   Data: data

REML criterion at convergence: 87.1

Scaled residuals:
    Min      1Q  Median      3Q     Max
-3.3539 -0.3992  0.0251  0.5901  1.6529

Random effects:
 Groups                       Name        Variance Std.Dev.
 Genotype_Zone_1:Plant_Zone_1 (Intercept) 0.0000   0.0000
 Genotype_Zone_2:Plant_Zone_2 (Intercept) 0.0000   0.0000
 Residual                                 0.9514   0.9754
Number of obs: 31, groups:  Genotype_Zone_1:Plant_Zone_1, 10; Genotype_Zone_2:Plant_Zone_2, 10

Fixed effects:
            Estimate Std. Error      df t value Pr(>|t|)
(Intercept)   2.0674     0.1752 30.0000    11.8 8.46e-13 ***
---
Signif. codes:  0 '***' 0.001 '**' 0.01 '*' 0.05 '.' 0.1 ' ' 1
```

Model residuals

Statistic	Value
Sample skewness	-1.168
Sample excess kurtosis	3.199
Passed Shapiro Wilk normality test	No (p-value = 0.02326 < 0.05)

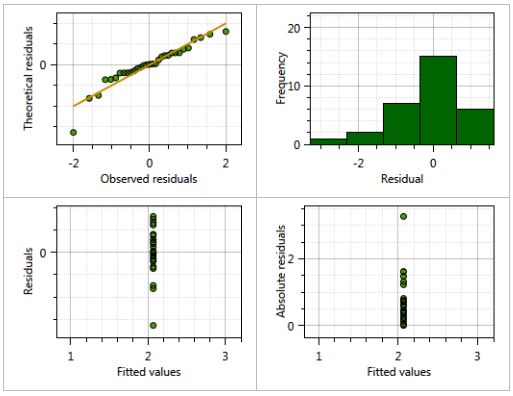

Analysis average halting duration (H5 - Zone 2)

**Analysis model**	Linear mixed model fit by REML: Average_halting_duration_H5_Zone_2 ~ 1 + (1|Genotype_Zone_1:Plant_Zone_1) + (1|Genotype_Zone_2:Plant_Zone_2)
**Transformation**	Natural logarithm

Confidence intervals average halting duration (H5 - Zone 2)

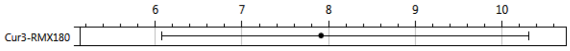

Confidence intervals average halting duration (H5 - Zone 2)

Genotype Zone 1	Genotype Zone 2	Mean	Lower 95% CL	Upper 95% CL	Group
Cur3	RMX180	7.912	6.07	10.31	a

Model summary

```
Linear mixed model fit by REML. t-tests use Satterthwaite's method ['lmerModLmerTest']
Formula: Average_halting_duration_H5_Zone_2 ~ 1 + (1 | Genotype_Zone_1:Plant_Zone_1) +      (1 |
Genotype_Zone_2:Plant_Zone_2)
   Data: data

REML criterion at convergence: 67.4

Scaled residuals:
    Min      1Q  Median      3Q     Max
-1.5492 -0.7561 -0.2024  0.6358  2.3580

Random effects:
 Groups                       Name        Variance Std.Dev.
 Genotype_Zone_1:Plant_Zone_1 (Intercept) 0.02478  0.1574
 Genotype_Zone_2:Plant_Zone_2 (Intercept) 0.02724  0.1651
 Residual                                 0.27123  0.5208
Number of obs: 39, groups:  Genotype_Zone_1:Plant_Zone_1, 10; Genotype_Zone_2:Plant_Zone_2, 10

Fixed effects:
            Estimate Std. Error     df t value Pr(>|t|)
(Intercept)   2.0683     0.1104 6.5128   18.74 6.55e-07 ***
---
Signif. codes:  0 '***' 0.001 '**' 0.01 '*' 0.05 '.' 0.1 ' ' 1
```

Model residuals

Statistic	Value
Sample skewness	0.6097
Sample excess kurtosis	-0.2208
Passed Shapiro Wilk normality test	Yes (p-value = 0.162 > 0.05)

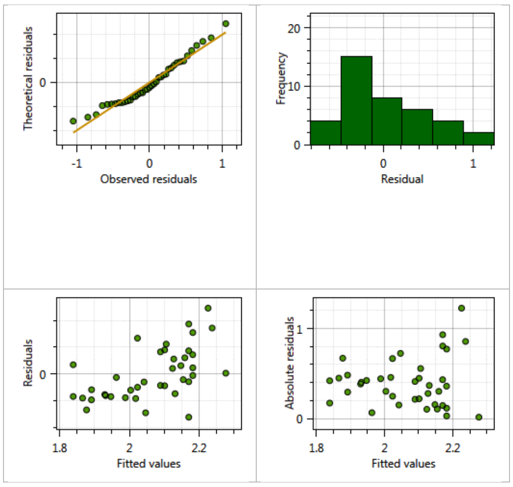

Analysis average halting duration (H6 - Zone 1)

**Analysis model**	Linear mixed model fit by REML: Average_halting_duration_H6_Zone_1 ~ 1 + (1|Genotype_Zone_1:Plant_Zone_1) + (1|Genotype_Zone_2:Plant_Zone_2)
**Transformation**	Natural logarithm

Confidence intervals average halting duration (H6 - Zone 1)

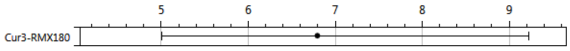

Confidence intervals average halting duration (H6 - Zone 1)

Genotype Zone 1	Genotype Zone 2	Mean	Lower 95% CL	Upper 95% CL	Group
Cur3	RMX180	6.794	5.002	9.228	a

Model summary

```
Linear mixed model fit by REML. t-tests use Satterthwaite's method ['lmerModLmerTest']
Formula: Average_halting_duration_H6_Zone_1 ~ 1 + (1 | Genotype_Zone_1:Plant_Zone_1) +      (1 |
Genotype_Zone_2:Plant_Zone_2)
   Data: data

REML criterion at convergence: 47.7

Scaled residuals:
    Min      1Q  Median      3Q     Max
-1.5281 -0.5942 -0.2682  0.7055  1.9203

Random effects:
 Groups                       Name        Variance Std.Dev.
 Genotype_Zone_1:Plant_Zone_1 (Intercept) 0.00000  0.0000
 Genotype_Zone_2:Plant_Zone_2 (Intercept) 0.07038  0.2653
 Residual                                 0.27041  0.5200
Number of obs: 27, groups:  Genotype_Zone_1:Plant_Zone_1, 10; Genotype_Zone_2:Plant_Zone_2, 10

Fixed effects:
            Estimate Std. Error     df t value Pr(>|t|)
(Intercept)   1.9160     0.1336 8.2849   14.34 3.87e-07 ***
---
Signif. codes:  0 '***' 0.001 '**' 0.01 '*' 0.05 '.' 0.1 ' ' 1
```

Model residuals

Statistic	Value
Sample skewness	0.6113
Sample excess kurtosis	-0.5554
Passed Shapiro Wilk normality test	Yes (p-value = 0.06944 > 0.05)

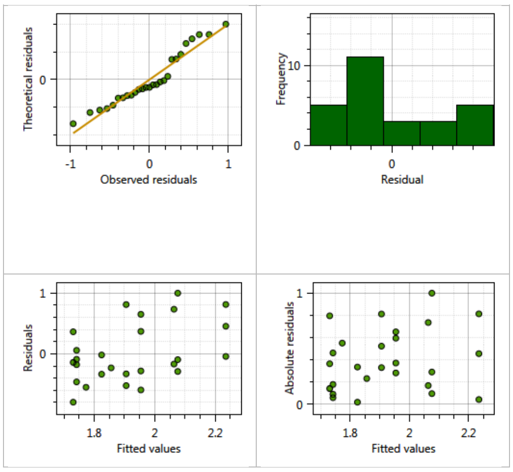

Analysis average halting duration (H6 - Zone 2)

**Analysis model**	Linear mixed model fit by REML: Average_halting_duration_H6_Zone_2 ~ 1 + (1|Genotype_Zone_1:Plant_Zone_1) + (1|Genotype_Zone_2:Plant_Zone_2)
**Transformation**	Natural logarithm

Confidence intervals average halting duration (H6 - Zone 2)

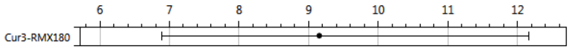

Confidence intervals average halting duration (H6 - Zone 2)

Genotype Zone 1	Genotype Zone 2	Mean	Lower 95% CL	Upper 95% CL	Group
Cur3	RMX180	9.151	6.884	12.16	a

Model summary

```
Linear mixed model fit by REML. t-tests use Satterthwaite's method ['lmerModLmerTest']
Formula: Average_halting_duration_H6_Zone_2 ~ 1 + (1 | Genotype_Zone_1:Plant_Zone_1) +      (1 |
Genotype_Zone_2:Plant_Zone_2)
   Data: data

REML criterion at convergence: 69.9

Scaled residuals:
    Min      1Q  Median      3Q     Max
-1.7740 -0.6720 -0.1559  0.6015  2.7034

Random effects:
 Groups                       Name        Variance Std.Dev.
 Genotype_Zone_1:Plant_Zone_1 (Intercept) 0.002518 0.05018
 Genotype_Zone_2:Plant_Zone_2 (Intercept) 0.043334 0.20817
 Residual                                 0.314569 0.56086
Number of obs: 38, groups:  Genotype_Zone_1:Plant_Zone_1, 10; Genotype_Zone_2:Plant_Zone_2, 10

Fixed effects:
            Estimate Std. Error     df t value Pr(>|t|)
(Intercept)   2.2139     0.1136 5.4627   19.49 2.93e-06 ***
---
Signif. codes:  0 '***' 0.001 '**' 0.01 '*' 0.05 '.' 0.1 ' ' 1
```

Model residuals

Statistic	Value
Sample skewness	0.6789
Sample excess kurtosis	0.5566
Passed Shapiro Wilk normality test	Yes (p-value = 0.2945 > 0.05)

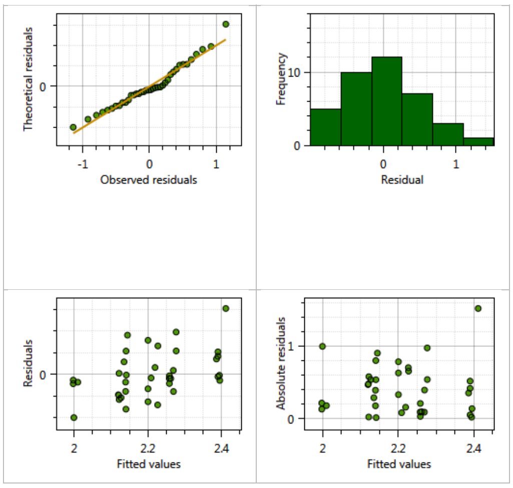

Analysis average halting duration (H7 - Zone 1)

**Analysis model**	Linear mixed model fit by REML: Average_halting_duration_H7_Zone_1 ~ 1 + (1|Genotype_Zone_1:Plant_Zone_1) + (1|Genotype_Zone_2:Plant_Zone_2)
**Transformation**	Natural logarithm

Confidence intervals average halting duration (H7 - Zone 1)

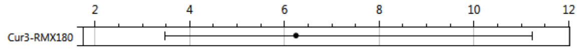

Confidence intervals average halting duration (H7 - Zone 1)

Genotype Zone 1	Genotype Zone 2	Mean	Lower 95% CL	Upper 95% CL	Group
Cur3	RMX180	6.243	3.468	11.24	a

Model summary

```
Linear mixed model fit by REML. t-tests use Satterthwaite's method ['lmerModLmerTest']
Formula: Average_halting_duration_H7_Zone_1 ~ 1 + (1 | Genotype_Zone_1:Plant_Zone_1) +      (1 |
Genotype_Zone_2:Plant_Zone_2)
   Data: data

REML criterion at convergence: 75.6

Scaled residuals:
     Min       1Q   Median       3Q      Max
-2.55025 -0.37595  0.06307  0.41382  1.96696

Random effects:
 Groups                       Name        Variance Std.Dev.
 Genotype_Zone_2:Plant_Zone_2 (Intercept) 0.2337   0.4834
 Genotype_Zone_1:Plant_Zone_1 (Intercept) 0.1517   0.3895
 Residual                                 0.6678   0.8172
Number of obs: 27, groups:  Genotype_Zone_2:Plant_Zone_2, 10; Genotype_Zone_1:Plant_Zone_1, 9

Fixed effects:
            Estimate Std. Error     df t value Pr(>|t|)
(Intercept)   1.8314     0.2622 9.5852   6.984 4.69e-05 ***
---
Signif. codes:  0 '***' 0.001 '**' 0.01 '*' 0.05 '.' 0.1 ' ' 1
```

Model residuals

Statistic	Value
Sample skewness	-0.5238
Sample excess kurtosis	2.302
Passed Shapiro Wilk normality test	Yes (p-value = 0.2957 > 0.05)

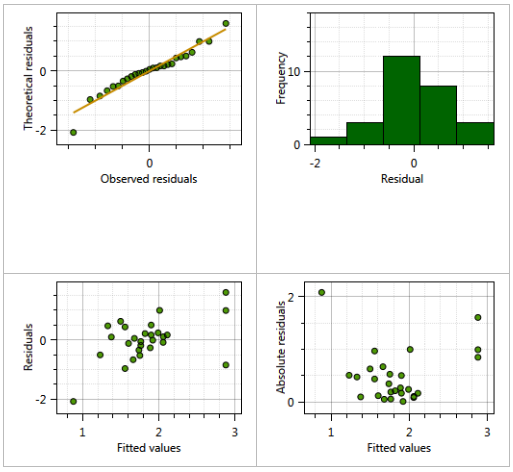

Analysis average halting duration (H7 - Zone 2)

**Analysis model**	Linear mixed model fit by REML: Average_halting_duration_H7_Zone_2 ~ 1 + (1|Genotype_Zone_1:Plant_Zone_1) + (1|Genotype_Zone_2:Plant_Zone_2)
**Transformation**	Natural logarithm

Confidence intervals average halting duration (H7 - Zone 2)

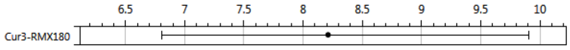

Confidence intervals average halting duration (H7 - Zone 2)

Genotype Zone 1	Genotype Zone 2	Mean	Lower 95% CL	Upper 95% CL	Group
Cur3	RMX180	8.21	6.803	9.908	a

Model summary

```
Linear mixed model fit by REML. t-tests use Satterthwaite's method ['lmerModLmerTest']
Formula: Average_halting_duration_H7_Zone_2 ~ 1 + (1 | Genotype_Zone_1:Plant_Zone_1) +      (1 |
Genotype_Zone_2:Plant_Zone_2)
   Data: data

REML criterion at convergence: 70.1

Scaled residuals: 
     Min       1Q   Median       3Q      Max 
-1.58238 -0.79765 -0.02972  0.65364  2.66451 

Random effects:
 Groups                       Name        Variance  Std.Dev. 
 Genotype_Zone_1:Plant_Zone_1 (Intercept) 0.000e+00 0.000e+00
 Genotype_Zone_2:Plant_Zone_2 (Intercept) 5.189e-17 7.204e-09
 Residual                                 3.362e-01 5.798e-01
Number of obs: 39, groups:  Genotype_Zone_1:Plant_Zone_1, 10; Genotype_Zone_2:Plant_Zone_2, 10

Fixed effects:
            Estimate Std. Error       df t value Pr(>|t|)    
(Intercept)  2.10538    0.09285 38.00000   22.68   <2e-16 ***
---
Signif. codes:  0 '***' 0.001 '**' 0.01 '*' 0.05 '.' 0.1 ' ' 1
```

Model residuals

Statistic	Value
Sample skewness	0.4867
Sample excess kurtosis	-0.04724
Passed Shapiro Wilk normality test	Yes (p-value = 0.411 > 0.05)

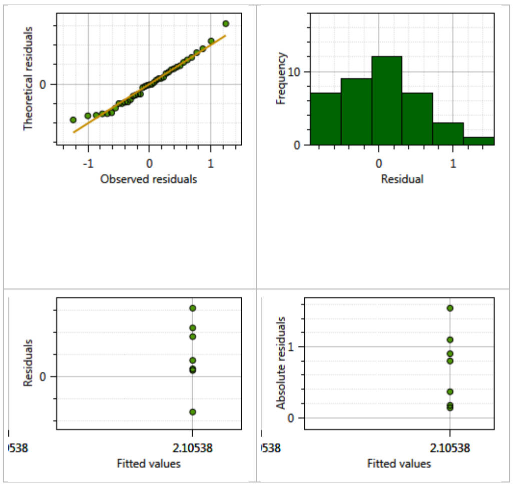

Analysis average halting duration H0 (diff. Zone 1 - Zone 2)

**Analysis model**	Generalized linear mixed model with dispersion factor, formula=cbind(Average_halting_duration_H0_Zone_1,Average_halting_duration_H0_Zone_2) ~ 1 + (1|Genotype_Zone_1:Plant_Zone_1) + (1|Genotype_Zone_2:Plant_Zone_2)
**Transformation**	Logit

Confidence intervals average halting duration H0 (diff. Zone 1 - Zone 2)

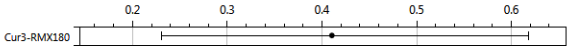

Confidence intervals average halting duration H0 (diff. Zone 1 - Zone 2)

Genotype Zone 1	Genotype Zone 2	Mean	Lower 95% CL	Upper 95% CL	Group
Cur3	RMX180	0.4104	0.2301	0.6184	a

Model summary

```
Linear mixed model fit by REML. t-tests use Satterthwaite's method ['lmerModLmerTest']
Formula: ziFormula
   Data: data
```

```
Weights: wi

REML criterion at convergence: 117

Scaled residuals:
    Min      1Q  Median      3Q     Max
-1.6038 -0.4878  0.1438  0.6131  1.6180

Random effects:
 Groups                        Name        Variance Std.Dev.
 Genotype_Zone_1:Plant_Zone_1 (Intercept) 0.5205   0.7214
 Genotype_Zone_2:Plant_Zone_2 (Intercept) 0.7290   0.8538
 Residual                                 3.6078   1.8994
Number of obs: 33, groups:  Genotype_Zone_1:Plant_Zone_1, 10; Genotype_Zone_2:Plant_Zone_2, 10

Fixed effects:
            Estimate Std. Error      df t value Pr(>|t|)
(Intercept)  -0.3623     0.3966 15.0184  -0.913    0.375

Dispersion: 1.899
```

Model residuals

Statistic	Value
Sample skewness	-0.05106
Sample excess kurtosis	-0.6438
Passed Shapiro Wilk normality test	Yes (p-value = 0.8327 > 0.05)

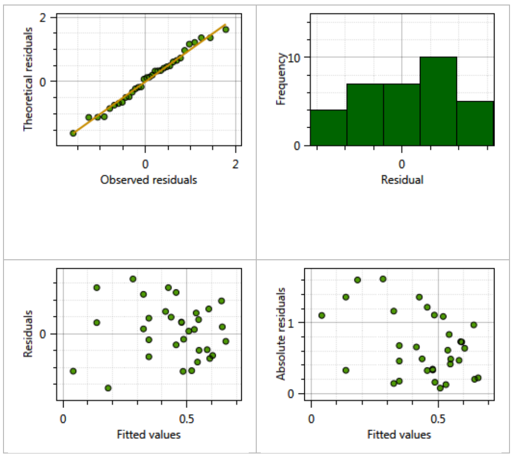

Analysis average halting duration H1 (diff. Zone 1 - Zone 2)

**Analysis model**	Generalized linear mixed model with dispersion factor, formula=cbind(Average_halting_duration_H1_Zone_1,Average_halting_duration_H1_Zone_2) ~ 1 + (1|Genotype_Zone_1:Plant_Zone_1) + (1|Genotype_Zone_2:Plant_Zone_2)
**Transformation**	Logit

Confidence intervals average halting duration H1 (diff. Zone 1 - Zone 2)

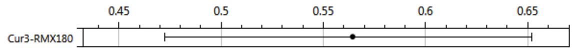

Confidence intervals average halting duration H1 (diff. Zone 1 - Zone 2)

Genotype Zone 1	Genotype Zone 2	Mean	Lower 95% CL	Upper 95% CL	Group
Cur3	RMX180	0.5643	0.4722	0.6522	a

Model summary

```
Linear mixed model fit by REML. t-tests use Satterthwaite's method ['lmerModLmerTest']
Formula: ziFormula
   Data: data
Weights: wi

REML criterion at convergence: 90.6

Scaled residuals:
    Min      1Q  Median      3Q     Max
-2.3289 -0.5574 -0.1687  0.2065  2.8032

Random effects:
 Groups                        Name        Variance Std.Dev.
 Genotype_Zone_1:Plant_Zone_1 (Intercept) 0.09854  0.3139
 Genotype_Zone_2:Plant_Zone_2 (Intercept) 0.00000  0.0000
 Residual                                 2.28479  1.5116
Number of obs: 35, groups:  Genotype_Zone_1:Plant_Zone_1, 10; Genotype_Zone_2:Plant_Zone_2, 10

Fixed effects:
            Estimate Std. Error      df t value Pr(>|t|)
(Intercept)   0.2586     0.1680 10.9647   1.539    0.152

Dispersion: 1.512
```

Model residuals

Statistic	Value
Sample skewness	0.7441
Sample excess kurtosis	2.376
Passed Shapiro Wilk normality test	No (p-value = 0.03246 < 0.05)

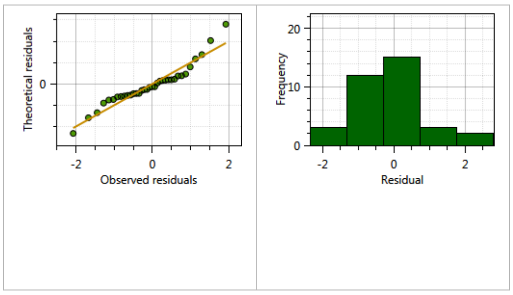

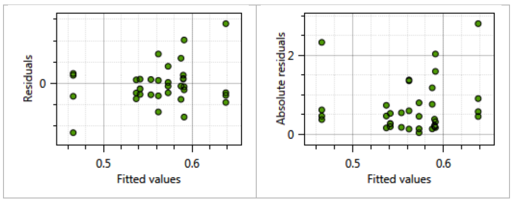

Analysis average halting duration H2 (diff. Zone 1 - Zone 2)

**Analysis model**	Generalized linear mixed model with dispersion factor, formula=cbind(Average_halting_duration_H2_Zone_1,Average_halting_duration_H2_Zone_2) ~ 1 + (1|Genotype_Zone_1:Plant_Zone_1) + (1|Genotype_Zone_2:Plant_Zone_2)
**Transformation**	Logit

Confidence intervals average halting duration H2 (diff. Zone 1 - Zone 2)

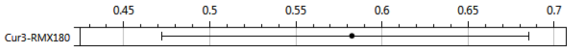

Confidence intervals average halting duration H2 (diff. Zone 1 - Zone 2)

Genotype Zone 1	Genotype Zone 2	Mean	Lower 95% CL	Upper 95% CL	Group
Cur3	RMX180	0.5825	0.4719	0.6854	a

Model summary

```
Linear mixed model fit by REML. t-tests use Satterthwaite's method ['lmerModLmerTest']
Formula: ziFormula
   Data: data
Weights: wi

REML criterion at convergence: 92.3

Scaled residuals:
    Min      1Q  Median      3Q     Max
-1.3563 -0.6502 -0.2767  0.1186  2.4838

Random effects:
 Groups                      Name        Variance Std.Dev.
 Genotype_Zone_1:Plant_Zone_1 (Intercept) 0.2282   0.4777
 Genotype_Zone_2:Plant_Zone_2 (Intercept) 0.0000   0.0000
 Residual                                2.1641   1.4711
Number of obs: 35, groups:  Genotype_Zone_1:Plant_Zone_1, 10; Genotype_Zone_2:Plant_Zone_2, 10

Fixed effects:
            Estimate Std. Error     df t value Pr(>|t|)
(Intercept)   0.3330     0.1987 9.5356   1.676    0.126

Dispersion: 1.471
```

Model residuals

Statistic	Value
Sample skewness	1.284
Sample excess kurtosis	1.56
Passed Shapiro Wilk normality test	No (p-value = 0.002221 < 0.05)

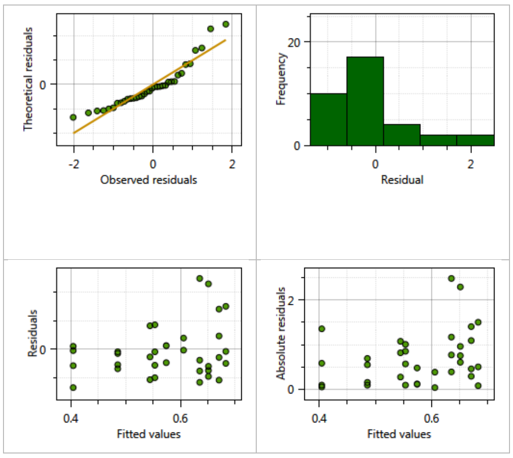

Analysis average halting duration H3 (diff. Zone 1 - Zone 2)

**Analysis model**	Generalized linear mixed model with dispersion factor, formula=cbind(Average_halting_duration_H3_Zone_1,Average_halting_duration_H3_Zone_2) ~ 1 + (1|Genotype_Zone_1:Plant_Zone_1) + (1|Genotype_Zone_2:Plant_Zone_2)
**Transformation**	Logit

Confidence intervals average halting duration H3 (diff. Zone 1 - Zone 2)

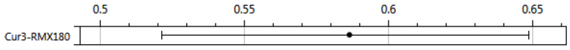

Confidence intervals average halting duration H3 (diff. Zone 1 - Zone 2)

Genotype Zone 1	Genotype Zone 2	Mean	Lower 95% CL	Upper 95% CL	Group
Cur3	RMX180	0.5864	0.5212	0.6488	a

Model summary

```
Linear mixed model fit by REML. t-tests use Satterthwaite's method ['lmerModLmerTest']
Formula: ziFormula
   Data: data
Weights: wi

REML criterion at convergence: 71.3

Scaled residuals:
     Min       1Q   Median       3Q      Max
-2.17619 -0.60581 -0.00441  0.64222  1.40181

Random effects:
 Groups                      Name        Variance Std.Dev.
 Genotype_Zone_1:Plant_Zone_1 (Intercept) 0.006669 0.08167
 Genotype_Zone_2:Plant_Zone_2 (Intercept) 0.000000 0.00000
 Residual                                1.721786 1.31217
Number of obs: 33, groups:  Genotype_Zone_1:Plant_Zone_1, 10; Genotype_Zone_2:Plant_Zone_2, 10

Fixed effects:
            Estimate Std. Error      df t value Pr(>|t|)
(Intercept)   0.3492     0.1195 10.5588   2.922   0.0145 *
---
```

```
Signif. codes:  0 '***' 0.001 '**' 0.01 '*' 0.05 '.' 0.1 ' ' 1

Dispersion: 1.312
```

Model residuals

Statistic	Value
Sample skewness	-0.3546
Sample excess kurtosis	-0.4697
Passed Shapiro Wilk normality test	Yes (p-value = 0.194 > 0.05)

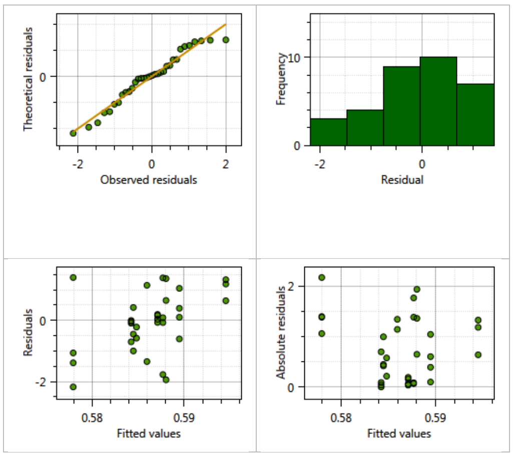

Analysis average halting duration H4 (diff. Zone 1 - Zone 2)

**Analysis model**	Generalized linear mixed model with dispersion factor, formula=cbind(Average_halting_duration_H4_Zone_1,Average_halting_duration_H4_Zone_2) ~ 1 + (1|Genotype_Zone_1:Plant_Zone_1) + (1|Genotype_Zone_2:Plant_Zone_2)
**Transformation**	Logit

Confidence intervals average halting duration H4 (diff. Zone 1 - Zone 2)

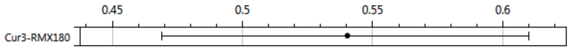

Confidence intervals average halting duration H4 (diff. Zone 1 - Zone 2)

Genotype Zone 1	Genotype Zone 2	Mean	Lower 95% CL	Upper 95% CL	Group
Cur3	RMX180	0.5403	0.4688	0.6101	a

Model summary

```
Linear mixed model fit by REML. t-tests use Satterthwaite's method ['lmerModLmerTest']
Formula: ziFormula
   Data: data
Weights: wi

REML criterion at convergence: 48.8

Scaled residuals: 
    Min      1Q  Median      3Q     Max 
-1.6572 -0.5782  0.1341  0.5940  1.8245 

Random effects:
 Groups                      Name        Variance Std.Dev.
 Genotype_Zone_1:Plant_Zone_1 (Intercept) 0.004435 0.06659 
 Genotype_Zone_2:Plant_Zone_2 (Intercept) 0.045816 0.21405 
 Residual                                1.173738 1.08339 
Number of obs: 27, groups:  Genotype_Zone_1:Plant_Zone_1, 10; Genotype_Zone_2:Plant_Zone_2, 10

Fixed effects:
            Estimate Std. Error     df t value Pr(>|t|)
(Intercept)   0.1615     0.1244 8.0931   1.298     0.23

Dispersion: 1.083
```

Model residuals

Statistic	Value
Sample skewness	-0.2447
Sample excess kurtosis	-0.6918
Passed Shapiro Wilk normality test	Yes (p-value = 0.4638 > 0.05)

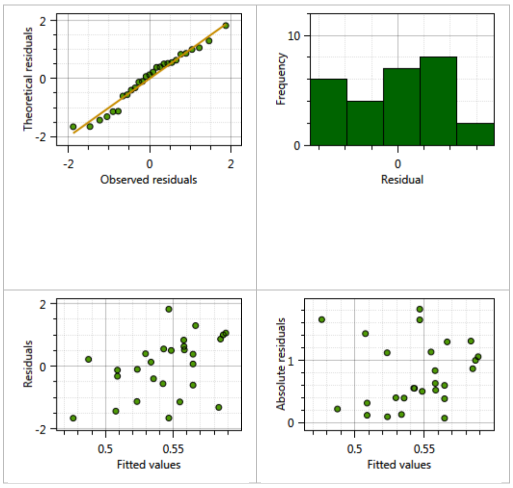

Analysis average halting duration H5 (diff. Zone 1 - Zone 2)

**Analysis model**	Generalized linear mixed model with dispersion factor, formula=cbind(Average_halting_duration_H5_Zone_1,Average_halting_duration_H5_Zone_2) ~ 1 + (1|Genotype_Zone_1:Plant_Zone_1) + (1|Genotype_Zone_2:Plant_Zone_2)
**Transformation**	Logit

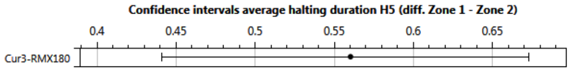

Confidence intervals average halting duration H5 (diff. Zone 1 - Zone 2)

Genotype Zone 1	Genotype Zone 2	Mean	Lower 95% CL	Upper 95% CL	Group
Cur3	RMX180	0.5602	0.4407	0.6731	a

Model summary

```
Linear mixed model fit by REML. t-tests use Satterthwaite's method ['lmerModLmerTest']
Formula: ziFormula
   Data: data
Weights: wi

REML criterion at convergence: 85.2

Scaled residuals:
    Min      1Q  Median      3Q     Max
-2.0323 -0.7033 -0.2510  0.4618  1.7943

Random effects:
 Groups                       Name        Variance Std.Dev.
 Genotype_Zone_1:Plant_Zone_1 (Intercept) 0.2335   0.4832
 Genotype_Zone_2:Plant_Zone_2 (Intercept) 0.0000   0.0000
 Residual                                 2.9094   1.7057
Number of obs: 31, groups:  Genotype_Zone_1:Plant_Zone_1, 10; Genotype_Zone_2:Plant_Zone_2, 10

Fixed effects:
            Estimate Std. Error     df t value Pr(>|t|)
(Intercept)   0.2421     0.2124 9.0069    1.14    0.284

Dispersion: 1.706
```

Model residuals

Statistic	Value
Sample skewness	0.2972
Sample excess kurtosis	-0.2266
Passed Shapiro Wilk normality test	Yes (p-value = 0.7477 > 0.05)

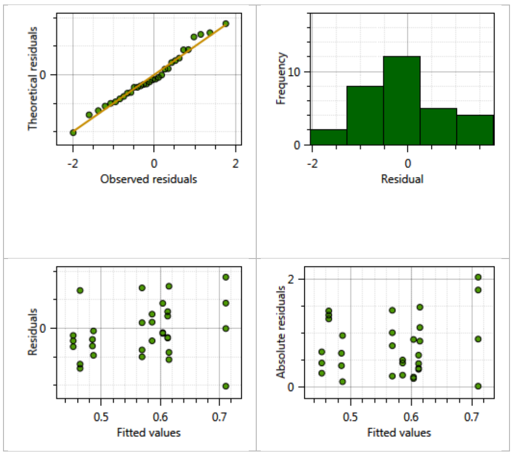

Analysis average halting duration H6 (diff. Zone 1 - Zone 2)

**Analysis model**	Generalized linear mixed model with dispersion factor, formula=cbind(Average_halting_duration_H6_Zone_1,Average_halting_duration_H6_Zone_2) ~ 1 + (1|Genotype_Zone_1:Plant_Zone_1) + (1|Genotype_Zone_2:Plant_Zone_2)
**Transformation**	Logit

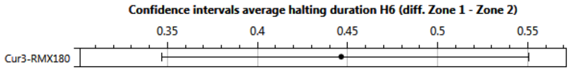

Confidence intervals average halting duration H6 (diff. Zone 1 - Zone 2)

Genotype Zone 1	Genotype Zone 2	Mean	Lower 95% CL	Upper 95% CL	Group
Cur3	RMX180	0.4465	0.3467	0.5507	a

Model summary

```
Linear mixed model fit by REML. t-tests use Satterthwaite's method ['lmerModLmerTest']
Formula: ziFormula
   Data: data
Weights: wi

REML criterion at convergence: 65.4

Scaled residuals:
    Min      1Q  Median      3Q     Max
-1.8190 -0.5598 -0.2856  0.4750  2.1531

Random effects:
 Groups                       Name        Variance Std.Dev.
 Genotype_Zone_1:Plant_Zone_1 (Intercept) 0.1018   0.3191
 Genotype_Zone_2:Plant_Zone_2 (Intercept) 0.0000   0.0000
 Residual                                 2.4347   1.5603
Number of obs: 26, groups:  Genotype_Zone_1:Plant_Zone_1, 10; Genotype_Zone_2:Plant_Zone_2, 10

Fixed effects:
            Estimate Std. Error     df t value Pr(>|t|)
(Intercept)   -0.215      0.181  7.874  -1.188    0.269

Dispersion: 1.56
```

Model residuals

Statistic	Value
Sample skewness	0.6024
Sample excess kurtosis	0.1224
Passed Shapiro Wilk normality test	Yes (p-value = 0.3142 > 0.05)

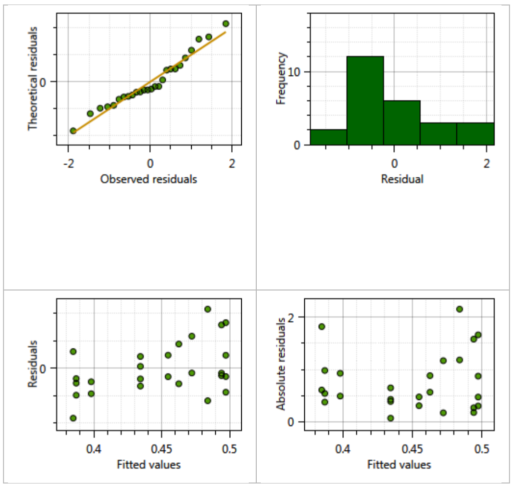

Analysis average halting duration H7 (diff. Zone 1 - Zone 2)

**Analysis model**	Generalized linear mixed model with dispersion factor, formula=cbind(Average_halting_duration_H7_Zone_1,Average_halting_duration_H7_Zone_2) ~ 1 + (1|Genotype_Zone_1:Plant_Zone_1) + (1|Genotype_Zone_2:Plant_Zone_2)
**Transformation**	Logit

Confidence intervals average halting duration H7 (diff. Zone 1 - Zone 2)

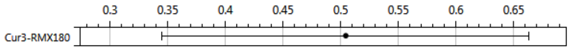

Confidence intervals average halting duration H7 (diff. Zone 1 - Zone 2)

Genotype Zone 1	Genotype Zone 2	Mean	Lower 95% CL	Upper 95% CL	Group
Cur3	RMX180	0.5043	0.3444	0.6633	a

Model summary

```
Linear mixed model fit by REML. t-tests use Satterthwaite's method ['lmerModLmerTest']
Formula: ziFormula
   Data: data
Weights: wi

REML criterion at convergence: 83.5

Scaled residuals:
     Min       1Q   Median       3Q      Max
-2.76783 -0.67564 -0.01331  0.40722  2.16157

Random effects:
 Groups                      Name        Variance  Std.Dev.
 Genotype_Zone_2:Plant_Zone_2 (Intercept) 1.085e-15 3.293e-08
 Genotype_Zone_1:Plant_Zone_1 (Intercept) 4.827e-01 6.947e-01
 Residual                                3.497e+00 1.870e+00
Number of obs: 27, groups:  Genotype_Zone_2:Plant_Zone_2, 10; Genotype_Zone_1:Plant_Zone_1, 9

Fixed effects:
            Estimate Std. Error       df t value Pr(>|t|)
(Intercept)  0.01709    0.29732 10.19447   0.057    0.955

Dispersion: 1.87
```

Model residuals

Statistic	Value
Sample skewness	-0.5453
Sample excess kurtosis	2.84
Passed Shapiro Wilk normality test	Yes (p-value = 0.05562 > 0.05)

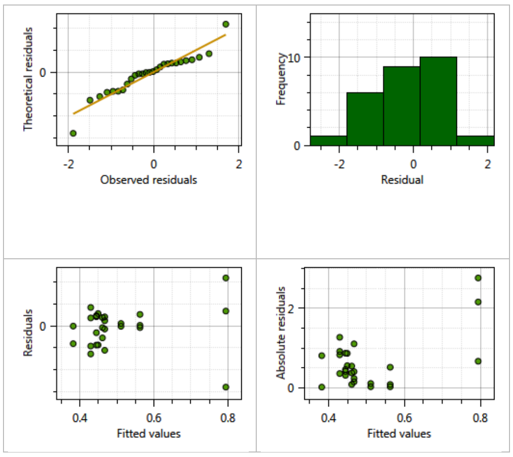

Average halting duration per zone per short/medium/long

Selected zones	Zone 1, Zone 2
Event duration categories	duration < 2, 2 <= duration < 10, duration >= 10
Data transformation	Natural logarithm
Analysis	Zone difference analysis

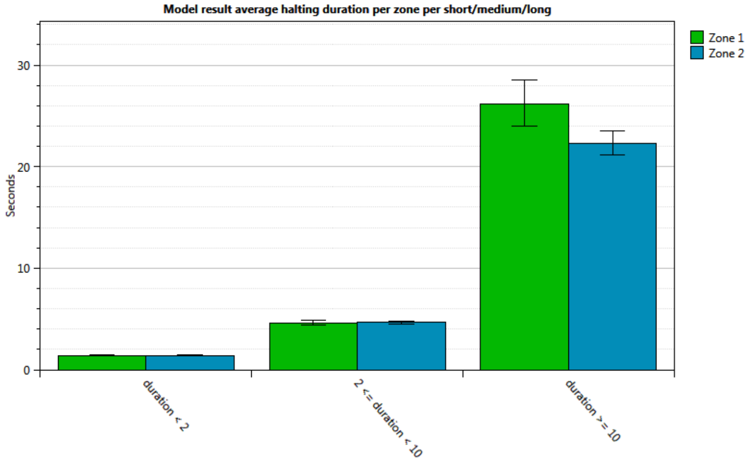

Results difference tests Zone 1 - Zone 2: p values and 95% confidence intervals of the difference on the transformed scale for each statistic.

Behaviour statistic	Cur3-RMX180	Remark
Average halting duration duration < 2 (diff. Zone 1 - Zone 2)	p=0.894<br>[-0.034, 0.0298]	CR
Average halting duration 2 <= duration < 10 (diff. Zone 1 - Zone 2)	p=0.9<br>[-0.0435, 0.0488]	CR
Average halting duration duration >= 10 (diff. Zone 1 - Zone 2)	p=0.000809***<br>[0.0753, 0.264]	

CR = Check residuals

The model predictions and 95% confidence intervals for each statistic.

Statistic	Cur3-RMX180	Remark
Average halting duration (duration < 2 - Zone 1)	1.4<br>[1.36, 1.44]	CR
Average halting duration (2 <= duration < 10 - Zone 1)	4.63<br>[4.41, 4.85]	CR
Average halting duration (duration >= 10 - Zone 1)	26.2<br>[24, 28.6]	
Average halting duration (duration < 2 - Zone 2)	1.41<br>[1.37, 1.45]	
Average halting duration (2 <= duration < 10 - Zone 2)	4.64<br>[4.54, 4.74]	CR
Average halting duration (duration >= 10 - Zone 2)	22.3<br>[21.2, 23.6]	CR

CR = Check residuals

Data summary

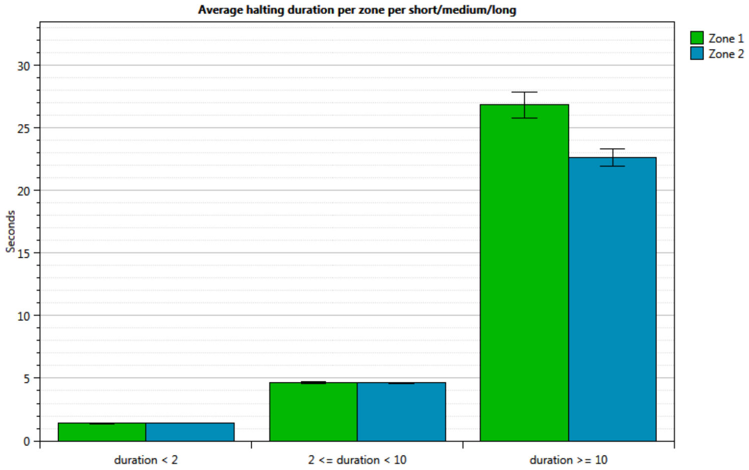

Genotype Zone 1	Genotype Zone 2	Genotype Zone 3	Mean duration < 2 - Zone 1	StdErr duration < 2 - Zone 1	Mean 2 <= duration < 10 - Zone 1	StdErr 2 <= duration < 10 - Zone 1	Mean duration >= 10 - Zone 1	StdErr duration >= 10 - Zone 1	Mean duration < 2 - Zone 2	StdErr duration < 2 - Zone 2	Mean 2 <= duration < 10 - Zone 2	StdErr 2 <= duration < 10 - Zone 2	Mean duration >= 10 - Zone 2	StdErr duration >= 10 - Zone 2
Cur3	RMX180	Neutral	1.4	0.02	4.64	0.07	26.84	1.03	1.41	0.01	4.65	0.04	22.65	0.67

Analysis average halting duration (duration < 2 - Zone 1)

**Analysis model**	Linear mixed model fit by REML: Average_halting_duration_duration_2_Zone_1 ~ 1 + (1|Genotype_Zone_1:Plant_Zone_1) + (1|Genotype_Zone_2:Plant_Zone_2)
**Transformation**	Natural logarithm

Confidence intervals average halting duration (duration < 2 - Zone 1)

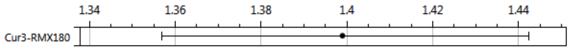

Confidence intervals average halting duration (duration < 2 - Zone 1)

Genotype Zone 1	Genotype Zone 2	Mean	Lower 95% CL	Upper 95% CL	Group
Cur3	RMX180	1.399	1.357	1.442	a

Model summary

```
Linear mixed model fit by REML. t-tests use Satterthwaite's method ['lmerModLmerTest']
Formula: Average_halting_duration_duration_2_Zone_1 ~ 1 + (1 | Genotype_Zone_1:Plant_Zone_1) +      (1 |
Genotype_Zone_2:Plant_Zone_2)
   Data: data

REML criterion at convergence: -66.2

Scaled residuals:
    Min      1Q  Median      3Q     Max
-2.3252 -0.3880  0.2390  0.6133  1.6646

Random effects:
 Groups                       Name        Variance  Std.Dev.
 Genotype_Zone_1:Plant_Zone_1 (Intercept) 1.738e-17 4.169e-09
 Genotype_Zone_2:Plant_Zone_2 (Intercept) 0.000e+00 0.000e+00
 Residual                                 8.432e-03 9.182e-02
Number of obs: 37, groups:  Genotype_Zone_1:Plant_Zone_1, 10; Genotype_Zone_2:Plant_Zone_2, 10
```

```
Fixed effects:
            Estimate Std. Error      df t value Pr(>|t|)
(Intercept)   0.3357     0.0151 36.0000   22.24   <2e-16 ***
---
Signif. codes:  0 '***' 0.001 '**' 0.01 '*' 0.05 '.' 0.1 ' ' 1
```

Model residuals

Statistic	Value
Sample skewness	-0.6699
Sample excess kurtosis	-0.1355
Passed Shapiro Wilk normality test	No (p-value = 0.04946 < 0.05)

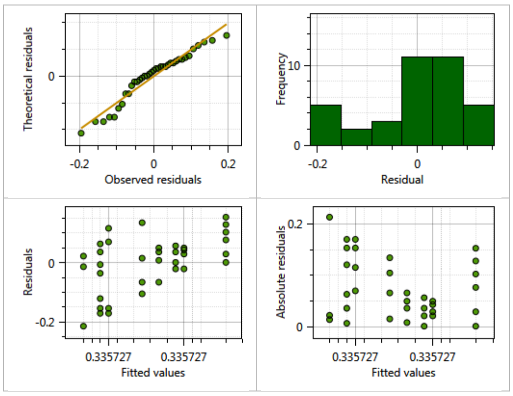

Analysis average halting duration (2 <= duration < 10 - Zone 1)

**Analysis model**	Linear mixed model fit by REML: Average_halting_duration_2_duration_10_Zone_1 ~ 1 + (1|Genotype_Zone_1:Plant_Zone_1) + (1|Genotype_Zone_2:Plant_Zone_2)
**Transformation**	Natural logarithm

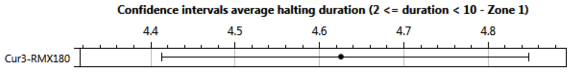

Confidence intervals average halting duration (2 <= duration < 10 - Zone 1)

Genotype Zone 1	Genotype Zone 2	Mean	Lower 95% CL	Upper 95% CL	Group
Cur3	RMX180	4.625	4.413	4.848	a

Model summary

```
Linear mixed model fit by REML. t-tests use Satterthwaite's method ['lmerModLmerTest']
Formula: Average_halting_duration_2_duration_10_Zone_1 ~ 1 + (1 | Genotype_Zone_1:Plant_Zone_1) +      (1
| Genotype_Zone_2:Plant_Zone_2)
   Data: data

REML criterion at convergence: -65

Scaled residuals:
    Min      1Q  Median      3Q     Max
-4.0009 -0.2823  0.1156  0.3411  1.9189

Random effects:
 Groups                       Name        Variance  Std.Dev.
 Genotype_Zone_1:Plant_Zone_1 (Intercept) 0.0015244 0.03904
 Genotype_Zone_2:Plant_Zone_2 (Intercept) 0.0007447 0.02729
 Residual                                 0.0074323 0.08621
Number of obs: 38, groups:  Genotype_Zone_1:Plant_Zone_1, 10; Genotype_Zone_2:Plant_Zone_2, 10

Fixed effects:
            Estimate Std. Error      df t value Pr(>|t|)
(Intercept)  1.53155    0.02064 8.52040   74.21 2.81e-13 ***
---
Signif. codes:  0 '***' 0.001 '**' 0.01 '*' 0.05 '.' 0.1 ' ' 1
```

Model residuals

Statistic	Value
Sample skewness	-2.13
Sample excess kurtosis	9.427
Passed Shapiro Wilk normality test	No (p-value = 2.572E-05 < 0.05)

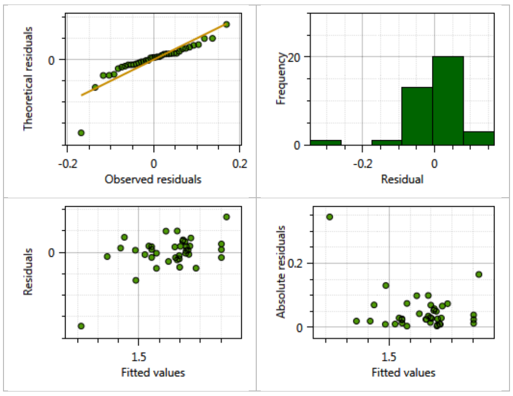

Analysis average halting duration (duration >= 10 - Zone 1)

**Analysis model**	Linear mixed model fit by REML: Average_halting_duration_duration_10_Zone_1 ~ 1 + (1|Genotype_Zone_1:Plant_Zone_1) + (1|Genotype_Zone_2:Plant_Zone_2)
**Transformation**	Natural logarithm

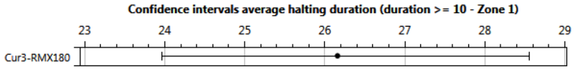

Confidence intervals average halting duration (duration >= 10 - Zone 1)

Genotype Zone 1	Genotype Zone 2	Mean	Lower 95% CL	Upper 95% CL	Group
Cur3	RMX180	26.16	23.96	28.56	a

Model summary

```
Linear mixed model fit by REML. t-tests use Satterthwaite's method ['lmerModLmerTest']
Formula: Average_halting_duration_duration_10_Zone_1 ~ 1 + (1 | Genotype_Zone_1:Plant_Zone_1) +      (1 |
Genotype_Zone_2:Plant_Zone_2)
   Data: data

REML criterion at convergence: -2.3

Scaled residuals: 
    Min      1Q  Median      3Q     Max 
-1.5163 -0.6875 -0.0740  0.5234  2.2895 

Random effects:
 Groups                       Name        Variance Std.Dev.
 Genotype_Zone_1:Plant_Zone_1 (Intercept) 0.002997 0.05474 
 Genotype_Zone_2:Plant_Zone_2 (Intercept) 0.000000 0.00000 
 Residual                                 0.047445 0.21782 
Number of obs: 39, groups:  Genotype_Zone_1:Plant_Zone_1, 10; Genotype_Zone_2:Plant_Zone_2, 10

Fixed effects:
            Estimate Std. Error      df t value Pr(>|t|)    
(Intercept)  3.26419    0.03896 9.24035   83.79 1.25e-14 ***
---
Signif. codes:  0 '***' 0.001 '**' 0.01 '*' 0.05 '.' 0.1 ' ' 1
```

Model residuals

Statistic	Value
Sample skewness	0.5589
Sample excess kurtosis	-0.1396
Passed Shapiro Wilk normality test	Yes (p-value = 0.2061 > 0.05)

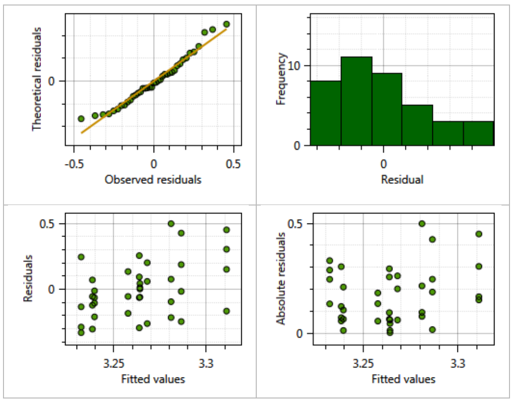

Analysis average halting duration (duration < 2 - Zone 2)

**Analysis model**	Linear mixed model fit by REML: Average_halting_duration_duration_2_Zone_2 ~ 1 + (1|Genotype_Zone_1:Plant_Zone_1) + (1|Genotype_Zone_2:Plant_Zone_2)
**Transformation**	Natural logarithm

Confidence intervals average halting duration (duration < 2 - Zone 2)

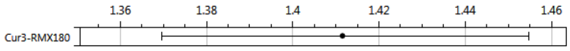

Confidence intervals average halting duration (duration < 2 - Zone 2)

Genotype Zone 1	Genotype Zone 2	Mean	Lower 95% CL	Upper 95% CL	Group
Cur3	RMX180	1.412	1.37	1.455	a

Model summary

```
Linear mixed model fit by REML. t-tests use Satterthwaite's method ['lmerModLmerTest']
Formula: Average_halting_duration_duration_2_Zone_2 ~ 1 + (1 | Genotype_Zone_1:Plant_Zone_1) +      (1 |
Genotype_Zone_2:Plant_Zone_2)
   Data: data

REML criterion at convergence: -95.5

Scaled residuals: 
     Min       1Q   Median       3Q      Max 
-2.24566 -0.74076  0.02459  0.81630  1.66152 

Random effects:
 Groups                       Name        Variance  Std.Dev.
 Genotype_Zone_1:Plant_Zone_1 (Intercept) 0.0004311 0.02076 
 Genotype_Zone_2:Plant_Zone_2 (Intercept) 0.0003798 0.01949 
 Residual                                 0.0036621 0.06051 
Number of obs: 39, groups:  Genotype_Zone_1:Plant_Zone_1, 10; Genotype_Zone_2:Plant_Zone_2, 10

Fixed effects:
            Estimate Std. Error      df t value Pr(>|t|)    
(Intercept)  0.34467    0.01324 8.53269   26.03 1.98e-09 ***
---
Signif. codes:  0 '***' 0.001 '**' 0.01 '*' 0.05 '.' 0.1 ' ' 1
```

Model residuals

Statistic	Value
Sample skewness	-0.2925
Sample excess kurtosis	-0.7283
Passed Shapiro Wilk normality test	Yes (p-value = 0.2521 > 0.05)

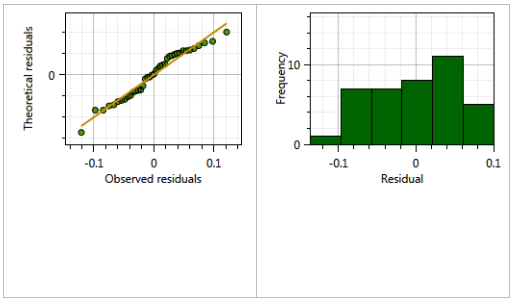

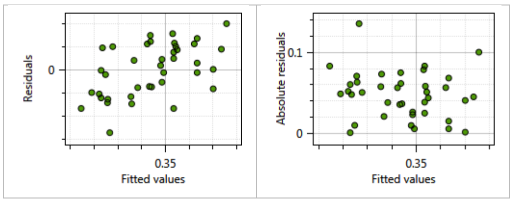

Analysis average halting duration (2 <= duration < 10 - Zone 2)

**Analysis model**	Linear mixed model fit by REML: Average_halting_duration_2_duration_10_Zone_2 ~ 1 + (1|Genotype_Zone_1:Plant_Zone_1) + (1|Genotype_Zone_2:Plant_Zone_2)
**Transformation**	Natural logarithm

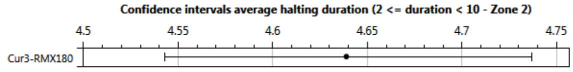

Confidence intervals average halting duration (2 <= duration < 10 - Zone 2)

Genotype Zone 1	Genotype Zone 2	Mean	Lower 95% CL	Upper 95% CL	Group
Cur3	RMX180	4.639	4.543	4.737	a

Model summary

```
Linear mixed model fit by REML. t-tests use Satterthwaite's method ['lmerModLmerTest']
Formula: Average_halting_duration_2_duration_10_Zone_2 ~ 1 + (1 | Genotype_Zone_1:Plant_Zone_1) +      (1
| Genotype_Zone_2:Plant_Zone_2)
   Data: data

REML criterion at convergence: -109.1

Scaled residuals: 
     Min       1Q   Median       3Q      Max 
-1.65901 -0.75232 -0.01197  0.40155  2.34031 

Random effects:
 Groups                       Name        Variance  Std.Dev.
 Genotype_Zone_1:Plant_Zone_1 (Intercept) 0.0000000 0.00000 
 Genotype_Zone_2:Plant_Zone_2 (Intercept) 0.0001094 0.01046 
 Residual                                 0.0029124 0.05397 
Number of obs: 39, groups:  Genotype_Zone_1:Plant_Zone_1, 10; Genotype_Zone_2:Plant_Zone_2, 10

Fixed effects:
            Estimate Std. Error       df t value Pr(>|t|)    
(Intercept) 1.534469   0.009256 8.952201   165.8   <2e-16 ***
---
Signif. codes:  0 '***' 0.001 '**' 0.01 '*' 0.05 '.' 0.1 ' ' 1
```

Model residuals

Statistic	Value
Sample skewness	0.7158
Sample excess kurtosis	0.3747
Passed Shapiro Wilk normality test	No (p-value = 0.02918 < 0.05)

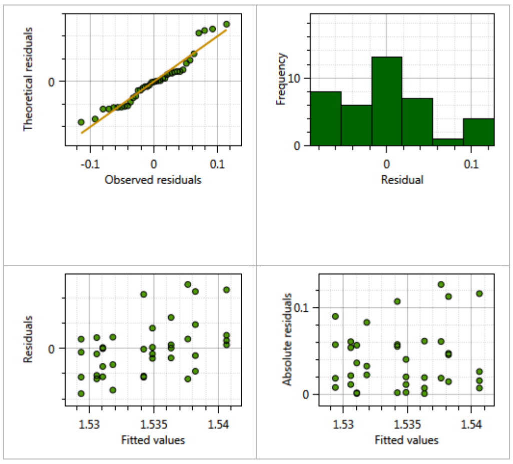

Analysis average halting duration (duration >= 10 - Zone 2)

**Analysis model**	Linear mixed model fit by REML: Average_halting_duration_duration_10_Zone_2 ~ 1 + (1|Genotype_Zone_1:Plant_Zone_1) + (1|Genotype_Zone_2:Plant_Zone_2)
**Transformation**	Natural logarithm

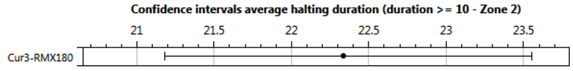

Confidence intervals average halting duration (duration >= 10 - Zone 2)

Genotype Zone 1	Genotype Zone 2	Mean	Lower 95% CL	Upper 95% CL	Group
Cur3	RMX180	22.34	21.18	23.55	a

Model summary

```
Linear mixed model fit by REML. t-tests use Satterthwaite's method ['lmerModLmerTest']
Formula: Average_halting_duration_duration_10_Zone_2 ~ 1 + (1 | Genotype_Zone_1:Plant_Zone_1) +      (1 |
Genotype_Zone_2:Plant_Zone_2)
   Data: data

REML criterion at convergence: -25.9

Scaled residuals: 
    Min      1Q  Median      3Q     Max 
-1.5504 -0.5691 -0.0756  0.3998  3.5633 

Random effects:
 Groups                       Name        Variance  Std.Dev.
 Genotype_Zone_1:Plant_Zone_1 (Intercept) 5.461e-19 7.39e-10
 Genotype_Zone_2:Plant_Zone_2 (Intercept) 0.000e+00 0.00e+00
 Residual                                 2.690e-02 1.64e-01
Number of obs: 39, groups:  Genotype_Zone_1:Plant_Zone_1, 10; Genotype_Zone_2:Plant_Zone_2, 10

Fixed effects:
            Estimate Std. Error       df t value Pr(>|t|)    
(Intercept)  3.10616    0.02626 38.00000   118.3   <2e-16 ***
---
Signif. codes:  0 '***' 0.001 '**' 0.01 '*' 0.05 '.' 0.1 ' ' 1
```

Model residuals

Statistic	Value
Sample skewness	1.254
Sample excess kurtosis	2.946
Passed Shapiro Wilk normality test	No (p-value = 0.009713 < 0.05)

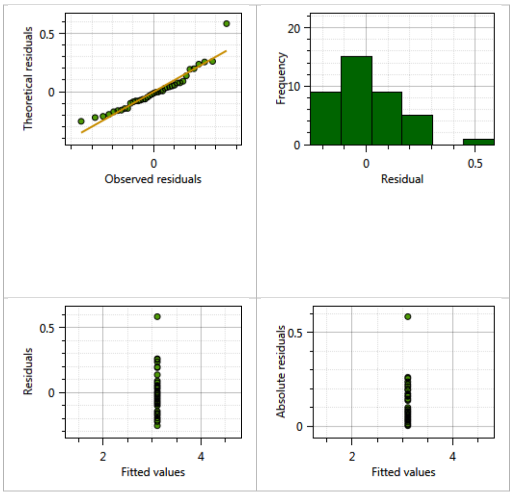

Data points with high residuals

Trial	Arena
Trial 3	12

Analysis average halting duration duration < 2 (diff. Zone 1 - Zone 2)

**Analysis model**	Generalized linear mixed model with dispersion factor, formula=cbind(Average_halting_duration_duration_2_Zone_1,Average_halting_duration_duration_2_Zone_2) ~ 1 + (1|Genotype_Zone_1:Plant_Zone_1) + (1|Genotype_Zone_2:Plant_Zone_2)
**Transformation**	Logit

Confidence intervals average halting duration duration < 2 (diff. Zone 1 - Zone 2)

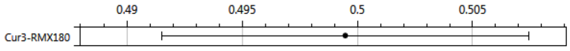

Confidence intervals average halting duration duration < 2 (diff. Zone 1 - Zone 2)

Genotype Zone 1	Genotype Zone 2	Mean	Lower 95% CL	Upper 95% CL	Group
Cur3	RMX180	0.4995	0.4915	0.5075	a

Model summary

```
Linear mixed model fit by REML. t-tests use Satterthwaite's method ['lmerModLmerTest']
Formula: ziFormula
   Data: data
Weights: wi

REML criterion at convergence: -63.1

Scaled residuals:
     Min       1Q   Median       3Q      Max
-2.48503 -0.51011 -0.05089  0.87496  1.29443

Random effects:
 Groups                      Name        Variance Std.Dev.
 Genotype_Zone_1:Plant_Zone_1 (Intercept) 0.000000 0.00000
 Genotype_Zone_2:Plant_Zone_2 (Intercept) 0.000000 0.00000
 Residual                                 0.006443 0.08027
Number of obs: 37, groups:  Genotype_Zone_1:Plant_Zone_1, 10; Genotype_Zone_2:Plant_Zone_2, 10

Fixed effects:
             Estimate Std. Error        df t value Pr(>|t|)
(Intercept) -0.002114   0.015738 36.000000  -0.134    0.894

Dispersion: 0.08027
```

Model residuals

Statistic	Value
Sample skewness	-0.6361
Sample excess kurtosis	-0.1831
Passed Shapiro Wilk normality test	No (p-value = 0.03379 < 0.05)

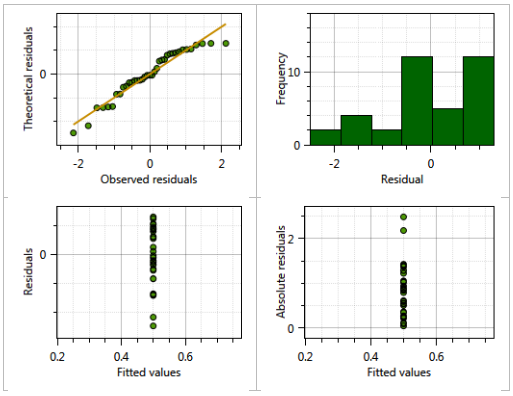

Analysis average halting duration 2 <= duration < 10 (diff. Zone 1 - Zone 2)

**Analysis model**	Generalized linear mixed model with dispersion factor, formula=cbind(Average_halting_duration_2_duration_10_Zone_1,Average_halting_duration_2_duration_10_Zone_2) ~ 1 + (1|Genotype_Zone_1:Plant_Zone_1) + (1|Genotype_Zone_2:Plant_Zone_2)
**Transformation**	Logit

Confidence intervals average halting duration 2 <= duration < 10 (diff. Zone 1 - Zone 2)

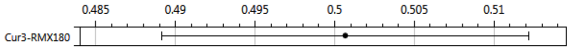

Confidence intervals average halting duration 2 <= duration < 10 (diff. Zone 1 - Zone 2)

Genotype Zone 1	Genotype Zone 2	Mean	Lower 95% CL	Upper 95% CL	Group
Cur3	RMX180	0.5007	0.4891	0.5122	a

Model summary

```
Linear mixed model fit by REML. t-tests use Satterthwaite's method ['lmerModLmerTest']
Formula: ziFormula
   Data: data
Weights: wi

REML criterion at convergence: -52.2

Scaled residuals: 
    Min      1Q  Median      3Q     Max 
-4.2372 -0.4083 -0.0444  0.6203  1.2931 

Random effects:
 Groups                      Name        Variance  Std.Dev.
 Genotype_Zone_1:Plant_Zone_1 (Intercept) 9.325e-04 3.054e-02
 Genotype_Zone_2:Plant_Zone_2 (Intercept) 4.501e-17 6.709e-09
 Residual                                2.820e-02 1.679e-01
Number of obs: 38, groups:  Genotype_Zone_1:Plant_Zone_1, 10; Genotype_Zone_2:Plant_Zone_2, 10

Fixed effects:
            Estimate Std. Error       df t value Pr(>|t|)
(Intercept) 0.002636   0.020382 8.926491   0.129      0.9

Dispersion: 0.1679
```

Model residuals

Statistic	Value
Sample skewness	-2.18
Sample excess kurtosis	8.725
Passed Shapiro Wilk normality test	No (p-value = 4.291E-05 < 0.05)

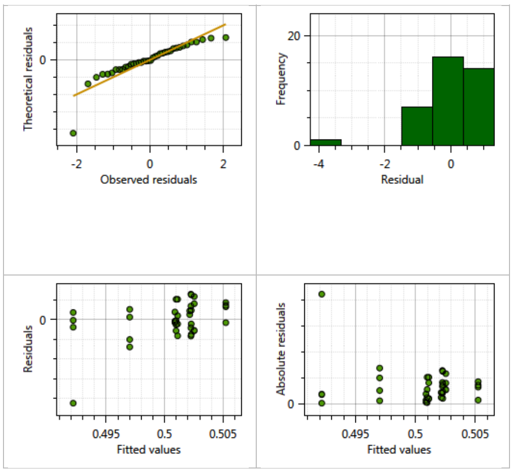

Analysis average halting duration duration >= 10 (diff. Zone 1 - Zone 2)

**Analysis model**	Generalized linear mixed model with dispersion factor, formula=cbind(Average_halting_duration_duration_10_Zone_1,Average_halting_duration_duration_10_Zone_2) ~ 1 + (1|Genotype_Zone_1:Plant_Zone_1) + (1|Genotype_Zone_2:Plant_Zone_2)
**Transformation**	Logit

Confidence intervals average halting duration duration >= 10 (diff. Zone 1 - Zone 2)

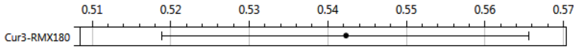

Confidence intervals average halting duration duration >= 10 (diff. Zone 1 - Zone 2)

Genotype Zone 1	Genotype Zone 2	Mean	Lower 95% CL	Upper 95% CL	Group
Cur3	RMX180	0.5423	0.5188	0.5656	a

Model summary

```
Linear mixed model fit by REML. t-tests use Satterthwaite's method ['lmerModLmerTest']
Formula: ziFormula
   Data: data
Weights: wi

REML criterion at convergence: 18.1

Scaled residuals: 
    Min      1Q  Median      3Q     Max 
-2.3386 -0.6071 -0.1946  0.5297  2.3232 

Random effects:
 Groups                      Name        Variance Std.Dev.
 Genotype_Zone_1:Plant_Zone_1 (Intercept) 0.00     0.00    
 Genotype_Zone_2:Plant_Zone_2 (Intercept) 0.00     0.00    
 Residual                                1.04     1.02    
Number of obs: 39, groups:  Genotype_Zone_1:Plant_Zone_1, 10; Genotype_Zone_2:Plant_Zone_2, 10

Fixed effects:
            Estimate Std. Error      df t value Pr(>|t|)    
(Intercept)   0.1696     0.0466 38.0000    3.64 0.000809 ***
---
Signif. codes:  0 '***' 0.001 '**' 0.01 '*' 0.05 '.' 0.1 ' ' 1

Dispersion: 1.02
```

Model residuals

Statistic	Value
Sample skewness	0.1779
Sample excess kurtosis	0.4665
Passed Shapiro Wilk normality test	Yes (p-value = 0.6761 > 0.05)

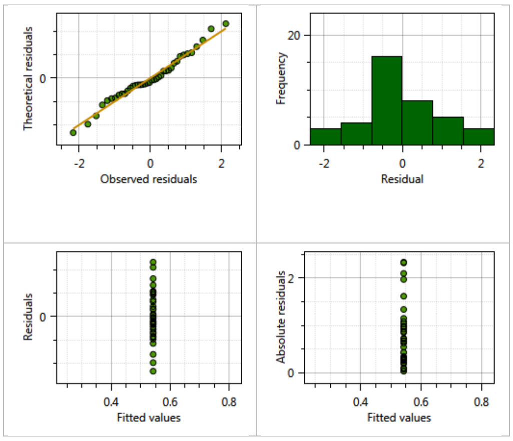

Average movement distance per zone

Selected zones	Zone 1, Zone 2
Data transformation	Natural logarithm
Analysis	Zone difference analysis

Model result average movement distance per zone

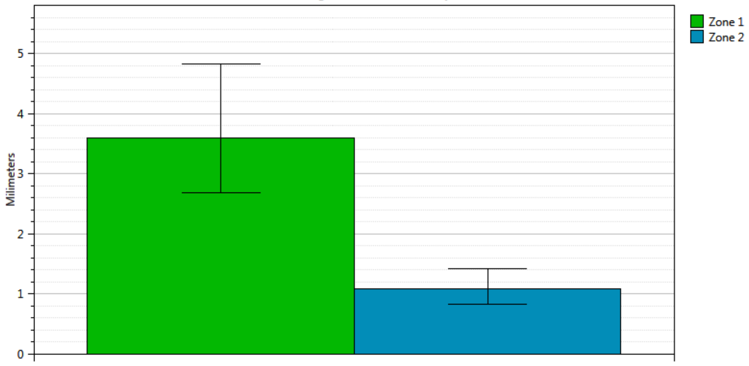

Results difference tests Zone 1 - Zone 2: p values and 95% confidence intervals of the difference on the transformed scale for each statistic.

Behaviour statistic	Cur3-RMX180
Average movement distance (diff. Zone 1 - Zone 2)	p=3.54E-05****<br>[0.711, 1.34]

The model predictions and 95% confidence intervals for each statistic.

Statistic	Cur3-RMX180	Remark
Average movement distance (Zone 1)	3.6<br>[2.68, 4.83]	CR
Average movement distance (Zone 2)	1.08<br>[0.821, 1.42]	CR

CR = Check residuals

Data summary

Average movement distance per zone

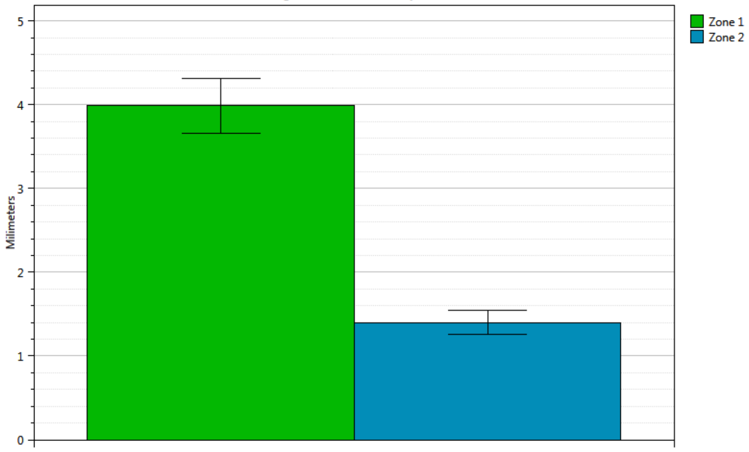

Genotype Zone 1	Genotype Zone 2	Genotype Zone 3	Mean Zone 1	StdErr Zone 1	Mean Zone 2	StdErr Zone 2
Cur3	RMX180	Neutral	3.99	0.33	1.4	0.14

Analysis average movement distance (Zone 1)

**Analysis model**	Linear mixed model fit by REML: Average_movement_distance_Zone_1 ~ 1 + (1|Genotype_Zone_1:Plant_Zone_1) + (1|Genotype_Zone_2:Plant_Zone_2)
**Transformation**	Natural logarithm

Confidence intervals average movement distance (Zone 1)

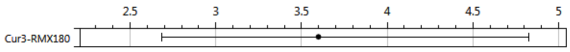

Confidence intervals average movement distance (Zone 1)

Genotype Zone 1	Genotype Zone 2	Mean	Lower 95% CL	Upper 95% CL	Group
Cur3	RMX180	3.598	2.682	4.827	a

Model summary

```
Linear mixed model fit by REML. t-tests use Satterthwaite's method ['lmerModLmerTest']
Formula: Average_movement_distance_Zone_1 ~ 1 + (1 | Genotype_Zone_1:Plant_Zone_1) +      (1 |
Genotype_Zone_2:Plant_Zone_2)
   Data: data

REML criterion at convergence: 54.8

Scaled residuals:
    Min      1Q  Median      3Q     Max
-2.6652 -0.2116  0.1607  0.4877  1.2779

Random effects:
 Groups                       Name        Variance Std.Dev.
 Genotype_Zone_1:Plant_Zone_1 (Intercept) 0.05131  0.2265
 Genotype_Zone_2:Plant_Zone_2 (Intercept) 0.08149  0.2855
 Residual                                 0.15018  0.3875
Number of obs: 38, groups:  Genotype_Zone_1:Plant_Zone_1, 10; Genotype_Zone_2:Plant_Zone_2, 10

Fixed effects:
            Estimate Std. Error     df t value Pr(>|t|)
(Intercept)   1.2805     0.1316 9.8337   9.732 2.31e-06 ***
---
Signif. codes:  0 '***' 0.001 '**' 0.01 '*' 0.05 '.' 0.1 ' ' 1
```

Model residuals

Statistic	Value
Sample skewness	-1.186
Sample excess kurtosis	1.759
Passed Shapiro Wilk normality test	No (p-value = 0.006385 < 0.05)

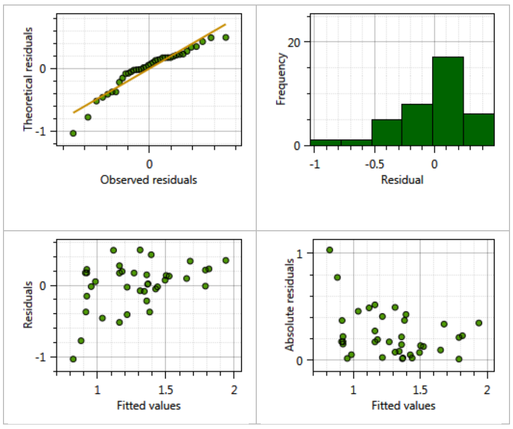

Analysis average movement distance (Zone 2)

**Analysis model**	Linear mixed model fit by REML: Average_movement_distance_Zone_2 ~ 1 + (1|Genotype_Zone_1:Plant_Zone_1) + (1|Genotype_Zone_2:Plant_Zone_2)
**Transformation**	Natural logarithm

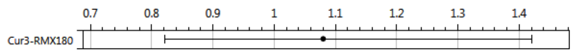

Confidence intervals average movement distance (Zone 2)

Genotype Zone 1	Genotype Zone 2	Mean	Lower 95% CL	Upper 95% CL	Group
Cur3	RMX180	1.08	0.8207	1.421	a

Model summary

```
Linear mixed model fit by REML. t-tests use Satterthwaite's method ['lmerModLmerTest']
Formula: Average_movement_distance_Zone_2 ~ 1 + (1 | Genotype_Zone_1:Plant_Zone_1) +      (1 |
Genotype_Zone_2:Plant_Zone_2)
   Data: data

REML criterion at convergence: 98.9

Scaled residuals:
    Min      1Q  Median      3Q     Max
-3.0737 -0.3693  0.2280  0.5901  1.6095

Random effects:
 Groups                       Name        Variance Std.Dev.
 Genotype_Zone_1:Plant_Zone_1 (Intercept) 0.000    0.0000
 Genotype_Zone_2:Plant_Zone_2 (Intercept) 0.000    0.0000
 Residual                                 0.717    0.8468
Number of obs: 39, groups:  Genotype_Zone_1:Plant_Zone_1, 10; Genotype_Zone_2:Plant_Zone_2, 10

Fixed effects:
            Estimate Std. Error       df t value Pr(>|t|)
(Intercept)  0.07693    0.13559 38.00000   0.567    0.574
```

Model residuals

Statistic	Value
Sample skewness	-1.39
Sample excess kurtosis	2.662
Passed Shapiro Wilk normality test	No (p-value = 0.00129 < 0.05)

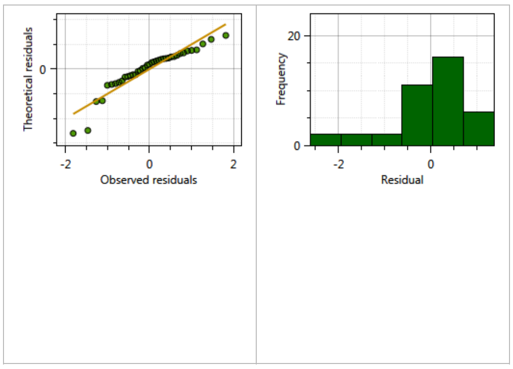

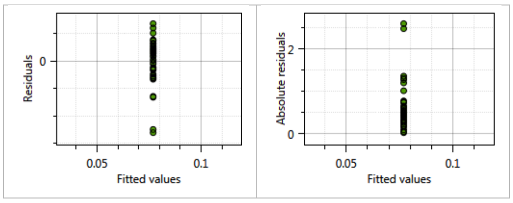

Analysis average movement distance (diff. Zone 1 - Zone 2)

**Analysis model**	Generalized linear mixed model with dispersion factor, formula=cbind(Average_movement_distance_Zone_1,Average_movement_distance_Zone_2) ~ 1 + (1|Genotype_Zone_1:Plant_Zone_1) + (1|Genotype_Zone_2:Plant_Zone_2)
**Transformation**	Logit

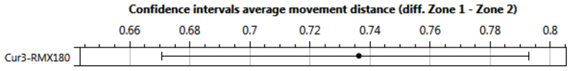

Confidence intervals average movement distance (diff. Zone 1 - Zone 2)

Genotype Zone 1	Genotype Zone 2	Mean	Lower 95% CL	Upper 95% CL	Group
Cur3	RMX180	0.7363	0.6706	0.7929	a

Model summary

```
Linear mixed model fit by REML. t-tests use Satterthwaite's method ['lmerModLmerTest']
Formula: ziFormula
   Data: data
Weights: wi

REML criterion at convergence: 87

Scaled residuals:
     Min       1Q   Median       3Q      Max
-2.51640 -0.62178  0.03957  0.69447  1.72543

Random effects:
 Groups                        Name        Variance Std.Dev.
 Genotype_Zone_1:Plant_Zone_1 (Intercept) 0.00000  0.0000
 Genotype_Zone_2:Plant_Zone_2 (Intercept) 0.09832  0.3136
 Residual                                 0.38573  0.6211
Number of obs: 39, groups:  Genotype_Zone_1:Plant_Zone_1, 10; Genotype_Zone_2:Plant_Zone_2, 10

Fixed effects:
            Estimate Std. Error     df t value Pr(>|t|)
(Intercept)   1.0267     0.1407 9.4449   7.298 3.54e-05 ***
---
Signif. codes:  0 '***' 0.001 '**' 0.01 '*' 0.05 '.' 0.1 ' ' 1

Dispersion: 0.6211
```

Model residuals

Statistic	Value
Sample skewness	-0.4995
Sample excess kurtosis	0.1404
Passed Shapiro Wilk normality test	Yes (p-value = 0.5277 > 0.05)

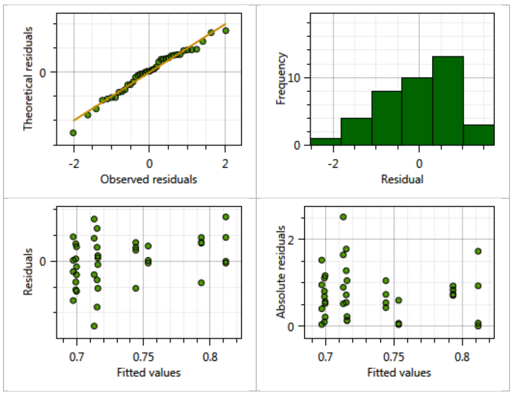

Average movement distance per zone per hour

Selected hours	0, 1, 2, 3, 4, 5, 6, 7
Selected zones	Zone 1, Zone 2
Data transformation	Natural logarithm
Analysis	Zone difference analysis

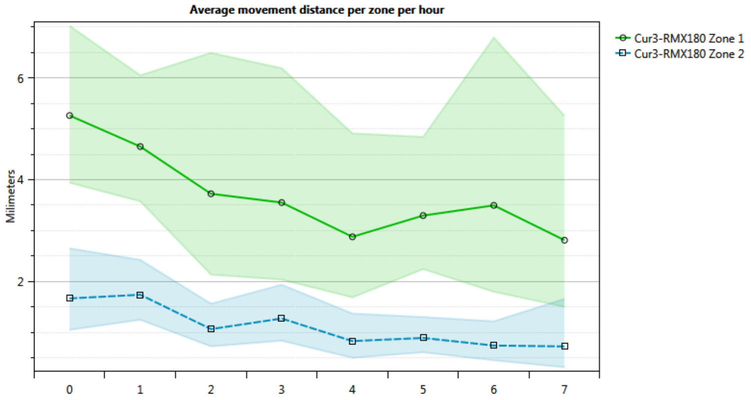

Results difference tests Zone 1 - Zone 2: p values and 95% confidence intervals of the difference on the transformed scale for each statistic.

Behaviour statistic	Cur3-RMX180	Remark
Average movement distance H0 (diff. Zone 1 - Zone 2)	p=0.00403** [0.434, 1.78]	CR

Behaviour statistic	Cur3-RMX180	Remark
Average movement distance H1 (diff. Zone 1 - Zone 2)	p=0.0122*<br>[0.215, 1.35]	CR
Average movement distance H2 (diff. Zone 1 - Zone 2)	p=2.8E-08****<br>[0.805, 1.44]	CR
Average movement distance H3 (diff. Zone 1 - Zone 2)	p=0.0494*<br>[0.00253, 1.37]	
Average movement distance H4 (diff. Zone 1 - Zone 2)	p=0.006**<br>[0.388, 1.72]	
Average movement distance H5 (diff. Zone 1 - Zone 2)	p=0.0165*<br>[0.195, 1.47]	
Average movement distance H6 (diff. Zone 1 - Zone 2)	p=0.000116***<br>[0.625, 1.34]	
Average movement distance H7 (diff. Zone 1 - Zone 2)	p=0.00788**<br>[0.273, 1.19]	

CR = Check residuals

The model predictions and 95% confidence intervals for each statistic.

Statistic	Cur3-RMX180	Remark
Average movement distance (H0 - Zone 1)	5.26<br>[3.94, 7.02]	
Average movement distance (H0 - Zone 2)	1.67<br>[1.05, 2.65]	
Average movement distance (H1 - Zone 1)	4.65<br>[3.58, 6.05]	
Average movement distance (H1 - Zone 2)	1.74<br>[1.25, 2.43]	
Average movement distance (H2 - Zone 1)	3.72<br>[2.14, 6.49]	
Average movement distance (H2 - Zone 2)	1.07<br>[0.727, 1.57]	CR
Average movement distance (H3 - Zone 1)	3.55<br>[2.04, 6.19]	CR
Average movement distance (H3 - Zone 2)	1.28<br>[0.838, 1.94]	
Average movement distance (H4 - Zone 1)	2.88<br>[1.69, 4.91]	
Average movement distance (H4 - Zone 2)	0.832<br>[0.503, 1.37]	
Average movement distance (H5 - Zone 1)	3.3<br>[2.24, 4.84]	
Average movement distance (H5 - Zone 2)	0.893<br>[0.611, 1.31]	
Average movement distance (H6 - Zone 1)	3.5<br>[1.8, 6.79]	
Average movement distance (H6 - Zone 2)	0.744<br>[0.454, 1.22]	
Average movement distance (H7 - Zone 1)	2.81<br>[1.5, 5.26]	CR
Average movement distance (H7 - Zone 2)	0.727<br>[0.318, 1.66]	

CR = Check residuals

Data summary

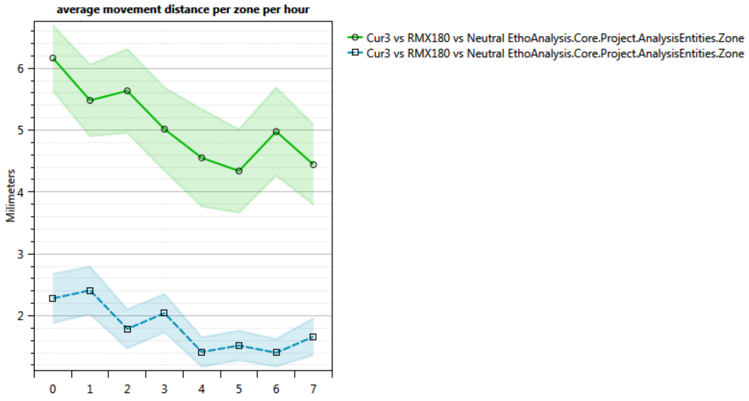

Genotype Zone 1	Genotype Zone 2	Genotype Zone 3	Mean H0 - Zone 1	StdErr H0 - Zone 1	Mean H0 - Zone 2	StdErr H0 - Zone 2	Mean H1 - Zone 1	StdErr H1 - Zone 1	Mean H1 - Zone 2	StdErr H1 - Zone 2	Mean H2 - Zone 1	StdErr H2 - Zone 1	Mean H2 - Zone 2	StdErr H2 - Zone 2	Mean H3 - Zone 1	StdErr H3 - Zone 1	Mean H3 - Zone 2	StdErr H3 - Zone 2	Mean H4 - Zone 1	StdErr H4 - Zone 1	Mean H4 - Zone 2	StdErr H4 - Zone 2	Mean H5 - Zone 1	StdErr H5 - Zone 1	Mean H5 - Zone 2	StdErr H5 - Zone 2	Mean H6 - Zone 1	StdErr H6 - Zone 1	Mean H6 - Zone 2	StdErr H6 - Zone 2
Cur3	RMX180	Neutral	6.17	0.53	2.28	0.4	5.48	0.58	2.41	0.38	5.64	0.68	1.79	0.32	5.02	0.67	2.04	0.31	4.55	0.79	1.42	0.24	4.34	0.67	1.52	0.24	4.98	0.71	1.4	0.22

Analysis average movement distance (H0 - Zone 1)

**Analysis model**	Linear mixed model fit by REML: Average_movement_distance_H0_Zone_1 ~ 1 + (1|Genotype_Zone_1:Plant_Zone_1) + (1|Genotype_Zone_2:Plant_Zone_2)
**Transformation**	Natural logarithm

Confidence intervals average movement distance (H0 - Zone 1)

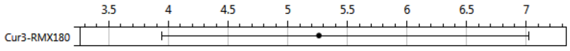

Confidence intervals average movement distance (H0 - Zone 1)

Genotype Zone 1	Genotype Zone 2	Mean	Lower 95% CL	Upper 95% CL	Group
Cur3	RMX180	5.26	3.941	7.022	a

Model summary

```
Linear mixed model fit by REML. t-tests use Satterthwaite's method ['lmerModLmerTest']
Formula: Average_movement_distance_H0_Zone_1 ~ 1 + (1 | Genotype_Zone_1:Plant_Zone_1) +      (1 |
Genotype_Zone_2:Plant_Zone_2)
   Data: data

REML criterion at convergence: 59.1

Scaled residuals:
    Min      1Q  Median      3Q     Max
-2.4422 -0.4958  0.1113  0.4914  1.3682

Random effects:
 Groups                       Name        Variance Std.Dev.
 Genotype_Zone_1:Plant_Zone_1 (Intercept) 0.03725  0.1930
 Genotype_Zone_2:Plant_Zone_2 (Intercept) 0.00000  0.0000
 Residual                                 0.30338  0.5508
Number of obs: 33, groups:  Genotype_Zone_1:Plant_Zone_1, 10; Genotype_Zone_2:Plant_Zone_2, 10

Fixed effects:
            Estimate Std. Error     df t value Pr(>|t|)
(Intercept)   1.6602     0.1149 5.4020   14.45  1.6e-05 ***
---
Signif. codes:  0 '***' 0.001 '**' 0.01 '*' 0.05 '.' 0.1 ' ' 1
```

Model residuals

Statistic	Value
Sample skewness	-0.8225
Sample excess kurtosis	0.5607
Passed Shapiro Wilk normality test	Yes (p-value = 0.07464 > 0.05)

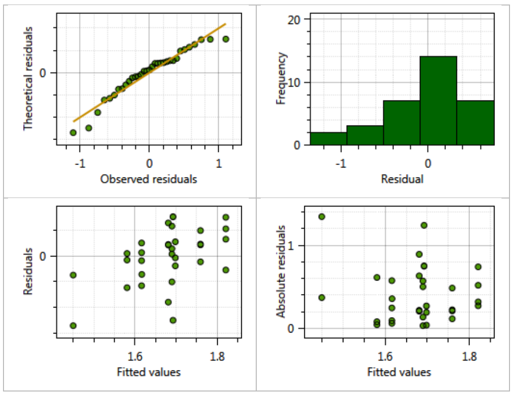

Analysis average movement distance (H0 - Zone 2)

**Analysis model**	Linear mixed model fit by REML: Average_movement_distance_H0_Zone_2 ~ 1 + (1|Genotype_Zone_1:Plant_Zone_1) + (1|Genotype_Zone_2:Plant_Zone_2)
**Transformation**	Natural logarithm

Confidence intervals average movement distance (H0 - Zone 2)

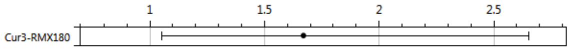

Confidence intervals average movement distance (H0 - Zone 2)

Genotype Zone 1	Genotype Zone 2	Mean	Lower 95% CL	Upper 95% CL	Group
Cur3	RMX180	1.67	1.051	2.654	a

Model summary

```
Linear mixed model fit by REML. t-tests use Satterthwaite's method ['lmerModLmerTest']
Formula: Average_movement_distance_H0_Zone_2 ~ 1 + (1 | Genotype_Zone_1:Plant_Zone_1) +      (1 |
Genotype_Zone_2:Plant_Zone_2)
   Data: data

REML criterion at convergence: 82.8

Scaled residuals: 
     Min       1Q   Median       3Q      Max 
-1.75795 -0.42394  0.00947  0.43522  2.28622 

Random effects:
 Groups                       Name        Variance Std.Dev.
 Genotype_Zone_1:Plant_Zone_1 (Intercept) 0.05512  0.2348  
 Genotype_Zone_2:Plant_Zone_2 (Intercept) 0.22184  0.4710  
 Residual                                 0.38967  0.6242  
Number of obs: 36, groups:  Genotype_Zone_1:Plant_Zone_1, 10; Genotype_Zone_2:Plant_Zone_2, 10

Fixed effects:
            Estimate Std. Error     df t value Pr(>|t|)  
(Intercept)   0.5127     0.1976 7.3037   2.595   0.0344 *
---
Signif. codes:  0 '***' 0.001 '**' 0.01 '*' 0.05 '.' 0.1 ' ' 1
```

Model residuals

Statistic	Value
Sample skewness	0.3837
Sample excess kurtosis	0.7731
Passed Shapiro Wilk normality test	Yes (p-value = 0.4072 > 0.05)

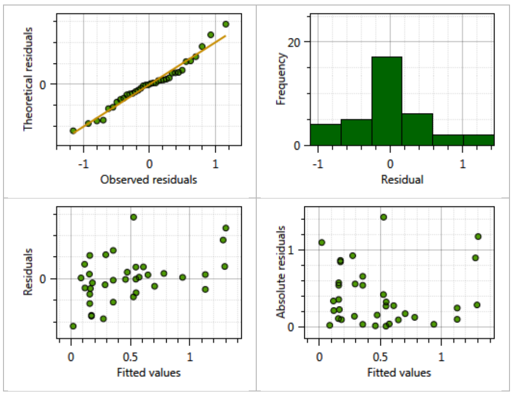

Analysis average movement distance (H1 - Zone 1)

**Analysis model**	Linear mixed model fit by REML: Average_movement_distance_H1_Zone_1 ~ 1 + (1|Genotype_Zone_1:Plant_Zone_1) + (1|Genotype_Zone_2:Plant_Zone_2)
**Transformation**	Natural logarithm

Confidence intervals average movement distance (H1 - Zone 1)

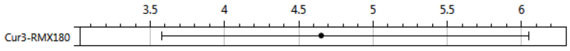

Confidence intervals average movement distance (H1 - Zone 1)

Genotype Zone 1	Genotype Zone 2	Mean	Lower 95% CL	Upper 95% CL	Group
Cur3	RMX180	4.651	3.575	6.051	a

Model summary

```
Linear mixed model fit by REML. t-tests use Satterthwaite's method ['lmerModLmerTest']
Formula: Average_movement_distance_H1_Zone_1 ~ 1 + (1 | Genotype_Zone_1:Plant_Zone_1) +      (1 |
Genotype_Zone_2:Plant_Zone_2)
   Data: data

REML criterion at convergence: 57.1
```

```
Scaled residuals:
    Min      1Q  Median      3Q     Max
-1.7053 -0.7978  0.1293  0.5695  2.1609

Random effects:
 Groups                     Name        Variance Std.Dev.
 Genotype_Zone_1:Plant_Zone_1 (Intercept) 0.00000  0.0000
 Genotype_Zone_2:Plant_Zone_2 (Intercept) 0.07535  0.2745
 Residual                               0.21988  0.4689
Number of obs: 36, groups:  Genotype_Zone_1:Plant_Zone_1, 10; Genotype_Zone_2:Plant_Zone_2, 10

Fixed effects:
            Estimate Std. Error     df t value Pr(>|t|)
(Intercept)   1.5371     0.1171 9.4032   13.13 2.32e-07 ***
---
Signif. codes:  0 '***' 0.001 '**' 0.01 '*' 0.05 '.' 0.1 ' ' 1
```

Model residuals

Statistic	Value
Sample skewness	0.07504
Sample excess kurtosis	-0.4166
Passed Shapiro Wilk normality test	Yes (p-value = 0.7779 > 0.05)

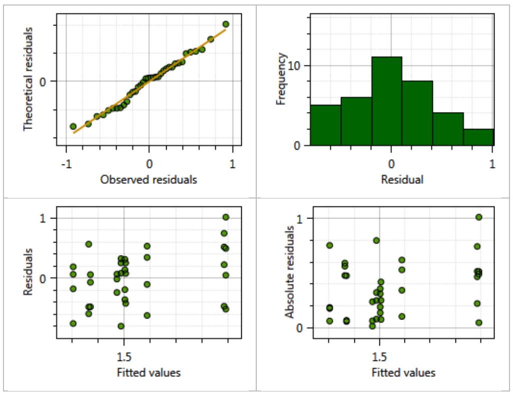

Analysis average movement distance (H1 - Zone 2)

**Analysis model**	Linear mixed model fit by REML: Average_movement_distance_H1_Zone_2 ~ 1 + (1|Genotype_Zone_1:Plant_Zone_1) + (1|Genotype_Zone_2:Plant_Zone_2)
**Transformation**	Natural logarithm

Confidence intervals average movement distance (H1 - Zone 2)

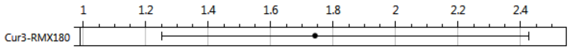

Confidence intervals average movement distance (H1 - Zone 2)

Genotype Zone 1	Genotype Zone 2	Mean	Lower 95% CL	Upper 95% CL	Group
Cur3	RMX180	1.742	1.25	2.427	a

Model summary

```
Linear mixed model fit by REML. t-tests use Satterthwaite's method ['lmerModLmerTest']
Formula: Average_movement_distance_H1_Zone_2 ~ 1 + (1 | Genotype_Zone_1:Plant_Zone_1) +      (1 |
Genotype_Zone_2:Plant_Zone_2)
   Data: data

REML criterion at convergence: 88.7

Scaled residuals:
    Min      1Q  Median      3Q     Max
-1.5653 -0.6755 -0.1552  0.5843  2.2784

Random effects:
 Groups                     Name        Variance Std.Dev.
 Genotype_Zone_1:Plant_Zone_1 (Intercept) 0.00000  0.0000
 Genotype_Zone_2:Plant_Zone_2 (Intercept) 0.04864  0.2206
 Residual                               0.58238  0.7631
Number of obs: 37, groups:  Genotype_Zone_1:Plant_Zone_1, 10; Genotype_Zone_2:Plant_Zone_2, 10

Fixed effects:
            Estimate Std. Error     df t value Pr(>|t|)
(Intercept)   0.5549     0.1437 7.9443    3.86  0.00487 **
---
Signif. codes:  0 '***' 0.001 '**' 0.01 '*' 0.05 '.' 0.1 ' ' 1
```

Model residuals

Statistic	Value
Sample skewness	0.3934
Sample excess kurtosis	-0.1209
Passed Shapiro Wilk normality test	Yes (p-value = 0.4396 > 0.05)

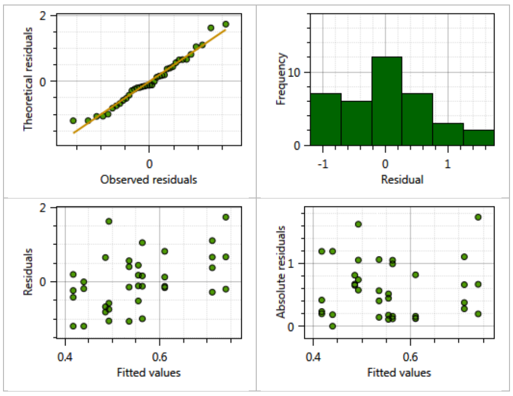

Analysis average movement distance (H2 - Zone 1)

**Analysis model**	Linear mixed model fit by REML: Average_movement_distance_H2_Zone_1 ~ 1 + (1|Genotype_Zone_1:Plant_Zone_1) + (1|Genotype_Zone_2:Plant_Zone_2)
**Transformation**	Natural logarithm

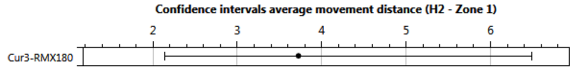

Confidence intervals average movement distance (H2 - Zone 1)

Genotype Zone 1	Genotype Zone 2	Mean	Lower 95% CL	Upper 95% CL	Group
Cur3	RMX180	3.724	2.136	6.492	a

Model summary

```
Linear mixed model fit by REML. t-tests use Satterthwaite's method ['lmerModLmerTest']
Formula: Average_movement_distance_H2_Zone_1 ~ 1 + (1 | Genotype_Zone_1:Plant_Zone_1) +      (1 |
Genotype_Zone_2:Plant_Zone_2)
   Data: data

REML criterion at convergence: 104

Scaled residuals:
    Min      1Q  Median      3Q     Max
-2.2279 -0.7191  0.1991  0.7050  1.4704

Random effects:
 Groups                       Name        Variance Std.Dev.
 Genotype_Zone_1:Plant_Zone_1 (Intercept) 0.01867  0.1366
 Genotype_Zone_2:Plant_Zone_2 (Intercept) 0.36568  0.6047
 Residual                                 0.79004  0.8888
Number of obs: 36, groups:  Genotype_Zone_1:Plant_Zone_1, 10; Genotype_Zone_2:Plant_Zone_2, 10

Fixed effects:
            Estimate Std. Error     df t value Pr(>|t|)
(Intercept)   1.3147     0.2464 9.1682   5.336 0.000442 ***
---
Signif. codes:  0 '***' 0.001 '**' 0.01 '*' 0.05 '.' 0.1 ' ' 1
```

Model residuals

Statistic	Value
Sample skewness	-0.5742
Sample excess kurtosis	-0.2727
Passed Shapiro Wilk normality test	Yes (p-value = 0.2587 > 0.05)

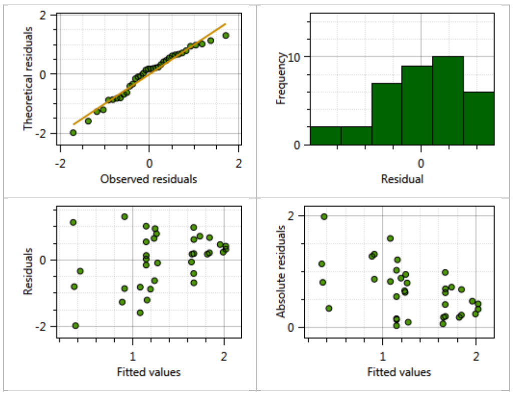

Analysis average movement distance (H2 - Zone 2)

Analysis model	Linear mixed model fit by REML: Average_movement_distance_H2_Zone_2 ~ 1 + (1|Genotype_Zone_1:Plant_Zone_1) + (1|Genotype_Zone_2:Plant_Zone_2)
Transformation	Natural logarithm

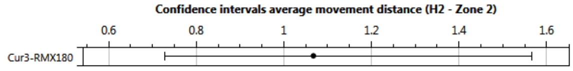

Confidence intervals average movement distance (H2 - Zone 2)

Genotype Zone 1	Genotype Zone 2	Mean	Lower 95% CL	Upper 95% CL	Group
Cur3	RMX180	1.067	0.7272	1.567	a

Model summary

```
Linear mixed model fit by REML. t-tests use Satterthwaite's method ['lmerModLmerTest']
Formula: Average_movement_distance_H2_Zone_2 ~ 1 + (1 | Genotype_Zone_1:Plant_Zone_1) +      (1 |
Genotype_Zone_2:Plant_Zone_2)
   Data: data

REML criterion at convergence: 115.9

Scaled residuals:
    Min      1Q  Median      3Q     Max
-3.4552 -0.5005  0.1089  0.4824  1.7322

Random effects:
 Groups                       Name        Variance  Std.Dev.
 Genotype_Zone_1:Plant_Zone_1 (Intercept) 2.364e-20 1.537e-10
 Genotype_Zone_2:Plant_Zone_2 (Intercept) 0.000e+00 0.000e+00
 Residual                                 1.325e+00 1.151e+00
Number of obs: 37, groups:  Genotype_Zone_1:Plant_Zone_1, 10; Genotype_Zone_2:Plant_Zone_2, 10

Fixed effects:
            Estimate Std. Error       df t value Pr(>|t|)
(Intercept)  0.06529    0.18924 36.00000   0.345    0.732
```

Model residuals

Statistic	Value
Sample skewness	-0.9723
Sample excess kurtosis	2.858
Passed Shapiro Wilk normality test	No (p-value = 0.03027 < 0.05)

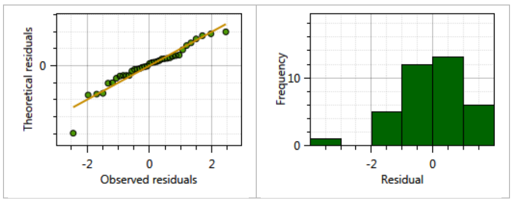

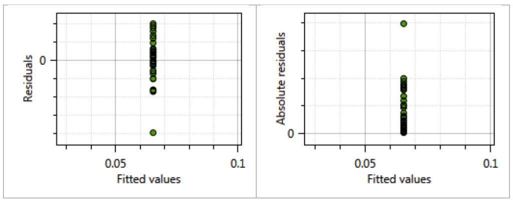

Analysis average movement distance (H3 - Zone 1)

**Analysis model**	Linear mixed model fit by REML: Average_movement_distance_H3_Zone_1 ~ 1 + (1|Genotype_Zone_1:Plant_Zone_1) + (1|Genotype_Zone_2:Plant_Zone_2)
**Transformation**	Natural logarithm

Confidence intervals average movement distance (H3 - Zone 1)

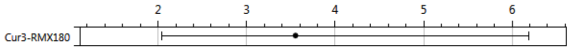

Confidence intervals average movement distance (H3 - Zone 1)

Genotype Zone 1	Genotype Zone 2	Mean	Lower 95% CL	Upper 95% CL	Group
Cur3	RMX180	3.552	2.038	6.191	a

Model summary

```
Linear mixed model fit by REML. t-tests use Satterthwaite's method ['lmerModLmerTest']
Formula: Average_movement_distance_H3_Zone_1 ~ 1 + (1 | Genotype_Zone_1:Plant_Zone_1) +      (1 |
Genotype_Zone_2:Plant_Zone_2)
   Data: data

REML criterion at convergence: 91.9

Scaled residuals:
    Min      1Q  Median      3Q     Max
-1.8539 -0.5646  0.1962  0.7301  1.2822

Random effects:
 Groups                       Name        Variance Std.Dev.
 Genotype_Zone_1:Plant_Zone_1 (Intercept) 0.01863  0.1365
 Genotype_Zone_2:Plant_Zone_2 (Intercept) 0.37739  0.6143
 Residual                                 0.68299  0.8264
Number of obs: 33, groups:  Genotype_Zone_1:Plant_Zone_1, 10; Genotype_Zone_2:Plant_Zone_2, 10

Fixed effects:
            Estimate Std. Error    df t value Pr(>|t|)
(Intercept)    1.268      0.247 9.335   5.133  0.00055 ***
---
Signif. codes:  0 '***' 0.001 '**' 0.01 '*' 0.05 '.' 0.1 ' ' 1
```

Model residuals

Statistic	Value
Sample skewness	-0.5541
Sample excess kurtosis	-0.7968
Passed Shapiro Wilk normality test	No (p-value = 0.04515 < 0.05)

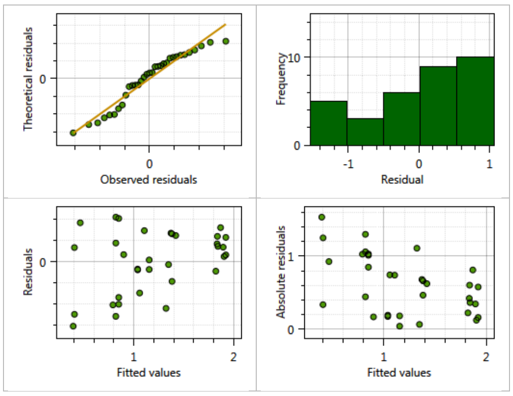

Analysis average movement distance (H3 - Zone 2)

**Analysis model**	Linear mixed model fit by REML: Average_movement_distance_H3_Zone_2 ~ 1 + (1|Genotype_Zone_1:Plant_Zone_1) + (1|Genotype_Zone_2:Plant_Zone_2)
**Transformation**	Natural logarithm

Confidence intervals average movement distance (H3 - Zone 2)

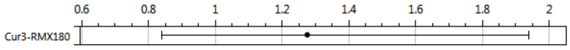

Confidence intervals average movement distance (H3 - Zone 2)

Genotype Zone 1	Genotype Zone 2	Mean	Lower 95% CL	Upper 95% CL	Group
Cur3	RMX180	1.275	0.8385	1.94	a

Model summary

```
Linear mixed model fit by REML. t-tests use Satterthwaite's method ['lmerModLmerTest']
Formula: Average_movement_distance_H3_Zone_2 ~ 1 + (1 | Genotype_Zone_1:Plant_Zone_1) +      (1 |
Genotype_Zone_2:Plant_Zone_2)
   Data: data

REML criterion at convergence: 119.1

Scaled residuals:
    Min      1Q  Median      3Q     Max
-2.5484 -0.3246  0.1167  0.7216  1.5708

Random effects:
 Groups                       Name        Variance  Std.Dev.
 Genotype_Zone_1:Plant_Zone_1 (Intercept) 6.652e-17 8.156e-09
 Genotype_Zone_2:Plant_Zone_2 (Intercept) 3.913e-02 1.978e-01
 Residual                                 1.187e+00 1.090e+00
Number of obs: 39, groups:  Genotype_Zone_1:Plant_Zone_1, 10; Genotype_Zone_2:Plant_Zone_2, 10

Fixed effects:
            Estimate Std. Error     df t value Pr(>|t|)
(Intercept)   0.2431     0.1854 9.0136   1.311    0.222
```

Model residuals

Statistic	Value
Sample skewness	-0.6799

Statistic	Value
Sample excess kurtosis	0.3124
Passed Shapiro Wilk normality test	Yes (p-value = 0.07678 > 0.05)

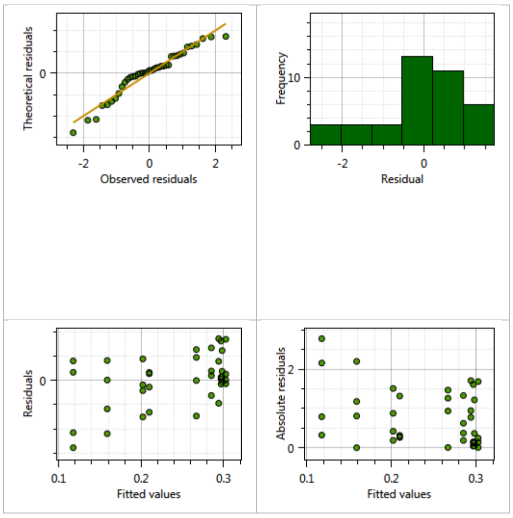

Analysis average movement distance (H4 - Zone 1)

**Analysis model**	Linear mixed model fit by REML: Average_movement_distance_H4_Zone_1 ~ 1 + (1|Genotype_Zone_1:Plant_Zone_1) + (1|Genotype_Zone_2:Plant_Zone_2)
**Transformation**	Natural logarithm

Confidence intervals average movement distance (H4 - Zone 1)

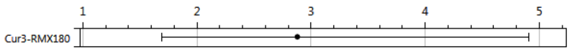

Confidence intervals average movement distance (H4 - Zone 1)

Genotype Zone 1	Genotype Zone 2	Mean	Lower 95% CL	Upper 95% CL	Group
Cur3	RMX180	2.879	1.688	4.911	a

Model summary

```
Linear mixed model fit by REML. t-tests use Satterthwaite's method ['lmerModLmerTest']
Formula: Average_movement_distance_H4_Zone_1 ~ 1 + (1 | Genotype_Zone_1:Plant_Zone_1) +      (1 |
Genotype_Zone_2:Plant_Zone_2)
   Data: data

REML criterion at convergence: 92.3

Scaled residuals:
    Min      1Q  Median      3Q     Max
-2.8078 -0.4285  0.1923  0.5910  1.4764

Random effects:
 Groups                       Name        Variance Std.Dev.
 Genotype_Zone_1:Plant_Zone_1 (Intercept) 0.00000  0.0000
 Genotype_Zone_2:Plant_Zone_2 (Intercept) 0.09144  0.3024
 Residual                                 1.17559  1.0842
Number of obs: 30, groups:  Genotype_Zone_1:Plant_Zone_1, 10; Genotype_Zone_2:Plant_Zone_2, 10

Fixed effects:
            Estimate Std. Error    df t value Pr(>|t|)
(Intercept)    1.058      0.221 6.336   4.786  0.00263 **
---
Signif. codes:  0 '***' 0.001 '**' 0.01 '*' 0.05 '.' 0.1 ' ' 1
```

Model residuals

Statistic	Value
Sample skewness	-0.9359
Sample excess kurtosis	1.256
Passed Shapiro Wilk normality test	Yes (p-value = 0.06194 > 0.05)

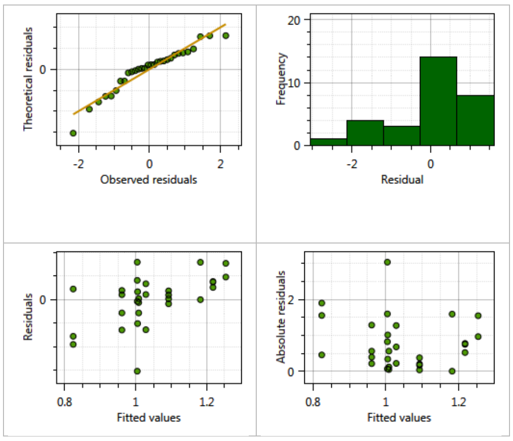

Analysis average movement distance (H4 - Zone 2)

**Analysis model**	Linear mixed model fit by REML: Average_movement_distance_H4_Zone_2 ~ 1 + (1|Genotype_Zone_1:Plant_Zone_1) + (1|Genotype_Zone_2:Plant_Zone_2)
**Transformation**	Natural logarithm

Confidence intervals average movement distance (H4 - Zone 2)

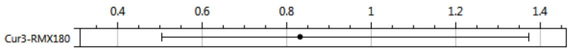

Confidence intervals average movement distance (H4 - Zone 2)

Genotype Zone 1	Genotype Zone 2	Mean	Lower 95% CL	Upper 95% CL	Group
Cur3	RMX180	0.8317	0.5033	1.374	a

Model summary

```
Linear mixed model fit by REML. t-tests use Satterthwaite's method ['lmerModLmerTest']
Formula: Average_movement_distance_H4_Zone_2 ~ 1 + (1 | Genotype_Zone_1:Plant_Zone_1) +      (1 |
Genotype_Zone_2:Plant_Zone_2)
   Data: data

REML criterion at convergence: 115.2

Scaled residuals:
     Min       1Q   Median       3Q      Max
-2.54788 -0.57031  0.00163  0.65023  1.58060

Random effects:
 Groups                       Name        Variance Std.Dev.
 Genotype_Zone_1:Plant_Zone_1 (Intercept) 0.0000   0.0000
 Genotype_Zone_2:Plant_Zone_2 (Intercept) 0.1813   0.4258
 Residual                                 1.1617   1.0778
Number of obs: 37, groups:  Genotype_Zone_1:Plant_Zone_1, 10; Genotype_Zone_2:Plant_Zone_2, 10

Fixed effects:
            Estimate Std. Error      df t value Pr(>|t|)
(Intercept)  -0.1843     0.2234  9.3944  -0.825     0.43
```

Model residuals

Statistic	Value
Sample skewness	-0.5362
Sample excess kurtosis	0.0118
Passed Shapiro Wilk normality test	Yes (p-value = 0.4125 > 0.05)

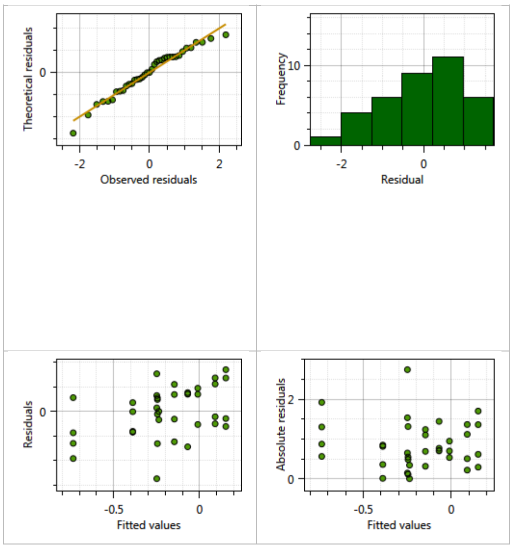

Analysis average movement distance (H5 - Zone 1)

**Analysis model**	Linear mixed model fit by REML: Average_movement_distance_H5_Zone_1 ~ 1 + (1|Genotype_Zone_1:Plant_Zone_1) + (1|Genotype_Zone_2:Plant_Zone_2)
**Transformation**	Natural logarithm

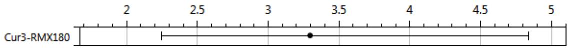

Confidence intervals average movement distance (H5 - Zone 1)

Genotype Zone 1	Genotype Zone 2	Mean	Lower 95% CL	Upper 95% CL	Group
Cur3	RMX180	3.296	2.244	4.842	a

Model summary

```
Linear mixed model fit by REML. t-tests use Satterthwaite's method ['lmerModLmerTest']
Formula: Average_movement_distance_H5_Zone_1 ~ 1 + (1 | Genotype_Zone_1:Plant_Zone_1) +      (1 |
Genotype_Zone_2:Plant_Zone_2)
   Data: data

REML criterion at convergence: 75.2

Scaled residuals:
    Min      1Q  Median      3Q     Max
-2.3694 -0.5425  0.2346  0.4707  1.8836

Random effects:
 Groups                       Name        Variance Std.Dev.
 Genotype_Zone_1:Plant_Zone_1 (Intercept) 0.09135  0.3022
 Genotype_Zone_2:Plant_Zone_2 (Intercept) 0.00000  0.0000
 Residual                                 0.62376  0.7898
Number of obs: 30, groups:  Genotype_Zone_1:Plant_Zone_1, 10; Genotype_Zone_2:Plant_Zone_2, 10

Fixed effects:
            Estimate Std. Error      df t value Pr(>|t|)
(Intercept)   1.1928     0.1742 10.7759   6.845 3.09e-05 ***
---
Signif. codes:  0 '***' 0.001 '**' 0.01 '*' 0.05 '.' 0.1 ' ' 1
```

Model residuals

Statistic	Value
Sample skewness	-0.5661
Sample excess kurtosis	0.9411
Passed Shapiro Wilk normality test	Yes (p-value = 0.1503 > 0.05)

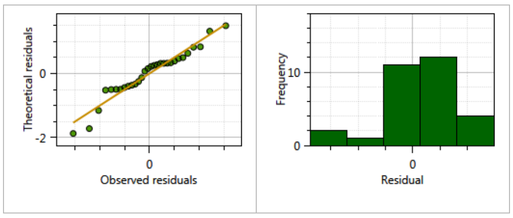

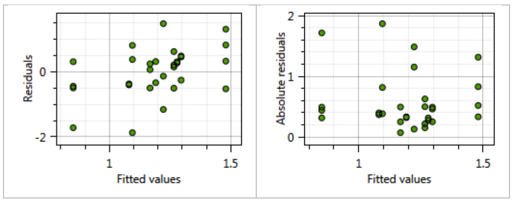

Analysis average movement distance (H5 - Zone 2)

**Analysis model**	Linear mixed model fit by REML: Average_movement_distance_H5_Zone_2 ~ 1 + (1|Genotype_Zone_1:Plant_Zone_1) + (1|Genotype_Zone_2:Plant_Zone_2)
**Transformation**	Natural logarithm

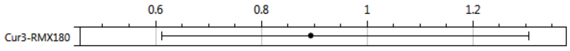

Confidence intervals average movement distance (H5 - Zone 2)

Genotype Zone 1	Genotype Zone 2	Mean	Lower 95% CL	Upper 95% CL	Group
Cur3	RMX180	0.8933	0.6111	1.306	a

Model summary

```
Linear mixed model fit by REML. t-tests use Satterthwaite's method ['lmerModLmerTest']
Formula: Average_movement_distance_H5_Zone_2 ~ 1 + (1 | Genotype_Zone_1:Plant_Zone_1) +      (1 |
Genotype_Zone_2:Plant_Zone_2)
   Data: data

REML criterion at convergence: 123.5

Scaled residuals:
    Min      1Q  Median      3Q     Max
-2.3052 -0.5763  0.1298  0.7823  1.6979

Random effects:
 Groups                       Name        Variance  Std.Dev.
 Genotype_Zone_1:Plant_Zone_1 (Intercept) 3.056e-18 1.748e-09
 Genotype_Zone_2:Plant_Zone_2 (Intercept) 6.282e-17 7.926e-09
 Residual                                 1.373e+00 1.172e+00
Number of obs: 39, groups:  Genotype_Zone_1:Plant_Zone_1, 10; Genotype_Zone_2:Plant_Zone_2, 10

Fixed effects:
            Estimate Std. Error      df t value Pr(>|t|)
(Intercept)  -0.1128     0.1876 38.0000  -0.601    0.551
```

Model residuals

Statistic	Value
Sample skewness	-0.5789
Sample excess kurtosis	-0.06877
Passed Shapiro Wilk normality test	Yes (p-value = 0.1784 > 0.05)

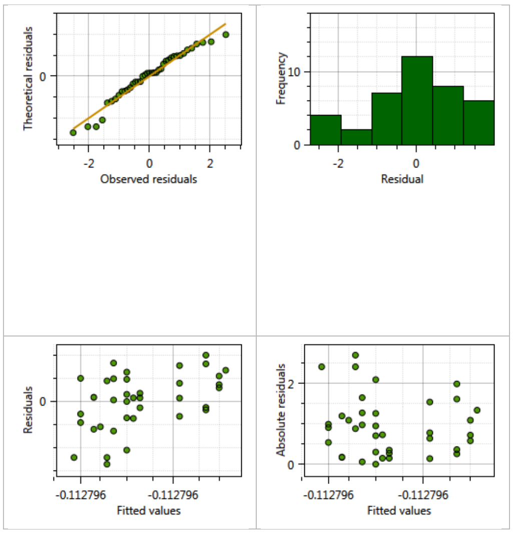

Analysis average movement distance (H6 - Zone 1)

**Analysis model**	Linear mixed model fit by REML: Average_movement_distance_H6_Zone_1 ~ 1 + (1|Genotype_Zone_1:Plant_Zone_1) + (1|Genotype_Zone_2:Plant_Zone_2)
**Transformation**	Natural logarithm

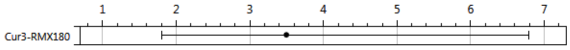

Confidence intervals average movement distance (H6 - Zone 1)

Genotype Zone 1	Genotype Zone 2	Mean	Lower 95% CL	Upper 95% CL	Group
Cur3	RMX180	3.496	1.8	6.793	a

Model summary

```
Linear mixed model fit by REML. t-tests use Satterthwaite's method ['lmerModLmerTest']
Formula: Average_movement_distance_H6_Zone_1 ~ 1 + (1 | Genotype_Zone_1:Plant_Zone_1) +      (1 |
Genotype_Zone_2:Plant_Zone_2)
   Data: data

REML criterion at convergence: 79

Scaled residuals:
    Min      1Q  Median      3Q     Max
-2.0123 -0.4240  0.1497  0.6202  1.4500

Random effects:
 Groups                       Name        Variance Std.Dev.
 Genotype_Zone_1:Plant_Zone_1 (Intercept) 0.0000   0.0000
 Genotype_Zone_2:Plant_Zone_2 (Intercept) 0.5246   0.7243
 Residual                                 0.7625   0.8732
Number of obs: 27, groups:  Genotype_Zone_1:Plant_Zone_1, 10; Genotype_Zone_2:Plant_Zone_2, 10

Fixed effects:
            Estimate Std. Error    df t value Pr(>|t|)
(Intercept)    1.252      0.291 8.505   4.301  0.00227 **
```

```
---
Signif. codes:  0 '***' 0.001 '**' 0.01 '*' 0.05 '.' 0.1 ' ' 1
```

Model residuals

Statistic	Value
Sample skewness	-0.5406
Sample excess kurtosis	-0.1658
Passed Shapiro Wilk normality test	Yes (p-value = 0.4935 > 0.05)

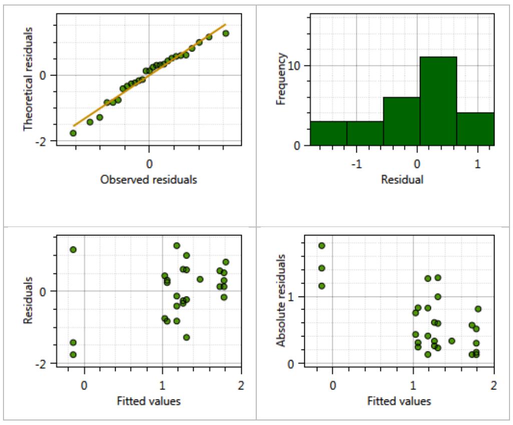

Analysis average movement distance (H6 - Zone 2)

**Analysis model**	Linear mixed model fit by REML: Average_movement_distance_H6_Zone_2 ~ 1 + (1|Genotype_Zone_1:Plant_Zone_1) + (1|Genotype_Zone_2:Plant_Zone_2)
**Transformation**	Natural logarithm

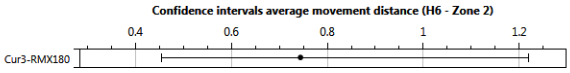

Confidence intervals average movement distance (H6 - Zone 2)

Genotype Zone 1	Genotype Zone 2	Mean	Lower 95% CL	Upper 95% CL	Group
Cur3	RMX180	0.7439	0.4537	1.22	a

Model summary

```
Linear mixed model fit by REML. t-tests use Satterthwaite's method ['lmerModLmerTest']
Formula: Average_movement_distance_H6_Zone_2 ~ 1 + (1 | Genotype_Zone_1:Plant_Zone_1) +      (1 |
Genotype_Zone_2:Plant_Zone_2)
   Data: data

REML criterion at convergence: 129.5

Scaled residuals:
    Min      1Q  Median      3Q     Max
-2.1996 -0.8562  0.2745  0.7633  1.5573

Random effects:
 Groups                       Name        Variance Std.Dev.
 Genotype_Zone_1:Plant_Zone_1 (Intercept) 0.000000 0.00000
 Genotype_Zone_2:Plant_Zone_2 (Intercept) 0.005831 0.07636
 Residual                                 1.750447 1.32304
Number of obs: 38, groups:  Genotype_Zone_1:Plant_Zone_1, 10; Genotype_Zone_2:Plant_Zone_2, 10

Fixed effects:
            Estimate Std. Error      df t value Pr(>|t|)
(Intercept)  -0.2958     0.2160  8.3361  -1.369    0.207
```

Model residuals

Statistic	Value
Sample skewness	-0.5662
Sample excess kurtosis	-0.6657
Passed Shapiro Wilk normality test	Yes (p-value = 0.0605 > 0.05)

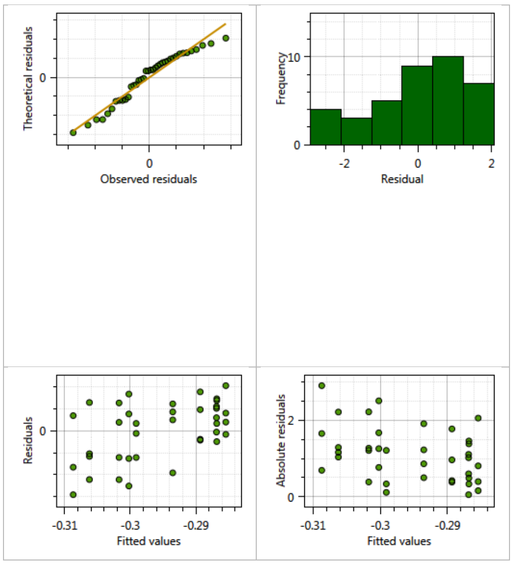

Analysis average movement distance (H7 - Zone 1)

**Analysis model**	Linear mixed model fit by REML: Average_movement_distance_H7_Zone_1 ~ 1 + (1|Genotype_Zone_1:Plant_Zone_1) + (1|Genotype_Zone_2:Plant_Zone_2)
**Transformation**	Natural logarithm

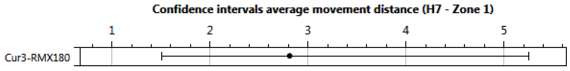

Confidence intervals average movement distance (H7 - Zone 1)

Genotype Zone 1	Genotype Zone 2	Mean	Lower 95% CL	Upper 95% CL	Group
Cur3	RMX180	2.812	1.505	5.256	a

Model summary

```
Linear mixed model fit by REML. t-tests use Satterthwaite's method ['lmerModLmerTest']
Formula: Average_movement_distance_H7_Zone_1 ~ 1 + (1 | Genotype_Zone_1:Plant_Zone_1) +      (1 |
Genotype_Zone_2:Plant_Zone_2)
   Data: data

REML criterion at convergence: 93.2

Scaled residuals:
    Min      1Q  Median      3Q     Max
-3.7819 -0.1907  0.1337  0.3710  1.2744

Random effects:
 Groups                       Name        Variance  Std.Dev.
 Genotype_Zone_2:Plant_Zone_2 (Intercept) 1.578e-01 3.973e-01
 Genotype_Zone_1:Plant_Zone_1 (Intercept) 1.176e-14 1.084e-07
 Residual                                 1.504e+00 1.226e+00
Number of obs: 28, groups:  Genotype_Zone_2:Plant_Zone_2, 10; Genotype_Zone_1:Plant_Zone_1, 9

Fixed effects:
            Estimate Std. Error     df t value Pr(>|t|)
(Intercept)   1.0340     0.2673 7.3945   3.868  0.00553 **
---
Signif. codes:  0 '***' 0.001 '**' 0.01 '*' 0.05 '.' 0.1 ' ' 1
```

Model residuals

Statistic	Value
Sample skewness	-2.411
Sample excess kurtosis	8.447
Passed Shapiro Wilk normality test	No (p-value = 4.246E-05 < 0.05)

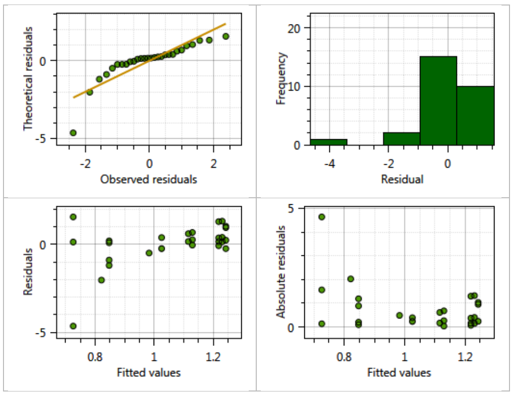

Analysis average movement distance (H7 - Zone 2)

**Analysis model**	Linear mixed model fit by REML: Average_movement_distance_H7_Zone_2 ~ 1 + (1|Genotype_Zone_1:Plant_Zone_1) + (1|Genotype_Zone_2:Plant_Zone_2)
**Transformation**	Natural logarithm

Confidence intervals average movement distance (H7 - Zone 2)

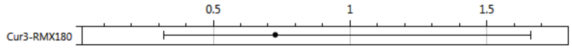

Confidence intervals average movement distance (H7 - Zone 2)

Genotype Zone 1	Genotype Zone 2	Mean	Lower 95% CL	Upper 95% CL	Group
Cur3	RMX180	0.7266	0.3176	1.662	a

Model summary

```
Linear mixed model fit by REML. t-tests use Satterthwaite's method ['lmerModLmerTest']
Formula: Average_movement_distance_H7_Zone_2 ~ 1 + (1 | Genotype_Zone_1:Plant_Zone_1) +      (1 |
Genotype_Zone_2:Plant_Zone_2)
   Data: data

REML criterion at convergence: 134.6

Scaled residuals:
    Min      1Q  Median      3Q     Max
-1.5881 -0.5940 -0.1449  0.6564  1.3616

Random effects:
 Groups                       Name        Variance Std.Dev.
 Genotype_Zone_1:Plant_Zone_1 (Intercept) 0.6414   0.8009
 Genotype_Zone_2:Plant_Zone_2 (Intercept) 0.4355   0.6599
 Residual                                 1.1736   1.0833
Number of obs: 39, groups:  Genotype_Zone_1:Plant_Zone_1, 10; Genotype_Zone_2:Plant_Zone_2, 10

Fixed effects:
            Estimate Std. Error      df t value Pr(>|t|)
(Intercept)  -0.3194     0.3716 10.0430   -0.86     0.41
```

Model residuals

Statistic	Value
Sample skewness	-0.0709
Sample excess kurtosis	-0.9584
Passed Shapiro Wilk normality test	Yes (p-value = 0.203 > 0.05)

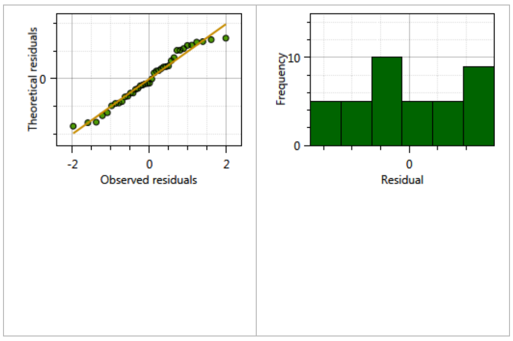

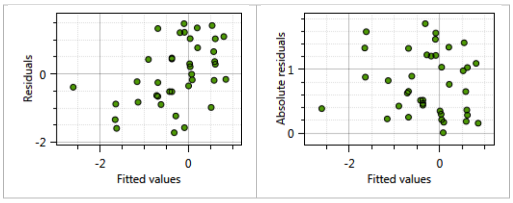

Analysis average movement distance H0 (diff. Zone 1 - Zone 2)

**Analysis model**	Generalized linear mixed model with dispersion factor, formula=cbind(Average_movement_distance_H0_Zone_1,Average_movement_distance_H0_Zone_2) ~ 1 + (1|Genotype_Zone_1:Plant_Zone_1) + (1|Genotype_Zone_2:Plant_Zone_2)
**Transformation**	Logit

Confidence intervals average movement distance H0 (diff. Zone 1 - Zone 2)

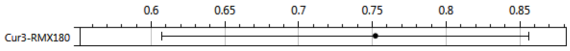

Confidence intervals average movement distance H0 (diff. Zone 1 - Zone 2)

Genotype Zone 1	Genotype Zone 2	Mean	Lower 95% CL	Upper 95% CL	Group
Cur3	RMX180	0.7521	0.6069	0.8563	a

Model summary

```
Linear mixed model fit by REML. t-tests use Satterthwaite's method ['lmerModLmerTest']
Formula: ziFormula
   Data: data
Weights: wi

REML criterion at convergence: 88.7

Scaled residuals:
    Min      1Q  Median      3Q     Max
-1.7369 -0.2223  0.2639  0.6123  1.1484

Random effects:
 Groups                      Name        Variance Std.Dev.
 Genotype_Zone_1:Plant_Zone_1 (Intercept) 0.3365   0.5801
 Genotype_Zone_2:Plant_Zone_2 (Intercept) 0.4183   0.6468
 Residual                                 0.6353   0.7971
Number of obs: 32, groups:  Genotype_Zone_1:Plant_Zone_1, 10; Genotype_Zone_2:Plant_Zone_2, 10

Fixed effects:
            Estimate Std. Error      df t value Pr(>|t|)
(Intercept)   1.1096     0.3072 11.0967   3.612  0.00403 **
---
Signif. codes:  0 '***' 0.001 '**' 0.01 '*' 0.05 '.' 0.1 ' ' 1

Dispersion: 0.7971
```

Model residuals

Statistic	Value
Sample skewness	-0.9714
Sample excess kurtosis	0.1997
Passed Shapiro Wilk normality test	No (p-value = 0.006346 < 0.05)

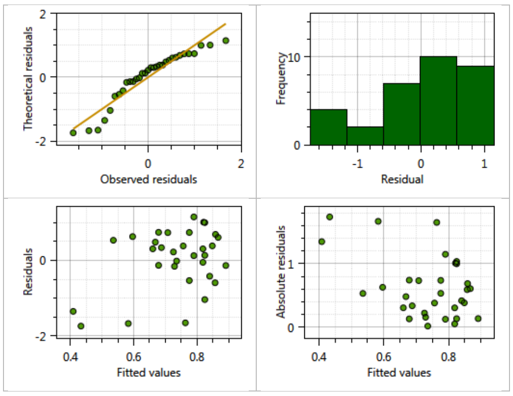

Analysis average movement distance H1 (diff. Zone 1 - Zone 2)

**Analysis model**	Generalized linear mixed model with dispersion factor, formula=cbind(Average_movement_distance_H1_Zone_1,Average_movement_distance_H1_Zone_2) ~ 1 + (1|Genotype_Zone_1:Plant_Zone_1) + (1|Genotype_Zone_2:Plant_Zone_2)
**Transformation**	Logit

Confidence intervals average movement distance H1 (diff. Zone 1 - Zone 2)

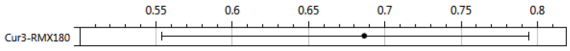

Confidence intervals average movement distance H1 (diff. Zone 1 - Zone 2)

Genotype Zone 1	Genotype Zone 2	Mean	Lower 95% CL	Upper 95% CL	Group
Cur3	RMX180	0.6865	0.5535	0.7945	a

Model summary

```
Linear mixed model fit by REML. t-tests use Satterthwaite's method ['lmerModLmerTest']
Formula: ziFormula
   Data: data
Weights: wi

REML criterion at convergence: 107.9

Scaled residuals:
    Min      1Q  Median      3Q     Max
-2.3508 -0.3239  0.1047  0.7275  1.4998

Random effects:
 Groups                      Name        Variance Std.Dev.
 Genotype_Zone_1:Plant_Zone_1 (Intercept) 0.0306   0.1749
 Genotype_Zone_2:Plant_Zone_2 (Intercept) 0.3658   0.6048
 Residual                                 1.3170   1.1476
Number of obs: 36, groups:  Genotype_Zone_1:Plant_Zone_1, 10; Genotype_Zone_2:Plant_Zone_2, 10

Fixed effects:
            Estimate Std. Error     df t value Pr(>|t|)
(Intercept)   0.7837     0.2526 9.2656   3.103   0.0122 *
---
Signif. codes:  0 '***' 0.001 '**' 0.01 '*' 0.05 '.' 0.1 ' ' 1
```

```
Dispersion: 1.148
```

Model residuals

Statistic	Value
Sample skewness	-0.9039
Sample excess kurtosis	0.6421
Passed Shapiro Wilk normality test	No (p-value = 0.04698 < 0.05)

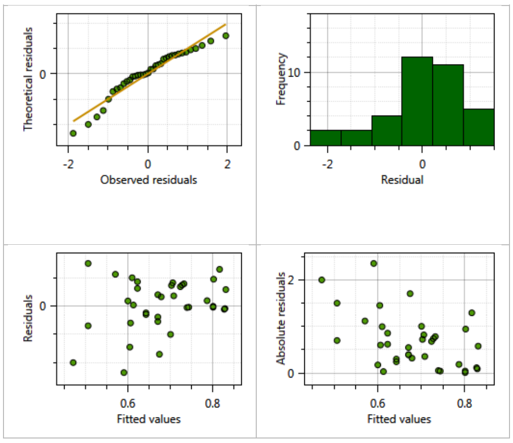

Analysis average movement distance H2 (diff. Zone 1 - Zone 2)

**Analysis model**	Generalized linear mixed model with dispersion factor, formula=cbind(Average_movement_distance_H2_Zone_1,Average_movement_distance_H2_Zone_2) ~ 1 + (1|Genotype_Zone_1:Plant_Zone_1) + (1|Genotype_Zone_2:Plant_Zone_2)
**Transformation**	Logit

Confidence intervals average movement distance H2 (diff. Zone 1 - Zone 2)

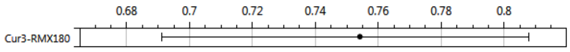

Confidence intervals average movement distance H2 (diff. Zone 1 - Zone 2)

Genotype Zone 1	Genotype Zone 2	Mean	Lower 95% CL	Upper 95% CL	Group
Cur3	RMX180	0.7541	0.691	0.8079	a

Model summary

```
Linear mixed model fit by REML. t-tests use Satterthwaite's method ['lmerModLmerTest']
Formula: ziFormula
   Data: data
Weights: wi

REML criterion at convergence: 99.2

Scaled residuals: 
    Min      1Q  Median      3Q     Max 
-3.2976 -0.4876  0.0476  0.6308  1.5389 

Random effects:
 Groups                       Name        Variance Std.Dev.
 Genotype_Zone_1:Plant_Zone_1 (Intercept) 0.000    0.000   
 Genotype_Zone_2:Plant_Zone_2 (Intercept) 0.000    0.000   
 Residual                                 1.195    1.093   
Number of obs: 34, groups:  Genotype_Zone_1:Plant_Zone_1, 10; Genotype_Zone_2:Plant_Zone_2, 10

Fixed effects:
            Estimate Std. Error      df t value Pr(>|t|)    
(Intercept)   1.1208     0.1552 33.0000   7.221  2.8e-08 ***
---
Signif. codes:  0 '***' 0.001 '**' 0.01 '*' 0.05 '.' 0.1 ' ' 1

Dispersion: 1.093
```

Model residuals

Statistic	Value
Sample skewness	-1.189
Sample excess kurtosis	2.462
Passed Shapiro Wilk normality test	No (p-value = 0.02781 < 0.05)

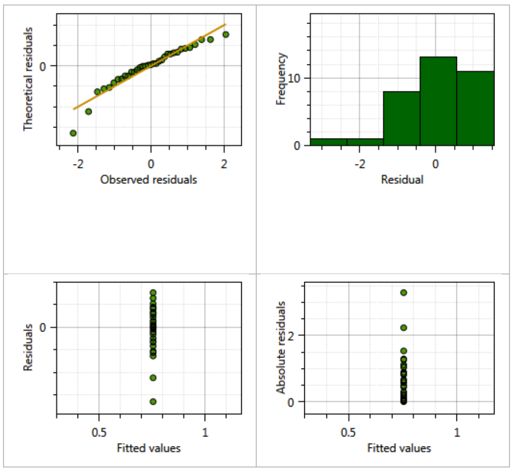

Analysis average movement distance H3 (diff. Zone 1 - Zone 2)

**Analysis model**	Generalized linear mixed model with dispersion factor, formula=cbind(Average_movement_distance_H3_Zone_1,Average_movement_distance_H3_Zone_2) ~ 1 + (1|Genotype_Zone_1:Plant_Zone_1) + (1|Genotype_Zone_2:Plant_Zone_2)
**Transformation**	Logit

Confidence intervals average movement distance H3 (diff. Zone 1 - Zone 2)

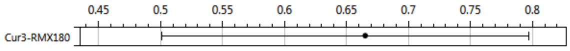

Confidence intervals average movement distance H3 (diff. Zone 1 - Zone 2)

Genotype Zone 1	Genotype Zone 2	Mean	Lower 95% CL	Upper 95% CL	Group
Cur3	RMX180	0.6651	0.5006	0.7973	a

Model summary

```
Linear mixed model fit by REML. t-tests use Satterthwaite's method ['lmerModLmerTest']
Formula: ziFormula
   Data: data
Weights: wi

REML criterion at convergence: 97.9

Scaled residuals: 
    Min      1Q  Median      3Q     Max 
-1.9148 -0.4261 -0.0397  0.6086  1.3697 

Random effects:
 Groups                      Name        Variance Std.Dev.
 Genotype_Zone_1:Plant_Zone_1 (Intercept) 0.2710   0.5206  
 Genotype_Zone_2:Plant_Zone_2 (Intercept) 0.3886   0.6234  
 Residual                                0.7889   0.8882  
Number of obs: 33, groups:  Genotype_Zone_1:Plant_Zone_1, 10; Genotype_Zone_2:Plant_Zone_2, 10

Fixed effects:
            Estimate Std. Error     df t value Pr(>|t|)  
(Intercept)   0.6861     0.2905 7.1679   2.362   0.0494 *
---
Signif. codes:  0 '***' 0.001 '**' 0.01 '*' 0.05 '.' 0.1 ' ' 1

Dispersion: 0.8882
```

Model residuals

Statistic	Value
Sample skewness	-0.3484
Sample excess kurtosis	-0.172
Passed Shapiro Wilk normality test	Yes (p-value = 0.5998 > 0.05)

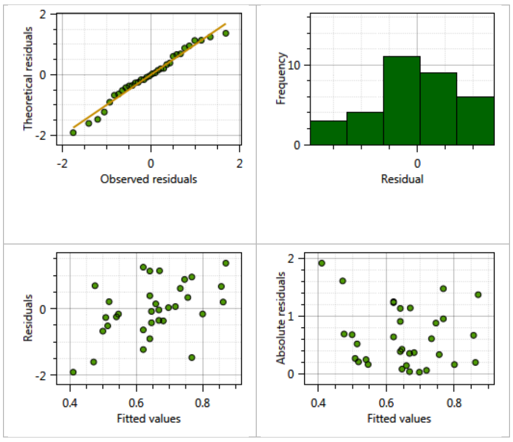

Analysis average movement distance H4 (diff. Zone 1 - Zone 2)

**Analysis model**	Generalized linear mixed model with dispersion factor, formula=cbind(Average_movement_distance_H4_Zone_1,Average_movement_distance_H4_Zone_2) ~ 1 + (1|Genotype_Zone_1:Plant_Zone_1) + (1|Genotype_Zone_2:Plant_Zone_2)
**Transformation**	Logit

Confidence intervals average movement distance H4 (diff. Zone 1 - Zone 2)

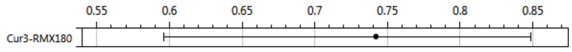

Confidence intervals average movement distance H4 (diff. Zone 1 - Zone 2)

Genotype Zone 1	Genotype Zone 2	Mean	Lower 95% CL	Upper 95% CL	Group
Cur3	RMX180	0.742	0.5958	0.8488	a

Model summary

```
Linear mixed model fit by REML. t-tests use Satterthwaite's method ['lmerModLmerTest']
Formula: ziFormula
   Data: data
Weights: wi

REML criterion at convergence: 80.9

Scaled residuals: 
     Min       1Q   Median       3Q      Max 
-1.76460 -0.78153  0.00054  0.68940  1.38088 

Random effects:
 Groups                      Name        Variance Std.Dev.
 Genotype_Zone_1:Plant_Zone_1 (Intercept) 0.0000   0.0000  
 Genotype_Zone_2:Plant_Zone_2 (Intercept) 0.6296   0.7935  
 Residual                                0.6461   0.8038  
Number of obs: 28, groups:  Genotype_Zone_1:Plant_Zone_1, 10; Genotype_Zone_2:Plant_Zone_2, 10

Fixed effects:
            Estimate Std. Error     df t value Pr(>|t|)   
(Intercept)   1.0565     0.2952 8.9504   3.578    0.006 **
---
Signif. codes:  0 '***' 0.001 '**' 0.01 '*' 0.05 '.' 0.1 ' ' 1

Dispersion: 0.8038
```

Model residuals

Statistic	Value
Sample skewness	-0.1464
Sample excess kurtosis	-0.8148
Passed Shapiro Wilk normality test	Yes (p-value = 0.5972 > 0.05)

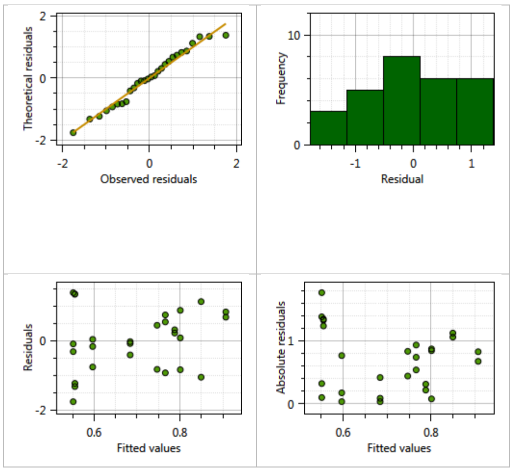

Analysis average movement distance H5 (diff. Zone 1 - Zone 2)

**Analysis model**	Generalized linear mixed model with dispersion factor, formula=cbind(Average_movement_distance_H5_Zone_1,Average_movement_distance_H5_Zone_2) ~ 1 + (1|Genotype_Zone_1:Plant_Zone_1) + (1|Genotype_Zone_2:Plant_Zone_2)
**Transformation**	Logit

Confidence intervals average movement distance H5 (diff. Zone 1 - Zone 2)

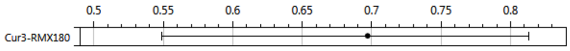

Confidence intervals average movement distance H5 (diff. Zone 1 - Zone 2)

Genotype Zone 1	Genotype Zone 2	Mean	Lower 95% CL	Upper 95% CL	Group
Cur3	RMX180	0.6971	0.5486	0.8133	a

Model summary

```
Linear mixed model fit by REML. t-tests use Satterthwaite's method ['lmerModLmerTest']
Formula: ziFormula
   Data: data
Weights: wi

REML criterion at convergence: 99.9

Scaled residuals:
     Min       1Q   Median       3Q      Max
-2.14457 -0.41393  0.03687  0.39553  1.45128

Random effects:
 Groups                      Name        Variance Std.Dev.
 Genotype_Zone_1:Plant_Zone_1 (Intercept) 0.2253   0.4747
 Genotype_Zone_2:Plant_Zone_2 (Intercept) 0.2672   0.5169
 Residual                                0.9214   0.9599
Number of obs: 31, groups:  Genotype_Zone_1:Plant_Zone_1, 10; Genotype_Zone_2:Plant_Zone_2, 10

Fixed effects:
            Estimate Std. Error     df t value Pr(>|t|)
(Intercept)   0.8333     0.2793 8.4511   2.983   0.0165 *
---
Signif. codes:  0 '***' 0.001 '**' 0.01 '*' 0.05 '.' 0.1 ' ' 1

Dispersion: 0.9599
```

Model residuals

Statistic	Value
Sample skewness	-0.3439
Sample excess kurtosis	0.1728
Passed Shapiro Wilk normality test	Yes (p-value = 0.5249 > 0.05)

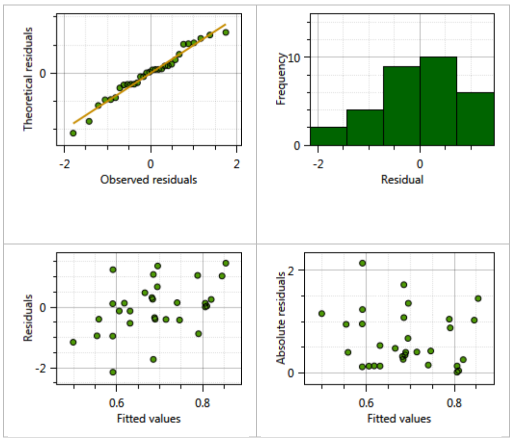

Analysis average movement distance H6 (diff. Zone 1 - Zone 2)

**Analysis model**	Generalized linear mixed model with dispersion factor, formula=cbind(Average_movement_distance_H6_Zone_1,Average_movement_distance_H6_Zone_2) ~ 1 + (1|Genotype_Zone_1:Plant_Zone_1) + (1|Genotype_Zone_2:Plant_Zone_2)
**Transformation**	Logit

Confidence intervals average movement distance H6 (diff. Zone 1 - Zone 2)

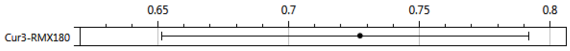

Confidence intervals average movement distance H6 (diff. Zone 1 - Zone 2)

Genotype Zone 1	Genotype Zone 2	Mean	Lower 95% CL	Upper 95% CL	Group
Cur3	RMX180	0.7273	0.6513	0.792	a

Model summary

```
Linear mixed model fit by REML. t-tests use Satterthwaite's method ['lmerModLmerTest']
Formula: ziFormula
   Data: data
```

```
Weights: wi

REML criterion at convergence: 64.1

Scaled residuals:
    Min      1Q  Median      3Q     Max
-1.8268 -0.5901 -0.1812  0.4946  1.8278

Random effects:
 Groups                        Name        Variance Std.Dev.
 Genotype_Zone_1:Plant_Zone_1 (Intercept) 0.06083  0.2466
 Genotype_Zone_2:Plant_Zone_2 (Intercept) 0.00000  0.0000
 Residual                                 0.67211  0.8198
Number of obs: 26, groups:  Genotype_Zone_1:Plant_Zone_1, 10; Genotype_Zone_2:Plant_Zone_2, 10

Fixed effects:
            Estimate Std. Error     df t value Pr(>|t|)
(Intercept)   0.9808     0.1594 9.8218   6.153 0.000116 ***
---
Signif. codes:  0 '***' 0.001 '**' 0.01 '*' 0.05 '.' 0.1 ' ' 1

Dispersion: 0.8198
```

Model residuals

Statistic	Value
Sample skewness	0.1692
Sample excess kurtosis	-0.5367
Passed Shapiro Wilk normality test	Yes (p-value = 0.7738 > 0.05)

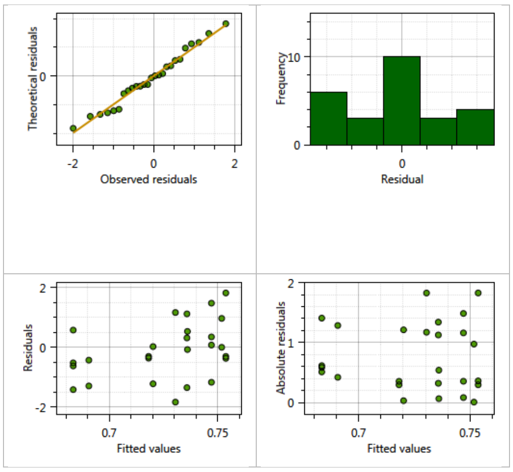

Analysis average movement distance H7 (diff. Zone 1 - Zone 2)

**Analysis model**	Generalized linear mixed model with dispersion factor, formula=cbind(Average_movement_distance_H7_Zone_1,Average_movement_distance_H7_Zone_2) ~ 1 + (1|Genotype_Zone_1:Plant_Zone_1) + (1|Genotype_Zone_2:Plant_Zone_2)
**Transformation**	Logit

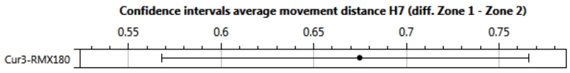

Confidence intervals average movement distance H7 (diff. Zone 1 - Zone 2)

Genotype Zone 1	Genotype Zone 2	Mean	Lower 95% CL	Upper 95% CL	Group
Cur3	RMX180	0.6748	0.5678	0.7662	a

Model summary

```
Linear mixed model fit by REML. t-tests use Satterthwaite's method ['lmerModLmerTest']
Formula: ziFormula
   Data: data
Weights: wi

REML criterion at convergence: 81.7

Scaled residuals:
     Min       1Q   Median       3Q      Max
-1.89826 -0.70426  0.07844  0.81380  1.25545

Random effects:
 Groups                        Name        Variance Std.Dev.
 Genotype_Zone_2:Plant_Zone_2 (Intercept) 0.0664   0.2577
 Genotype_Zone_1:Plant_Zone_1 (Intercept) 0.0000   0.0000
 Residual                                 1.0392   1.0194
Number of obs: 28, groups:  Genotype_Zone_2:Plant_Zone_2, 10; Genotype_Zone_1:Plant_Zone_1, 9

Fixed effects:
            Estimate Std. Error     df t value Pr(>|t|)
(Intercept)   0.7299     0.1869 6.0237   3.905  0.00788 **
---
Signif. codes:  0 '***' 0.001 '**' 0.01 '*' 0.05 '.' 0.1 ' ' 1

Dispersion: 1.019
```

Model residuals

Statistic	Value
Sample skewness	-0.4784
Sample excess kurtosis	-0.9836
Passed Shapiro Wilk normality test	Yes (p-value = 0.06193 > 0.05)

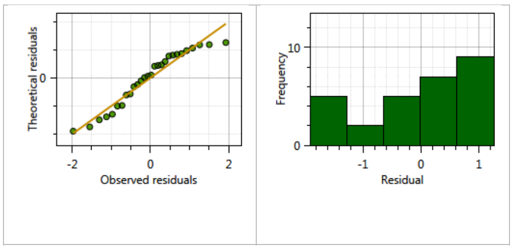

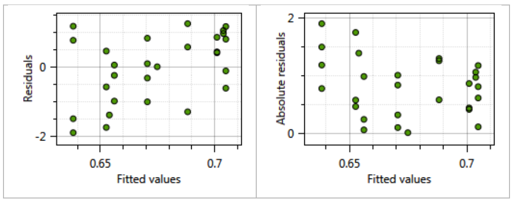

Average movement duration per zone

Selected zones	Zone 1, Zone 2
Data transformation	Natural logarithm
Analysis	Zone difference analysis

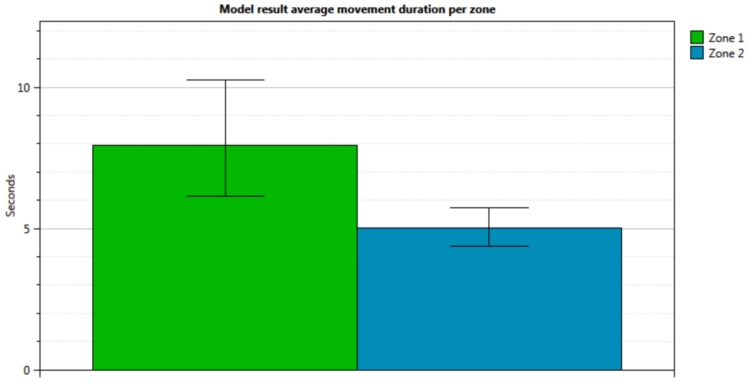

Results difference tests Zone 1 - Zone 2: p values and 95% confidence intervals of the difference on the transformed scale for each statistic.

Behaviour statistic	Cur3-RMX180
Average movement duration (diff. Zone 1 - Zone 2)	p=2.58E-11****<br>[0.419, 0.653]

The model predictions and 95% confidence intervals for each statistic.

Statistic	Cur3-RMX180	Remark
Average movement duration (Zone 1)	7.95<br>[6.15, 10.3]	CR
Average movement duration (Zone 2)	5<br>[4.37, 5.73]	CR

CR = Check residuals

Data summary

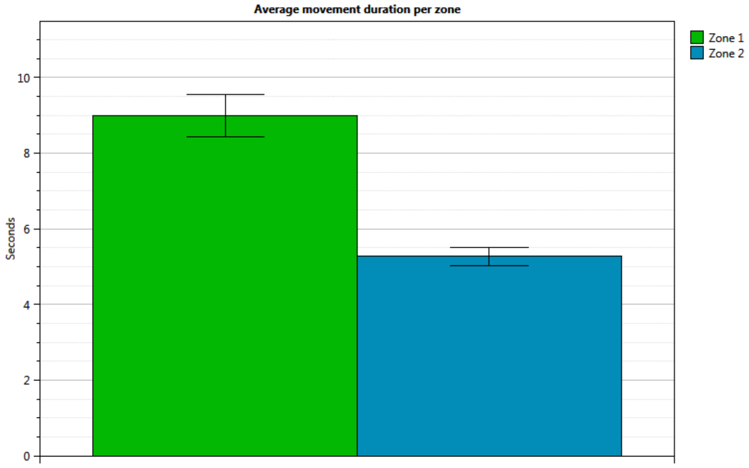

Genotype Zone 1	Genotype Zone 2	Genotype Zone 3	Mean Zone 1	StdErr Zone 1	Mean Zone 2	StdErr Zone 2
Cur3	RMX180	Neutral	9	0.56	5.26	0.23

Analysis average movement duration (Zone 1)

**Analysis model**	Linear mixed model fit by REML: Average_movement_duration_Zone_1 ~ 1 + (1|Genotype_Zone_1:Plant_Zone_1) + (1|Genotype_Zone_2:Plant_Zone_2)
**Transformation**	Natural logarithm

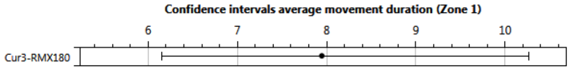

Confidence intervals average movement duration (Zone 1)

Genotype Zone 1	Genotype Zone 2	Mean	Lower 95% CL	Upper 95% CL	Group
Cur3	RMX180	7.946	6.149	10.27	a

Model summary

```
Linear mixed model fit by REML. t-tests use Satterthwaite's method ['lmerModLmerTest']
Formula: Average_movement_duration_Zone_1 ~ 1 + (1 | Genotype_Zone_1:Plant_Zone_1) +      (1 |
Genotype_Zone_2:Plant_Zone_2)
   Data: data

REML criterion at convergence: 73.3

Scaled residuals:
    Min      1Q  Median      3Q     Max
-4.0541 -0.2324  0.3074  0.4970  1.2577

Random effects:
 Groups                       Name        Variance Std.Dev.
 Genotype_Zone_1:Plant_Zone_1 (Intercept) 0.04313  0.2077
```

```
 Genotype_Zone_2:Plant_Zone_2 (Intercept) 0.00000  0.0000
 Residual                                 0.33214  0.5763
Number of obs: 39, groups:  Genotype_Zone_1:Plant_Zone_1, 10; Genotype_Zone_2:Plant_Zone_2, 10

Fixed effects:
            Estimate Std. Error     df t value Pr(>|t|)
(Intercept)   2.0727     0.1133 8.9994   18.29    2e-08 ***
---
Signif. codes:  0 '***' 0.001 '**' 0.01 '*' 0.05 '.' 0.1 ' ' 1
```

Model residuals

Statistic	Value
Sample skewness	-2.611
Sample excess kurtosis	8.476
Passed Shapiro Wilk normality test	No (p-value = 3.747E-07 < 0.05)

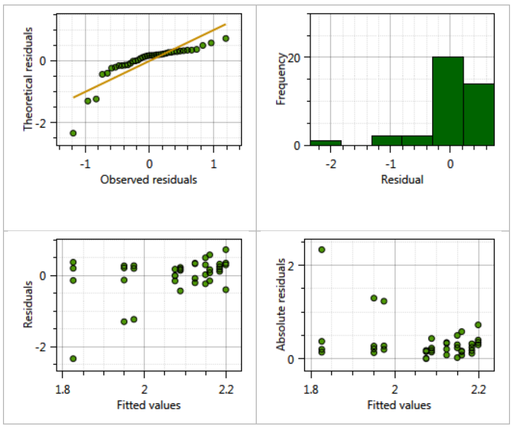

Analysis average movement duration (Zone 2)

Analysis model	Linear mixed model fit by REML: Average_movement_duration_Zone_2 ~ 1 + (1|Genotype_Zone_1:Plant_Zone_1) + (1|Genotype_Zone_2:Plant_Zone_2)
Transformation	Natural logarithm

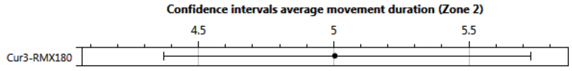

Confidence intervals average movement duration (Zone 2)

Genotype Zone 1	Genotype Zone 2	Mean	Lower 95% CL	Upper 95% CL	Group
Cur3	RMX180	5.004	4.37	5.73	a

Model summary

```
Linear mixed model fit by REML. t-tests use Satterthwaite's method ['lmerModLmerTest']
Formula: Average_movement_duration_Zone_2 ~ 1 + (1 | Genotype_Zone_1:Plant_Zone_1) +      (1 |
Genotype_Zone_2:Plant_Zone_2)
   Data: data

REML criterion at convergence: 31.4

Scaled residuals:
     Min       1Q   Median       3Q      Max
-2.91276 -0.29127  0.09983  0.49370  1.47134

Random effects:
 Groups                       Name        Variance Std.Dev.
 Genotype_Zone_1:Plant_Zone_1 (Intercept) 0.000000 0.0000
 Genotype_Zone_2:Plant_Zone_2 (Intercept) 0.004816 0.0694
 Residual                                 0.117320 0.3425
Number of obs: 39, groups:  Genotype_Zone_1:Plant_Zone_1, 10; Genotype_Zone_2:Plant_Zone_2, 10

Fixed effects:
            Estimate Std. Error     df t value Pr(>|t|)
(Intercept)   1.6102     0.0591 8.2808   27.25 2.12e-09 ***
---
Signif. codes:  0 '***' 0.001 '**' 0.01 '*' 0.05 '.' 0.1 ' ' 1
```

Model residuals

Statistic	Value
Sample skewness	-1.756
Sample excess kurtosis	3.823
Passed Shapiro Wilk normality test	No (p-value = 1.314E-05 < 0.05)

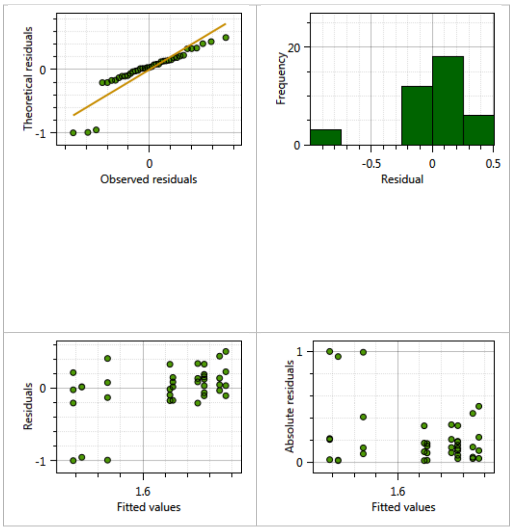

Analysis average movement duration (diff. Zone 1 - Zone 2)

Analysis model	Generalized linear mixed model with dispersion factor, formula=cbind(Average_movement_duration_Zone_1,Average_movement_duration_Zone_2) ~ 1 + (1|Genotype_Zone_1:Plant_Zone_1) + (1|Genotype_Zone_2:Plant_Zone_2)
Transformation	Logit

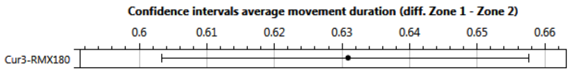

Confidence intervals average movement duration (diff. Zone 1 - Zone 2)

Genotype Zone 1	Genotype Zone 2	Mean	Lower 95% CL	Upper 95% CL	Group
Cur3	RMX180	0.6309	0.6033	0.6577	a

Model summary

```
Linear mixed model fit by REML. t-tests use Satterthwaite's method ['lmerModLmerTest']
Formula: ziFormula
   Data: data
Weights: wi

REML criterion at convergence: 36.8

Scaled residuals: 
    Min      1Q  Median      3Q     Max 
-2.5921 -0.6428 -0.2202  0.6305  2.7771 

Random effects:
 Groups                      Name        Variance  Std.Dev.
 Genotype_Zone_1:Plant_Zone_1 (Intercept) 0.000e+00 0.00e+00
 Genotype_Zone_2:Plant_Zone_2 (Intercept) 2.191e-16 1.48e-08
 Residual                                4.316e-01 6.57e-01
Number of obs: 39, groups:  Genotype_Zone_1:Plant_Zone_1, 10; Genotype_Zone_2:Plant_Zone_2, 10

Fixed effects:
            Estimate Std. Error       df t value Pr(>|t|)    
(Intercept)  0.53603    0.05773 38.00000   9.285 2.58e-11 ***
---
Signif. codes:  0 '***' 0.001 '**' 0.01 '*' 0.05 '.' 0.1 ' ' 1

Dispersion: 0.657
```

Model residuals

Statistic	Value
Sample skewness	0.1189
Sample excess kurtosis	1.223
Passed Shapiro Wilk normality test	Yes (p-value = 0.5398 > 0.05)

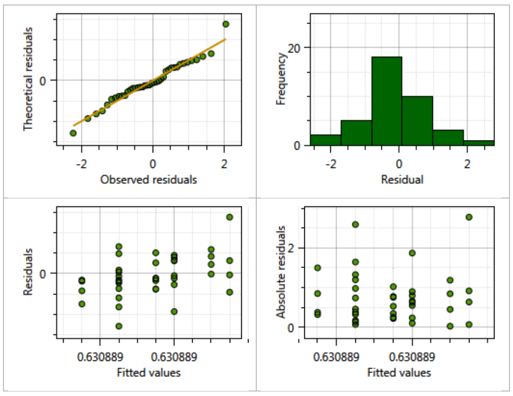

Average movement duration per zone per hour

Selected hours	0, 1, 2, 3, 4, 5, 6, 7
Selected zones	Zone 1, Zone 2
Data transformation	Natural logarithm
Analysis	Zone difference analysis

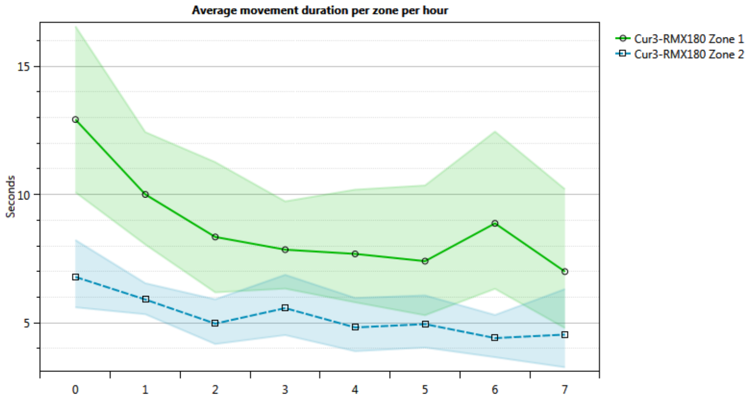

Results difference tests Zone 1 - Zone 2: p values and 95% confidence intervals of the difference on the transformed scale for each statistic.

Behaviour statistic	Cur3-RMX180	Remark
Average movement duration H0 (diff. Zone 1 - Zone 2)	p=0.000892***<br>[0.416, 1.01]	
Average movement duration H1 (diff. Zone 1 - Zone 2)	p=0.000126***<br>[0.354, 0.787]	
Average movement duration H2 (diff. Zone 1 - Zone 2)	p=0.0177*<br>[0.104, 0.835]	
Average movement duration H3 (diff. Zone 1 - Zone 2)	p=0.082<br>[-0.0421, 0.561]	CR
Average movement duration H4 (diff. Zone 1 - Zone 2)	p=0.00331**<br>[0.231, 0.777]	
Average movement duration H5 (diff. Zone 1 - Zone 2)	p=0.0488*<br>[0.00278, 0.779]	
Average movement duration H6 (diff. Zone 1 - Zone 2)	p=3.56E-06****<br>[0.4, 0.828]	
Average movement duration H7 (diff. Zone 1 - Zone 2)	p=0.0149*<br>[0.0976, 0.665]	

CR = Check residuals

The model predictions and 95% confidence intervals for each statistic.

Statistic	Cur3-RMX180	Remark
Average movement duration (H0 - Zone 1)	12.9<br>[10.1, 16.6]	CR
Average movement duration (H0 - Zone 2)	6.79<br>[5.6, 8.23]	
Average movement duration (H1 - Zone 1)	10<br>[8.05, 12.4]	CR
Average movement duration (H1 - Zone 2)	5.91<br>[5.34, 6.55]	CR
Average movement duration (H2 - Zone 1)	8.35<br>[6.19, 11.3]	
Average movement duration (H2 - Zone 2)	4.97<br>[4.17, 5.92]	CR
Average movement duration (H3 - Zone 1)	7.85<br>[6.33, 9.74]	
Average movement duration (H3 - Zone 2)	5.58<br>[4.52, 6.87]	
Average movement duration (H4 - Zone 1)	7.69<br>[5.8, 10.2]	CR
Average movement duration (H4 - Zone 2)	4.82<br>[3.89, 5.97]	
Average movement duration (H5 - Zone 1)	7.41<br>[5.3, 10.4]	CR
Average movement duration (H5 - Zone 2)	4.95<br>[4.04, 6.07]	CR
Average movement duration (H6 - Zone 1)	8.88<br>[6.33, 12.5]	CR
Average movement duration (H6 - Zone 2)	4.41<br>[3.66, 5.31]	CR
Average movement duration (H7 - Zone 1)	7<br>[4.8, 10.2]	CR
Average movement duration (H7 - Zone 2)	4.54<br>[3.27, 6.32]	

CR = Check residuals

Data summary

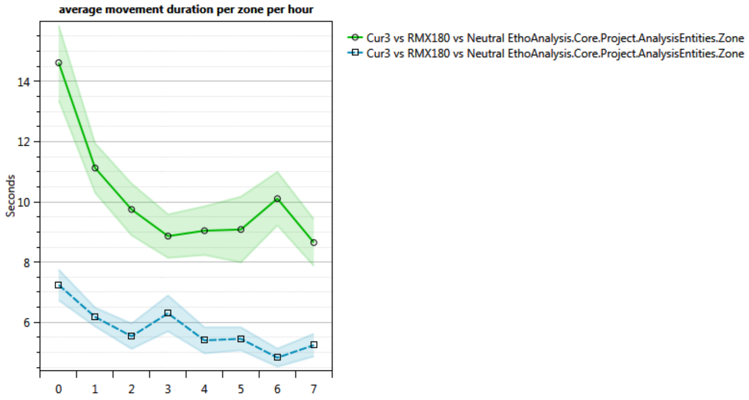

Genotype Zone 1	Genotype Zone 2	Genotype Zone 3	Mean H0 - Zone 1	StdErr H0 - Zone 1	Mean H0 - Zone 2	StdErr H0 - Zone 2	Mean H1 - Zone 1	StdErr H1 - Zone 1	Mean H1 - Zone 2	StdErr H1 - Zone 2	Mean H2 - Zone 1	StdErr H2 - Zone 1	Mean H2 - Zone 2	StdErr H2 - Zone 2	Mean H3 - Zone 1	StdErr H3 - Zone 1	Mean H3 - Zone 2	StdErr H3 - Zone 2	Mean H4 - Zone 1	StdErr H4 - Zone 1	Mean H4 - Zone 2	StdErr H4 - Zone 2	Mean H5 - Zone 1	StdErr H5 - Zone 1	Mean H5 - Zone 2	StdErr H5 - Zone 2	Mean H6 - Zone 1	StdErr H6 - Zone 1	Mean H6 - Zone 2	StdErr H6 - Zone 2
Cur3	RMX180	Neutral	14.62	1.24	7.25	0.51	11.12	0.82	6.18	0.31	9.75	0.86	5.54	0.42	8.86	0.72	6.3	0.6	9.04	0.81	5.4	0.44	9.08	1.09	5.45	0.38	10.11	0.89	4.83	0.3

Analysis average movement duration (H0 - Zone 1)

**Analysis model**	Linear mixed model fit by REML: Average_movement_duration_H0_Zone_1 ~ 1 + (1|Genotype_Zone_1:Plant_Zone_1) + (1|Genotype_Zone_2:Plant_Zone_2)
**Transformation**	Natural logarithm

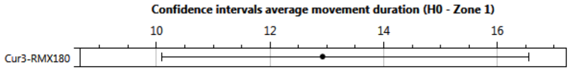

Confidence intervals average movement duration (H0 - Zone 1)

Genotype Zone 1	Genotype Zone 2	Mean	Lower 95% CL	Upper 95% CL	Group
Cur3	RMX180	12.92	10.09	16.56	a

Model summary

```
Linear mixed model fit by REML. t-tests use Satterthwaite's method ['lmerModLmerTest']
Formula: Average_movement_duration_H0_Zone_1 ~ 1 + (1 | Genotype_Zone_1:Plant_Zone_1) +      (1 |
Genotype_Zone_2:Plant_Zone_2)
   Data: data

REML criterion at convergence: 54

Scaled residuals: 
    Min      1Q  Median      3Q     Max 
-3.3621 -0.3663  0.0321  0.4087  1.9378 

Random effects:
 Groups                       Name        Variance Std.Dev.
 Genotype_Zone_1:Plant_Zone_1 (Intercept) 0.005997 0.07744 
 Genotype_Zone_2:Plant_Zone_2 (Intercept) 0.000000 0.00000 
 Residual                                 0.278653 0.52788 
Number of obs: 33, groups:  Genotype_Zone_1:Plant_Zone_1, 10; Genotype_Zone_2:Plant_Zone_2, 10

Fixed effects:
            Estimate Std. Error      df t value Pr(>|t|)    
(Intercept)  2.55908    0.09538 4.82653   26.83 1.93e-06 ***
---
Signif. codes:  0 '***' 0.001 '**' 0.01 '*' 0.05 '.' 0.1 ' ' 1
```

Model residuals

Statistic	Value
Sample skewness	-1.037
Sample excess kurtosis	3.32
Passed Shapiro Wilk normality test	No (p-value = 0.02896 < 0.05)

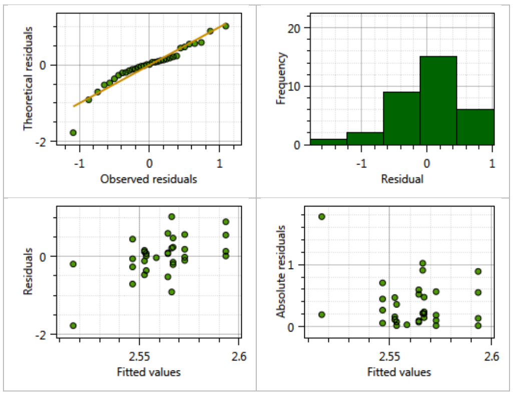

Analysis average movement duration (H0 - Zone 2)

**Analysis model**	Linear mixed model fit by REML: Average_movement_duration_H0_Zone_2 ~ 1 + (1|Genotype_Zone_1:Plant_Zone_1) + (1|Genotype_Zone_2:Plant_Zone_2)
**Transformation**	Natural logarithm

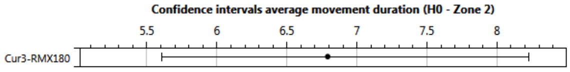

Confidence intervals average movement duration (H0 - Zone 2)

Genotype Zone 1	Genotype Zone 2	Mean	Lower 95% CL	Upper 95% CL	Group
Cur3	RMX180	6.791	5.604	8.229	a

Model summary

```
Linear mixed model fit by REML. t-tests use Satterthwaite's method ['lmerModLmerTest']
Formula: Average_movement_duration_H0_Zone_2 ~ 1 + (1 | Genotype_Zone_1:Plant_Zone_1) +      (1 |
Genotype_Zone_2:Plant_Zone_2)
   Data: data

REML criterion at convergence: 32

Scaled residuals:
     Min       1Q   Median       3Q      Max
-1.66493 -0.62514  0.00281  0.48572  2.24009

Random effects:
 Groups                       Name        Variance Std.Dev.
 Genotype_Zone_1:Plant_Zone_1 (Intercept) 0.003904 0.06248
 Genotype_Zone_2:Plant_Zone_2 (Intercept) 0.022952 0.15150
 Residual                                 0.111452 0.33384
Number of obs: 36, groups:  Genotype_Zone_1:Plant_Zone_1, 10; Genotype_Zone_2:Plant_Zone_2, 10

Fixed effects:
            Estimate Std. Error      df t value Pr(>|t|)
(Intercept)  1.91556    0.07647 5.41478   25.05 8.29e-07 ***
---
Signif. codes:  0 '***' 0.001 '**' 0.01 '*' 0.05 '.' 0.1 ' ' 1
```

Model residuals

Statistic	Value
Sample skewness	0.5883
Sample excess kurtosis	0.4034
Passed Shapiro Wilk normality test	Yes (p-value = 0.2081 > 0.05)

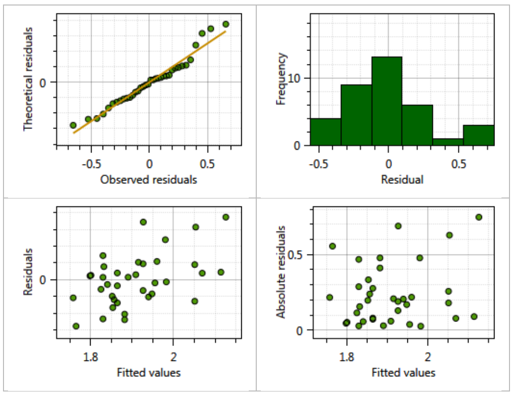

Analysis average movement duration (H1 - Zone 1)

**Analysis model**	Linear mixed model fit by REML: Average_movement_duration_H1_Zone_1 ~ 1 + (1|Genotype_Zone_1:Plant_Zone_1) + (1|Genotype_Zone_2:Plant_Zone_2)
**Transformation**	Natural logarithm

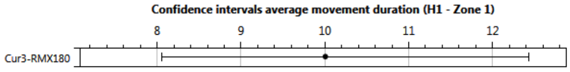

Confidence intervals average movement duration (H1 - Zone 1)

Genotype Zone 1	Genotype Zone 2	Mean	Lower 95% CL	Upper 95% CL	Group
Cur3	RMX180	10.01	8.051	12.43	a

Model summary

```
Linear mixed model fit by REML. t-tests use Satterthwaite's method ['lmerModLmerTest']
Formula: Average_movement_duration_H1_Zone_1 ~ 1 + (1 | Genotype_Zone_1:Plant_Zone_1) +      (1 |
Genotype_Zone_2:Plant_Zone_2)
   Data: data

REML criterion at convergence: 55

Scaled residuals:
    Min      1Q  Median      3Q     Max
-4.0344 -0.4744  0.2194  0.4840  2.0994

Random effects:
 Groups                       Name        Variance Std.Dev.
 Genotype_Zone_1:Plant_Zone_1 (Intercept) 0.00000  0.0000
 Genotype_Zone_2:Plant_Zone_2 (Intercept) 0.02519  0.1587
```

```
 Residual                                 0.23407  0.4838
Number of obs: 36, groups:  Genotype_Zone_1:Plant_Zone_1, 10; Genotype_Zone_2:Plant_Zone_2, 10

Fixed effects:
            Estimate Std. Error      df t value Pr(>|t|)
(Intercept)  2.30315    0.09516 8.47230    24.2 4.05e-09 ***
---
Signif. codes:  0 '***' 0.001 '**' 0.01 '*' 0.05 '.' 0.1 ' ' 1
```

Model residuals

Statistic	Value
Sample skewness	-1.97
Sample excess kurtosis	8.263
Passed Shapiro Wilk normality test	No (p-value = 5.626E-05 < 0.05)

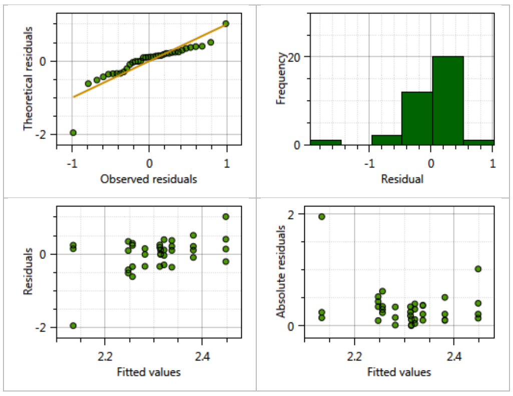

Analysis average movement duration (H1 - Zone 2)

**Analysis model**	Linear mixed model fit by REML: Average_movement_duration_H1_Zone_2 ~ 1 + (1|Genotype_Zone_1:Plant_Zone_1) + (1|Genotype_Zone_2:Plant_Zone_2)
**Transformation**	Natural logarithm

Confidence intervals average movement duration (H1 - Zone 2)

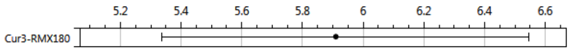

Confidence intervals average movement duration (H1 - Zone 2)

Genotype Zone 1	Genotype Zone 2	Mean	Lower 95% CL	Upper 95% CL	Group
Cur3	RMX180	5.91	5.335	6.547	a

Model summary

```
Linear mixed model fit by REML. t-tests use Satterthwaite's method ['lmerModLmerTest']
Formula: Average_movement_duration_H1_Zone_2 ~ 1 + (1 | Genotype_Zone_1:Plant_Zone_1) +      (1 |
Genotype_Zone_2:Plant_Zone_2)
   Data: data

REML criterion at convergence: 20.7

Scaled residuals:
    Min      1Q  Median      3Q     Max
-3.3717 -0.4548 -0.1064  0.6207  2.5004

Random effects:
 Groups                       Name        Variance Std.Dev.
 Genotype_Zone_1:Plant_Zone_1 (Intercept) 0.00000  0.0000
 Genotype_Zone_2:Plant_Zone_2 (Intercept) 0.00000  0.0000
 Residual                                 0.09418  0.3069
Number of obs: 37, groups:  Genotype_Zone_1:Plant_Zone_1, 10; Genotype_Zone_2:Plant_Zone_2, 10

Fixed effects:
            Estimate Std. Error       df t value Pr(>|t|)
(Intercept)  1.77663    0.05045 36.00000   35.22   <2e-16 ***
---
Signif. codes:  0 '***' 0.001 '**' 0.01 '*' 0.05 '.' 0.1 ' ' 1
```

Model residuals

Statistic	Value
Sample skewness	-0.475
Sample excess kurtosis	3.141
Passed Shapiro Wilk normality test	No (p-value = 0.03997 < 0.05)

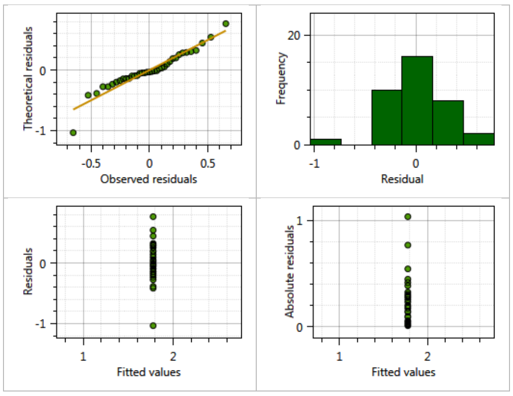

Analysis average movement duration (H2 - Zone 1)

**Analysis model**	Linear mixed model fit by REML: Average_movement_duration_H2_Zone_1 ~ 1 + (1|Genotype_Zone_1:Plant_Zone_1) + (1|Genotype_Zone_2:Plant_Zone_2)
**Transformation**	Natural logarithm

Confidence intervals average movement duration (H2 - Zone 1)

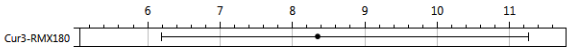

Confidence intervals average movement duration (H2 - Zone 1)

Genotype Zone 1	Genotype Zone 2	Mean	Lower 95% CL	Upper 95% CL	Group
Cur3	RMX180	8.348	6.186	11.27	a

Model summary

```
Linear mixed model fit by REML. t-tests use Satterthwaite's method ['lmerModLmerTest']
Formula: Average_movement_duration_H2_Zone_1 ~ 1 + (1 | Genotype_Zone_1:Plant_Zone_1) +      (1 |
Genotype_Zone_2:Plant_Zone_2)
   Data: data

REML criterion at convergence: 64.9

Scaled residuals:
     Min       1Q   Median       3Q      Max
-2.37695 -0.43917  0.04724  0.61878  1.75294

Random effects:
 Groups                       Name        Variance Std.Dev.
 Genotype_Zone_1:Plant_Zone_1 (Intercept) 0.00000  0.0000
 Genotype_Zone_2:Plant_Zone_2 (Intercept) 0.09905  0.3147
 Residual                                 0.27221  0.5217
Number of obs: 36, groups:  Genotype_Zone_1:Plant_Zone_1, 10; Genotype_Zone_2:Plant_Zone_2, 10

Fixed effects:
            Estimate Std. Error     df t value Pr(>|t|)
(Intercept)   2.1220     0.1325 8.9941   16.02 6.42e-08 ***
---
Signif. codes:  0 '***' 0.001 '**' 0.01 '*' 0.05 '.' 0.1 ' ' 1
```

Model residuals

Statistic	Value
Sample skewness	-0.8304
Sample excess kurtosis	0.8605
Passed Shapiro Wilk normality test	Yes (p-value = 0.05553 > 0.05)

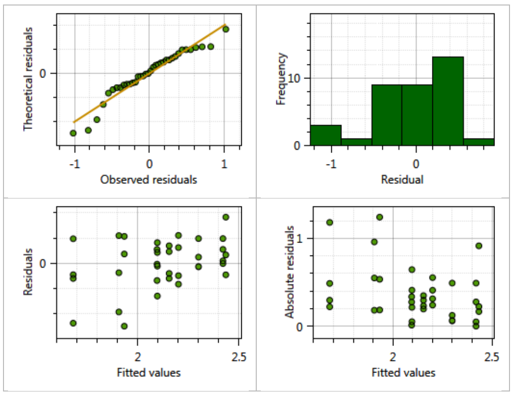

Analysis average movement duration (H2 - Zone 2)

Analysis model	Linear mixed model fit by REML: Average_movement_duration_H2_Zone_2 ~ 1 + (1|Genotype_Zone_1:Plant_Zone_1) + (1|Genotype_Zone_2:Plant_Zone_2)
Transformation	Natural logarithm

Confidence intervals average movement duration (H2 - Zone 2)

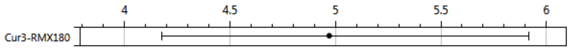

Confidence intervals average movement duration (H2 - Zone 2)

Genotype Zone 1	Genotype Zone 2	Mean	Lower 95% CL	Upper 95% CL	Group
Cur3	RMX180	4.97	4.174	5.918	a

Model summary

```
Linear mixed model fit by REML. t-tests use Satterthwaite's method ['lmerModLmerTest']
Formula: Average_movement_duration_H2_Zone_2 ~ 1 + (1 | Genotype_Zone_1:Plant_Zone_1) +      (1 |
Genotype_Zone_2:Plant_Zone_2)
   Data: data

REML criterion at convergence: 59.2

Scaled residuals:
    Min      1Q  Median      3Q     Max
-4.0394 -0.1730  0.0900  0.4223  2.3222

Random effects:
 Groups                       Name        Variance Std.Dev.
 Genotype_Zone_1:Plant_Zone_1 (Intercept) 0.000    0.0000
 Genotype_Zone_2:Plant_Zone_2 (Intercept) 0.000    0.0000
 Residual                                 0.274    0.5234
Number of obs: 37, groups:  Genotype_Zone_1:Plant_Zone_1, 10; Genotype_Zone_2:Plant_Zone_2, 10

Fixed effects:
            Estimate Std. Error       df t value Pr(>|t|)
(Intercept)  1.60349    0.08605 36.00000   18.63   <2e-16 ***
---
Signif. codes:  0 '***' 0.001 '**' 0.01 '*' 0.05 '.' 0.1 ' ' 1
```

Model residuals

Statistic	Value
Sample skewness	-1.805
Sample excess kurtosis	7.346
Passed Shapiro Wilk normality test	No (p-value = 5.105E-05 < 0.05)

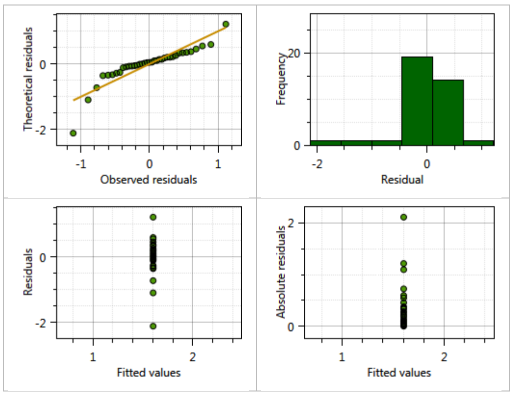

Analysis average movement duration (H3 - Zone 1)

**Analysis model**	Linear mixed model fit by REML: Average_movement_duration_H3_Zone_1 ~ 1 + (1|Genotype_Zone_1:Plant_Zone_1) + (1|Genotype_Zone_2:Plant_Zone_2)
**Transformation**	Natural logarithm

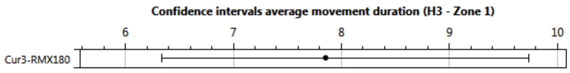

Confidence intervals average movement duration (H3 - Zone 1)

Genotype Zone 1	Genotype Zone 2	Mean	Lower 95% CL	Upper 95% CL	Group
Cur3	RMX180	7.853	6.334	9.737	a

Model summary

```
Linear mixed model fit by REML. t-tests use Satterthwaite's method ['lmerModLmerTest']
Formula: Average_movement_duration_H3_Zone_1 ~ 1 + (1 | Genotype_Zone_1:Plant_Zone_1) +      (1 |
Genotype_Zone_2:Plant_Zone_2)
   Data: data

REML criterion at convergence: 53.8

Scaled residuals:
    Min      1Q  Median      3Q     Max
-2.4075 -0.4994  0.1303  0.7047  1.6897

Random effects:
 Groups                       Name        Variance Std.Dev.
 Genotype_Zone_1:Plant_Zone_1 (Intercept) 0.004185 0.06469
 Genotype_Zone_2:Plant_Zone_2 (Intercept) 0.000000 0.00000
 Residual                                 0.278459 0.52769
Number of obs: 33, groups:  Genotype_Zone_1:Plant_Zone_1, 10; Genotype_Zone_2:Plant_Zone_2, 10

Fixed effects:
            Estimate Std. Error      df t value Pr(>|t|)
(Intercept)  2.06092    0.09423 8.51350   21.87 8.85e-09 ***
---
Signif. codes:  0 '***' 0.001 '**' 0.01 '*' 0.05 '.' 0.1 ' ' 1
```

Model residuals

Statistic	Value
Sample skewness	-0.6615
Sample excess kurtosis	0.2188
Passed Shapiro Wilk normality test	Yes (p-value = 0.237 > 0.05)

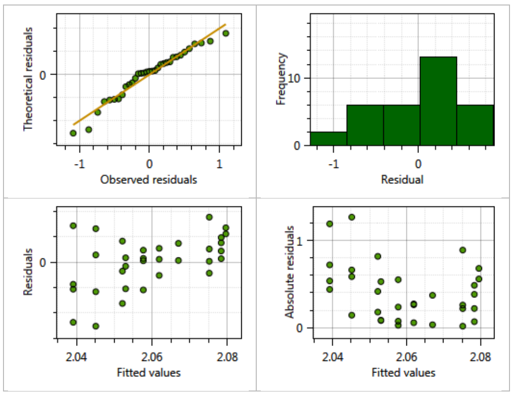

Analysis average movement duration (H3 - Zone 2)

**Analysis model**	Linear mixed model fit by REML: Average_movement_duration_H3_Zone_2 ~ 1 + (1|Genotype_Zone_1:Plant_Zone_1) + (1|Genotype_Zone_2:Plant_Zone_2)
**Transformation**	Natural logarithm

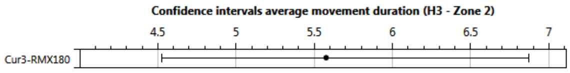

Confidence intervals average movement duration (H3 - Zone 2)

Genotype Zone 1	Genotype Zone 2	Mean	Lower 95% CL	Upper 95% CL	Group
Cur3	RMX180	5.576	4.523	6.873	a

Model summary

```
Linear mixed model fit by REML. t-tests use Satterthwaite's method ['lmerModLmerTest']
Formula: Average_movement_duration_H3_Zone_2 ~ 1 + (1 | Genotype_Zone_1:Plant_Zone_1) +      (1 |
Genotype_Zone_2:Plant_Zone_2)
   Data: data

REML criterion at convergence: 56.6

Scaled residuals:
     Min       1Q   Median       3Q      Max
-2.43814 -0.48926 -0.01966  0.49488  2.92464

Random effects:
 Groups                       Name        Variance Std.Dev.
 Genotype_Zone_1:Plant_Zone_1 (Intercept) 0.00000  0.0000
 Genotype_Zone_2:Plant_Zone_2 (Intercept) 0.03169  0.1780
 Residual                                 0.21168  0.4601
Number of obs: 39, groups:  Genotype_Zone_1:Plant_Zone_1, 10; Genotype_Zone_2:Plant_Zone_2, 10

Fixed effects:
            Estimate Std. Error      df t value Pr(>|t|)
(Intercept)  1.71841    0.09279 9.20240   18.52 1.35e-08 ***
---
Signif. codes:  0 '***' 0.001 '**' 0.01 '*' 0.05 '.' 0.1 ' ' 1
```

Model residuals

Statistic	Value
Sample skewness	0.04414
Sample excess kurtosis	2.082
Passed Shapiro Wilk normality test	Yes (p-value = 0.1537 > 0.05)

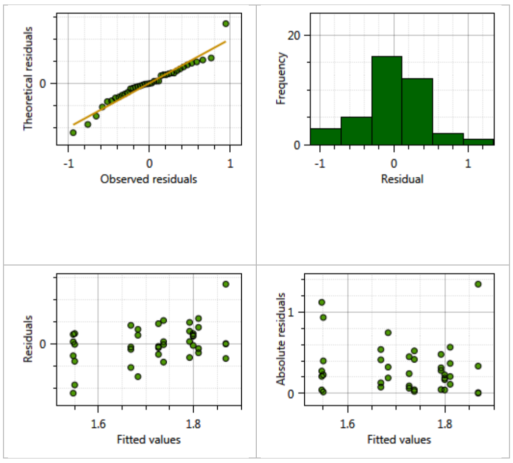

Data points with high residuals

Trial	Arena
Trial 3	26

Analysis average movement duration (H4 - Zone 1)

**Analysis model**	Linear mixed model fit by REML: Average_movement_duration_H4_Zone_1 ~ 1 + (1|Genotype_Zone_1:Plant_Zone_1) + (1|Genotype_Zone_2:Plant_Zone_2)
**Transformation**	Natural logarithm

Confidence intervals average movement duration (H4 - Zone 1)

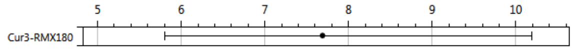

Confidence intervals average movement duration (H4 - Zone 1)

Genotype Zone 1	Genotype Zone 2	Mean	Lower 95% CL	Upper 95% CL	Group
Cur3	RMX180	7.689	5.799	10.19	a

Model summary

```
Linear mixed model fit by REML. t-tests use Satterthwaite's method ['lmerModLmerTest']
Formula: Average_movement_duration_H4_Zone_1 ~ 1 + (1 | Genotype_Zone_1:Plant_Zone_1) +      (1 |
Genotype_Zone_2:Plant_Zone_2)
   Data: data

REML criterion at convergence: 60.3

Scaled residuals:
    Min      1Q  Median      3Q     Max
-2.4092 -0.3312  0.2779  0.7029  1.2621

Random effects:
 Groups                       Name        Variance Std.Dev.
 Genotype_Zone_1:Plant_Zone_1 (Intercept) 0.00000  0.0000
 Genotype_Zone_2:Plant_Zone_2 (Intercept) 0.01058  0.1029
 Residual                                 0.40685  0.6378
Number of obs: 30, groups:  Genotype_Zone_1:Plant_Zone_1, 10; Genotype_Zone_2:Plant_Zone_2, 10

Fixed effects:
            Estimate Std. Error     df t value Pr(>|t|)
(Intercept)   2.0397     0.1212 7.5908   16.83 2.77e-07 ***
---
Signif. codes:  0 '***' 0.001 '**' 0.01 '*' 0.05 '.' 0.1 ' ' 1
```

Model residuals

Statistic	Value
Sample skewness	-1.017
Sample excess kurtosis	0.2583
Passed Shapiro Wilk normality test	No (p-value = 0.009072 < 0.05)

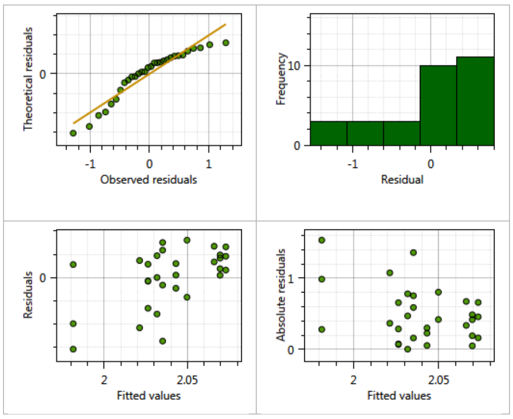

Analysis average movement duration (H4 - Zone 2)

**Analysis model**	Linear mixed model fit by REML: Average_movement_duration_H4_Zone_2 ~ 1 + (1|Genotype_Zone_1:Plant_Zone_1) + (1|Genotype_Zone_2:Plant_Zone_2)
**Transformation**	Natural logarithm

Confidence intervals average movement duration (H4 - Zone 2)

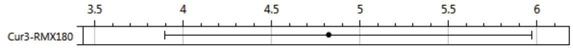

Confidence intervals average movement duration (H4 - Zone 2)

Genotype Zone 1	Genotype Zone 2	Mean	Lower 95% CL	Upper 95% CL	Group
Cur3	RMX180	4.822	3.893	5.972	a

Model summary

```
Linear mixed model fit by REML. t-tests use Satterthwaite's method ['lmerModLmerTest']
Formula: Average_movement_duration_H4_Zone_2 ~ 1 + (1 | Genotype_Zone_1:Plant_Zone_1) +      (1 |
Genotype_Zone_2:Plant_Zone_2)
   Data: data
```

```
REML criterion at convergence: 51.7

Scaled residuals:
     Min       1Q   Median       3Q      Max
-2.28263 -0.38914 -0.06957  0.48030  2.07853

Random effects:
 Groups                      Name        Variance Std.Dev.
 Genotype_Zone_1:Plant_Zone_1 (Intercept) 0.00000  0.0000
 Genotype_Zone_2:Plant_Zone_2 (Intercept) 0.03251  0.1803
 Residual                                0.19803  0.4450
Number of obs: 37, groups:  Genotype_Zone_1:Plant_Zone_1, 10; Genotype_Zone_2:Plant_Zone_2, 10

Fixed effects:
            Estimate Std. Error      df t value Pr(>|t|)
(Intercept)  1.57323    0.09313 8.18312   16.89 1.19e-07 ***
---
Signif. codes:  0 '***' 0.001 '**' 0.01 '*' 0.05 '.' 0.1 ' ' 1
```

Model residuals

Statistic	Value
Sample skewness	-0.1715
Sample excess kurtosis	0.3177
Passed Shapiro Wilk normality test	Yes (p-value = 0.6172 > 0.05)

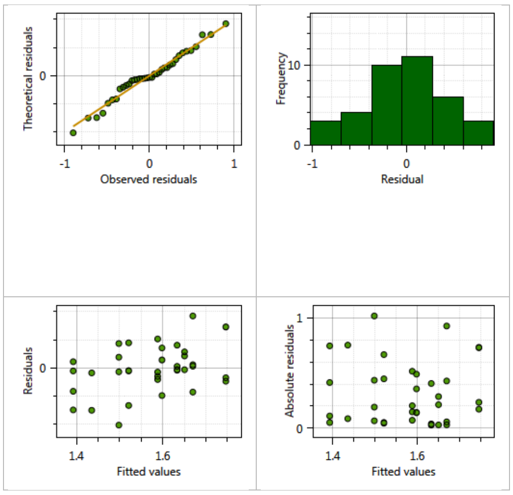

Analysis average movement duration (H5 - Zone 1)

**Analysis model**	Linear mixed model fit by REML: Average_movement_duration_H5_Zone_1 ~ 1 + (1|Genotype_Zone_1:Plant_Zone_1) + (1|Genotype_Zone_2:Plant_Zone_2)
**Transformation**	Natural logarithm

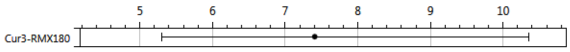

Confidence intervals average movement duration (H5 - Zone 1)

Genotype Zone 1	Genotype Zone 2	Mean	Lower 95% CL	Upper 95% CL	Group
Cur3	RMX180	7.406	5.296	10.36	a

Model summary

```
Linear mixed model fit by REML. t-tests use Satterthwaite's method ['lmerModLmerTest']
Formula: Average_movement_duration_H5_Zone_1 ~ 1 + (1 | Genotype_Zone_1:Plant_Zone_1) +      (1 |
Genotype_Zone_2:Plant_Zone_2)
   Data: data

REML criterion at convergence: 69.8

Scaled residuals:
    Min      1Q  Median      3Q     Max
-3.3014 -0.4290  0.1387  0.5239  1.9390

Random effects:
 Groups                      Name        Variance Std.Dev.
 Genotype_Zone_1:Plant_Zone_1 (Intercept) 0.06795  0.2607
 Genotype_Zone_2:Plant_Zone_2 (Intercept) 0.00000  0.0000
 Residual                                0.48014  0.6929
Number of obs: 31, groups:  Genotype_Zone_1:Plant_Zone_1, 10; Genotype_Zone_2:Plant_Zone_2, 10

Fixed effects:
            Estimate Std. Error     df t value Pr(>|t|)
(Intercept)   2.0023     0.1505 9.9666   13.31 1.14e-07 ***
---
Signif. codes:  0 '***' 0.001 '**' 0.01 '*' 0.05 '.' 0.1 ' ' 1
```

Model residuals

Statistic	Value
Sample skewness	-1.213
Sample excess kurtosis	3.883
Passed Shapiro Wilk normality test	No (p-value = 0.01541 < 0.05)

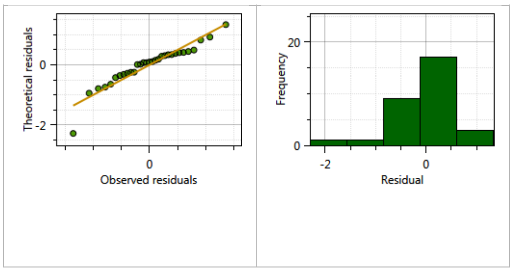

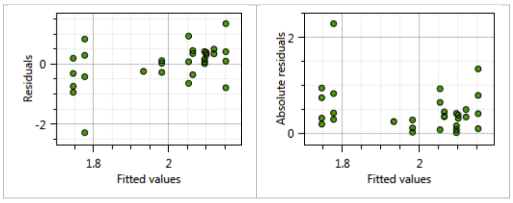

Analysis average movement duration (H5 - Zone 2)

**Analysis model**	Linear mixed model fit by REML: Average_movement_duration_H5_Zone_2 ~ 1 + (1|Genotype_Zone_1:Plant_Zone_1) + (1|Genotype_Zone_2:Plant_Zone_2)
**Transformation**	Natural logarithm

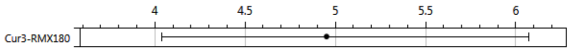

Confidence intervals average movement duration (H5 - Zone 2)

Genotype Zone 1	Genotype Zone 2	Mean	Lower 95% CL	Upper 95% CL	Group
Cur3	RMX180	4.951	4.037	6.073	a

Model summary

```
Linear mixed model fit by REML. t-tests use Satterthwaite's method ['lmerModLmerTest']
Formula: Average_movement_duration_H5_Zone_2 ~ 1 + (1 | Genotype_Zone_1:Plant_Zone_1) +      (1 |
Genotype_Zone_2:Plant_Zone_2)
   Data: data

REML criterion at convergence: 52.8

Scaled residuals:
    Min      1Q  Median      3Q     Max
-2.6244 -0.3874  0.0524  0.5940  1.9198

Random effects:
 Groups                       Name        Variance Std.Dev.
 Genotype_Zone_1:Plant_Zone_1 (Intercept) 0.003813 0.06175
 Genotype_Zone_2:Plant_Zone_2 (Intercept) 0.007120 0.08438
 Residual                                 0.203604 0.45122
Number of obs: 39, groups:  Genotype_Zone_1:Plant_Zone_1, 10; Genotype_Zone_2:Plant_Zone_2, 10

Fixed effects:
            Estimate Std. Error     df t value Pr(>|t|)
(Intercept)   1.5997     0.0795 5.0089   20.12 5.52e-06 ***
---
Signif. codes:  0 '***' 0.001 '**' 0.01 '*' 0.05 '.' 0.1 ' ' 1
```

Model residuals

Statistic	Value
Sample skewness	-0.6648
Sample excess kurtosis	1.261
Passed Shapiro Wilk normality test	No (p-value = 0.02959 < 0.05)

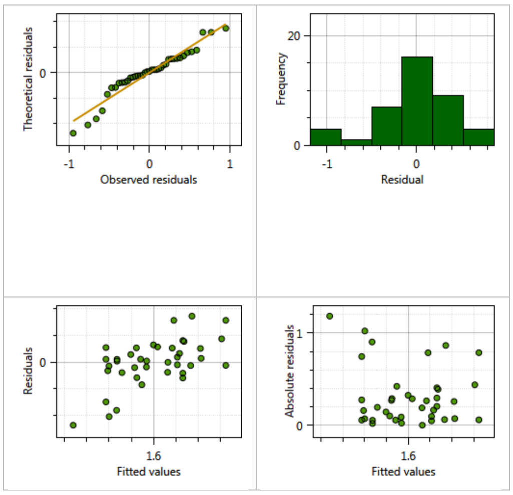

Analysis average movement duration (H6 - Zone 1)

**Analysis model**	Linear mixed model fit by REML: Average_movement_duration_H6_Zone_1 ~ 1 + (1|Genotype_Zone_1:Plant_Zone_1) + (1|Genotype_Zone_2:Plant_Zone_2)
**Transformation**	Natural logarithm

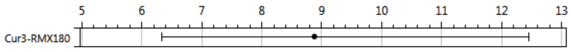

Confidence intervals average movement duration (H6 - Zone 1)

Genotype Zone 1	Genotype Zone 2	Mean	Lower 95% CL	Upper 95% CL	Group
Cur3	RMX180	8.878	6.327	12.46	a

Model summary

```
Linear mixed model fit by REML. t-tests use Satterthwaite's method ['lmerModLmerTest']
Formula: Average_movement_duration_H6_Zone_1 ~ 1 + (1 | Genotype_Zone_1:Plant_Zone_1) +      (1 |
Genotype_Zone_2:Plant_Zone_2)
   Data: data

REML criterion at convergence: 52.6

Scaled residuals:
    Min      1Q  Median      3Q     Max
-2.1390 -0.2929  0.1075  0.6883  1.1694

Random effects:
 Groups                       Name        Variance Std.Dev.
 Genotype_Zone_1:Plant_Zone_1 (Intercept) 0.0000   0.0000
 Genotype_Zone_2:Plant_Zone_2 (Intercept) 0.0839   0.2897
 Residual                                 0.3280   0.5727
Number of obs: 27, groups:  Genotype_Zone_1:Plant_Zone_1, 10; Genotype_Zone_2:Plant_Zone_2, 10

Fixed effects:
            Estimate Std. Error     df t value Pr(>|t|)
(Intercept)   2.1835     0.1466 7.9148   14.89 4.53e-07 ***
```

```
---
Signif. codes:  0 '***' 0.001 '**' 0.01 '*' 0.05 '.' 0.1 ' ' 1
```

Model residuals

Statistic	Value
Sample skewness	-1.029
Sample excess kurtosis	0.325
Passed Shapiro Wilk normality test	No (p-value = 0.00886 < 0.05)

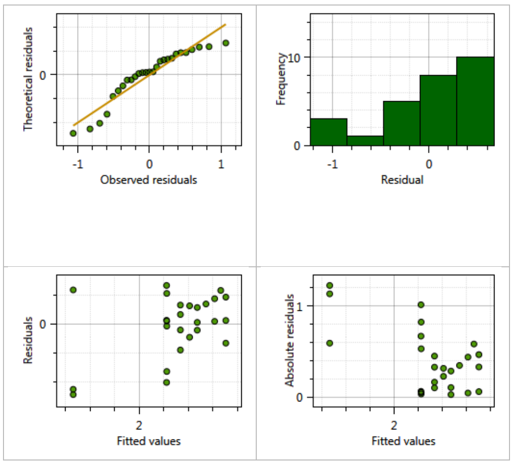

Analysis average movement duration (H6 - Zone 2)

**Analysis model**	Linear mixed model fit by REML: Average_movement_duration_H6_Zone_2 ~ 1 + (1|Genotype_Zone_1:Plant_Zone_1) + (1|Genotype_Zone_2:Plant_Zone_2)
**Transformation**	Natural logarithm

Confidence intervals average movement duration (H6 - Zone 2)

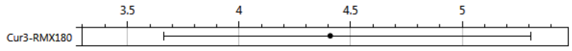

Confidence intervals average movement duration (H6 - Zone 2)

Genotype Zone 1	Genotype Zone 2	Mean	Lower 95% CL	Upper 95% CL	Group
Cur3	RMX180	4.408	3.661	5.309	a

Model summary

```
Linear mixed model fit by REML. t-tests use Satterthwaite's method ['lmerModLmerTest']
Formula: Average_movement_duration_H6_Zone_2 ~ 1 + (1 | Genotype_Zone_1:Plant_Zone_1) +      (1 |
Genotype_Zone_2:Plant_Zone_2)
   Data: data

REML criterion at convergence: 51.4

Scaled residuals:
    Min      1Q  Median      3Q     Max
-2.7340 -0.3644  0.1449  0.7350  1.5292

Random effects:
 Groups                       Name        Variance  Std.Dev.
 Genotype_Zone_1:Plant_Zone_1 (Intercept) 1.491e-02 1.221e-01
 Genotype_Zone_2:Plant_Zone_2 (Intercept) 3.176e-17 5.636e-09
 Residual                                 2.005e-01 4.478e-01
Number of obs: 38, groups:  Genotype_Zone_1:Plant_Zone_1, 10; Genotype_Zone_2:Plant_Zone_2, 10

Fixed effects:
            Estimate Std. Error      df t value Pr(>|t|)
(Intercept)  1.48349    0.08234 9.13848   18.02 1.88e-08 ***
---
Signif. codes:  0 '***' 0.001 '**' 0.01 '*' 0.05 '.' 0.1 ' ' 1
```

Model residuals

Statistic	Value
Sample skewness	-1.022
Sample excess kurtosis	0.941
Passed Shapiro Wilk normality test	No (p-value = 0.01887 < 0.05)

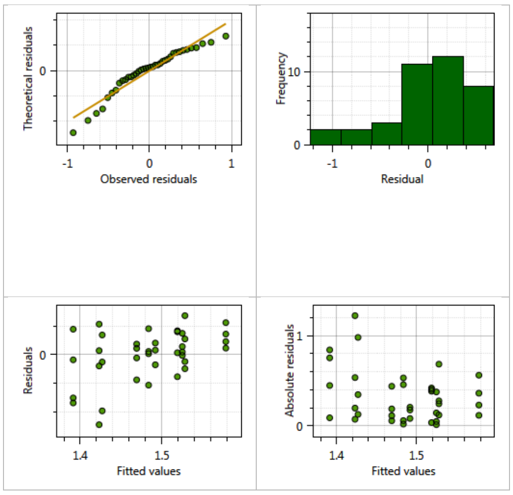

Analysis average movement duration (H7 - Zone 1)

**Analysis model**	Linear mixed model fit by REML: Average_movement_duration_H7_Zone_1 ~ 1 + (1|Genotype_Zone_1:Plant_Zone_1) + (1|Genotype_Zone_2:Plant_Zone_2)
**Transformation**	Natural logarithm

Confidence intervals average movement duration (H7 - Zone 1)

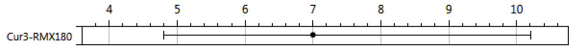

Confidence intervals average movement duration (H7 - Zone 1)

Genotype Zone 1	Genotype Zone 2	Mean	Lower 95% CL	Upper 95% CL	Group
Cur3	RMX180	6.998	4.796	10.21	a

Model summary

```
Linear mixed model fit by REML. t-tests use Satterthwaite's method ['lmerModLmerTest']
Formula: Average_movement_duration_H7_Zone_1 ~ 1 + (1 | Genotype_Zone_1:Plant_Zone_1) +      (1 |
Genotype_Zone_2:Plant_Zone_2)
   Data: data

REML criterion at convergence: 67.2

Scaled residuals:
    Min      1Q  Median      3Q     Max
-3.0856 -0.1677  0.1866  0.6752  0.9303

Random effects:
 Groups                       Name        Variance Std.Dev.
 Genotype_Zone_2:Plant_Zone_2 (Intercept) 0.04877  0.2208
 Genotype_Zone_1:Plant_Zone_1 (Intercept) 0.00000  0.0000
 Residual                                 0.58270  0.7633
Number of obs: 28, groups:  Genotype_Zone_2:Plant_Zone_2, 10; Genotype_Zone_1:Plant_Zone_1, 9

Fixed effects:
            Estimate Std. Error     df t value Pr(>|t|)
(Intercept)   1.9457     0.1622 7.5679   11.99 3.41e-06 ***
---
Signif. codes:  0 '***' 0.001 '**' 0.01 '*' 0.05 '.' 0.1 ' ' 1
```

Model residuals

Statistic	Value
Sample skewness	-1.959
Sample excess kurtosis	3.958
Passed Shapiro Wilk normality test	No (p-value = 5.029E-05 < 0.05)

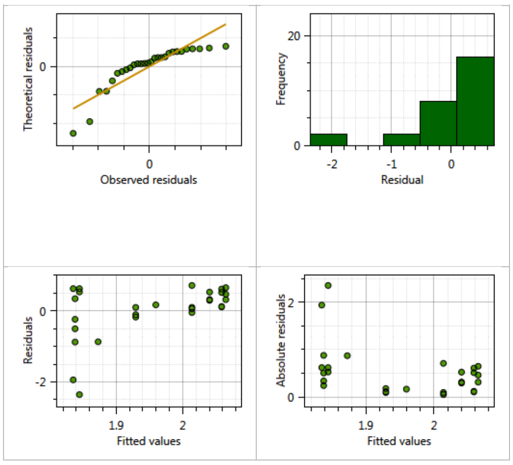

Analysis average movement duration (H7 - Zone 2)

**Analysis model**	Linear mixed model fit by REML: Average_movement_duration_H7_Zone_2 ~ 1 + (1|Genotype_Zone_1:Plant_Zone_1) + (1|Genotype_Zone_2:Plant_Zone_2)
**Transformation**	Natural logarithm

Confidence intervals average movement duration (H7 - Zone 2)

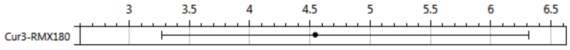

Confidence intervals average movement duration (H7 - Zone 2)

Genotype Zone 1	Genotype Zone 2	Mean	Lower 95% CL	Upper 95% CL	Group
Cur3	RMX180	4.544	3.268	6.318	a

Model summary

```
Linear mixed model fit by REML. t-tests use Satterthwaite's method ['lmerModLmerTest']
Formula: Average_movement_duration_H7_Zone_2 ~ 1 + (1 | Genotype_Zone_1:Plant_Zone_1) +      (1 |
Genotype_Zone_2:Plant_Zone_2)
   Data: data

REML criterion at convergence: 56.9

Scaled residuals:
     Min       1Q   Median       3Q      Max
-2.05501 -0.36560  0.09657  0.51678  1.75502

Random effects:
 Groups                       Name        Variance Std.Dev.
 Genotype_Zone_1:Plant_Zone_1 (Intercept) 0.12344  0.3513
 Genotype_Zone_2:Plant_Zone_2 (Intercept) 0.05984  0.2446
 Residual                                 0.13721  0.3704
Number of obs: 39, groups:  Genotype_Zone_1:Plant_Zone_1, 10; Genotype_Zone_2:Plant_Zone_2, 10

Fixed effects:
            Estimate Std. Error      df t value Pr(>|t|)
(Intercept)   1.5138     0.1479 10.0151   10.23 1.27e-06 ***
---
Signif. codes:  0 '***' 0.001 '**' 0.01 '*' 0.05 '.' 0.1 ' ' 1
```

Model residuals

Statistic	Value
Sample skewness	-0.2549
Sample excess kurtosis	0.1397
Passed Shapiro Wilk normality test	Yes (p-value = 0.9078 > 0.05)

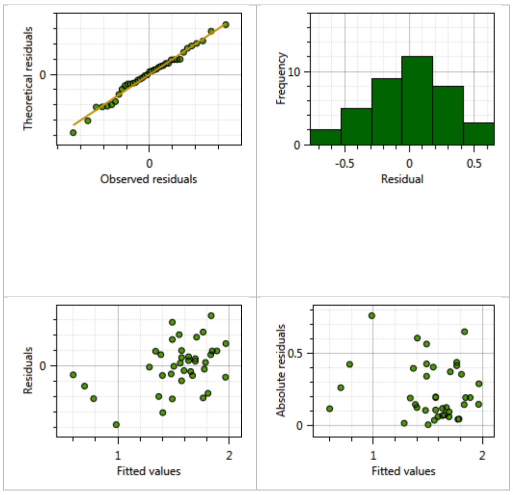

Analysis average movement duration H0 (diff. Zone 1 - Zone 2)

**Analysis model**	Generalized linear mixed model with dispersion factor, formula=cbind(Average_movement_duration_H0_Zone_1,Average_movement_duration_H0_Zone_2) ~ 1 + (1|Genotype_Zone_1:Plant_Zone_1) + (1|Genotype_Zone_2:Plant_Zone_2)
**Transformation**	Logit

Confidence intervals average movement duration H0 (diff. Zone 1 - Zone 2)

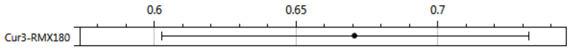

Confidence intervals average movement duration H0 (diff. Zone 1 - Zone 2)

Genotype Zone 1	Genotype Zone 2	Mean	Lower 95% CL	Upper 95% CL	Group
Cur3	RMX180	0.6706	0.6024	0.7322	a

Model summary

```
Linear mixed model fit by REML. t-tests use Satterthwaite's method ['lmerModLmerTest']
Formula: ziFormula
   Data: data
Weights: wi

REML criterion at convergence: 54.7

Scaled residuals: 
     Min       1Q   Median       3Q      Max 
-2.58825 -0.45201  0.05375  0.56104  1.74825 

Random effects:
 Groups                      Name        Variance Std.Dev.
 Genotype_Zone_1:Plant_Zone_1 (Intercept) 0.02322  0.1524  
 Genotype_Zone_2:Plant_Zone_2 (Intercept) 0.04864  0.2205  
 Residual                                 1.15077  1.0727  
Number of obs: 32, groups:  Genotype_Zone_1:Plant_Zone_1, 10; Genotype_Zone_2:Plant_Zone_2, 10

Fixed effects:
            Estimate Std. Error     df t value Pr(>|t|)    
(Intercept)   0.7107     0.1227 6.4610   5.793 0.000892 ***
---
Signif. codes:  0 '***' 0.001 '**' 0.01 '*' 0.05 '.' 0.1 ' ' 1

Dispersion: 1.073
```

Model residuals

Statistic	Value
Sample skewness	-0.6083
Sample excess kurtosis	0.9254
Passed Shapiro Wilk normality test	Yes (p-value = 0.565 > 0.05)

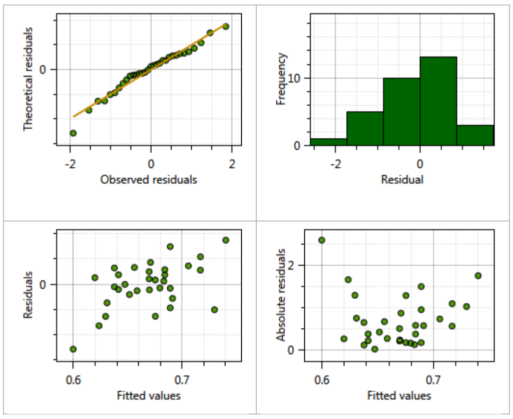

Analysis average movement duration H1 (diff. Zone 1 - Zone 2)

**Analysis model**	Generalized linear mixed model with dispersion factor, formula=cbind(Average_movement_duration_H1_Zone_1,Average_movement_duration_H1_Zone_2) ~ 1 + (1|Genotype_Zone_1:Plant_Zone_1) + (1|Genotype_Zone_2:Plant_Zone_2)
**Transformation**	Logit

Confidence intervals average movement duration H1 (diff. Zone 1 - Zone 2)

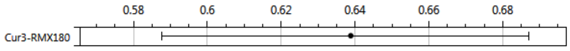

Confidence intervals average movement duration H1 (diff. Zone 1 - Zone 2)

Genotype Zone 1	Genotype Zone 2	Mean	Lower 95% CL	Upper 95% CL	Group
Cur3	RMX180	0.6388	0.5875	0.6871	a

Model summary

```
Linear mixed model fit by REML. t-tests use Satterthwaite's method ['lmerModLmerTest']
Formula: ziFormula
   Data: data
```

```
Weights: wi

REML criterion at convergence: 54.8

Scaled residuals:
     Min       1Q   Median       3Q      Max
-1.93435 -0.56250  0.03949  0.47558  2.69152

Random effects:
 Groups                      Name        Variance Std.Dev.
 Genotype_Zone_1:Plant_Zone_1 (Intercept) 0.0000   0.0000
 Genotype_Zone_2:Plant_Zone_2 (Intercept) 0.0356   0.1887
 Residual                                0.8479   0.9208
Number of obs: 36, groups:  Genotype_Zone_1:Plant_Zone_1, 10; Genotype_Zone_2:Plant_Zone_2, 10

Fixed effects:
            Estimate Std. Error       df t value Pr(>|t|)
(Intercept)  0.57029    0.09813 10.80963   5.812 0.000126 ***
---
Signif. codes:  0 '***' 0.001 '**' 0.01 '*' 0.05 '.' 0.1 ' ' 1

Dispersion: 0.9208
```

Model residuals

Statistic	Value
Sample skewness	0.1141
Sample excess kurtosis	0.9819
Passed Shapiro Wilk normality test	Yes (p-value = 0.175 > 0.05)

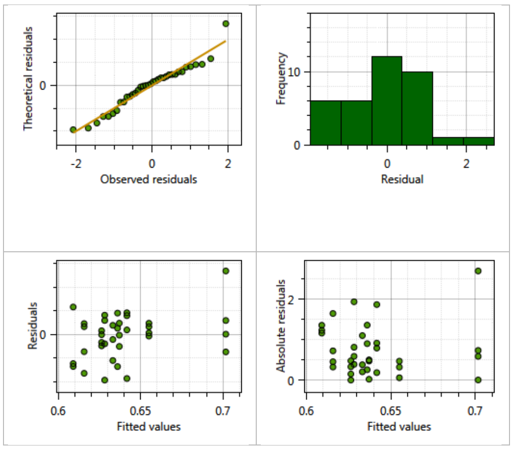

Analysis average movement duration H2 (diff. Zone 1 - Zone 2)

**Analysis model**	Generalized linear mixed model with dispersion factor, formula=cbind(Average_movement_duration_H2_Zone_1,Average_movement_duration_H2_Zone_2) ~ 1 + (1|Genotype_Zone_1:Plant_Zone_1) + (1|Genotype_Zone_2:Plant_Zone_2)
**Transformation**	Logit

Confidence intervals average movement duration H2 (diff. Zone 1 - Zone 2)

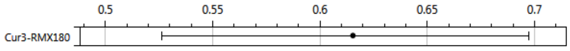

Confidence intervals average movement duration H2 (diff. Zone 1 - Zone 2)

Genotype Zone 1	Genotype Zone 2	Mean	Lower 95% CL	Upper 95% CL	Group
Cur3	RMX180	0.6153	0.5261	0.6974	a

Model summary

```
Linear mixed model fit by REML. t-tests use Satterthwaite's method ['lmerModLmerTest']
Formula: ziFormula
   Data: data
Weights: wi

REML criterion at convergence: 56.6

Scaled residuals:
    Min      1Q  Median      3Q     Max
-2.1096 -0.6887 -0.1613  0.5369  1.8817

Random effects:
 Groups                      Name        Variance Std.Dev.
 Genotype_Zone_1:Plant_Zone_1 (Intercept) 0.13771  0.3711
 Genotype_Zone_2:Plant_Zone_2 (Intercept) 0.06699  0.2588
 Residual                                0.57225  0.7565
Number of obs: 34, groups:  Genotype_Zone_1:Plant_Zone_1, 10; Genotype_Zone_2:Plant_Zone_2, 10

Fixed effects:
            Estimate Std. Error     df t value Pr(>|t|)
(Intercept)   0.4697     0.1598 8.4067    2.94   0.0177 *
---
Signif. codes:  0 '***' 0.001 '**' 0.01 '*' 0.05 '.' 0.1 ' ' 1

Dispersion: 0.7565
```

Model residuals

Statistic	Value
Sample skewness	0.0691
Sample excess kurtosis	0.2271
Passed Shapiro Wilk normality test	Yes (p-value = 0.9254 > 0.05)

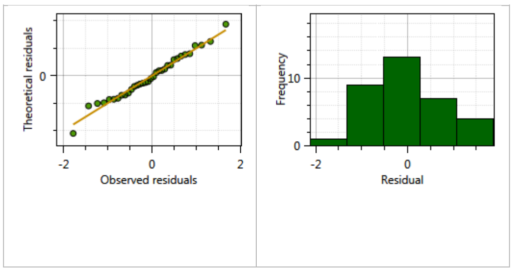

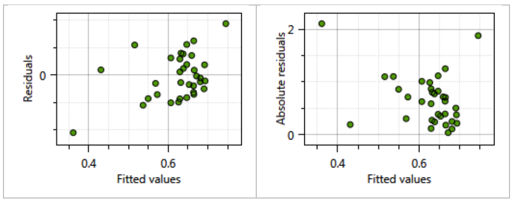

Analysis average movement duration H3 (diff. Zone 1 - Zone 2)

**Analysis model**	Generalized linear mixed model with dispersion factor, formula=cbind(Average_movement_duration_H3_Zone_1,Average_movement_duration_H3_Zone_2) ~ 1 + (1|Genotype_Zone_1:Plant_Zone_1) + (1|Genotype_Zone_2:Plant_Zone_2)
**Transformation**	Logit

Confidence intervals average movement duration H3 (diff. Zone 1 - Zone 2)

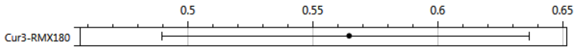

Confidence intervals average movement duration H3 (diff. Zone 1 - Zone 2)

Genotype Zone 1	Genotype Zone 2	Mean	Lower 95% CL	Upper 95% CL	Group
Cur3	RMX180	0.5645	0.4895	0.6368	a

Model summary

```
Linear mixed model fit by REML. t-tests use Satterthwaite's method ['lmerModLmerTest']
Formula: ziFormula
   Data: data
Weights: wi

REML criterion at convergence: 61.3

Scaled residuals:
    Min      1Q  Median      3Q     Max
-3.0961 -0.6862  0.1729  0.5624  1.3294

Random effects:
 Groups                        Name        Variance Std.Dev.
 Genotype_Zone_1:Plant_Zone_1 (Intercept) 0.05245  0.2290
 Genotype_Zone_2:Plant_Zone_2 (Intercept) 0.03165  0.1779
 Residual                                 1.02464  1.0122
Number of obs: 33, groups:  Genotype_Zone_1:Plant_Zone_1, 10; Genotype_Zone_2:Plant_Zone_2, 10

Fixed effects:
            Estimate Std. Error     df t value Pr(>|t|)
(Intercept)   0.2596     0.1296 7.5869   2.003    0.082 .
---
Signif. codes:  0 '***' 0.001 '**' 0.01 '*' 0.05 '.' 0.1 ' ' 1

Dispersion: 1.012
```

Model residuals

Statistic	Value
Sample skewness	-1.122
Sample excess kurtosis	2.532
Passed Shapiro Wilk normality test	No (p-value = 0.0246 < 0.05)

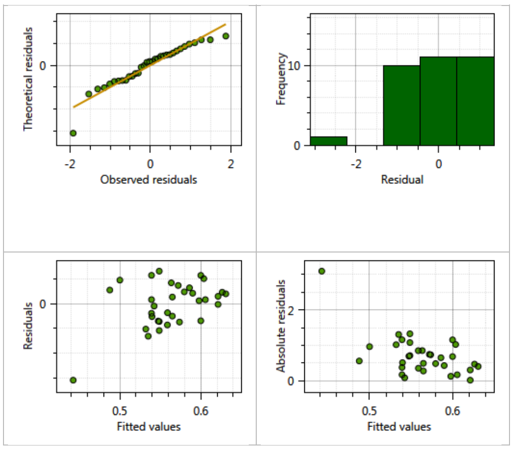

Analysis average movement duration H4 (diff. Zone 1 - Zone 2)

**Analysis model**	Generalized linear mixed model with dispersion factor, formula=cbind(Average_movement_duration_H4_Zone_1,Average_movement_duration_H4_Zone_2) ~ 1 + (1|Genotype_Zone_1:Plant_Zone_1) + (1|Genotype_Zone_2:Plant_Zone_2)
**Transformation**	Logit

Confidence intervals average movement duration H4 (diff. Zone 1 - Zone 2)

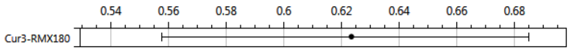

Confidence intervals average movement duration H4 (diff. Zone 1 - Zone 2)

Genotype Zone 1	Genotype Zone 2	Mean	Lower 95% CL	Upper 95% CL	Group
Cur3	RMX180	0.6234	0.5575	0.685	a

Model summary

```
Linear mixed model fit by REML. t-tests use Satterthwaite's method ['lmerModLmerTest']
Formula: ziFormula
   Data: data
Weights: wi

REML criterion at convergence: 43.5

Scaled residuals:
    Min      1Q  Median      3Q     Max
-2.2587 -0.6971 -0.0515  0.6876  1.3251

Random effects:
 Groups                        Name        Variance Std.Dev.
 Genotype_Zone_1:Plant_Zone_1 (Intercept) 0.01023  0.1012
 Genotype_Zone_2:Plant_Zone_2 (Intercept) 0.04806  0.2192
 Residual                                 0.70061  0.8370
Number of obs: 28, groups:  Genotype_Zone_1:Plant_Zone_1, 10; Genotype_Zone_2:Plant_Zone_2, 10

Fixed effects:
            Estimate Std. Error     df t value Pr(>|t|)
```

```
(Intercept)   0.5040     0.1154 6.9751   4.369  0.00331 **
---
Signif. codes:  0 '***' 0.001 '**' 0.01 '*' 0.05 '.' 0.1 ' ' 1

Dispersion: 0.837
```

Model residuals

Statistic	Value
Sample skewness	-0.6245
Sample excess kurtosis	-0.04019
Passed Shapiro Wilk normality test	Yes (p-value = 0.2488 > 0.05)

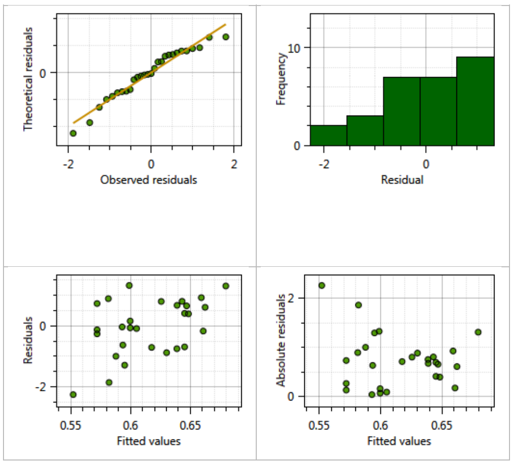

Analysis average movement duration H5 (diff. Zone 1 - Zone 2)

Analysis model	Generalized linear mixed model with dispersion factor, formula=cbind(Average_movement_duration_H5_Zone_1,Average_movement_duration_H5_Zone_2) ~ 1 + (1|Genotype_Zone_1:Plant_Zone_1) + (1|Genotype_Zone_2:Plant_Zone_2)
Transformation	Logit

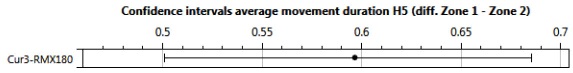

Confidence intervals average movement duration H5 (diff. Zone 1 - Zone 2)

Genotype Zone 1	Genotype Zone 2	Mean	Lower 95% CL	Upper 95% CL	Group
Cur3	RMX180	0.5965	0.5007	0.6855	a

Model summary

```
Linear mixed model fit by REML. t-tests use Satterthwaite's method ['lmerModLmerTest']
Formula: ziFormula
   Data: data
Weights: wi

REML criterion at convergence: 65.4

Scaled residuals:
     Min       1Q   Median       3Q      Max
-1.53472 -0.58918  0.04135  0.41041  2.31526

Random effects:
 Groups                      Name        Variance Std.Dev.
 Genotype_Zone_1:Plant_Zone_1 (Intercept) 0.07423  0.2725
 Genotype_Zone_2:Plant_Zone_2 (Intercept) 0.06207  0.2491
 Residual                                1.11148  1.0543
Number of obs: 31, groups:  Genotype_Zone_1:Plant_Zone_1, 10; Genotype_Zone_2:Plant_Zone_2, 10

Fixed effects:
            Estimate Std. Error     df t value Pr(>|t|)
(Intercept)   0.3910     0.1571 5.7610   2.489   0.0488 *
---
Signif. codes:  0 '***' 0.001 '**' 0.01 '*' 0.05 '.' 0.1 ' ' 1

Dispersion: 1.054
```

Model residuals

Statistic	Value
Sample skewness	0.5062
Sample excess kurtosis	0.5888
Passed Shapiro Wilk normality test	Yes (p-value = 0.4223 > 0.05)

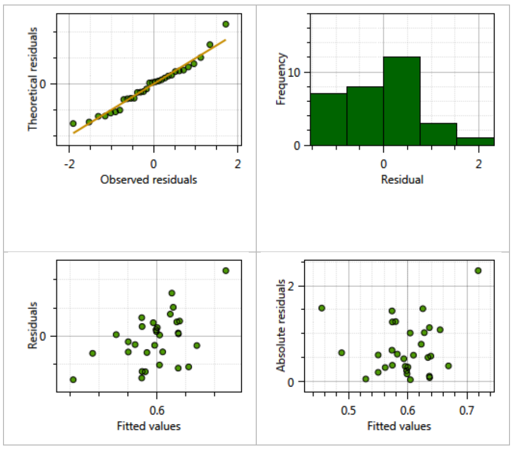

Analysis average movement duration H6 (diff. Zone 1 - Zone 2)

Analysis model	Generalized linear mixed model with dispersion factor, formula=cbind(Average_movement_duration_H6_Zone_1,Average_movement_duration_H6_Zone_2) ~ 1 + (1|Genotype_Zone_1:Plant_Zone_1) + (1|Genotype_Zone_2:Plant_Zone_2)
Transformation	Logit

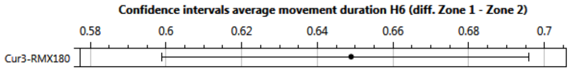

Confidence intervals average movement duration H6 (diff. Zone 1 - Zone 2)

Genotype Zone 1	Genotype Zone 2	Mean	Lower 95% CL	Upper 95% CL	Group
Cur3	RMX180	0.6489	0.5988	0.6959	a

Model summary

```
Linear mixed model fit by REML. t-tests use Satterthwaite's method ['lmerModLmerTest']
Formula: ziFormula
   Data: data
Weights: wi

REML criterion at convergence: 43.6

Scaled residuals:
    Min      1Q  Median      3Q     Max
-2.8100 -0.8525 -0.2171  0.6705  1.4620

Random effects:
 Groups                        Name        Variance Std.Dev.
 Genotype_Zone_1:Plant_Zone_1 (Intercept) 0.000    0.000
 Genotype_Zone_2:Plant_Zone_2 (Intercept) 0.000    0.000
 Residual                                 1.026    1.013
Number of obs: 26, groups:  Genotype_Zone_1:Plant_Zone_1, 10; Genotype_Zone_2:Plant_Zone_2, 10

Fixed effects:
            Estimate Std. Error      df t value Pr(>|t|)
(Intercept)   0.6142     0.1038 25.0000   5.917 3.56e-06 ***
---
Signif. codes:  0 '***' 0.001 '**' 0.01 '*' 0.05 '.' 0.1 ' ' 1

Dispersion: 1.013
```

Model residuals

Statistic	Value
Sample skewness	-0.6151
Sample excess kurtosis	0.5375
Passed Shapiro Wilk normality test	Yes (p-value = 0.2301 > 0.05)

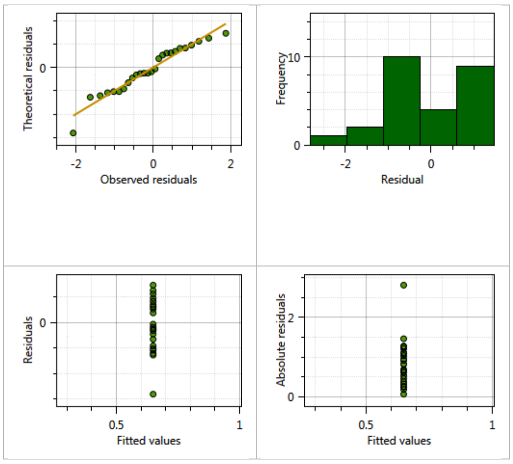

Analysis average movement duration H7 (diff. Zone 1 - Zone 2)

**Analysis model**	Generalized linear mixed model with dispersion factor, formula=cbind(Average_movement_duration_H7_Zone_1,Average_movement_duration_H7_Zone_2) ~ 1 + (1|Genotype_Zone_1:Plant_Zone_1) + (1|Genotype_Zone_2:Plant_Zone_2)
**Transformation**	Logit

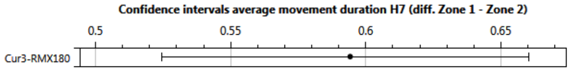

Confidence intervals average movement duration H7 (diff. Zone 1 - Zone 2)

Genotype Zone 1	Genotype Zone 2	Mean	Lower 95% CL	Upper 95% CL	Group
Cur3	RMX180	0.5942	0.5244	0.6604	a

Model summary

```
Linear mixed model fit by REML. t-tests use Satterthwaite's method ['lmerModLmerTest']
Formula: ziFormula
   Data: data
Weights: wi

REML criterion at convergence: 51.8

Scaled residuals:
     Min       1Q   Median       3Q      Max
-2.03475 -0.75653  0.06589  0.62285  1.33171

Random effects:
 Groups                        Name        Variance Std.Dev.
 Genotype_Zone_2:Plant_Zone_2 (Intercept) 0.03561  0.1887
 Genotype_Zone_1:Plant_Zone_1 (Intercept) 0.00000  0.0000
 Residual                                 1.03753  1.0186
Number of obs: 28, groups:  Genotype_Zone_2:Plant_Zone_2, 10; Genotype_Zone_1:Plant_Zone_1, 9

Fixed effects:
            Estimate Std. Error     df t value Pr(>|t|)
(Intercept)   0.3814     0.1223 7.7119    3.12   0.0149 *
---
Signif. codes:  0 '***' 0.001 '**' 0.01 '*' 0.05 '.' 0.1 ' ' 1

Dispersion: 1.019
```

Model residuals

Statistic	Value
Sample skewness	-0.6426
Sample excess kurtosis	-0.3784
Passed Shapiro Wilk normality test	Yes (p-value = 0.08009 > 0.05)

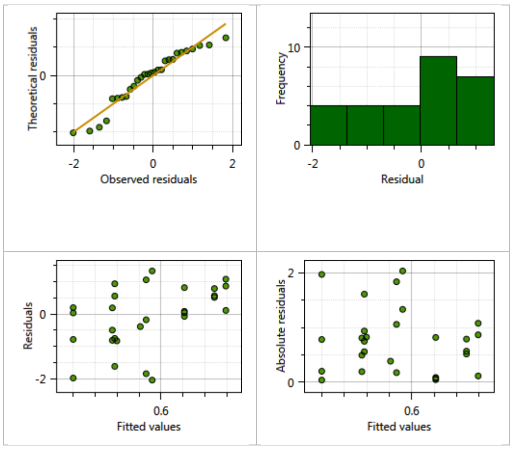

Average movement duration per zone per short/medium/long

Selected zones	Zone 1, Zone 2
Event duration categories	duration < 2, 2 <= duration < 5, duration >= 5
Data transformation	Natural logarithm
Analysis	Zone difference analysis

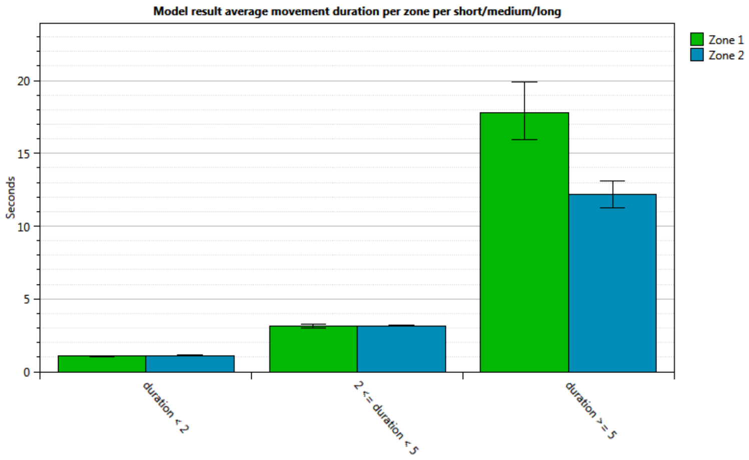

Results difference tests Zone 1 - Zone 2: p values and 95% confidence intervals of the difference on the transformed scale for each statistic.

Behaviour statistic	Cur3-RMX180	Remark
Average movement duration duration < 2 (diff. Zone 1 - Zone 2)	p=0.07<br>[-0.0612, 0.00252]	CR
Average movement duration 2 <= duration < 5 (diff. Zone 1 - Zone 2)	p=0.497<br>[-0.0299, 0.0148]	CR
Average movement duration duration >= 5 (diff. Zone 1 - Zone 2)	p=4.86E-11****<br>[0.286, 0.446]	CR

CR = Check residuals

The model predictions and 95% confidence intervals for each statistic.

Statistic	Cur3-RMX180	Remark
Average movement duration (duration < 2 - Zone 1)	1.08<br>[1.05, 1.12]	CR
Average movement duration (2 <= duration < 5 - Zone 1)	3.14<br>[3.03, 3.25]	CR
Average movement duration (duration >= 5 - Zone 1)	17.8<br>[15.9, 19.9]	CR
Average movement duration (duration < 2 - Zone 2)	1.12<br>[1.11, 1.13]	CR
Average movement duration (2 <= duration < 5 - Zone 2)	3.17<br>[3.12, 3.21]	CR
Average movement duration (duration >= 5 - Zone 2)	12.2<br>[11.3, 13.1]	CR

CR = Check residuals

Data summary

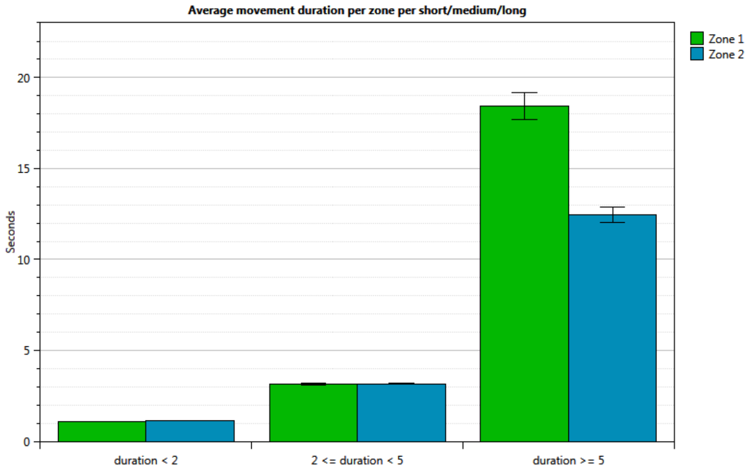

Genotype Zone 1	Genotype Zone 2	Genotype Zone 3	Mean duration < 2 - Zone 1	StdErr duration < 2 - Zone 1	Mean 2 <= duration < 5 - Zone 1	StdErr 2 <= duration < 5 - Zone 1	Mean duration >= 5 - Zone 1	StdErr duration >= 5 - Zone 1	Mean duration < 2 - Zone 2	StdErr duration < 2 - Zone 2	Mean 2 <= duration < 5 - Zone 2	StdErr 2 <= duration < 5 - Zone 2	Mean duration >= 5 - Zone 2	StdErr duration >= 5 - Zone 2
Cur3	RMX180	Neutral	1.09	0.01	3.15	0.04	18.43	0.74	1.12	0.01	3.17	0.01	12.47	0.44

Analysis average movement duration (duration < 2 - Zone 1)

**Analysis model**	Linear mixed model fit by REML: Average_movement_duration_duration_2_Zone_1 ~ 1 + (1|Genotype_Zone_1:Plant_Zone_1) + (1|Genotype_Zone_2:Plant_Zone_2)
**Transformation**	Natural logarithm

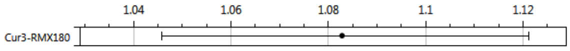

Confidence intervals average movement duration (duration < 2 - Zone 1)

Genotype Zone 1	Genotype Zone 2	Mean	Lower 95% CL	Upper 95% CL	Group
Cur3	RMX180	1.083	1.046	1.121	a

Model summary

```
Linear mixed model fit by REML. t-tests use Satterthwaite's method ['lmerModLmerTest']
Formula: Average_movement_duration_duration_2_Zone_1 ~ 1 + (1 | Genotype_Zone_1:Plant_Zone_1) +      (1 |
Genotype_Zone_2:Plant_Zone_2)
   Data: data

REML criterion at convergence: -57.4

Scaled residuals:
    Min      1Q  Median      3Q     Max
-5.5648 -0.1121  0.1486  0.4022  1.0470

Random effects:
 Groups                       Name        Variance Std.Dev.
 Genotype_Zone_1:Plant_Zone_1 (Intercept) 0.00000  0.0000
 Genotype_Zone_2:Plant_Zone_2 (Intercept) 0.00000  0.0000
 Residual                                 0.01126  0.1061
Number of obs: 38, groups:  Genotype_Zone_1:Plant_Zone_1, 10; Genotype_Zone_2:Plant_Zone_2, 10

Fixed effects:
            Estimate Std. Error       df t value Pr(>|t|)
(Intercept)  0.07955    0.01721 37.00000   4.622  4.5e-05 ***
---
Signif. codes:  0 '***' 0.001 '**' 0.01 '*' 0.05 '.' 0.1 ' ' 1
```

Model residuals

Statistic	Value
Sample skewness	-4.853
Sample excess kurtosis	27.3
Passed Shapiro Wilk normality test	No (p-value = 2.652E-10 < 0.05)

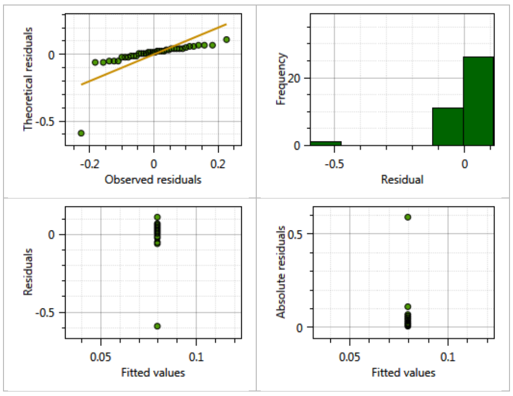

Analysis average movement duration (2 <= duration < 5 - Zone 1)

**Analysis model**	Linear mixed model fit by REML: Average_movement_duration_2_duration_5_Zone_1 ~ 1 + (1|Genotype_Zone_1:Plant_Zone_1) + (1|Genotype_Zone_2:Plant_Zone_2)
**Transformation**	Natural logarithm

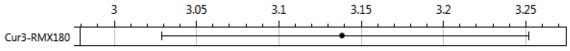

Confidence intervals average movement duration (2 <= duration < 5 - Zone 1)

Genotype Zone 1	Genotype Zone 2	Mean	Lower 95% CL	Upper 95% CL	Group
Cur3	RMX180	3.138	3.029	3.252	a

Model summary

```
Linear mixed model fit by REML. t-tests use Satterthwaite's method ['lmerModLmerTest']
Formula: Average_movement_duration_2_duration_5_Zone_1 ~ 1 + (1 | Genotype_Zone_1:Plant_Zone_1) +      (1
| Genotype_Zone_2:Plant_Zone_2)
   Data: data

REML criterion at convergence: -80.8

Scaled residuals:
    Min      1Q  Median      3Q     Max
-4.8937 -0.2465  0.1146  0.4597  1.1135

Random effects:
 Groups                       Name        Variance  Std.Dev.
 Genotype_Zone_1:Plant_Zone_1 (Intercept) 9.157e-04 3.026e-02
 Genotype_Zone_2:Plant_Zone_2 (Intercept) 7.527e-18 2.743e-09
 Residual                                 5.279e-03 7.265e-02
Number of obs: 38, groups:  Genotype_Zone_1:Plant_Zone_1, 10; Genotype_Zone_2:Plant_Zone_2, 10

Fixed effects:
            Estimate Std. Error     df t value Pr(>|t|)
(Intercept)   1.1437     0.0152 7.3862   75.23  6.3e-12 ***
---
Signif. codes:  0 '***' 0.001 '**' 0.01 '*' 0.05 '.' 0.1 ' ' 1
```

Model residuals

Statistic	Value
Sample skewness	-3.756
Sample excess kurtosis	19.32
Passed Shapiro Wilk normality test	No (p-value = 2.53E-08 < 0.05)

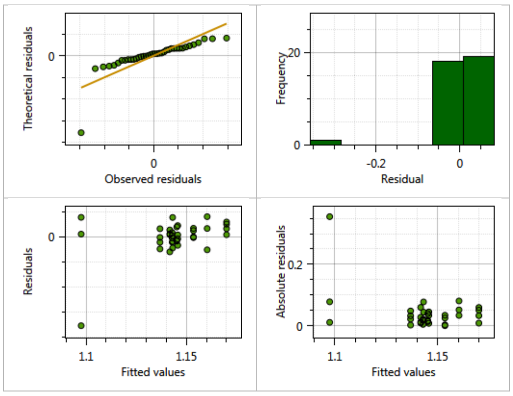

Analysis average movement duration (duration >= 5 - Zone 1)

**Analysis model**	Linear mixed model fit by REML: Average_movement_duration_duration_5_Zone_1 ~ 1 + (1|Genotype_Zone_1:Plant_Zone_1) + (1|Genotype_Zone_2:Plant_Zone_2)
**Transformation**	Natural logarithm

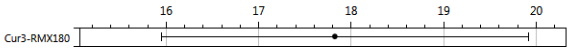

Confidence intervals average movement duration (duration >= 5 - Zone 1)

Genotype Zone 1	Genotype Zone 2	Mean	Lower 95% CL	Upper 95% CL	Group
Cur3	RMX180	17.82	15.95	19.92	a

Model summary

```
Linear mixed model fit by REML. t-tests use Satterthwaite's method ['lmerModLmerTest']
Formula: Average_movement_duration_duration_5_Zone_1 ~ 1 + (1 | Genotype_Zone_1:Plant_Zone_1) +      (1 |
Genotype_Zone_2:Plant_Zone_2)
   Data: data

REML criterion at convergence: 13.9

Scaled residuals:
    Min      1Q  Median      3Q     Max
-3.8970 -0.3255  0.0750  0.6054  1.9852

Random effects:
 Groups                       Name        Variance  Std.Dev.
 Genotype_Zone_1:Plant_Zone_1 (Intercept) 3.968e-03 6.299e-02
 Genotype_Zone_2:Plant_Zone_2 (Intercept) 1.830e-17 4.278e-09
 Residual                                 7.462e-02 2.732e-01
Number of obs: 37, groups:  Genotype_Zone_1:Plant_Zone_1, 10; Genotype_Zone_2:Plant_Zone_2, 10

Fixed effects:
            Estimate Std. Error      df t value Pr(>|t|)
(Intercept)  2.88047    0.04923 9.07376   58.51 5.21e-13 ***
---
Signif. codes:  0 '***' 0.001 '**' 0.01 '*' 0.05 '.' 0.1 ' ' 1
```

Model residuals

Statistic	Value
Sample skewness	-1.658
Sample excess kurtosis	6.199
Passed Shapiro Wilk normality test	No (p-value = 0.0007474 < 0.05)

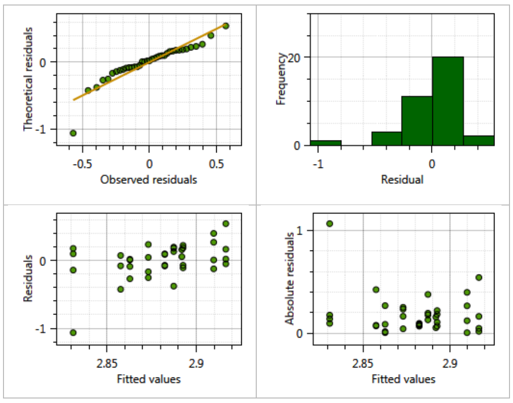

Analysis average movement duration (duration < 2 - Zone 2)

**Analysis model**	Linear mixed model fit by REML: Average_movement_duration_duration_2_Zone_2 ~ 1 + (1|Genotype_Zone_1:Plant_Zone_1) + (1|Genotype_Zone_2:Plant_Zone_2)
**Transformation**	Natural logarithm

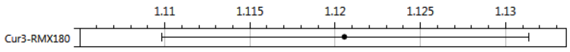

Confidence intervals average movement duration (duration < 2 - Zone 2)

Genotype Zone 1	Genotype Zone 2	Mean	Lower 95% CL	Upper 95% CL	Group
Cur3	RMX180	1.121	1.11	1.131	a

Model summary

```
Linear mixed model fit by REML. t-tests use Satterthwaite's method ['lmerModLmerTest']
Formula: Average_movement_duration_duration_2_Zone_2 ~ 1 + (1 | Genotype_Zone_1:Plant_Zone_1) +      (1 |
Genotype_Zone_2:Plant_Zone_2)
   Data: data

REML criterion at convergence: -155.8

Scaled residuals:
     Min       1Q   Median       3Q      Max
-1.87044 -0.77681 -0.01624  0.57981  3.13871

Random effects:
 Groups                       Name        Variance  Std.Dev.
 Genotype_Zone_1:Plant_Zone_1 (Intercept) 0.0000000 0.00000
 Genotype_Zone_2:Plant_Zone_2 (Intercept) 0.0000000 0.00000
```

```
 Residual                                 0.0008818 0.02969
Number of obs: 39, groups:  Genotype_Zone_1:Plant_Zone_1, 10; Genotype_Zone_2:Plant_Zone_2, 10

Fixed effects:
             Estimate Std. Error        df t value Pr(>|t|)    
(Intercept)  0.113811   0.004755 38.000000   23.93   <2e-16 ***
---
Signif. codes:  0 '***' 0.001 '**' 0.01 '*' 0.05 '.' 0.1 ' ' 1
```

Model residuals

Statistic	Value
Sample skewness	0.8466
Sample excess kurtosis	1.613
Passed Shapiro Wilk normality test	No (p-value = 0.04088 < 0.05)

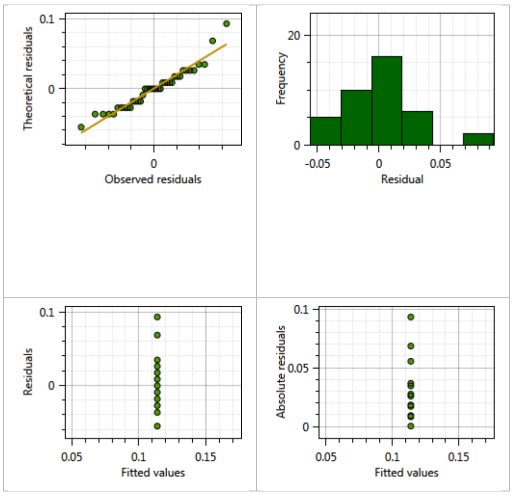

Data points with high residuals

Trial	Arena
Trial 3	12

Analysis average movement duration (2 <= duration < 5 - Zone 2)

**Analysis model**	Linear mixed model fit by REML: Average_movement_duration_2_duration_5_Zone_2 ~ 1 + (1|Genotype_Zone_1:Plant_Zone_1) + (1|Genotype_Zone_2:Plant_Zone_2)
**Transformation**	Natural logarithm

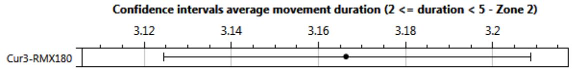

Confidence intervals average movement duration (2 <= duration < 5 - Zone 2)

Genotype Zone 1	Genotype Zone 2	Mean	Lower 95% CL	Upper 95% CL	Group
Cur3	RMX180	3.166	3.124	3.209	a

Model summary

```
Linear mixed model fit by REML. t-tests use Satterthwaite's method ['lmerModLmerTest']
Formula: Average_movement_duration_2_duration_5_Zone_2 ~ 1 + (1 | Genotype_Zone_1:Plant_Zone_1) +      (1
| Genotype_Zone_2:Plant_Zone_2)
   Data: data

REML criterion at convergence: -161.4

Scaled residuals: 
    Min      1Q  Median      3Q     Max 
-2.6439 -0.3677  0.2898  0.5166  2.2532 

Random effects:
 Groups                       Name        Variance  Std.Dev.
 Genotype_Zone_1:Plant_Zone_1 (Intercept) 1.095e-04 0.010462
 Genotype_Zone_2:Plant_Zone_2 (Intercept) 1.956e-05 0.004423
 Residual                                 6.612e-04 0.025714
Number of obs: 39, groups:  Genotype_Zone_1:Plant_Zone_1, 10; Genotype_Zone_2:Plant_Zone_2, 10

Fixed effects:
            Estimate Std. Error       df t value Pr(>|t|)    
(Intercept) 1.152572   0.005469 6.104668   210.7 5.05e-13 ***
---
Signif. codes:  0 '***' 0.001 '**' 0.01 '*' 0.05 '.' 0.1 ' ' 1
```

Model residuals

Statistic	Value
Sample skewness	-0.6762
Sample excess kurtosis	1.359
Passed Shapiro Wilk normality test	No (p-value = 0.03554 < 0.05)

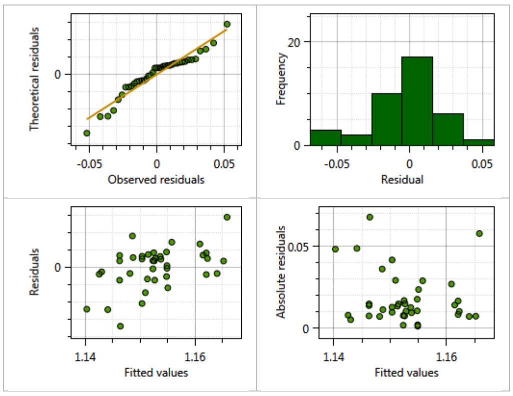

Analysis average movement duration (duration >= 5 - Zone 2)

**Analysis model**	Linear mixed model fit by REML: Average_movement_duration_duration_5_Zone_2 ~ 1 + (1|Genotype_Zone_1:Plant_Zone_1) + (1|Genotype_Zone_2:Plant_Zone_2)

Transformation	Natural logarithm

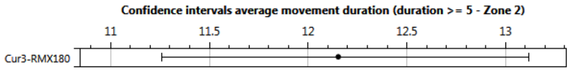

Confidence intervals average movement duration (duration >= 5 - Zone 2)

Genotype Zone 1	Genotype Zone 2	Mean	Lower 95% CL	Upper 95% CL	Group
Cur3	RMX180	12.15	11.26	13.12	a

Model summary

```
Linear mixed model fit by REML. t-tests use Satterthwaite's method ['lmerModLmerTest']
Formula: Average_movement_duration_duration_5_Zone_2 ~ 1 + (1 | Genotype_Zone_1:Plant_Zone_1) +      (1 |
Genotype_Zone_2:Plant_Zone_2)
   Data: data

REML criterion at convergence: 1.8

Scaled residuals: 
     Min       1Q   Median       3Q      Max 
-2.76907 -0.43929  0.00618  0.59035  1.92841 

Random effects:
 Groups                       Name        Variance  Std.Dev. 
 Genotype_Zone_1:Plant_Zone_1 (Intercept) 0.000e+00 0.000e+00
 Genotype_Zone_2:Plant_Zone_2 (Intercept) 1.797e-18 1.341e-09
 Residual                                 5.575e-02 2.361e-01
Number of obs: 39, groups:  Genotype_Zone_1:Plant_Zone_1, 10; Genotype_Zone_2:Plant_Zone_2, 10

Fixed effects:
            Estimate Std. Error       df t value Pr(>|t|)    
(Intercept)  2.49751    0.03781 38.00000   66.06   <2e-16 ***
---
Signif. codes:  0 '***' 0.001 '**' 0.01 '*' 0.05 '.' 0.1 ' ' 1
```

Model residuals

Statistic	Value
Sample skewness	-0.9093
Sample excess kurtosis	1.911
Passed Shapiro Wilk normality test	No (p-value = 0.005292 < 0.05)

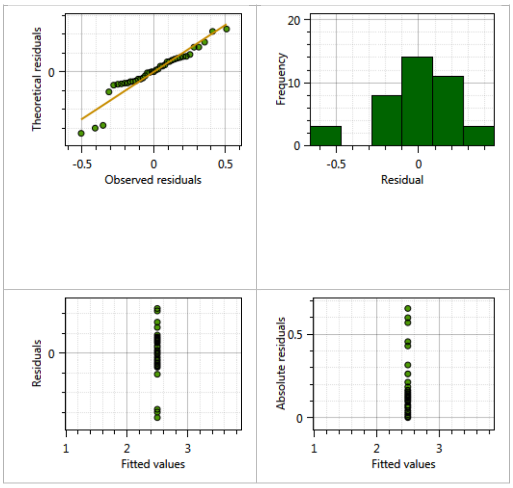

Analysis average movement duration duration < 2 (diff. Zone 1 - Zone 2)

Analysis model	Generalized linear mixed model with dispersion factor, formula=cbind(Average_movement_duration_duration_2_Zone_1,Average_movement_duration_duration_2_Zone_2) ~ 1 + (1|Genotype_Zone_1:Plant_Zone_1) + (1|Genotype_Zone_2:Plant_Zone_2)
Transformation	Logit

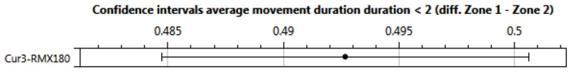

Confidence intervals average movement duration duration < 2 (diff. Zone 1 - Zone 2)

Genotype Zone 1	Genotype Zone 2	Mean	Lower 95% CL	Upper 95% CL	Group
Cur3	RMX180	0.4927	0.4847	0.5006	a

Model summary

```
Linear mixed model fit by REML. t-tests use Satterthwaite's method ['lmerModLmerTest']
Formula: ziFormula
   Data: data
Weights: wi

REML criterion at convergence: -64.1

Scaled residuals: 
    Min      1Q  Median      3Q     Max 
-5.3319 -0.2400  0.1203  0.3977  1.2362 

Random effects:
 Groups                       Name        Variance Std.Dev.
 Genotype_Zone_1:Plant_Zone_1 (Intercept) 0.000000 0.00000 
 Genotype_Zone_2:Plant_Zone_2 (Intercept) 0.000000 0.00000 
 Residual                                 0.005179 0.07197 
Number of obs: 38, groups:  Genotype_Zone_1:Plant_Zone_1, 10; Genotype_Zone_2:Plant_Zone_2, 10

Fixed effects:
            Estimate Std. Error       df t value Pr(>|t|)  
(Intercept) -0.02932    0.01571 37.00000  -1.866     0.07 .
---
Signif. codes:  0 '***' 0.001 '**' 0.01 '*' 0.05 '.' 0.1 ' ' 1

Dispersion: 0.07197
```

Model residuals

Statistic	Value
Sample skewness	-4.192
Sample excess kurtosis	22.28
Passed Shapiro Wilk normality test	No (p-value = 4.365E-09 < 0.05)

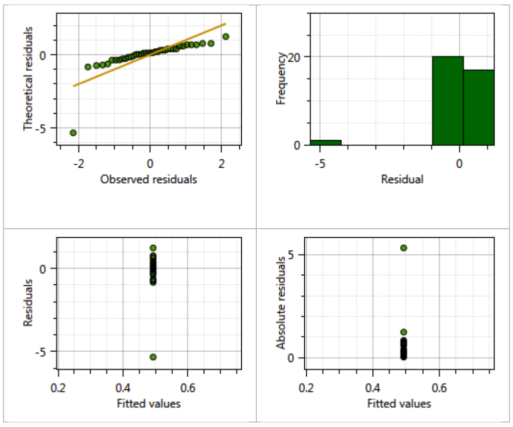

Analysis average movement duration 2 <= duration < 5 (diff. Zone 1 - Zone 2)

**Analysis model**	Generalized linear mixed model with dispersion factor, formula=cbind(Average_movement_duration_2_duration_5_Zone_1,Average_movement_duration_2_duration_5_Zone_2) ~ 1 + (1|Genotype_Zone_1:Plant_Zone_1) + (1|Genotype_Zone_2:Plant_Zone_2)
**Transformation**	Logit

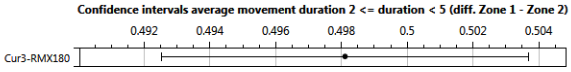

Confidence intervals average movement duration 2 <= duration < 5 (diff. Zone 1 - Zone 2)

Genotype Zone 1	Genotype Zone 2	Mean	Lower 95% CL	Upper 95% CL	Group
Cur3	RMX180	0.4981	0.4925	0.5037	a

Model summary

```
Linear mixed model fit by REML. t-tests use Satterthwaite's method ['lmerModLmerTest']
Formula: ziFormula
   Data: data
Weights: wi

REML criterion at convergence: -90.2

Scaled residuals:
    Min      1Q  Median      3Q     Max
-4.4632 -0.3452  0.0885  0.5939  1.3655

Random effects:
 Groups                      Name        Variance  Std.Dev.
 Genotype_Zone_1:Plant_Zone_1 (Intercept) 5.231e-17 7.233e-09
 Genotype_Zone_2:Plant_Zone_2 (Intercept) 0.000e+00 0.000e+00
 Residual                                7.322e-03 8.557e-02
Number of obs: 38, groups:  Genotype_Zone_1:Plant_Zone_1, 10; Genotype_Zone_2:Plant_Zone_2, 10

Fixed effects:
             Estimate Std. Error        df t value Pr(>|t|)
(Intercept) -0.007572   0.011039 37.000000  -0.686    0.497

Dispersion: 0.08557
```

Model residuals

Statistic	Value
Sample skewness	-2.353
Sample excess kurtosis	9.782
Passed Shapiro Wilk normality test	No (p-value = 2.157E-05 < 0.05)

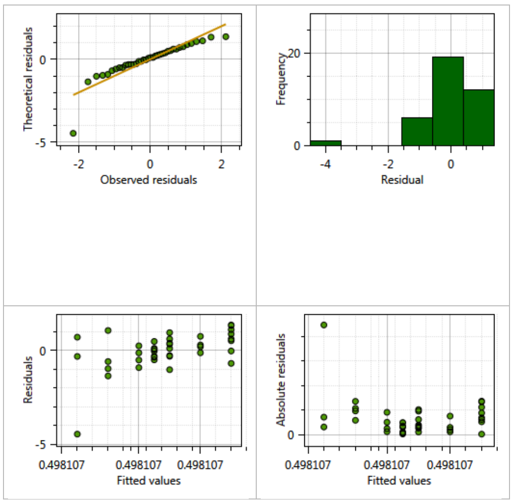

Analysis average movement duration duration >= 5 (diff. Zone 1 - Zone 2)

**Analysis model**	Generalized linear mixed model with dispersion factor, formula=cbind(Average_movement_duration_duration_5_Zone_1,Average_movement_duration_duration_5_Zone_2) ~ 1 + (1|Genotype_Zone_1:Plant_Zone_1) + (1|Genotype_Zone_2:Plant_Zone_2)
**Transformation**	Logit

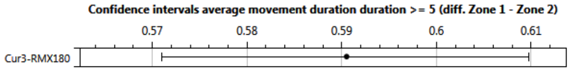

Confidence intervals average movement duration duration >= 5 (diff. Zone 1 - Zone 2)

Genotype Zone 1	Genotype Zone 2	Mean	Lower 95% CL	Upper 95% CL	Group
Cur3	RMX180	0.5905	0.571	0.6098	a

Model summary

```
Linear mixed model fit by REML. t-tests use Satterthwaite's method ['lmerModLmerTest']
Formula: ziFormula
   Data: data
```

```
Weights: wi

REML criterion at convergence: 4

Scaled residuals:
    Min      1Q  Median      3Q     Max
-1.7442 -0.7658 -0.0311  0.6109  3.4373

Random effects:
 Groups                      Name        Variance Std.Dev.
 Genotype_Zone_1:Plant_Zone_1 (Intercept) 0.00e+00 0.000e+00
 Genotype_Zone_2:Plant_Zone_2 (Intercept) 2.14e-18 1.463e-09
 Residual                                4.38e-01 6.618e-01
Number of obs: 37, groups:  Genotype_Zone_1:Plant_Zone_1, 10; Genotype_Zone_2:Plant_Zone_2, 10

Fixed effects:
            Estimate Std. Error      df t value Pr(>|t|)
(Intercept)   0.3661     0.0396 36.0000   9.244 4.86e-11 ***
---
Signif. codes:  0 '***' 0.001 '**' 0.01 '*' 0.05 '.' 0.1 ' ' 1

Dispersion: 0.6618
```

Model residuals

Statistic	Value
Sample skewness	0.9662
Sample excess kurtosis	2.647
Passed Shapiro Wilk normality test	No (p-value = 0.02424 < 0.05)

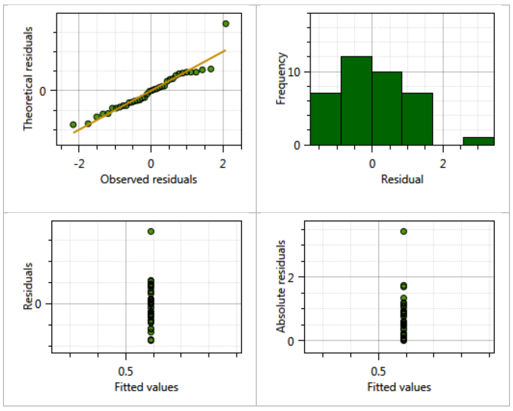

Data points with high residuals

Trial	Arena
Trial 3	24

Average velocity per zone

Selected zones	Zone 1, Zone 2
Data transformation	Natural logarithm
Analysis	Zone difference analysis

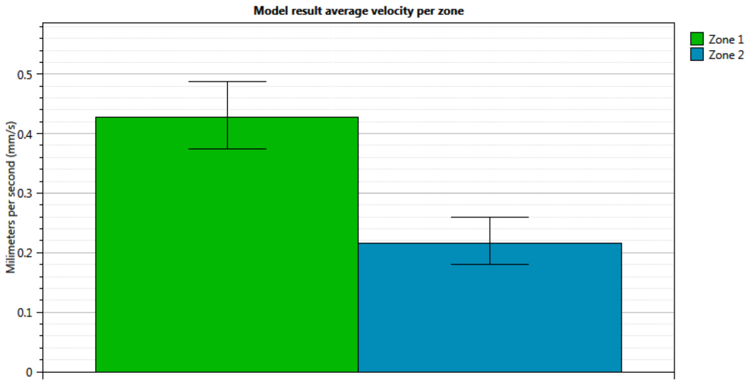

Results difference tests Zone 1 - Zone 2: p values and 95% confidence intervals of the difference on the transformed scale for each statistic.

Behaviour statistic	Cur3-RMX180
Average velocity (diff. Zone 1 - Zone 2)	p=0.000461***
	[0.316, 0.774]

The model predictions and 95% confidence intervals for each statistic.

Statistic	Cur3-RMX180	Remark
Average velocity (Zone 1)	0.427	
	[0.374, 0.488]	
Average velocity (Zone 2)	0.216	CR
	[0.18, 0.26]	

CR = Check residuals

Data summary

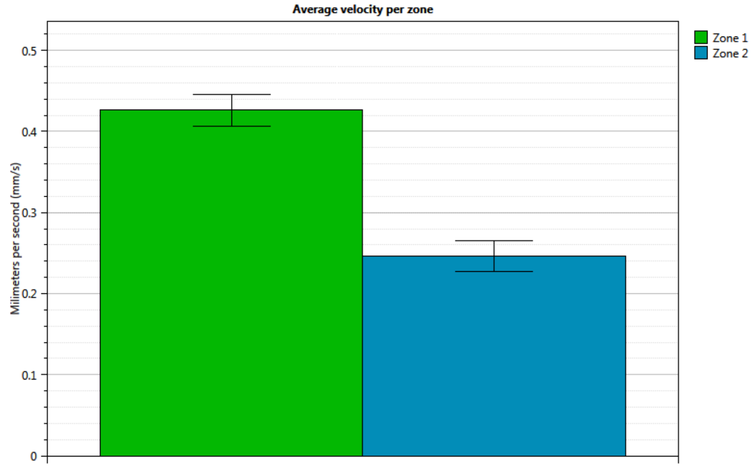

Genotype Zone 1	Genotype Zone 2	Genotype Zone 3	Mean Zone 1	StdErr Zone 1	Mean Zone 2	StdErr Zone 2
Cur3	RMX180	Neutral	0.43	0.02	0.25	0.02

Analysis average velocity (Zone 1)

**Analysis model**	Linear mixed model fit by REML: Average_velocity_Zone_1 ~ 1 + (1|Genotype_Zone_1:Plant_Zone_1) + (1|Genotype_Zone_2:Plant_Zone_2)
**Transformation**	Natural logarithm

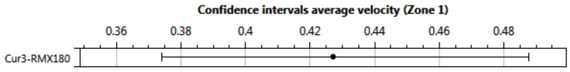

Confidence intervals average velocity (Zone 1)

Genotype Zone 1	Genotype Zone 2	Mean	Lower 95% CL	Upper 95% CL	Group
Cur3	RMX180	0.4271	0.374	0.4878	a

Model summary

```
Linear mixed model fit by REML. t-tests use Satterthwaite's method ['lmerModLmerTest']
Formula: Average_velocity_Zone_1 ~ 1 + (1 | Genotype_Zone_1:Plant_Zone_1) +      (1 |
Genotype_Zone_2:Plant_Zone_2)
   Data: data

REML criterion at convergence: -4.4

Scaled residuals:
    Min      1Q  Median      3Q     Max
-2.0731 -0.5542  0.1016  0.5345  1.7247

Random effects:
 Groups                       Name        Variance Std.Dev.
 Genotype_Zone_1:Plant_Zone_1 (Intercept) 0.004477 0.06691
 Genotype_Zone_2:Plant_Zone_2 (Intercept) 0.022924 0.15141
 Residual                                 0.031427 0.17728
Number of obs: 38, groups:  Genotype_Zone_1:Plant_Zone_1, 10; Genotype_Zone_2:Plant_Zone_2, 10

Fixed effects:
            Estimate Std. Error       df t value Pr(>|t|)
(Intercept) -0.85070    0.05984 10.23402  -14.22 4.53e-08 ***
---
Signif. codes:  0 '***' 0.001 '**' 0.01 '*' 0.05 '.' 0.1 ' ' 1
```

Model residuals

Statistic	Value
Sample skewness	-0.1527
Sample excess kurtosis	-0.04382
Passed Shapiro Wilk normality test	Yes (p-value = 0.6667 > 0.05)

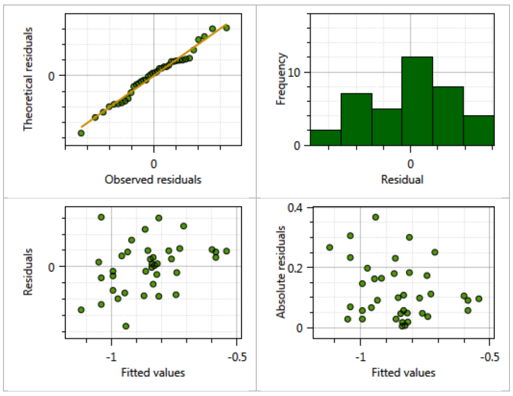

Analysis average velocity (Zone 2)

**Analysis model**	Linear mixed model fit by REML: Average_velocity_Zone_2 ~ 1 + (1|Genotype_Zone_1:Plant_Zone_1) + (1|Genotype_Zone_2:Plant_Zone_2)
**Transformation**	Natural logarithm

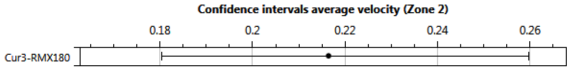

Confidence intervals average velocity (Zone 2)

Genotype Zone 1	Genotype Zone 2	Mean	Lower 95% CL	Upper 95% CL	Group
Cur3	RMX180	0.2164	0.1803	0.2598	a

Model summary

```
Linear mixed model fit by REML. t-tests use Satterthwaite's method ['lmerModLmerTest']
Formula: Average_velocity_Zone_2 ~ 1 + (1 | Genotype_Zone_1:Plant_Zone_1) +      (1 |
Genotype_Zone_2:Plant_Zone_2)
   Data: data

REML criterion at convergence: 67.9

Scaled residuals:
```

```
    Min      1Q  Median      3Q     Max 
-2.6012 -0.6508  0.1836  0.7217  1.7502 

Random effects:
 Groups                        Name        Variance Std.Dev.
 Genotype_Zone_1:Plant_Zone_1 (Intercept) 0.0000   0.0000  
 Genotype_Zone_2:Plant_Zone_2 (Intercept) 0.0000   0.0000  
 Residual                                 0.3173   0.5633  
Number of obs: 39, groups:  Genotype_Zone_1:Plant_Zone_1, 10; Genotype_Zone_2:Plant_Zone_2, 10

Fixed effects:
            Estimate Std. Error       df t value Pr(>|t|)    
(Intercept) -1.53054    0.09019 38.00000  -16.97   <2e-16 ***
---
Signif. codes:  0 '***' 0.001 '**' 0.01 '*' 0.05 '.' 0.1 ' ' 1
```

Model residuals

Statistic	Value
Sample skewness	-0.8895
Sample excess kurtosis	0.8517
Passed Shapiro Wilk normality test	No (p-value = 0.03232 < 0.05)

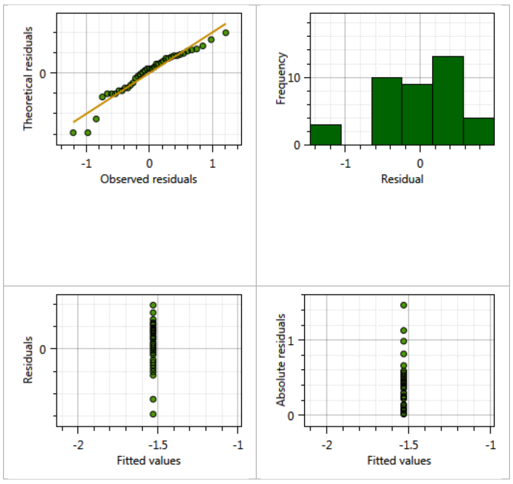

Analysis average velocity (diff. Zone 1 - Zone 2)

**Analysis model**	Generalized linear mixed model with dispersion factor, formula=cbind(Average_velocity_Zone_1,Average_velocity_Zone_2) ~ 1 + (1|Genotype_Zone_1:Plant_Zone_1) + (1|Genotype_Zone_2:Plant_Zone_2)
**Transformation**	Logit

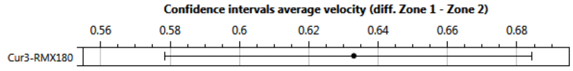

Confidence intervals average velocity (diff. Zone 1 - Zone 2)

Genotype Zone 1	Genotype Zone 2	Mean	Lower 95% CL	Upper 95% CL	Group
Cur3	RMX180	0.633	0.5784	0.6844	a

Model summary

```
Linear mixed model fit by REML. t-tests use Satterthwaite's method ['lmerModLmerTest']
Formula: ziFormula
   Data: data
Weights: wi

REML criterion at convergence: 61.5

Scaled residuals: 
     Min       1Q   Median       3Q      Max 
-1.94683 -0.59815  0.02392  0.64507  2.29962 

Random effects:
 Groups                        Name        Variance Std.Dev.
 Genotype_Zone_1:Plant_Zone_1 (Intercept) 0.00000  0.0000  
 Genotype_Zone_2:Plant_Zone_2 (Intercept) 0.04571  0.2138  
 Residual                                 0.03381  0.1839  
Number of obs: 39, groups:  Genotype_Zone_1:Plant_Zone_1, 10; Genotype_Zone_2:Plant_Zone_2, 10

Fixed effects:
            Estimate Std. Error     df t value Pr(>|t|)    
(Intercept)   0.5450     0.1009 8.8365     5.4 0.000461 ***
---
Signif. codes:  0 '***' 0.001 '**' 0.01 '*' 0.05 '.' 0.1 ' ' 1

Dispersion: 0.1839
```

Model residuals

Statistic	Value
Sample skewness	0.06683
Sample excess kurtosis	-0.3361
Passed Shapiro Wilk normality test	Yes (p-value = 0.9519 > 0.05)

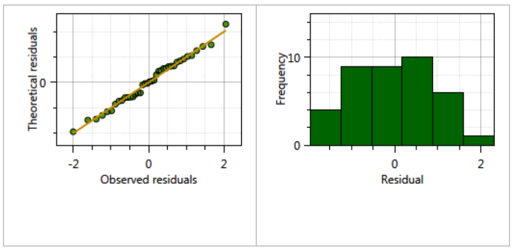

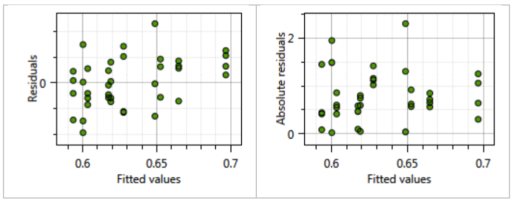

Average velocity per zone per hour

Selected hours	0, 1, 2, 3, 4, 5, 6, 7
Selected zones	Zone 1, Zone 2
Data transformation	Natural logarithm
Analysis	Zone difference analysis

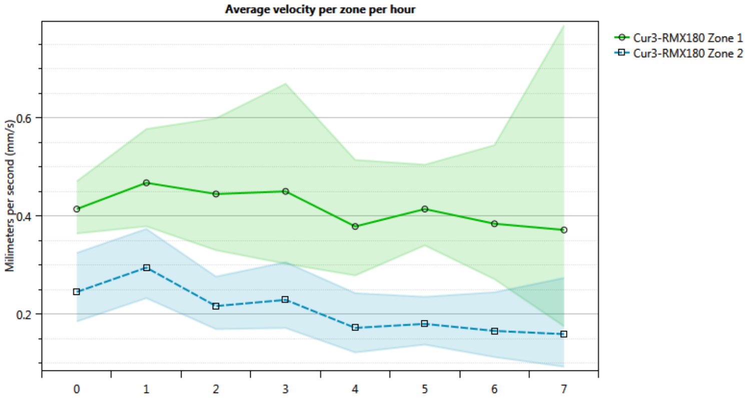

Results difference tests Zone 1 - Zone 2: p values and 95% confidence intervals of the difference on the transformed scale for each statistic.

Behaviour statistic	Cur3-RMX180
Average velocity H0 (diff. Zone 1 - Zone 2)	p=0.0213* [0.0881, 0.854]
Average velocity H1 (diff. Zone 1 - Zone 2)	p=0.102 [-0.0954, 0.897]
Average velocity H2 (diff. Zone 1 - Zone 2)	p=2.46E-06**** [0.379, 0.801]
Average velocity H3 (diff. Zone 1 - Zone 2)	p=0.0362* [0.0358, 0.796]
Average velocity H4 (diff. Zone 1 - Zone 2)	p=0.0193* [0.117, 1.02]
Average velocity H5 (diff. Zone 1 - Zone 2)	p=0.0151* [0.119, 0.853]
Average velocity H6 (diff. Zone 1 - Zone 2)	p=0.0243* [0.0513, 0.599]
Average velocity H7 (diff. Zone 1 - Zone 2)	p=0.12 [-0.168, 1.19]

The model predictions and 95% confidence intervals for each statistic.

Statistic	Cur3-RMX180	Remark
Average velocity (H0 - Zone 1)	0.414 [0.364, 0.471]	
Average velocity (H0 - Zone 2)	0.245 [0.185, 0.325]	
Average velocity (H1 - Zone 1)	0.467 [0.379, 0.577]	CR
Average velocity (H1 - Zone 2)	0.295 [0.232, 0.374]	
Average velocity (H2 - Zone 1)	0.445 [0.33, 0.599]	
Average velocity (H2 - Zone 2)	0.216 [0.169, 0.277]	
Average velocity (H3 - Zone 1)	0.45 [0.303, 0.67]	
Average velocity (H3 - Zone 2)	0.229 [0.171, 0.306]	
Average velocity (H4 - Zone 1)	0.379 [0.279, 0.514]	
Average velocity (H4 - Zone 2)	0.172 [0.122, 0.243]	CR
Average velocity (H5 - Zone 1)	0.414 [0.34, 0.504]	CR
Average velocity (H5 - Zone 2)	0.18 [0.138, 0.235]	
Average velocity (H6 - Zone 1)	0.384 [0.271, 0.544]	
Average velocity (H6 - Zone 2)	0.166 [0.112, 0.245]	
Average velocity (H7 - Zone 1)	0.371 [0.175, 0.788]	
Average velocity (H7 - Zone 2)	0.159 [0.0927, 0.274]	

CR = Check residuals

Data summary

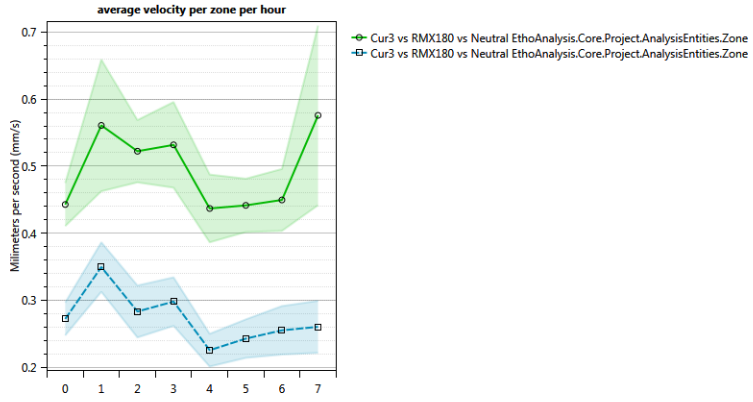

Genotype Zone 1	Genotype Zone 2	Genotype Zone 3	Mean H0 - Zone 1	StdErr H0 - Zone 1	Mean H0 - Zone 2	StdErr H0 - Zone 2	Mean H1 - Zone 1	StdErr H1 - Zone 1	Mean H1 - Zone 2	StdErr H1 - Zone 2	Mean H2 - Zone 1	StdErr H2 - Zone 1	Mean H2 - Zone 2	StdErr H2 - Zone 2	Mean H3 - Zone 1	StdErr H3 - Zone 1	Mean H3 - Zone 2	StdErr H3 - Zone 2	Mean H4 - Zone 1	StdErr H4 - Zone 1	Mean H4 - Zone 2	StdErr H4 - Zone 2	Mean H5 - Zone 1	StdErr H5 - Zone 1	Mean H5 - Zone 2	StdErr H5 - Zone 2	Mean H6 - Zone 1	StdErr H6 - Zone 1	Mean H6 - Zone 2	StdErr H6 - Zone 2
Cur3	RMX180	Neutral	0.44	0.03	0.27	0.02	0.56	0.1	0.35	0.04	0.52	0.05	0.28	0.04	0.53	0.06	0.3	0.04	0.44	0.05	0.23	0.02	0.44	0.04	0.24	0.03	0.45	0.05	0.26	0.04

Analysis average velocity (H0 - Zone 1)

**Analysis model**	Linear mixed model fit by REML: Average_velocity_H0_Zone_1 ~ 1 + (1|Genotype_Zone_1:Plant_Zone_1) + (1|Genotype_Zone_2:Plant_Zone_2)
**Transformation**	Natural logarithm

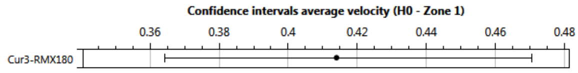

Confidence intervals average velocity (H0 - Zone 1)

Genotype Zone 1	Genotype Zone 2	Mean	Lower 95% CL	Upper 95% CL	Group
Cur3	RMX180	0.414	0.3642	0.4706	a

Model summary

```
Linear mixed model fit by REML. t-tests use Satterthwaite's method ['lmerModLmerTest']
Formula: Average_velocity_H0_Zone_1 ~ 1 + (1 | Genotype_Zone_1:Plant_Zone_1) +      (1 |
Genotype_Zone_2:Plant_Zone_2)
   Data: data

REML criterion at convergence: 29.2

Scaled residuals:
     Min       1Q   Median       3Q      Max
-1.62571 -0.71220 -0.09493  0.57710  2.60090

Random effects:
 Groups                       Name        Variance Std.Dev.
 Genotype_Zone_1:Plant_Zone_1 (Intercept) 0.0000   0.0000
 Genotype_Zone_2:Plant_Zone_2 (Intercept) 0.0000   0.0000
 Residual                                 0.1307   0.3615
Number of obs: 33, groups:  Genotype_Zone_1:Plant_Zone_1, 10; Genotype_Zone_2:Plant_Zone_2, 10

Fixed effects:
            Estimate Std. Error       df t value Pr(>|t|)
(Intercept) -0.88197    0.06293 32.00000  -14.02 3.31e-15 ***
---
Signif. codes:  0 '***' 0.001 '**' 0.01 '*' 0.05 '.' 0.1 ' ' 1
```

Model residuals

Statistic	Value
Sample skewness	0.7131
Sample excess kurtosis	0.4729
Passed Shapiro Wilk normality test	Yes (p-value = 0.2496 > 0.05)

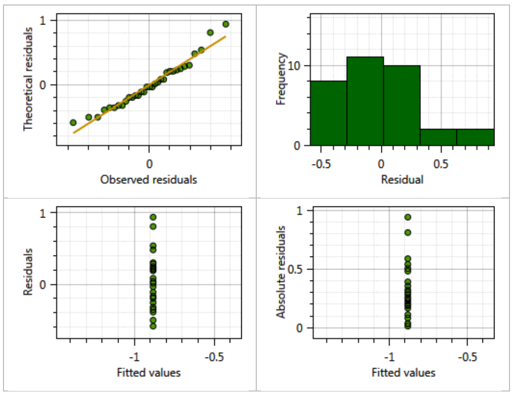

Analysis average velocity (H0 - Zone 2)

**Analysis model**	Linear mixed model fit by REML: Average_velocity_H0_Zone_2 ~ 1 + (1|Genotype_Zone_1:Plant_Zone_1) + (1|Genotype_Zone_2:Plant_Zone_2)
**Transformation**	Natural logarithm

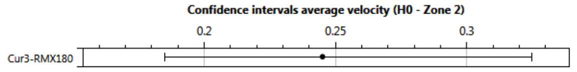

Confidence intervals average velocity (H0 - Zone 2)

Genotype Zone 1	Genotype Zone 2	Mean	Lower 95% CL	Upper 95% CL	Group
Cur3	RMX180	0.245	0.1848	0.3248	a

Model summary

```
Linear mixed model fit by REML. t-tests use Satterthwaite's method ['lmerModLmerTest']
Formula: Average_velocity_H0_Zone_2 ~ 1 + (1 | Genotype_Zone_1:Plant_Zone_1) +      (1 |
Genotype_Zone_2:Plant_Zone_2)
   Data: data
```

```
REML criterion at convergence: 50.3

Scaled residuals:
     Min       1Q   Median       3Q      Max
-1.69464 -0.54395  0.01485  0.47986  1.98368

Random effects:
 Groups                      Name        Variance Std.Dev.
 Genotype_Zone_1:Plant_Zone_1 (Intercept) 0.01577  0.1256
 Genotype_Zone_2:Plant_Zone_2 (Intercept) 0.07427  0.2725
 Residual                                 0.16316  0.4039
Number of obs: 36, groups:  Genotype_Zone_1:Plant_Zone_1, 10; Genotype_Zone_2:Plant_Zone_2, 10

Fixed effects:
            Estimate Std. Error      df t value Pr(>|t|)
(Intercept)  -1.4065     0.1171  6.4290  -12.01 1.22e-05 ***
---
Signif. codes:  0 '***' 0.001 '**' 0.01 '*' 0.05 '.' 0.1 ' ' 1
```

Model residuals

Statistic	Value
Sample skewness	0.1321
Sample excess kurtosis	-0.1797
Passed Shapiro Wilk normality test	Yes (p-value = 0.9061 > 0.05)

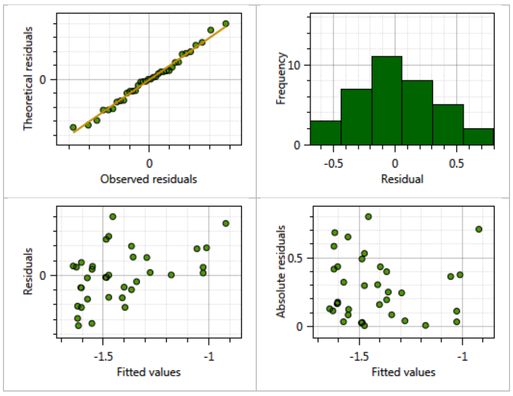

Analysis average velocity (H1 - Zone 1)

**Analysis model**	Linear mixed model fit by REML: Average_velocity_H1_Zone_1 ~ 1 + (1|Genotype_Zone_1:Plant_Zone_1) + (1|Genotype_Zone_2:Plant_Zone_2)
**Transformation**	Natural logarithm

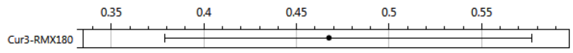

Confidence intervals average velocity (H1 - Zone 1)

Genotype Zone 1	Genotype Zone 2	Mean	Lower 95% CL	Upper 95% CL	Group
Cur3	RMX180	0.4675	0.3786	0.5772	a

Model summary

```
Linear mixed model fit by REML. t-tests use Satterthwaite's method ['lmerModLmerTest']
Formula: Average_velocity_H1_Zone_1 ~ 1 + (1 | Genotype_Zone_1:Plant_Zone_1) +      (1 |
Genotype_Zone_2:Plant_Zone_2)
   Data: data

REML criterion at convergence: 53.3

Scaled residuals:
    Min      1Q  Median      3Q     Max
-1.6158 -0.5356 -0.1470  0.4007  4.2011

Random effects:
 Groups                      Name        Variance  Std.Dev.
 Genotype_Zone_1:Plant_Zone_1 (Intercept) 2.512e-19 5.012e-10
 Genotype_Zone_2:Plant_Zone_2 (Intercept) 1.969e-02 1.403e-01
 Residual                                 2.262e-01 4.756e-01
Number of obs: 36, groups:  Genotype_Zone_1:Plant_Zone_1, 10; Genotype_Zone_2:Plant_Zone_2, 10

Fixed effects:
            Estimate Std. Error      df t value Pr(>|t|)
(Intercept)  -0.7604     0.0910  7.8029  -8.356 3.69e-05 ***
---
Signif. codes:  0 '***' 0.001 '**' 0.01 '*' 0.05 '.' 0.1 ' ' 1
```

Model residuals

Statistic	Value
Sample skewness	2.276
Sample excess kurtosis	9.447
Passed Shapiro Wilk normality test	No (p-value = 3.284E-05 < 0.05)

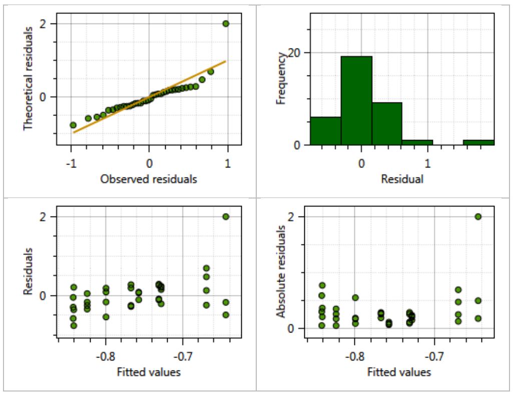

Data points with high residuals

Trial	Arena
Trial 3	12

Analysis average velocity (H1 - Zone 2)

**Analysis model**	Linear mixed model fit by REML: Average_velocity_H1_Zone_2 ~ 1 + (1|Genotype_Zone_1:Plant_Zone_1) + (1|Genotype_Zone_2:Plant_Zone_2)
**Transformation**	Natural logarithm

Confidence intervals average velocity (H1 - Zone 2)

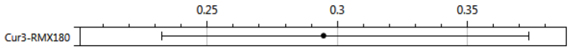

Confidence intervals average velocity (H1 - Zone 2)

Genotype Zone 1	Genotype Zone 2	Mean	Lower 95% CL	Upper 95% CL	Group
Cur3	RMX180	0.2947	0.2324	0.3737	a

Model summary

```
Linear mixed model fit by REML. t-tests use Satterthwaite's method ['lmerModLmerTest']
Formula: Average_velocity_H1_Zone_2 ~ 1 + (1 | Genotype_Zone_1:Plant_Zone_1) +      (1 |
Genotype_Zone_2:Plant_Zone_2)
   Data: data

REML criterion at convergence: 67

Scaled residuals: 
    Min      1Q  Median      3Q     Max 
-1.8731 -0.5678 -0.1508  0.5533  2.2971 

Random effects:
 Groups                       Name        Variance  Std.Dev.
 Genotype_Zone_1:Plant_Zone_1 (Intercept) 5.167e-17 7.188e-09
 Genotype_Zone_2:Plant_Zone_2 (Intercept) 2.043e-02 1.429e-01
 Residual                                 3.231e-01 5.684e-01
Number of obs: 37, groups:  Genotype_Zone_1:Plant_Zone_1, 10; Genotype_Zone_2:Plant_Zone_2, 10

Fixed effects:
            Estimate Std. Error      df t value Pr(>|t|)    
(Intercept)  -1.2218     0.1039  8.4496  -11.76 1.58e-06 ***
---
Signif. codes:  0 '***' 0.001 '**' 0.01 '*' 0.05 '.' 0.1 ' ' 1
```

Model residuals

Statistic	Value
Sample skewness	0.3035
Sample excess kurtosis	-0.1333
Passed Shapiro Wilk normality test	Yes (p-value = 0.915 > 0.05)

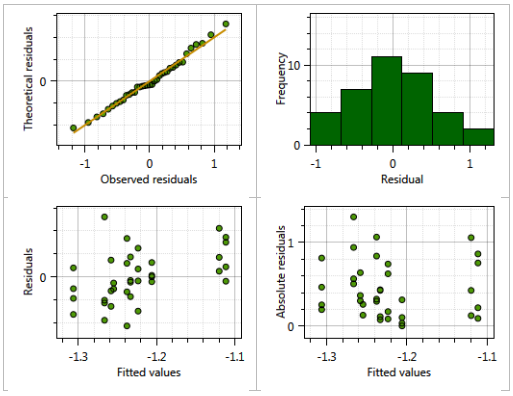

Analysis average velocity (H2 - Zone 1)

**Analysis model**	Linear mixed model fit by REML: Average_velocity_H2_Zone_1 ~ 1 + (1|Genotype_Zone_1:Plant_Zone_1) + (1|Genotype_Zone_2:Plant_Zone_2)
**Transformation**	Natural logarithm

Confidence intervals average velocity (H2 - Zone 1)

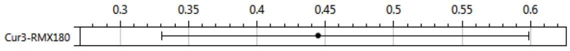

Confidence intervals average velocity (H2 - Zone 1)

Genotype Zone 1	Genotype Zone 2	Mean	Lower 95% CL	Upper 95% CL	Group
Cur3	RMX180	0.4447	0.3301	0.599	a

Model summary

```
Linear mixed model fit by REML. t-tests use Satterthwaite's method ['lmerModLmerTest']
Formula: Average_velocity_H2_Zone_1 ~ 1 + (1 | Genotype_Zone_1:Plant_Zone_1) +      (1 |
Genotype_Zone_2:Plant_Zone_2)
   Data: data

REML criterion at convergence: 63.9

Scaled residuals: 
    Min      1Q  Median      3Q     Max 
-1.7899 -0.6867 -0.0854  0.7646  1.4923 

Random effects:
 Groups                       Name        Variance Std.Dev.
 Genotype_Zone_1:Plant_Zone_1 (Intercept) 0.04191  0.2047  
 Genotype_Zone_2:Plant_Zone_2 (Intercept) 0.06173  0.2485  
 Residual                                 0.25105  0.5010  
Number of obs: 36, groups:  Genotype_Zone_1:Plant_Zone_1, 10; Genotype_Zone_2:Plant_Zone_2, 10

Fixed effects:
            Estimate Std. Error      df t value Pr(>|t|)    
(Intercept)  -0.8104     0.1323  9.2794  -6.126 0.000153 ***
---
Signif. codes:  0 '***' 0.001 '**' 0.01 '*' 0.05 '.' 0.1 ' ' 1
```

Model residuals

Statistic	Value
Sample skewness	-0.1718
Sample excess kurtosis	-0.9494
Passed Shapiro Wilk normality test	Yes (p-value = 0.2771 > 0.05)

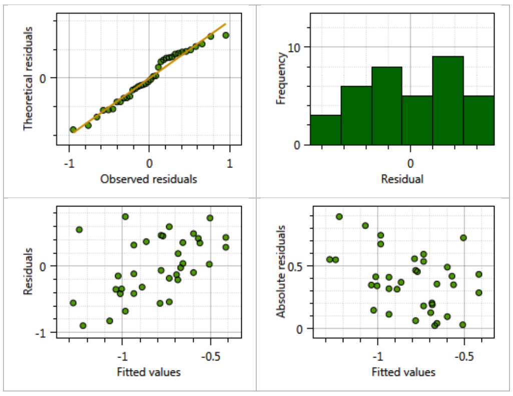

Analysis average velocity (H2 - Zone 2)

**Analysis model**	Linear mixed model fit by REML: Average_velocity_H2_Zone_2 ~ 1 + (1|Genotype_Zone_1:Plant_Zone_1) + (1|Genotype_Zone_2:Plant_Zone_2)
**Transformation**	Natural logarithm

Confidence intervals average velocity (H2 - Zone 2)

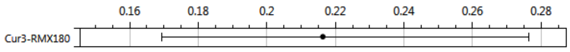

Confidence intervals average velocity (H2 - Zone 2)

Genotype Zone 1	Genotype Zone 2	Mean	Lower 95% CL	Upper 95% CL	Group
Cur3	RMX180	0.2163	0.1692	0.2765	a

Model summary

```
Linear mixed model fit by REML. t-tests use Satterthwaite's method ['lmerModLmerTest']
Formula: Average_velocity_H2_Zone_2 ~ 1 + (1 | Genotype_Zone_1:Plant_Zone_1) +      (1 |
Genotype_Zone_2:Plant_Zone_2)
   Data: data

REML criterion at convergence: 83.7

Scaled residuals:
    Min      1Q  Median      3Q     Max
-2.2930 -0.5911 -0.1066  0.3982  2.1067

Random effects:
 Groups                       Name        Variance Std.Dev.
 Genotype_Zone_1:Plant_Zone_1 (Intercept) 0.0000   0.0000
 Genotype_Zone_2:Plant_Zone_2 (Intercept) 0.0000   0.0000
 Residual                                 0.5419   0.7361
Number of obs: 37, groups:  Genotype_Zone_1:Plant_Zone_1, 10; Genotype_Zone_2:Plant_Zone_2, 10

Fixed effects:
            Estimate Std. Error     df t value Pr(>|t|)
(Intercept)   -1.531      0.121 36.000  -12.65 8.19e-15 ***
---
Signif. codes:  0 '***' 0.001 '**' 0.01 '*' 0.05 '.' 0.1 ' ' 1
```

Model residuals

Statistic	Value
Sample skewness	0.1489
Sample excess kurtosis	0.2146
Passed Shapiro Wilk normality test	Yes (p-value = 0.4503 > 0.05)

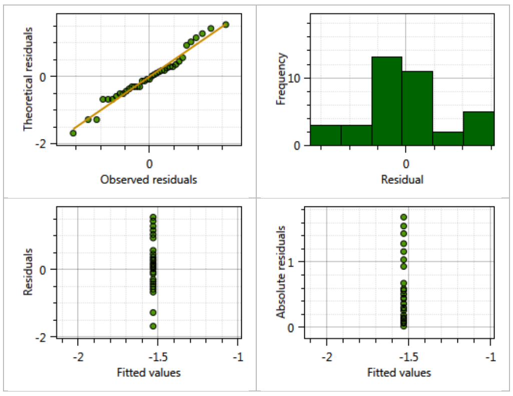

Analysis average velocity (H3 - Zone 1)

**Analysis model**	Linear mixed model fit by REML: Average_velocity_H3_Zone_1 ~ 1 + (1|Genotype_Zone_1:Plant_Zone_1) + (1|Genotype_Zone_2:Plant_Zone_2)
**Transformation**	Natural logarithm

Confidence intervals average velocity (H3 - Zone 1)

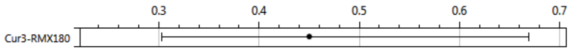

Confidence intervals average velocity (H3 - Zone 1)

Genotype Zone 1	Genotype Zone 2	Mean	Lower 95% CL	Upper 95% CL	Group
Cur3	RMX180	0.45	0.3025	0.6695	a

Model summary

```
Linear mixed model fit by REML. t-tests use Satterthwaite's method ['lmerModLmerTest']
Formula: Average_velocity_H3_Zone_1 ~ 1 + (1 | Genotype_Zone_1:Plant_Zone_1) +      (1 |
Genotype_Zone_2:Plant_Zone_2)
   Data: data

REML criterion at convergence: 63.2

Scaled residuals:
     Min       1Q   Median       3Q      Max
-2.12104 -0.45402  0.09116  0.53058  1.72437

Random effects:
 Groups                       Name        Variance Std.Dev.
 Genotype_Zone_1:Plant_Zone_1 (Intercept) 0.0000   0.0000
 Genotype_Zone_2:Plant_Zone_2 (Intercept) 0.2303   0.4799
```

```
 Residual                                  0.2578   0.5078
Number of obs: 33, groups:  Genotype_Zone_1:Plant_Zone_1, 10; Genotype_Zone_2:Plant_Zone_2, 10

Fixed effects:
            Estimate Std. Error      df t value Pr(>|t|)
(Intercept)  -0.7984     0.1763  9.2523  -4.528  0.00133 **
---
Signif. codes:  0 '***' 0.001 '**' 0.01 '*' 0.05 '.' 0.1 ' ' 1
```

Model residuals

Statistic	Value
Sample skewness	-0.2425
Sample excess kurtosis	-0.07554
Passed Shapiro Wilk normality test	Yes (p-value = 0.8875 > 0.05)

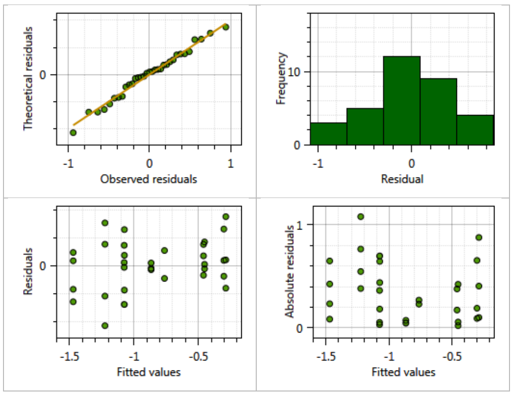

Analysis average velocity (H3 - Zone 2)

**Analysis model**	Linear mixed model fit by REML: Average_velocity_H3_Zone_2 ~ 1 + (1|Genotype_Zone_1:Plant_Zone_1) + (1|Genotype_Zone_2:Plant_Zone_2)
**Transformation**	Natural logarithm

Confidence intervals average velocity (H3 - Zone 2)

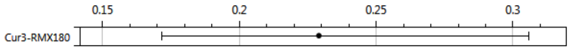

Confidence intervals average velocity (H3 - Zone 2)

Genotype Zone 1	Genotype Zone 2	Mean	Lower 95% CL	Upper 95% CL	Group
Cur3	RMX180	0.229	0.1715	0.3059	a

Model summary

```
Linear mixed model fit by REML. t-tests use Satterthwaite's method ['lmerModLmerTest']
Formula: Average_velocity_H3_Zone_2 ~ 1 + (1 | Genotype_Zone_1:Plant_Zone_1) +      (1 |
Genotype_Zone_2:Plant_Zone_2)
   Data: data

REML criterion at convergence: 91.8

Scaled residuals:
     Min       1Q   Median       3Q      Max
-2.26168 -0.31273 -0.05163  0.56670  1.90964

Random effects:
 Groups                       Name        Variance  Std.Dev.
 Genotype_Zone_1:Plant_Zone_1 (Intercept) 0.0002197 0.01482
 Genotype_Zone_2:Plant_Zone_2 (Intercept) 0.0000000 0.00000
 Residual                                 0.5949911 0.77136
Number of obs: 39, groups:  Genotype_Zone_1:Plant_Zone_1, 10; Genotype_Zone_2:Plant_Zone_2, 10

Fixed effects:
            Estimate Std. Error      df t value Pr(>|t|)
(Intercept)  -1.4739     0.1236  7.3602  -11.92 4.46e-06 ***
---
Signif. codes:  0 '***' 0.001 '**' 0.01 '*' 0.05 '.' 0.1 ' ' 1
```

Model residuals

Statistic	Value
Sample skewness	-0.3572
Sample excess kurtosis	0.1168
Passed Shapiro Wilk normality test	Yes (p-value = 0.3109 > 0.05)

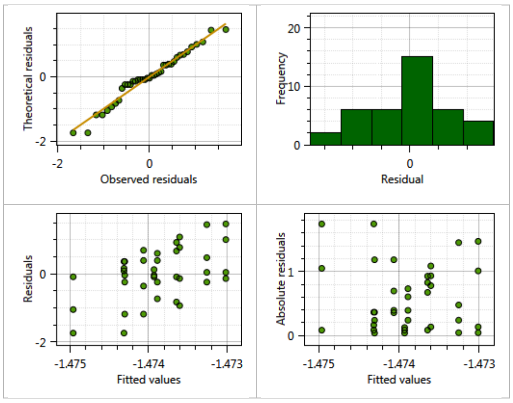

Analysis average velocity (H4 - Zone 1)

**Analysis model**	Linear mixed model fit by REML: Average_velocity_H4_Zone_1 ~ 1 + (1|Genotype_Zone_1:Plant_Zone_1) + (1|Genotype_Zone_2:Plant_Zone_2)
**Transformation**	Natural logarithm

Confidence intervals average velocity (H4 - Zone 1)

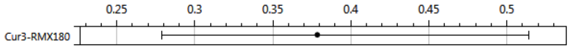

Confidence intervals average velocity (H4 - Zone 1)

Genotype Zone 1	Genotype Zone 2	Mean	Lower 95% CL	Upper 95% CL	Group
Cur3	RMX180	0.3785	0.2786	0.5142	a

Model summary

```
Linear mixed model fit by REML. t-tests use Satterthwaite's method ['lmerModLmerTest']
Formula: Average_velocity_H4_Zone_1 ~ 1 + (1 | Genotype_Zone_1:Plant_Zone_1) +      (1 |
Genotype_Zone_2:Plant_Zone_2)
   Data: data

REML criterion at convergence: 55.3

Scaled residuals:
     Min       1Q   Median       3Q      Max
-2.90706 -0.33262  0.09275  0.42585  2.04749

Random effects:
 Groups                       Name        Variance Std.Dev.
 Genotype_Zone_1:Plant_Zone_1 (Intercept) 0.00000  0.0000
 Genotype_Zone_2:Plant_Zone_2 (Intercept) 0.05304  0.2303
 Residual                                 0.30924  0.5561
Number of obs: 30, groups:  Genotype_Zone_1:Plant_Zone_1, 10; Genotype_Zone_2:Plant_Zone_2, 10

Fixed effects:
            Estimate Std. Error      df t value Pr(>|t|)
(Intercept)  -0.9715     0.1259  6.1396  -7.716 0.000222 ***
---
Signif. codes:  0 '***' 0.001 '**' 0.01 '*' 0.05 '.' 0.1 ' ' 1
```

Model residuals

Statistic	Value
Sample skewness	-0.7258
Sample excess kurtosis	2.438
Passed Shapiro Wilk normality test	Yes (p-value = 0.1461 > 0.05)

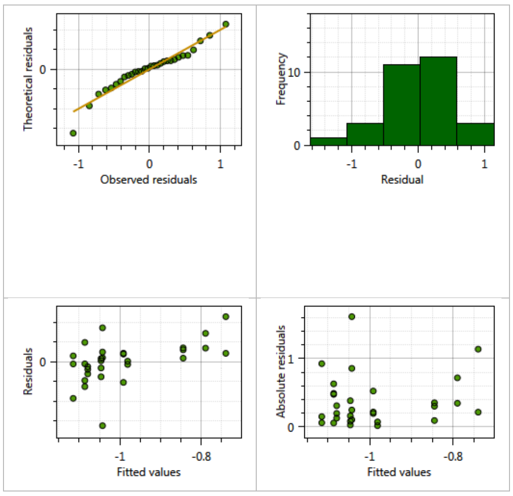

Analysis average velocity (H4 - Zone 2)

**Analysis model**	Linear mixed model fit by REML: Average_velocity_H4_Zone_2 ~ 1 + (1|Genotype_Zone_1:Plant_Zone_1) + (1|Genotype_Zone_2:Plant_Zone_2)
**Transformation**	Natural logarithm

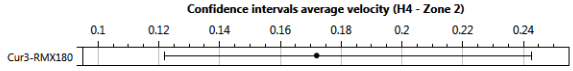

Confidence intervals average velocity (H4 - Zone 2)

Genotype Zone 1	Genotype Zone 2	Mean	Lower 95% CL	Upper 95% CL	Group
Cur3	RMX180	0.1719	0.1218	0.2427	a

Model summary

```
Linear mixed model fit by REML. t-tests use Satterthwaite's method ['lmerModLmerTest']
Formula: Average_velocity_H4_Zone_2 ~ 1 + (1 | Genotype_Zone_1:Plant_Zone_1) +      (1 |
Genotype_Zone_2:Plant_Zone_2)
   Data: data

REML criterion at convergence: 91.7

Scaled residuals:
    Min      1Q  Median      3Q     Max
-2.2171 -0.7438  0.3471  0.7403  1.3912

Random effects:
 Groups                       Name        Variance Std.Dev.
 Genotype_Zone_1:Plant_Zone_1 (Intercept) 0.00000  0.0000
 Genotype_Zone_2:Plant_Zone_2 (Intercept) 0.06441  0.2538
 Residual                                 0.62500  0.7906
Number of obs: 37, groups:  Genotype_Zone_1:Plant_Zone_1, 10; Genotype_Zone_2:Plant_Zone_2, 10

Fixed effects:
            Estimate Std. Error      df t value Pr(>|t|)
(Intercept)  -1.7607     0.1533  9.3037  -11.49 8.36e-07 ***
---
Signif. codes:  0 '***' 0.001 '**' 0.01 '*' 0.05 '.' 0.1 ' ' 1
```

Model residuals

Statistic	Value
Sample skewness	-0.5548
Sample excess kurtosis	-0.8162
Passed Shapiro Wilk normality test	No (p-value = 0.01441 < 0.05)

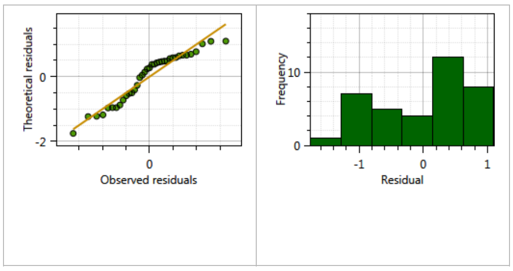

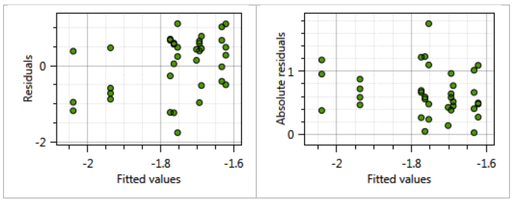

Analysis average velocity (H5 - Zone 1)

**Analysis model**	Linear mixed model fit by REML: Average_velocity_H5_Zone_1 ~ 1 + (1|Genotype_Zone_1:Plant_Zone_1) + (1|Genotype_Zone_2:Plant_Zone_2)
**Transformation**	Natural logarithm

Confidence intervals average velocity (H5 - Zone 1)

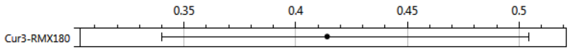

Confidence intervals average velocity (H5 - Zone 1)

Genotype Zone 1	Genotype Zone 2	Mean	Lower 95% CL	Upper 95% CL	Group
Cur3	RMX180	0.414	0.3398	0.5045	a

Model summary

```
Linear mixed model fit by REML. t-tests use Satterthwaite's method ['lmerModLmerTest']
Formula: Average_velocity_H5_Zone_1 ~ 1 + (1 | Genotype_Zone_1:Plant_Zone_1) +      (1 |
Genotype_Zone_2:Plant_Zone_2)
   Data: data

REML criterion at convergence: 39.6

Scaled residuals:
    Min      1Q  Median      3Q     Max
-2.8587 -0.2832  0.1598  0.4430  2.1816

Random effects:
 Groups                       Name        Variance Std.Dev.
 Genotype_Zone_1:Plant_Zone_1 (Intercept) 0.01219  0.1104
 Genotype_Zone_2:Plant_Zone_2 (Intercept) 0.00000  0.0000
 Residual                                 0.19330  0.4397
Number of obs: 30, groups:  Genotype_Zone_1:Plant_Zone_1, 10; Genotype_Zone_2:Plant_Zone_2, 10

Fixed effects:
            Estimate Std. Error       df t value Pr(>|t|)
(Intercept) -0.88178    0.08793  9.41978  -10.03 2.47e-06 ***
---
Signif. codes:  0 '***' 0.001 '**' 0.01 '*' 0.05 '.' 0.1 ' ' 1
```

Model residuals

Statistic	Value
Sample skewness	-0.5711
Sample excess kurtosis	2.302
Passed Shapiro Wilk normality test	No (p-value = 0.03003 < 0.05)

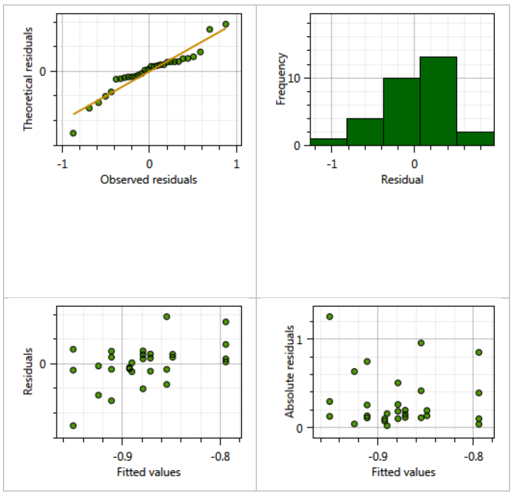

Analysis average velocity (H5 - Zone 2)

**Analysis model**	Linear mixed model fit by REML: Average_velocity_H5_Zone_2 ~ 1 + (1|Genotype_Zone_1:Plant_Zone_1) + (1|Genotype_Zone_2:Plant_Zone_2)
**Transformation**	Natural logarithm

Confidence intervals average velocity (H5 - Zone 2)

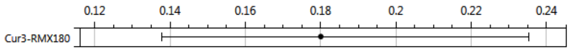

Confidence intervals average velocity (H5 - Zone 2)

Genotype Zone 1	Genotype Zone 2	Mean	Lower 95% CL	Upper 95% CL	Group
Cur3	RMX180	0.18	0.1377	0.2354	a

Model summary

```
Linear mixed model fit by REML. t-tests use Satterthwaite's method ['lmerModLmerTest']
Formula: Average_velocity_H5_Zone_2 ~ 1 + (1 | Genotype_Zone_1:Plant_Zone_1) +      (1 |
Genotype_Zone_2:Plant_Zone_2)
   Data: data

REML criterion at convergence: 97

Scaled residuals:
     Min       1Q   Median       3Q      Max
-2.16750 -0.59587  0.06523  0.83827  1.60757

Random effects:
 Groups                       Name        Variance  Std.Dev.
 Genotype_Zone_1:Plant_Zone_1 (Intercept) 0.000e+00 0.000e+00
 Genotype_Zone_2:Plant_Zone_2 (Intercept) 3.473e-15 5.893e-08
 Residual                                 6.835e-01 8.267e-01
Number of obs: 39, groups:  Genotype_Zone_1:Plant_Zone_1, 10; Genotype_Zone_2:Plant_Zone_2, 10

Fixed effects:
            Estimate Std. Error      df t value Pr(>|t|)
(Intercept)  -1.7147     0.1324 38.0000  -12.95 1.63e-15 ***
```

```
---
Signif. codes:  0 '***' 0.001 '**' 0.01 '*' 0.05 '.' 0.1 ' ' 1
```

Model residuals

Statistic	Value
Sample skewness	-0.245
Sample excess kurtosis	-0.8185
Passed Shapiro Wilk normality test	Yes (p-value = 0.27 > 0.05)

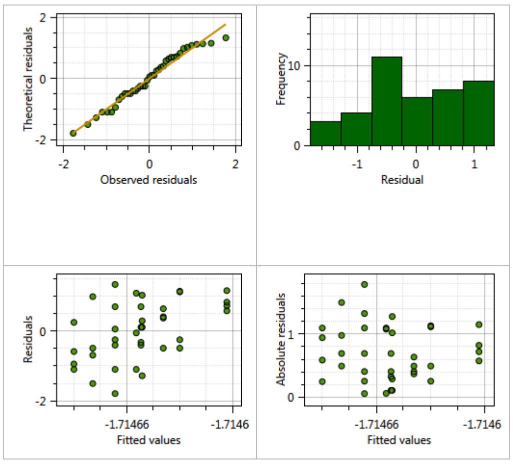

Analysis average velocity (H6 - Zone 1)

**Analysis model**	Linear mixed model fit by REML: Average_velocity_H6_Zone_1 ~ 1 + (1|Genotype_Zone_1:Plant_Zone_1) + (1|Genotype_Zone_2:Plant_Zone_2)
**Transformation**	Natural logarithm

Confidence intervals average velocity (H6 - Zone 1)

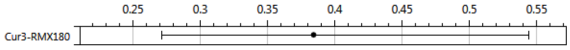

Confidence intervals average velocity (H6 - Zone 1)

Genotype Zone 1	Genotype Zone 2	Mean	Lower 95% CL	Upper 95% CL	Group
Cur3	RMX180	0.3841	0.2711	0.5442	a

Model summary

```
Linear mixed model fit by REML. t-tests use Satterthwaite's method ['lmerModLmerTest']
Formula: Average_velocity_H6_Zone_1 ~ 1 + (1 | Genotype_Zone_1:Plant_Zone_1) +      (1 |
Genotype_Zone_2:Plant_Zone_2)
   Data: data

REML criterion at convergence: 49.4

Scaled residuals: 
    Min      1Q  Median      3Q     Max 
-1.9123 -0.7587  0.2224  0.5453  1.8144 

Random effects:
 Groups                       Name        Variance Std.Dev.
 Genotype_Zone_1:Plant_Zone_1 (Intercept) 0.0000   0.0000  
 Genotype_Zone_2:Plant_Zone_2 (Intercept) 0.1247   0.3531  
 Residual                                 0.2631   0.5129  
Number of obs: 27, groups:  Genotype_Zone_1:Plant_Zone_1, 10; Genotype_Zone_2:Plant_Zone_2, 10

Fixed effects:
            Estimate Std. Error      df t value Pr(>|t|)    
(Intercept)  -0.9569     0.1528  8.5314  -6.263 0.000185 ***
---
Signif. codes:  0 '***' 0.001 '**' 0.01 '*' 0.05 '.' 0.1 ' ' 1
```

Model residuals

Statistic	Value
Sample skewness	-0.03375
Sample excess kurtosis	-0.5358
Passed Shapiro Wilk normality test	Yes (p-value = 0.6358 > 0.05)

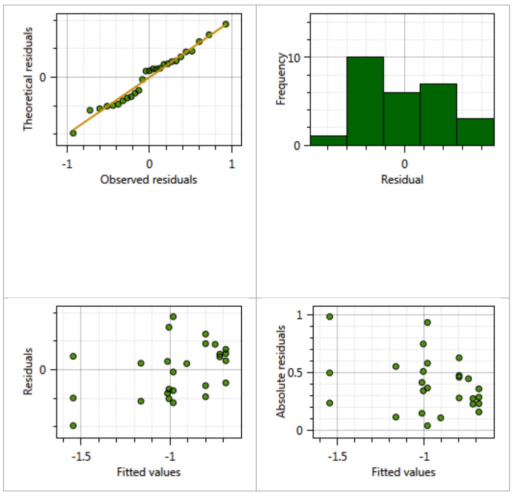

Analysis average velocity (H6 - Zone 2)

**Analysis model**	Linear mixed model fit by REML: Average_velocity_H6_Zone_2 ~ 1 + (1|Genotype_Zone_1:Plant_Zone_1) + (1|Genotype_Zone_2:Plant_Zone_2)
**Transformation**	Natural logarithm

Confidence intervals average velocity (H6 - Zone 2)

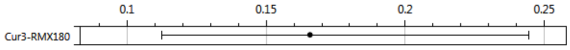

Confidence intervals average velocity (H6 - Zone 2)

Genotype Zone 1	Genotype Zone 2	Mean	Lower 95% CL	Upper 95% CL	Group
Cur3	RMX180	0.1657	0.1123	0.2445	a

Model summary

```
Linear mixed model fit by REML. t-tests use Satterthwaite's method ['lmerModLmerTest']
Formula: Average_velocity_H6_Zone_2 ~ 1 + (1 | Genotype_Zone_1:Plant_Zone_1) +      (1 |
Genotype_Zone_2:Plant_Zone_2)
   Data: data

REML criterion at convergence: 108.7

Scaled residuals: 
    Min      1Q  Median      3Q     Max 
-1.6973 -0.9920  0.2281  0.7182  1.7617 

Random effects:
 Groups                       Name        Variance Std.Dev.
 Genotype_Zone_1:Plant_Zone_1 (Intercept) 0.00000  0.0000  
 Genotype_Zone_2:Plant_Zone_2 (Intercept) 0.03609  0.1900  
 Residual                                 0.96996  0.9849  
Number of obs: 38, groups:  Genotype_Zone_1:Plant_Zone_1, 10; Genotype_Zone_2:Plant_Zone_2, 10

Fixed effects:
            Estimate Std. Error      df t value Pr(>|t|)    
(Intercept)  -1.7973     0.1708  8.6357  -10.52 3.23e-06 ***
---
Signif. codes:  0 '***' 0.001 '**' 0.01 '*' 0.05 '.' 0.1 ' ' 1
```

Model residuals

Statistic	Value
Sample skewness	-0.1812
Sample excess kurtosis	-1.188
Passed Shapiro Wilk normality test	Yes (p-value = 0.06215 > 0.05)

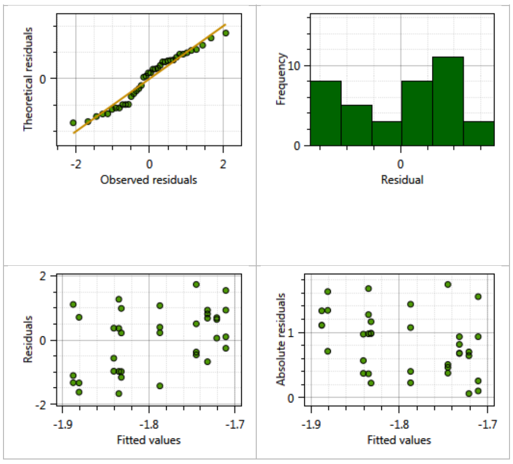

Analysis average velocity (H7 - Zone 1)

**Analysis model**	Linear mixed model fit by REML: Average_velocity_H7_Zone_1 ~ 1 + (1|Genotype_Zone_1:Plant_Zone_1) + (1|Genotype_Zone_2:Plant_Zone_2)
**Transformation**	Natural logarithm

Confidence intervals average velocity (H7 - Zone 1)

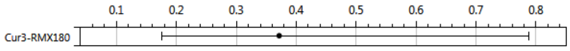

Confidence intervals average velocity (H7 - Zone 1)

Genotype Zone 1	Genotype Zone 2	Mean	Lower 95% CL	Upper 95% CL	Group
Cur3	RMX180	0.3715	0.175	0.7884	a

Model summary

```
Linear mixed model fit by REML. t-tests use Satterthwaite's method ['lmerModLmerTest']
Formula: Average_velocity_H7_Zone_1 ~ 1 + (1 | Genotype_Zone_1:Plant_Zone_1) +      (1 |
Genotype_Zone_2:Plant_Zone_2)
   Data: data

REML criterion at convergence: 66.5

Scaled residuals: 
    Min      1Q  Median      3Q     Max 
-1.8832 -0.4049 -0.1386  0.2900  1.4995 

Random effects:
 Groups                       Name        Variance Std.Dev.
 Genotype_Zone_2:Plant_Zone_2 (Intercept) 0.5650   0.7517  
 Genotype_Zone_1:Plant_Zone_1 (Intercept) 0.3269   0.5717  
 Residual                                 0.2030   0.4506  
Number of obs: 28, groups:  Genotype_Zone_2:Plant_Zone_2, 10; Genotype_Zone_1:Plant_Zone_1, 9

Fixed effects:
            Estimate Std. Error      df t value Pr(>|t|)  
(Intercept)  -0.9903     0.3207  7.2718  -3.088   0.0168 *
---
Signif. codes:  0 '***' 0.001 '**' 0.01 '*' 0.05 '.' 0.1 ' ' 1
```

Model residuals

Statistic	Value
Sample skewness	-0.0192
Sample excess kurtosis	0.8524
Passed Shapiro Wilk normality test	Yes (p-value = 0.4259 > 0.05)

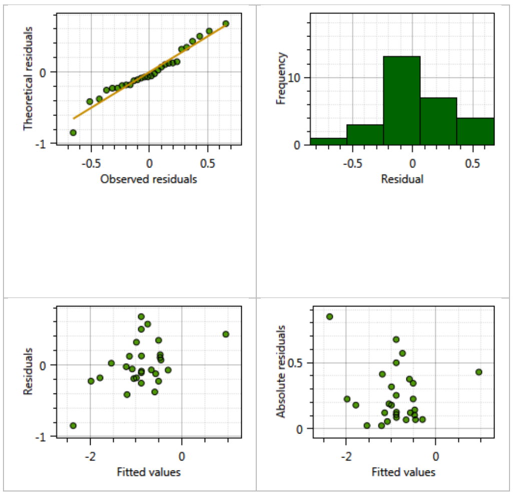

Analysis average velocity (H7 - Zone 2)

**Analysis model**	Linear mixed model fit by REML: Average_velocity_H7_Zone_2 ~ 1 + (1|Genotype_Zone_1:Plant_Zone_1) + (1|Genotype_Zone_2:Plant_Zone_2)
**Transformation**	Natural logarithm

Confidence intervals average velocity (H7 - Zone 2)

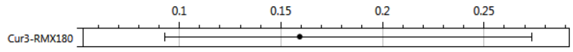

Confidence intervals average velocity (H7 - Zone 2)

Genotype Zone 1	Genotype Zone 2	Mean	Lower 95% CL	Upper 95% CL	Group
Cur3	RMX180	0.1592	0.09266	0.2737	a

Model summary

```
Linear mixed model fit by REML. t-tests use Satterthwaite's method ['lmerModLmerTest']
Formula: Average_velocity_H7_Zone_2 ~ 1 + (1 | Genotype_Zone_1:Plant_Zone_1) +      (1 |
Genotype_Zone_2:Plant_Zone_2)
   Data: data

REML criterion at convergence: 110.9

Scaled residuals:
     Min       1Q   Median       3Q      Max
-1.37109 -0.71195 -0.02461  0.70421  1.79367

Random effects:
 Groups                       Name        Variance Std.Dev.
 Genotype_Zone_1:Plant_Zone_1 (Intercept) 0.1809   0.4254
 Genotype_Zone_2:Plant_Zone_2 (Intercept) 0.2193   0.4683
 Residual                                 0.7070   0.8408
Number of obs: 39, groups:  Genotype_Zone_1:Plant_Zone_1, 10; Genotype_Zone_2:Plant_Zone_2, 10

Fixed effects:
            Estimate Std. Error      df t value Pr(>|t|)
(Intercept)  -1.8374     0.2414  9.5404   -7.61 2.38e-05 ***
---
Signif. codes:  0 '***' 0.001 '**' 0.01 '*' 0.05 '.' 0.1 ' ' 1
```

Model residuals

Statistic	Value
Sample skewness	0.08375
Sample excess kurtosis	-1.123
Passed Shapiro Wilk normality test	Yes (p-value = 0.1304 > 0.05)

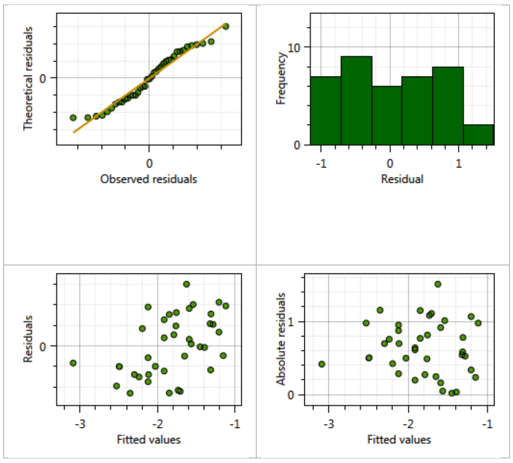

Analysis average velocity H0 (diff. Zone 1 - Zone 2)

**Analysis model**	Generalized linear mixed model with dispersion factor, formula=cbind(Average_velocity_H0_Zone_1,Average_velocity_H0_Zone_2) ~ 1 + (1|Genotype_Zone_1:Plant_Zone_1) + (1|Genotype_Zone_2:Plant_Zone_2)
**Transformation**	Logit

Confidence intervals average velocity H0 (diff. Zone 1 - Zone 2)

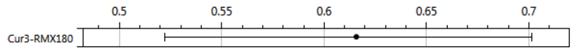

Confidence intervals average velocity H0 (diff. Zone 1 - Zone 2)

Genotype Zone 1	Genotype Zone 2	Mean	Lower 95% CL	Upper 95% CL	Group
Cur3	RMX180	0.6157	0.522	0.7015	a

Model summary

```
Linear mixed model fit by REML. t-tests use Satterthwaite's method ['lmerModLmerTest']
Formula: ziFormula
   Data: data
Weights: wi
```

```
REML criterion at convergence: 60.9

Scaled residuals:
    Min      1Q  Median      3Q     Max
-1.6789 -0.3560  0.0781  0.4209  2.0716

Random effects:
 Groups                        Name        Variance Std.Dev.
 Genotype_Zone_1:Plant_Zone_1 (Intercept) 0.08577  0.2929
 Genotype_Zone_2:Plant_Zone_2 (Intercept) 0.11463  0.3386
 Residual                                0.03749  0.1936
Number of obs: 32, groups:  Genotype_Zone_1:Plant_Zone_1, 10; Genotype_Zone_2:Plant_Zone_2, 10

Fixed effects:
            Estimate Std. Error     df t value Pr(>|t|)
(Intercept)   0.4713     0.1692 8.9458   2.785   0.0213 *
---
Signif. codes:  0 '***' 0.001 '**' 0.01 '*' 0.05 '.' 0.1 ' ' 1

Dispersion: 0.1936
```

Model residuals

Statistic	Value
Sample skewness	0.004093
Sample excess kurtosis	0.3483
Passed Shapiro Wilk normality test	Yes (p-value = 0.4491 > 0.05)

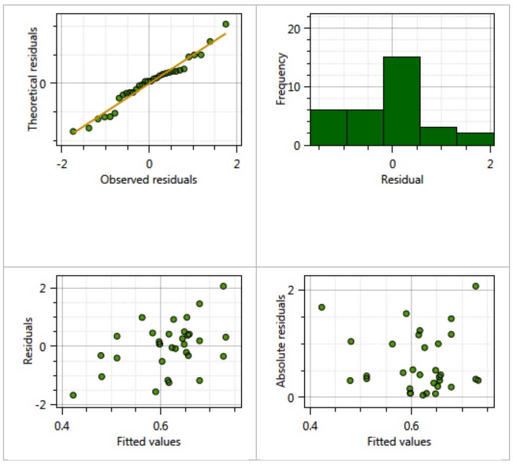

Analysis average velocity H1 (diff. Zone 1 - Zone 2)

**Analysis model**	Generalized linear mixed model with dispersion factor, formula=cbind(Average_velocity_H1_Zone_1,Average_velocity_H1_Zone_2) ~ 1 + (1|Genotype_Zone_1:Plant_Zone_1) + (1|Genotype_Zone_2:Plant_Zone_2)
**Transformation**	Logit

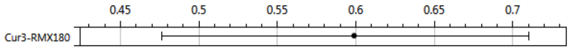

Confidence intervals average velocity H1 (diff. Zone 1 - Zone 2)

Genotype Zone 1	Genotype Zone 2	Mean	Lower 95% CL	Upper 95% CL	Group
Cur3	RMX180	0.5989	0.4762	0.7104	a

Model summary

```
Linear mixed model fit by REML. t-tests use Satterthwaite's method ['lmerModLmerTest']
Formula: ziFormula
   Data: data
Weights: wi

REML criterion at convergence: 90.1

Scaled residuals:
    Min      1Q  Median      3Q     Max
-1.9801 -0.5415  0.2071  0.6024  2.1941

Random effects:
 Groups                        Name        Variance Std.Dev.
 Genotype_Zone_1:Plant_Zone_1 (Intercept) 0.04567  0.2137
 Genotype_Zone_2:Plant_Zone_2 (Intercept) 0.32453  0.5697
 Residual                                0.08779  0.2963
Number of obs: 36, groups:  Genotype_Zone_1:Plant_Zone_1, 10; Genotype_Zone_2:Plant_Zone_2, 10

Fixed effects:
            Estimate Std. Error     df t value Pr(>|t|)
(Intercept)   0.4010     0.2227 9.9656   1.801    0.102

Dispersion: 0.2963
```

Model residuals

Statistic	Value
Sample skewness	-0.1612
Sample excess kurtosis	0.2992
Passed Shapiro Wilk normality test	Yes (p-value = 0.7532 > 0.05)

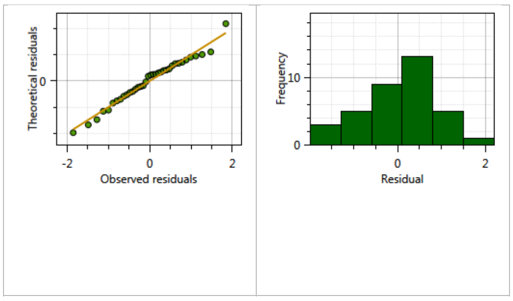

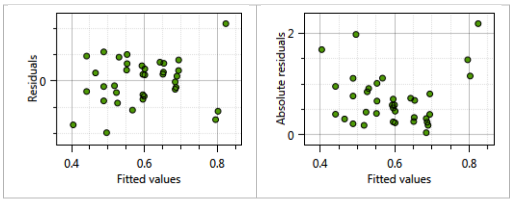

Analysis average velocity H2 (diff. Zone 1 - Zone 2)

**Analysis model**	Generalized linear mixed model with dispersion factor, formula=cbind(Average_velocity_H2_Zone_1,Average_velocity_H2_Zone_2) ~ 1 + (1|Genotype_Zone_1:Plant_Zone_1) + (1|Genotype_Zone_2:Plant_Zone_2)
**Transformation**	Logit

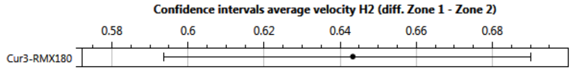

Confidence intervals average velocity H2 (diff. Zone 1 - Zone 2)

Genotype Zone 1	Genotype Zone 2	Mean	Lower 95% CL	Upper 95% CL	Group
Cur3	RMX180	0.6433	0.5935	0.6902	a

Model summary

```
Linear mixed model fit by REML. t-tests use Satterthwaite's method ['lmerModLmerTest']
Formula: ziFormula
   Data: data
Weights: wi

REML criterion at convergence: 68.1

Scaled residuals: 
    Min      1Q  Median      3Q     Max 
-2.5512 -0.6119  0.2946  0.7444  1.5983 

Random effects:
 Groups                      Name        Variance Std.Dev.
 Genotype_Zone_1:Plant_Zone_1 (Intercept) 0.00000  0.0000  
 Genotype_Zone_2:Plant_Zone_2 (Intercept) 0.00000  0.0000  
 Residual                                0.07124  0.2669  
Number of obs: 34, groups:  Genotype_Zone_1:Plant_Zone_1, 10; Genotype_Zone_2:Plant_Zone_2, 10

Fixed effects:
            Estimate Std. Error      df t value Pr(>|t|)    
(Intercept)   0.5897     0.1038 33.0000   5.682 2.46e-06 ***
---
Signif. codes:  0 '***' 0.001 '**' 0.01 '*' 0.05 '.' 0.1 ' ' 1

Dispersion: 0.2669
```

Model residuals

Statistic	Value
Sample skewness	-0.7948
Sample excess kurtosis	0.12
Passed Shapiro Wilk normality test	Yes (p-value = 0.06692 > 0.05)

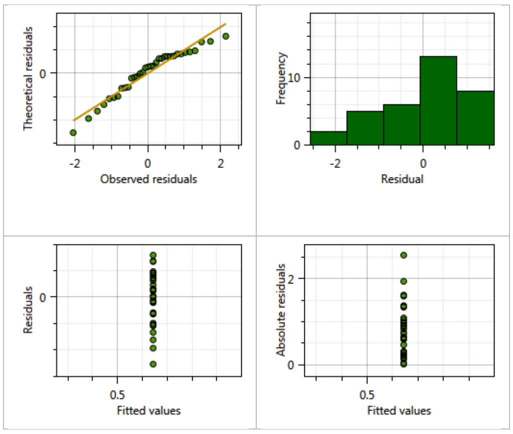

Analysis average velocity H3 (diff. Zone 1 - Zone 2)

**Analysis model**	Generalized linear mixed model with dispersion factor, formula=cbind(Average_velocity_H3_Zone_1,Average_velocity_H3_Zone_2) ~ 1 + (1|Genotype_Zone_1:Plant_Zone_1) + (1|Genotype_Zone_2:Plant_Zone_2)
**Transformation**	Logit

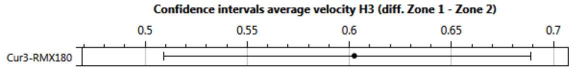

Confidence intervals average velocity H3 (diff. Zone 1 - Zone 2)

Genotype Zone 1	Genotype Zone 2	Mean	Lower 95% CL	Upper 95% CL	Group
Cur3	RMX180	0.6025	0.509	0.689	a

Model summary

```
Linear mixed model fit by REML. t-tests use Satterthwaite's method ['lmerModLmerTest']
Formula: ziFormula
   Data: data
Weights: wi

REML criterion at convergence: 73.8

Scaled residuals: 
     Min       1Q   Median       3Q      Max 
-1.86057 -0.69197  0.04667  0.47987  2.47300 

Random effects:
 Groups                      Name        Variance Std.Dev.
 Genotype_Zone_1:Plant_Zone_1 (Intercept) 0.02191  0.1480  
 Genotype_Zone_2:Plant_Zone_2 (Intercept) 0.11535  0.3396  
 Residual                                0.07441  0.2728  
Number of obs: 33, groups:  Genotype_Zone_1:Plant_Zone_1, 10; Genotype_Zone_2:Plant_Zone_2, 10

Fixed effects:
            Estimate Std. Error     df t value Pr(>|t|)  
(Intercept)   0.4157     0.1597 6.7893   2.604   0.0362 *
---
Signif. codes:  0 '***' 0.001 '**' 0.01 '*' 0.05 '.' 0.1 ' ' 1
```

```
Dispersion: 0.2728
```

Model residuals

Statistic	Value
Sample skewness	0.4513
Sample excess kurtosis	0.6495
Passed Shapiro Wilk normality test	Yes (p-value = 0.8346 > 0.05)

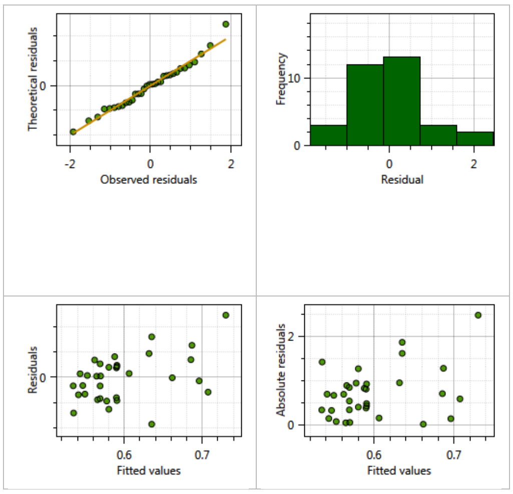

Analysis average velocity H4 (diff. Zone 1 - Zone 2)

**Analysis model**	Generalized linear mixed model with dispersion factor, formula=cbind(Average_velocity_H4_Zone_1,Average_velocity_H4_Zone_2) ~ 1 + (1|Genotype_Zone_1:Plant_Zone_1) + (1|Genotype_Zone_2:Plant_Zone_2)
**Transformation**	Logit

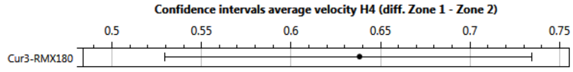

Confidence intervals average velocity H4 (diff. Zone 1 - Zone 2)

Genotype Zone 1	Genotype Zone 2	Mean	Lower 95% CL	Upper 95% CL	Group
Cur3	RMX180	0.6383	0.5293	0.7346	a

Model summary

```
Linear mixed model fit by REML. t-tests use Satterthwaite's method ['lmerModLmerTest']
Formula: ziFormula
   Data: data
Weights: wi

REML criterion at convergence: 54.9

Scaled residuals:
     Min       1Q   Median       3Q      Max
-1.43169 -0.70729 -0.04817  0.79386  1.45180

Random effects:
 Groups                      Name        Variance Std.Dev.
 Genotype_Zone_1:Plant_Zone_1 (Intercept) 0.0000   0.0000
 Genotype_Zone_2:Plant_Zone_2 (Intercept) 0.3022   0.5497
 Residual                                0.0367   0.1916
Number of obs: 28, groups:  Genotype_Zone_1:Plant_Zone_1, 10; Genotype_Zone_2:Plant_Zone_2, 10

Fixed effects:
            Estimate Std. Error     df t value Pr(>|t|)
(Intercept)   0.5678     0.1976 8.5666   2.874   0.0193 *
---
Signif. codes:  0 '***' 0.001 '**' 0.01 '*' 0.05 '.' 0.1 ' ' 1

Dispersion: 0.1916
```

Model residuals

Statistic	Value
Sample skewness	0.0772
Sample excess kurtosis	-1.253
Passed Shapiro Wilk normality test	Yes (p-value = 0.1609 > 0.05)

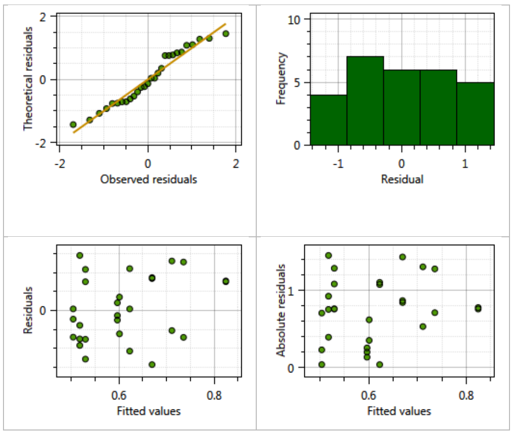

Analysis average velocity H5 (diff. Zone 1 - Zone 2)

**Analysis model**	Generalized linear mixed model with dispersion factor, formula=cbind(Average_velocity_H5_Zone_1,Average_velocity_H5_Zone_2) ~ 1 + (1|Genotype_Zone_1:Plant_Zone_1) + (1|Genotype_Zone_2:Plant_Zone_2)
**Transformation**	Logit

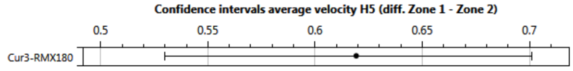

Confidence intervals average velocity H5 (diff. Zone 1 - Zone 2)

Genotype Zone 1	Genotype Zone 2	Mean	Lower 95% CL	Upper 95% CL	Group
Cur3	RMX180	0.6192	0.5297	0.7012	a

Model summary

```
Linear mixed model fit by REML. t-tests use Satterthwaite's method ['lmerModLmerTest']
Formula: ziFormula
   Data: data
Weights: wi

REML criterion at convergence: 69.9

Scaled residuals:
     Min       1Q   Median       3Q      Max
-1.92297 -0.74933  0.02265  0.93100  1.45334

Random effects:
 Groups                      Name        Variance  Std.Dev.
 Genotype_Zone_1:Plant_Zone_1 (Intercept) 1.793e-17 4.234e-09
 Genotype_Zone_2:Plant_Zone_2 (Intercept) 1.356e-01 3.682e-01
 Residual                                6.217e-02 2.493e-01
Number of obs: 31, groups:  Genotype_Zone_1:Plant_Zone_1, 10; Genotype_Zone_2:Plant_Zone_2, 10

Fixed effects:
            Estimate Std. Error     df t value Pr(>|t|)
(Intercept)   0.4860     0.1618 8.8542   3.003   0.0151 *
---
Signif. codes:  0 '***' 0.001 '**' 0.01 '*' 0.05 '.' 0.1 ' ' 1

Dispersion: 0.2493
```

Model residuals

Statistic	Value
Sample skewness	-0.1819
Sample excess kurtosis	-0.904
Passed Shapiro Wilk normality test	Yes (p-value = 0.2958 > 0.05)

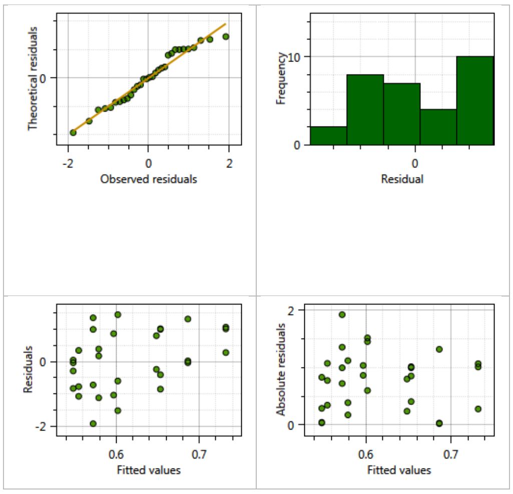

Analysis average velocity H6 (diff. Zone 1 - Zone 2)

**Analysis model**	Generalized linear mixed model with dispersion factor, formula=cbind(Average_velocity_H6_Zone_1,Average_velocity_H6_Zone_2) ~ 1 + (1|Genotype_Zone_1:Plant_Zone_1) + (1|Genotype_Zone_2:Plant_Zone_2)
**Transformation**	Logit

Confidence intervals average velocity H6 (diff. Zone 1 - Zone 2)

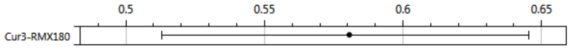

Confidence intervals average velocity H6 (diff. Zone 1 - Zone 2)

Genotype Zone 1	Genotype Zone 2	Mean	Lower 95% CL	Upper 95% CL	Group
Cur3	RMX180	0.5806	0.5128	0.6455	a

Model summary

```
Linear mixed model fit by REML. t-tests use Satterthwaite's method ['lmerModLmerTest']
Formula: ziFormula
   Data: data
Weights: wi

REML criterion at convergence: 43.8

Scaled residuals:
    Min      1Q  Median      3Q     Max
-1.3126 -0.5480 -0.0038  0.3286  2.1739

Random effects:
 Groups                      Name        Variance Std.Dev.
 Genotype_Zone_1:Plant_Zone_1 (Intercept) 0.06451  0.2540
 Genotype_Zone_2:Plant_Zone_2 (Intercept) 0.00000  0.0000
 Residual                                0.04136  0.2034
Number of obs: 26, groups:  Genotype_Zone_1:Plant_Zone_1, 10; Genotype_Zone_2:Plant_Zone_2, 10

Fixed effects:
            Estimate Std. Error      df t value Pr(>|t|)
(Intercept)   0.3253     0.1239 10.5634   2.626   0.0243 *
---
Signif. codes:  0 '***' 0.001 '**' 0.01 '*' 0.05 '.' 0.1 ' ' 1

Dispersion: 0.2034
```

Model residuals

Statistic	Value
Sample skewness	0.7925
Sample excess kurtosis	0.3862
Passed Shapiro Wilk normality test	Yes (p-value = 0.05105 > 0.05)

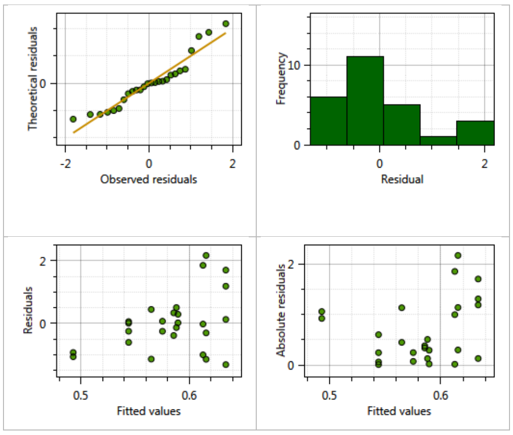

Analysis average velocity H7 (diff. Zone 1 - Zone 2)

**Analysis model**	Generalized linear mixed model with dispersion factor, formula=cbind(Average_velocity_H7_Zone_1,Average_velocity_H7_Zone_2) ~ 1 + (1|Genotype_Zone_1:Plant_Zone_1) + (1|Genotype_Zone_2:Plant_Zone_2)
**Transformation**	Logit

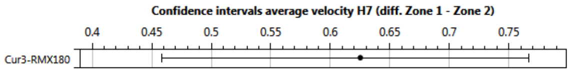

Confidence intervals average velocity H7 (diff. Zone 1 - Zone 2)

Genotype Zone 1	Genotype Zone 2	Mean	Lower 95% CL	Upper 95% CL	Group
Cur3	RMX180	0.6252	0.4581	0.7669	a

Model summary

```
Linear mixed model fit by REML. t-tests use Satterthwaite's method ['lmerModLmerTest']
Formula: ziFormula
   Data: data
Weights: wi

REML criterion at convergence: 77

Scaled residuals:
     Min       1Q   Median       3Q      Max
-1.44866 -0.70709  0.02056  0.66250  1.92638

Random effects:
 Groups                      Name        Variance Std.Dev.
 Genotype_Zone_2:Plant_Zone_2 (Intercept) 0.61434  0.7838
 Genotype_Zone_1:Plant_Zone_1 (Intercept) 0.00000  0.0000
 Residual                                 0.08907  0.2985
Number of obs: 28, groups:  Genotype_Zone_2:Plant_Zone_2, 10; Genotype_Zone_1:Plant_Zone_1, 9

Fixed effects:
            Estimate Std. Error     df t value Pr(>|t|)
(Intercept)   0.5115     0.2935 7.8231   1.742     0.12

Dispersion: 0.2985
```

Model residuals

Statistic	Value
Sample skewness	0.197
Sample excess kurtosis	-0.5832
Passed Shapiro Wilk normality test	Yes (p-value = 0.7284 > 0.05)

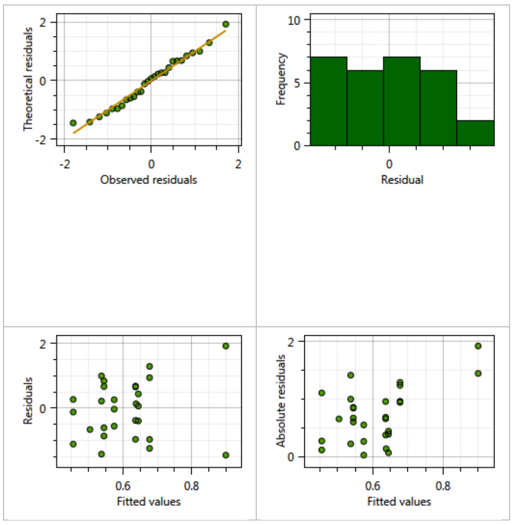

Estimated distance moved per zone

Selected zones	Zone 1, Zone 2
Data transformation	Natural logarithm
Analysis	Zone difference analysis

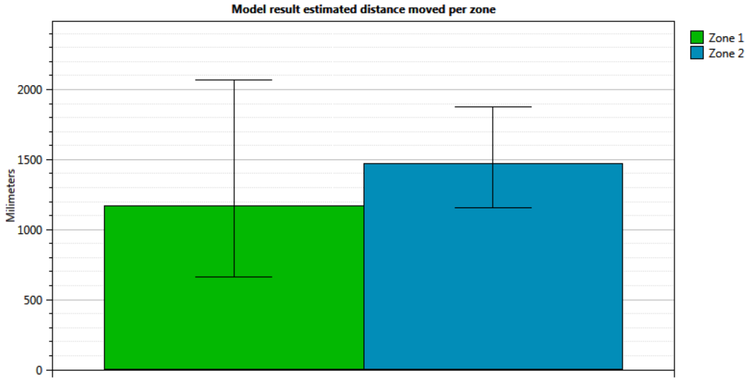

Results difference tests Zone 1 - Zone 2: p values and 95% confidence intervals of the difference on the transformed scale for each statistic.

Behaviour statistic	Cur3-RMX180
Estimated distance moved (diff. Zone 1 - Zone 2)	p=0.118
	[-0.263, 0.0356]

The model predictions and 95% confidence intervals for each statistic.

Statistic	Cur3-RMX180	Remark
Estimated distance moved (Zone 1)	1.17E+03	CR
	[662, 2.07E+03]	
Estimated distance moved (Zone 2)	1.47E+03	CR
	[1.16E+03, 1.87E+03]	

CR = Check residuals

Data summary

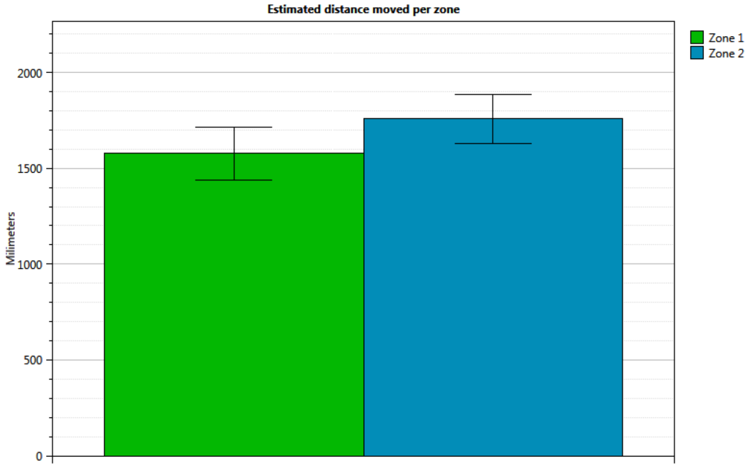

Genotype Zone 1	Genotype Zone 2	Genotype Zone 3	Mean Zone 1	StdErr Zone 1	Mean Zone 2	StdErr Zone 2
Cur3	RMX180	Neutral	1577.46	139.13	1758.24	128.9

Analysis estimated distance moved (Zone 1)

**Analysis model**	Linear mixed model fit by REML: Estimated_distance_moved_Zone_1 ~ 1 + (1|Genotype_Zone_1:Plant_Zone_1) + (1|Genotype_Zone_2:Plant_Zone_2)
**Transformation**	Natural logarithm

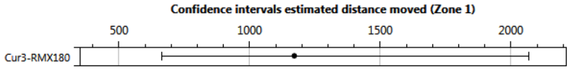

Confidence intervals estimated distance moved (Zone 1)

Genotype Zone 1	Genotype Zone 2	Mean	Lower 95% CL	Upper 95% CL	Group
Cur3	RMX180	1171	662.3	2070	a

Model summary

```
Linear mixed model fit by REML. t-tests use Satterthwaite's method ['lmerModLmerTest']
Formula: Estimated_distance_moved_Zone_1 ~ 1 + (1 | Genotype_Zone_1:Plant_Zone_1) +      (1 |
Genotype_Zone_2:Plant_Zone_2)
   Data: data

REML criterion at convergence: 128.1

Scaled residuals:
    Min      1Q  Median      3Q     Max
-4.9667 -0.0176  0.1762  0.3949  0.7958

Random effects:
 Groups                       Name        Variance  Std.Dev.
 Genotype_Zone_1:Plant_Zone_1 (Intercept) 1.685e-01 4.105e-01
 Genotype_Zone_2:Plant_Zone_2 (Intercept) 3.053e-13 5.526e-07
 Residual                                 1.559e+00 1.248e+00
Number of obs: 38, groups:  Genotype_Zone_1:Plant_Zone_1, 10; Genotype_Zone_2:Plant_Zone_2, 10

Fixed effects:
            Estimate Std. Error     df t value Pr(>|t|)
(Intercept)   7.0654     0.2408 6.9897   29.34 1.41e-08 ***
---
Signif. codes:  0 '***' 0.001 '**' 0.01 '*' 0.05 '.' 0.1 ' ' 1
```

Model residuals

Statistic	Value
Sample skewness	-4.077
Sample excess kurtosis	19.77
Passed Shapiro Wilk normality test	No (p-value = 1.791E-09 < 0.05)

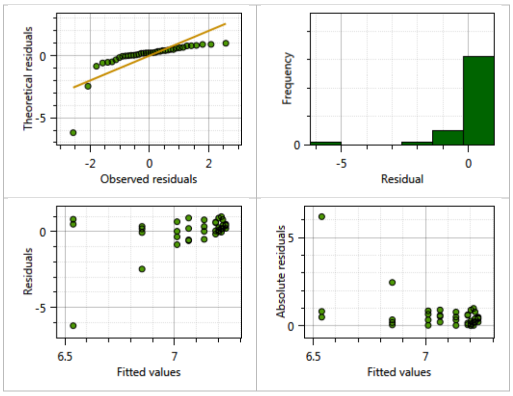

Analysis estimated distance moved (Zone 2)

**Analysis model**	Linear mixed model fit by REML: Estimated_distance_moved_Zone_2 ~ 1 + (1|Genotype_Zone_1:Plant_Zone_1) + (1|Genotype_Zone_2:Plant_Zone_2)
**Transformation**	Natural logarithm

Confidence intervals estimated distance moved (Zone 2)

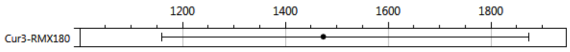

Confidence intervals estimated distance moved (Zone 2)

Genotype Zone 1	Genotype Zone 2	Mean	Lower 95% CL	Upper 95% CL	Group
Cur3	RMX180	1474	1159	1874	a

Model summary

```
Linear mixed model fit by REML. t-tests use Satterthwaite's method ['lmerModLmerTest']
Formula: Estimated_distance_moved_Zone_2 ~ 1 + (1 | Genotype_Zone_1:Plant_Zone_1) +      (1 |
Genotype_Zone_2:Plant_Zone_2)
   Data: data

REML criterion at convergence: 88.8

Scaled residuals:
    Min      1Q  Median      3Q     Max
-3.2494 -0.1954  0.1764  0.6102  1.2767

Random effects:
 Groups                       Name        Variance Std.Dev.
 Genotype_Zone_1:Plant_Zone_1 (Intercept) 0.0000   0.0000
 Genotype_Zone_2:Plant_Zone_2 (Intercept) 0.0000   0.0000
 Residual                                 0.5495   0.7413
Number of obs: 39, groups:  Genotype_Zone_1:Plant_Zone_1, 10; Genotype_Zone_2:Plant_Zone_2, 10

Fixed effects:
            Estimate Std. Error      df t value Pr(>|t|)
(Intercept)   7.2957     0.1187 38.0000   61.46   <2e-16 ***
---
Signif. codes:  0 '***' 0.001 '**' 0.01 '*' 0.05 '.' 0.1 ' ' 1
```

Model residuals

Statistic	Value
Sample skewness	-2.114
Sample excess kurtosis	4.807
Passed Shapiro Wilk normality test	No (p-value = 1.675E-06 < 0.05)

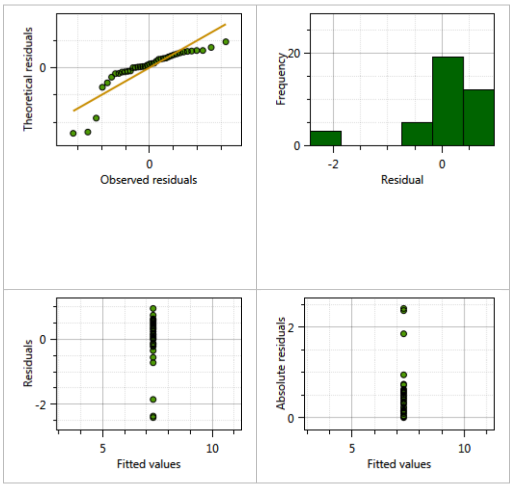

Analysis estimated distance moved (diff. Zone 1 - Zone 2)

**Analysis model**	Generalized linear mixed model with dispersion factor, formula=cbind(Estimated_distance_moved_Zone_1,Estimated_distance_moved_Zone_2) ~ 1 + (1|Genotype_Zone_1:Plant_Zone_1) + (1|Genotype_Zone_2:Plant_Zone_2)
**Transformation**	Logit

Confidence intervals estimated distance moved (diff. Zone 1 - Zone 2)

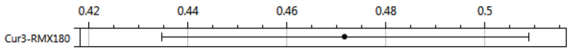

Confidence intervals estimated distance moved (diff. Zone 1 - Zone 2)

Genotype Zone 1	Genotype Zone 2	Mean	Lower 95% CL	Upper 95% CL	Group
Cur3	RMX180	0.4716	0.4346	0.5089	a

Model summary

```
Linear mixed model fit by REML. t-tests use Satterthwaite's method ['lmerModLmerTest']
Formula: ziFormula
   Data: data
Weights: wi

REML criterion at convergence: 40.1

Scaled residuals:
```

```
    Min      1Q  Median      3Q     Max 
-1.7858 -0.7553 -0.1765  0.6902  1.8209 

Random effects:
 Groups                      Name        Variance Std.Dev.
 Genotype_Zone_1:Plant_Zone_1 (Intercept)  0.01347 0.1161  
 Genotype_Zone_2:Plant_Zone_2 (Intercept)  0.00000 0.0000  
 Residual                              92.81723 9.6342  
Number of obs: 39, groups:  Genotype_Zone_1:Plant_Zone_1, 10; Genotype_Zone_2:Plant_Zone_2, 10

Fixed effects:
            Estimate Std. Error       df t value Pr(>|t|)
(Intercept) -0.11372    0.06544  8.52961  -1.738    0.118

Dispersion: 9.634
```

Model residuals

Statistic	Value
Sample skewness	0.04159
Sample excess kurtosis	-0.8493
Passed Shapiro Wilk normality test	Yes (p-value = 0.5684 > 0.05)

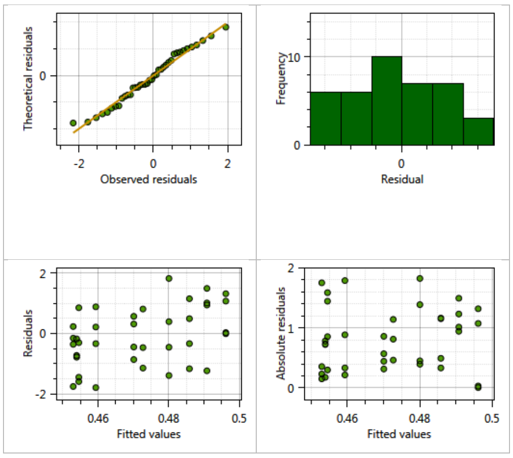

Estimated distance moved per zone per hour

Selected hours	0, 1, 2, 3, 4, 5, 6, 7
Selected zones	Zone 1, Zone 2
Data transformation	Natural logarithm
Analysis	Zone difference analysis

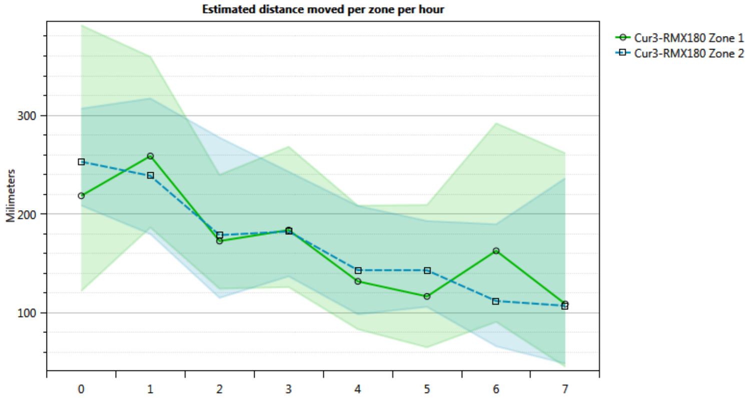

Results difference tests Zone 1 - Zone 2: p values and 95% confidence intervals of the difference on the transformed scale for each statistic.

Behaviour statistic	Cur3-RMX180
Estimated distance moved H0 (diff. Zone 1 - Zone 2)	p=0.771<br>[-0.385, 0.294]
Estimated distance moved H1 (diff. Zone 1 - Zone 2)	p=0.309<br>[-0.0891, 0.273]
Estimated distance moved H2 (diff. Zone 1 - Zone 2)	p=0.422<br>[-0.386, 0.184]
Estimated distance moved H3 (diff. Zone 1 - Zone 2)	p=0.226<br>[-0.295, 0.0718]
Estimated distance moved H4 (diff. Zone 1 - Zone 2)	p=0.076<br>[-0.57, 0.0356]
Estimated distance moved H5 (diff. Zone 1 - Zone 2)	p=0.0637<br>[-0.645, 0.0246]
Estimated distance moved H6 (diff. Zone 1 - Zone 2)	p=0.615<br>[-0.309, 0.185]
Estimated distance moved H7 (diff. Zone 1 - Zone 2)	p=0.121<br>[-0.709, 0.1]

The model predictions and 95% confidence intervals for each statistic.

Statistic	Cur3-RMX180	Remark
Estimated distance moved (H0 - Zone 1)	218<br>[122, 391]	CR
Estimated distance moved (H0 - Zone 2)	253<br>[209, 307]	CR
Estimated distance moved (H1 - Zone 1)	259<br>[186, 359]	CR
Estimated distance moved (H1 - Zone 2)	239<br>[180, 317]	
Estimated distance moved (H2 - Zone 1)	173<br>[124, 240]	
Estimated distance moved (H2 - Zone 2)	179<br>[115, 277]	CR
Estimated distance moved (H3 - Zone 1)	184<br>[126, 268]	CR
Estimated distance moved (H3 - Zone 2)	182<br>[137, 243]	CR
Estimated distance moved (H4 - Zone 1)	132<br>[83.3, 209]	CR
Estimated distance moved (H4 - Zone 2)	143	CR

Statistic	Cur3-RMX180	Remark
	[98.4, 208]	
Estimated distance moved (H5 - Zone 1)	117	CR
	[65, 209]	
Estimated distance moved (H5 - Zone 2)	143	CR
	[106, 193]	
Estimated distance moved (H6 - Zone 1)	163	CR
	[90.8, 292]	
Estimated distance moved (H6 - Zone 2)	112	CR
	[66.1, 190]	
Estimated distance moved (H7 - Zone 1)	109	CR
	[45.3, 262]	
Estimated distance moved (H7 - Zone 2)	107	
	[48.5, 236]	

CR = Check residuals

Data summary

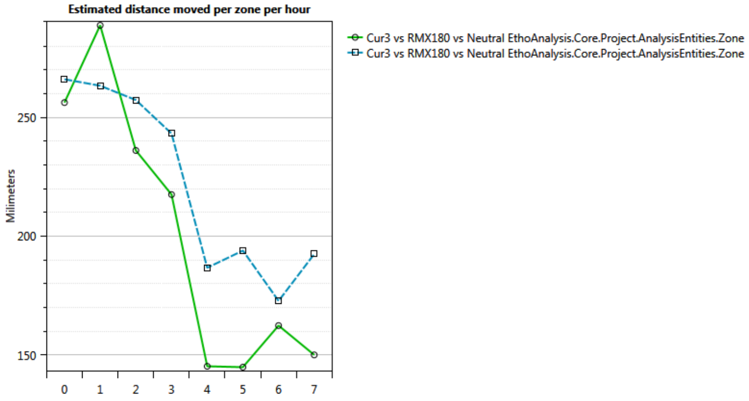

Genotype Zone 1	Genotype Zone 2	Genotype Zone 3	Mean H0 - Zone 1	StdErr H0 - Zone 1	Mean H0 - Zone 2	StdErr H0 - Zone 2	Mean H1 - Zone 1	StdErr H1 - Zone 1	Mean H1 - Zone 2	StdErr H1 - Zone 2	Mean H2 - Zone 1	StdErr H2 - Zone 1	Mean H2 - Zone 2	StdErr H2 - Zone 2	Mean H3 - Zone 1	StdErr H3 - Zone 1	Mean H3 - Zone 2	StdErr H3 - Zone 2	Mean H4 - Zone 1	StdErr H4 - Zone 1	Mean H4 - Zone 2	StdErr H4 - Zone 2	Mean H5 - Zone 1	StdErr H5 - Zone 1	Mean H5 - Zone 2	StdErr H5 - Zone 2	Mean H6 - Zone 1	StdErr H6 - Zone 1	Mean H6 - Zone 2	St H[illegible] Z[illegible] 2
Cur3	RMX180	Neutral	256.21	31.53	266.01	24.32	288.72	27.56	263.29	23.91	236.05	37.32	257.28	36.3	217.5	32.08	243.18	25.53	145.21	21.94	186.67	21.44	144.85	23.12	193.91	21.15	162.35	29.98	172.73	24[illegible]

Analysis estimated distance moved (H0 - Zone 1)

**Analysis model**	Linear mixed model fit by REML: Estimated_distance_moved_H0_Zone_1 ~ 1 + (1|Genotype_Zone_1:Plant_Zone_1) + (1|Genotype_Zone_2:Plant_Zone_2)
**Transformation**	Natural logarithm

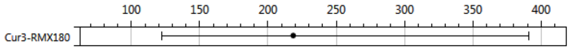

Confidence intervals estimated distance moved (H0 - Zone 1)

Genotype Zone 1	Genotype Zone 2	Mean	Lower 95% CL	Upper 95% CL	Group
Cur3	RMX180	218.5	122	391.2	a

Model summary

```
Linear mixed model fit by REML. t-tests use Satterthwaite's method ['lmerModLmerTest']
Formula: Estimated_distance_moved_H0_Zone_1 ~ 1 + (1 | Genotype_Zone_1:Plant_Zone_1) +      (1 |
Genotype_Zone_2:Plant_Zone_2)
   Data: data

REML criterion at convergence: 100.3

Scaled residuals:
    Min      1Q  Median      3Q     Max
-3.9617 -0.1803  0.1699  0.4781  1.1422

Random effects:
 Groups                       Name        Variance Std.Dev.
 Genotype_Zone_1:Plant_Zone_1 (Intercept) 0.115    0.3391
 Genotype_Zone_2:Plant_Zone_2 (Intercept) 0.000    0.0000
 Residual                                 1.113    1.0551
Number of obs: 33, groups:  Genotype_Zone_1:Plant_Zone_1, 10; Genotype_Zone_2:Plant_Zone_2, 10

Fixed effects:
            Estimate Std. Error     df t value Pr(>|t|)
(Intercept)   5.3867     0.2148 4.2566   25.08  8.7e-06 ***
---
Signif. codes:  0 '***' 0.001 '**' 0.01 '*' 0.05 '.' 0.1 ' ' 1
```

Model residuals

Statistic	Value
Sample skewness	-2.663
Sample excess kurtosis	9.052
Passed Shapiro Wilk normality test	No (p-value = 2.432E-06 < 0.05)

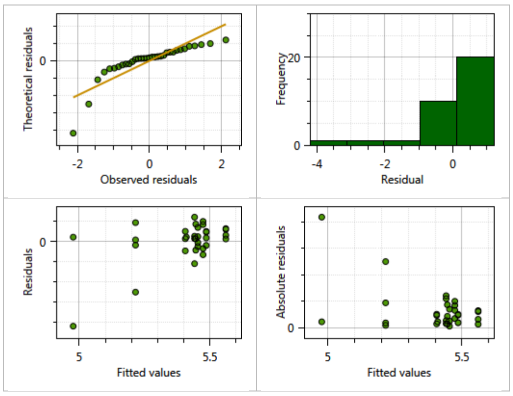

Analysis estimated distance moved (H0 - Zone 2)

Analysis model	Linear mixed model fit by REML: Estimated_distance_moved_H0_Zone_2 ~ 1 + (1|Genotype_Zone_1:Plant_Zone_1) + (1|Genotype_Zone_2:Plant_Zone_2)
Transformation	Natural logarithm

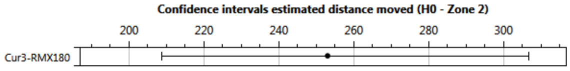

Confidence intervals estimated distance moved (H0 - Zone 2)

Genotype Zone 1	Genotype Zone 2	Mean	Lower 95% CL	Upper 95% CL	Group
Cur3	RMX180	253	208.6	306.8	a

Model summary

```
Linear mixed model fit by REML. t-tests use Satterthwaite's method ['lmerModLmerTest']
Formula: Estimated_distance_moved_H0_Zone_2 ~ 1 + (1 | Genotype_Zone_1:Plant_Zone_1) +      (1 |
Genotype_Zone_2:Plant_Zone_2)
   Data: data

REML criterion at convergence: 63.6

Scaled residuals: 
    Min      1Q  Median      3Q     Max 
-3.1187 -0.4613  0.2648  0.6072  1.8444 

Random effects:
 Groups                       Name        Variance Std.Dev.
 Genotype_Zone_1:Plant_Zone_1 (Intercept) 0.0000   0.00    
 Genotype_Zone_2:Plant_Zone_2 (Intercept) 0.0000   0.00    
 Residual                                 0.3249   0.57    
Number of obs: 36, groups:  Genotype_Zone_1:Plant_Zone_1, 10; Genotype_Zone_2:Plant_Zone_2, 10

Fixed effects:
            Estimate Std. Error     df t value Pr(>|t|)    
(Intercept)    5.533      0.095 35.000   58.25   <2e-16 ***
---
Signif. codes:  0 '***' 0.001 '**' 0.01 '*' 0.05 '.' 0.1 ' ' 1
```

Model residuals

Statistic	Value
Sample skewness	-1.263
Sample excess kurtosis	2.618
Passed Shapiro Wilk normality test	No (p-value = 0.006754 < 0.05)

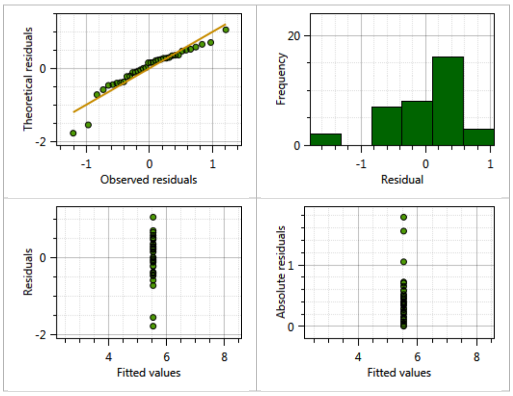

Analysis estimated distance moved (H1 - Zone 1)

Analysis model	Linear mixed model fit by REML: Estimated_distance_moved_H1_Zone_1 ~ 1 + (1|Genotype_Zone_1:Plant_Zone_1) + (1|Genotype_Zone_2:Plant_Zone_2)
Transformation	Natural logarithm

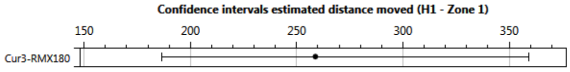

Confidence intervals estimated distance moved (H1 - Zone 1)

Genotype Zone 1	Genotype Zone 2	Mean	Lower 95% CL	Upper 95% CL	Group
Cur3	RMX180	258.8	186.4	359.3	a

Model summary

```
Linear mixed model fit by REML. t-tests use Satterthwaite's method ['lmerModLmerTest']
Formula: Estimated_distance_moved_H1_Zone_1 ~ 1 + (1 | Genotype_Zone_1:Plant_Zone_1) +      (1 |
Genotype_Zone_2:Plant_Zone_2)
   Data: data

REML criterion at convergence: 86.8

Scaled residuals: 
    Min      1Q  Median      3Q     Max 
-4.5739 -0.2362  0.1018  0.6252  1.3282 

Random effects:
 Groups                       Name        Variance Std.Dev.
 Genotype_Zone_1:Plant_Zone_1 (Intercept) 0.00000  0.0000  
 Genotype_Zone_2:Plant_Zone_2 (Intercept) 0.03107  0.1763  
 Residual                                 0.60489  0.7777  
Number of obs: 36, groups:  Genotype_Zone_1:Plant_Zone_1, 10; Genotype_Zone_2:Plant_Zone_2, 10

Fixed effects:
            Estimate Std. Error     df t value Pr(>|t|)    
(Intercept)   5.5560     0.1413 7.6656   39.33 4.02e-10 ***
---
Signif. codes:  0 '***' 0.001 '**' 0.01 '*' 0.05 '.' 0.1 ' ' 1
```

Model residuals

Statistic	Value
Sample skewness	-2.99
Sample excess kurtosis	13.26
Passed Shapiro Wilk normality test	No (p-value = 1.21E-06 < 0.05)

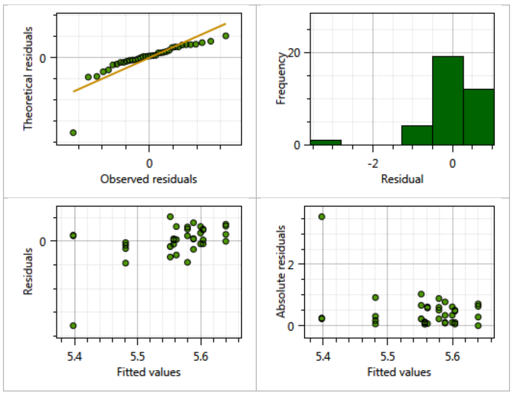

Analysis estimated distance moved (H1 - Zone 2)

**Analysis model**	Linear mixed model fit by REML: Estimated_distance_moved_H1_Zone_2 ~ 1 + (1|Genotype_Zone_1:Plant_Zone_1) + (1|Genotype_Zone_2:Plant_Zone_2)
**Transformation**	Natural logarithm

Confidence intervals estimated distance moved (H1 - Zone 2)

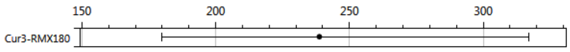

Confidence intervals estimated distance moved (H1 - Zone 2)

Genotype Zone 1	Genotype Zone 2	Mean	Lower 95% CL	Upper 95% CL	Group
Cur3	RMX180	238.7	179.7	317.1	a

Model summary

```
Linear mixed model fit by REML. t-tests use Satterthwaite's method ['lmerModLmerTest']
Formula: Estimated_distance_moved_H1_Zone_2 ~ 1 + (1 | Genotype_Zone_1:Plant_Zone_1) +      (1 |
Genotype_Zone_2:Plant_Zone_2)
   Data: data

REML criterion at convergence: 61.9

Scaled residuals:
     Min       1Q   Median       3Q      Max
-2.15986 -0.41324  0.06628  0.79050  1.54690

Random effects:
 Groups                       Name        Variance Std.Dev.
 Genotype_Zone_1:Plant_Zone_1 (Intercept) 0.00000  0.0000
 Genotype_Zone_2:Plant_Zone_2 (Intercept) 0.09136  0.3023
 Residual                                 0.23711  0.4869
Number of obs: 37, groups:  Genotype_Zone_1:Plant_Zone_1, 10; Genotype_Zone_2:Plant_Zone_2, 10

Fixed effects:
            Estimate Std. Error     df t value Pr(>|t|)
(Intercept)   5.4754     0.1249 8.7323   43.83 1.53e-11 ***
---
Signif. codes:  0 '***' 0.001 '**' 0.01 '*' 0.05 '.' 0.1 ' ' 1
```

Model residuals

Statistic	Value
Sample skewness	-0.5353
Sample excess kurtosis	-0.2199
Passed Shapiro Wilk normality test	Yes (p-value = 0.2313 > 0.05)

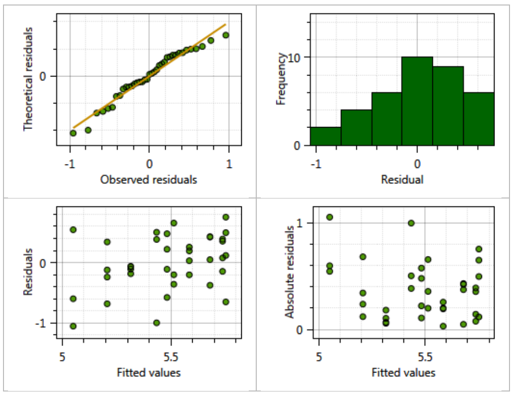

Analysis estimated distance moved (H2 - Zone 1)

**Analysis model**	Linear mixed model fit by REML: Estimated_distance_moved_H2_Zone_1 ~ 1 + (1|Genotype_Zone_1:Plant_Zone_1) + (1|Genotype_Zone_2:Plant_Zone_2)
**Transformation**	Natural logarithm

Confidence intervals estimated distance moved (H2 - Zone 1)

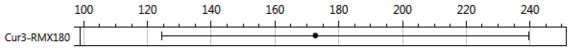

Confidence intervals estimated distance moved (H2 - Zone 1)

Genotype Zone 1	Genotype Zone 2	Mean	Lower 95% CL	Upper 95% CL	Group
Cur3	RMX180	172.7	124.4	239.7	a

Model summary

```
Linear mixed model fit by REML. t-tests use Satterthwaite's method ['lmerModLmerTest']
Formula: Estimated_distance_moved_H2_Zone_1 ~ 1 + (1 | Genotype_Zone_1:Plant_Zone_1) +      (1 |
Genotype_Zone_2:Plant_Zone_2)
   Data: data

REML criterion at convergence: 100.8

Scaled residuals:
     Min       1Q   Median       3Q      Max
-2.59146 -0.53581 -0.07171  0.69220  1.84331

Random effects:
 Groups                       Name        Variance  Std.Dev.
 Genotype_Zone_1:Plant_Zone_1 (Intercept) 6.620e-18 2.573e-09
 Genotype_Zone_2:Plant_Zone_2 (Intercept) 0.000e+00 0.000e+00
```

```
 Residual                                  9.404e-01 9.697e-01
Number of obs: 36, groups:  Genotype_Zone_1:Plant_Zone_1, 10; Genotype_Zone_2:Plant_Zone_2, 10

Fixed effects:
            Estimate Std. Error      df t value Pr(>|t|)
(Intercept)   5.1513     0.1616 35.0000   31.87   <2e-16 ***
---
Signif. codes:  0 '***' 0.001 '**' 0.01 '*' 0.05 '.' 0.1 ' ' 1
```

Model residuals

Statistic	Value
Sample skewness	-0.5504
Sample excess kurtosis	0.5751
Passed Shapiro Wilk normality test	Yes (p-value = 0.2346 > 0.05)

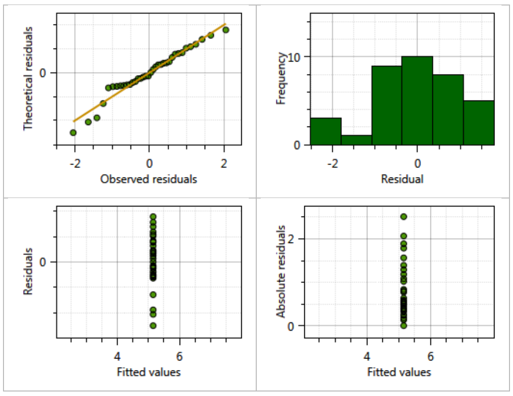

Analysis estimated distance moved (H2 - Zone 2)

Analysis model	Linear mixed model fit by REML: Estimated_distance_moved_H2_Zone_2 ~ 1 + (1|Genotype_Zone_1:Plant_Zone_1) + (1|Genotype_Zone_2:Plant_Zone_2)
Transformation	Natural logarithm

Confidence intervals estimated distance moved (H2 - Zone 2)

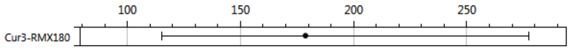

Confidence intervals estimated distance moved (H2 - Zone 2)

Genotype Zone 1	Genotype Zone 2	Mean	Lower 95% CL	Upper 95% CL	Group
Cur3	RMX180	178.7	115.1	277.5	a

Model summary

```
Linear mixed model fit by REML. t-tests use Satterthwaite's method ['lmerModLmerTest']
Formula: Estimated_distance_moved_H2_Zone_2 ~ 1 + (1 | Genotype_Zone_1:Plant_Zone_1) +      (1 |
Genotype_Zone_2:Plant_Zone_2)
   Data: data

REML criterion at convergence: 112.9

Scaled residuals:
    Min      1Q  Median      3Q     Max
-3.7003 -0.4566  0.1796  0.4341  1.4601

Random effects:
 Groups                       Name        Variance Std.Dev.
 Genotype_Zone_1:Plant_Zone_1 (Intercept) 0.04597  0.2144
 Genotype_Zone_2:Plant_Zone_2 (Intercept) 0.00000  0.0000
 Residual                                 1.17772  1.0852
Number of obs: 37, groups:  Genotype_Zone_1:Plant_Zone_1, 10; Genotype_Zone_2:Plant_Zone_2, 10

Fixed effects:
            Estimate Std. Error    df t value Pr(>|t|)
(Intercept)    5.186      0.191 8.037   27.15 3.41e-09 ***
---
Signif. codes:  0 '***' 0.001 '**' 0.01 '*' 0.05 '.' 0.1 ' ' 1
```

Model residuals

Statistic	Value
Sample skewness	-1.49
Sample excess kurtosis	4.306
Passed Shapiro Wilk normality test	No (p-value = 0.002968 < 0.05)

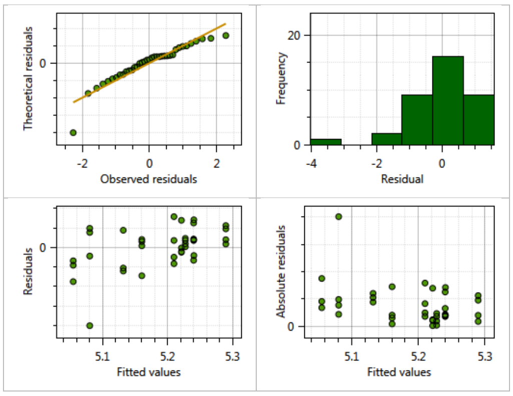

Analysis estimated distance moved (H3 - Zone 1)

Analysis model	Linear mixed model fit by REML: Estimated_distance_moved_H3_Zone_1 ~ 1 + (1|Genotype_Zone_1:Plant_Zone_1) + (1|Genotype_Zone_2:Plant_Zone_2)
Transformation	Natural logarithm

Confidence intervals estimated distance moved (H3 - Zone 1)

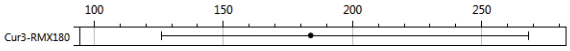

Confidence intervals estimated distance moved (H3 - Zone 1)

Genotype Zone 1	Genotype Zone 2	Mean	Lower 95% CL	Upper 95% CL	Group
Cur3	RMX180	183.8	125.9	268.2	a

Model summary

```
Linear mixed model fit by REML. t-tests use Satterthwaite's method ['lmerModLmerTest']
Formula: Estimated_distance_moved_H3_Zone_1 ~ 1 + (1 | Genotype_Zone_1:Plant_Zone_1) +      (1 |
Genotype_Zone_2:Plant_Zone_2)
   Data: data

REML criterion at convergence: 92.7

Scaled residuals:
    Min      1Q  Median      3Q     Max
-2.8705 -0.2986  0.2767  0.5079  1.6481

Random effects:
 Groups                       Name        Variance Std.Dev.
 Genotype_Zone_1:Plant_Zone_1 (Intercept) 0.002828 0.05318
 Genotype_Zone_2:Plant_Zone_2 (Intercept) 0.000000 0.00000
 Residual                                 0.948611 0.97397
Number of obs: 33, groups:  Genotype_Zone_1:Plant_Zone_1, 10; Genotype_Zone_2:Plant_Zone_2, 10

Fixed effects:
            Estimate Std. Error      df t value Pr(>|t|)
(Intercept)   5.2138     0.1704 10.3416   30.59 1.75e-11 ***
---
Signif. codes:  0 '***' 0.001 '**' 0.01 '*' 0.05 '.' 0.1 ' ' 1
```

Model residuals

Statistic	Value
Sample skewness	-1.26
Sample excess kurtosis	1.911
Passed Shapiro Wilk normality test	No (p-value = 0.005167 < 0.05)

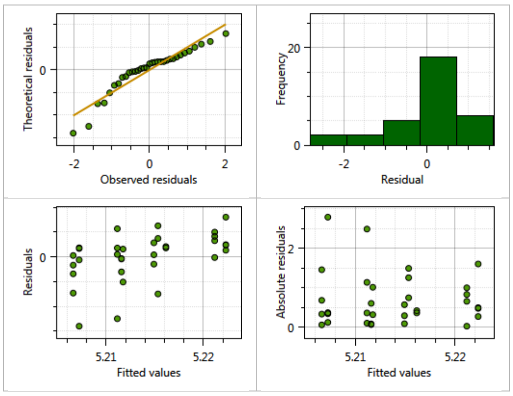

Analysis estimated distance moved (H3 - Zone 2)

**Analysis model**	Linear mixed model fit by REML: Estimated_distance_moved_H3_Zone_2 ~ 1 + (1|Genotype_Zone_1:Plant_Zone_1) + (1|Genotype_Zone_2:Plant_Zone_2)
**Transformation**	Natural logarithm

Confidence intervals estimated distance moved (H3 - Zone 2)

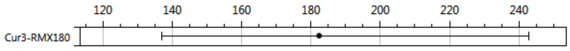

Confidence intervals estimated distance moved (H3 - Zone 2)

Genotype Zone 1	Genotype Zone 2	Mean	Lower 95% CL	Upper 95% CL	Group
Cur3	RMX180	182.4	136.9	242.9	a

Model summary

```
Linear mixed model fit by REML. t-tests use Satterthwaite's method ['lmerModLmerTest']
Formula: Estimated_distance_moved_H3_Zone_2 ~ 1 + (1 | Genotype_Zone_1:Plant_Zone_1) +      (1 |
Genotype_Zone_2:Plant_Zone_2)
   Data: data

REML criterion at convergence: 102.2

Scaled residuals:
    Min      1Q  Median      3Q     Max
-2.7743 -0.6314  0.2484  0.6434  1.3842

Random effects:
 Groups                       Name        Variance Std.Dev.
 Genotype_Zone_1:Plant_Zone_1 (Intercept) 0.0000   0.0000
 Genotype_Zone_2:Plant_Zone_2 (Intercept) 0.0000   0.0000
 Residual                                 0.7826   0.8846
Number of obs: 39, groups:  Genotype_Zone_1:Plant_Zone_1, 10; Genotype_Zone_2:Plant_Zone_2, 10

Fixed effects:
            Estimate Std. Error      df t value Pr(>|t|)
(Intercept)   5.2060     0.1417 38.0000   36.75   <2e-16 ***
---
Signif. codes:  0 '***' 0.001 '**' 0.01 '*' 0.05 '.' 0.1 ' ' 1
```

Model residuals

Statistic	Value
Sample skewness	-1.093
Sample excess kurtosis	1.141
Passed Shapiro Wilk normality test	No (p-value = 0.005416 < 0.05)

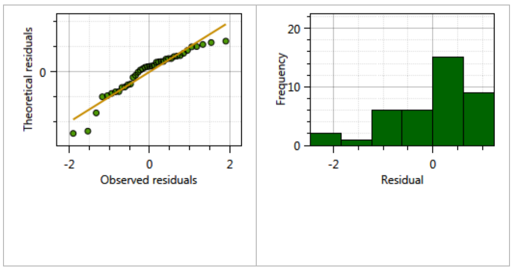

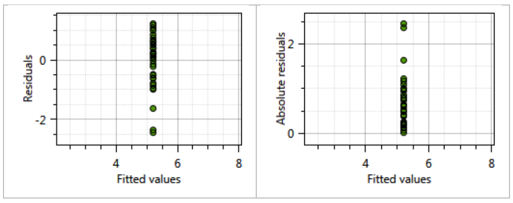

Analysis estimated distance moved (H4 - Zone 1)

**Analysis model**	Linear mixed model fit by REML: Estimated_distance_moved_H4_Zone_1 ~ 1 + (1|Genotype_Zone_1:Plant_Zone_1) + (1|Genotype_Zone_2:Plant_Zone_2)
**Transformation**	Natural logarithm

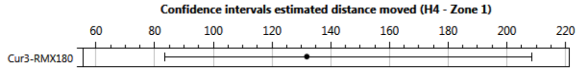

Confidence intervals estimated distance moved (H4 - Zone 1)

Genotype Zone 1	Genotype Zone 2	Mean	Lower 95% CL	Upper 95% CL	Group
Cur3	RMX180	131.8	83.31	208.6	a

Model summary

```
Linear mixed model fit by REML. t-tests use Satterthwaite's method ['lmerModLmerTest']
Formula: Estimated_distance_moved_H4_Zone_1 ~ 1 + (1 | Genotype_Zone_1:Plant_Zone_1) +      (1 |
Genotype_Zone_2:Plant_Zone_2)
   Data: data

REML criterion at convergence: 85.4

Scaled residuals:
    Min      1Q  Median      3Q     Max
-2.0853 -0.6331  0.3688  0.6644  1.3381

Random effects:
 Groups                       Name        Variance Std.Dev.
 Genotype_Zone_1:Plant_Zone_1 (Intercept) 0.00000  0.0000
 Genotype_Zone_2:Plant_Zone_2 (Intercept) 0.09509  0.3084
 Residual                                 0.90917  0.9535
Number of obs: 30, groups:  Genotype_Zone_1:Plant_Zone_1, 10; Genotype_Zone_2:Plant_Zone_2, 10

Fixed effects:
            Estimate Std. Error     df t value Pr(>|t|)
(Intercept)   4.8815     0.2008 8.4297   24.32 4.19e-09 ***
---
Signif. codes:  0 '***' 0.001 '**' 0.01 '*' 0.05 '.' 0.1 ' ' 1
```

Model residuals

Statistic	Value
Sample skewness	-0.8455
Sample excess kurtosis	-0.3268
Passed Shapiro Wilk normality test	No (p-value = 0.007712 < 0.05)

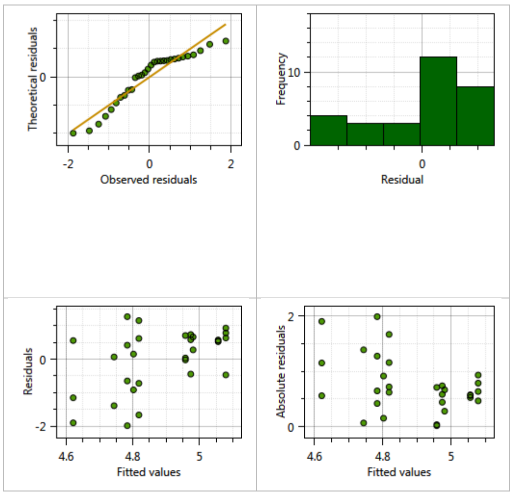

Analysis estimated distance moved (H4 - Zone 2)

**Analysis model**	Linear mixed model fit by REML: Estimated_distance_moved_H4_Zone_2 ~ 1 + (1|Genotype_Zone_1:Plant_Zone_1) + (1|Genotype_Zone_2:Plant_Zone_2)
**Transformation**	Natural logarithm

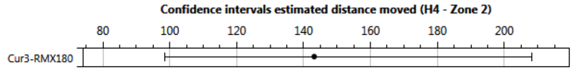

Confidence intervals estimated distance moved (H4 - Zone 2)

Genotype Zone 1	Genotype Zone 2	Mean	Lower 95% CL	Upper 95% CL	Group
Cur3	RMX180	143.2	98.4	208.3	a

Model summary

```
Linear mixed model fit by REML. t-tests use Satterthwaite's method ['lmerModLmerTest']
Formula: Estimated_distance_moved_H4_Zone_2 ~ 1 + (1 | Genotype_Zone_1:Plant_Zone_1) +      (1 |
Genotype_Zone_2:Plant_Zone_2)
   Data: data

REML criterion at convergence: 103.3

Scaled residuals:
    Min      1Q  Median      3Q     Max
-3.0942 -0.3470  0.1552  0.7085  1.3049

Random effects:
 Groups                       Name        Variance Std.Dev.
 Genotype_Zone_1:Plant_Zone_1 (Intercept) 0.00000  0.0000
 Genotype_Zone_2:Plant_Zone_2 (Intercept) 0.02137  0.1462
 Residual                                 0.91363  0.9558
Number of obs: 37, groups:  Genotype_Zone_1:Plant_Zone_1, 10; Genotype_Zone_2:Plant_Zone_2, 10

Fixed effects:
            Estimate Std. Error    df t value Pr(>|t|)
(Intercept)    4.964      0.164 8.409   30.27 7.01e-10 ***
```

```
---
Signif. codes:  0 '***' 0.001 '**' 0.01 '*' 0.05 '.' 0.1 ' ' 1
```

Model residuals

Statistic	Value
Sample skewness	-1.356
Sample excess kurtosis	2.108
Passed Shapiro Wilk normality test	No (p-value = 0.001666 < 0.05)

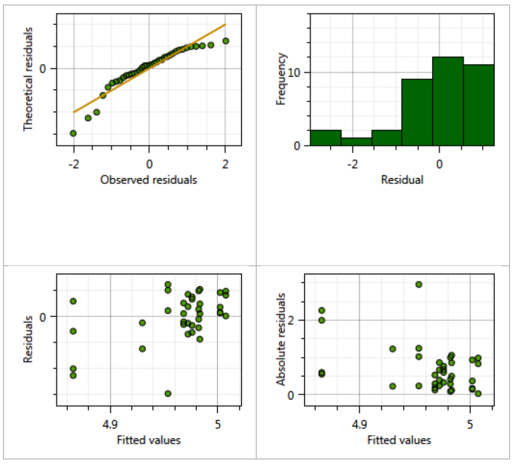

Analysis estimated distance moved (H5 - Zone 1)

**Analysis model**	Linear mixed model fit by REML: Estimated_distance_moved_H5_Zone_1 ~ 1 + (1|Genotype_Zone_1:Plant_Zone_1) + (1|Genotype_Zone_2:Plant_Zone_2)
**Transformation**	Natural logarithm

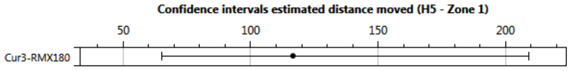

Confidence intervals estimated distance moved (H5 - Zone 1)

Genotype Zone 1	Genotype Zone 2	Mean	Lower 95% CL	Upper 95% CL	Group
Cur3	RMX180	116.6	64.97	209.3	a

Model summary

```
Linear mixed model fit by REML. t-tests use Satterthwaite's method ['lmerModLmerTest']
Formula: Estimated_distance_moved_H5_Zone_1 ~ 1 + (1 | Genotype_Zone_1:Plant_Zone_1) +      (1 |
Genotype_Zone_2:Plant_Zone_2)
   Data: data

REML criterion at convergence: 99

Scaled residuals: 
    Min      1Q  Median      3Q     Max 
-2.4575 -0.5480  0.3394  0.6662  1.3577 

Random effects:
 Groups                       Name        Variance Std.Dev.
 Genotype_Zone_1:Plant_Zone_1 (Intercept) 0.00819  0.0905  
 Genotype_Zone_2:Plant_Zone_2 (Intercept) 0.12572  0.3546  
 Residual                                 1.47026  1.2125  
Number of obs: 30, groups:  Genotype_Zone_1:Plant_Zone_1, 10; Genotype_Zone_2:Plant_Zone_2, 10

Fixed effects:
            Estimate Std. Error     df t value Pr(>|t|)    
(Intercept)   4.7588     0.2513 7.5969   18.94 1.14e-07 ***
---
Signif. codes:  0 '***' 0.001 '**' 0.01 '*' 0.05 '.' 0.1 ' ' 1
```

Model residuals

Statistic	Value
Sample skewness	-1.166
Sample excess kurtosis	0.8167
Passed Shapiro Wilk normality test	No (p-value = 0.002991 < 0.05)

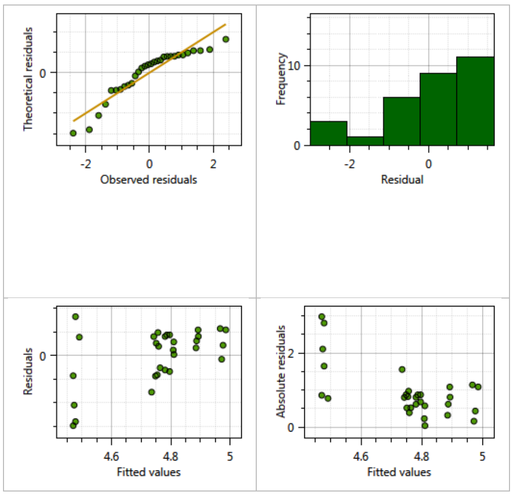

Analysis estimated distance moved (H5 - Zone 2)

**Analysis model**	Linear mixed model fit by REML: Estimated_distance_moved_H5_Zone_2 ~ 1 + (1|Genotype_Zone_1:Plant_Zone_1) + (1|Genotype_Zone_2:Plant_Zone_2)
**Transformation**	Natural logarithm

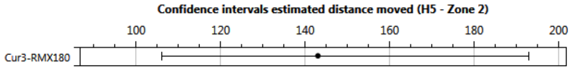

Confidence intervals estimated distance moved (H5 - Zone 2)

Genotype Zone 1	Genotype Zone 2	Mean	Lower 95% CL	Upper 95% CL	Group
Cur3	RMX180	143	106	193	a

Model summary

```
Linear mixed model fit by REML. t-tests use Satterthwaite's method ['lmerModLmerTest']
Formula: Estimated_distance_moved_H5_Zone_2 ~ 1 + (1 | Genotype_Zone_1:Plant_Zone_1) +      (1 |
Genotype_Zone_2:Plant_Zone_2)
   Data: data

REML criterion at convergence: 105.6

Scaled residuals:
    Min      1Q  Median      3Q     Max
-3.0017 -0.3730  0.1801  0.6830  1.4729

Random effects:
 Groups                       Name        Variance  Std.Dev.
 Genotype_Zone_1:Plant_Zone_1 (Intercept) 2.114e-16 1.454e-08
 Genotype_Zone_2:Plant_Zone_2 (Intercept) 0.000e+00 0.000e+00
 Residual                                 8.552e-01 9.247e-01
Number of obs: 39, groups:  Genotype_Zone_1:Plant_Zone_1, 10; Genotype_Zone_2:Plant_Zone_2, 10

Fixed effects:
            Estimate Std. Error      df t value Pr(>|t|)
(Intercept)   4.9630     0.1481 38.0000   33.52   <2e-16 ***
---
Signif. codes:  0 '***' 0.001 '**' 0.01 '*' 0.05 '.' 0.1 ' ' 1
```

Model residuals

Statistic	Value
Sample skewness	-1.264
Sample excess kurtosis	1.926
Passed Shapiro Wilk normality test	No (p-value = 0.002997 < 0.05)

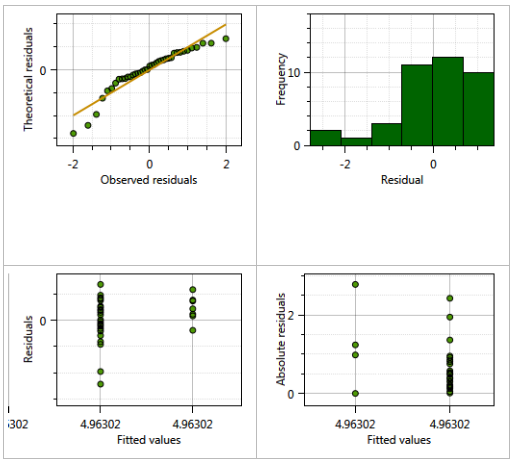

Analysis estimated distance moved (H6 - Zone 1)

**Analysis model**	Linear mixed model fit by REML: Estimated_distance_moved_H6_Zone_1 ~ 1 + (1|Genotype_Zone_1:Plant_Zone_1) + (1|Genotype_Zone_2:Plant_Zone_2)
**Transformation**	Natural logarithm

Confidence intervals estimated distance moved (H6 - Zone 1)

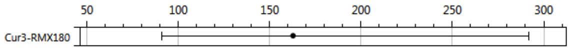

Confidence intervals estimated distance moved (H6 - Zone 1)

Genotype Zone 1	Genotype Zone 2	Mean	Lower 95% CL	Upper 95% CL	Group
Cur3	RMX180	162.8	90.76	292	a

Model summary

```
Linear mixed model fit by REML. t-tests use Satterthwaite's method ['lmerModLmerTest']
Formula: Estimated_distance_moved_H6_Zone_1 ~ 1 + (1 | Genotype_Zone_1:Plant_Zone_1) +      (1 |
Genotype_Zone_2:Plant_Zone_2)
   Data: data

REML criterion at convergence: 79.1

Scaled residuals:
    Min      1Q  Median      3Q     Max
-1.7607 -0.7254  0.1658  0.7891  1.3278

Random effects:
 Groups                       Name        Variance Std.Dev.
 Genotype_Zone_1:Plant_Zone_1 (Intercept) 0.0000   0.0000
 Genotype_Zone_2:Plant_Zone_2 (Intercept) 0.2419   0.4918
 Residual                                 0.9006   0.9490
Number of obs: 27, groups:  Genotype_Zone_1:Plant_Zone_1, 10; Genotype_Zone_2:Plant_Zone_2, 10

Fixed effects:
            Estimate Std. Error     df t value Pr(>|t|)
(Intercept)   5.0924     0.2455 6.7810   20.75 2.18e-07 ***
---
Signif. codes:  0 '***' 0.001 '**' 0.01 '*' 0.05 '.' 0.1 ' ' 1
```

Model residuals

Statistic	Value
Sample skewness	-0.5699
Sample excess kurtosis	-0.9727
Passed Shapiro Wilk normality test	No (p-value = 0.02698 < 0.05)

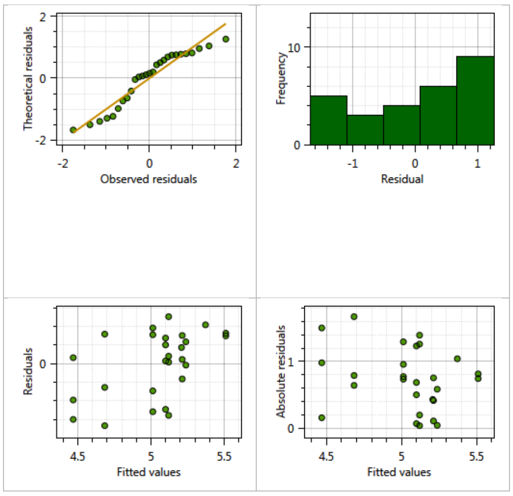

Analysis estimated distance moved (H6 - Zone 2)

**Analysis model**	Linear mixed model fit by REML: Estimated_distance_moved_H6_Zone_2 ~ 1 + (1|Genotype_Zone_1:Plant_Zone_1) + (1|Genotype_Zone_2:Plant_Zone_2)
**Transformation**	Natural logarithm

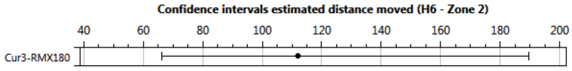

Confidence intervals estimated distance moved (H6 - Zone 2)

Genotype Zone 1	Genotype Zone 2	Mean	Lower 95% CL	Upper 95% CL	Group
Cur3	RMX180	112	66.07	189.7	a

Model summary

```
Linear mixed model fit by REML. t-tests use Satterthwaite's method ['lmerModLmerTest']
Formula: Estimated_distance_moved_H6_Zone_2 ~ 1 + (1 | Genotype_Zone_1:Plant_Zone_1) +      (1 |
Genotype_Zone_2:Plant_Zone_2)
   Data: data

REML criterion at convergence: 118.2

Scaled residuals:
     Min       1Q   Median       3Q      Max
-2.97685 -0.30123  0.07863  0.69421  1.34660

Random effects:
 Groups                       Name        Variance Std.Dev.
 Genotype_Zone_1:Plant_Zone_1 (Intercept) 0.04894  0.2212
 Genotype_Zone_2:Plant_Zone_2 (Intercept) 0.12303  0.3508
 Residual                                 1.15242  1.0735
Number of obs: 38, groups:  Genotype_Zone_1:Plant_Zone_1, 10; Genotype_Zone_2:Plant_Zone_2, 10

Fixed effects:
            Estimate Std. Error     df t value Pr(>|t|)
(Intercept)   4.7181     0.2183 6.3386   21.61 3.55e-07 ***
---
Signif. codes:  0 '***' 0.001 '**' 0.01 '*' 0.05 '.' 0.1 ' ' 1
```

Model residuals

Statistic	Value
Sample skewness	-1.086
Sample excess kurtosis	1.45
Passed Shapiro Wilk normality test	No (p-value = 0.009372 < 0.05)

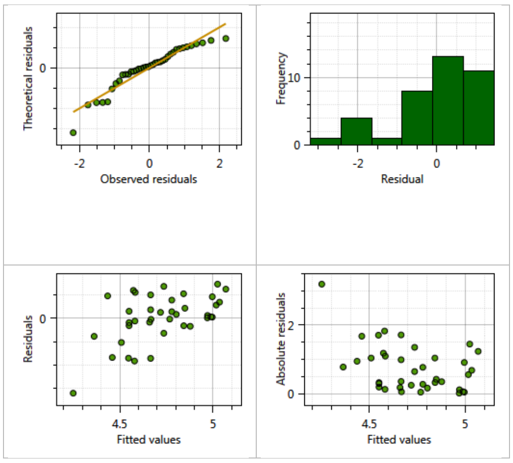

Analysis estimated distance moved (H7 - Zone 1)

**Analysis model**	Linear mixed model fit by REML: Estimated_distance_moved_H7_Zone_1 ~ 1 + (1|Genotype_Zone_1:Plant_Zone_1) + (1|Genotype_Zone_2:Plant_Zone_2)
**Transformation**	Natural logarithm

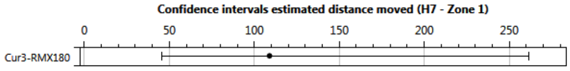

Confidence intervals estimated distance moved (H7 - Zone 1)

Genotype Zone 1	Genotype Zone 2	Mean	Lower 95% CL	Upper 95% CL	Group
Cur3	RMX180	108.9	45.33	261.7	a

Model summary

```
Linear mixed model fit by REML. t-tests use Satterthwaite's method ['lmerModLmerTest']
Formula: Estimated_distance_moved_H7_Zone_1 ~ 1 + (1 | Genotype_Zone_1:Plant_Zone_1) +      (1 |
Genotype_Zone_2:Plant_Zone_2)
   Data: data
```

```
REML criterion at convergence: 115.6

Scaled residuals:
    Min      1Q  Median      3Q     Max
-4.1964 -0.1653  0.2796  0.4892  0.7896

Random effects:
 Groups                      Name        Variance Std.Dev.
 Genotype_Zone_2:Plant_Zone_2 (Intercept) 0.1044   0.3231
 Genotype_Zone_1:Plant_Zone_1 (Intercept) 0.0000   0.0000
 Residual                                3.6478   1.9099
Number of obs: 28, groups:  Genotype_Zone_2:Plant_Zone_2, 10; Genotype_Zone_1:Plant_Zone_1, 9

Fixed effects:
            Estimate Std. Error     df t value Pr(>|t|)
(Intercept)   4.6905     0.3771 7.6467   12.44  2.4e-06 ***
---
Signif. codes:  0 '***' 0.001 '**' 0.01 '*' 0.05 '.' 0.1 ' ' 1
```

Model residuals

Statistic	Value
Sample skewness	-3.192
Sample excess kurtosis	12.25
Passed Shapiro Wilk normality test	No (p-value = 5.735E-07 < 0.05)

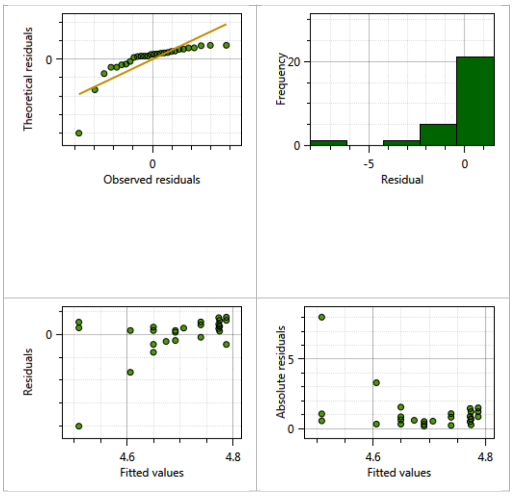

Analysis estimated distance moved (H7 - Zone 2)

**Analysis model**	Linear mixed model fit by REML: Estimated_distance_moved_H7_Zone_2 ~ 1 + (1|Genotype_Zone_1:Plant_Zone_1) + (1|Genotype_Zone_2:Plant_Zone_2)
**Transformation**	Natural logarithm

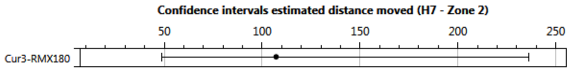

Confidence intervals estimated distance moved (H7 - Zone 2)

Genotype Zone 1	Genotype Zone 2	Mean	Lower 95% CL	Upper 95% CL	Group
Cur3	RMX180	107.1	48.53	236.3	a

Model summary

```
Linear mixed model fit by REML. t-tests use Satterthwaite's method ['lmerModLmerTest']
Formula: Estimated_distance_moved_H7_Zone_2 ~ 1 + (1 | Genotype_Zone_1:Plant_Zone_1) +      (1 |
Genotype_Zone_2:Plant_Zone_2)
   Data: data

REML criterion at convergence: 115.5

Scaled residuals:
     Min       1Q   Median       3Q      Max
-2.20722 -0.38150 -0.05443  0.49600  1.42373

Random effects:
 Groups                      Name        Variance Std.Dev.
 Genotype_Zone_1:Plant_Zone_1 (Intercept) 0.7638   0.8740
 Genotype_Zone_2:Plant_Zone_2 (Intercept) 0.4044   0.6359
 Residual                                0.5486   0.7406
Number of obs: 39, groups:  Genotype_Zone_1:Plant_Zone_1, 10; Genotype_Zone_2:Plant_Zone_2, 10

Fixed effects:
            Estimate Std. Error      df t value Pr(>|t|)
(Intercept)   4.6737     0.3621 11.6400   12.91 2.98e-08 ***
---
Signif. codes:  0 '***' 0.001 '**' 0.01 '*' 0.05 '.' 0.1 ' ' 1
```

Model residuals

Statistic	Value
Sample skewness	-0.3694
Sample excess kurtosis	0.2502
Passed Shapiro Wilk normality test	Yes (p-value = 0.6084 > 0.05)

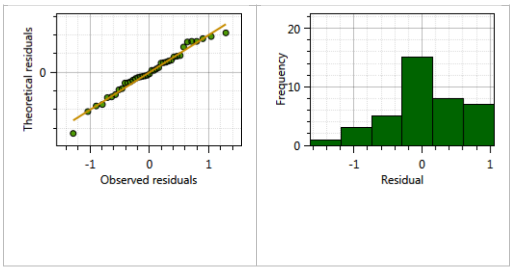

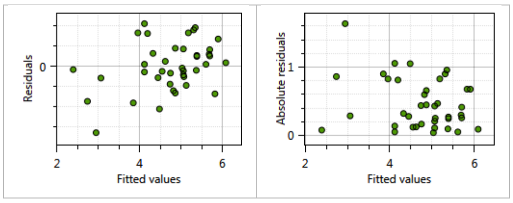

Analysis estimated distance moved H0 (diff. Zone 1 - Zone 2)

**Analysis model**	Generalized linear mixed model with dispersion factor, formula=cbind(Estimated_distance_moved_H0_Zone_1,Estimated_distance_moved_H0_Zone_2) ~ 1 + (1|Genotype_Zone_1:Plant_Zone_1) + (1|Genotype_Zone_2:Plant_Zone_2)
**Transformation**	Logit

Confidence intervals estimated distance moved H0 (diff. Zone 1 - Zone 2)

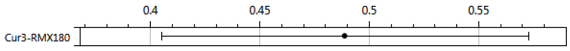

Confidence intervals estimated distance moved H0 (diff. Zone 1 - Zone 2)

Genotype Zone 1	Genotype Zone 2	Mean	Lower 95% CL	Upper 95% CL	Group
Cur3	RMX180	0.4887	0.405	0.573	a

Model summary

```
Linear mixed model fit by REML. t-tests use Satterthwaite's method ['lmerModLmerTest']
Formula: ziFormula
   Data: data
Weights: wi

REML criterion at convergence: 103.3

Scaled residuals:
     Min       1Q   Median       3Q      Max
-1.95027 -0.44139 -0.02179  0.50860  1.92384

Random effects:
 Groups                      Name        Variance  Std.Dev.
 Genotype_Zone_1:Plant_Zone_1 (Intercept)   0.00000  0.0000
 Genotype_Zone_2:Plant_Zone_2 (Intercept)   0.02931  0.1712
 Residual                               100.42120 10.0210
Number of obs: 37, groups:  Genotype_Zone_1:Plant_Zone_1, 10; Genotype_Zone_2:Plant_Zone_2, 10

Fixed effects:
            Estimate Std. Error      df t value Pr(>|t|)
(Intercept)  -0.0453     0.1511  9.4454    -0.3    0.771

Dispersion: 10.02
```

Model residuals

Statistic	Value
Sample skewness	-0.1064
Sample excess kurtosis	-0.2578
Passed Shapiro Wilk normality test	Yes (p-value = 0.4582 > 0.05)

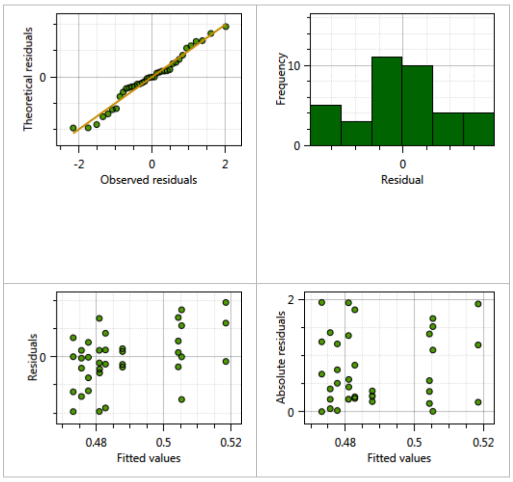

Analysis estimated distance moved H1 (diff. Zone 1 - Zone 2)

**Analysis model**	Generalized linear mixed model with dispersion factor, formula=cbind(Estimated_distance_moved_H1_Zone_1,Estimated_distance_moved_H1_Zone_2) ~ 1 + (1|Genotype_Zone_1:Plant_Zone_1) + (1|Genotype_Zone_2:Plant_Zone_2)
**Transformation**	Logit

Confidence intervals estimated distance moved H1 (diff. Zone 1 - Zone 2)

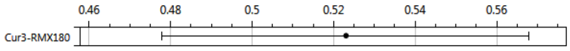

Confidence intervals estimated distance moved H1 (diff. Zone 1 - Zone 2)

Genotype Zone 1	Genotype Zone 2	Mean	Lower 95% CL	Upper 95% CL	Group
Cur3	RMX180	0.523	0.4777	0.5679	a

Model summary

```
Linear mixed model fit by REML. t-tests use Satterthwaite's method ['lmerModLmerTest']
Formula: ziFormula
   Data: data
Weights: wi

REML criterion at convergence: 66.5

Scaled residuals:
    Min      1Q  Median      3Q     Max
-2.2024 -0.6252  0.1305  0.6001  1.7763

Random effects:
 Groups                      Name        Variance Std.Dev.
 Genotype_Zone_1:Plant_Zone_1 (Intercept)  0.00    0.000
 Genotype_Zone_2:Plant_Zone_2 (Intercept)  0.00    0.000
 Residual                               42.92    6.551
Number of obs: 37, groups:  Genotype_Zone_1:Plant_Zone_1, 10; Genotype_Zone_2:Plant_Zone_2, 10

Fixed effects:
            Estimate Std. Error       df t value Pr(>|t|)
```

```
(Intercept)  0.09219    0.08939 36.00000   1.031    0.309

Dispersion: 6.551
```

Model residuals

Statistic	Value
Sample skewness	-0.3119
Sample excess kurtosis	-0.4079
Passed Shapiro Wilk normality test	Yes (p-value = 0.6387 > 0.05)

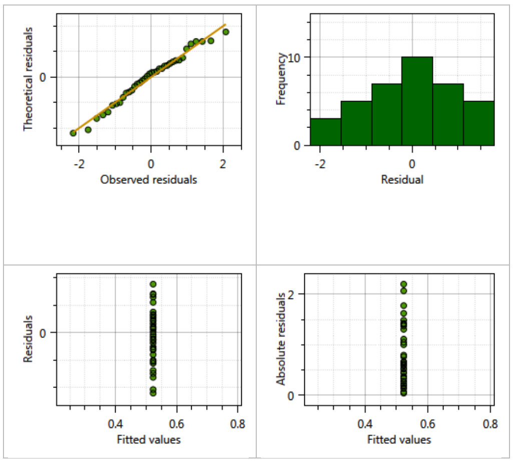

Analysis estimated distance moved H2 (diff. Zone 1 - Zone 2)

**Analysis model**	Generalized linear mixed model with dispersion factor, formula=cbind(Estimated_distance_moved_H2_Zone_1,Estimated_distance_moved_H2_Zone_2) ~ 1 + (1|Genotype_Zone_1:Plant_Zone_1) + (1|Genotype_Zone_2:Plant_Zone_2)
**Transformation**	Logit

Confidence intervals estimated distance moved H2 (diff. Zone 1 - Zone 2)

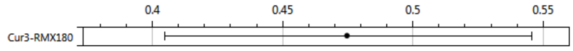

Confidence intervals estimated distance moved H2 (diff. Zone 1 - Zone 2)

Genotype Zone 1	Genotype Zone 2	Mean	Lower 95% CL	Upper 95% CL	Group
Cur3	RMX180	0.4747	0.4046	0.5458	a

Model summary

```
Linear mixed model fit by REML. t-tests use Satterthwaite's method ['lmerModLmerTest']
Formula: ziFormula
   Data: data
Weights: wi

REML criterion at convergence: 85.8

Scaled residuals:
    Min      1Q  Median      3Q     Max
-1.7178 -0.6478 -0.2831  0.4326  2.7297

Random effects:
 Groups                      Name        Variance Std.Dev.
 Genotype_Zone_1:Plant_Zone_1 (Intercept)  0.06237 0.24974
 Genotype_Zone_2:Plant_Zone_2 (Intercept)  0.00321 0.05665
 Residual                                 31.90458 5.64841
Number of obs: 39, groups:  Genotype_Zone_1:Plant_Zone_1, 10; Genotype_Zone_2:Plant_Zone_2, 10

Fixed effects:
            Estimate Std. Error      df t value Pr(>|t|)
(Intercept)  -0.1014     0.1182  6.4026  -0.858    0.422

Dispersion: 5.648
```

Model residuals

Statistic	Value
Sample skewness	0.8541
Sample excess kurtosis	0.831
Passed Shapiro Wilk normality test	Yes (p-value = 0.1091 > 0.05)

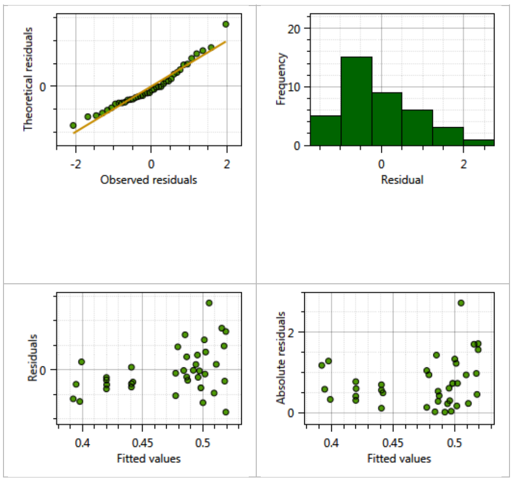

Analysis estimated distance moved H3 (diff. Zone 1 - Zone 2)

**Analysis model**	Generalized linear mixed model with dispersion factor, formula=cbind(Estimated_distance_moved_H3_Zone_1,Estimated_distance_moved_H3_Zone_2) ~ 1 + (1|Genotype_Zone_1:Plant_Zone_1) + (1|Genotype_Zone_2:Plant_Zone_2)
**Transformation**	Logit

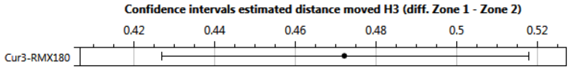

Confidence intervals estimated distance moved H3 (diff. Zone 1 - Zone 2)

Genotype Zone 1	Genotype Zone 2	Mean	Lower 95% CL	Upper 95% CL	Group
Cur3	RMX180	0.4721	0.4268	0.518	a

Model summary

```
Linear mixed model fit by REML. t-tests use Satterthwaite's method ['lmerModLmerTest']
Formula: ziFormula
   Data: data
Weights: wi

REML criterion at convergence: 83.6

Scaled residuals:
    Min      1Q  Median      3Q     Max
-1.6511 -0.9781 -0.3743  0.4679  1.9907

Random effects:
 Groups                         Name        Variance Std.Dev.
 Genotype_Zone_1:Plant_Zone_1 (Intercept)  0.00    0.000
 Genotype_Zone_2:Plant_Zone_2 (Intercept)  0.00    0.000
 Residual                                 36.77    6.064
Number of obs: 39, groups:  Genotype_Zone_1:Plant_Zone_1, 10; Genotype_Zone_2:Plant_Zone_2, 10

Fixed effects:
            Estimate Std. Error       df t value Pr(>|t|)
(Intercept) -0.11160    0.09062 38.00000  -1.232    0.226

Dispersion: 6.064
```

Model residuals

Statistic	Value
Sample skewness	0.5034
Sample excess kurtosis	-0.7466
Passed Shapiro Wilk normality test	Yes (p-value = 0.0503 > 0.05)

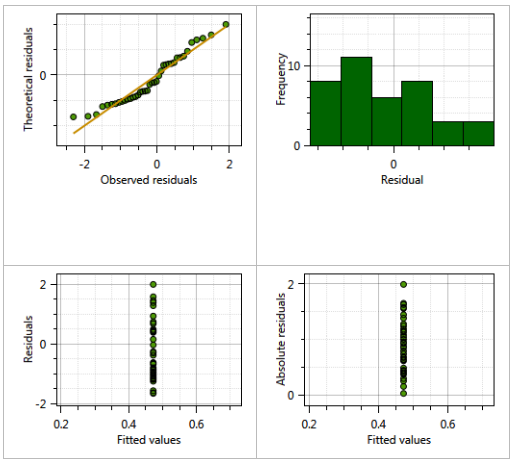

Analysis estimated distance moved H4 (diff. Zone 1 - Zone 2)

**Analysis model**	Generalized linear mixed model with dispersion factor, formula=cbind(Estimated_distance_moved_H4_Zone_1,Estimated_distance_moved_H4_Zone_2) ~ 1 + (1|Genotype_Zone_1:Plant_Zone_1) + (1|Genotype_Zone_2:Plant_Zone_2)
**Transformation**	Logit

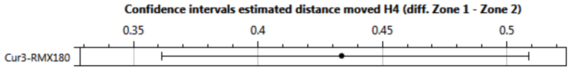

Confidence intervals estimated distance moved H4 (diff. Zone 1 - Zone 2)

Genotype Zone 1	Genotype Zone 2	Mean	Lower 95% CL	Upper 95% CL	Group
Cur3	RMX180	0.4336	0.3612	0.5089	a

Model summary

```
Linear mixed model fit by REML. t-tests use Satterthwaite's method ['lmerModLmerTest']
Formula: ziFormula
   Data: data
Weights: wi

REML criterion at convergence: 105.5

Scaled residuals:
    Min      1Q  Median      3Q     Max
-1.7055 -0.9585 -0.3428  0.7561  1.7761

Random effects:
 Groups                         Name        Variance Std.Dev.
 Genotype_Zone_1:Plant_Zone_1 (Intercept)  0.03848 0.1962
 Genotype_Zone_2:Plant_Zone_2 (Intercept)  0.00000 0.0000
 Residual                                 40.65623 6.3762
Number of obs: 39, groups:  Genotype_Zone_1:Plant_Zone_1, 10; Genotype_Zone_2:Plant_Zone_2, 10

Fixed effects:
            Estimate Std. Error      df t value Pr(>|t|)
(Intercept)  -0.2672     0.1302  7.6128  -2.053    0.076 .
---
Signif. codes:  0 '***' 0.001 '**' 0.01 '*' 0.05 '.' 0.1 ' ' 1

Dispersion: 6.376
```

Model residuals

Statistic	Value
Sample skewness	0.2008
Sample excess kurtosis	-1.187
Passed Shapiro Wilk normality test	Yes (p-value = 0.07986 > 0.05)

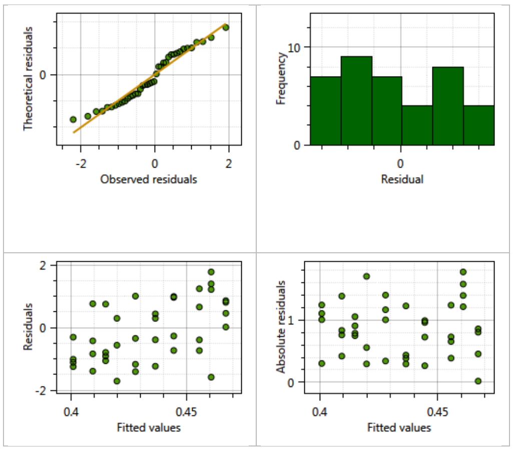

Analysis estimated distance moved H5 (diff. Zone 1 - Zone 2)

**Analysis model**	Generalized linear mixed model with dispersion factor, formula=cbind(Estimated_distance_moved_H5_Zone_1,Estimated_distance_moved_H5_Zone_2) ~ 1 + (1|Genotype_Zone_1:Plant_Zone_1) + (1|Genotype_Zone_2:Plant_Zone_2)
**Transformation**	Logit

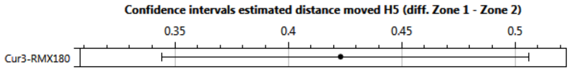

Confidence intervals estimated distance moved H5 (diff. Zone 1 - Zone 2)

Genotype Zone 1	Genotype Zone 2	Mean	Lower 95% CL	Upper 95% CL	Group
Cur3	RMX180	0.423	0.344	0.5062	a

Model summary

```
Linear mixed model fit by REML. t-tests use Satterthwaite's method ['lmerModLmerTest']
Formula: ziFormula
   Data: data
Weights: wi

REML criterion at convergence: 106.3

Scaled residuals: 
    Min      1Q  Median      3Q     Max 
-1.9066 -0.8878 -0.2225  0.5118  1.9226 

Random effects:
 Groups                      Name        Variance Std.Dev.
 Genotype_Zone_1:Plant_Zone_1 (Intercept)  0.04214 0.2053  
 Genotype_Zone_2:Plant_Zone_2 (Intercept)  0.00000 0.0000  
 Residual                                 44.34602 6.6593  
Number of obs: 39, groups:  Genotype_Zone_1:Plant_Zone_1, 10; Genotype_Zone_2:Plant_Zone_2, 10

Fixed effects:
            Estimate Std. Error      df t value Pr(>|t|)  
(Intercept)  -0.3104     0.1355  5.7516  -2.291   0.0637 .
---
Signif. codes:  0 '***' 0.001 '**' 0.01 '*' 0.05 '.' 0.1 ' ' 1

Dispersion: 6.659
```

Model residuals

Statistic	Value
Sample skewness	0.1314
Sample excess kurtosis	-0.7983
Passed Shapiro Wilk normality test	Yes (p-value = 0.62 > 0.05)

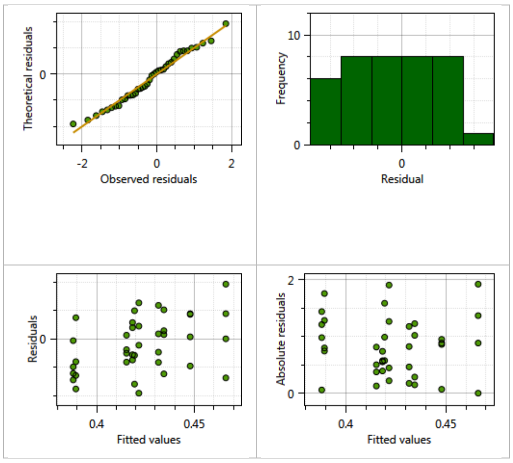

Analysis estimated distance moved H6 (diff. Zone 1 - Zone 2)

**Analysis model**	Generalized linear mixed model with dispersion factor, formula=cbind(Estimated_distance_moved_H6_Zone_1,Estimated_distance_moved_H6_Zone_2) ~ 1 + (1|Genotype_Zone_1:Plant_Zone_1) + (1|Genotype_Zone_2:Plant_Zone_2)
**Transformation**	Logit

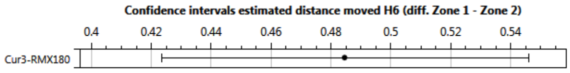

Confidence intervals estimated distance moved H6 (diff. Zone 1 - Zone 2)

Genotype Zone 1	Genotype Zone 2	Mean	Lower 95% CL	Upper 95% CL	Group
Cur3	RMX180	0.4845	0.4234	0.5461	a

Model summary

```
Linear mixed model fit by REML. t-tests use Satterthwaite's method ['lmerModLmerTest']
Formula: ziFormula
   Data: data
```

```
Weights: wi

REML criterion at convergence: 115.9

Scaled residuals:
    Min      1Q  Median      3Q     Max
-2.3674 -0.7873 -0.3879  0.4950  1.7341

Random effects:
 Groups                      Name        Variance Std.Dev.
 Genotype_Zone_1:Plant_Zone_1 (Intercept)  0.0      0.000
 Genotype_Zone_2:Plant_Zone_2 (Intercept)  0.0      0.000
 Residual                                 48.6      6.971
Number of obs: 39, groups:  Genotype_Zone_1:Plant_Zone_1, 10; Genotype_Zone_2:Plant_Zone_2, 10

Fixed effects:
            Estimate Std. Error       df t value Pr(>|t|)
(Intercept) -0.06196    0.12202 38.00000  -0.508    0.615

Dispersion: 6.971
```

Model residuals

Statistic	Value
Sample skewness	0.1487
Sample excess kurtosis	-0.3696
Passed Shapiro Wilk normality test	Yes (p-value = 0.8069 > 0.05)

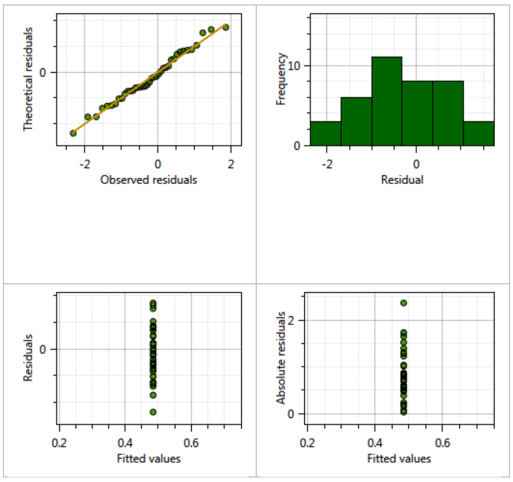

Analysis estimated distance moved H7 (diff. Zone 1 - Zone 2)

**Analysis model**	Generalized linear mixed model with dispersion factor, formula=cbind(Estimated_distance_moved_H7_Zone_1,Estimated_distance_moved_H7_Zone_2) ~ 1 + (1|Genotype_Zone_1:Plant_Zone_1) + (1|Genotype_Zone_2:Plant_Zone_2)
**Transformation**	Logit

Confidence intervals estimated distance moved H7 (diff. Zone 1 - Zone 2)

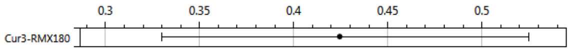

Confidence intervals estimated distance moved H7 (diff. Zone 1 - Zone 2)

Genotype Zone 1	Genotype Zone 2	Mean	Lower 95% CL	Upper 95% CL	Group
Cur3	RMX180	0.4244	0.3297	0.525	a

Model summary

```
Linear mixed model fit by REML. t-tests use Satterthwaite's method ['lmerModLmerTest']
Formula: ziFormula
   Data: data
Weights: wi

REML criterion at convergence: 104.9

Scaled residuals:
    Min      1Q  Median      3Q     Max
-1.6179 -0.8983 -0.2520  0.5270  1.5122

Random effects:
 Groups                      Name        Variance Std.Dev.
 Genotype_Zone_1:Plant_Zone_1 (Intercept)  0.05874 0.2424
 Genotype_Zone_2:Plant_Zone_2 (Intercept)  0.13353 0.3654
 Residual                                 26.11069 5.1099
Number of obs: 39, groups:  Genotype_Zone_1:Plant_Zone_1, 10; Genotype_Zone_2:Plant_Zone_2, 10

Fixed effects:
            Estimate Std. Error      df t value Pr(>|t|)
(Intercept)  -0.3046     0.1761  8.1459   -1.73    0.121

Dispersion: 5.11
```

Model residuals

Statistic	Value
Sample skewness	0.2569
Sample excess kurtosis	-0.8603
Passed Shapiro Wilk normality test	Yes (p-value = 0.2355 > 0.05)

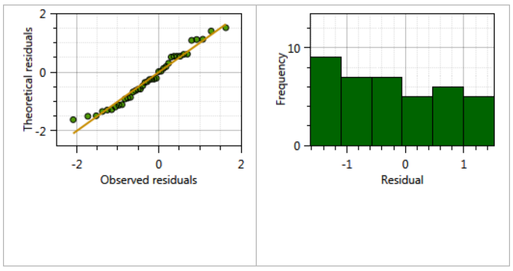

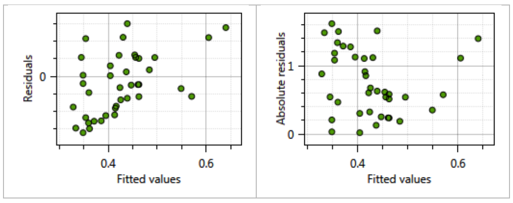

Estimated duration halting per zone

Selected zones	Zone 1, Zone 2
Data transformation	Natural logarithm
Analysis	Zone difference analysis

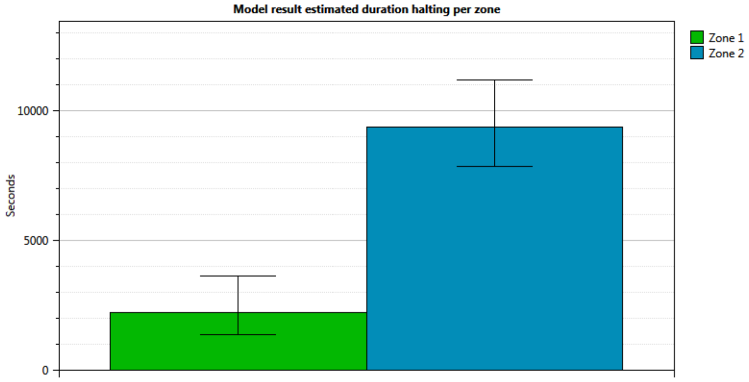

Results difference tests Zone 1 - Zone 2: p values and 95% confidence intervals of the difference on the transformed scale for each statistic.

Behaviour statistic	Cur3-RMX180
Estimated duration halting (diff. Zone 1 - Zone 2)	p=0.00476**
	[-1.73, -0.572]

The model predictions and 95% confidence intervals for each statistic.

Statistic	Cur3-RMX180	Remark
Estimated duration halting (Zone 1)	2.21E+03	CR
	[1.34E+03, 3.63E+03]	
Estimated duration halting (Zone 2)	9.35E+03	CR
	[7.83E+03, 1.12E+04]	

CR = Check residuals

Data summary

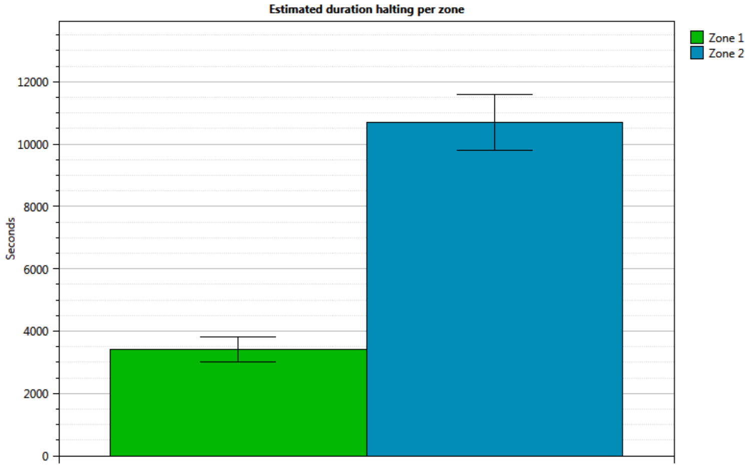

Genotype Zone 1	Genotype Zone 2	Genotype Zone 3	Mean Zone 1	StdErr Zone 1	Mean Zone 2	StdErr Zone 2
Cur3	RMX180	Neutral	3406.74	411.96	10696.09	905.43

Analysis estimated duration halting (Zone 1)

**Analysis model**	Linear mixed model fit by REML: Estimated_duration_halting_Zone_1 ~ 1 + (1|Genotype_Zone_1:Plant_Zone_1) + (1|Genotype_Zone_2:Plant_Zone_2)
**Transformation**	Natural logarithm

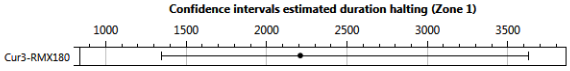

Confidence intervals estimated duration halting (Zone 1)

Genotype Zone 1	Genotype Zone 2	Mean	Lower 95% CL	Upper 95% CL	Group
Cur3	RMX180	2209	1344	3630	a

Model summary

```
Linear mixed model fit by REML. t-tests use Satterthwaite's method ['lmerModLmerTest']
Formula: Estimated_duration_halting_Zone_1 ~ 1 + (1 | Genotype_Zone_1:Plant_Zone_1) +      (1 |
Genotype_Zone_2:Plant_Zone_2)
   Data: data

REML criterion at convergence: 124.6

Scaled residuals:
    Min      1Q  Median      3Q     Max
-3.5135 -0.3221  0.1740  0.5518  1.2597

Random effects:
 Groups                       Name        Variance Std.Dev.
 Genotype_Zone_1:Plant_Zone_1 (Intercept) 0.0000   0.0000
```

```
 Genotype_Zone_2:Plant_Zone_2 (Intercept) 0.1541   0.3925
 Residual                                 1.2899   1.1357
Number of obs: 39, groups:  Genotype_Zone_1:Plant_Zone_1, 10; Genotype_Zone_2:Plant_Zone_2, 10

Fixed effects:
            Estimate Std. Error     df t value Pr(>|t|)
(Intercept)   7.7004     0.2203 9.2142   34.95 4.13e-11 ***
---
Signif. codes:  0 '***' 0.001 '**' 0.01 '*' 0.05 '.' 0.1 ' ' 1
```

Model residuals

Statistic	Value
Sample skewness	-1.775
Sample excess kurtosis	4.04
Passed Shapiro Wilk normality test	No (p-value = 9.771E-05 < 0.05)

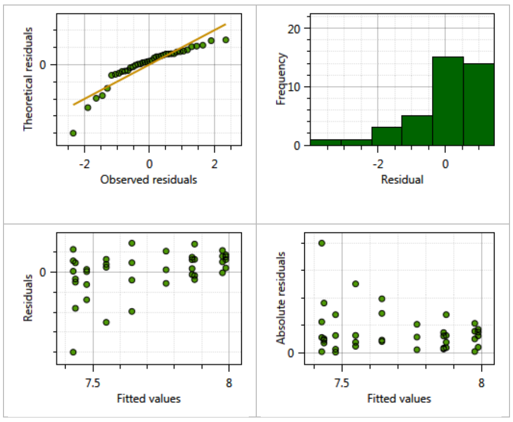

Analysis estimated duration halting (Zone 2)

**Analysis model**	Linear mixed model fit by REML: Estimated_duration_halting_Zone_2 ~ 1 + (1|Genotype_Zone_1:Plant_Zone_1) + (1|Genotype_Zone_2:Plant_Zone_2)
**Transformation**	Natural logarithm

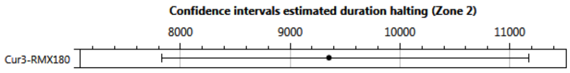

Confidence intervals estimated duration halting (Zone 2)

Genotype Zone 1	Genotype Zone 2	Mean	Lower 95% CL	Upper 95% CL	Group
Cur3	RMX180	9353	7827	1.118E+04	a

Model summary

```
Linear mixed model fit by REML. t-tests use Satterthwaite's method ['lmerModLmerTest']
Formula: Estimated_duration_halting_Zone_2 ~ 1 + (1 | Genotype_Zone_1:Plant_Zone_1) +      (1 |
Genotype_Zone_2:Plant_Zone_2)
   Data: data

REML criterion at convergence: 66

Scaled residuals:
    Min      1Q  Median      3Q     Max
-3.2870 -0.5380  0.1692  0.4418  1.8595

Random effects:
 Groups                       Name        Variance  Std.Dev.
 Genotype_Zone_1:Plant_Zone_1 (Intercept) 1.577e-20 1.256e-10
 Genotype_Zone_2:Plant_Zone_2 (Intercept) 0.000e+00 0.000e+00
 Residual                                 3.019e-01 5.494e-01
Number of obs: 39, groups:  Genotype_Zone_1:Plant_Zone_1, 10; Genotype_Zone_2:Plant_Zone_2, 10

Fixed effects:
            Estimate Std. Error       df t value Pr(>|t|)
(Intercept)  9.14342    0.08798 38.00000   103.9   <2e-16 ***
---
Signif. codes:  0 '***' 0.001 '**' 0.01 '*' 0.05 '.' 0.1 ' ' 1
```

Model residuals

Statistic	Value
Sample skewness	-0.6986
Sample excess kurtosis	2.084
Passed Shapiro Wilk normality test	No (p-value = 0.04147 < 0.05)

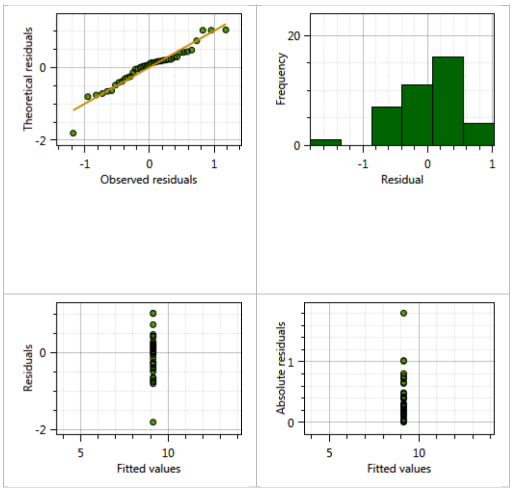

Analysis estimated duration halting (diff. Zone 1 - Zone 2)

**Analysis model**	Generalized linear mixed model with dispersion factor, formula=cbind(Estimated_duration_halting_Zone_1,Estimated_duration_halting_Zone_2) ~ 1 + (1|Genotype_Zone_1:Plant_Zone_1) + (1|Genotype_Zone_2:Plant_Zone_2)
**Transformation**	Logit

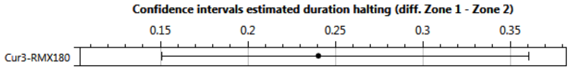

Confidence intervals estimated duration halting (diff. Zone 1 - Zone 2)

Genotype Zone 1	Genotype Zone 2	Mean	Lower 95% CL	Upper 95% CL	Group
Cur3	RMX180	0.2403	0.1506	0.3608	a

Model summary

```
Linear mixed model fit by REML. t-tests use Satterthwaite's method ['lmerModLmerTest']
Formula: ziFormula
   Data: data
Weights: wi

REML criterion at convergence: 120.1

Scaled residuals: 
     Min       1Q   Median       3Q      Max 
-1.52872 -0.56741 -0.02098  0.52707  2.58959 

Random effects:
 Groups                            Name        Variance  Std.Dev.
 Genotype_Zone_1:Plant_Zone_1 (Intercept) 3.943e-02  0.1986 
 Genotype_Zone_2:Plant_Zone_2 (Intercept) 1.466e-01  0.3829 
 Residual                                  2.681e+03 51.7748 
Number of obs: 39, groups:  Genotype_Zone_1:Plant_Zone_1, 10; Genotype_Zone_2:Plant_Zone_2, 10

Fixed effects:
            Estimate Std. Error      df t value Pr(>|t|)   
(Intercept)  -1.1510     0.2139  4.2746  -5.381  0.00476 **
---
Signif. codes:  0 '***' 0.001 '**' 0.01 '*' 0.05 '.' 0.1 ' ' 1

Dispersion: 51.77
```

Model residuals

Statistic	Value
Sample skewness	0.5106
Sample excess kurtosis	0.3007
Passed Shapiro Wilk normality test	Yes (p-value = 0.4928 > 0.05)

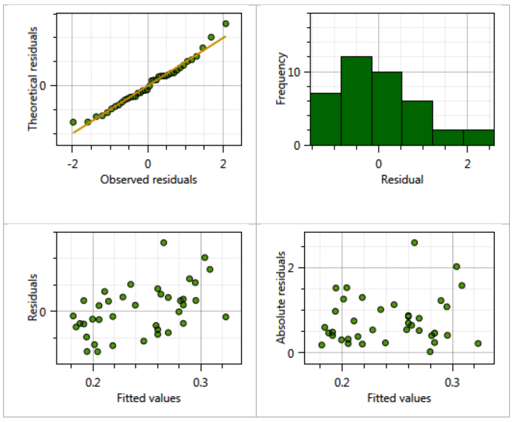

Estimated duration halting per zone per hour

Selected hours	0, 1, 2, 3, 4, 5, 6, 7
Selected zones	Zone 1, Zone 2
Data transformation	Natural logarithm
Analysis	Zone difference analysis

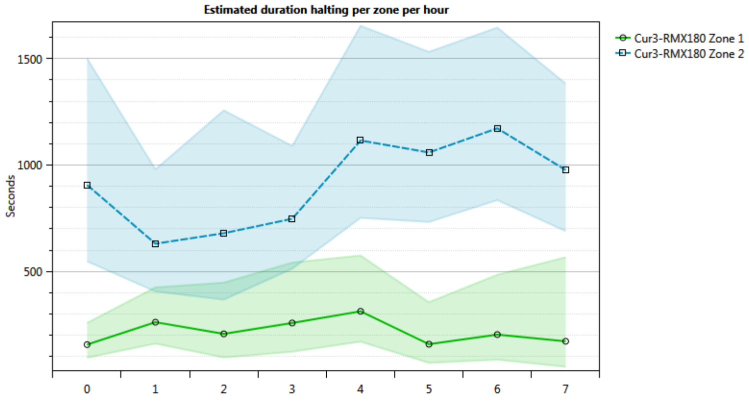

Results difference tests Zone 1 - Zone 2: p values and 95% confidence intervals of the difference on the transformed scale for each statistic.

Behaviour statistic	Cur3-RMX180	Remark
Estimated duration halting H0 (diff. Zone 1 - Zone 2)	p=8.57E-06****<br>[-2.02, -0.878]	CR
Estimated duration halting H1 (diff. Zone 1 - Zone 2)	p=0.0355*<br>[-1.22, -0.0535]	CR
Estimated duration halting H2 (diff. Zone 1 - Zone 2)	p=0.0822<br>[-2.12, 0.166]	
Estimated duration halting H3 (diff. Zone 1 - Zone 2)	p=0.0362*<br>[-1.75, -0.0734]	CR
Estimated duration halting H4 (diff. Zone 1 - Zone 2)	p=0.0377*<br>[-1.77, -0.0765]	CR
Estimated duration halting H5 (diff. Zone 1 - Zone 2)	p=0.00532**<br>[-1.94, -0.508]	CR
Estimated duration halting H6 (diff. Zone 1 - Zone 2)	p=0.00431**<br>[-2.66, -0.685]	CR
Estimated duration halting H7 (diff. Zone 1 - Zone 2)	p=0.00316**<br>[-2.51, -0.72]	CR

CR = Check residuals

The model predictions and 95% confidence intervals for each statistic.

Statistic	Cur3-RMX180	Remark
Estimated duration halting (H0 - Zone 1)	157<br>[94.7, 259]	
Estimated duration halting (H0 - Zone 2)	907<br>[548, 1.5E+03]	CR
Estimated duration halting (H1 - Zone 1)	262<br>[161, 426]	
Estimated duration halting (H1 - Zone 2)	631<br>[406, 980]	CR
Estimated duration halting (H2 - Zone 1)	207<br>[95.7, 448]	
Estimated duration halting (H2 - Zone 2)	680<br>[368, 1.26E+03]	CR
Estimated duration halting (H3 - Zone 1)	258<br>[123, 542]	CR
Estimated duration halting (H3 - Zone 2)	748<br>[513, 1.09E+03]	CR
Estimated duration halting (H4 - Zone 1)	313<br>[170, 575]	
Estimated duration halting (H4 - Zone 2)	1.12E+03<br>[752, 1.65E+03]	CR
Estimated duration halting (H5 - Zone 1)	158<br>[70.6, 356]	CR
Estimated duration halting (H5 - Zone 2)	1.06E+03<br>[732, 1.53E+03]	CR
Estimated duration halting (H6 - Zone 1)	204<br>[85.6, 485]	
Estimated duration halting (H6 - Zone 2)	1.17E+03<br>[835, 1.65E+03]	CR
Estimated duration halting (H7 - Zone 1)	172<br>[52.2, 567]	
Estimated duration halting (H7 - Zone 2)	977<br>[690, 1.38E+03]	CR

CR = Check residuals

Data summary

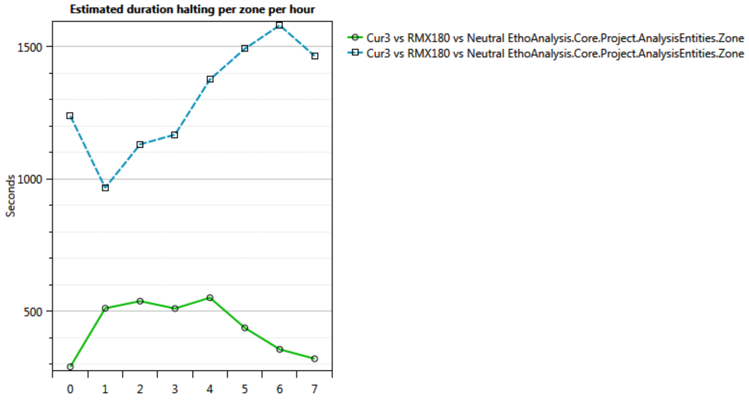

Genotype Zone 1	Genotype Zone 2	Genotype Zone 3	Mean H0 - Zone 1	StdErr H0 - Zone 1	Mean H0 - Zone 2	StdErr H0 - Zone 2	Mean H1 - Zone 1	StdErr H1 - Zone 1	Mean H1 - Zone 2	StdErr H1 - Zone 2	Mean H2 - Zone 1	StdErr H2 - Zone 1	Mean H2 - Zone 2	StdErr H2 - Zone 2	Mean H3 - Zone 1	StdErr H3 - Zone 1	Mean H3 - Zone 2	StdErr H3 - Zone 2	Mean H4 - Zone 1	StdErr H4 - Zone 1	Mean H4 - Zone 2	StdErr H4 - Zone 2	Mean H5 - Zone 1	StdErr H5 - Zone 1	Mean H5 - Zone 2	StdErr H5 - Zone 2	Mean H6 - Zone 1	StdErr H6 - Zone 1	Mean H6 - Zone
Cur3	RMX180	Neutral	291.2	58.13	1238.57	145.36	512.12	109.81	967.43	125.92	539.12	141.49	1131.43	132.56	511.28	99.56	1168.38	149.95	552.16	133.51	1376.35	142.87	438.18	100.6	1492.45	153.85	356.98	111.39	1581.

Analysis estimated duration halting (H0 - Zone 1)

**Analysis model**	Linear mixed model fit by REML: Estimated_duration_halting_H0_Zone_1 ~ 1 + (1|Genotype_Zone_1:Plant_Zone_1) + (1|Genotype_Zone_2:Plant_Zone_2)
**Transformation**	Natural logarithm

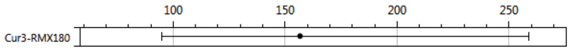

Confidence intervals estimated duration halting (H0 - Zone 1)

Genotype Zone 1	Genotype Zone 2	Mean	Lower 95% CL	Upper 95% CL	Group
Cur3	RMX180	156.6	94.72	259	a

Model summary

```
Linear mixed model fit by REML. t-tests use Satterthwaite's method ['lmerModLmerTest']
Formula: Estimated_duration_halting_H0_Zone_1 ~ 1 + (1 | Genotype_Zone_1:Plant_Zone_1) +      (1 |
Genotype_Zone_2:Plant_Zone_2)
   Data: data

REML criterion at convergence: 121.3

Scaled residuals: 
     Min       1Q   Median       3Q      Max 
-2.35861 -0.81239  0.07999  0.76496  1.51131 

Random effects:
 Groups                       Name        Variance  Std.Dev. 
 Genotype_Zone_1:Plant_Zone_1 (Intercept) 2.409e-16 1.552e-08
 Genotype_Zone_2:Plant_Zone_2 (Intercept) 2.818e-16 1.679e-08
 Residual                                 2.077e+00 1.441e+00
Number of obs: 34, groups:  Genotype_Zone_1:Plant_Zone_1, 10; Genotype_Zone_2:Plant_Zone_2, 10

Fixed effects:
            Estimate Std. Error      df t value Pr(>|t|)    
(Intercept)   5.0538     0.2472 33.0000   20.45   <2e-16 ***
---
Signif. codes:  0 '***' 0.001 '**' 0.01 '*' 0.05 '.' 0.1 ' ' 1
```

Model residuals

Statistic	Value
Sample skewness	-0.4864
Sample excess kurtosis	-0.5283
Passed Shapiro Wilk normality test	Yes (p-value = 0.2676 > 0.05)

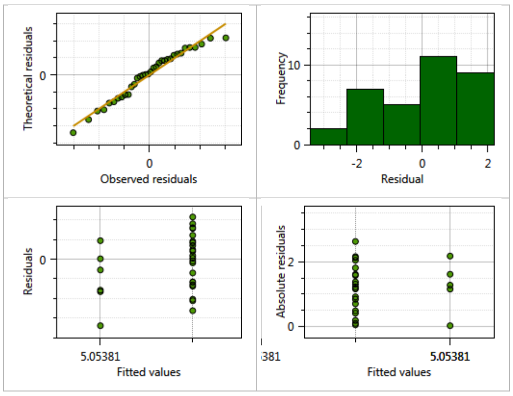

Analysis estimated duration halting (H0 - Zone 2)

**Analysis model**	Linear mixed model fit by REML: Estimated_duration_halting_H0_Zone_2 ~ 1 + (1|Genotype_Zone_1:Plant_Zone_1) + (1|Genotype_Zone_2:Plant_Zone_2)
**Transformation**	Natural logarithm

Confidence intervals estimated duration halting (H0 - Zone 2)

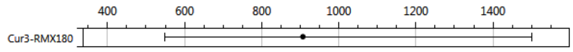

Confidence intervals estimated duration halting (H0 - Zone 2)

Genotype Zone 1	Genotype Zone 2	Mean	Lower 95% CL	Upper 95% CL	Group
Cur3	RMX180	906.8	547.6	1501	a

Model summary

```
Linear mixed model fit by REML. t-tests use Satterthwaite's method ['lmerModLmerTest']
Formula: Estimated_duration_halting_H0_Zone_2 ~ 1 + (1 | Genotype_Zone_1:Plant_Zone_1) +      (1 |
Genotype_Zone_2:Plant_Zone_2)
   Data: data

REML criterion at convergence: 115.1

Scaled residuals:
    Min      1Q  Median      3Q     Max
-4.4002 -0.2869  0.1681  0.6036  1.3422

Random effects:
 Groups                       Name        Variance Std.Dev.
 Genotype_Zone_1:Plant_Zone_1 (Intercept) 0.01672  0.1293
 Genotype_Zone_2:Plant_Zone_2 (Intercept) 0.02089  0.1445
 Residual                                 1.15634  1.0753
Number of obs: 38, groups:  Genotype_Zone_1:Plant_Zone_1, 10; Genotype_Zone_2:Plant_Zone_2, 10

Fixed effects:
            Estimate Std. Error    df t value Pr(>|t|)
(Intercept)    6.810      0.185 4.198   36.81 1.98e-06 ***
---
Signif. codes:  0 '***' 0.001 '**' 0.01 '*' 0.05 '.' 0.1 ' ' 1
```

Model residuals

Statistic	Value
Sample skewness	-2.519
Sample excess kurtosis	10.07
Passed Shapiro Wilk normality test	No (p-value = 8.107E-06 < 0.05)

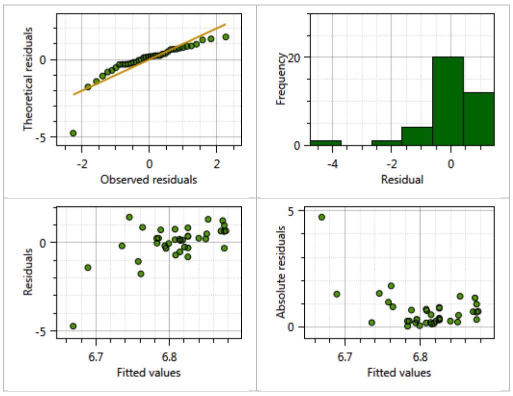

Analysis estimated duration halting (H1 - Zone 1)

**Analysis model**	Linear mixed model fit by REML: Estimated_duration_halting_H1_Zone_1 ~ 1 + (1|Genotype_Zone_1:Plant_Zone_1) + (1|Genotype_Zone_2:Plant_Zone_2)
**Transformation**	Natural logarithm

Confidence intervals estimated duration halting (H1 - Zone 1)

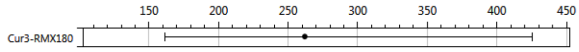

Confidence intervals estimated duration halting (H1 - Zone 1)

Genotype Zone 1	Genotype Zone 2	Mean	Lower 95% CL	Upper 95% CL	Group
Cur3	RMX180	261.9	161.2	425.6	a

Model summary

```
Linear mixed model fit by REML. t-tests use Satterthwaite's method ['lmerModLmerTest']
Formula: Estimated_duration_halting_H1_Zone_1 ~ 1 + (1 | Genotype_Zone_1:Plant_Zone_1) +      (1 |
Genotype_Zone_2:Plant_Zone_2)
   Data: data

REML criterion at convergence: 128.2

Scaled residuals:
    Min      1Q  Median      3Q     Max
-3.0092 -0.5418  0.1177  0.7546  1.8232

Random effects:
 Groups                       Name        Variance Std.Dev.
 Genotype_Zone_1:Plant_Zone_1 (Intercept) 0.000    0.000
 Genotype_Zone_2:Plant_Zone_2 (Intercept) 0.000    0.000
```

```
 Residual                                 2.059    1.435
Number of obs: 36, groups:  Genotype_Zone_1:Plant_Zone_1, 10; Genotype_Zone_2:Plant_Zone_2, 10

Fixed effects:
            Estimate Std. Error      df t value Pr(>|t|)
(Intercept)   5.5680     0.2392 35.0000   23.28   <2e-16 ***
---
Signif. codes:  0 '***' 0.001 '**' 0.01 '*' 0.05 '.' 0.1 ' ' 1
```

Model residuals

Statistic	Value
Sample skewness	-0.7535
Sample excess kurtosis	0.9482
Passed Shapiro Wilk normality test	Yes (p-value = 0.2887 > 0.05)

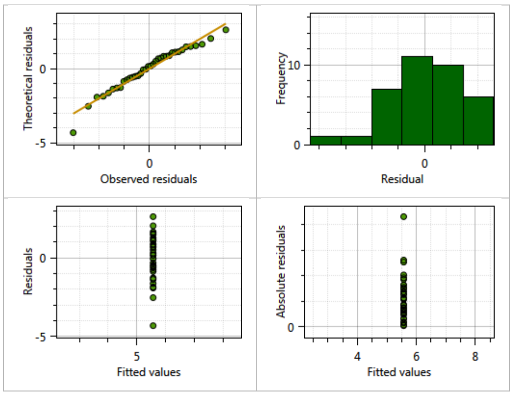

Analysis estimated duration halting (H1 - Zone 2)

Analysis model	Linear mixed model fit by REML: Estimated_duration_halting_H1_Zone_2 ~ 1 + (1|Genotype_Zone_1:Plant_Zone_1) + (1|Genotype_Zone_2:Plant_Zone_2)
Transformation	Natural logarithm

Confidence intervals estimated duration halting (H1 - Zone 2)

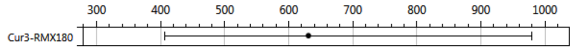

Confidence intervals estimated duration halting (H1 - Zone 2)

Genotype Zone 1	Genotype Zone 2	Mean	Lower 95% CL	Upper 95% CL	Group
Cur3	RMX180	630.7	405.9	980	a

Model summary

```
Linear mixed model fit by REML. t-tests use Satterthwaite's method ['lmerModLmerTest']
Formula: Estimated_duration_halting_H1_Zone_2 ~ 1 + (1 | Genotype_Zone_1:Plant_Zone_1) +      (1 |
Genotype_Zone_2:Plant_Zone_2)
   Data: data

REML criterion at convergence: 130.3

Scaled residuals:
    Min      1Q  Median      3Q     Max
-4.1486 -0.2031  0.2960  0.5615  1.2990

Random effects:
 Groups                       Name        Variance Std.Dev.
 Genotype_Zone_1:Plant_Zone_1 (Intercept) 0.000    0.000
 Genotype_Zone_2:Plant_Zone_2 (Intercept) 0.000    0.000
 Residual                                 1.798    1.341
Number of obs: 38, groups:  Genotype_Zone_1:Plant_Zone_1, 10; Genotype_Zone_2:Plant_Zone_2, 10

Fixed effects:
            Estimate Std. Error      df t value Pr(>|t|)
(Intercept)   6.4468     0.2175 37.0000   29.64   <2e-16 ***
---
Signif. codes:  0 '***' 0.001 '**' 0.01 '*' 0.05 '.' 0.1 ' ' 1
```

Model residuals

Statistic	Value
Sample skewness	-2.49
Sample excess kurtosis	8.246
Passed Shapiro Wilk normality test	No (p-value = 2.319E-06 < 0.05)

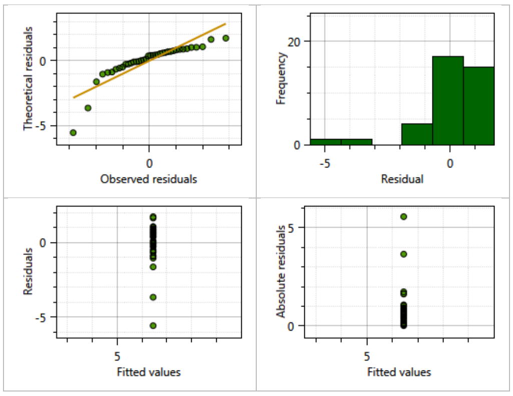

Analysis estimated duration halting (H2 - Zone 1)

Analysis model	Linear mixed model fit by REML: Estimated_duration_halting_H2_Zone_1 ~ 1 + (1|Genotype_Zone_1:Plant_Zone_1) + (1|Genotype_Zone_2:Plant_Zone_2)
Transformation	Natural logarithm

Confidence intervals estimated duration halting (H2 - Zone 1)

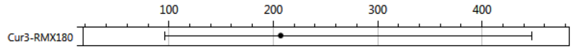

Confidence intervals estimated duration halting (H2 - Zone 1)

Genotype Zone 1	Genotype Zone 2	Mean	Lower 95% CL	Upper 95% CL	Group
Cur3	RMX180	207	95.68	447.9	a

Model summary

```
Linear mixed model fit by REML. t-tests use Satterthwaite's method ['lmerModLmerTest']
Formula: Estimated_duration_halting_H2_Zone_1 ~ 1 + (1 | Genotype_Zone_1:Plant_Zone_1) +       (1 |
Genotype_Zone_2:Plant_Zone_2)
   Data: data

REML criterion at convergence: 132.4

Scaled residuals: 
     Min       1Q   Median       3Q      Max 
-2.06685 -0.76585  0.00738  0.74825  1.75024 

Random effects:
 Groups                       Name        Variance Std.Dev.
 Genotype_Zone_1:Plant_Zone_1 (Intercept) 0.54744  0.7399  
 Genotype_Zone_2:Plant_Zone_2 (Intercept) 0.05037  0.2244  
 Residual                                 1.90071  1.3787  
Number of obs: 36, groups:  Genotype_Zone_1:Plant_Zone_1, 10; Genotype_Zone_2:Plant_Zone_2, 10

Fixed effects:
            Estimate Std. Error     df t value Pr(>|t|)    
(Intercept)   5.3328     0.3372 8.3689   15.81 1.59e-07 ***
---
Signif. codes:  0 '***' 0.001 '**' 0.01 '*' 0.05 '.' 0.1 ' ' 1
```

Model residuals

Statistic	Value
Sample skewness	-0.07747
Sample excess kurtosis	-0.6465
Passed Shapiro Wilk normality test	Yes (p-value = 0.7765 > 0.05)

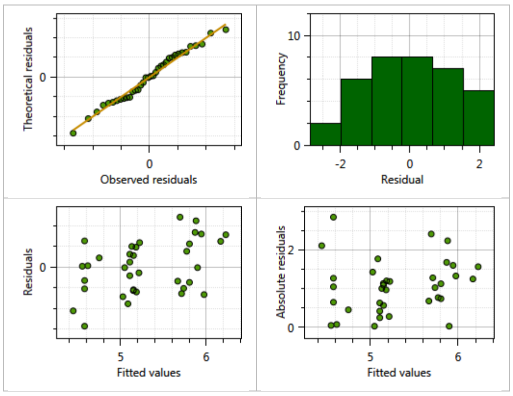

Analysis estimated duration halting (H2 - Zone 2)

**Analysis model**	Linear mixed model fit by REML: Estimated_duration_halting_H2_Zone_2 ~ 1 + (1|Genotype_Zone_1:Plant_Zone_1) + (1|Genotype_Zone_2:Plant_Zone_2)
**Transformation**	Natural logarithm

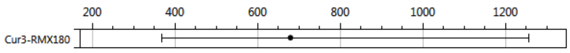

Confidence intervals estimated duration halting (H2 - Zone 2)

Genotype Zone 1	Genotype Zone 2	Mean	Lower 95% CL	Upper 95% CL	Group
Cur3	RMX180	679.9	367.6	1257	a

Model summary

```
Linear mixed model fit by REML. t-tests use Satterthwaite's method ['lmerModLmerTest']
Formula: Estimated_duration_halting_H2_Zone_2 ~ 1 + (1 | Genotype_Zone_1:Plant_Zone_1) +       (1 |
Genotype_Zone_2:Plant_Zone_2)
   Data: data

REML criterion at convergence: 145.3

Scaled residuals: 
    Min      1Q  Median      3Q     Max 
-4.6586 -0.0686  0.3280  0.4798  0.9525 

Random effects:
 Groups                       Name        Variance Std.Dev.
 Genotype_Zone_1:Plant_Zone_1 (Intercept) 0.00000  0.0000  
 Genotype_Zone_2:Plant_Zone_2 (Intercept) 0.04701  0.2168  
 Residual                                 2.65150  1.6283  
Number of obs: 38, groups:  Genotype_Zone_1:Plant_Zone_1, 10; Genotype_Zone_2:Plant_Zone_2, 10

Fixed effects:
            Estimate Std. Error    df t value Pr(>|t|)    
(Intercept)    6.522      0.273 9.270   23.89 1.21e-09 ***
---
Signif. codes:  0 '***' 0.001 '**' 0.01 '*' 0.05 '.' 0.1 ' ' 1
```

Model residuals

Statistic	Value
Sample skewness	-3.091
Sample excess kurtosis	12.67
Passed Shapiro Wilk normality test	No (p-value = 1.037E-07 < 0.05)

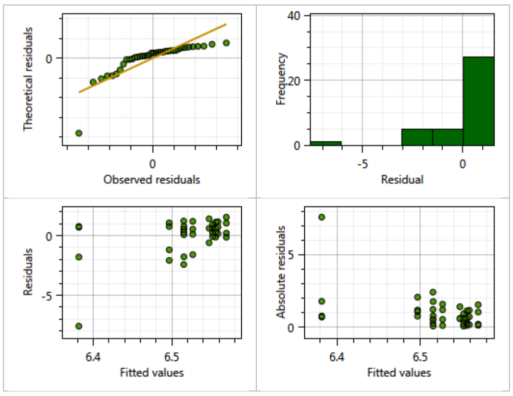

Analysis estimated duration halting (H3 - Zone 1)

**Analysis model**	Linear mixed model fit by REML: Estimated_duration_halting_H3_Zone_1 ~ 1 + (1|Genotype_Zone_1:Plant_Zone_1) + (1|Genotype_Zone_2:Plant_Zone_2)
**Transformation**	Natural logarithm

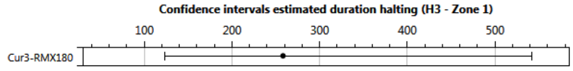

Confidence intervals estimated duration halting (H3 - Zone 1)

Genotype Zone 1	Genotype Zone 2	Mean	Lower 95% CL	Upper 95% CL	Group
Cur3	RMX180	258.1	122.9	542.2	a

Model summary

```
Linear mixed model fit by REML. t-tests use Satterthwaite's method ['lmerModLmerTest']
Formula: Estimated_duration_halting_H3_Zone_1 ~ 1 + (1 | Genotype_Zone_1:Plant_Zone_1) +      (1 |
Genotype_Zone_2:Plant_Zone_2)
   Data: data

REML criterion at convergence: 129.5

Scaled residuals:
    Min      1Q  Median      3Q     Max
-2.7107 -0.3185  0.2526  0.5901  1.2360

Random effects:
 Groups                       Name        Variance Std.Dev.
 Genotype_Zone_1:Plant_Zone_1 (Intercept) 0.0000   0.0000
 Genotype_Zone_2:Plant_Zone_2 (Intercept) 0.2018   0.4493
 Residual                                 2.8338   1.6834
Number of obs: 33, groups:  Genotype_Zone_1:Plant_Zone_1, 10; Genotype_Zone_2:Plant_Zone_2, 10

Fixed effects:
            Estimate Std. Error     df t value Pr(>|t|)
(Intercept)   5.5535     0.3271 8.8239   16.98 4.84e-08 ***
---
Signif. codes:  0 '***' 0.001 '**' 0.01 '*' 0.05 '.' 0.1 ' ' 1
```

Model residuals

Statistic	Value
Sample skewness	-1.269
Sample excess kurtosis	1.266
Passed Shapiro Wilk normality test	No (p-value = 0.002329 < 0.05)

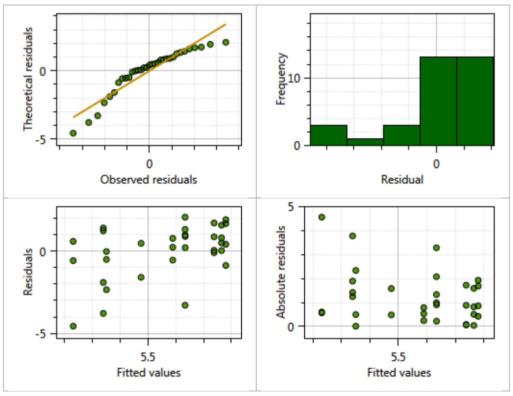

Analysis estimated duration halting (H3 - Zone 2)

**Analysis model**	Linear mixed model fit by REML: Estimated_duration_halting_H3_Zone_2 ~ 1 + (1|Genotype_Zone_1:Plant_Zone_1) + (1|Genotype_Zone_2:Plant_Zone_2)
**Transformation**	Natural logarithm

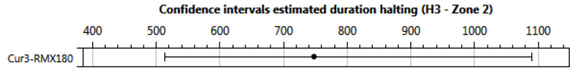

Confidence intervals estimated duration halting (H3 - Zone 2)

Genotype Zone 1	Genotype Zone 2	Mean	Lower 95% CL	Upper 95% CL	Group
Cur3	RMX180	747.7	512.8	1090	a

Model summary

```
Linear mixed model fit by REML. t-tests use Satterthwaite's method ['lmerModLmerTest']
Formula: Estimated_duration_halting_H3_Zone_2 ~ 1 + (1 | Genotype_Zone_1:Plant_Zone_1) +      (1 |
Genotype_Zone_2:Plant_Zone_2)
   Data: data

REML criterion at convergence: 123

Scaled residuals:
    Min      1Q  Median      3Q     Max
-3.0200 -0.3606  0.1466  0.5928  1.3059

Random effects:
 Groups                       Name        Variance Std.Dev.
 Genotype_Zone_1:Plant_Zone_1 (Intercept) 0.000    0.000
 Genotype_Zone_2:Plant_Zone_2 (Intercept) 0.000    0.000
 Residual                                 1.354    1.163
Number of obs: 39, groups:  Genotype_Zone_1:Plant_Zone_1, 10; Genotype_Zone_2:Plant_Zone_2, 10

Fixed effects:
            Estimate Std. Error      df t value Pr(>|t|)
(Intercept)   6.6170     0.1863 38.0000   35.52   <2e-16 ***
---
Signif. codes:  0 '***' 0.001 '**' 0.01 '*' 0.05 '.' 0.1 ' ' 1
```

Model residuals

Statistic	Value
Sample skewness	-1.28
Sample excess kurtosis	1.828
Passed Shapiro Wilk normality test	No (p-value = 0.001648 < 0.05)

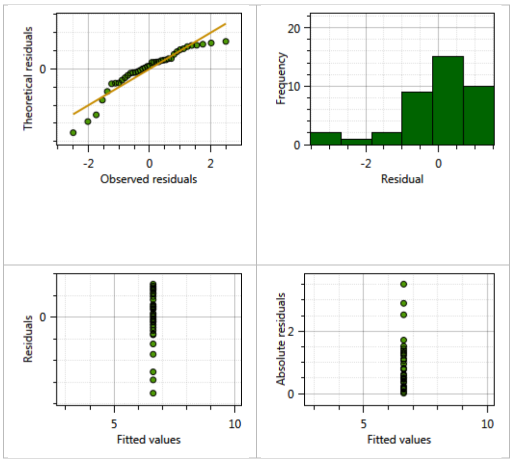

Analysis estimated duration halting (H4 - Zone 1)

**Analysis model**	Linear mixed model fit by REML: Estimated_duration_halting_H4_Zone_1 ~ 1 + (1|Genotype_Zone_1:Plant_Zone_1) + (1|Genotype_Zone_2:Plant_Zone_2)
**Transformation**	Natural logarithm

Confidence intervals estimated duration halting (H4 - Zone 1)

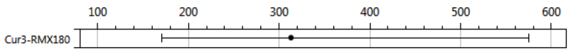

Confidence intervals estimated duration halting (H4 - Zone 1)

Genotype Zone 1	Genotype Zone 2	Mean	Lower 95% CL	Upper 95% CL	Group
Cur3	RMX180	313	170.3	575.4	a

Model summary

```
Linear mixed model fit by REML. t-tests use Satterthwaite's method ['lmerModLmerTest']
Formula: Estimated_duration_halting_H4_Zone_1 ~ 1 + (1 | Genotype_Zone_1:Plant_Zone_1) +      (1 |
Genotype_Zone_2:Plant_Zone_2)
   Data: data

REML criterion at convergence: 109.2

Scaled residuals:
    Min      1Q  Median      3Q     Max
-2.2846 -0.4234  0.1660  0.7482  1.4785

Random effects:
 Groups                       Name        Variance  Std.Dev.
 Genotype_Zone_1:Plant_Zone_1 (Intercept) 1.190e-15 3.450e-08
 Genotype_Zone_2:Plant_Zone_2 (Intercept) 0.000e+00 0.000e+00
 Residual                                 2.562e+00 1.601e+00
Number of obs: 29, groups:  Genotype_Zone_1:Plant_Zone_1, 10; Genotype_Zone_2:Plant_Zone_2, 10

Fixed effects:
            Estimate Std. Error      df t value Pr(>|t|)
(Intercept)   5.7462     0.2972 28.0000   19.33   <2e-16 ***
---
Signif. codes:  0 '***' 0.001 '**' 0.01 '*' 0.05 '.' 0.1 ' ' 1
```

Model residuals

Statistic	Value
Sample skewness	-0.7149
Sample excess kurtosis	0.1012
Passed Shapiro Wilk normality test	Yes (p-value = 0.1527 > 0.05)

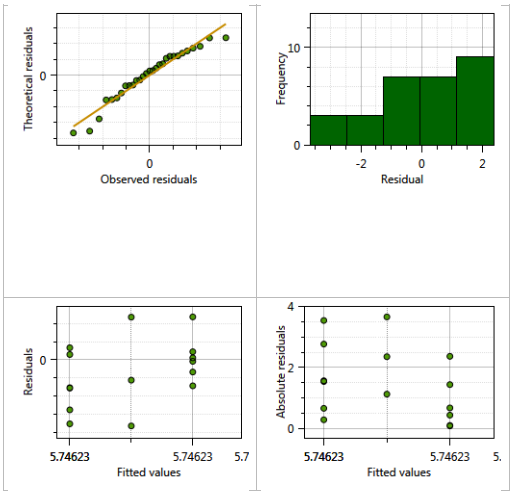

Analysis estimated duration halting (H4 - Zone 2)

**Analysis model**	Linear mixed model fit by REML: Estimated_duration_halting_H4_Zone_2 ~ 1 + (1|Genotype_Zone_1:Plant_Zone_1) + (1|Genotype_Zone_2:Plant_Zone_2)
**Transformation**	Natural logarithm

Confidence intervals estimated duration halting (H4 - Zone 2)

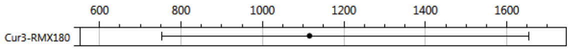

Confidence intervals estimated duration halting (H4 - Zone 2)

Genotype Zone 1	Genotype Zone 2	Mean	Lower 95% CL	Upper 95% CL	Group
Cur3	RMX180	1115	751.9	1655	a

Model summary

```
Linear mixed model fit by REML. t-tests use Satterthwaite's method ['lmerModLmerTest']
Formula: Estimated_duration_halting_H4_Zone_2 ~ 1 + (1 | Genotype_Zone_1:Plant_Zone_1) +      (1 |
Genotype_Zone_2:Plant_Zone_2)
   Data: data
```

```
REML criterion at convergence: 98.3

Scaled residuals:
    Min      1Q  Median      3Q     Max
-3.3190 -0.2917  0.1053  0.5543  1.2091

Random effects:
 Groups                       Name        Variance Std.Dev.
 Genotype_Zone_1:Plant_Zone_1 (Intercept) 0.0000   0.0000
 Genotype_Zone_2:Plant_Zone_2 (Intercept) 0.1076   0.3281
 Residual                                 0.7287   0.8536
Number of obs: 37, groups:  Genotype_Zone_1:Plant_Zone_1, 10; Genotype_Zone_2:Plant_Zone_2, 10

Fixed effects:
            Estimate Std. Error     df t value Pr(>|t|)
(Intercept)   7.0170     0.1752 9.3122   40.05 9.62e-12 ***
---
Signif. codes:  0 '***' 0.001 '**' 0.01 '*' 0.05 '.' 0.1 ' ' 1
```

Model residuals

Statistic	Value
Sample skewness	-1.568
Sample excess kurtosis	3.249
Passed Shapiro Wilk normality test	No (p-value = 0.0005907 < 0.05)

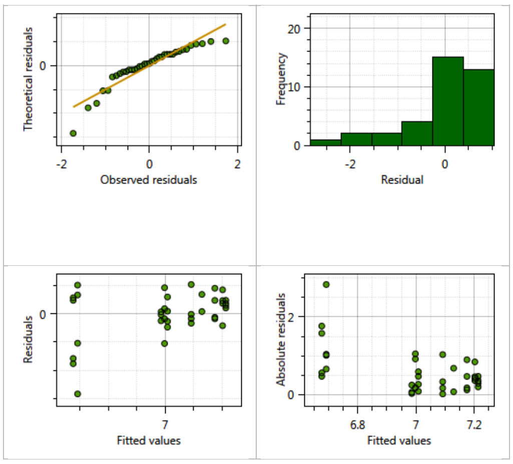

Analysis estimated duration halting (H5 - Zone 1)

**Analysis model**	Linear mixed model fit by REML: Estimated_duration_halting_H5_Zone_1 ~ 1 + (1|Genotype_Zone_1:Plant_Zone_1) + (1|Genotype_Zone_2:Plant_Zone_2)
**Transformation**	Natural logarithm

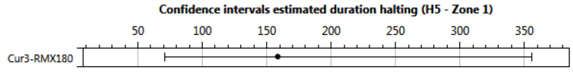

Confidence intervals estimated duration halting (H5 - Zone 1)

Genotype Zone 1	Genotype Zone 2	Mean	Lower 95% CL	Upper 95% CL	Group
Cur3	RMX180	158.5	70.55	356	a

Model summary

```
Linear mixed model fit by REML. t-tests use Satterthwaite's method ['lmerModLmerTest']
Formula: Estimated_duration_halting_H5_Zone_1 ~ 1 + (1 | Genotype_Zone_1:Plant_Zone_1) +      (1 |
Genotype_Zone_2:Plant_Zone_2)
   Data: data

REML criterion at convergence: 136

Scaled residuals:
    Min      1Q  Median      3Q     Max
-2.8418 -0.9246  0.3110  0.7377  1.3042

Random effects:
 Groups                       Name        Variance Std.Dev.
 Genotype_Zone_1:Plant_Zone_1 (Intercept) 0.000    0.000
 Genotype_Zone_2:Plant_Zone_2 (Intercept) 0.000    0.000
 Residual                                 4.867    2.206
Number of obs: 31, groups:  Genotype_Zone_1:Plant_Zone_1, 10; Genotype_Zone_2:Plant_Zone_2, 10

Fixed effects:
            Estimate Std. Error      df t value Pr(>|t|)
(Intercept)   5.0656     0.3962 30.0000   12.78 1.13e-13 ***
---
Signif. codes:  0 '***' 0.001 '**' 0.01 '*' 0.05 '.' 0.1 ' ' 1
```

Model residuals

Statistic	Value
Sample skewness	-1.016
Sample excess kurtosis	0.5299
Passed Shapiro Wilk normality test	No (p-value = 0.005691 < 0.05)

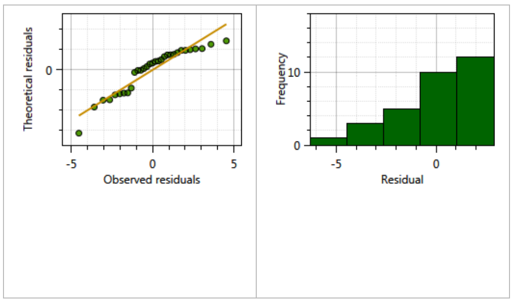

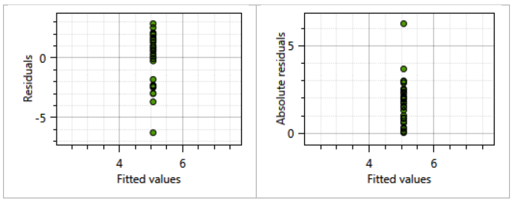

Analysis estimated duration halting (H5 - Zone 2)

**Analysis model**	Linear mixed model fit by REML: Estimated_duration_halting_H5_Zone_2 ~ 1 + (1|Genotype_Zone_1:Plant_Zone_1) + (1|Genotype_Zone_2:Plant_Zone_2)
**Transformation**	Natural logarithm

Confidence intervals estimated duration halting (H5 - Zone 2)

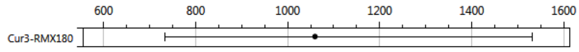

Confidence intervals estimated duration halting (H5 - Zone 2)

Genotype Zone 1	Genotype Zone 2	Mean	Lower 95% CL	Upper 95% CL	Group
Cur3	RMX180	1059	732.4	1532	a

Model summary

```
Linear mixed model fit by REML. t-tests use Satterthwaite's method ['lmerModLmerTest']
Formula: Estimated_duration_halting_H5_Zone_2 ~ 1 + (1 | Genotype_Zone_1:Plant_Zone_1) +      (1 |
Genotype_Zone_2:Plant_Zone_2)
   Data: data

REML criterion at convergence: 111.1

Scaled residuals: 
    Min      1Q  Median      3Q     Max 
-2.4053 -0.5114  0.4624  0.6561  1.1663 

Random effects:
 Groups                       Name        Variance Std.Dev.
 Genotype_Zone_1:Plant_Zone_1 (Intercept) 0.01862  0.1365  
 Genotype_Zone_2:Plant_Zone_2 (Intercept) 0.00000  0.0000  
 Residual                                 0.97176  0.9858  
Number of obs: 39, groups:  Genotype_Zone_1:Plant_Zone_1, 10; Genotype_Zone_2:Plant_Zone_2, 10

Fixed effects:
            Estimate Std. Error     df t value Pr(>|t|)    
(Intercept)   6.9654     0.1637 9.2042   42.56 6.95e-12 ***
---
Signif. codes:  0 '***' 0.001 '**' 0.01 '*' 0.05 '.' 0.1 ' ' 1
```

Model residuals

Statistic	Value
Sample skewness	-1.012
Sample excess kurtosis	-0.09354
Passed Shapiro Wilk normality test	No (p-value = 0.0003484 < 0.05)

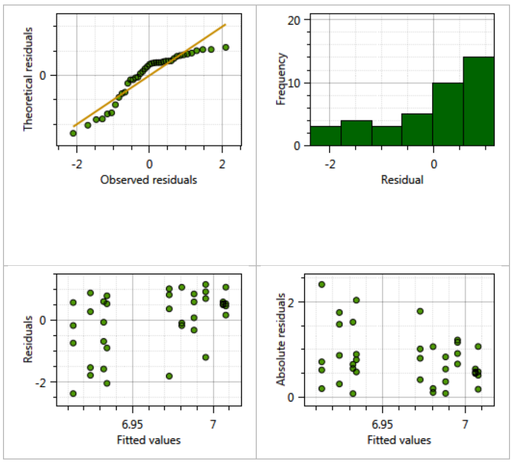

Analysis estimated duration halting (H6 - Zone 1)

**Analysis model**	Linear mixed model fit by REML: Estimated_duration_halting_H6_Zone_1 ~ 1 + (1|Genotype_Zone_1:Plant_Zone_1) + (1|Genotype_Zone_2:Plant_Zone_2)
**Transformation**	Natural logarithm

Confidence intervals estimated duration halting (H6 - Zone 1)

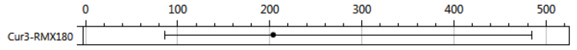

Confidence intervals estimated duration halting (H6 - Zone 1)

Genotype Zone 1	Genotype Zone 2	Mean	Lower 95% CL	Upper 95% CL	Group
Cur3	RMX180	203.7	85.61	484.8	a

Model summary

```
Linear mixed model fit by REML. t-tests use Satterthwaite's method ['lmerModLmerTest']
Formula: Estimated_duration_halting_H6_Zone_1 ~ 1 + (1 | Genotype_Zone_1:Plant_Zone_1) +      (1 |
Genotype_Zone_2:Plant_Zone_2)
   Data: data

REML criterion at convergence: 99.2

Scaled residuals: 
    Min      1Q  Median      3Q     Max 
-1.9606 -0.4628  0.0174  0.5498  1.3994 

Random effects:
 Groups                       Name        Variance Std.Dev.
 Genotype_Zone_1:Plant_Zone_1 (Intercept) 0.1225   0.3500  
 Genotype_Zone_2:Plant_Zone_2 (Intercept) 0.4207   0.6486  
 Residual                                 1.9137   1.3834  
Number of obs: 27, groups:  Genotype_Zone_1:Plant_Zone_1, 10; Genotype_Zone_2:Plant_Zone_2, 10

Fixed effects:
            Estimate Std. Error    df t value Pr(>|t|)    
(Intercept)    5.317      0.362 6.588   14.69  2.8e-06 ***
---
Signif. codes:  0 '***' 0.001 '**' 0.01 '*' 0.05 '.' 0.1 ' ' 1
```

Model residuals

Statistic	Value
Sample skewness	-0.499
Sample excess kurtosis	-0.2463
Passed Shapiro Wilk normality test	Yes (p-value = 0.3156 > 0.05)

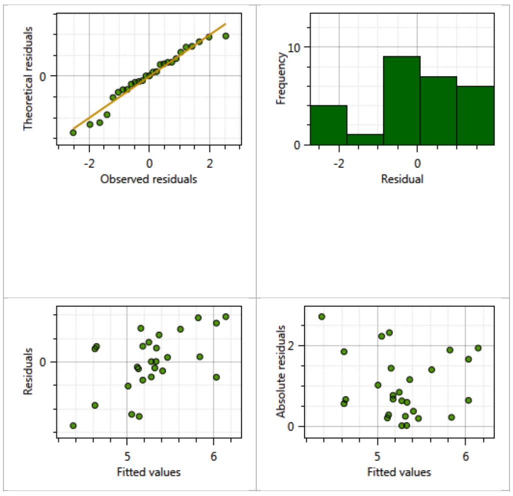

Analysis estimated duration halting (H6 - Zone 2)

Analysis model	Linear mixed model fit by REML: Estimated_duration_halting_H6_Zone_2 ~ 1 + (1|Genotype_Zone_1:Plant_Zone_1) + (1|Genotype_Zone_2:Plant_Zone_2)
Transformation	Natural logarithm

Confidence intervals estimated duration halting (H6 - Zone 2)

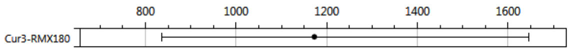

Confidence intervals estimated duration halting (H6 - Zone 2)

Genotype Zone 1	Genotype Zone 2	Mean	Lower 95% CL	Upper 95% CL	Group
Cur3	RMX180	1173	835.2	1646	a

Model summary

```
Linear mixed model fit by REML. t-tests use Satterthwaite's method ['lmerModLmerTest']
Formula: Estimated_duration_halting_H6_Zone_2 ~ 1 + (1 | Genotype_Zone_1:Plant_Zone_1) +      (1 |
Genotype_Zone_2:Plant_Zone_2)
   Data: data

REML criterion at convergence: 111

Scaled residuals:
    Min      1Q  Median      3Q     Max
-3.7085 -0.5702  0.3798  0.5913  1.0640

Random effects:
 Groups                       Name        Variance Std.Dev.
 Genotype_Zone_1:Plant_Zone_1 (Intercept) 0.000    0.000
 Genotype_Zone_2:Plant_Zone_2 (Intercept) 0.000    0.000
 Residual                                 1.066    1.032
Number of obs: 38, groups:  Genotype_Zone_1:Plant_Zone_1, 10; Genotype_Zone_2:Plant_Zone_2, 10

Fixed effects:
            Estimate Std. Error      df t value Pr(>|t|)
(Intercept)   7.0669     0.1675 37.0000    42.2   <2e-16 ***
---
Signif. codes:  0 '***' 0.001 '**' 0.01 '*' 0.05 '.' 0.1 ' ' 1
```

Model residuals

Statistic	Value
Sample skewness	-1.858
Sample excess kurtosis	4.53
Passed Shapiro Wilk normality test	No (p-value = 3.766E-05 < 0.05)

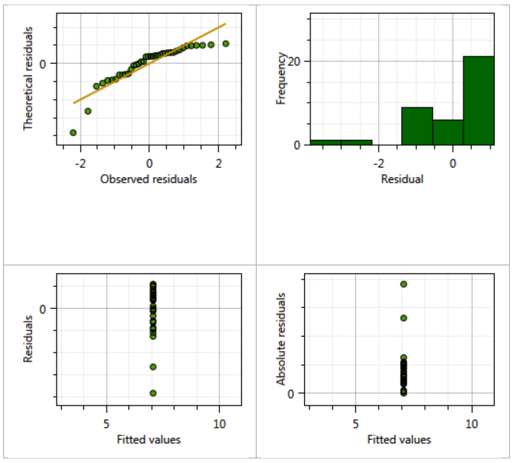

Analysis estimated duration halting (H7 - Zone 1)

Analysis model	Linear mixed model fit by REML: Estimated_duration_halting_H7_Zone_1 ~ 1 + (1|Genotype_Zone_1:Plant_Zone_1) + (1|Genotype_Zone_2:Plant_Zone_2)
Transformation	Natural logarithm

Confidence intervals estimated duration halting (H7 - Zone 1)

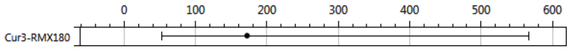

Confidence intervals estimated duration halting (H7 - Zone 1)

Genotype Zone 1	Genotype Zone 2	Mean	Lower 95% CL	Upper 95% CL	Group
Cur3	RMX180	172.1	52.21	567.2	a

Model summary

```
Linear mixed model fit by REML. t-tests use Satterthwaite's method ['lmerModLmerTest']
Formula: Estimated_duration_halting_H7_Zone_1 ~ 1 + (1 | Genotype_Zone_1:Plant_Zone_1) +      (1 |
Genotype_Zone_2:Plant_Zone_2)
   Data: data

REML criterion at convergence: 110.3

Scaled residuals:
    Min      1Q  Median      3Q     Max
-2.5840 -0.3872  0.2228  0.6594  1.3677

Random effects:
 Groups                       Name        Variance Std.Dev.
 Genotype_Zone_2:Plant_Zone_2 (Intercept) 0.9808   0.9903
 Genotype_Zone_1:Plant_Zone_1 (Intercept) 0.5706   0.7554
 Residual                                 2.4893   1.5777
Number of obs: 27, groups:  Genotype_Zone_2:Plant_Zone_2, 10; Genotype_Zone_1:Plant_Zone_1, 9

Fixed effects:
            Estimate Std. Error     df t value Pr(>|t|)
(Intercept)   5.1480     0.5184 8.1088    9.93 8.13e-06 ***
---
Signif. codes:  0 '***' 0.001 '**' 0.01 '*' 0.05 '.' 0.1 ' ' 1
```

Model residuals

Statistic	Value
Sample skewness	-0.9615
Sample excess kurtosis	1.729
Passed Shapiro Wilk normality test	Yes (p-value = 0.08814 > 0.05)

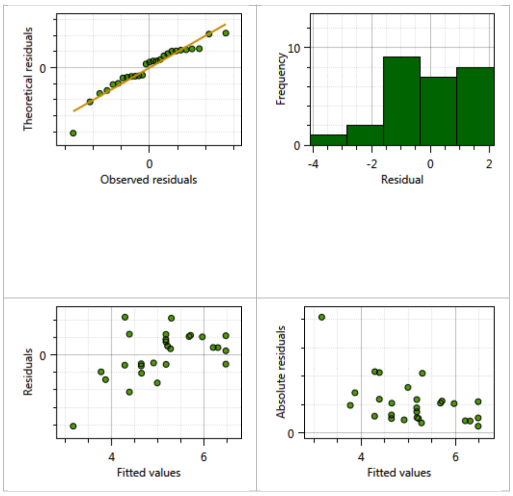

Analysis estimated duration halting (H7 - Zone 2)

**Analysis model**	Linear mixed model fit by REML: Estimated_duration_halting_H7_Zone_2 ~ 1 + (1|Genotype_Zone_1:Plant_Zone_1) + (1|Genotype_Zone_2:Plant_Zone_2)
**Transformation**	Natural logarithm

Confidence intervals estimated duration halting (H7 - Zone 2)

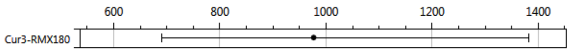

Confidence intervals estimated duration halting (H7 - Zone 2)

Genotype Zone 1	Genotype Zone 2	Mean	Lower 95% CL	Upper 95% CL	Group
Cur3	RMX180	976.6	689.8	1383	a

Model summary

```
Linear mixed model fit by REML. t-tests use Satterthwaite's method ['lmerModLmerTest']
Formula: Estimated_duration_halting_H7_Zone_2 ~ 1 + (1 | Genotype_Zone_1:Plant_Zone_1) +      (1 |
Genotype_Zone_2:Plant_Zone_2)
   Data: data

REML criterion at convergence: 116.8

Scaled residuals:
    Min      1Q  Median      3Q     Max
-2.3522 -0.8055  0.4677  0.7073  1.1876

Random effects:
 Groups                       Name        Variance Std.Dev.
 Genotype_Zone_1:Plant_Zone_1 (Intercept) 0.00     0.000
 Genotype_Zone_2:Plant_Zone_2 (Intercept) 0.00     0.000
 Residual                                 1.15     1.072
Number of obs: 39, groups:  Genotype_Zone_1:Plant_Zone_1, 10; Genotype_Zone_2:Plant_Zone_2, 10

Fixed effects:
            Estimate Std. Error      df t value Pr(>|t|)
(Intercept)   6.8841     0.1717 38.0000   40.09   <2e-16 ***
---
Signif. codes:  0 '***' 0.001 '**' 0.01 '*' 0.05 '.' 0.1 ' ' 1
```

Model residuals

Statistic	Value
Sample skewness	-0.818
Sample excess kurtosis	-0.581
Passed Shapiro Wilk normality test	No (p-value = 0.000724 < 0.05)

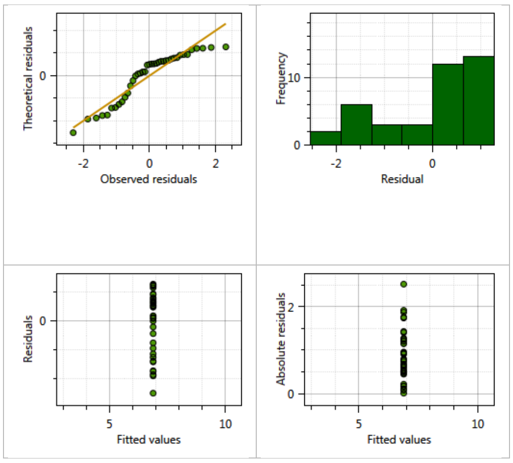

Analysis estimated duration halting H0 (diff. Zone 1 - Zone 2)

**Analysis model**	Generalized linear mixed model with dispersion factor, formula=cbind(Estimated_duration_halting_H0_Zone_1,Estimated_duration_halting_H0_Zone_2) ~ 1 + (1|Genotype_Zone_1:Plant_Zone_1) + (1|Genotype_Zone_2:Plant_Zone_2)
**Transformation**	Logit

Confidence intervals estimated duration halting H0 (diff. Zone 1 - Zone 2)

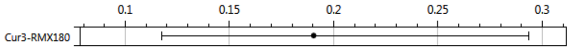

Confidence intervals estimated duration halting H0 (diff. Zone 1 - Zone 2)

Genotype Zone 1	Genotype Zone 2	Mean	Lower 95% CL	Upper 95% CL	Group
Cur3	RMX180	0.1904	0.1173	0.2937	a

Model summary

```
Linear mixed model fit by REML. t-tests use Satterthwaite's method ['lmerModLmerTest']
Formula: ziFormula
   Data: data
Weights: wi

REML criterion at convergence: 158.8

Scaled residuals: 
    Min      1Q  Median      3Q     Max 
-1.0772 -0.6598 -0.1135  0.5231  2.8293 

Random effects:
 Groups                        Name        Variance Std.Dev.
 Genotype_Zone_1:Plant_Zone_1 (Intercept)   0.0     0.00   
 Genotype_Zone_2:Plant_Zone_2 (Intercept)   0.0     0.00   
 Residual                                 729.4    27.01   
Number of obs: 39, groups:  Genotype_Zone_1:Plant_Zone_1, 10; Genotype_Zone_2:Plant_Zone_2, 10

Fixed effects:
            Estimate Std. Error      df t value Pr(>|t|)    
(Intercept)  -1.4477     0.2817 38.0000   -5.14 8.57e-06 ***
---
Signif. codes:  0 '***' 0.001 '**' 0.01 '*' 0.05 '.' 0.1 ' ' 1

Dispersion: 27.01
```

Model residuals

Statistic	Value
Sample skewness	1.209
Sample excess kurtosis	0.8446
Passed Shapiro Wilk normality test	No (p-value = 0.0005149 < 0.05)

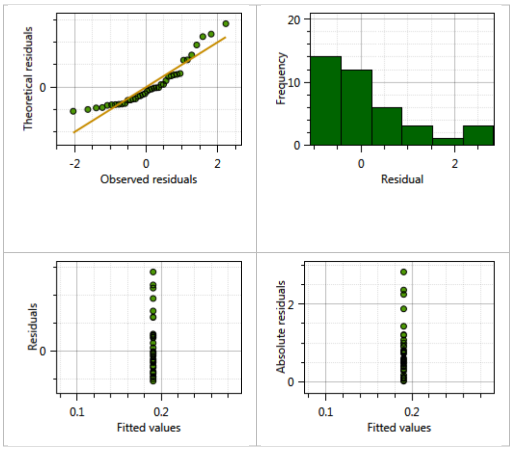

Analysis estimated duration halting H1 (diff. Zone 1 - Zone 2)

**Analysis model**	Generalized linear mixed model with dispersion factor, formula=cbind(Estimated_duration_halting_H1_Zone_1,Estimated_duration_halting_H1_Zone_2) ~ 1 + (1|Genotype_Zone_1:Plant_Zone_1) + (1|Genotype_Zone_2:Plant_Zone_2)
**Transformation**	Logit

Confidence intervals estimated duration halting H1 (diff. Zone 1 - Zone 2)

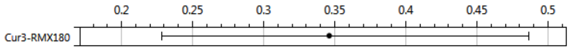

Confidence intervals estimated duration halting H1 (diff. Zone 1 - Zone 2)

Genotype Zone 1	Genotype Zone 2	Mean	Lower 95% CL	Upper 95% CL	Group
Cur3	RMX180	0.3461	0.228	0.4866	a

Model summary

```
Linear mixed model fit by REML. t-tests use Satterthwaite's method ['lmerModLmerTest']
Formula: ziFormula
   Data: data
```

```
Weights: wi

REML criterion at convergence: 149.9

Scaled residuals:
    Min      1Q  Median      3Q     Max
-1.4740 -0.7853 -0.2031  0.6852  2.7470

Random effects:
 Groups                      Name        Variance  Std.Dev.
 Genotype_Zone_1:Plant_Zone_1 (Intercept)   0.03403  0.1845
 Genotype_Zone_2:Plant_Zone_2 (Intercept)   0.00000  0.0000
 Residual                               833.80678 28.8757
Number of obs: 39, groups:  Genotype_Zone_1:Plant_Zone_1, 10; Genotype_Zone_2:Plant_Zone_2, 10

Fixed effects:
            Estimate Std. Error      df t value Pr(>|t|)
(Intercept)  -0.6364     0.2595  9.4384  -2.452   0.0355 *
---
Signif. codes:  0 '***' 0.001 '**' 0.01 '*' 0.05 '.' 0.1 ' ' 1

Dispersion: 28.88
```

Model residuals

Statistic	Value
Sample skewness	0.8246
Sample excess kurtosis	0.2167
Passed Shapiro Wilk normality test	No (p-value = 0.02922 < 0.05)

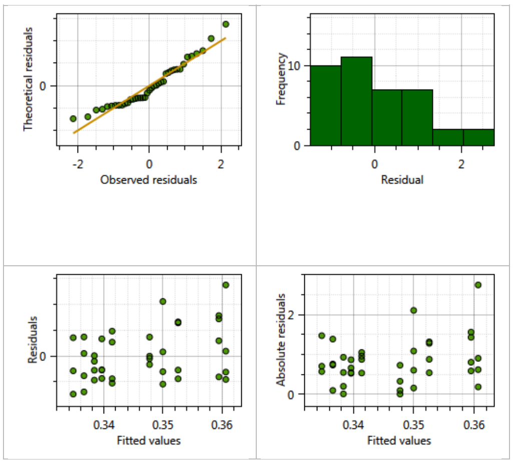

Analysis estimated duration halting H2 (diff. Zone 1 - Zone 2)

Analysis model	Generalized linear mixed model with dispersion factor, formula=cbind(Estimated_duration_halting_H2_Zone_1,Estimated_duration_halting_H2_Zone_2) ~ 1 + (1|Genotype_Zone_1:Plant_Zone_1) + (1|Genotype_Zone_2:Plant_Zone_2)
Transformation	Logit

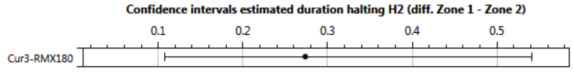

Confidence intervals estimated duration halting H2 (diff. Zone 1 - Zone 2)

Genotype Zone 1	Genotype Zone 2	Mean	Lower 95% CL	Upper 95% CL	Group
Cur3	RMX180	0.2736	0.1072	0.5415	a

Model summary

```
Linear mixed model fit by REML. t-tests use Satterthwaite's method ['lmerModLmerTest']
Formula: ziFormula
   Data: data
Weights: wi

REML criterion at convergence: 164.3

Scaled residuals:
    Min      1Q  Median      3Q     Max
-1.5599 -0.6154 -0.3300  0.5153  1.8816

Random effects:
 Groups                      Name        Variance Std.Dev.
 Genotype_Zone_1:Plant_Zone_1 (Intercept)   1.1710  1.0821
 Genotype_Zone_2:Plant_Zone_2 (Intercept)   0.4454  0.6674
 Residual                               648.1894 25.4596
Number of obs: 39, groups:  Genotype_Zone_1:Plant_Zone_1, 10; Genotype_Zone_2:Plant_Zone_2, 10

Fixed effects:
            Estimate Std. Error      df t value Pr(>|t|)
(Intercept)  -0.9766     0.4738  6.3809  -2.061   0.0822 .
---
Signif. codes:  0 '***' 0.001 '**' 0.01 '*' 0.05 '.' 0.1 ' ' 1

Dispersion: 25.46
```

Model residuals

Statistic	Value
Sample skewness	0.5465
Sample excess kurtosis	-0.4083
Passed Shapiro Wilk normality test	Yes (p-value = 0.0574 > 0.05)

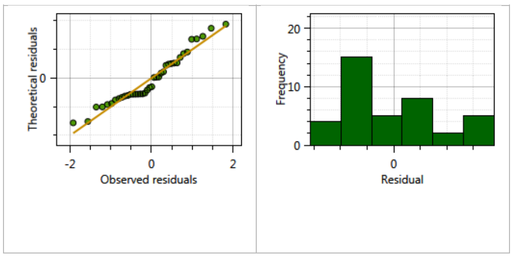

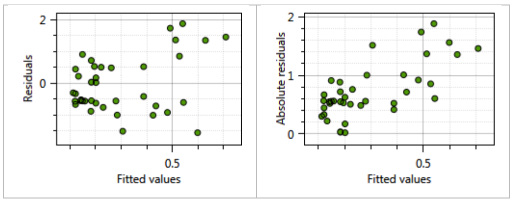

Analysis estimated duration halting H3 (diff. Zone 1 - Zone 2)

**Analysis model**	Generalized linear mixed model with dispersion factor, formula=cbind(Estimated_duration_halting_H3_Zone_1,Estimated_duration_halting_H3_Zone_2) ~ 1 + (1|Genotype_Zone_1:Plant_Zone_1) + (1|Genotype_Zone_2:Plant_Zone_2)
**Transformation**	Logit

Confidence intervals estimated duration halting H3 (diff. Zone 1 - Zone 2)

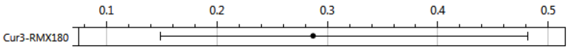

Confidence intervals estimated duration halting H3 (diff. Zone 1 - Zone 2)

Genotype Zone 1	Genotype Zone 2	Mean	Lower 95% CL	Upper 95% CL	Group
Cur3	RMX180	0.287	0.1484	0.4817	a

Model summary

```
Linear mixed model fit by REML. t-tests use Satterthwaite's method ['lmerModLmerTest']
Formula: ziFormula
   Data: data
Weights: wi

REML criterion at convergence: 153.1

Scaled residuals:
    Min      1Q  Median      3Q     Max
-1.4260 -0.7947 -0.1628  0.8222  1.8337

Random effects:
 Groups                      Name        Variance Std.Dev.
 Genotype_Zone_1:Plant_Zone_1 (Intercept)   0.0000  0.0000
 Genotype_Zone_2:Plant_Zone_2 (Intercept)   0.8052  0.8973
 Residual                               662.1146 25.7316
Number of obs: 39, groups:  Genotype_Zone_1:Plant_Zone_1, 10; Genotype_Zone_2:Plant_Zone_2, 10

Fixed effects:
            Estimate Std. Error      df t value Pr(>|t|)
(Intercept)  -0.9101     0.3693  8.9025  -2.465   0.0362 *
---
Signif. codes:  0 '***' 0.001 '**' 0.01 '*' 0.05 '.' 0.1 ' ' 1

Dispersion: 25.73
```

Model residuals

Statistic	Value
Sample skewness	0.3591
Sample excess kurtosis	-1.134
Passed Shapiro Wilk normality test	No (p-value = 0.02699 < 0.05)

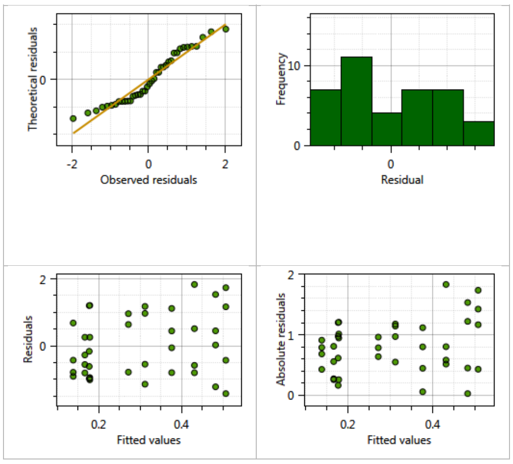

Analysis estimated duration halting H4 (diff. Zone 1 - Zone 2)

**Analysis model**	Generalized linear mixed model with dispersion factor, formula=cbind(Estimated_duration_halting_H4_Zone_1,Estimated_duration_halting_H4_Zone_2) ~ 1 + (1|Genotype_Zone_1:Plant_Zone_1) + (1|Genotype_Zone_2:Plant_Zone_2)
**Transformation**	Logit

Confidence intervals estimated duration halting H4 (diff. Zone 1 - Zone 2)

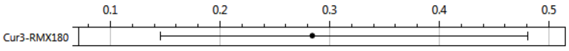

Confidence intervals estimated duration halting H4 (diff. Zone 1 - Zone 2)

Genotype Zone 1	Genotype Zone 2	Mean	Lower 95% CL	Upper 95% CL	Group
Cur3	RMX180	0.2841	0.1453	0.4809	a

Model summary

```
Linear mixed model fit by REML. t-tests use Satterthwaite's method ['lmerModLmerTest']
Formula: ziFormula
   Data: data
Weights: wi

REML criterion at convergence: 156

Scaled residuals:
    Min      1Q  Median      3Q     Max
-0.9973 -0.7234 -0.4313  0.6547  2.2899

Random effects:
 Groups                      Name        Variance  Std.Dev.
 Genotype_Zone_1:Plant_Zone_1 (Intercept) 8.926e-02  0.2988
 Genotype_Zone_2:Plant_Zone_2 (Intercept) 3.435e-01  0.5861
 Residual                               1.011e+03 31.7903
Number of obs: 39, groups:  Genotype_Zone_1:Plant_Zone_1, 10; Genotype_Zone_2:Plant_Zone_2, 10

Fixed effects:
            Estimate Std. Error      df t value Pr(>|t|)
```

```
(Intercept)  -0.9242     0.3336  5.2028   -2.77   0.0377 *
---
Signif. codes:  0 '***' 0.001 '**' 0.01 '*' 0.05 '.' 0.1 ' ' 1

Dispersion: 31.79
```

Model residuals

Statistic	Value
Sample skewness	1.14
Sample excess kurtosis	0.1232
Passed Shapiro Wilk normality test	No (p-value = 3.03E-05 < 0.05)

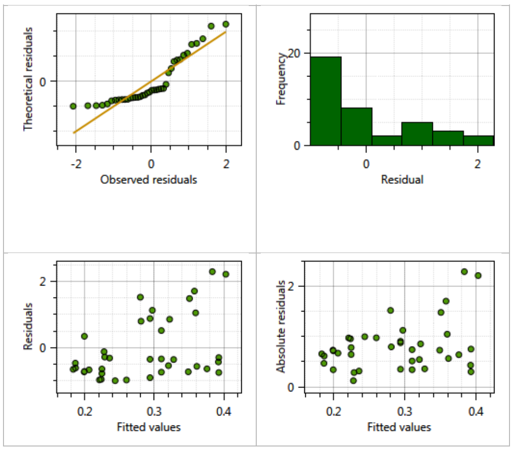

Analysis estimated duration halting H5 (diff. Zone 1 - Zone 2)

**Analysis model**	Generalized linear mixed model with dispersion factor, formula=cbind(Estimated_duration_halting_H5_Zone_1,Estimated_duration_halting_H5_Zone_2) ~ 1 + (1|Genotype_Zone_1:Plant_Zone_1) + (1|Genotype_Zone_2:Plant_Zone_2)
**Transformation**	Logit

Confidence intervals estimated duration halting H5 (diff. Zone 1 - Zone 2)

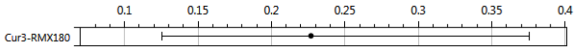

Confidence intervals estimated duration halting H5 (diff. Zone 1 - Zone 2)

Genotype Zone 1	Genotype Zone 2	Mean	Lower 95% CL	Upper 95% CL	Group
Cur3	RMX180	0.2269	0.1252	0.3757	a

Model summary

```
Linear mixed model fit by REML. t-tests use Satterthwaite's method ['lmerModLmerTest']
Formula: ziFormula
   Data: data
Weights: wi

REML criterion at convergence: 159.6

Scaled residuals:
    Min      1Q  Median      3Q     Max
-0.9471 -0.7035 -0.3962  0.7043  2.7715

Random effects:
 Groups                      Name        Variance  Std.Dev.
 Genotype_Zone_1:Plant_Zone_1 (Intercept) 6.783e-02  0.26044
 Genotype_Zone_2:Plant_Zone_2 (Intercept) 7.995e-03  0.08941
 Residual                                1.077e+03 32.82052
Number of obs: 39, groups:  Genotype_Zone_1:Plant_Zone_1, 10; Genotype_Zone_2:Plant_Zone_2, 10

Fixed effects:
            Estimate Std. Error      df t value Pr(>|t|)
(Intercept)  -1.2262     0.2993  6.5340  -4.096  0.00532 **
---
Signif. codes:  0 '***' 0.001 '**' 0.01 '*' 0.05 '.' 0.1 ' ' 1

Dispersion: 32.82
```

Model residuals

Statistic	Value
Sample skewness	1.097
Sample excess kurtosis	0.3347
Passed Shapiro Wilk normality test	No (p-value = 0.0001362 < 0.05)

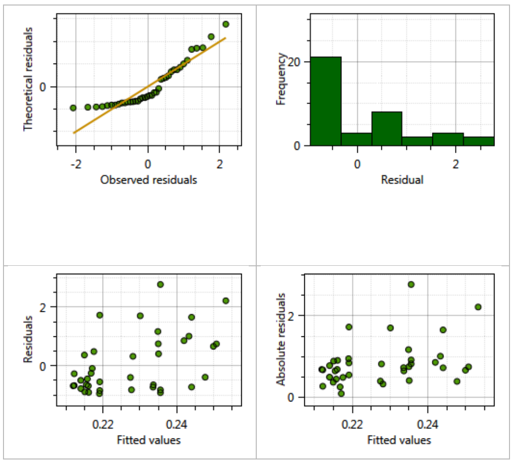

Analysis estimated duration halting H6 (diff. Zone 1 - Zone 2)

**Analysis model**	Generalized linear mixed model with dispersion factor, formula=cbind(Estimated_duration_halting_H6_Zone_1,Estimated_duration_halting_H6_Zone_2) ~ 1 + (1|Genotype_Zone_1:Plant_Zone_1) + (1|Genotype_Zone_2:Plant_Zone_2)
**Transformation**	Logit

Confidence intervals estimated duration halting H6 (diff. Zone 1 - Zone 2)

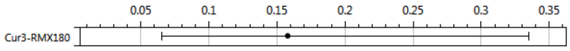

Confidence intervals estimated duration halting H6 (diff. Zone 1 - Zone 2)

Genotype Zone 1	Genotype Zone 2	Mean	Lower 95% CL	Upper 95% CL	Group
Cur3	RMX180	0.1578	0.06508	0.3352	a

Model summary

```
Linear mixed model fit by REML. t-tests use Satterthwaite's method ['lmerModLmerTest']
Formula: ziFormula
   Data: data
Weights: wi

REML criterion at convergence: 167.9

Scaled residuals:
    Min      1Q  Median      3Q     Max
-1.3164 -0.6540 -0.2286  0.2614  2.4858

Random effects:
 Groups                      Name        Variance Std.Dev.
 Genotype_Zone_1:Plant_Zone_1 (Intercept)   0.3311  0.5754
 Genotype_Zone_2:Plant_Zone_2 (Intercept)   0.7297  0.8542
 Residual                               746.2009 27.3167
Number of obs: 39, groups:  Genotype_Zone_1:Plant_Zone_1, 10; Genotype_Zone_2:Plant_Zone_2, 10

Fixed effects:
            Estimate Std. Error      df t value Pr(>|t|)
(Intercept)  -1.6747     0.4333  8.4446  -3.865  0.00431 **
---
Signif. codes:  0 '***' 0.001 '**' 0.01 '*' 0.05 '.' 0.1 ' ' 1

Dispersion: 27.32
```

Model residuals

Statistic	Value
Sample skewness	1.11
Sample excess kurtosis	0.711
Passed Shapiro Wilk normality test	No (p-value = 0.001946 < 0.05)

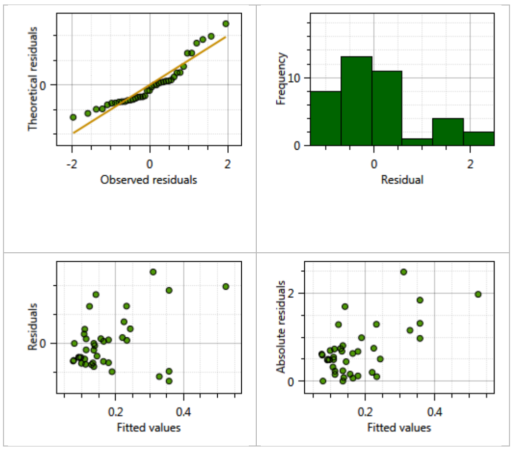

Analysis estimated duration halting H7 (diff. Zone 1 - Zone 2)

**Analysis model**	Generalized linear mixed model with dispersion factor, formula=cbind(Estimated_duration_halting_H7_Zone_1,Estimated_duration_halting_H7_Zone_2) ~ 1 + (1|Genotype_Zone_1:Plant_Zone_1) + (1|Genotype_Zone_2:Plant_Zone_2)
**Transformation**	Logit

Confidence intervals estimated duration halting H7 (diff. Zone 1 - Zone 2)

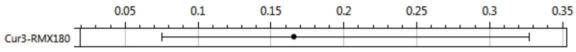

Confidence intervals estimated duration halting H7 (diff. Zone 1 - Zone 2)

Genotype Zone 1	Genotype Zone 2	Mean	Lower 95% CL	Upper 95% CL	Group
Cur3	RMX180	0.1657	0.07496	0.3273	a

Model summary

```
Linear mixed model fit by REML. t-tests use Satterthwaite's method ['lmerModLmerTest']
Formula: ziFormula
   Data: data
Weights: wi

REML criterion at convergence: 166.2

Scaled residuals:
    Min      1Q  Median      3Q     Max
-1.1560 -0.6318 -0.4023  0.7911  2.0564

Random effects:
 Groups                      Name        Variance  Std.Dev.
 Genotype_Zone_1:Plant_Zone_1 (Intercept)   0.06106  0.2471
 Genotype_Zone_2:Plant_Zone_2 (Intercept)   0.65019  0.8063
 Residual                               715.10909 26.7415
Number of obs: 39, groups:  Genotype_Zone_1:Plant_Zone_1, 10; Genotype_Zone_2:Plant_Zone_2, 10

Fixed effects:
            Estimate Std. Error      df t value Pr(>|t|)
(Intercept)  -1.6166     0.3889  8.0251  -4.157  0.00316 **
---
Signif. codes:  0 '***' 0.001 '**' 0.01 '*' 0.05 '.' 0.1 ' ' 1

Dispersion: 26.74
```

Model residuals

Statistic	Value
Sample skewness	0.8325
Sample excess kurtosis	-0.7594
Passed Shapiro Wilk normality test	No (p-value = 9.521E-05 < 0.05)

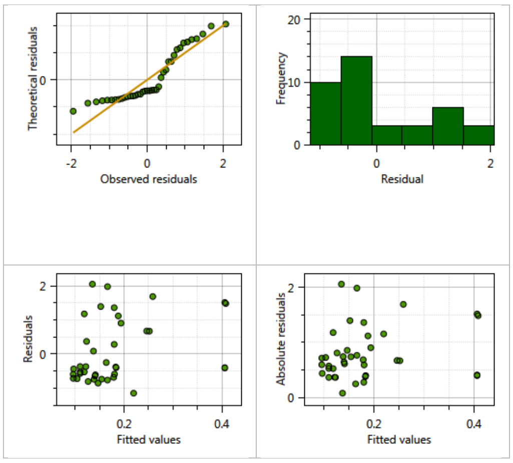

Estimated duration halting per zone per short/medium/long

Selected zones	Zone 1, Zone 2
Event duration categories	duration < 2, 2 <= duration < 10, duration >= 10
Data transformation	Natural logarithm
Analysis	Zone difference analysis

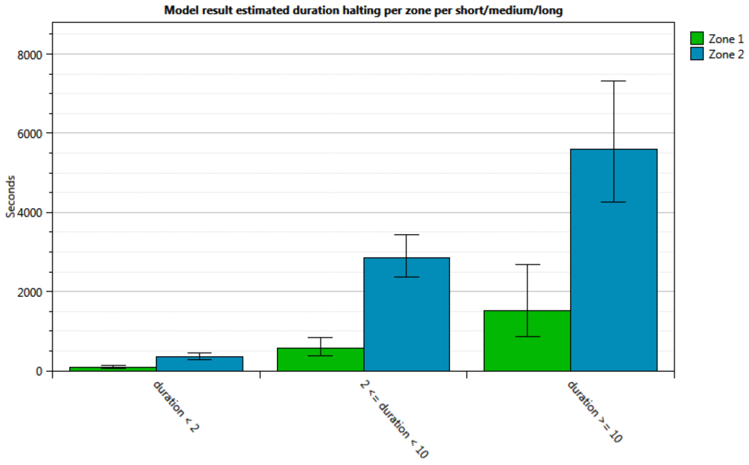

Results difference tests Zone 1 - Zone 2: p values and 95% confidence intervals of the difference on the transformed scale for each statistic.

Behaviour statistic	Cur3-RMX180
Estimated duration halting duration < 2 (diff. Zone 1 - Zone 2)	p=1.25E-05****
	[-1.89, -1.08]
Estimated duration halting 2 <= duration < 10 (diff. Zone 1 - Zone 2)	p=2.7E-05****
	[-1.89, -1.05]
Estimated duration halting duration >= 10 (diff. Zone 1 - Zone 2)	p=0.0129*
	[-1.71, -0.318]

The model predictions and 95% confidence intervals for each statistic.

Statistic	Cur3-RMX180	Remark
Estimated duration halting (duration < 2 - Zone 1)	80.7	
	[53.6, 121]	
Estimated duration halting (2 <= duration < 10 - Zone 1)	567	CR
	[388, 828]	
Estimated duration halting (duration >= 10 - Zone 1)	1.53E+03	CR
	[870, 2.67E+03]	
Estimated duration halting (duration < 2 - Zone 2)	355	
	[282, 446]	
Estimated duration halting (2 <= duration < 10 - Zone 2)	2.85E+03	
	[2.36E+03, 3.44E+03]	
Estimated duration halting (duration >= 10 - Zone 2)	5.59E+03	CR
	[4.26E+03, 7.33E+03]	

CR = Check residuals

Data summary

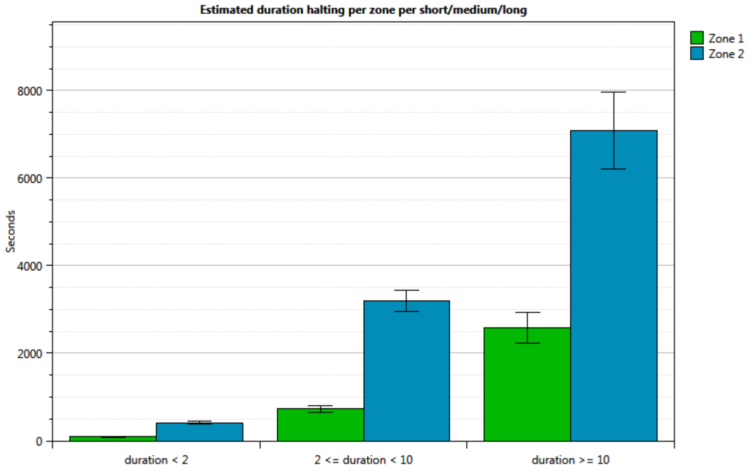

Genotype Zone 1	Genotype Zone 2	Genotype Zone 3	Mean duration < 2 - Zone 1	StdErr duration < 2 - Zone 1	Mean 2 <= duration < 10 - Zone 1	StdErr 2 <= duration < 10 - Zone 1	Mean duration >= 10 - Zone 1	StdErr duration >= 10 - Zone 1	Mean duration < 2 - Zone 2	StdErr duration < 2 - Zone 2	Mean 2 <= duration < 10 - Zone 2	StdErr 2 <= duration < 10 - Zone 2	Mean duration >= 10 - Zone 2	StdErr duration >= 10 - Zone 2
Cur3	RMX180	Neutral	92.67	9.18	734.62	77.45	2579.45	352.76	418.18	38.05	3196.86	238.46	7081.04	882.38

Analysis estimated duration halting (duration < 2 - Zone 1)

**Analysis model**	Linear mixed model fit by REML: Estimated_duration_halting_duration_2_Zone_1 ~ 1 + (1|Genotype_Zone_1:Plant_Zone_1) + (1|Genotype_Zone_2:Plant_Zone_2)
**Transformation**	Natural logarithm

Confidence intervals estimated duration halting (duration < 2 - Zone 1)

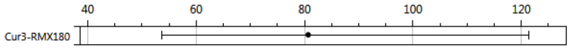

Confidence intervals estimated duration halting (duration < 2 - Zone 1)

Genotype Zone 1	Genotype Zone 2	Mean	Lower 95% CL	Upper 95% CL	Group
Cur3	RMX180	80.67	53.59	121.4	a

Model summary

```
Linear mixed model fit by REML. t-tests use Satterthwaite's method ['lmerModLmerTest']
Formula: Estimated_duration_halting_duration_2_Zone_1 ~ 1 + (1 | Genotype_Zone_1:Plant_Zone_1) +      (1
| Genotype_Zone_2:Plant_Zone_2)
   Data: data

REML criterion at convergence: 79.7

Scaled residuals: 
    Min      1Q  Median      3Q     Max 
-2.3828 -0.4325  0.1112  0.5255  1.5471 

Random effects:
 Groups                       Name        Variance Std.Dev.
 Genotype_Zone_1:Plant_Zone_1 (Intercept) 0.1196   0.3458  
 Genotype_Zone_2:Plant_Zone_2 (Intercept) 0.1088   0.3298  
 Residual                                 0.3307   0.5751  
Number of obs: 37, groups:  Genotype_Zone_1:Plant_Zone_1, 10; Genotype_Zone_2:Plant_Zone_2, 10

Fixed effects:
            Estimate Std. Error     df t value Pr(>|t|)    
(Intercept)   4.3903     0.1792 8.5016    24.5 3.48e-09 ***
---
Signif. codes:  0 '***' 0.001 '**' 0.01 '*' 0.05 '.' 0.1 ' ' 1
```

Model residuals

Statistic	Value
Sample skewness	-0.6164
Sample excess kurtosis	0.5363
Passed Shapiro Wilk normality test	Yes (p-value = 0.4762 > 0.05)

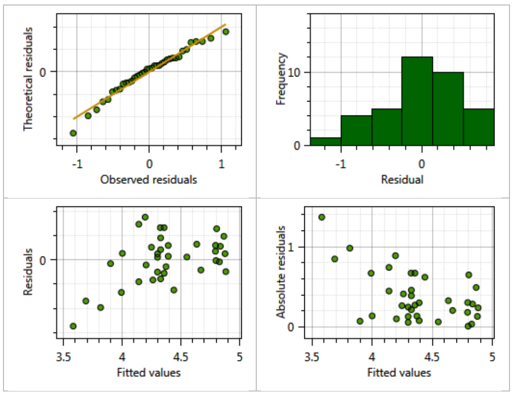

Analysis estimated duration halting (2 <= duration < 10 - Zone 1)

**Analysis model**	Linear mixed model fit by REML: Estimated_duration_halting_2_duration_10_Zone_1 ~ 1 + (1|Genotype_Zone_1:Plant_Zone_1) + (1|Genotype_Zone_2:Plant_Zone_2)
**Transformation**	Natural logarithm

Confidence intervals estimated duration halting (2 <= duration < 10 - Zone 1)

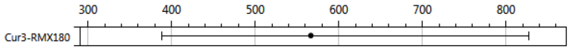

Confidence intervals estimated duration halting (2 <= duration < 10 - Zone 1)

Genotype Zone 1	Genotype Zone 2	Mean	Lower 95% CL	Upper 95% CL	Group
Cur3	RMX180	566.5	387.8	827.6	a

Model summary

```
Linear mixed model fit by REML. t-tests use Satterthwaite's method ['lmerModLmerTest']
Formula: Estimated_duration_halting_2_duration_10_Zone_1 ~ 1 + (1 | Genotype_Zone_1:Plant_Zone_1) +  
(1 | Genotype_Zone_2:Plant_Zone_2)
   Data: data

REML criterion at convergence: 109.9

Scaled residuals: 
    Min      1Q  Median      3Q     Max 
-4.6026 -0.1962  0.1202  0.5080  1.2140 

Random effects:
 Groups                       Name        Variance Std.Dev.
 Genotype_Zone_1:Plant_Zone_1 (Intercept) 0.00000  0.000   
 Genotype_Zone_2:Plant_Zone_2 (Intercept) 0.01716  0.131   
 Residual                                 1.01804  1.009   
Number of obs: 38, groups:  Genotype_Zone_1:Plant_Zone_1, 10; Genotype_Zone_2:Plant_Zone_2, 10

Fixed effects:
            Estimate Std. Error     df t value Pr(>|t|)    
(Intercept)   6.3395     0.1689 9.4864   37.54 1.22e-11 ***
---
Signif. codes:  0 '***' 0.001 '**' 0.01 '*' 0.05 '.' 0.1 ' ' 1
```

Model residuals

Statistic	Value
Sample skewness	-2.857
Sample excess kurtosis	12.04
Passed Shapiro Wilk normality test	No (p-value = 1.153E-06 < 0.05)

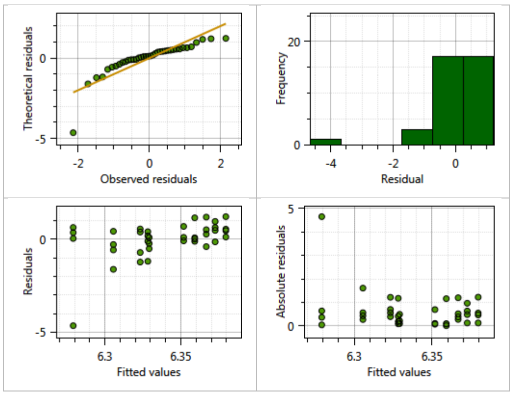

Analysis estimated duration halting (duration >= 10 - Zone 1)

**Analysis model**	Linear mixed model fit by REML: Estimated_duration_halting_duration_10_Zone_1 ~ 1 + (1|Genotype_Zone_1:Plant_Zone_1) + (1|Genotype_Zone_2:Plant_Zone_2)
**Transformation**	Natural logarithm

Confidence intervals estimated duration halting (duration >= 10 - Zone 1)

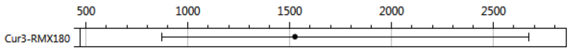

Confidence intervals estimated duration halting (duration >= 10 - Zone 1)

Genotype Zone 1	Genotype Zone 2	Mean	Lower 95% CL	Upper 95% CL	Group
Cur3	RMX180	1525	870.1	2674	a

Model summary

```
Linear mixed model fit by REML. t-tests use Satterthwaite's method ['lmerModLmerTest']
Formula: Estimated_duration_halting_duration_10_Zone_1 ~ 1 + (1 | Genotype_Zone_1:Plant_Zone_1) +      (1
| Genotype_Zone_2:Plant_Zone_2)
   Data: data

REML criterion at convergence: 129.4

Scaled residuals:
    Min      1Q  Median      3Q     Max
-3.0143 -0.1818  0.1411  0.6014  1.4937

Random effects:
 Groups                       Name        Variance  Std.Dev.
 Genotype_Zone_1:Plant_Zone_1 (Intercept) 2.486e-15 4.986e-08
 Genotype_Zone_2:Plant_Zone_2 (Intercept) 2.568e-01 5.068e-01
 Residual                                 1.410e+00 1.187e+00
Number of obs: 39, groups:  Genotype_Zone_1:Plant_Zone_1, 10; Genotype_Zone_2:Plant_Zone_2, 10

Fixed effects:
            Estimate Std. Error     df t value Pr(>|t|)
(Intercept)   7.3300     0.2489 9.1714   29.45 2.13e-10 ***
---
Signif. codes:  0 '***' 0.001 '**' 0.01 '*' 0.05 '.' 0.1 ' ' 1
```

Model residuals

Statistic	Value
Sample skewness	-1.364
Sample excess kurtosis	2.21
Passed Shapiro Wilk normality test	No (p-value = 0.001378 < 0.05)

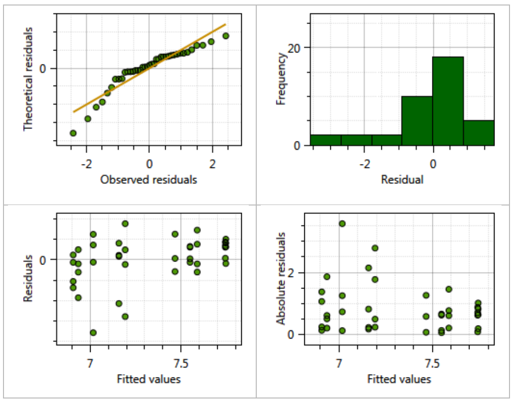

Analysis estimated duration halting (duration < 2 - Zone 2)

**Analysis model**	Linear mixed model fit by REML: Estimated_duration_halting_duration_2_Zone_2 ~ 1 + (1|Genotype_Zone_1:Plant_Zone_1) + (1|Genotype_Zone_2:Plant_Zone_2)
**Transformation**	Natural logarithm

Confidence intervals estimated duration halting (duration < 2 - Zone 2)

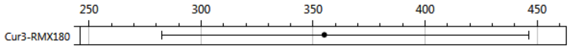

Confidence intervals estimated duration halting (duration < 2 - Zone 2)

Genotype Zone 1	Genotype Zone 2	Mean	Lower 95% CL	Upper 95% CL	Group
Cur3	RMX180	355	282.3	446.4	a

Model summary

```
Linear mixed model fit by REML. t-tests use Satterthwaite's method ['lmerModLmerTest']
Formula: Estimated_duration_halting_duration_2_Zone_2 ~ 1 + (1 | Genotype_Zone_1:Plant_Zone_1) +      (1
| Genotype_Zone_2:Plant_Zone_2)
   Data: data

REML criterion at convergence: 72.1

Scaled residuals:
     Min       1Q   Median       3Q      Max
-2.65091 -0.68737 -0.09242  0.82437  1.65288

Random effects:
 Groups                       Name        Variance Std.Dev.
 Genotype_Zone_1:Plant_Zone_1 (Intercept) 0.00000  0.0000
 Genotype_Zone_2:Plant_Zone_2 (Intercept) 0.01092  0.1045
```

```
 Residual                                0.34489  0.5873  
Number of obs: 39, groups:  Genotype_Zone_1:Plant_Zone_1, 10; Genotype_Zone_2:Plant_Zone_2, 10

Fixed effects:
            Estimate Std. Error      df t value Pr(>|t|)    
(Intercept)  5.87207    0.09971 8.17590   58.89 4.87e-12 ***
---
Signif. codes:  0 '***' 0.001 '**' 0.01 '*' 0.05 '.' 0.1 ' ' 1
```

Model residuals

Statistic	Value
Sample skewness	-0.3113
Sample excess kurtosis	-0.08991
Passed Shapiro Wilk normality test	Yes (p-value = 0.3061 > 0.05)

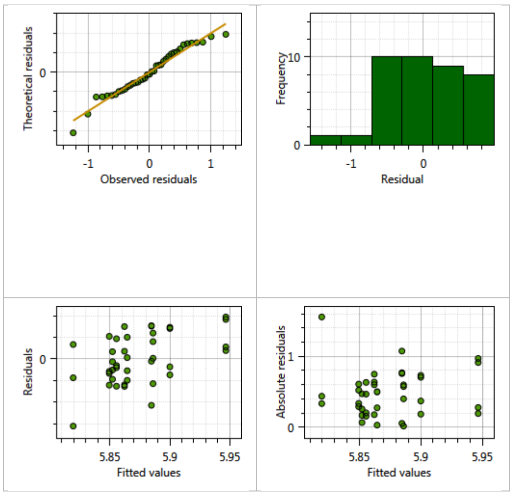

Analysis estimated duration halting (2 <= duration < 10 - Zone 2)

**Analysis model**	Linear mixed model fit by REML: Estimated_duration_halting_2_duration_10_Zone_2 ~ 1 + (1|Genotype_Zone_1:Plant_Zone_1) + (1|Genotype_Zone_2:Plant_Zone_2)
**Transformation**	Natural logarithm

Confidence intervals estimated duration halting (2 <= duration < 10 - Zone 2)

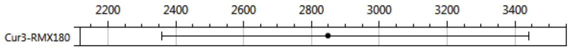

Confidence intervals estimated duration halting (2 <= duration < 10 - Zone 2)

Genotype Zone 1	Genotype Zone 2	Mean	Lower 95% CL	Upper 95% CL	Group
Cur3	RMX180	2849	2358	3442	a

Model summary

```
Linear mixed model fit by REML. t-tests use Satterthwaite's method ['lmerModLmerTest']
Formula: Estimated_duration_halting_2_duration_10_Zone_2 ~ 1 + (1 | Genotype_Zone_1:Plant_Zone_1) + 
(1 | Genotype_Zone_2:Plant_Zone_2)
   Data: data

REML criterion at convergence: 60.2

Scaled residuals: 
     Min       1Q   Median       3Q      Max 
-2.54816 -0.59345  0.06214  0.72494  1.87553 

Random effects:
 Groups                       Name        Variance Std.Dev.
 Genotype_Zone_1:Plant_Zone_1 (Intercept) 0.000000 0.0000  
 Genotype_Zone_2:Plant_Zone_2 (Intercept) 0.001452 0.0381  
 Residual                                 0.257870 0.5078  
Number of obs: 39, groups:  Genotype_Zone_1:Plant_Zone_1, 10; Genotype_Zone_2:Plant_Zone_2, 10

Fixed effects:
            Estimate Std. Error      df t value Pr(>|t|)    
(Intercept)  7.95457    0.08221 8.10103   96.76 1.06e-13 ***
---
Signif. codes:  0 '***' 0.001 '**' 0.01 '*' 0.05 '.' 0.1 ' ' 1
```

Model residuals

Statistic	Value
Sample skewness	-0.5884
Sample excess kurtosis	0.3941
Passed Shapiro Wilk normality test	Yes (p-value = 0.3285 > 0.05)

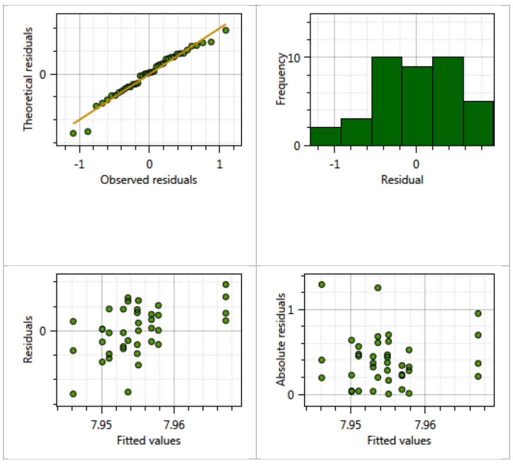

Analysis estimated duration halting (duration >= 10 - Zone 2)

**Analysis model**	Linear mixed model fit by REML: Estimated_duration_halting_duration_10_Zone_2 ~ 1 + (1|Genotype_Zone_1:Plant_Zone_1) + (1|Genotype_Zone_2:Plant_Zone_2)
**Transformation**	Natural logarithm

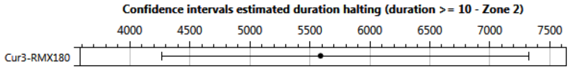

Confidence intervals estimated duration halting (duration >= 10 - Zone 2)

Genotype Zone 1	Genotype Zone 2	Mean	Lower 95% CL	Upper 95% CL	Group
Cur3	RMX180	5589	4263	7327	a

Model summary

```
Linear mixed model fit by REML. t-tests use Satterthwaite's method ['lmerModLmerTest']
Formula: Estimated_duration_halting_duration_10_Zone_2 ~ 1 + (1 | Genotype_Zone_1:Plant_Zone_1) +      (1
| Genotype_Zone_2:Plant_Zone_2)
   Data: data

REML criterion at convergence: 85.6

Scaled residuals: 
     Min       1Q   Median       3Q      Max 
-3.10500 -0.32916  0.04443  0.43979  2.05833 

Random effects:
 Groups                       Name        Variance Std.Dev.
 Genotype_Zone_1:Plant_Zone_1 (Intercept) 0.0131   0.1144  
 Genotype_Zone_2:Plant_Zone_2 (Intercept) 0.0000   0.0000  
 Residual                                 0.4936   0.7025  
Number of obs: 39, groups:  Genotype_Zone_1:Plant_Zone_1, 10; Genotype_Zone_2:Plant_Zone_2, 10

Fixed effects:
            Estimate Std. Error     df t value Pr(>|t|)    
(Intercept)   8.6285     0.1182 8.3238      73 5.58e-13 ***
---
Signif. codes:  0 '***' 0.001 '**' 0.01 '*' 0.05 '.' 0.1 ' ' 1
```

Model residuals

Statistic	Value
Sample skewness	-0.3541
Sample excess kurtosis	1.864
Passed Shapiro Wilk normality test	No (p-value = 0.04264 < 0.05)

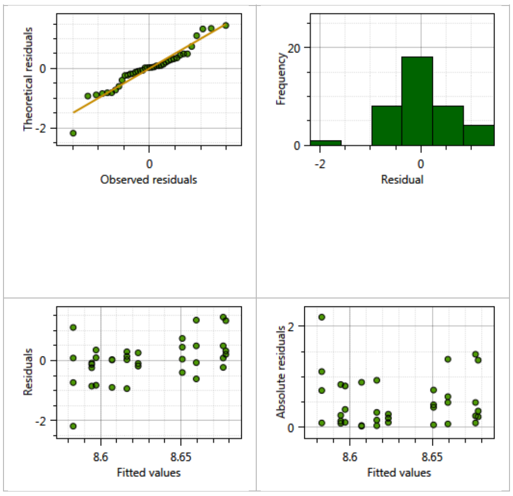

Analysis estimated duration halting duration < 2 (diff. Zone 1 - Zone 2)

**Analysis model**	Generalized linear mixed model with dispersion factor, formula=cbind(Estimated_duration_halting_duration_2_Zone_1,Estimated_duration_halting_duration_2_Zone_2) ~ 1 + (1|Genotype_Zone_1:Plant_Zone_1) + (1|Genotype_Zone_2:Plant_Zone_2)
**Transformation**	Logit

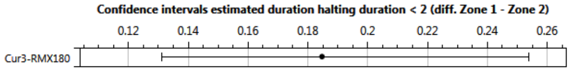

Confidence intervals estimated duration halting duration < 2 (diff. Zone 1 - Zone 2)

Genotype Zone 1	Genotype Zone 2	Mean	Lower 95% CL	Upper 95% CL	Group
Cur3	RMX180	0.1847	0.131	0.2539	a

Model summary

```
Linear mixed model fit by REML. t-tests use Satterthwaite's method ['lmerModLmerTest']
Formula: ziFormula
   Data: data
Weights: wi

REML criterion at convergence: 107.7

Scaled residuals: 
     Min       1Q   Median       3Q      Max 
-1.50771 -0.85702  0.03003  0.83140  1.86462 

Random effects:
 Groups                       Name        Variance Std.Dev.
 Genotype_Zone_1:Plant_Zone_1 (Intercept)  0.03943 0.1986  
 Genotype_Zone_2:Plant_Zone_2 (Intercept)  0.10516 0.3243  
 Residual                                 53.02243 7.2817  
Number of obs: 39, groups:  Genotype_Zone_1:Plant_Zone_1, 10; Genotype_Zone_2:Plant_Zone_2, 10

Fixed effects:
            Estimate Std. Error      df t value Pr(>|t|)    
(Intercept)  -1.4848     0.1817  9.6125  -8.172 1.25e-05 ***
---
Signif. codes:  0 '***' 0.001 '**' 0.01 '*' 0.05 '.' 0.1 ' ' 1

Dispersion: 7.282
```

Model residuals

Statistic	Value
Sample skewness	0.2564
Sample excess kurtosis	-1.135
Passed Shapiro Wilk normality test	Yes (p-value = 0.05025 > 0.05)

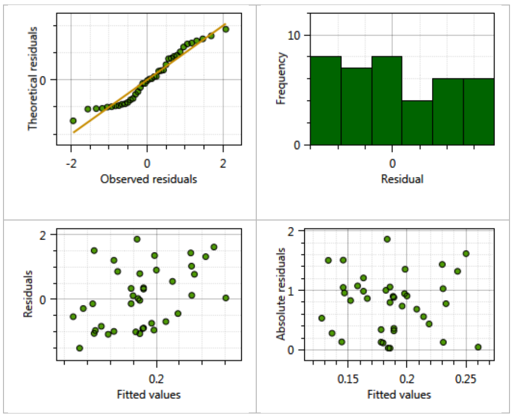

Analysis estimated duration halting 2 <= duration < 10 (diff. Zone 1 - Zone 2)

**Analysis model**	Generalized linear mixed model with dispersion factor, formula=cbind(Estimated_duration_halting_2_duration_10_Zone_1,Estimated_duration_halting_2_duration_10_Zone_2) ~ 1 + (1|Genotype_Zone_1:Plant_Zone_1) + (1|Genotype_Zone_2:Plant_Zone_2)
**Transformation**	Logit

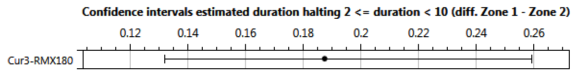

Confidence intervals estimated duration halting 2 <= duration < 10 (diff. Zone 1 - Zone 2)

Genotype Zone 1	Genotype Zone 2	Mean	Lower 95% CL	Upper 95% CL	Group
Cur3	RMX180	0.1873	0.1318	0.2593	a

Model summary

```
Linear mixed model fit by REML. t-tests use Satterthwaite's method ['lmerModLmerTest']
Formula: ziFormula
   Data: data
Weights: wi

REML criterion at convergence: 108.5

Scaled residuals:
    Min      1Q  Median      3Q     Max
-1.4935 -0.6422 -0.0164  0.7735  1.7932

Random effects:
 Groups                      Name        Variance  Std.Dev.
 Genotype_Zone_1:Plant_Zone_1 (Intercept)   0.07264  0.2695
 Genotype_Zone_2:Plant_Zone_2 (Intercept)   0.06719  0.2592
 Residual                               449.32871 21.1974
Number of obs: 39, groups:  Genotype_Zone_1:Plant_Zone_1, 10; Genotype_Zone_2:Plant_Zone_2, 10

Fixed effects:
            Estimate Std. Error      df t value Pr(>|t|)
(Intercept)  -1.4674     0.1838  8.7219  -7.984  2.7e-05 ***
---
Signif. codes:  0 '***' 0.001 '**' 0.01 '*' 0.05 '.' 0.1 ' ' 1

Dispersion: 21.2
```

Model residuals

Statistic	Value
Sample skewness	0.2664
Sample excess kurtosis	-0.8793
Passed Shapiro Wilk normality test	Yes (p-value = 0.1628 > 0.05)

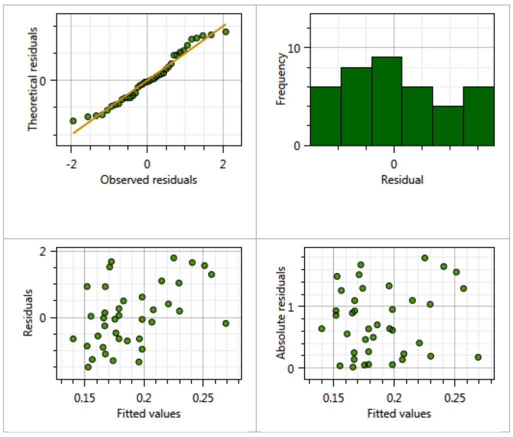

Analysis estimated duration halting duration >= 10 (diff. Zone 1 - Zone 2)

**Analysis model**	Generalized linear mixed model with dispersion factor, formula=cbind(Estimated_duration_halting_duration_10_Zone_1,Estimated_duration_halting_duration_10_Zone_2) ~ 1 + (1|Genotype_Zone_1:Plant_Zone_1) + (1|Genotype_Zone_2:Plant_Zone_2)
**Transformation**	Logit

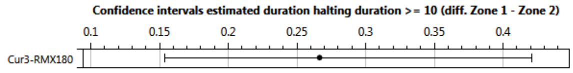

Confidence intervals estimated duration halting duration >= 10 (diff. Zone 1 - Zone 2)

Genotype Zone 1	Genotype Zone 2	Mean	Lower 95% CL	Upper 95% CL	Group
Cur3	RMX180	0.2664	0.1535	0.4211	a

Model summary

```
Linear mixed model fit by REML. t-tests use Satterthwaite's method ['lmerModLmerTest']
Formula: ziFormula
   Data: data
Weights: wi

REML criterion at convergence: 130
```

```
Scaled residuals:
     Min       1Q   Median       3Q      Max
-1.50204 -0.58073  0.01577  0.55464  2.41383

Random effects:
 Groups                       Name        Variance  Std.Dev.
 Genotype_Zone_1:Plant_Zone_1 (Intercept)    0.1537  0.3920
 Genotype_Zone_2:Plant_Zone_2 (Intercept)    0.2902  0.5387
 Residual                                 2104.1744 45.8713
Number of obs: 39, groups:  Genotype_Zone_1:Plant_Zone_1, 10; Genotype_Zone_2:Plant_Zone_2, 10

Fixed effects:
            Estimate Std. Error     df t value Pr(>|t|)
(Intercept)   -1.013      0.274  5.249  -3.697   0.0129 *
---
Signif. codes:  0 '***' 0.001 '**' 0.01 '*' 0.05 '.' 0.1 ' ' 1

Dispersion: 45.87
```

Model residuals

Statistic	Value
Sample skewness	0.3178
Sample excess kurtosis	0.0114
Passed Shapiro Wilk normality test	Yes (p-value = 0.6858 > 0.05)

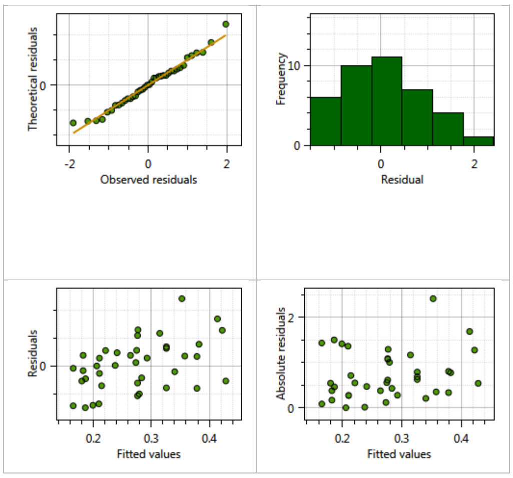

Estimated duration moving per zone

Selected zones	Zone 1, Zone 2
Data transformation	Natural logarithm
Analysis	Zone difference analysis

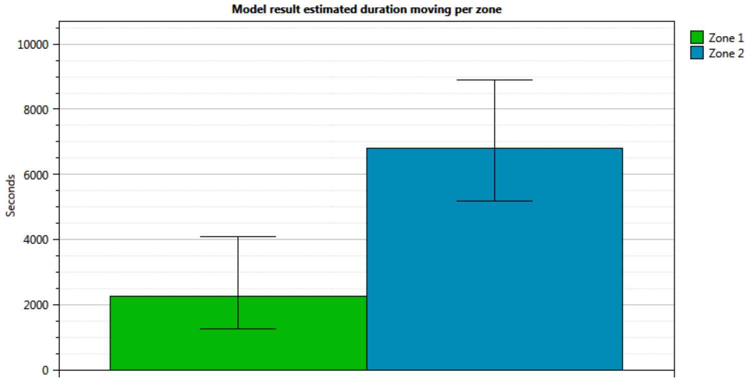

Results difference tests Zone 1 - Zone 2: p values and 95% confidence intervals of the difference on the transformed scale for each statistic.

Behaviour statistic	Cur3-RMX180	Remark
Estimated duration moving (diff. Zone 1 - Zone 2)	p=0.000403***<br>[-0.982, -0.408]	CR

CR = Check residuals

The model predictions and 95% confidence intervals for each statistic.

Statistic	Cur3-RMX180	Remark
Estimated duration moving (Zone 1)	2.27E+03<br>[1.26E+03, 4.1E+03]	CR
Estimated duration moving (Zone 2)	6.8E+03<br>[5.19E+03, 8.91E+03]	CR

CR = Check residuals

Data summary

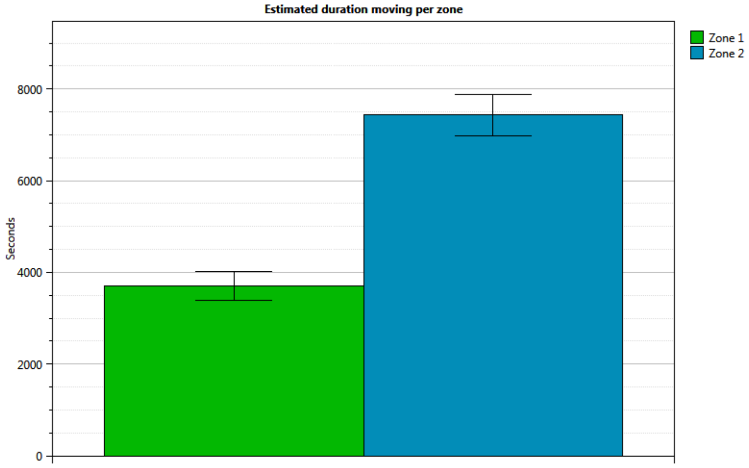

Genotype Zone 1	Genotype Zone 2	Genotype Zone 3	Mean Zone 1	StdErr Zone 1	Mean Zone 2	StdErr Zone 2
Cur3	RMX180	Neutral	3701.71	312.41	7437.47	449.79

Analysis estimated duration moving (Zone 1)

**Analysis model**	Linear mixed model fit by REML: Estimated_duration_moving_Zone_1 ~ 1 + (1|Genotype_Zone_1:Plant_Zone_1) + (1|Genotype_Zone_2:Plant_Zone_2)
**Transformation**	Natural logarithm

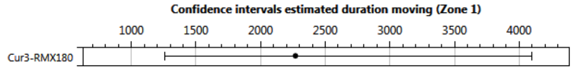

Confidence intervals estimated duration moving (Zone 1)

Genotype Zone 1	Genotype Zone 2	Mean	Lower 95% CL	Upper 95% CL	Group
Cur3	RMX180	2269	1256	4100	a

Model summary

```
Linear mixed model fit by REML. t-tests use Satterthwaite's method ['lmerModLmerTest']
Formula: Estimated_duration_moving_Zone_1 ~ 1 + (1 | Genotype_Zone_1:Plant_Zone_1) +      (1 |
Genotype_Zone_2:Plant_Zone_2)
   Data: data

REML criterion at convergence: 157.2

Scaled residuals:
    Min      1Q  Median      3Q     Max
-4.2176  0.0521  0.2792  0.4315  0.6887

Random effects:
 Groups                       Name        Variance Std.Dev.
 Genotype_Zone_1:Plant_Zone_1 (Intercept) 0.000    0.000
 Genotype_Zone_2:Plant_Zone_2 (Intercept) 0.000    0.000
 Residual                                 3.331    1.825
Number of obs: 39, groups:  Genotype_Zone_1:Plant_Zone_1, 10; Genotype_Zone_2:Plant_Zone_2, 10

Fixed effects:
            Estimate Std. Error      df t value Pr(>|t|)
(Intercept)   7.7271     0.2923 38.0000   26.44   <2e-16 ***
---
Signif. codes:  0 '***' 0.001 '**' 0.01 '*' 0.05 '.' 0.1 ' ' 1
```

Model residuals

Statistic	Value
Sample skewness	-3.495
Sample excess kurtosis	12.4
Passed Shapiro Wilk normality test	No (p-value = 3.854E-10 < 0.05)

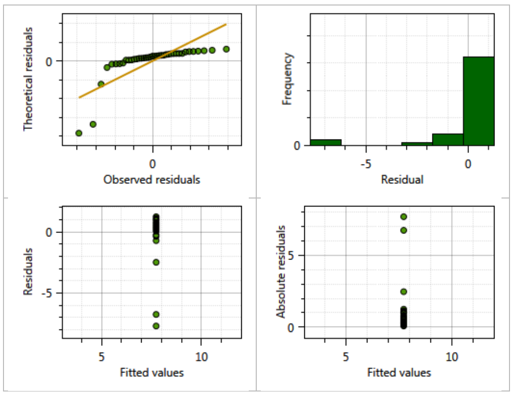

Analysis estimated duration moving (Zone 2)

**Analysis model**	Linear mixed model fit by REML: Estimated_duration_moving_Zone_2 ~ 1 + (1|Genotype_Zone_1:Plant_Zone_1) + (1|Genotype_Zone_2:Plant_Zone_2)
**Transformation**	Natural logarithm

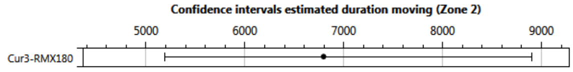

Confidence intervals estimated duration moving (Zone 2)

Genotype Zone 1	Genotype Zone 2	Mean	Lower 95% CL	Upper 95% CL	Group
Cur3	RMX180	6796	5186	8905	a

Model summary

```
Linear mixed model fit by REML. t-tests use Satterthwaite's method ['lmerModLmerTest']
Formula: Estimated_duration_moving_Zone_2 ~ 1 + (1 | Genotype_Zone_1:Plant_Zone_1) +      (1 |
Genotype_Zone_2:Plant_Zone_2)
   Data: data

REML criterion at convergence: 49.5
```

```
Scaled residuals:
     Min       1Q   Median       3Q      Max
-3.15723 -0.46555  0.03048  0.79248  1.37299

Random effects:
 Groups                        Name        Variance Std.Dev.
 Genotype_Zone_1:Plant_Zone_1 (Intercept) 0.001133 0.03366
 Genotype_Zone_2:Plant_Zone_2 (Intercept) 0.035655 0.18883
 Residual                                 0.168910 0.41099
Number of obs: 39, groups:  Genotype_Zone_1:Plant_Zone_1, 10; Genotype_Zone_2:Plant_Zone_2, 10

Fixed effects:
            Estimate Std. Error      df t value Pr(>|t|)
(Intercept)  8.82405    0.08957 3.31508   98.51  7.3e-07 ***
---
Signif. codes:  0 '***' 0.001 '**' 0.01 '*' 0.05 '.' 0.1 ' ' 1
```

Model residuals

Statistic	Value
Sample skewness	-1.258
Sample excess kurtosis	2.288
Passed Shapiro Wilk normality test	No (p-value = 0.002427 < 0.05)

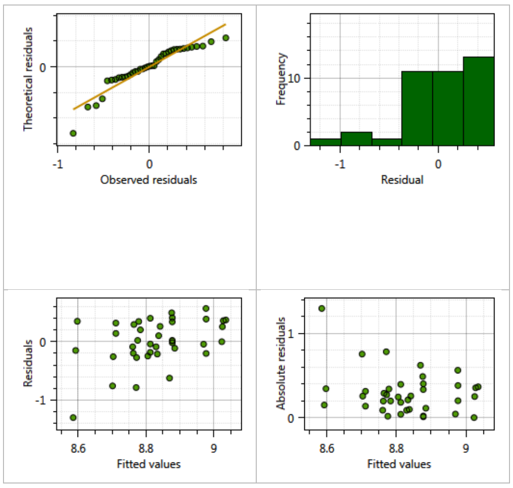

Analysis estimated duration moving (diff. Zone 1 - Zone 2)

**Analysis model**	Generalized linear mixed model with dispersion factor, formula=cbind(Estimated_duration_moving_Zone_1,Estimated_duration_moving_Zone_2) ~ 1 + (1|Genotype_Zone_1:Plant_Zone_1) + (1|Genotype_Zone_2:Plant_Zone_2)
**Transformation**	Logit

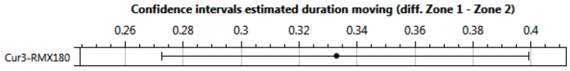

Confidence intervals estimated duration moving (diff. Zone 1 - Zone 2)

Genotype Zone 1	Genotype Zone 2	Mean	Lower 95% CL	Upper 95% CL	Group
Cur3	RMX180	0.3329	0.2725	0.3993	a

Model summary

```
Linear mixed model fit by REML. t-tests use Satterthwaite's method ['lmerModLmerTest']
Formula: ziFormula
   Data: data
Weights: wi

REML criterion at convergence: 82.2

Scaled residuals:
     Min       1Q   Median       3Q      Max
-1.46735 -0.78687 -0.05515  0.82239  1.55168

Random effects:
 Groups                        Name        Variance  Std.Dev.
 Genotype_Zone_1:Plant_Zone_1 (Intercept) 8.769e-03  0.09364
 Genotype_Zone_2:Plant_Zone_2 (Intercept) 5.188e-02  0.22777
 Residual                                 9.356e+02 30.58728
Number of obs: 39, groups:  Genotype_Zone_1:Plant_Zone_1, 10; Genotype_Zone_2:Plant_Zone_2, 10

Fixed effects:
            Estimate Std. Error      df t value Pr(>|t|)
(Intercept)  -0.6951     0.1265  8.8687  -5.494 0.000403 ***
---
Signif. codes:  0 '***' 0.001 '**' 0.01 '*' 0.05 '.' 0.1 ' ' 1

Dispersion: 30.59
```

Model residuals

Statistic	Value
Sample skewness	0.1232
Sample excess kurtosis	-1.244
Passed Shapiro Wilk normality test	No (p-value = 0.04148 < 0.05)

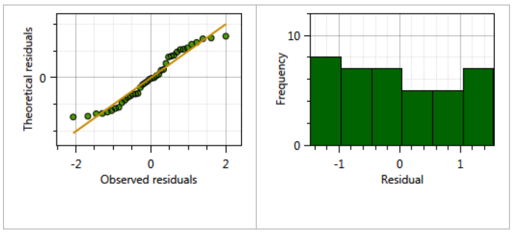

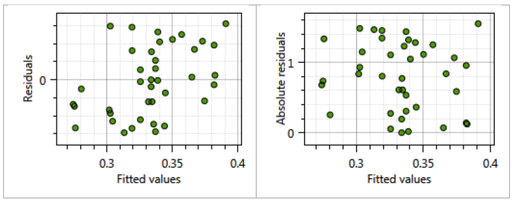

Estimated duration moving per zone per hour

Selected hours	0, 1, 2, 3, 4, 5, 6, 7
Selected zones	Zone 1, Zone 2
Data transformation	Natural logarithm
Analysis	Zone difference analysis

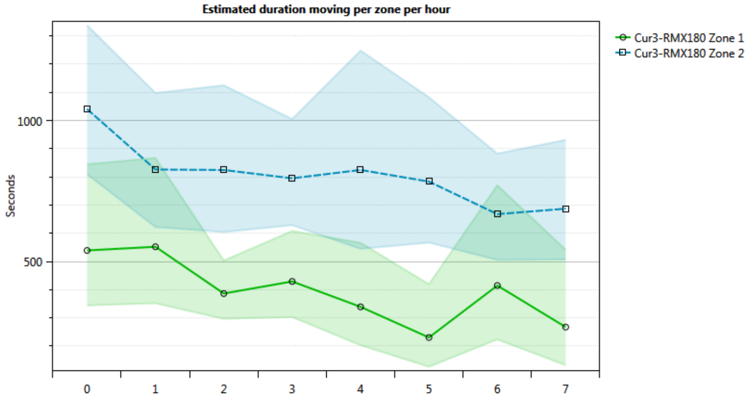

Results difference tests Zone 1 - Zone 2: p values and 95% confidence intervals of the difference on the transformed scale for each statistic.

Behaviour statistic	Cur3-RMX180	Remark
Estimated duration moving H0 (diff. Zone 1 - Zone 2)	p=0.0451*<br>[-0.955, -0.0133]	
Estimated duration moving H1 (diff. Zone 1 - Zone 2)	p=0.0157*<br>[-0.656, -0.073]	
Estimated duration moving H2 (diff. Zone 1 - Zone 2)	p=0.00426**<br>[-1.09, -0.306]	CR
Estimated duration moving H3 (diff. Zone 1 - Zone 2)	p=0.00289**<br>[-1.02, -0.28]	
Estimated duration moving H4 (diff. Zone 1 - Zone 2)	p=0.0127*<br>[-1.66, -0.264]	CR
Estimated duration moving H5 (diff. Zone 1 - Zone 2)	p=0.00138**<br>[-1.55, -0.507]	CR
Estimated duration moving H6 (diff. Zone 1 - Zone 2)	p=0.00339**<br>[-1.2, -0.305]	
Estimated duration moving H7 (diff. Zone 1 - Zone 2)	p=0.00891**<br>[-1.42, -0.28]	CR

CR = Check residuals

The model predictions and 95% confidence intervals for each statistic.

Statistic	Cur3-RMX180	Remark
Estimated duration moving (H0 - Zone 1)	539<br>[344, 845]	CR
Estimated duration moving (H0 - Zone 2)	1.04E+03<br>[810, 1.34E+03]	CR
Estimated duration moving (H1 - Zone 1)	552<br>[352, 868]	CR
Estimated duration moving (H1 - Zone 2)	827<br>[622, 1.1E+03]	CR
Estimated duration moving (H2 - Zone 1)	386<br>[297, 504]	
Estimated duration moving (H2 - Zone 2)	825<br>[604, 1.13E+03]	CR
Estimated duration moving (H3 - Zone 1)	429<br>[303, 609]	CR
Estimated duration moving (H3 - Zone 2)	795<br>[629, 1.01E+03]	
Estimated duration moving (H4 - Zone 1)	339<br>[203, 567]	
Estimated duration moving (H4 - Zone 2)	825<br>[545, 1.25E+03]	CR
Estimated duration moving (H5 - Zone 1)	230<br>[127, 420]	CR
Estimated duration moving (H5 - Zone 2)	784<br>[567, 1.08E+03]	
Estimated duration moving (H6 - Zone 1)	415<br>[223, 771]	CR
Estimated duration moving (H6 - Zone 2)	668<br>[506, 883]	CR
Estimated duration moving (H7 - Zone 1)	267<br>[132, 541]	CR
Estimated duration moving (H7 - Zone 2)	688<br>[507, 932]	

CR = Check residuals

Data summary

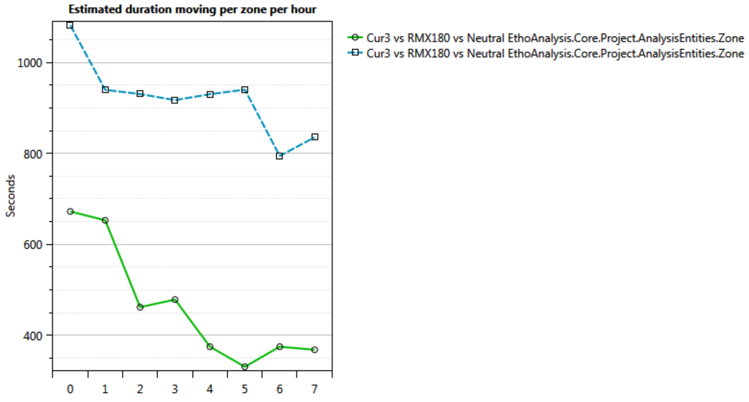

Genotype Zone 1	Genotype Zone 2	Genotype Zone 3	Mean H0 - Zone 1	StdErr H0 - Zone 1	Mean H0 - Zone 2	StdErr H0 - Zone 2	Mean H1 - Zone 1	StdErr H1 - Zone 1	Mean H1 - Zone 2	StdErr H1 - Zone 2	Mean H2 - Zone 1	StdErr H2 - Zone 1	Mean H2 - Zone 2	StdErr H2 - Zone 2
Cur3	RMX180	Neutral	672.24	87.67	1081.73	83.73	652.88	61.4	939.9	81.45	461.8	58.06	930.55	77.86

Genotype Zone 1	Mean H3 - Zone 1	StdErr H3 - Zone 1	Mean H3 - Zone 2	StdErr H3 - Zone 2	Mean H4 - Zone 1	StdErr H4 - Zone 1	Mean H4 - Zone 2	StdErr H4 - Zone 2	Mean H5 - Zone 1	StdErr H5 - Zone 1	Mean H5 - Zone 2	StdErr H5 - Zone 2	Mean H6 - Zone 1	StdErr H6 - Zone 1	Mean H6 - Zone 2	S H Z 2
Cur3	478.53	57.52	917.12	74.22	374.93	57.89	930.18	84.68	330.74	50.61	940.23	84.42	374.87	60.4	794.43	7[illegible]

Analysis estimated duration moving (H0 - Zone 1)

**Analysis model**	Linear mixed model fit by REML: Estimated_duration_moving_H0_Zone_1 ~ 1 + (1|Genotype_Zone_1:Plant_Zone_1) + (1|Genotype_Zone_2:Plant_Zone_2)
**Transformation**	Natural logarithm

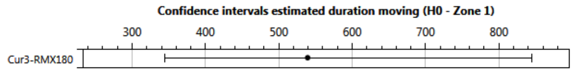

Confidence intervals estimated duration moving (H0 - Zone 1)

Genotype Zone 1	Genotype Zone 2	Mean	Lower 95% CL	Upper 95% CL	Group
Cur3	RMX180	539.2	344.1	845.2	a

Model summary

```
Linear mixed model fit by REML. t-tests use Satterthwaite's method ['lmerModLmerTest']
Formula: Estimated_duration_moving_H0_Zone_1 ~ 1 + (1 | Genotype_Zone_1:Plant_Zone_1) +      (1 |
Genotype_Zone_2:Plant_Zone_2)
   Data: data

REML criterion at convergence: 109.5

Scaled residuals:
    Min      1Q  Median      3Q     Max
-3.8311 -0.1308  0.2311  0.4814  0.9806

Random effects:
 Groups                       Name        Variance  Std.Dev.
 Genotype_Zone_1:Plant_Zone_1 (Intercept) 4.556e-20 2.135e-10
 Genotype_Zone_2:Plant_Zone_2 (Intercept) 0.000e+00 0.000e+00
 Residual                                 1.606e+00 1.267e+00
Number of obs: 33, groups:  Genotype_Zone_1:Plant_Zone_1, 10; Genotype_Zone_2:Plant_Zone_2, 10

Fixed effects:
            Estimate Std. Error      df t value Pr(>|t|)
(Intercept)   6.2902     0.2206 32.0000   28.51   <2e-16 ***
---
Signif. codes:  0 '***' 0.001 '**' 0.01 '*' 0.05 '.' 0.1 ' ' 1
```

Model residuals

Statistic	Value
Sample skewness	-2.549
Sample excess kurtosis	7.58
Passed Shapiro Wilk normality test	No (p-value = 1.629E-06 < 0.05)

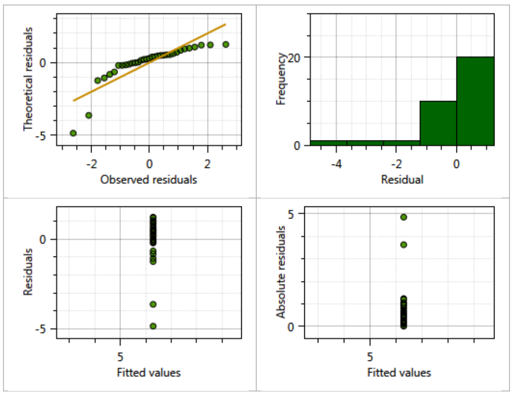

Analysis estimated duration moving (H0 - Zone 2)

**Analysis model**	Linear mixed model fit by REML: Estimated_duration_moving_H0_Zone_2 ~ 1 + (1|Genotype_Zone_1:Plant_Zone_1) + (1|Genotype_Zone_2:Plant_Zone_2)
**Transformation**	Natural logarithm

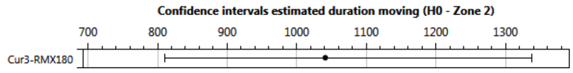

Confidence intervals estimated duration moving (H0 - Zone 2)

Genotype Zone 1	Genotype Zone 2	Mean	Lower 95% CL	Upper 95% CL	Group
Cur3	RMX180	1041	809.8	1338	a

Model summary

```
Linear mixed model fit by REML. t-tests use Satterthwaite's method ['lmerModLmerTest']
Formula: Estimated_duration_moving_H0_Zone_2 ~ 1 + (1 | Genotype_Zone_1:Plant_Zone_1) +      (1 |
Genotype_Zone_2:Plant_Zone_2)
   Data: data
```

```
REML criterion at convergence: 65.2

Scaled residuals:
    Min      1Q  Median      3Q     Max
-3.7717 -0.2284  0.2504  0.5697  1.1864

Random effects:
 Groups                      Name        Variance Std.Dev.
 Genotype_Zone_1:Plant_Zone_1 (Intercept) 0.00000  0.0000
 Genotype_Zone_2:Plant_Zone_2 (Intercept) 0.01979  0.1407
 Residual                                 0.32348  0.5688
Number of obs: 36, groups:  Genotype_Zone_1:Plant_Zone_1, 10; Genotype_Zone_2:Plant_Zone_2, 10

Fixed effects:
            Estimate Std. Error    df t value Pr(>|t|)
(Intercept)    6.948      0.105 6.623   66.18 1.36e-10 ***
---
Signif. codes:  0 '***' 0.001 '**' 0.01 '*' 0.05 '.' 0.1 ' ' 1
```

Model residuals

Statistic	Value
Sample skewness	-2.31
Sample excess kurtosis	6.57
Passed Shapiro Wilk normality test	No (p-value = 5.238E-06 < 0.05)

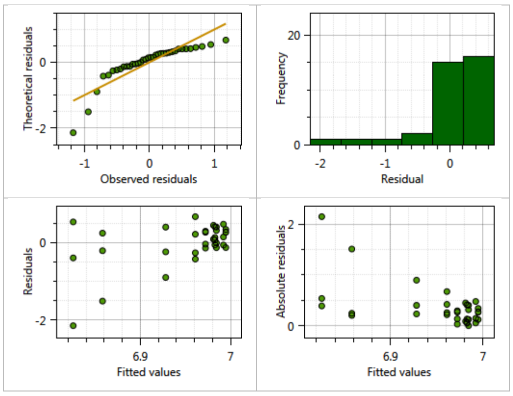

Analysis estimated duration moving (H1 - Zone 1)

**Analysis model**	Linear mixed model fit by REML: Estimated_duration_moving_H1_Zone_1 ~ 1 + (1|Genotype_Zone_1:Plant_Zone_1) + (1|Genotype_Zone_2:Plant_Zone_2)
**Transformation**	Natural logarithm

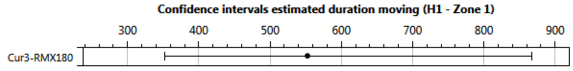

Confidence intervals estimated duration moving (H1 - Zone 1)

Genotype Zone 1	Genotype Zone 2	Mean	Lower 95% CL	Upper 95% CL	Group
Cur3	RMX180	552.4	351.7	867.6	a

Model summary

```
Linear mixed model fit by REML. t-tests use Satterthwaite's method ['lmerModLmerTest']
Formula: Estimated_duration_moving_H1_Zone_1 ~ 1 + (1 | Genotype_Zone_1:Plant_Zone_1) +      (1 |
Genotype_Zone_2:Plant_Zone_2)
   Data: data

REML criterion at convergence: 110.2

Scaled residuals:
    Min      1Q  Median      3Q     Max
-5.2367 -0.1481  0.1698  0.4410  0.8769

Random effects:
 Groups                      Name        Variance Std.Dev.
 Genotype_Zone_1:Plant_Zone_1 (Intercept) 0.000000 0.0000
 Genotype_Zone_2:Plant_Zone_2 (Intercept) 0.007191 0.0848
 Residual                                 1.224830 1.1067
Number of obs: 36, groups:  Genotype_Zone_1:Plant_Zone_1, 10; Genotype_Zone_2:Plant_Zone_2, 10

Fixed effects:
            Estimate Std. Error     df t value Pr(>|t|)
(Intercept)   6.3142     0.1864 6.2664   33.87 2.46e-08 ***
---
Signif. codes:  0 '***' 0.001 '**' 0.01 '*' 0.05 '.' 0.1 ' ' 1
```

Model residuals

Statistic	Value
Sample skewness	-4.323
Sample excess kurtosis	22.62
Passed Shapiro Wilk normality test	No (p-value = 3.192E-09 < 0.05)

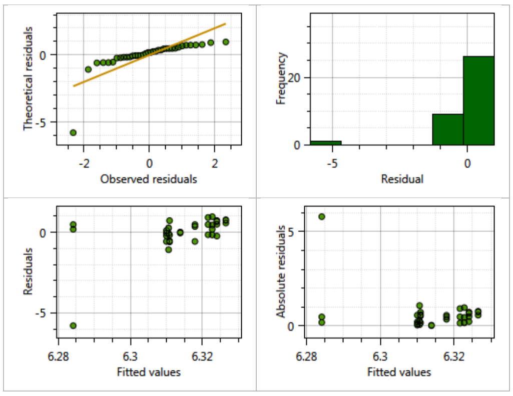

Analysis estimated duration moving (H1 - Zone 2)

**Analysis model**	Linear mixed model fit by REML: Estimated_duration_moving_H1_Zone_2 ~ 1 + (1|Genotype_Zone_1:Plant_Zone_1) + (1|Genotype_Zone_2:Plant_Zone_2)
**Transformation**	Natural logarithm

Confidence intervals estimated duration moving (H1 - Zone 2)

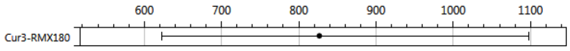

Confidence intervals estimated duration moving (H1 - Zone 2)

Genotype Zone 1	Genotype Zone 2	Mean	Lower 95% CL	Upper 95% CL	Group
Cur3	RMX180	826.6	622.1	1098	a

Model summary

```
Linear mixed model fit by REML. t-tests use Satterthwaite's method ['lmerModLmerTest']
Formula: Estimated_duration_moving_H1_Zone_2 ~ 1 + (1 | Genotype_Zone_1:Plant_Zone_1) +      (1 |
Genotype_Zone_2:Plant_Zone_2)
   Data: data

REML criterion at convergence: 81.2

Scaled residuals:
    Min      1Q  Median      3Q     Max
-3.0629 -0.3097  0.3666  0.6545  1.1656

Random effects:
 Groups                       Name        Variance Std.Dev.
 Genotype_Zone_1:Plant_Zone_1 (Intercept) 0.00000  0.0000
 Genotype_Zone_2:Plant_Zone_2 (Intercept) 0.01707  0.1307
 Residual                                 0.49055  0.7004
Number of obs: 37, groups:  Genotype_Zone_1:Plant_Zone_1, 10; Genotype_Zone_2:Plant_Zone_2, 10

Fixed effects:
            Estimate Std. Error     df t value Pr(>|t|)
(Intercept)   6.7173     0.1224 7.7210   54.87 2.74e-11 ***
---
Signif. codes:  0 '***' 0.001 '**' 0.01 '*' 0.05 '.' 0.1 ' ' 1
```

Model residuals

Statistic	Value
Sample skewness	-1.566
Sample excess kurtosis	2.285
Passed Shapiro Wilk normality test	No (p-value = 9.811E-05 < 0.05)

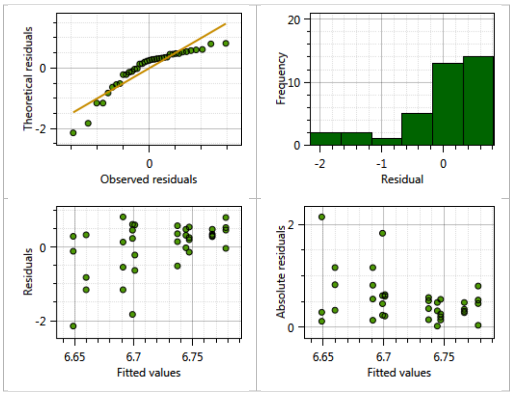

Analysis estimated duration moving (H2 - Zone 1)

**Analysis model**	Linear mixed model fit by REML: Estimated_duration_moving_H2_Zone_1 ~ 1 + (1|Genotype_Zone_1:Plant_Zone_1) + (1|Genotype_Zone_2:Plant_Zone_2)
**Transformation**	Natural logarithm

Confidence intervals estimated duration moving (H2 - Zone 1)

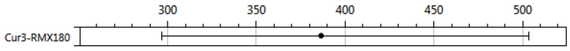

Confidence intervals estimated duration moving (H2 - Zone 1)

Genotype Zone 1	Genotype Zone 2	Mean	Lower 95% CL	Upper 95% CL	Group
Cur3	RMX180	386.4	296.5	503.6	a

Model summary

```
Linear mixed model fit by REML. t-tests use Satterthwaite's method ['lmerModLmerTest']
Formula: Estimated_duration_moving_H2_Zone_1 ~ 1 + (1 | Genotype_Zone_1:Plant_Zone_1) +      (1 |
Genotype_Zone_2:Plant_Zone_2)
   Data: data

REML criterion at convergence: 85.8

Scaled residuals:
     Min       1Q   Median       3Q      Max
-2.69890 -0.55542  0.09798  0.69664  1.85849

Random effects:
 Groups                       Name        Variance Std.Dev.
 Genotype_Zone_1:Plant_Zone_1 (Intercept) 0.000    0.0000
 Genotype_Zone_2:Plant_Zone_2 (Intercept) 0.000    0.0000
 Residual                                 0.613    0.7829
Number of obs: 36, groups:  Genotype_Zone_1:Plant_Zone_1, 10; Genotype_Zone_2:Plant_Zone_2, 10

Fixed effects:
            Estimate Std. Error      df t value Pr(>|t|)
(Intercept)   5.9570     0.1305 35.0000   45.65   <2e-16 ***
---
Signif. codes:  0 '***' 0.001 '**' 0.01 '*' 0.05 '.' 0.1 ' ' 1
```

Model residuals

Statistic	Value
Sample skewness	-0.5891
Sample excess kurtosis	0.3496
Passed Shapiro Wilk normality test	Yes (p-value = 0.6406 > 0.05)

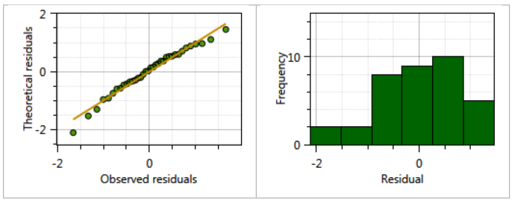

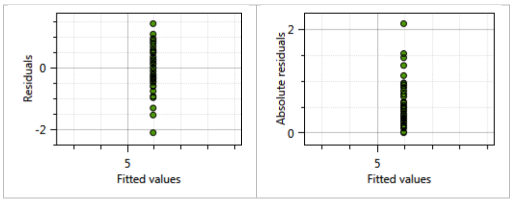

Analysis estimated duration moving (H2 - Zone 2)

**Analysis model**	Linear mixed model fit by REML: Estimated_duration_moving_H2_Zone_2 ~ 1 + (1|Genotype_Zone_1:Plant_Zone_1) + (1|Genotype_Zone_2:Plant_Zone_2)
**Transformation**	Natural logarithm

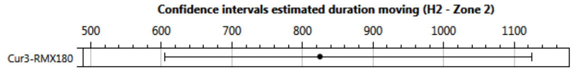

Confidence intervals estimated duration moving (H2 - Zone 2)

Genotype Zone 1	Genotype Zone 2	Mean	Lower 95% CL	Upper 95% CL	Group
Cur3	RMX180	824.7	604.4	1125	a

Model summary

```
Linear mixed model fit by REML. t-tests use Satterthwaite's method ['lmerModLmerTest']
Formula: Estimated_duration_moving_H2_Zone_2 ~ 1 + (1 | Genotype_Zone_1:Plant_Zone_1) +      (1 |
Genotype_Zone_2:Plant_Zone_2)
   Data: data

REML criterion at convergence: 77.4

Scaled residuals:
    Min      1Q  Median      3Q     Max
-3.4025 -0.0777  0.2117  0.5198  1.4051

Random effects:
 Groups                       Name        Variance Std.Dev.
 Genotype_Zone_1:Plant_Zone_1 (Intercept) 0.06992  0.2644
 Genotype_Zone_2:Plant_Zone_2 (Intercept) 0.00000  0.0000
 Residual                                 0.40132  0.6335
Number of obs: 37, groups:  Genotype_Zone_1:Plant_Zone_1, 10; Genotype_Zone_2:Plant_Zone_2, 10

Fixed effects:
            Estimate Std. Error     df t value Pr(>|t|)
(Intercept)   6.7150     0.1338 7.6958   50.18  5.8e-11 ***
---
Signif. codes:  0 '***' 0.001 '**' 0.01 '*' 0.05 '.' 0.1 ' ' 1
```

Model residuals

Statistic	Value
Sample skewness	-1.596
Sample excess kurtosis	3.435
Passed Shapiro Wilk normality test	No (p-value = 0.0002749 < 0.05)

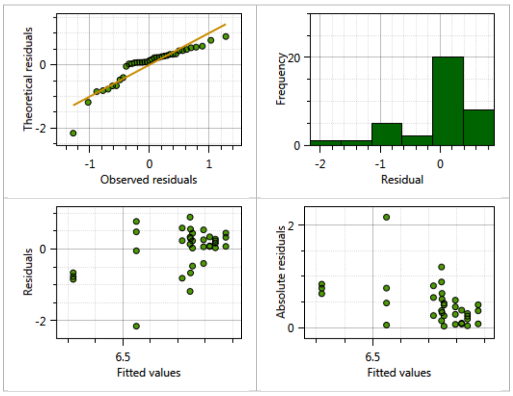

Analysis estimated duration moving (H3 - Zone 1)

**Analysis model**	Linear mixed model fit by REML: Estimated_duration_moving_H3_Zone_1 ~ 1 + (1|Genotype_Zone_1:Plant_Zone_1) + (1|Genotype_Zone_2:Plant_Zone_2)
**Transformation**	Natural logarithm

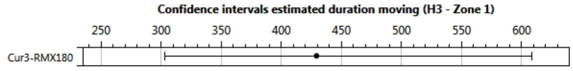

Confidence intervals estimated duration moving (H3 - Zone 1)

Genotype Zone 1	Genotype Zone 2	Mean	Lower 95% CL	Upper 95% CL	Group
Cur3	RMX180	429.2	302.7	608.6	a

Model summary

```
Linear mixed model fit by REML. t-tests use Satterthwaite's method ['lmerModLmerTest']
Formula: Estimated_duration_moving_H3_Zone_1 ~ 1 + (1 | Genotype_Zone_1:Plant_Zone_1) +      (1 |
Genotype_Zone_2:Plant_Zone_2)
   Data: data

REML criterion at convergence: 93.3

Scaled residuals:
    Min      1Q  Median      3Q     Max
-3.9937 -0.0793  0.2235  0.5723  1.1327

Random effects:
 Groups                       Name        Variance Std.Dev.
 Genotype_Zone_1:Plant_Zone_1 (Intercept) 0.0000   0.0000
 Genotype_Zone_2:Plant_Zone_2 (Intercept) 0.0000   0.0000
 Residual                                 0.9696   0.9847
Number of obs: 33, groups:  Genotype_Zone_1:Plant_Zone_1, 10; Genotype_Zone_2:Plant_Zone_2, 10

Fixed effects:
            Estimate Std. Error      df t value Pr(>|t|)
(Intercept)   6.0620     0.1714 32.0000   35.37   <2e-16 ***
---
Signif. codes:  0 '***' 0.001 '**' 0.01 '*' 0.05 '.' 0.1 ' ' 1
```

Model residuals

Statistic	Value
Sample skewness	-2.361
Sample excess kurtosis	7.32
Passed Shapiro Wilk normality test	No (p-value = 1.009E-05 < 0.05)

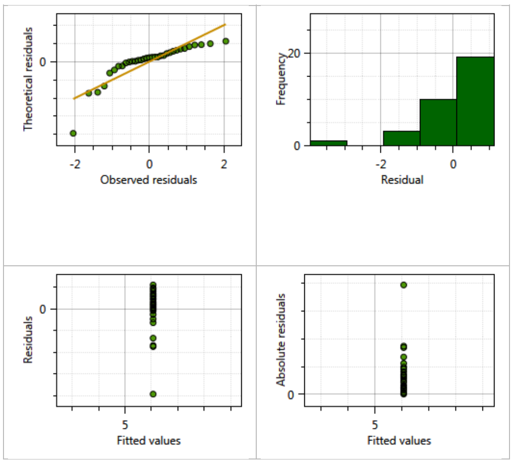

Analysis estimated duration moving (H3 - Zone 2)

**Analysis model**	Linear mixed model fit by REML: Estimated_duration_moving_H3_Zone_2 ~ 1 + (1|Genotype_Zone_1:Plant_Zone_1) + (1|Genotype_Zone_2:Plant_Zone_2)
**Transformation**	Natural logarithm

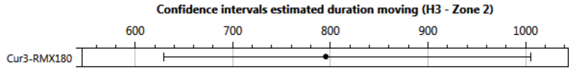

Confidence intervals estimated duration moving (H3 - Zone 2)

Genotype Zone 1	Genotype Zone 2	Mean	Lower 95% CL	Upper 95% CL	Group
Cur3	RMX180	795.3	628.9	1006	a

Model summary

```
Linear mixed model fit by REML. t-tests use Satterthwaite's method ['lmerModLmerTest']
Formula: Estimated_duration_moving_H3_Zone_2 ~ 1 + (1 | Genotype_Zone_1:Plant_Zone_1) +      (1 |
Genotype_Zone_2:Plant_Zone_2)
   Data: data

REML criterion at convergence: 67.4

Scaled residuals:
    Min      1Q  Median      3Q     Max
-2.6058 -0.3872  0.1659  0.5740  1.4811

Random effects:
 Groups                       Name        Variance Std.Dev.
 Genotype_Zone_1:Plant_Zone_1 (Intercept) 0.00000  0.0000
 Genotype_Zone_2:Plant_Zone_2 (Intercept) 0.03026  0.1740
 Residual                                 0.28930  0.5379
Number of obs: 39, groups:  Genotype_Zone_1:Plant_Zone_1, 10; Genotype_Zone_2:Plant_Zone_2, 10

Fixed effects:
            Estimate Std. Error     df t value Pr(>|t|)
(Intercept)   6.6787     0.1023 8.2299   65.31 1.81e-12 ***
---
Signif. codes:  0 '***' 0.001 '**' 0.01 '*' 0.05 '.' 0.1 ' ' 1
```

Model residuals

Statistic	Value
Sample skewness	-0.6682
Sample excess kurtosis	0.1274
Passed Shapiro Wilk normality test	Yes (p-value = 0.1016 > 0.05)

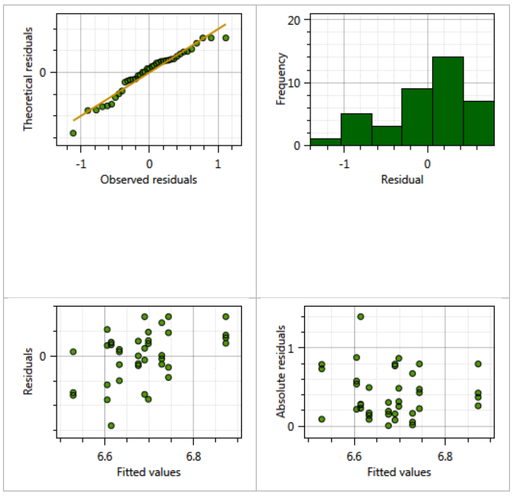

Analysis estimated duration moving (H4 - Zone 1)

**Analysis model**	Linear mixed model fit by REML: Estimated_duration_moving_H4_Zone_1 ~ 1 + (1|Genotype_Zone_1:Plant_Zone_1) + (1|Genotype_Zone_2:Plant_Zone_2)
**Transformation**	Natural logarithm

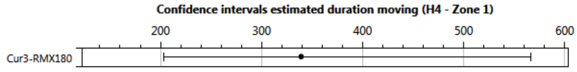

Confidence intervals estimated duration moving (H4 - Zone 1)

Genotype Zone 1	Genotype Zone 2	Mean	Lower 95% CL	Upper 95% CL	Group
Cur3	RMX180	339.1	202.9	566.7	a

Model summary

```
Linear mixed model fit by REML. t-tests use Satterthwaite's method ['lmerModLmerTest']
Formula: Estimated_duration_moving_H4_Zone_1 ~ 1 + (1 | Genotype_Zone_1:Plant_Zone_1) +      (1 |
Genotype_Zone_2:Plant_Zone_2)
   Data: data

REML criterion at convergence: 75.5

Scaled residuals:
     Min       1Q   Median       3Q      Max
-2.13875 -0.58884  0.09216  0.50197  1.54447

Random effects:
 Groups                       Name        Variance Std.Dev.
 Genotype_Zone_1:Plant_Zone_1 (Intercept) 0.0000   0.0000
 Genotype_Zone_2:Plant_Zone_2 (Intercept) 0.3169   0.5630
 Residual                                 0.5101   0.7142
Number of obs: 30, groups:  Genotype_Zone_1:Plant_Zone_1, 10; Genotype_Zone_2:Plant_Zone_2, 10

Fixed effects:
            Estimate Std. Error     df t value Pr(>|t|)
(Intercept)   5.8262     0.2225 7.9488   26.19 5.32e-09 ***
---
Signif. codes:  0 '***' 0.001 '**' 0.01 '*' 0.05 '.' 0.1 ' ' 1
```

Model residuals

Statistic	Value
Sample skewness	-0.3674
Sample excess kurtosis	-0.1597
Passed Shapiro Wilk normality test	Yes (p-value = 0.79 > 0.05)

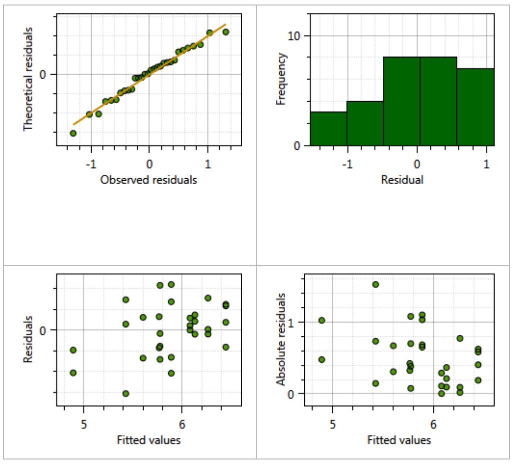

Analysis estimated duration moving (H4 - Zone 2)

**Analysis model**	Linear mixed model fit by REML: Estimated_duration_moving_H4_Zone_2 ~ 1 + (1|Genotype_Zone_1:Plant_Zone_1) + (1|Genotype_Zone_2:Plant_Zone_2)
**Transformation**	Natural logarithm

Confidence intervals estimated duration moving (H4 - Zone 2)

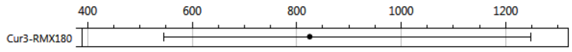

Confidence intervals estimated duration moving (H4 - Zone 2)

Genotype Zone 1	Genotype Zone 2	Mean	Lower 95% CL	Upper 95% CL	Group
Cur3	RMX180	824.9	545	1249	a

Model summary

```
Linear mixed model fit by REML. t-tests use Satterthwaite's method ['lmerModLmerTest']
Formula: Estimated_duration_moving_H4_Zone_2 ~ 1 + (1 | Genotype_Zone_1:Plant_Zone_1) +      (1 |
Genotype_Zone_2:Plant_Zone_2)
   Data: data

REML criterion at convergence: 71.9

Scaled residuals:
    Min      1Q  Median      3Q     Max
-2.4442 -0.4414  0.1686  0.6439  1.3418

Random effects:
 Groups                       Name        Variance Std.Dev.
 Genotype_Zone_1:Plant_Zone_1 (Intercept) 0.04075  0.2019
 Genotype_Zone_2:Plant_Zone_2 (Intercept) 0.06681  0.2585
 Residual                                 0.30959  0.5564
Number of obs: 37, groups:  Genotype_Zone_1:Plant_Zone_1, 10; Genotype_Zone_2:Plant_Zone_2, 10

Fixed effects:
            Estimate Std. Error     df t value Pr(>|t|)
(Intercept)   6.7153     0.1389 3.3942   48.33 6.14e-06 ***
---
Signif. codes:  0 '***' 0.001 '**' 0.01 '*' 0.05 '.' 0.1 ' ' 1
```

Model residuals

Statistic	Value
Sample skewness	-0.8454
Sample excess kurtosis	0.3786
Passed Shapiro Wilk normality test	No (p-value = 0.04813 < 0.05)

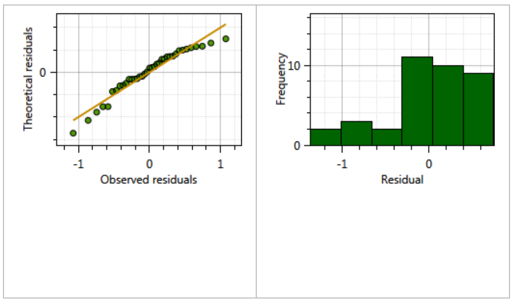

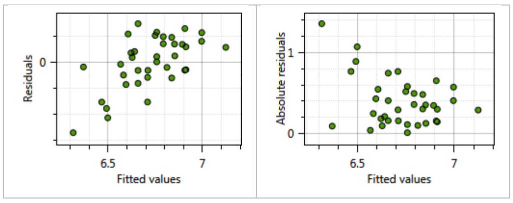

Analysis estimated duration moving (H5 - Zone 1)

**Analysis model**	Linear mixed model fit by REML: Estimated_duration_moving_H5_Zone_1 ~ 1 + (1|Genotype_Zone_1:Plant_Zone_1) + (1|Genotype_Zone_2:Plant_Zone_2)
**Transformation**	Natural logarithm

Confidence intervals estimated duration moving (H5 - Zone 1)

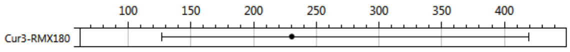

Confidence intervals estimated duration moving (H5 - Zone 1)

Genotype Zone 1	Genotype Zone 2	Mean	Lower 95% CL	Upper 95% CL	Group
Cur3	RMX180	230.4	126.5	419.7	a

Model summary

```
Linear mixed model fit by REML. t-tests use Satterthwaite's method ['lmerModLmerTest']
Formula: Estimated_duration_moving_H5_Zone_1 ~ 1 + (1 | Genotype_Zone_1:Plant_Zone_1) +      (1 |
Genotype_Zone_2:Plant_Zone_2)
   Data: data

REML criterion at convergence: 118

Scaled residuals:
    Min      1Q  Median      3Q     Max
-3.6409 -0.2410  0.3382  0.5687  0.9498

Random effects:
 Groups                       Name        Variance Std.Dev.
 Genotype_Zone_1:Plant_Zone_1 (Intercept) 0.000    0.000
 Genotype_Zone_2:Plant_Zone_2 (Intercept) 0.000    0.000
 Residual                                 2.671    1.634
Number of obs: 31, groups:  Genotype_Zone_1:Plant_Zone_1, 10; Genotype_Zone_2:Plant_Zone_2, 10

Fixed effects:
            Estimate Std. Error      df t value Pr(>|t|)
(Intercept)   5.4399     0.2935 30.0000   18.53   <2e-16 ***
---
Signif. codes:  0 '***' 0.001 '**' 0.01 '*' 0.05 '.' 0.1 ' ' 1
```

Model residuals

Statistic	Value
Sample skewness	-2.205
Sample excess kurtosis	5.419
Passed Shapiro Wilk normality test	No (p-value = 8.417E-06 < 0.05)

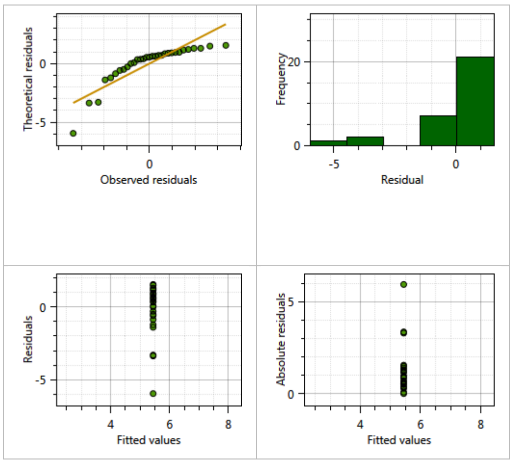

Analysis estimated duration moving (H5 - Zone 2)

**Analysis model**	Linear mixed model fit by REML: Estimated_duration_moving_H5_Zone_2 ~ 1 + (1|Genotype_Zone_1:Plant_Zone_1) + (1|Genotype_Zone_2:Plant_Zone_2)
**Transformation**	Natural logarithm

Confidence intervals estimated duration moving (H5 - Zone 2)

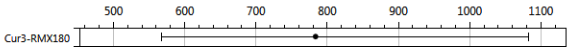

Confidence intervals estimated duration moving (H5 - Zone 2)

Genotype Zone 1	Genotype Zone 2	Mean	Lower 95% CL	Upper 95% CL	Group
Cur3	RMX180	783.6	566.9	1083	a

Model summary

```
Linear mixed model fit by REML. t-tests use Satterthwaite's method ['lmerModLmerTest']
Formula: Estimated_duration_moving_H5_Zone_2 ~ 1 + (1 | Genotype_Zone_1:Plant_Zone_1) +      (1 |
Genotype_Zone_2:Plant_Zone_2)
   Data: data

REML criterion at convergence: 71.8

Scaled residuals:
    Min      1Q  Median      3Q     Max
-2.1665 -0.7662  0.1534  0.7225  1.4448

Random effects:
 Groups                       Name        Variance  Std.Dev.
 Genotype_Zone_1:Plant_Zone_1 (Intercept) 2.573e-15 5.072e-08
 Genotype_Zone_2:Plant_Zone_2 (Intercept) 1.308e-01 3.617e-01
 Residual                                 2.744e-01 5.239e-01
Number of obs: 39, groups:  Genotype_Zone_1:Plant_Zone_1, 10; Genotype_Zone_2:Plant_Zone_2, 10

Fixed effects:
            Estimate Std. Error    df t value Pr(>|t|)
(Intercept)    6.664      0.142 8.556   46.95 1.27e-11 ***
---
Signif. codes:  0 '***' 0.001 '**' 0.01 '*' 0.05 '.' 0.1 ' ' 1
```

Model residuals

Statistic	Value
Sample skewness	-0.402
Sample excess kurtosis	-0.5149
Passed Shapiro Wilk normality test	Yes (p-value = 0.3341 > 0.05)

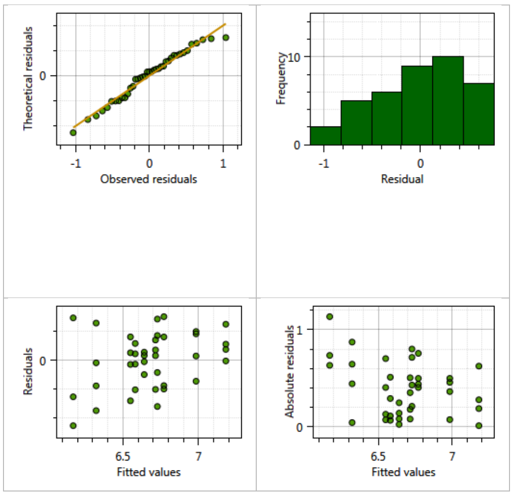

Analysis estimated duration moving (H6 - Zone 1)

**Analysis model**	Linear mixed model fit by REML: Estimated_duration_moving_H6_Zone_1 ~ 1 + (1|Genotype_Zone_1:Plant_Zone_1) + (1|Genotype_Zone_2:Plant_Zone_2)
**Transformation**	Natural logarithm

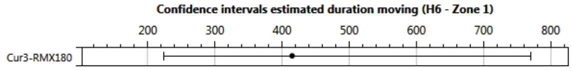

Confidence intervals estimated duration moving (H6 - Zone 1)

Genotype Zone 1	Genotype Zone 2	Mean	Lower 95% CL	Upper 95% CL	Group
Cur3	RMX180	414.9	223.3	770.8	a

Model summary

```
Linear mixed model fit by REML. t-tests use Satterthwaite's method ['lmerModLmerTest']
Formula: Estimated_duration_moving_H6_Zone_1 ~ 1 + (1 | Genotype_Zone_1:Plant_Zone_1) +      (1 |
Genotype_Zone_2:Plant_Zone_2)
   Data: data

REML criterion at convergence: 71.3

Scaled residuals:
    Min      1Q  Median      3Q     Max
-2.0821 -0.7107  0.2912  0.7461  1.1857

Random effects:
 Groups                       Name        Variance Std.Dev.
 Genotype_Zone_1:Plant_Zone_1 (Intercept) 0.08502  0.2916
 Genotype_Zone_2:Plant_Zone_2 (Intercept) 0.07435  0.2727
 Residual                                 0.66837  0.8175
Number of obs: 27, groups:  Genotype_Zone_1:Plant_Zone_1, 10; Genotype_Zone_2:Plant_Zone_2, 10

Fixed effects:
            Estimate Std. Error     df t value Pr(>|t|)
(Intercept)   6.0280     0.2055 3.3221   29.33 3.96e-05 ***
---
Signif. codes:  0 '***' 0.001 '**' 0.01 '*' 0.05 '.' 0.1 ' ' 1
```

Model residuals

Statistic	Value
Sample skewness	-0.8171
Sample excess kurtosis	-0.3317
Passed Shapiro Wilk normality test	No (p-value = 0.02286 < 0.05)

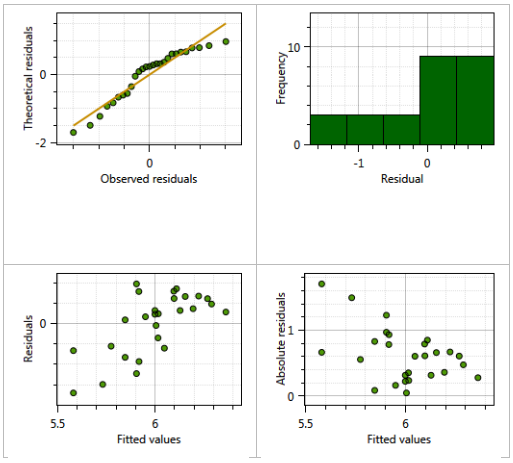

Analysis estimated duration moving (H6 - Zone 2)

**Analysis model**	Linear mixed model fit by REML: Estimated_duration_moving_H6_Zone_2 ~ 1 + (1|Genotype_Zone_1:Plant_Zone_1) + (1|Genotype_Zone_2:Plant_Zone_2)
**Transformation**	Natural logarithm

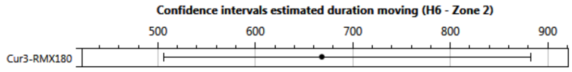

Genotype Zone 1	Genotype Zone 2	Mean	Lower 95% CL	Upper 95% CL	Group
Cur3	RMX180	668.2	505.7	882.9	a

Confidence intervals estimated duration moving (H6 - Zone 2)

Model summary

```
Linear mixed model fit by REML. t-tests use Satterthwaite's method ['lmerModLmerTest']
Formula: Estimated_duration_moving_H6_Zone_2 ~ 1 + (1 | Genotype_Zone_1:Plant_Zone_1) +      (1 |
Genotype_Zone_2:Plant_Zone_2)
   Data: data

REML criterion at convergence: 84.5

Scaled residuals: 
    Min      1Q  Median      3Q     Max 
-2.8620 -0.3899  0.1680  0.4969  1.4608 

Random effects:
 Groups                       Name        Variance Std.Dev.
 Genotype_Zone_1:Plant_Zone_1 (Intercept) 0.01911  0.1382  
 Genotype_Zone_2:Plant_Zone_2 (Intercept) 0.00000  0.0000  
 Residual                                 0.50400  0.7099  
Number of obs: 38, groups:  Genotype_Zone_1:Plant_Zone_1, 10; Genotype_Zone_2:Plant_Zone_2, 10

Fixed effects:
            Estimate Std. Error     df t value Pr(>|t|)    
(Intercept)   6.5045     0.1233 9.0434   52.77 1.43e-12 ***
---
Signif. codes:  0 '***' 0.001 '**' 0.01 '*' 0.05 '.' 0.1 ' ' 1
```

Model residuals

Statistic	Value
Sample skewness	-1.277
Sample excess kurtosis	2.209
Passed Shapiro Wilk normality test	No (p-value = 0.001406 < 0.05)

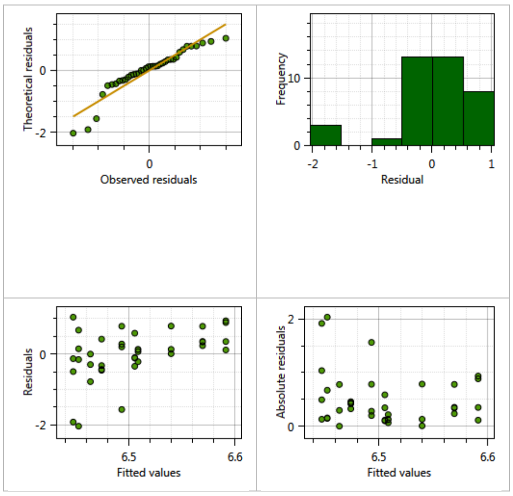

Analysis estimated duration moving (H7 - Zone 1)

**Analysis model**	Linear mixed model fit by REML: Estimated_duration_moving_H7_Zone_1 ~ 1 + (1|Genotype_Zone_1:Plant_Zone_1) + (1|Genotype_Zone_2:Plant_Zone_2)
**Transformation**	Natural logarithm

Confidence intervals estimated duration moving (H7 - Zone 1)

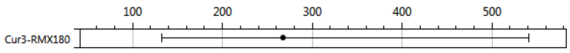

Confidence intervals estimated duration moving (H7 - Zone 1)

Genotype Zone 1	Genotype Zone 2	Mean	Lower 95% CL	Upper 95% CL	Group
Cur3	RMX180	267.4	132.1	541.2	a

Model summary

```
Linear mixed model fit by REML. t-tests use Satterthwaite's method ['lmerModLmerTest']
Formula: Estimated_duration_moving_H7_Zone_1 ~ 1 + (1 | Genotype_Zone_1:Plant_Zone_1) +      (1 |
Genotype_Zone_2:Plant_Zone_2)
   Data: data

REML criterion at convergence: 112.3

Scaled residuals: 
    Min      1Q  Median      3Q     Max 
-3.2022 -0.0309  0.2724  0.5915  0.9168 

Random effects:
 Groups                       Name        Variance Std.Dev.
 Genotype_Zone_2:Plant_Zone_2 (Intercept) 0.000    0.000   
 Genotype_Zone_1:Plant_Zone_1 (Intercept) 0.000    0.000   
 Residual                                 3.308    1.819   
Number of obs: 28, groups:  Genotype_Zone_2:Plant_Zone_2, 10; Genotype_Zone_1:Plant_Zone_1, 9

Fixed effects:
            Estimate Std. Error      df t value Pr(>|t|)    
(Intercept)   5.5886     0.3437 27.0000   16.26  1.8e-15 ***
---
Signif. codes:  0 '***' 0.001 '**' 0.01 '*' 0.05 '.' 0.1 ' ' 1
```

Model residuals

Statistic	Value
Sample skewness	-2.423
Sample excess kurtosis	6.078
Passed Shapiro Wilk normality test	No (p-value = 2.839E-06 < 0.05)

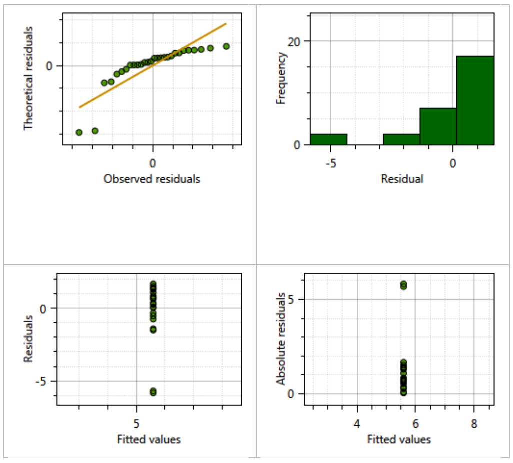

Analysis estimated duration moving (H7 - Zone 2)

**Analysis model**	Linear mixed model fit by REML: Estimated_duration_moving_H7_Zone_2 ~ 1 + (1|Genotype_Zone_1:Plant_Zone_1) + (1|Genotype_Zone_2:Plant_Zone_2)
**Transformation**	Natural logarithm

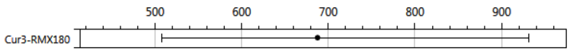

Confidence intervals estimated duration moving (H7 - Zone 2)

Genotype Zone 1	Genotype Zone 2	Mean	Lower 95% CL	Upper 95% CL	Group
Cur3	RMX180	687.6	507.4	931.7	a

Model summary

```
Linear mixed model fit by REML. t-tests use Satterthwaite's method ['lmerModLmerTest']
Formula: Estimated_duration_moving_H7_Zone_2 ~ 1 + (1 | Genotype_Zone_1:Plant_Zone_1) +      (1 |
Genotype_Zone_2:Plant_Zone_2)
   Data: data

REML criterion at convergence: 78.8

Scaled residuals:
     Min       1Q   Median       3Q      Max
-2.71276 -0.69823  0.06712  0.72765  1.50267

Random effects:
 Groups                       Name        Variance Std.Dev.
 Genotype_Zone_1:Plant_Zone_1 (Intercept) 0.05502  0.2346
 Genotype_Zone_2:Plant_Zone_2 (Intercept) 0.01863  0.1365
 Residual                                 0.36443  0.6037
Number of obs: 39, groups:  Genotype_Zone_1:Plant_Zone_1, 10; Genotype_Zone_2:Plant_Zone_2, 10

Fixed effects:
            Estimate Std. Error     df t value Pr(>|t|)
(Intercept)   6.5332     0.1294 7.2549   50.49 1.65e-10 ***
---
Signif. codes:  0 '***' 0.001 '**' 0.01 '*' 0.05 '.' 0.1 ' ' 1
```

Model residuals

Statistic	Value
Sample skewness	-0.594
Sample excess kurtosis	0.4378
Passed Shapiro Wilk normality test	Yes (p-value = 0.1514 > 0.05)

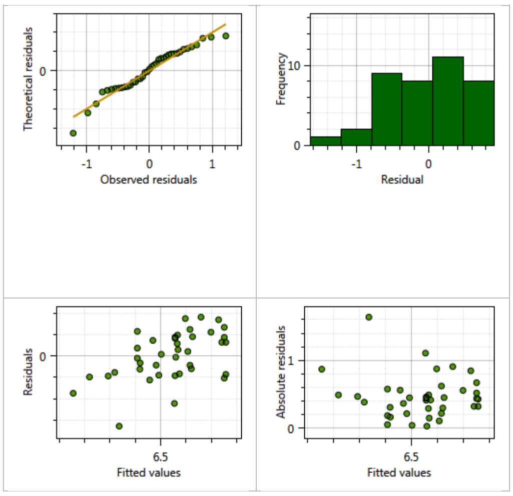

Analysis estimated duration moving H0 (diff. Zone 1 - Zone 2)

**Analysis model**	Generalized linear mixed model with dispersion factor, formula=cbind(Estimated_duration_moving_H0_Zone_1,Estimated_duration_moving_H0_Zone_2) ~ 1 + (1|Genotype_Zone_1:Plant_Zone_1) + (1|Genotype_Zone_2:Plant_Zone_2)
**Transformation**	Logit

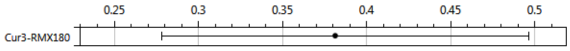

Confidence intervals estimated duration moving H0 (diff. Zone 1 - Zone 2)

Genotype Zone 1	Genotype Zone 2	Mean	Lower 95% CL	Upper 95% CL	Group
Cur3	RMX180	0.3813	0.278	0.4967	a

Model summary

```
Linear mixed model fit by REML. t-tests use Satterthwaite's method ['lmerModLmerTest']
Formula: ziFormula
   Data: data
```

```
Weights: wi

REML criterion at convergence: 114.5

Scaled residuals:
    Min      1Q  Median      3Q     Max
-1.5988 -0.7117 -0.1117  0.5502  1.9715

Random effects:
 Groups                        Name        Variance Std.Dev.
 Genotype_Zone_1:Plant_Zone_1 (Intercept)   0.0000  0.0000
 Genotype_Zone_2:Plant_Zone_2 (Intercept)   0.1602  0.4003
 Residual                                 416.9781 20.4200
Number of obs: 37, groups:  Genotype_Zone_1:Plant_Zone_1, 10; Genotype_Zone_2:Plant_Zone_2, 10

Fixed effects:
            Estimate Std. Error      df t value Pr(>|t|)
(Intercept)  -0.4839     0.2062  8.5097  -2.347   0.0451 *
---
Signif. codes:  0 '***' 0.001 '**' 0.01 '*' 0.05 '.' 0.1 ' ' 1

Dispersion: 20.42
```

Model residuals

Statistic	Value
Sample skewness	0.2618
Sample excess kurtosis	-0.5147
Passed Shapiro Wilk normality test	Yes (p-value = 0.4709 > 0.05)

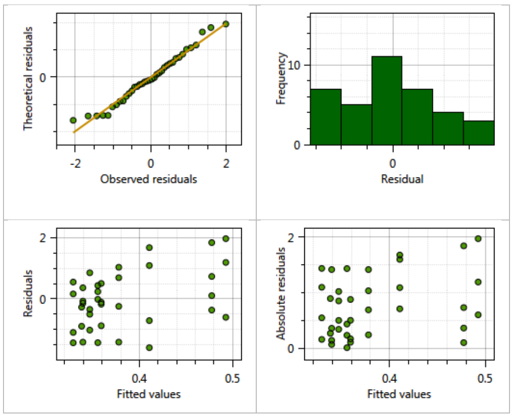

Analysis estimated duration moving H1 (diff. Zone 1 - Zone 2)

**Analysis model**	Generalized linear mixed model with dispersion factor, formula=cbind(Estimated_duration_moving_H1_Zone_1,Estimated_duration_moving_H1_Zone_2) ~ 1 + (1|Genotype_Zone_1:Plant_Zone_1) + (1|Genotype_Zone_2:Plant_Zone_2)
**Transformation**	Logit

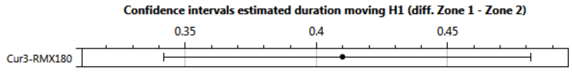

Confidence intervals estimated duration moving H1 (diff. Zone 1 - Zone 2)

Genotype Zone 1	Genotype Zone 2	Mean	Lower 95% CL	Upper 95% CL	Group
Cur3	RMX180	0.4099	0.3417	0.4818	a

Model summary

```
Linear mixed model fit by REML. t-tests use Satterthwaite's method ['lmerModLmerTest']
Formula: ziFormula
   Data: data
Weights: wi

REML criterion at convergence: 98.1

Scaled residuals:
     Min       1Q   Median       3Q      Max
-2.01985 -0.70455 -0.03301  0.73535  2.35570

Random effects:
 Groups                        Name        Variance Std.Dev.
 Genotype_Zone_1:Plant_Zone_1 (Intercept)   0.0     0.00
 Genotype_Zone_2:Plant_Zone_2 (Intercept)   0.0     0.00
 Residual                                 310.1    17.61
Number of obs: 37, groups:  Genotype_Zone_1:Plant_Zone_1, 10; Genotype_Zone_2:Plant_Zone_2, 10

Fixed effects:
            Estimate Std. Error      df t value Pr(>|t|)
(Intercept)  -0.3644     0.1437 36.0000  -2.536   0.0157 *
---
Signif. codes:  0 '***' 0.001 '**' 0.01 '*' 0.05 '.' 0.1 ' ' 1

Dispersion: 17.61
```

Model residuals

Statistic	Value
Sample skewness	0.2559
Sample excess kurtosis	-0.3053
Passed Shapiro Wilk normality test	Yes (p-value = 0.8644 > 0.05)

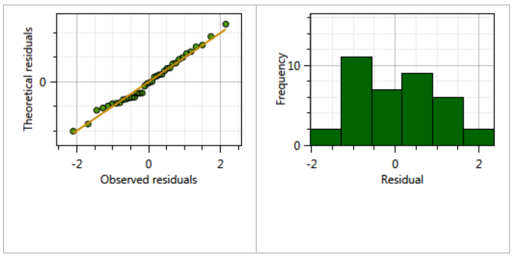

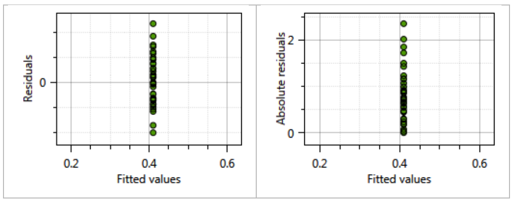

Analysis estimated duration moving H2 (diff. Zone 1 - Zone 2)

**Analysis model**	Generalized linear mixed model with dispersion factor, formula=cbind(Estimated_duration_moving_H2_Zone_1,Estimated_duration_moving_H2_Zone_2) ~ 1 + (1|Genotype_Zone_1:Plant_Zone_1) + (1|Genotype_Zone_2:Plant_Zone_2)
**Transformation**	Logit

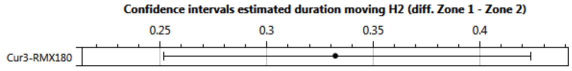

Confidence intervals estimated duration moving H2 (diff. Zone 1 - Zone 2)

Genotype Zone 1	Genotype Zone 2	Mean	Lower 95% CL	Upper 95% CL	Group
Cur3	RMX180	0.3322	0.2515	0.424	a

Model summary

```
Linear mixed model fit by REML. t-tests use Satterthwaite's method ['lmerModLmerTest']
Formula: ziFormula
   Data: data
Weights: wi

REML criterion at convergence: 117.4

Scaled residuals: 
    Min      1Q  Median      3Q     Max 
-1.4223 -0.7000 -0.3715  0.6439  3.2448 

Random effects:
 Groups                      Name        Variance  Std.Dev.
 Genotype_Zone_1:Plant_Zone_1 (Intercept)   0.01426  0.1194 
 Genotype_Zone_2:Plant_Zone_2 (Intercept)   0.00000  0.0000 
 Residual                               304.91028 17.4617 
Number of obs: 39, groups:  Genotype_Zone_1:Plant_Zone_1, 10; Genotype_Zone_2:Plant_Zone_2, 10

Fixed effects:
            Estimate Std. Error      df t value Pr(>|t|)   
(Intercept)  -0.6985     0.1638  6.5995  -4.265  0.00426 **
---
Signif. codes:  0 '***' 0.001 '**' 0.01 '*' 0.05 '.' 0.1 ' ' 1

Dispersion: 17.46
```

Model residuals

Statistic	Value
Sample skewness	1.166
Sample excess kurtosis	1.558
Passed Shapiro Wilk normality test	No (p-value = 0.002728 < 0.05)

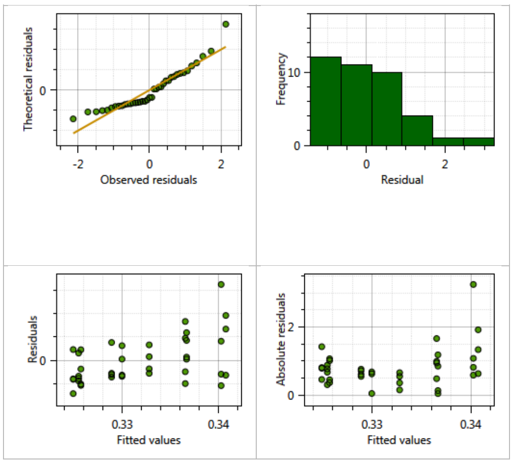

Data points with high residuals

Trial	Arena
Trial 3	7

Analysis estimated duration moving H3 (diff. Zone 1 - Zone 2)

**Analysis model**	Generalized linear mixed model with dispersion factor, formula=cbind(Estimated_duration_moving_H3_Zone_1,Estimated_duration_moving_H3_Zone_2) ~ 1 + (1|Genotype_Zone_1:Plant_Zone_1) + (1|Genotype_Zone_2:Plant_Zone_2)
**Transformation**	Logit

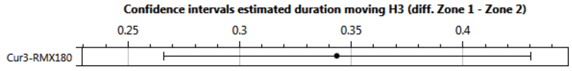

Confidence intervals estimated duration moving H3 (diff. Zone 1 - Zone 2)

Genotype Zone 1	Genotype Zone 2	Mean	Lower 95% CL	Upper 95% CL	Group
Cur3	RMX180	0.3435	0.2658	0.4305	a

Model summary

```
Linear mixed model fit by REML. t-tests use Satterthwaite's method ['lmerModLmerTest']
Formula: ziFormula
   Data: data
Weights: wi

REML criterion at convergence: 112.6

Scaled residuals: 
    Min      1Q  Median      3Q     Max 
-1.7248 -0.8401 -0.0809  0.6312  1.8493 

Random effects:
 Groups                      Name        Variance  Std.Dev.
 Genotype_Zone_1:Plant_Zone_1 (Intercept)   0.00000  0.0000 
 Genotype_Zone_2:Plant_Zone_2 (Intercept)   0.04056  0.2014 
```

```
 Residual                                 281.08054 16.7655
Number of obs: 39, groups:  Genotype_Zone_1:Plant_Zone_1, 10; Genotype_Zone_2:Plant_Zone_2, 10

Fixed effects:
            Estimate Std. Error      df t value Pr(>|t|)
(Intercept)  -0.6478     0.1650  9.9218  -3.925  0.00289 **
---
Signif. codes:  0 '***' 0.001 '**' 0.01 '*' 0.05 '.' 0.1 ' ' 1

Dispersion: 16.77
```

Model residuals

Statistic	Value
Sample skewness	0.2549
Sample excess kurtosis	-0.7679
Passed Shapiro Wilk normality test	Yes (p-value = 0.3462 > 0.05)

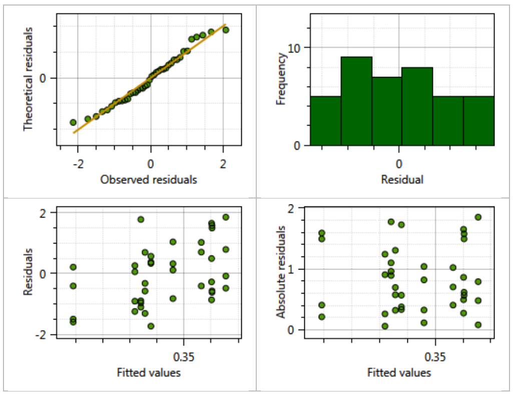

Analysis estimated duration moving H4 (diff. Zone 1 - Zone 2)

**Analysis model**	Generalized linear mixed model with dispersion factor, formula=cbind(Estimated_duration_moving_H4_Zone_1,Estimated_duration_moving_H4_Zone_2) ~ 1 + (1|Genotype_Zone_1:Plant_Zone_1) + (1|Genotype_Zone_2:Plant_Zone_2)
**Transformation**	Logit

Confidence intervals estimated duration moving H4 (diff. Zone 1 - Zone 2)

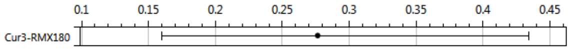

Confidence intervals estimated duration moving H4 (diff. Zone 1 - Zone 2)

Genotype Zone 1	Genotype Zone 2	Mean	Lower 95% CL	Upper 95% CL	Group
Cur3	RMX180	0.2764	0.1596	0.4344	a

Model summary

```
Linear mixed model fit by REML. t-tests use Satterthwaite's method ['lmerModLmerTest']
Formula: ziFormula
   Data: data
Weights: wi

REML criterion at convergence: 131.7

Scaled residuals:
    Min      1Q  Median      3Q     Max
-1.4930 -0.7129 -0.1462  0.7927  1.6369

Random effects:
 Groups                       Name        Variance Std.Dev.
 Genotype_Zone_1:Plant_Zone_1 (Intercept)   0.2548  0.5047
 Genotype_Zone_2:Plant_Zone_2 (Intercept)   0.3871  0.6222
 Residual                                 254.3667 15.9489
Number of obs: 39, groups:  Genotype_Zone_1:Plant_Zone_1, 10; Genotype_Zone_2:Plant_Zone_2, 10

Fixed effects:
            Estimate Std. Error      df t value Pr(>|t|)
(Intercept)  -0.9624     0.3063  8.5424  -3.142   0.0127 *
---
Signif. codes:  0 '***' 0.001 '**' 0.01 '*' 0.05 '.' 0.1 ' ' 1

Dispersion: 15.95
```

Model residuals

Statistic	Value
Sample skewness	0.3085
Sample excess kurtosis	-1.189
Passed Shapiro Wilk normality test	No (p-value = 0.03202 < 0.05)

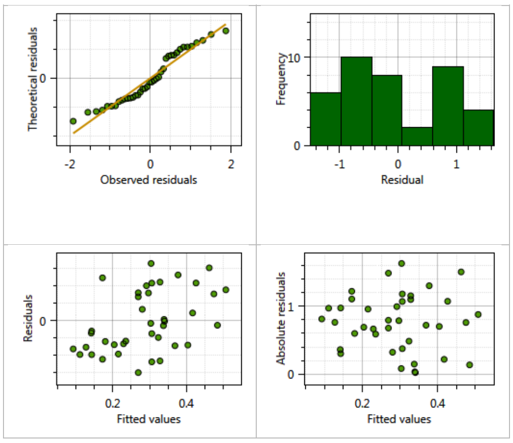

Analysis estimated duration moving H5 (diff. Zone 1 - Zone 2)

**Analysis model**	Generalized linear mixed model with dispersion factor, formula=cbind(Estimated_duration_moving_H5_Zone_1,Estimated_duration_moving_H5_Zone_2) ~ 1 + (1|Genotype_Zone_1:Plant_Zone_1) + (1|Genotype_Zone_2:Plant_Zone_2)
**Transformation**	Logit

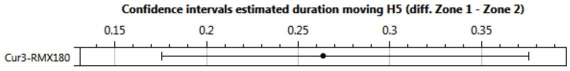

Confidence intervals estimated duration moving H5 (diff. Zone 1 - Zone 2)

Genotype Zone 1	Genotype Zone 2	Mean	Lower 95% CL	Upper 95% CL	Group
Cur3	RMX180	0.2636	0.1754	0.3759	a

Model summary

```
Linear mixed model fit by REML. t-tests use Satterthwaite's method ['lmerModLmerTest']
Formula: ziFormula
   Data: data
Weights: wi

REML criterion at convergence: 128.4

Scaled residuals:
    Min      1Q  Median      3Q     Max
-1.3429 -0.8668 -0.1809  0.9336  1.9980

Random effects:
 Groups                        Name        Variance Std.Dev.
 Genotype_Zone_1:Plant_Zone_1 (Intercept)   0.0000  0.0000
 Genotype_Zone_2:Plant_Zone_2 (Intercept)   0.2149  0.4635
 Residual                                 298.1035 17.2657
Number of obs: 39, groups:  Genotype_Zone_1:Plant_Zone_1, 10; Genotype_Zone_2:Plant_Zone_2, 10

Fixed effects:
            Estimate Std. Error      df t value Pr(>|t|)
(Intercept)  -1.0273     0.2328  9.7765  -4.412  0.00138 **
---
Signif. codes:  0 '***' 0.001 '**' 0.01 '*' 0.05 '.' 0.1 ' ' 1

Dispersion: 17.27
```

Model residuals

Statistic	Value
Sample skewness	0.4216
Sample excess kurtosis	-0.9675
Passed Shapiro Wilk normality test	No (p-value = 0.02295 < 0.05)

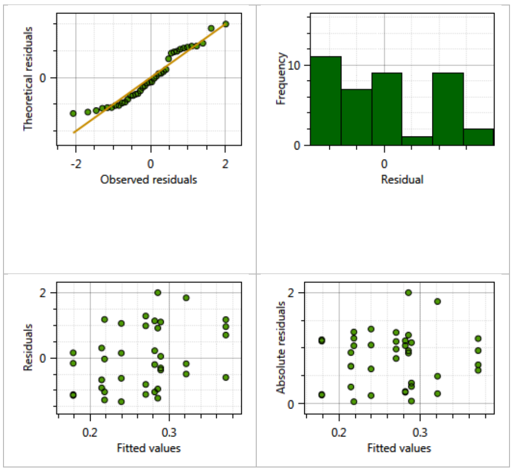

Analysis estimated duration moving H6 (diff. Zone 1 - Zone 2)

Analysis model	Generalized linear mixed model with dispersion factor, formula=cbind(Estimated_duration_moving_H6_Zone_1,Estimated_duration_moving_H6_Zone_2) ~ 1 + (1|Genotype_Zone_1:Plant_Zone_1) + (1|Genotype_Zone_2:Plant_Zone_2)
Transformation	Logit

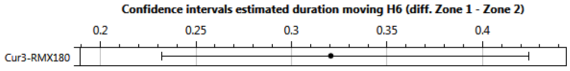

Confidence intervals estimated duration moving H6 (diff. Zone 1 - Zone 2)

Genotype Zone 1	Genotype Zone 2	Mean	Lower 95% CL	Upper 95% CL	Group
Cur3	RMX180	0.3206	0.2319	0.4243	a

Model summary

```
Linear mixed model fit by REML. t-tests use Satterthwaite's method ['lmerModLmerTest']
Formula: ziFormula
   Data: data
Weights: wi

REML criterion at convergence: 132.3

Scaled residuals:
    Min      1Q  Median      3Q     Max
-1.5254 -0.7821 -0.0025  0.8627  2.0517

Random effects:
 Groups                        Name        Variance  Std.Dev.
 Genotype_Zone_1:Plant_Zone_1 (Intercept)   0.04419  0.2102
 Genotype_Zone_2:Plant_Zone_2 (Intercept)   0.00000  0.0000
 Residual                                 363.56810 19.0675
Number of obs: 39, groups:  Genotype_Zone_1:Plant_Zone_1, 10; Genotype_Zone_2:Plant_Zone_2, 10

Fixed effects:
            Estimate Std. Error      df t value Pr(>|t|)
(Intercept)  -0.7512     0.2034 11.2918  -3.694  0.00339 **
---
Signif. codes:  0 '***' 0.001 '**' 0.01 '*' 0.05 '.' 0.1 ' ' 1

Dispersion: 19.07
```

Model residuals

Statistic	Value
Sample skewness	0.1649
Sample excess kurtosis	-1.16
Passed Shapiro Wilk normality test	Yes (p-value = 0.05542 > 0.05)

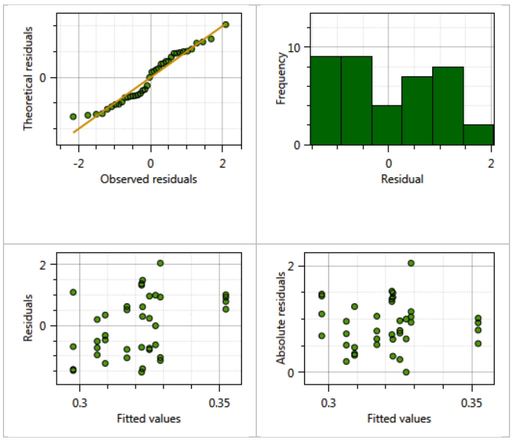

Analysis estimated duration moving H7 (diff. Zone 1 - Zone 2)

**Analysis model**	Generalized linear mixed model with dispersion factor, formula=cbind(Estimated_duration_moving_H7_Zone_1,Estimated_duration_moving_H7_Zone_2) ~ 1 + (1|Genotype_Zone_1:Plant_Zone_1) + (1|Genotype_Zone_2:Plant_Zone_2)
**Transformation**	Logit

Confidence intervals estimated duration moving H7 (diff. Zone 1 - Zone 2)

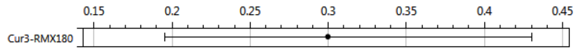

Confidence intervals estimated duration moving H7 (diff. Zone 1 - Zone 2)

Genotype Zone 1	Genotype Zone 2	Mean	Lower 95% CL	Upper 95% CL	Group
Cur3	RMX180	0.2997	0.195	0.4304	a

Model summary

```
Linear mixed model fit by REML. t-tests use Satterthwaite's method ['lmerModLmerTest']
Formula: ziFormula
   Data: data
Weights: wi

REML criterion at convergence: 133.8

Scaled residuals:
    Min      1Q  Median      3Q     Max
-1.3894 -0.8097 -0.1488  0.5466  1.7670

Random effects:
 Groups                      Name        Variance  Std.Dev.
 Genotype_Zone_1:Plant_Zone_1 (Intercept)   0.18141  0.4259
 Genotype_Zone_2:Plant_Zone_2 (Intercept)   0.06732  0.2595
 Residual                              328.31874 18.1196
Number of obs: 39, groups:  Genotype_Zone_1:Plant_Zone_1, 10; Genotype_Zone_2:Plant_Zone_2, 10

Fixed effects:
            Estimate Std. Error      df t value Pr(>|t|)
(Intercept)  -0.8488     0.2459  7.8554  -3.452  0.00891 **
---
Signif. codes:  0 '***' 0.001 '**' 0.01 '*' 0.05 '.' 0.1 ' ' 1

Dispersion: 18.12
```

Model residuals

Statistic	Value
Sample skewness	0.4923
Sample excess kurtosis	-0.777
Passed Shapiro Wilk normality test	No (p-value = 0.03432 < 0.05)

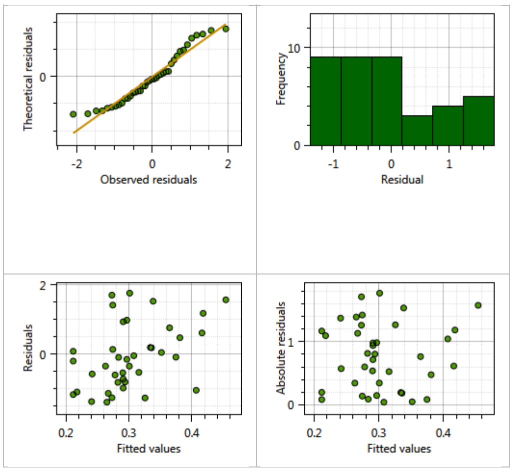

Estimated duration moving per zone per short/medium/long

Selected zones	Zone 1, Zone 2
Event duration categories	duration < 2, 2 <= duration < 5, duration >= 5
Data transformation	Natural logarithm
Analysis	Zone difference analysis

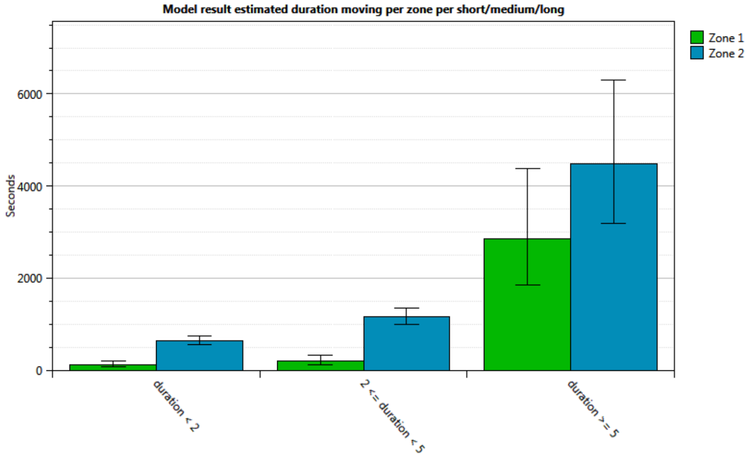

Results difference tests Zone 1 - Zone 2: p values and 95% confidence intervals of the difference on the transformed scale for each statistic.

Behaviour statistic	Cur3-RMX180	Remark
Estimated duration moving duration < 2 (diff. Zone 1 - Zone 2)	p=0.000274***<br>[-1.89, -0.938]	
Estimated duration moving 2 <= duration < 5 (diff. Zone 1 - Zone 2)	p=2.58E-05****<br>[-2.01, -1.11]	CR
Estimated duration moving duration >= 5 (diff. Zone 1 - Zone 2)	p=0.00275**<br>[-0.79, -0.227]	

CR = Check residuals

The model predictions and 95% confidence intervals for each statistic.

Statistic	Cur3-RMX180	Remark
Estimated duration moving (duration < 2 - Zone 1)	125<br>[80.1, 196]	CR
Estimated duration moving (2 <= duration < 5 - Zone 1)	204<br>[124, 334]	CR
Estimated duration moving (duration >= 5 - Zone 1)	2.85E+03<br>[1.85E+03, 4.38E+03]	CR
Estimated duration moving (duration < 2 - Zone 2)	641<br>[556, 739]	
Estimated duration moving (2 <= duration < 5 - Zone 2)	1.16E+03<br>[989, 1.35E+03]	
Estimated duration moving (duration >= 5 - Zone 2)	4.48E+03<br>[3.19E+03, 6.31E+03]	CR

CR = Check residuals

Data summary

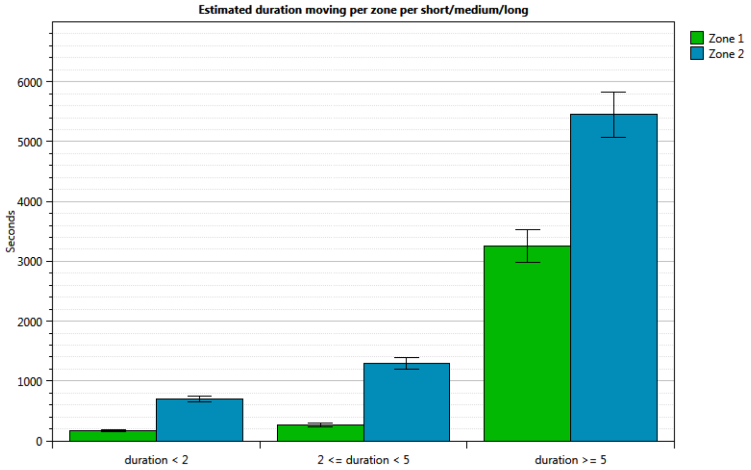

Genotype Zone 1	Genotype Zone 2	Genotype Zone 3	Mean duration < 2 - Zone 1	StdErr duration < 2 - Zone 1	Mean 2 <= duration < 5 - Zone 1	StdErr 2 <= duration < 5 - Zone 1	Mean duration >= 5 - Zone 1	StdErr duration >= 5 - Zone 1	Mean duration < 2 - Zone 2	StdErr duration < 2 - Zone 2	Mean 2 <= duration < 5 - Zone 2	StdErr 2 <= duration < 5 - Zone 2	Mean duration >= 5 - Zone 2	StdErr duration >= 5 - Zone 2
Cur3	RMX180	Neutral	170.64	19.31	272.33	32.92	3258.74	275.88	697.43	43.71	1287.53	97.01	5452.5	375.31

Analysis estimated duration moving (duration < 2 - Zone 1)

**Analysis model**	Linear mixed model fit by REML: Estimated_duration_moving_duration_2_Zone_1 ~ 1 + (1|Genotype_Zone_1:Plant_Zone_1) + (1|Genotype_Zone_2:Plant_Zone_2)
**Transformation**	Natural logarithm

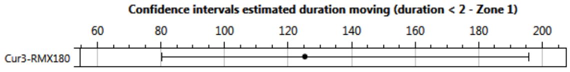

Confidence intervals estimated duration moving (duration < 2 - Zone 1)

Genotype Zone 1	Genotype Zone 2	Mean	Lower 95% CL	Upper 95% CL	Group
Cur3	RMX180	125.3	80.13	195.8	a

Model summary

```
Linear mixed model fit by REML. t-tests use Satterthwaite's method ['lmerModLmerTest']
Formula: Estimated_duration_moving_duration_2_Zone_1 ~ 1 + (1 | Genotype_Zone_1:Plant_Zone_1) +      (1 |
Genotype_Zone_2:Plant_Zone_2)
   Data: data

REML criterion at convergence: 115.3

Scaled residuals: 
    Min      1Q  Median      3Q     Max 
-4.2841 -0.2283  0.2514  0.4731  1.1324 

Random effects:
 Groups                       Name        Variance Std.Dev.
 Genotype_Zone_1:Plant_Zone_1 (Intercept) 0.0000   0.0000  
 Genotype_Zone_2:Plant_Zone_2 (Intercept) 0.1029   0.3207  
 Residual                                 1.1131   1.0550  
Number of obs: 38, groups:  Genotype_Zone_1:Plant_Zone_1, 10; Genotype_Zone_2:Plant_Zone_2, 10
```

```
Fixed effects:
            Estimate Std. Error     df t value Pr(>|t|)
(Intercept)   4.8305     0.1992 9.5218   24.25    7e-10 ***
---
Signif. codes:  0 '***' 0.001 '**' 0.01 '*' 0.05 '.' 0.1 ' ' 1
```

Model residuals

Statistic	Value
Sample skewness	-2.627
Sample excess kurtosis	9.838
Passed Shapiro Wilk normality test	No (p-value = 1.897E-06 < 0.05)

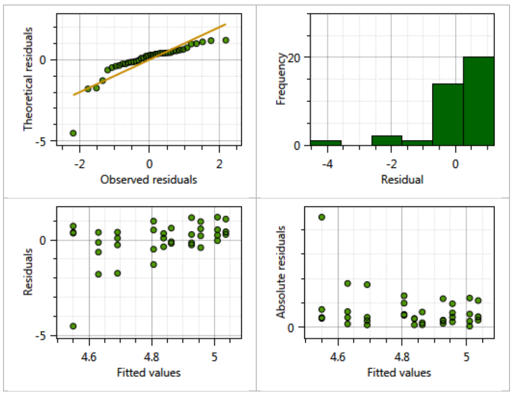

Analysis estimated duration moving (2 <= duration < 5 - Zone 1)

Analysis model	Linear mixed model fit by REML: Estimated_duration_moving_2_duration_5_Zone_1 ~ 1 + (1|Genotype_Zone_1:Plant_Zone_1) + (1|Genotype_Zone_2:Plant_Zone_2)
Transformation	Natural logarithm

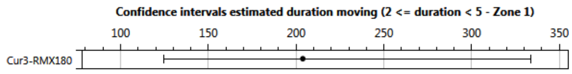

Confidence intervals estimated duration moving (2 <= duration < 5 - Zone 1)

Genotype Zone 1	Genotype Zone 2	Mean	Lower 95% CL	Upper 95% CL	Group
Cur3	RMX180	203.8	124.4	333.8	a

Model summary

```
Linear mixed model fit by REML. t-tests use Satterthwaite's method ['lmerModLmerTest']
Formula: Estimated_duration_moving_2_duration_5_Zone_1 ~ 1 + (1 | Genotype_Zone_1:Plant_Zone_1) +      (1
| Genotype_Zone_2:Plant_Zone_2)
   Data: data

REML criterion at convergence: 107.7

Scaled residuals: 
    Min      1Q  Median      3Q     Max 
-4.0944 -0.1895  0.2030  0.5169  1.3479 

Random effects:
 Groups                       Name        Variance Std.Dev.
 Genotype_Zone_1:Plant_Zone_1 (Intercept) 0.15549  0.3943  
 Genotype_Zone_2:Plant_Zone_2 (Intercept) 0.04729  0.2175  
 Residual                                 0.81978  0.9054  
Number of obs: 38, groups:  Genotype_Zone_1:Plant_Zone_1, 10; Genotype_Zone_2:Plant_Zone_2, 10

Fixed effects:
            Estimate Std. Error    df t value Pr(>|t|)    
(Intercept)    5.317      0.205 6.428   25.94 9.48e-08 ***
---
Signif. codes:  0 '***' 0.001 '**' 0.01 '*' 0.05 '.' 0.1 ' ' 1
```

Model residuals

Statistic	Value
Sample skewness	-2.456
Sample excess kurtosis	9.445
Passed Shapiro Wilk normality test	No (p-value = 1.161E-05 < 0.05)

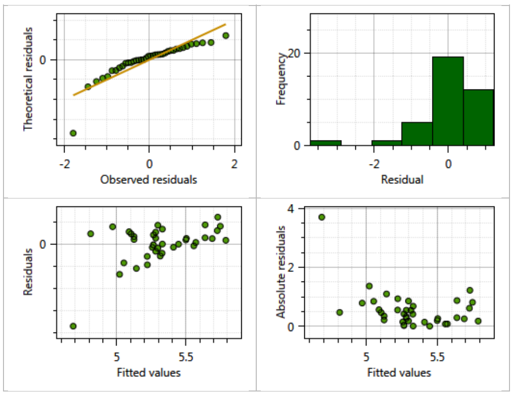

Analysis estimated duration moving (duration >= 5 - Zone 1)

Analysis model	Linear mixed model fit by REML: Estimated_duration_moving_duration_5_Zone_1 ~ 1 + (1|Genotype_Zone_1:Plant_Zone_1) + (1|Genotype_Zone_2:Plant_Zone_2)
Transformation	Natural logarithm

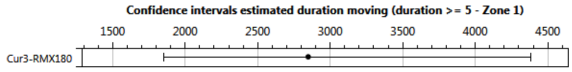

Confidence intervals estimated duration moving (duration >= 5 - Zone 1)

Genotype Zone 1	Genotype Zone 2	Mean	Lower 95% CL	Upper 95% CL	Group
Cur3	RMX180	2848	1850	4385	a

Model summary

```
Linear mixed model fit by REML. t-tests use Satterthwaite's method ['lmerModLmerTest']
Formula: Estimated_duration_moving_duration_5_Zone_1 ~ 1 + (1 | Genotype_Zone_1:Plant_Zone_1) +      (1 |
Genotype_Zone_2:Plant_Zone_2)
   Data: data

REML criterion at convergence: 92.5

Scaled residuals:
    Min      1Q  Median      3Q     Max
-4.5892 -0.1771  0.1908  0.4718  1.0752

Random effects:
 Groups                       Name        Variance Std.Dev.
 Genotype_Zone_1:Plant_Zone_1 (Intercept) 0.05846  0.2418
 Genotype_Zone_2:Plant_Zone_2 (Intercept) 0.06561  0.2561
 Residual                                 0.59186  0.7693
Number of obs: 37, groups:  Genotype_Zone_1:Plant_Zone_1, 10; Genotype_Zone_2:Plant_Zone_2, 10

Fixed effects:
            Estimate Std. Error     df t value Pr(>|t|)
(Intercept)   7.9544     0.1692 5.1397   47.01  5.7e-08 ***
---
Signif. codes:  0 '***' 0.001 '**' 0.01 '*' 0.05 '.' 0.1 ' ' 1
```

Model residuals

Statistic	Value
Sample skewness	-3.451
Sample excess kurtosis	16.26
Passed Shapiro Wilk normality test	No (p-value = 1.132E-07 < 0.05)

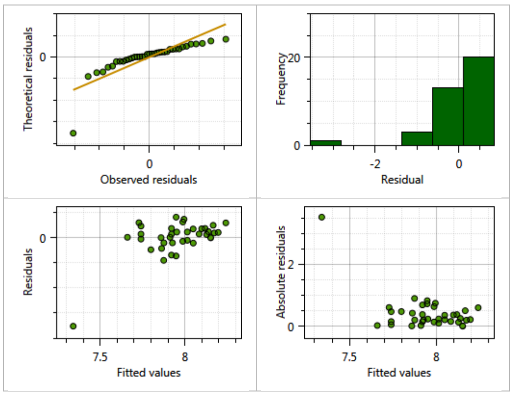

Analysis estimated duration moving (duration < 2 - Zone 2)

Analysis model	Linear mixed model fit by REML: Estimated_duration_moving_duration_2_Zone_2 ~ 1 + (1|Genotype_Zone_1:Plant_Zone_1) + (1|Genotype_Zone_2:Plant_Zone_2)
Transformation	Natural logarithm

Confidence intervals estimated duration moving (duration < 2 - Zone 2)

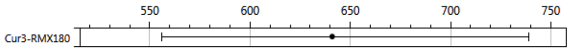

Confidence intervals estimated duration moving (duration < 2 - Zone 2)

Genotype Zone 1	Genotype Zone 2	Mean	Lower 95% CL	Upper 95% CL	Group
Cur3	RMX180	641	555.8	739.2	a

Model summary

```
Linear mixed model fit by REML. t-tests use Satterthwaite's method ['lmerModLmerTest']
Formula: Estimated_duration_moving_duration_2_Zone_2 ~ 1 + (1 | Genotype_Zone_1:Plant_Zone_1) +      (1 |
Genotype_Zone_2:Plant_Zone_2)
   Data: data

REML criterion at convergence: 49.1

Scaled residuals:
    Min      1Q  Median      3Q     Max
-3.2193 -0.6166  0.1056  0.7587  1.6559

Random effects:
 Groups                       Name        Variance Std.Dev.
 Genotype_Zone_1:Plant_Zone_1 (Intercept) 0.0000   0.0000
 Genotype_Zone_2:Plant_Zone_2 (Intercept) 0.0000   0.0000
 Residual                                 0.1933   0.4397
Number of obs: 39, groups:  Genotype_Zone_1:Plant_Zone_1, 10; Genotype_Zone_2:Plant_Zone_2, 10

Fixed effects:
            Estimate Std. Error       df t value Pr(>|t|)
(Intercept)  6.46297    0.07041 38.00000   91.79   <2e-16 ***
---
Signif. codes:  0 '***' 0.001 '**' 0.01 '*' 0.05 '.' 0.1 ' ' 1
```

Model residuals

Statistic	Value
Sample skewness	-0.862
Sample excess kurtosis	1.488
Passed Shapiro Wilk normality test	Yes (p-value = 0.06851 > 0.05)

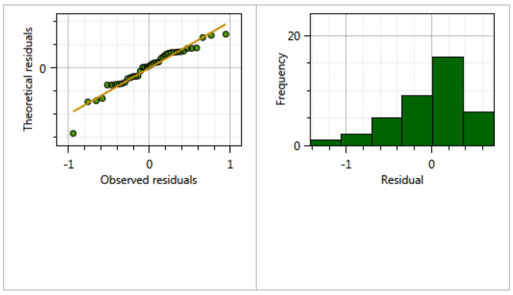

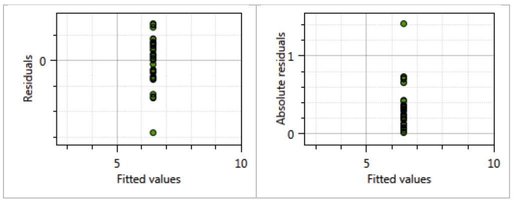

Analysis estimated duration moving (2 <= duration < 5 - Zone 2)

**Analysis model**	Linear mixed model fit by REML: Estimated_duration_moving_2_duration_5_Zone_2 ~ 1 + (1|Genotype_Zone_1:Plant_Zone_1) + (1|Genotype_Zone_2:Plant_Zone_2)
**Transformation**	Natural logarithm

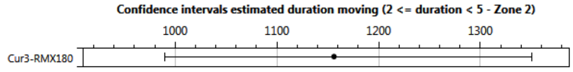

Confidence intervals estimated duration moving (2 <= duration < 5 - Zone 2)

Genotype Zone 1	Genotype Zone 2	Mean	Lower 95% CL	Upper 95% CL	Group
Cur3	RMX180	1156	989.5	1351	a

Model summary

```
Linear mixed model fit by REML. t-tests use Satterthwaite's method ['lmerModLmerTest']
Formula: Estimated_duration_moving_2_duration_5_Zone_2 ~ 1 + (1 | Genotype_Zone_1:Plant_Zone_1) +      (1
| Genotype_Zone_2:Plant_Zone_2)
   Data: data

REML criterion at convergence: 55.7

Scaled residuals:
     Min       1Q   Median       3Q      Max
-2.69428 -0.58265 -0.06474  0.76099  2.02507

Random effects:
 Groups                       Name        Variance  Std.Dev.
 Genotype_Zone_1:Plant_Zone_1 (Intercept) 0.000e+00 0.000e+00
 Genotype_Zone_2:Plant_Zone_2 (Intercept) 3.643e-19 6.036e-10
 Residual                                 2.306e-01 4.802e-01
Number of obs: 39, groups:  Genotype_Zone_1:Plant_Zone_1, 10; Genotype_Zone_2:Plant_Zone_2, 10

Fixed effects:
            Estimate Std. Error       df t value Pr(>|t|)
(Intercept)  7.05281    0.07689 38.00000   91.73   <2e-16 ***
---
Signif. codes:  0 '***' 0.001 '**' 0.01 '*' 0.05 '.' 0.1 ' ' 1
```

Model residuals

Statistic	Value
Sample skewness	-0.2688
Sample excess kurtosis	0.1722
Passed Shapiro Wilk normality test	Yes (p-value = 0.9146 > 0.05)

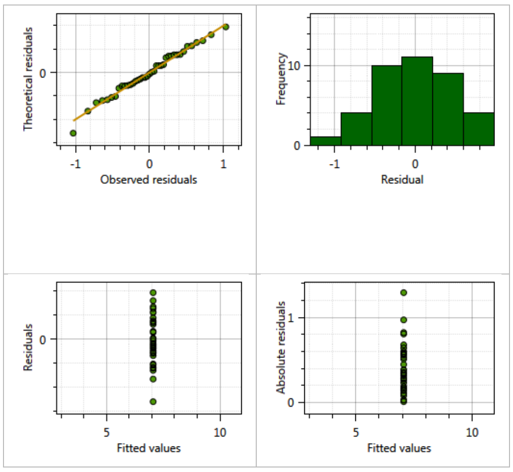

Analysis estimated duration moving (duration >= 5 - Zone 2)

**Analysis model**	Linear mixed model fit by REML: Estimated_duration_moving_duration_5_Zone_2 ~ 1 + (1|Genotype_Zone_1:Plant_Zone_1) + (1|Genotype_Zone_2:Plant_Zone_2)
**Transformation**	Natural logarithm

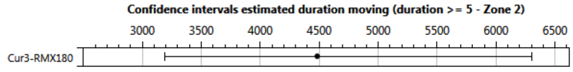

Confidence intervals estimated duration moving (duration >= 5 - Zone 2)

Genotype Zone 1	Genotype Zone 2	Mean	Lower 95% CL	Upper 95% CL	Group
Cur3	RMX180	4483	3187	6305	a

Model summary

```
Linear mixed model fit by REML. t-tests use Satterthwaite's method ['lmerModLmerTest']
Formula: Estimated_duration_moving_duration_5_Zone_2 ~ 1 + (1 | Genotype_Zone_1:Plant_Zone_1) +      (1 |
Genotype_Zone_2:Plant_Zone_2)
   Data: data

REML criterion at convergence: 96.6

Scaled residuals:
    Min      1Q  Median      3Q     Max
-3.4206 -0.0870  0.2667  0.4905  1.0480

Random effects:
 Groups                       Name        Variance Std.Dev.
 Genotype_Zone_1:Plant_Zone_1 (Intercept) 0.00000  0.0000
 Genotype_Zone_2:Plant_Zone_2 (Intercept) 0.05754  0.2399
 Residual                                 0.62777  0.7923
Number of obs: 39, groups:  Genotype_Zone_1:Plant_Zone_1, 10; Genotype_Zone_2:Plant_Zone_2, 10

Fixed effects:
            Estimate Std. Error     df t value Pr(>|t|)
(Intercept)   8.4080     0.1479 7.9886   56.84 1.05e-11 ***
---
Signif. codes:  0 '***' 0.001 '**' 0.01 '*' 0.05 '.' 0.1 ' ' 1
```

Model residuals

Statistic	Value
Sample skewness	-2.636
Sample excess kurtosis	6.83
Passed Shapiro Wilk normality test	No (p-value = 1.853E-08 < 0.05)

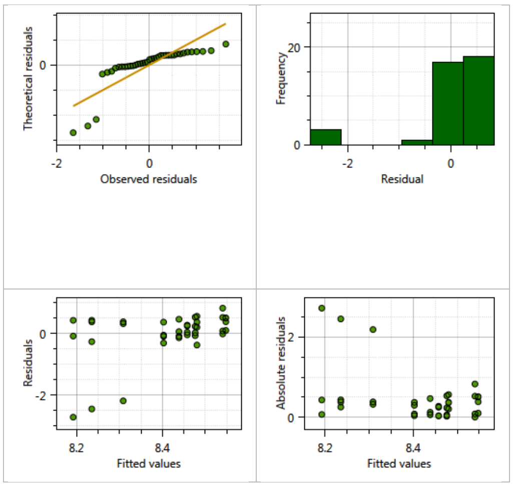

Analysis estimated duration moving duration < 2 (diff. Zone 1 - Zone 2)

**Analysis model**	Generalized linear mixed model with dispersion factor, formula=cbind(Estimated_duration_moving_duration_2_Zone_1,Estimated_duration_moving_duration_2_Zone_2) ~ 1 + (1|Genotype_Zone_1:Plant_Zone_1) + (1|Genotype_Zone_2:Plant_Zone_2)
**Transformation**	Logit

Confidence intervals estimated duration moving duration < 2 (diff. Zone 1 - Zone 2)

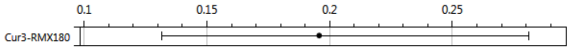

Confidence intervals estimated duration moving duration < 2 (diff. Zone 1 - Zone 2)

Genotype Zone 1	Genotype Zone 2	Mean	Lower 95% CL	Upper 95% CL	Group
Cur3	RMX180	0.1957	0.1314	0.2813	a

Model summary

```
Linear mixed model fit by REML. t-tests use Satterthwaite's method ['lmerModLmerTest']
Formula: ziFormula
   Data: data
Weights: wi

REML criterion at convergence: 112.9

Scaled residuals:
     Min       1Q   Median       3Q      Max
-1.50375 -0.54535  0.02918  0.48328  2.38217

Random effects:
 Groups                      Name        Variance  Std.Dev.
 Genotype_Zone_1:Plant_Zone_1 (Intercept)   0.04129  0.2032
 Genotype_Zone_2:Plant_Zone_2 (Intercept)   0.11966  0.3459
 Residual                              119.61625 10.9369
Number of obs: 39, groups:  Genotype_Zone_1:Plant_Zone_1, 10; Genotype_Zone_2:Plant_Zone_2, 10

Fixed effects:
            Estimate Std. Error      df t value Pr(>|t|)
(Intercept)  -1.4133     0.1972  6.4182  -7.166 0.000274 ***
---
Signif. codes:  0 '***' 0.001 '**' 0.01 '*' 0.05 '.' 0.1 ' ' 1

Dispersion: 10.94
```

Model residuals

Statistic	Value
Sample skewness	0.651
Sample excess kurtosis	0.1442
Passed Shapiro Wilk normality test	Yes (p-value = 0.1375 > 0.05)

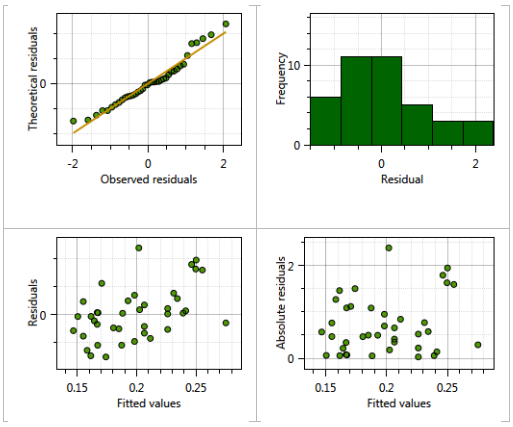

Analysis estimated duration moving 2 <= duration < 5 (diff. Zone 1 - Zone 2)

**Analysis model**	Generalized linear mixed model with dispersion factor, formula=cbind(Estimated_duration_moving_2_duration_5_Zone_1,Estimated_duration_moving_2_duration_5_Zone_2) ~ 1 + (1|Genotype_Zone_1:Plant_Zone_1) + (1|Genotype_Zone_2:Plant_Zone_2)
**Transformation**	Logit

Confidence intervals estimated duration moving 2 <= duration < 5 (diff. Zone 1 - Zone 2)

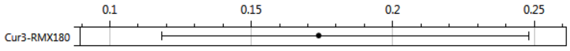

Confidence intervals estimated duration moving 2 <= duration < 5 (diff. Zone 1 - Zone 2)

Genotype Zone 1	Genotype Zone 2	Mean	Lower 95% CL	Upper 95% CL	Group
Cur3	RMX180	0.1738	0.1182	0.2482	a

Model summary

```
Linear mixed model fit by REML. t-tests use Satterthwaite's method ['lmerModLmerTest']
Formula: ziFormula
   Data: data
Weights: wi

REML criterion at convergence: 108.9

Scaled residuals:
    Min      1Q  Median      3Q     Max
-1.3420 -0.7072 -0.1197  0.5343  1.8556

Random effects:
 Groups                      Name        Variance  Std.Dev.
 Genotype_Zone_1:Plant_Zone_1 (Intercept)   0.07523  0.2743
 Genotype_Zone_2:Plant_Zone_2 (Intercept)   0.13218  0.3636
 Residual                               158.71001 12.5980
Number of obs: 39, groups:  Genotype_Zone_1:Plant_Zone_1, 10; Genotype_Zone_2:Plant_Zone_2, 10

Fixed effects:
            Estimate Std. Error      df t value Pr(>|t|)
(Intercept)  -1.5588     0.1993  9.0460   -7.82 2.58e-05 ***
---
Signif. codes:  0 '***' 0.001 '**' 0.01 '*' 0.05 '.' 0.1 ' ' 1

Dispersion: 12.6
```

Model residuals

Statistic	Value
Sample skewness	0.5034
Sample excess kurtosis	-0.7932
Passed Shapiro Wilk normality test	No (p-value = 0.03286 < 0.05)

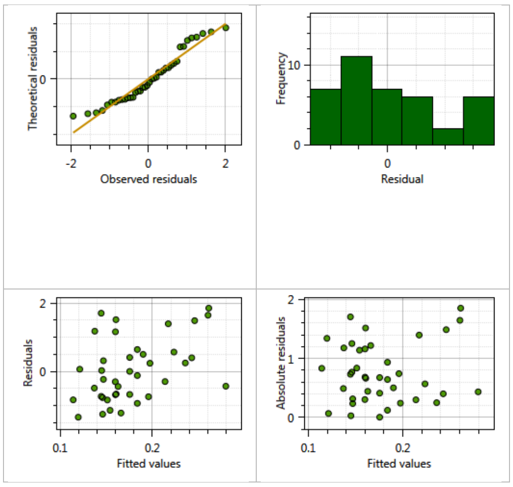

Analysis estimated duration moving duration >= 5 (diff. Zone 1 - Zone 2)

**Analysis model**	Generalized linear mixed model with dispersion factor, formula=cbind(Estimated_duration_moving_duration_5_Zone_1,Estimated_duration_moving_duration_5_Zone_2) ~ 1 + (1|Genotype_Zone_1:Plant_Zone_1) + (1|Genotype_Zone_2:Plant_Zone_2)
**Transformation**	Logit

Confidence intervals estimated duration moving duration >= 5 (diff. Zone 1 - Zone 2)

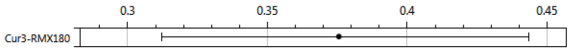

Confidence intervals estimated duration moving duration >= 5 (diff. Zone 1 - Zone 2)

Genotype Zone 1	Genotype Zone 2	Mean	Lower 95% CL	Upper 95% CL	Group
Cur3	RMX180	0.3755	0.3121	0.4436	a

Model summary

```
Linear mixed model fit by REML. t-tests use Satterthwaite's method ['lmerModLmerTest']
Formula: ziFormula
   Data: data
Weights: wi

REML criterion at convergence: 79.8

Scaled residuals:
     Min       1Q   Median       3Q      Max
-1.70654 -0.73715 -0.08766  0.68778  1.68656

Random effects:
 Groups                      Name        Variance  Std.Dev.
 Genotype_Zone_1:Plant_Zone_1 (Intercept)   0.01082  0.1040
 Genotype_Zone_2:Plant_Zone_2 (Intercept)   0.06497  0.2549
 Residual                               608.48639 24.6675
Number of obs: 39, groups:  Genotype_Zone_1:Plant_Zone_1, 10; Genotype_Zone_2:Plant_Zone_2, 10

Fixed effects:
            Estimate Std. Error      df t value Pr(>|t|)
(Intercept)  -0.5085     0.1247  9.0155  -4.079  0.00275 **
---
Signif. codes:  0 '***' 0.001 '**' 0.01 '*' 0.05 '.' 0.1 ' ' 1

Dispersion: 24.67
```

Model residuals

Statistic	Value
Sample skewness	0.1055
Sample excess kurtosis	-0.9616
Passed Shapiro Wilk normality test	Yes (p-value = 0.4312 > 0.05)

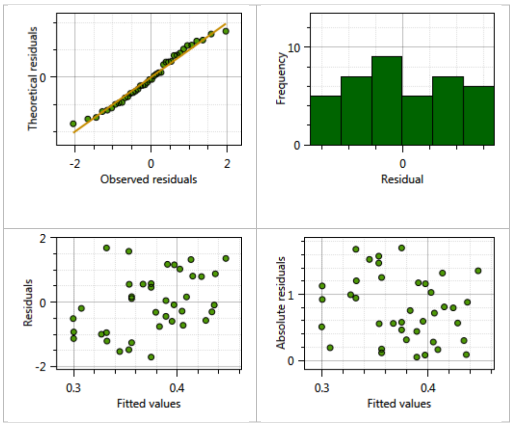

Estimated duration moving per zone per slow/medium/fast

Selected zones	Zone 1, Zone 2
Velocity categories	velocity < 0.025, 0.025 <= velocity < 0.075, velocity >= 0.075
Data transformation	Natural logarithm
Analysis	Zone difference analysis

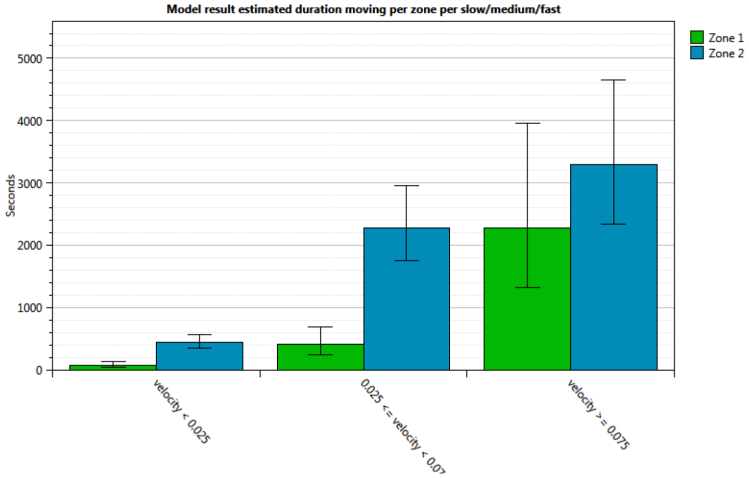

Results difference tests Zone 1 - Zone 2: p values and 95% confidence intervals of the difference on the transformed scale for each statistic.

Behaviour statistic	Cur3-RMX180
Estimated duration moving velocity < 0.025 (diff. Zone 1 - Zone 2)	p=0.000244***<br>[-2.09, -1.05]
Estimated duration moving 0.025 <= velocity < 0.075 (diff. Zone 1 - Zone 2)	p=5.86E-05****<br>[-2.26, -1.1]
Estimated duration moving velocity >= 0.075 (diff. Zone 1 - Zone 2)	p=0.0102*<br>[-0.477, -0.0892]

The model predictions and 95% confidence intervals for each statistic.

Statistic	Cur3-RMX180	Remark
Estimated duration moving (velocity < 0.025 - Zone 1)	74.8<br>[43.7, 128]	CR
Estimated duration moving (0.025 <= velocity < 0.075 - Zone 1)	407<br>[241, 686]	
Estimated duration moving (velocity >= 0.075 - Zone 1)	2.28E+03<br>[1.32E+03, 3.95E+03]	CR
Estimated duration moving (velocity < 0.025 - Zone 2)	444<br>[354, 557]	CR
Estimated duration moving (0.025 <= velocity < 0.075 - Zone 2)	2.27E+03<br>[1.75E+03, 2.95E+03]	
Estimated duration moving (velocity >= 0.075 - Zone 2)	3.3E+03<br>[2.33E+03, 4.65E+03]	CR

CR = Check residuals

Data summary

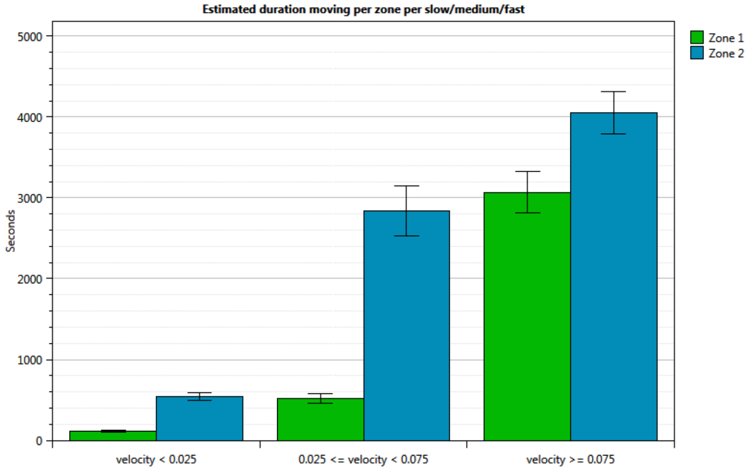

Genotype Zone 1	Genotype Zone 2	Genotype Zone 3	Mean velocity < 0.025 - Zone 1	StdErr velocity < 0.025 - Zone 1	Mean 0.025 <= velocity < 0.075 - Zone 1	StdErr 0.025 <= velocity < 0.075 - Zone 1	Mean velocity >= 0.075 - Zone 1	StdErr velocity >= 0.075 - Zone 1	Mean velocity < 0.025 - Zone 2	StdErr velocity < 0.025 - Zone 2	Mean 0.025 <= velocity < 0.075 - Zone 2	StdErr 0.025 <= velocity < 0.075 - Zone 2	Mean velocity >= 0.075 - Zone 2	StdErr velocity >= 0.075 - Zone 2
Cur3	RMX180	Neutral	111.73	13.97	519.81	62.68	3070.18	254.23	542.24	51.5	2840.68	310.85	4054.54	260.17

Analysis estimated duration moving (velocity < 0.025 - Zone 1)

**Analysis model**	Linear mixed model fit by REML: Estimated_duration_moving_velocity_0_025_Zone_1 ~ 1 + (1|Genotype_Zone_1:Plant_Zone_1) + (1|Genotype_Zone_2:Plant_Zone_2)
**Transformation**	Natural logarithm

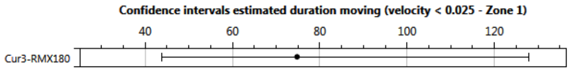

Confidence intervals estimated duration moving (velocity < 0.025 - Zone 1)

Genotype Zone 1	Genotype Zone 2	Mean	Lower 95% CL	Upper 95% CL	Group
Cur3	RMX180	74.78	43.71	128	a

Model summary

```
Linear mixed model fit by REML. t-tests use Satterthwaite's method ['lmerModLmerTest']
Formula: Estimated_duration_moving_velocity_0_025_Zone_1 ~ 1 + (1 | Genotype_Zone_1:Plant_Zone_1) +
(1 | Genotype_Zone_2:Plant_Zone_2)
   Data: data

REML criterion at convergence: 121.5

Scaled residuals:
    Min      1Q  Median      3Q     Max
-3.5276 -0.2905  0.1350  0.5887  1.1183

Random effects:
 Groups                       Name        Variance Std.Dev.
 Genotype_Zone_1:Plant_Zone_1 (Intercept) 0.0000   0.000
 Genotype_Zone_2:Plant_Zone_2 (Intercept) 0.2401   0.490
 Residual                                 1.2369   1.112
Number of obs: 38, groups:  Genotype_Zone_1:Plant_Zone_1, 10; Genotype_Zone_2:Plant_Zone_2, 10

Fixed effects:
            Estimate Std. Error     df t value Pr(>|t|)
(Intercept)   4.3146     0.2382 9.1960   18.11 1.66e-08 ***
---
Signif. codes:  0 '***' 0.001 '**' 0.01 '*' 0.05 '.' 0.1 ' ' 1
```

Model residuals

Statistic	Value
Sample skewness	-2.027
Sample excess kurtosis	5.304
Passed Shapiro Wilk normality test	No (p-value = 2.591E-05 < 0.05)

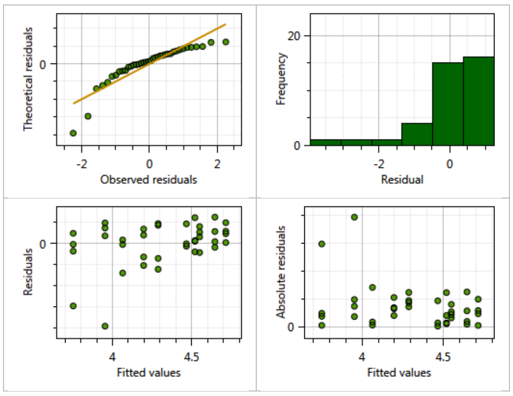

Analysis estimated duration moving (0.025 <= velocity < 0.075 - Zone 1)

**Analysis model**	Linear mixed model fit by REML: Estimated_duration_moving_0_025_velocity_0_075_Zone_1 ~ 1 + (1|Genotype_Zone_1:Plant_Zone_1) + (1|Genotype_Zone_2:Plant_Zone_2)
**Transformation**	Natural logarithm

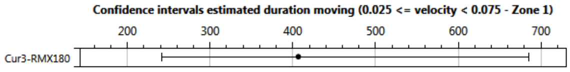

Confidence intervals estimated duration moving (0.025 <= velocity < 0.075 - Zone 1)

Genotype Zone 1	Genotype Zone 2	Mean	Lower 95% CL	Upper 95% CL	Group
Cur3	RMX180	406.6	241.2	685.6	a

Model summary

```
Linear mixed model fit by REML. t-tests use Satterthwaite's method ['lmerModLmerTest']
Formula: Estimated_duration_moving_0_025_velocity_0_075_Zone_1 ~ 1 + (1 |
Genotype_Zone_1:Plant_Zone_1) + (1 | Genotype_Zone_2:Plant_Zone_2)
   Data: data

REML criterion at convergence: 96.6

Scaled residuals:
    Min      1Q  Median      3Q     Max
-2.7788 -0.5073 -0.0066  0.4950  1.4961

Random effects:
 Groups                       Name        Variance Std.Dev.
 Genotype_Zone_1:Plant_Zone_1 (Intercept) 0.1780   0.4219
 Genotype_Zone_2:Plant_Zone_2 (Intercept) 0.2175   0.4664
 Residual                                 0.5149   0.7176
Number of obs: 37, groups:  Genotype_Zone_1:Plant_Zone_1, 10; Genotype_Zone_2:Plant_Zone_2, 10

Fixed effects:
            Estimate Std. Error     df t value Pr(>|t|)
(Intercept)   6.0079     0.2324 9.3841   25.86 4.85e-10 ***
---
Signif. codes:  0 '***' 0.001 '**' 0.01 '*' 0.05 '.' 0.1 ' ' 1
```

Model residuals

Statistic	Value
Sample skewness	-1.033
Sample excess kurtosis	1.881
Passed Shapiro Wilk normality test	Yes (p-value = 0.06232 > 0.05)

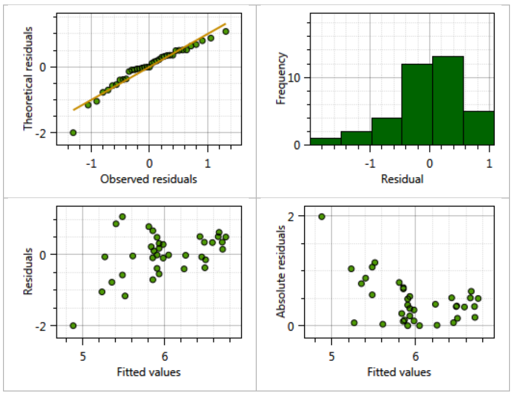

Analysis estimated duration moving (velocity >= 0.075 - Zone 1)

**Analysis model**	Linear mixed model fit by REML: Estimated_duration_moving_velocity_0_075_Zone_1 ~ 1 + (1|Genotype_Zone_1:Plant_Zone_1) + (1|Genotype_Zone_2:Plant_Zone_2)
**Transformation**	Natural logarithm

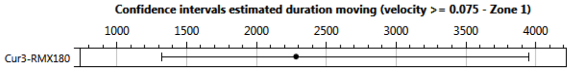

Confidence intervals estimated duration moving (velocity >= 0.075 - Zone 1)

Genotype Zone 1	Genotype Zone 2	Mean	Lower 95% CL	Upper 95% CL	Group
Cur3	RMX180	2283	1319	3952	a

Model summary

```
Linear mixed model fit by REML. t-tests use Satterthwaite's method ['lmerModLmerTest']
Formula: Estimated_duration_moving_velocity_0_075_Zone_1 ~ 1 + (1 | Genotype_Zone_1:Plant_Zone_1) +
(1 | Genotype_Zone_2:Plant_Zone_2)
   Data: data

REML criterion at convergence: 130.5

Scaled residuals:
    Min      1Q  Median      3Q     Max
-4.9451  0.0222  0.2202  0.4102  0.7360

Random effects:
 Groups                       Name        Variance Std.Dev.
 Genotype_Zone_1:Plant_Zone_1 (Intercept) 0.06902  0.2627
 Genotype_Zone_2:Plant_Zone_2 (Intercept) 0.00000  0.0000
 Residual                                 1.74364  1.3205
Number of obs: 38, groups:  Genotype_Zone_1:Plant_Zone_1, 10; Genotype_Zone_2:Plant_Zone_2, 10

Fixed effects:
            Estimate Std. Error     df t value Pr(>|t|)
(Intercept)   7.7332     0.2299 6.6858   33.64 1.04e-08 ***
---
Signif. codes:  0 '***' 0.001 '**' 0.01 '*' 0.05 '.' 0.1 ' ' 1
```

Model residuals

Statistic	Value
Sample skewness	-4.014
Sample excess kurtosis	18.34
Passed Shapiro Wilk normality test	No (p-value = 8.218E-10 < 0.05)

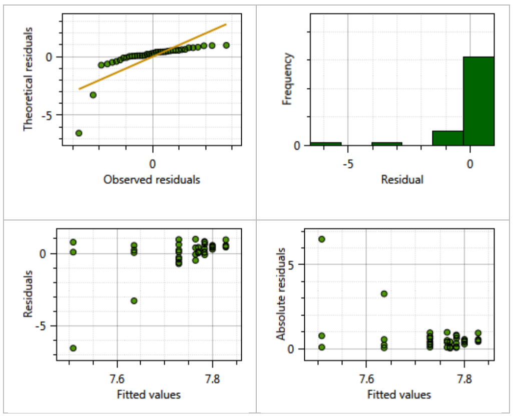

Analysis estimated duration moving (velocity < 0.025 - Zone 2)

**Analysis model**	Linear mixed model fit by REML: Estimated_duration_moving_velocity_0_025_Zone_2 ~ 1 + (1|Genotype_Zone_1:Plant_Zone_1) + (1|Genotype_Zone_2:Plant_Zone_2)
**Transformation**	Natural logarithm

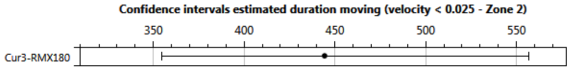

Confidence intervals estimated duration moving (velocity < 0.025 - Zone 2)

Genotype Zone 1	Genotype Zone 2	Mean	Lower 95% CL	Upper 95% CL	Group
Cur3	RMX180	444.3	354.4	557	a

Model summary

```
Linear mixed model fit by REML. t-tests use Satterthwaite's method ['lmerModLmerTest']
Formula: Estimated_duration_moving_velocity_0_025_Zone_2 ~ 1 + (1 | Genotype_Zone_1:Plant_Zone_1) +
(1 | Genotype_Zone_2:Plant_Zone_2)
   Data: data

REML criterion at convergence: 84.1

Scaled residuals:
    Min      1Q  Median      3Q     Max
-2.6699 -0.6155  0.2229  0.6672  1.8572

Random effects:
 Groups                       Name        Variance Std.Dev.
 Genotype_Zone_1:Plant_Zone_1 (Intercept) 0.000    0.0000
```

```
 Genotype_Zone_2:Plant_Zone_2 (Intercept) 0.000    0.0000
 Residual                                 0.486    0.6972
Number of obs: 39, groups:  Genotype_Zone_1:Plant_Zone_1, 10; Genotype_Zone_2:Plant_Zone_2, 10

Fixed effects:
            Estimate Std. Error      df t value Pr(>|t|)
(Intercept)   6.0965     0.1116 38.0000   54.61   <2e-16 ***
---
Signif. codes:  0 '***' 0.001 '**' 0.01 '*' 0.05 '.' 0.1 ' ' 1
```

Model residuals

Statistic	Value
Sample skewness	-0.835
Sample excess kurtosis	0.7831
Passed Shapiro Wilk normality test	No (p-value = 0.03979 < 0.05)

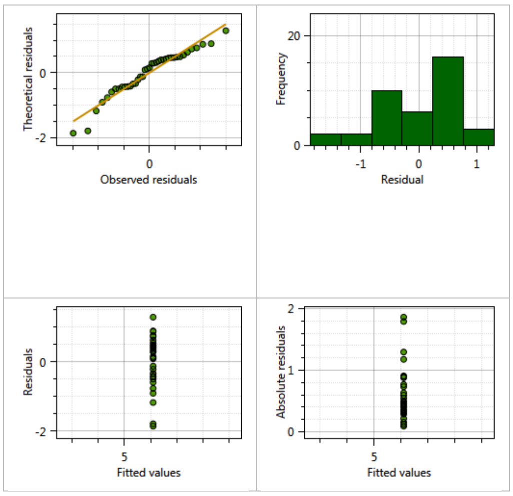

Analysis estimated duration moving (0.025 <= velocity < 0.075 - Zone 2)

**Analysis model**	Linear mixed model fit by REML: Estimated_duration_moving_0_025_velocity_0_075_Zone_2 ~ 1 + (1|Genotype_Zone_1:Plant_Zone_1) + (1|Genotype_Zone_2:Plant_Zone_2)
**Transformation**	Natural logarithm

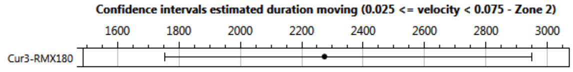

Confidence intervals estimated duration moving (0.025 <= velocity < 0.075 - Zone 2)

Genotype Zone 1	Genotype Zone 2	Mean	Lower 95% CL	Upper 95% CL	Group
Cur3	RMX180	2274	1753	2950	a

Model summary

```
Linear mixed model fit by REML. t-tests use Satterthwaite's method ['lmerModLmerTest']
Formula: Estimated_duration_moving_0_025_velocity_0_075_Zone_2 ~ 1 + (1 |
Genotype_Zone_1:Plant_Zone_1) + (1 | Genotype_Zone_2:Plant_Zone_2)
   Data: data

REML criterion at convergence: 81.8

Scaled residuals:
    Min      1Q  Median      3Q     Max
-1.8051 -0.7523 -0.2064  0.8612  1.6990

Random effects:
 Groups                       Name        Variance  Std.Dev.
 Genotype_Zone_1:Plant_Zone_1 (Intercept) 6.725e-18 2.593e-09
 Genotype_Zone_2:Plant_Zone_2 (Intercept) 1.896e-02 1.377e-01
 Residual                                 4.416e-01 6.645e-01
Number of obs: 39, groups:  Genotype_Zone_1:Plant_Zone_1, 10; Genotype_Zone_2:Plant_Zone_2, 10

Fixed effects:
            Estimate Std. Error    df t value Pr(>|t|)
(Intercept)    7.729      0.115 8.997    67.2 1.82e-13 ***
---
Signif. codes:  0 '***' 0.001 '**' 0.01 '*' 0.05 '.' 0.1 ' ' 1
```

Model residuals

Statistic	Value
Sample skewness	0.08388
Sample excess kurtosis	-1.034
Passed Shapiro Wilk normality test	Yes (p-value = 0.1588 > 0.05)

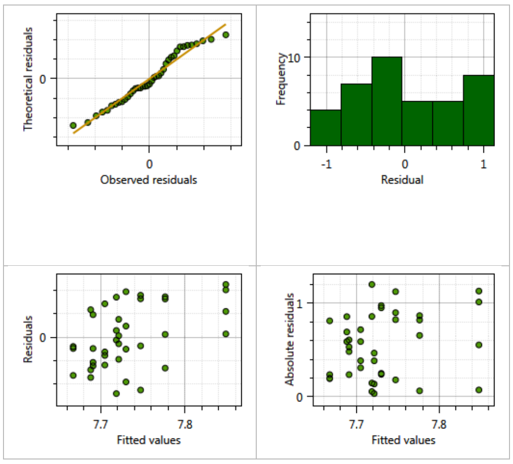

Analysis estimated duration moving (velocity >= 0.075 - Zone 2)

**Analysis model**	Linear mixed model fit by REML: Estimated_duration_moving_velocity_0_075_Zone_2 ~ 1 + (1|Genotype_Zone_1:Plant_Zone_1) + (1|Genotype_Zone_2:Plant_Zone_2)

**Transformation**	Natural logarithm

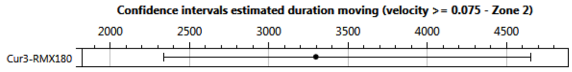

Confidence intervals estimated duration moving (velocity >= 0.075 - Zone 2)

Genotype Zone 1	Genotype Zone 2	Mean	Lower 95% CL	Upper 95% CL	Group
Cur3	RMX180	3297	2335	4655	a

Model summary

```
Linear mixed model fit by REML. t-tests use Satterthwaite's method ['lmerModLmerTest']
Formula: Estimated_duration_moving_velocity_0_075_Zone_2 ~ 1 + (1 | Genotype_Zone_1:Plant_Zone_1) +
(1 | Genotype_Zone_2:Plant_Zone_2)
   Data: data

REML criterion at convergence: 104

Scaled residuals:
    Min      1Q  Median      3Q     Max
-3.7442 -0.0170  0.2774  0.4477  0.9013

Random effects:
 Groups                       Name        Variance  Std.Dev.
 Genotype_Zone_1:Plant_Zone_1 (Intercept) 6.864e-17 8.285e-09
 Genotype_Zone_2:Plant_Zone_2 (Intercept) 2.528e-02 1.590e-01
 Residual                                 7.983e-01 8.935e-01
Number of obs: 39, groups:  Genotype_Zone_1:Plant_Zone_1, 10; Genotype_Zone_2:Plant_Zone_2, 10

Fixed effects:
            Estimate Std. Error     df t value Pr(>|t|)
(Intercept)   8.1007     0.1517 8.6943    53.4    3e-12 ***
---
Signif. codes:  0 '***' 0.001 '**' 0.01 '*' 0.05 '.' 0.1 ' ' 1
```

Model residuals

Statistic	Value
Sample skewness	-2.95
Sample excess kurtosis	8.756
Passed Shapiro Wilk normality test	No (p-value = 4.53E-09 < 0.05)

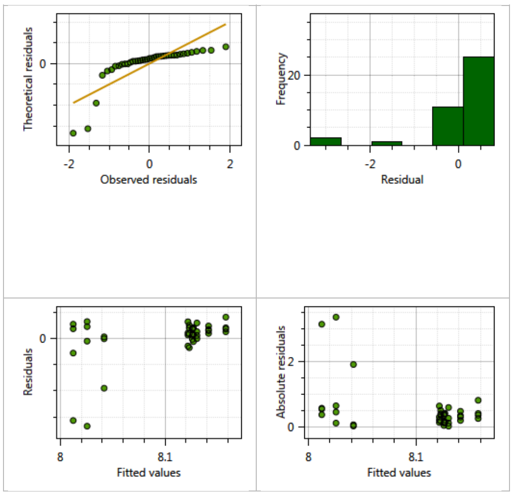

Analysis estimated duration moving velocity < 0.025 (diff. Zone 1 - Zone 2)

**Analysis model**	Generalized linear mixed model with dispersion factor, formula=cbind(Estimated_duration_moving_velocity_0_025_Zone_1,Estimated_duration_moving_velocity_0_025_Zone_2) ~ 1 + (1|Genotype_Zone_1:Plant_Zone_1) + (1|Genotype_Zone_2:Plant_Zone_2)
**Transformation**	Logit

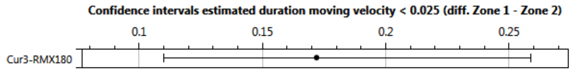

Confidence intervals estimated duration moving velocity < 0.025 (diff. Zone 1 - Zone 2)

Genotype Zone 1	Genotype Zone 2	Mean	Lower 95% CL	Upper 95% CL	Group
Cur3	RMX180	0.1719	0.1098	0.2589	a

Model summary

```
Linear mixed model fit by REML. t-tests use Satterthwaite's method ['lmerModLmerTest']
Formula: ziFormula
   Data: data
Weights: wi

REML criterion at convergence: 125.8

Scaled residuals:
     Min       1Q   Median       3Q      Max
-1.31960 -0.66342 -0.04034  0.56765  2.25676

Random effects:
 Groups                       Name        Variance  Std.Dev.
 Genotype_Zone_1:Plant_Zone_1 (Intercept) 2.608e-03  0.05107
 Genotype_Zone_2:Plant_Zone_2 (Intercept) 1.555e-01  0.39434
 Residual                                 1.088e+02 10.42833
Number of obs: 39, groups:  Genotype_Zone_1:Plant_Zone_1, 10; Genotype_Zone_2:Plant_Zone_2, 10

Fixed effects:
            Estimate Std. Error      df t value Pr(>|t|)
(Intercept)  -1.5723     0.2165  6.4740  -7.263 0.000244 ***
---
Signif. codes:  0 '***' 0.001 '**' 0.01 '*' 0.05 '.' 0.1 ' ' 1

Dispersion: 10.43
```

Model residuals

Statistic	Value
Sample skewness	0.6566
Sample excess kurtosis	-0.359
Passed Shapiro Wilk normality test	Yes (p-value = 0.05037 > 0.05)

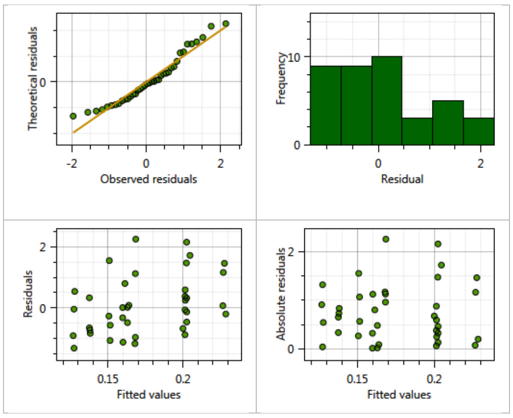

Analysis estimated duration moving 0.025 <= velocity < 0.075 (diff. Zone 1 - Zone 2)

**Analysis model**	Generalized linear mixed model with dispersion factor, formula=cbind(Estimated_duration_moving_0_025_velocity_0_075_Zone_1,Estimated_duration_moving_0_025_velocity_0_075_Zone_2) ~ 1 + (1|Genotype_Zone_1:Plant_Zone_1) + (1|Genotype_Zone_2:Plant_Zone_2)
**Transformation**	Logit

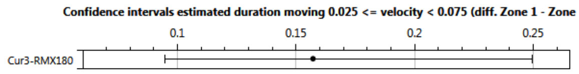

Confidence intervals estimated duration moving 0.025 <= velocity < 0.075 (diff. Zone 1 - Zone 2)

Genotype Zone 1	Genotype Zone 2	Mean	Lower 95% CL	Upper 95% CL	Group
Cur3	RMX180	0.1572	0.09458	0.2497	a

Model summary

```
Linear mixed model fit by REML. t-tests use Satterthwaite's method ['lmerModLmerTest']
Formula: ziFormula
   Data: data
Weights: wi

REML criterion at convergence: 126.7

Scaled residuals:
     Min       1Q   Median       3Q      Max
-1.22685 -0.79728  0.00827  0.95022  1.88724

Random effects:
 Groups                      Name        Variance  Std.Dev.
 Genotype_Zone_1:Plant_Zone_1 (Intercept)   0.08382  0.2895
 Genotype_Zone_2:Plant_Zone_2 (Intercept)   0.32417  0.5694
 Residual                               443.20821 21.0525
Number of obs: 39, groups:  Genotype_Zone_1:Plant_Zone_1, 10; Genotype_Zone_2:Plant_Zone_2, 10

Fixed effects:
            Estimate Std. Error      df t value Pr(>|t|)
(Intercept)  -1.6795     0.2622 10.6448  -6.405 5.86e-05 ***
---
Signif. codes:  0 '***' 0.001 '**' 0.01 '*' 0.05 '.' 0.1 ' ' 1

Dispersion: 21.05
```

Model residuals

Statistic	Value
Sample skewness	0.2752
Sample excess kurtosis	-1.04
Passed Shapiro Wilk normality test	Yes (p-value = 0.05816 > 0.05)

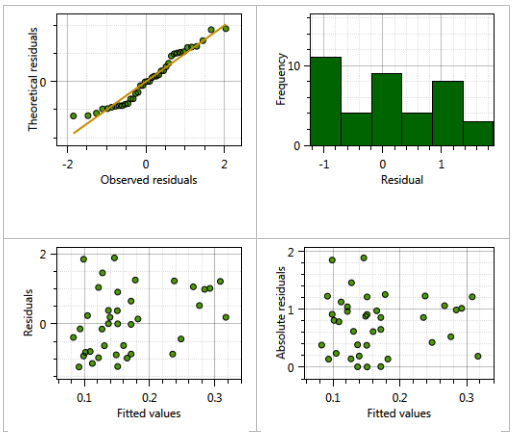

Analysis estimated duration moving velocity >= 0.075 (diff. Zone 1 - Zone 2)

**Analysis model**	Generalized linear mixed model with dispersion factor, formula=cbind(Estimated_duration_moving_velocity_0_075_Zone_1,Estimated_duration_moving_velocity_0_075_Zone_2) ~ 1 + (1|Genotype_Zone_1:Plant_Zone_1) + (1|Genotype_Zone_2:Plant_Zone_2)
**Transformation**	Logit

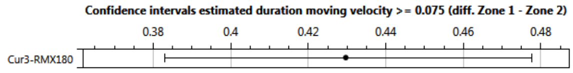

Confidence intervals estimated duration moving velocity >= 0.075 (diff. Zone 1 - Zone 2)

Genotype Zone 1	Genotype Zone 2	Mean	Lower 95% CL	Upper 95% CL	Group
Cur3	RMX180	0.4297	0.3829	0.4777	a

Model summary

```
Linear mixed model fit by REML. t-tests use Satterthwaite's method ['lmerModLmerTest']
Formula: ziFormula
   Data: data
Weights: wi

REML criterion at convergence: 61.4
```

```
Scaled residuals:
    Min      1Q  Median      3Q     Max
-1.8455 -0.7356 -0.1421  0.7096  2.0087

Random effects:
 Groups                      Name        Variance  Std.Dev.
 Genotype_Zone_1:Plant_Zone_1 (Intercept) 1.721e-02  0.13121
 Genotype_Zone_2:Plant_Zone_2 (Intercept) 1.342e-03  0.03663
 Residual                                3.405e+02 18.45247
Number of obs: 39, groups:  Genotype_Zone_1:Plant_Zone_1, 10; Genotype_Zone_2:Plant_Zone_2, 10

Fixed effects:
            Estimate Std. Error       df t value Pr(>|t|)
(Intercept) -0.28325    0.08317  7.49360  -3.406   0.0102 *
---
Signif. codes:  0 '***' 0.001 '**' 0.01 '*' 0.05 '.' 0.1 ' ' 1

Dispersion: 18.45
```

Model residuals

Statistic	Value
Sample skewness	0.06689
Sample excess kurtosis	-0.7175
Passed Shapiro Wilk normality test	Yes (p-value = 0.7951 > 0.05)

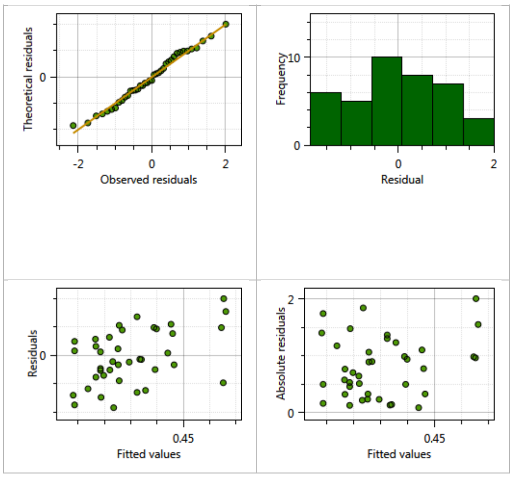

Halt frequency per zone

Selected zones	Zone 1, Zone 2
Data transformation	Natural logarithm
Analysis	Zone difference analysis

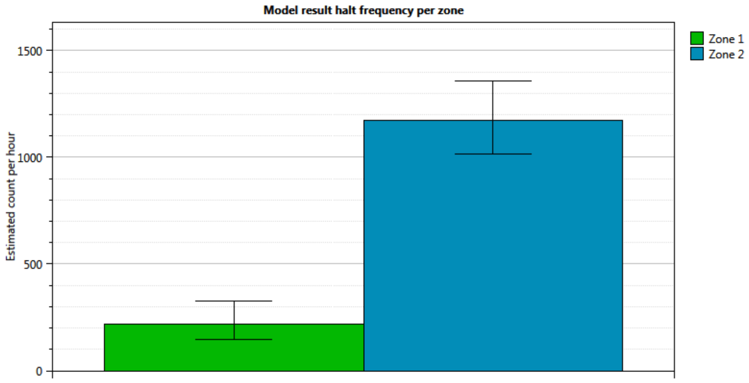

Results difference tests Zone 1 - Zone 2: p values and 95% confidence intervals of the difference on the transformed scale for each statistic.

Behaviour statistic	Cur3-RMX180
Halt frequency (diff. Zone 1 - Zone 2)	p=7.73E-05****
	[-1.84, -0.967]

The model predictions and 95% confidence intervals for each statistic.

Statistic	Cur3-RMX180	Remark
Halt frequency (Zone 1)	217	CR
	[145, 325]	
Halt frequency (Zone 2)	1.17E+03	
	[1.02E+03, 1.36E+03]	

CR = Check residuals

Data summary

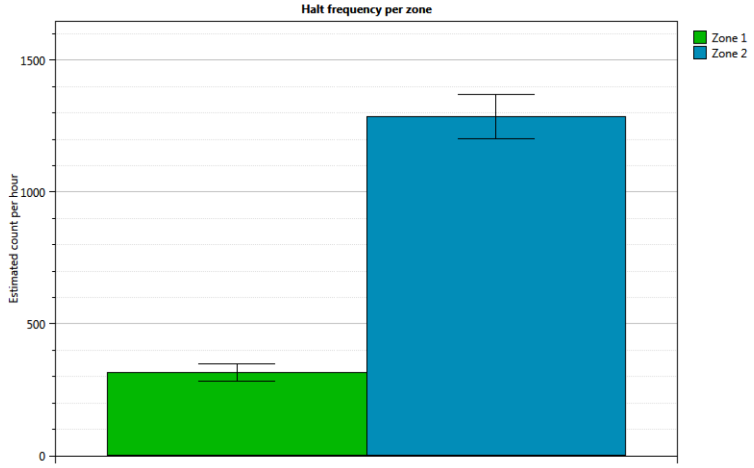

Genotype Zone 1	Genotype Zone 2	Genotype Zone 3	Mean Zone 1	StdErr Zone 1	Mean Zone 2	StdErr Zone 2
Cur3	RMX180	Neutral	316.26	32.84	1286.11	85.21

Analysis halt frequency (Zone 1)

**Analysis model**	Linear mixed model fit by REML: Halt_frequency_Zone_1 ~ 1 + (1|Genotype_Zone_1:Plant_Zone_1) + (1|Genotype_Zone_2:Plant_Zone_2)
**Transformation**	Natural logarithm

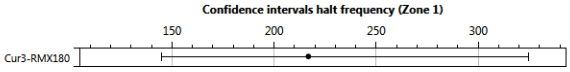

Confidence intervals halt frequency (Zone 1)

Genotype Zone 1	Genotype Zone 2	Mean	Lower 95% CL	Upper 95% CL	Group
Cur3	RMX180	216.8	144.7	324.7	a

Model summary

```
Linear mixed model fit by REML. t-tests use Satterthwaite's method ['lmerModLmerTest']
Formula: Halt_frequency_Zone_1 ~ 1 + (1 | Genotype_Zone_1:Plant_Zone_1) +      (1 |
Genotype_Zone_2:Plant_Zone_2)
   Data: data

REML criterion at convergence: 128.3

Scaled residuals:
    Min      1Q  Median      3Q     Max
-4.1279 -0.0675  0.1788  0.5340  1.0915

Random effects:
 Groups                       Name        Variance  Std.Dev.
 Genotype_Zone_1:Plant_Zone_1 (Intercept) 0.000e+00 0.000e+00
 Genotype_Zone_2:Plant_Zone_2 (Intercept) 4.891e-15 6.994e-08
 Residual                                 1.555e+00 1.247e+00
Number of obs: 39, groups:  Genotype_Zone_1:Plant_Zone_1, 10; Genotype_Zone_2:Plant_Zone_2, 10

Fixed effects:
            Estimate Std. Error      df t value Pr(>|t|)
(Intercept)   5.3788     0.1997 38.0000   26.94   <2e-16 ***
---
Signif. codes:  0 '***' 0.001 '**' 0.01 '*' 0.05 '.' 0.1 ' ' 1
```

Model residuals

Statistic	Value
Sample skewness	-2.648
Sample excess kurtosis	8.513
Passed Shapiro Wilk normality test	No (p-value = 3.19E-07 < 0.05)

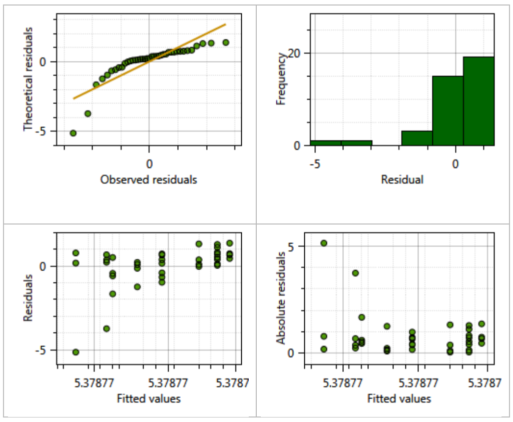

Analysis halt frequency (Zone 2)

**Analysis model**	Linear mixed model fit by REML: Halt_frequency_Zone_2 ~ 1 + (1|Genotype_Zone_1:Plant_Zone_1) + (1|Genotype_Zone_2:Plant_Zone_2)
**Transformation**	Natural logarithm

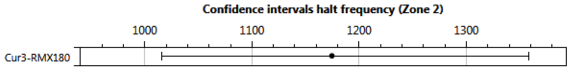

Confidence intervals halt frequency (Zone 2)

Genotype Zone 1	Genotype Zone 2	Mean	Lower 95% CL	Upper 95% CL	Group
Cur3	RMX180	1175	1016	1358	a

Model summary

```
Linear mixed model fit by REML. t-tests use Satterthwaite's method ['lmerModLmerTest']
Formula: Halt_frequency_Zone_2 ~ 1 + (1 | Genotype_Zone_1:Plant_Zone_1) +      (1 |
Genotype_Zone_2:Plant_Zone_2)
   Data: data

REML criterion at convergence: 50.6
```

```
Scaled residuals:
     Min       1Q   Median       3Q      Max
-2.95640 -0.71408  0.06847  0.79717  1.86046

Random effects:
 Groups                         Name        Variance Std.Dev.
 Genotype_Zone_1:Plant_Zone_1 (Intercept) 0.0000   0.0000
 Genotype_Zone_2:Plant_Zone_2 (Intercept) 0.0000   0.0000
 Residual                                 0.2014   0.4488
Number of obs: 39, groups:  Genotype_Zone_1:Plant_Zone_1, 10; Genotype_Zone_2:Plant_Zone_2, 10

Fixed effects:
            Estimate Std. Error       df t value Pr(>|t|)
(Intercept)  7.06865    0.07187 38.00000   98.36   <2e-16 ***
---
Signif. codes:  0 '***' 0.001 '**' 0.01 '*' 0.05 '.' 0.1 ' ' 1
```

Model residuals

Statistic	Value
Sample skewness	-0.558
Sample excess kurtosis	0.4709
Passed Shapiro Wilk normality test	Yes (p-value = 0.2477 > 0.05)

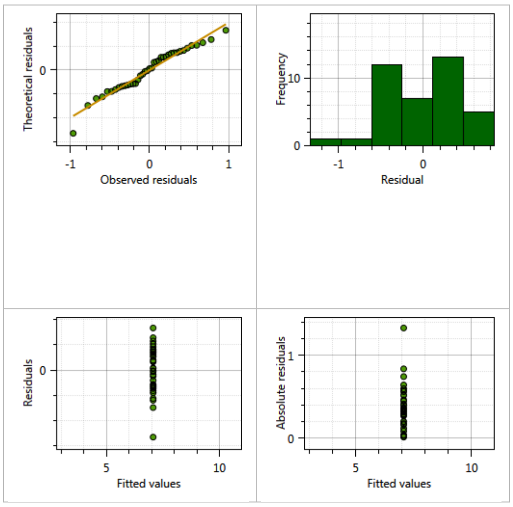

Analysis halt frequency (diff. Zone 1 - Zone 2)

**Analysis model**	Generalized linear mixed model with dispersion factor, formula=cbind(Halt_frequency_Zone_1,Halt_frequency_Zone_2) ~ 1 + (1|Genotype_Zone_1:Plant_Zone_1) + (1|Genotype_Zone_2:Plant_Zone_2)
**Transformation**	Logit

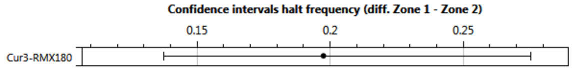

Confidence intervals halt frequency (diff. Zone 1 - Zone 2)

Genotype Zone 1	Genotype Zone 2	Mean	Lower 95% CL	Upper 95% CL	Group
Cur3	RMX180	0.1974	0.1373	0.2754	a

Model summary

```
Linear mixed model fit by REML. t-tests use Satterthwaite's method ['lmerModLmerTest']
Formula: ziFormula
   Data: data
Weights: wi

REML criterion at convergence: 108.7

Scaled residuals:
     Min       1Q   Median       3Q      Max
-1.40874 -0.66926 -0.06651  0.64189  1.99740

Random effects:
 Groups                         Name        Variance  Std.Dev.
 Genotype_Zone_1:Plant_Zone_1 (Intercept)   0.05099  0.2258
 Genotype_Zone_2:Plant_Zone_2 (Intercept)   0.10237  0.3199
 Residual                                 194.95683 13.9627
Number of obs: 39, groups:  Genotype_Zone_1:Plant_Zone_1, 10; Genotype_Zone_2:Plant_Zone_2, 10

Fixed effects:
            Estimate Std. Error      df t value Pr(>|t|)
(Intercept)  -1.4023     0.1884  7.9113  -7.445 7.73e-05 ***
---
Signif. codes:  0 '***' 0.001 '**' 0.01 '*' 0.05 '.' 0.1 ' ' 1

Dispersion: 13.96
```

Model residuals

Statistic	Value
Sample skewness	0.4086
Sample excess kurtosis	-0.6569
Passed Shapiro Wilk normality test	Yes (p-value = 0.191 > 0.05)

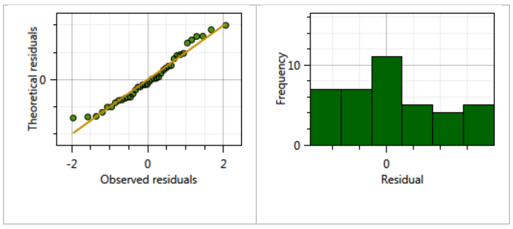

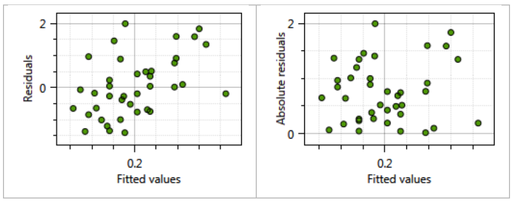

Halt frequency per zone per hour

Selected hours	0, 1, 2, 3, 4, 5, 6, 7
Selected zones	Zone 1, Zone 2
Data transformation	Natural logarithm
Analysis	Zone difference analysis

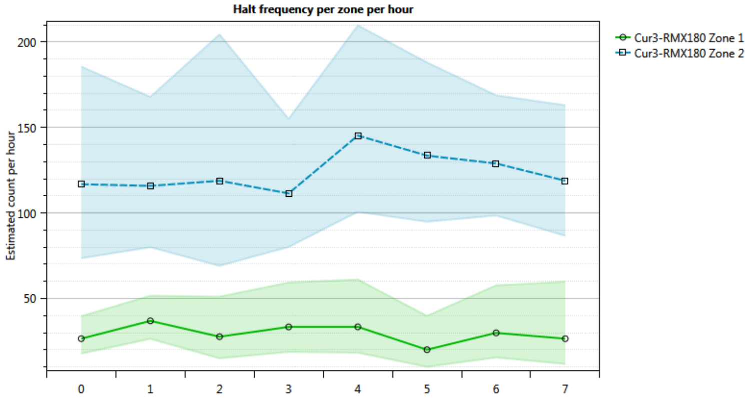

Results difference tests Zone 1 - Zone 2: p values and 95% confidence intervals of the difference on the transformed scale for each statistic.

Behaviour statistic	Cur3-RMX180	Remark
Halt frequency H0 (diff. Zone 1 - Zone 2)	p=0.000622***<br>[-1.95, -0.769]	CR
Halt frequency H1 (diff. Zone 1 - Zone 2)	p=0.000185***<br>[-1.86, -0.761]	
Halt frequency H2 (diff. Zone 1 - Zone 2)	p=0.00314**<br>[-2.05, -0.58]	CR
Halt frequency H3 (diff. Zone 1 - Zone 2)	p=0.00358**<br>[-1.81, -0.482]	CR
Halt frequency H4 (diff. Zone 1 - Zone 2)	p=0.00279**<br>[-2.18, -0.7]	CR
Halt frequency H5 (diff. Zone 1 - Zone 2)	p=0.000343***<br>[-2.3, -0.97]	CR
Halt frequency H6 (diff. Zone 1 - Zone 2)	p=0.000276***<br>[-2.23, -0.922]	CR
Halt frequency H7 (diff. Zone 1 - Zone 2)	p=0.00422**<br>[-2.32, -0.692]	CR

CR = Check residuals

The model predictions and 95% confidence intervals for each statistic.

Statistic	Cur3-RMX180	Remark
Halt frequency (H0 - Zone 1)	26.5<br>[17.7, 39.7]	
Halt frequency (H0 - Zone 2)	117<br>[73.5, 186]	CR
Halt frequency (H1 - Zone 1)	37<br>[26.4, 51.7]	CR
Halt frequency (H1 - Zone 2)	116<br>[80, 168]	CR
Halt frequency (H2 - Zone 1)	27.7<br>[15, 51.1]	
Halt frequency (H2 - Zone 2)	119<br>[69.1, 204]	CR
Halt frequency (H3 - Zone 1)	33.4<br>[18.8, 59.4]	CR
Halt frequency (H3 - Zone 2)	111<br>[80.1, 155]	CR
Halt frequency (H4 - Zone 1)	33.4<br>[18.3, 61.1]	CR
Halt frequency (H4 - Zone 2)	145<br>[101, 210]	CR
Halt frequency (H5 - Zone 1)	20.1<br>[10.1, 39.9]	CR
Halt frequency (H5 - Zone 2)	134<br>[94.8, 188]	CR
Halt frequency (H6 - Zone 1)	29.9<br>[15.5, 57.8]	
Halt frequency (H6 - Zone 2)	129<br>[98.5, 169]	CR
Halt frequency (H7 - Zone 1)	26.5<br>[11.8, 59.8]	
Halt frequency (H7 - Zone 2)	119<br>[86.6, 163]	CR

CR = Check residuals

Data summary

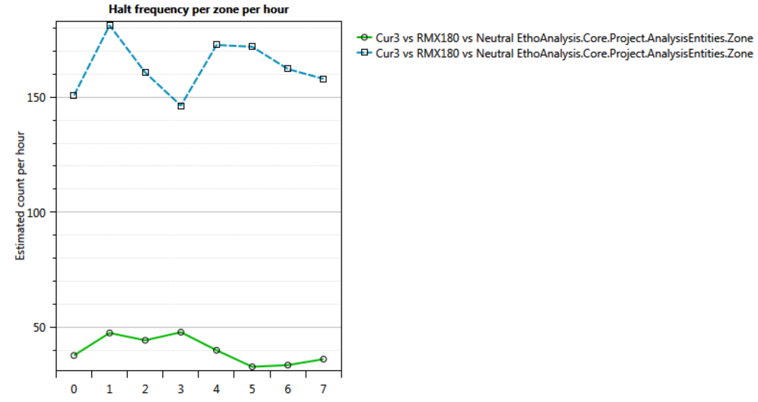

Genotype Zone 1	Genotype Zone 2	Genotype Zone 3	Mean H0 - Zone 1	StdErr H0 - Zone 1	Mean H0 - Zone 2	StdErr H0 - Zone 2	Mean H1 - Zone 1	StdErr H1 - Zone 1	Mean H1 - Zone 2	StdErr H1 - Zone 2	Mean H2 - Zone 1	StdErr H2 - Zone 1	Mean H2 - Zone 2	StdErr H2 - Zone 2	Mean H3 - Zone 1	StdErr H3 - Zone 1	Mean H3 - Zone 2	StdErr H3 - Zone 2	Mean H4 - Zone 1	StdErr H4 - Zone 1	Mean H4 - Zone 2	StdErr H4 - Zone 2	Mean H5 - Zone 1	StdErr H5 - Zone 1	Mean H5 - Zone 2	StdErr H5 - Zone 2	Mean H6 - Zone 1	StdErr H6 - Zone 1	Mean H6 - Zone 2	StdE H6 - Zone 2
Cur3	RMX180	Neutral	37.78	6.47	150.72	13.16	47.56	5.6	181.21	42.45	44.37	8.47	160.67	14.9	47.9	7.82	146.3	14.19	40.04	7.31	172.67	17.16	32.88	5.92	171.89	16.55	33.6	7.52	162.2	17.48

Analysis halt frequency (H0 - Zone 1)

**Analysis model**	Linear mixed model fit by REML: Halt_frequency_H0_Zone_1 ~ 1 + (1|Genotype_Zone_1:Plant_Zone_1) + (1|Genotype_Zone_2:Plant_Zone_2)
**Transformation**	Natural logarithm

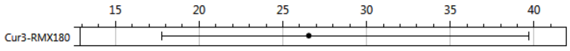

Confidence intervals halt frequency (H0 - Zone 1)

Genotype Zone 1	Genotype Zone 2	Mean	Lower 95% CL	Upper 95% CL	Group
Cur3	RMX180	26.55	17.75	39.7	a

Model summary

```
Linear mixed model fit by REML. t-tests use Satterthwaite's method ['lmerModLmerTest']
Formula: Halt_frequency_H0_Zone_1 ~ 1 + (1 | Genotype_Zone_1:Plant_Zone_1) +      (1 |
Genotype_Zone_2:Plant_Zone_2)
   Data: data

REML criterion at convergence: 106.6

Scaled residuals:
    Min      1Q  Median      3Q     Max
-2.8249 -0.7370  0.2439  0.6566  1.7006

Random effects:
 Groups                       Name        Variance  Std.Dev.
 Genotype_Zone_1:Plant_Zone_1 (Intercept) 9.845e-19 9.922e-10
 Genotype_Zone_2:Plant_Zone_2 (Intercept) 9.644e-16 3.106e-08
 Residual                                 1.331e+00 1.154e+00
Number of obs: 34, groups:  Genotype_Zone_1:Plant_Zone_1, 10; Genotype_Zone_2:Plant_Zone_2, 10

Fixed effects:
            Estimate Std. Error      df t value Pr(>|t|)
(Intercept)   3.2789     0.1979 33.0000   16.57   <2e-16 ***
---
Signif. codes:  0 '***' 0.001 '**' 0.01 '*' 0.05 '.' 0.1 ' ' 1
```

Model residuals

Statistic	Value
Sample skewness	-0.8491
Sample excess kurtosis	0.8543
Passed Shapiro Wilk normality test	Yes (p-value = 0.09851 > 0.05)

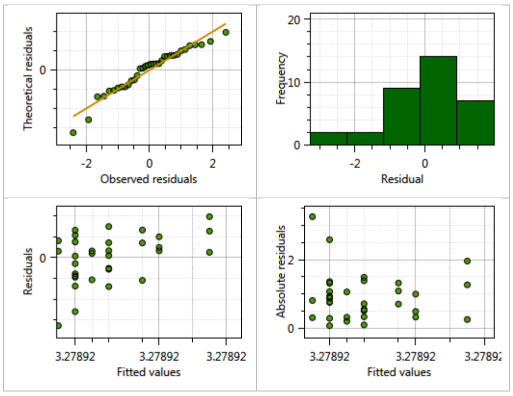

Analysis halt frequency (H0 - Zone 2)

**Analysis model**	Linear mixed model fit by REML: Halt_frequency_H0_Zone_2 ~ 1 + (1|Genotype_Zone_1:Plant_Zone_1) + (1|Genotype_Zone_2:Plant_Zone_2)
**Transformation**	Natural logarithm

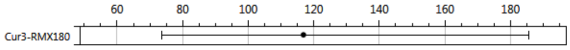

Confidence intervals halt frequency (H0 - Zone 2)

Genotype Zone 1	Genotype Zone 2	Mean	Lower 95% CL	Upper 95% CL	Group
Cur3	RMX180	116.8	73.53	185.7	a

Model summary

```
Linear mixed model fit by REML. t-tests use Satterthwaite's method ['lmerModLmerTest']
Formula: Halt_frequency_H0_Zone_2 ~ 1 + (1 | Genotype_Zone_1:Plant_Zone_1) +      (1 |
Genotype_Zone_2:Plant_Zone_2)
   Data: data

REML criterion at convergence: 108.3
```

```
Scaled residuals:
    Min      1Q  Median      3Q     Max
-3.5893 -0.2300  0.2751  0.5137  1.0619

Random effects:
 Groups                       Name        Variance  Std.Dev.
 Genotype_Zone_1:Plant_Zone_1 (Intercept) 4.061e-18 2.015e-09
 Genotype_Zone_2:Plant_Zone_2 (Intercept) 1.842e-01 4.292e-01
 Residual                                 8.582e-01 9.264e-01
Number of obs: 38, groups:  Genotype_Zone_1:Plant_Zone_1, 10; Genotype_Zone_2:Plant_Zone_2, 10

Fixed effects:
            Estimate Std. Error     df t value Pr(>|t|)
(Intercept)   4.7609     0.2028 8.4728   23.48 5.23e-09 ***
---
Signif. codes:  0 '***' 0.001 '**' 0.01 '*' 0.05 '.' 0.1 ' ' 1
```

Model residuals

Statistic	Value
Sample skewness	-2.537
Sample excess kurtosis	7.37
Passed Shapiro Wilk normality test	No (p-value = 3.956E-07 < 0.05)

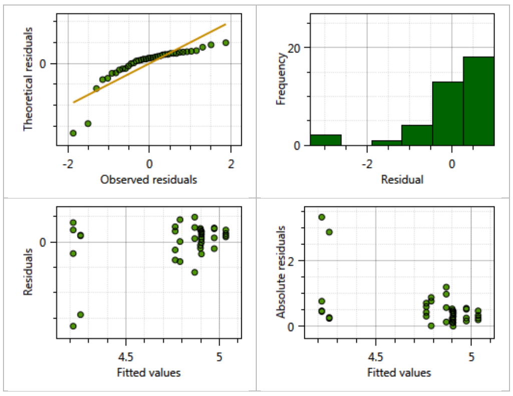

Analysis halt frequency (H1 - Zone 1)

**Analysis model**	Linear mixed model fit by REML: Halt_frequency_H1_Zone_1 ~ 1 + (1|Genotype_Zone_1:Plant_Zone_1) + (1|Genotype_Zone_2:Plant_Zone_2)
**Transformation**	Natural logarithm

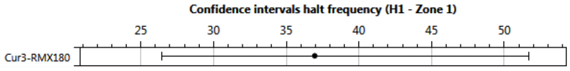

Confidence intervals halt frequency (H1 - Zone 1)

Genotype Zone 1	Genotype Zone 2	Mean	Lower 95% CL	Upper 95% CL	Group
Cur3	RMX180	36.96	26.42	51.7	a

Model summary

```
Linear mixed model fit by REML. t-tests use Satterthwaite's method ['lmerModLmerTest']
Formula: Halt_frequency_H1_Zone_1 ~ 1 + (1 | Genotype_Zone_1:Plant_Zone_1) +      (1 |
Genotype_Zone_2:Plant_Zone_2)
   Data: data

REML criterion at convergence: 102.4

Scaled residuals:
    Min      1Q  Median      3Q     Max
-2.8805 -0.3526  0.1873  0.7406  1.1875

Random effects:
 Groups                       Name        Variance Std.Dev.
 Genotype_Zone_1:Plant_Zone_1 (Intercept) 0.0000   0.0000
 Genotype_Zone_2:Plant_Zone_2 (Intercept) 0.0000   0.0000
 Residual                                 0.9846   0.9923
Number of obs: 36, groups:  Genotype_Zone_1:Plant_Zone_1, 10; Genotype_Zone_2:Plant_Zone_2, 10

Fixed effects:
            Estimate Std. Error      df t value Pr(>|t|)
(Intercept)   3.6097     0.1654 35.0000   21.83   <2e-16 ***
---
Signif. codes:  0 '***' 0.001 '**' 0.01 '*' 0.05 '.' 0.1 ' ' 1
```

Model residuals

Statistic	Value
Sample skewness	-1.257
Sample excess kurtosis	1.202
Passed Shapiro Wilk normality test	No (p-value = 0.001176 < 0.05)

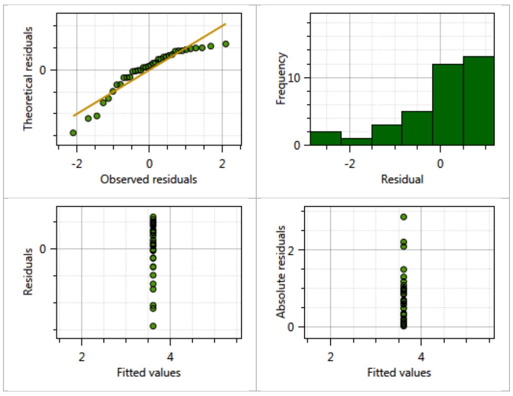

Analysis halt frequency (H1 - Zone 2)

**Analysis model**	Linear mixed model fit by REML: Halt_frequency_H1_Zone_2 ~ 1 + (1|Genotype_Zone_1:Plant_Zone_1) + (1|Genotype_Zone_2:Plant_Zone_2)
**Transformation**	Natural logarithm

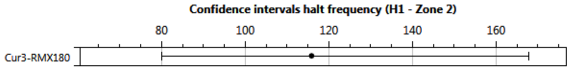

Confidence intervals halt frequency (H1 - Zone 2)

Genotype Zone 1	Genotype Zone 2	Mean	Lower 95% CL	Upper 95% CL	Group
Cur3	RMX180	115.9	79.96	167.9	a

Model summary

```
Linear mixed model fit by REML. t-tests use Satterthwaite's method ['lmerModLmerTest']
Formula: Halt_frequency_H1_Zone_2 ~ 1 + (1 | Genotype_Zone_1:Plant_Zone_1) +      (1 |
Genotype_Zone_2:Plant_Zone_2)
   Data: data

REML criterion at convergence: 117.6

Scaled residuals: 
    Min      1Q  Median      3Q     Max 
-4.2028 -0.3595  0.2161  0.5381  2.3877 

Random effects:
 Groups                       Name        Variance Std.Dev.
 Genotype_Zone_1:Plant_Zone_1 (Intercept) 0.000    0.000   
 Genotype_Zone_2:Plant_Zone_2 (Intercept) 0.000    0.000   
 Residual                                 1.273    1.128   
Number of obs: 38, groups:  Genotype_Zone_1:Plant_Zone_1, 10; Genotype_Zone_2:Plant_Zone_2, 10

Fixed effects:
            Estimate Std. Error      df t value Pr(>|t|)    
(Intercept)   4.7525     0.1831 37.0000   25.96   <2e-16 ***
---
Signif. codes:  0 '***' 0.001 '**' 0.01 '*' 0.05 '.' 0.1 ' ' 1
```

Model residuals

Statistic	Value
Sample skewness	-1.884
Sample excess kurtosis	8.115
Passed Shapiro Wilk normality test	No (p-value = 2.162E-05 < 0.05)

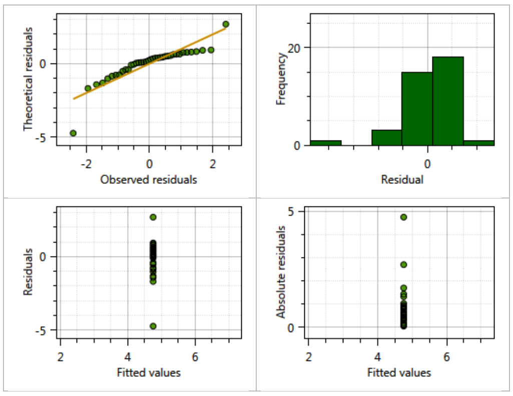

Analysis halt frequency (H2 - Zone 1)

**Analysis model**	Linear mixed model fit by REML: Halt_frequency_H2_Zone_1 ~ 1 + (1|Genotype_Zone_1:Plant_Zone_1) + (1|Genotype_Zone_2:Plant_Zone_2)
**Transformation**	Natural logarithm

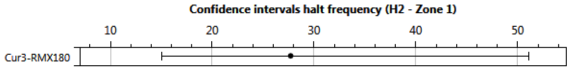

Confidence intervals halt frequency (H2 - Zone 1)

Genotype Zone 1	Genotype Zone 2	Mean	Lower 95% CL	Upper 95% CL	Group
Cur3	RMX180	27.7	15.01	51.12	a

Model summary

```
Linear mixed model fit by REML. t-tests use Satterthwaite's method ['lmerModLmerTest']
Formula: Halt_frequency_H2_Zone_1 ~ 1 + (1 | Genotype_Zone_1:Plant_Zone_1) +      (1 |
Genotype_Zone_2:Plant_Zone_2)
   Data: data

REML criterion at convergence: 109.4

Scaled residuals: 
     Min       1Q   Median       3Q      Max 
-2.18997 -0.52381 -0.00736  0.70586  1.51948 

Random effects:
 Groups                       Name        Variance Std.Dev.
 Genotype_Zone_1:Plant_Zone_1 (Intercept) 0.4395   0.6630  
 Genotype_Zone_2:Plant_Zone_2 (Intercept) 0.0000   0.0000  
 Residual                                 0.9343   0.9666  
Number of obs: 36, groups:  Genotype_Zone_1:Plant_Zone_1, 10; Genotype_Zone_2:Plant_Zone_2, 10

Fixed effects:
            Estimate Std. Error     df t value Pr(>|t|)    
(Intercept)   3.3214     0.2657 7.9891    12.5 1.59e-06 ***
---
Signif. codes:  0 '***' 0.001 '**' 0.01 '*' 0.05 '.' 0.1 ' ' 1
```

Model residuals

Statistic	Value
Sample skewness	-0.37
Sample excess kurtosis	-0.3845
Passed Shapiro Wilk normality test	Yes (p-value = 0.5993 > 0.05)

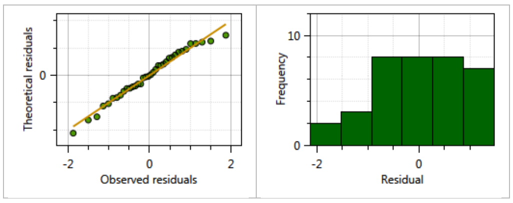

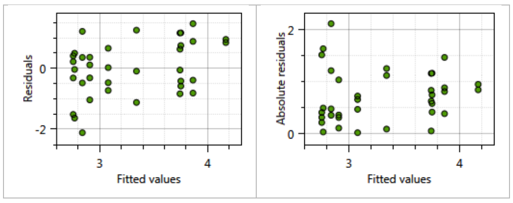

Analysis halt frequency (H2 - Zone 2)

**Analysis model**	Linear mixed model fit by REML: Halt_frequency_H2_Zone_2 ~ 1 + (1|Genotype_Zone_1:Plant_Zone_1) + (1|Genotype_Zone_2:Plant_Zone_2)
**Transformation**	Natural logarithm

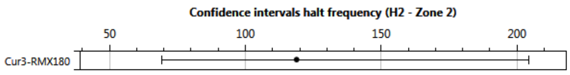

Confidence intervals halt frequency (H2 - Zone 2)

Genotype Zone 1	Genotype Zone 2	Mean	Lower 95% CL	Upper 95% CL	Group
Cur3	RMX180	118.8	69.07	204.5	a

Model summary

```
Linear mixed model fit by REML. t-tests use Satterthwaite's method ['lmerModLmerTest']
Formula: Halt_frequency_H2_Zone_2 ~ 1 + (1 | Genotype_Zone_1:Plant_Zone_1) +      (1 |
Genotype_Zone_2:Plant_Zone_2)
   Data: data

REML criterion at convergence: 117.3

Scaled residuals:
    Min      1Q  Median      3Q     Max
-3.9703 -0.1487  0.4056  0.5393  1.0067

Random effects:
 Groups                       Name        Variance Std.Dev.
 Genotype_Zone_1:Plant_Zone_1 (Intercept) 0.09501  0.3082
 Genotype_Zone_2:Plant_Zone_2 (Intercept) 0.05383  0.2320
 Residual                                 1.13844  1.0670
Number of obs: 38, groups:  Genotype_Zone_1:Plant_Zone_1, 10; Genotype_Zone_2:Plant_Zone_2, 10

Fixed effects:
            Estimate Std. Error     df t value Pr(>|t|)
(Intercept)   4.7778     0.2121 5.0787   22.53 2.76e-06 ***
---
Signif. codes:  0 '***' 0.001 '**' 0.01 '*' 0.05 '.' 0.1 ' ' 1
```

Model residuals

Statistic	Value
Sample skewness	-2.394
Sample excess kurtosis	7.319
Passed Shapiro Wilk normality test	No (p-value = 9.62E-07 < 0.05)

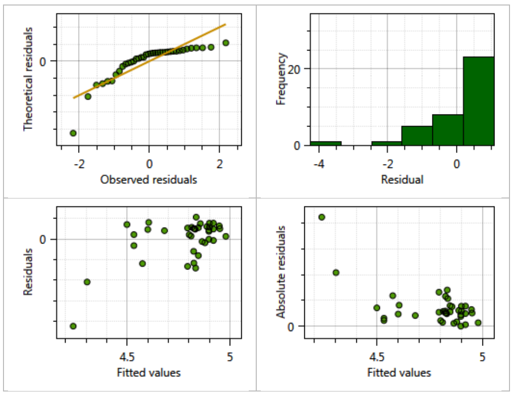

Analysis halt frequency (H3 - Zone 1)

**Analysis model**	Linear mixed model fit by REML: Halt_frequency_H3_Zone_1 ~ 1 + (1|Genotype_Zone_1:Plant_Zone_1) + (1|Genotype_Zone_2:Plant_Zone_2)
**Transformation**	Natural logarithm

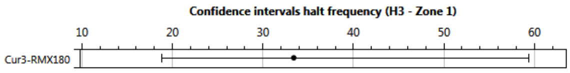

Confidence intervals halt frequency (H3 - Zone 1)

Genotype Zone 1	Genotype Zone 2	Mean	Lower 95% CL	Upper 95% CL	Group
Cur3	RMX180	33.4	18.78	59.4	a

Model summary

```
Linear mixed model fit by REML. t-tests use Satterthwaite's method ['lmerModLmerTest']
Formula: Halt_frequency_H3_Zone_1 ~ 1 + (1 | Genotype_Zone_1:Plant_Zone_1) +      (1 |
Genotype_Zone_2:Plant_Zone_2)
   Data: data

REML criterion at convergence: 108

Scaled residuals:
     Min       1Q   Median       3Q      Max
-2.71802 -0.35683  0.06245  0.71211  1.23960

Random effects:
 Groups                       Name        Variance Std.Dev.
 Genotype_Zone_1:Plant_Zone_1 (Intercept) 0.002235 0.04728
 Genotype_Zone_2:Plant_Zone_2 (Intercept) 0.109772 0.33132
 Residual                                 1.437781 1.19908
Number of obs: 33, groups:  Genotype_Zone_1:Plant_Zone_1, 10; Genotype_Zone_2:Plant_Zone_2, 10

Fixed effects:
            Estimate Std. Error     df t value Pr(>|t|)
(Intercept)   3.5086     0.2351 5.9847   14.92 5.83e-06 ***
---
Signif. codes:  0 '***' 0.001 '**' 0.01 '*' 0.05 '.' 0.1 ' ' 1
```

Model residuals

Statistic	Value
Sample skewness	-1.071

Statistic	Value
Sample excess kurtosis	0.8806
Passed Shapiro Wilk normality test	No (p-value = 0.01406 < 0.05)

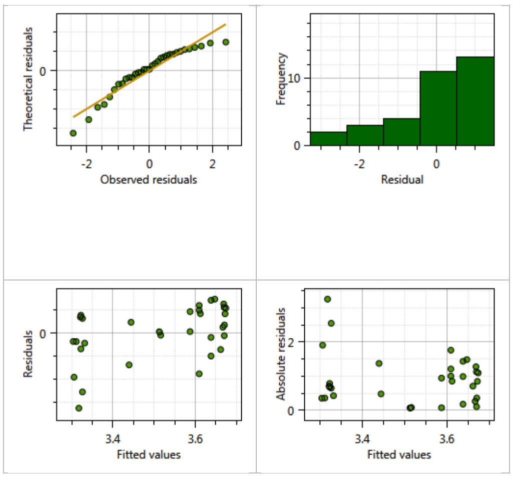

Analysis halt frequency (H3 - Zone 2)

**Analysis model**	Linear mixed model fit by REML: Halt_frequency_H3_Zone_2 ~ 1 + (1|Genotype_Zone_1:Plant_Zone_1) + (1|Genotype_Zone_2:Plant_Zone_2)
**Transformation**	Natural logarithm

Confidence intervals halt frequency (H3 - Zone 2)

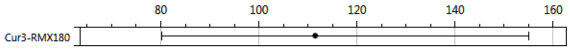

Confidence intervals halt frequency (H3 - Zone 2)

Genotype Zone 1	Genotype Zone 2	Mean	Lower 95% CL	Upper 95% CL	Group
Cur3	RMX180	111.5	80.12	155.1	a

Model summary

```
Linear mixed model fit by REML. t-tests use Satterthwaite's method ['lmerModLmerTest']
Formula: Halt_frequency_H3_Zone_2 ~ 1 + (1 | Genotype_Zone_1:Plant_Zone_1) +      (1 |
Genotype_Zone_2:Plant_Zone_2)
   Data: data

REML criterion at convergence: 103.7

Scaled residuals:
    Min      1Q  Median      3Q     Max
-2.9391 -0.3770  0.2304  0.6896  1.3376

Random effects:
 Groups                       Name        Variance  Std.Dev.
 Genotype_Zone_1:Plant_Zone_1 (Intercept) 1.117e-69 3.343e-35
 Genotype_Zone_2:Plant_Zone_2 (Intercept) 9.663e-05 9.830e-03
 Residual                                 8.132e-01 9.018e-01
Number of obs: 39, groups:  Genotype_Zone_1:Plant_Zone_1, 10; Genotype_Zone_2:Plant_Zone_2, 10

Fixed effects:
            Estimate Std. Error     df t value Pr(>|t|)
(Intercept)   4.7137     0.1444 8.4146   32.63  3.7e-10 ***
---
Signif. codes:  0 '***' 0.001 '**' 0.01 '*' 0.05 '.' 0.1 ' ' 1
```

Model residuals

Statistic	Value
Sample skewness	-1.558
Sample excess kurtosis	2.611
Passed Shapiro Wilk normality test	No (p-value = 0.0001429 < 0.05)

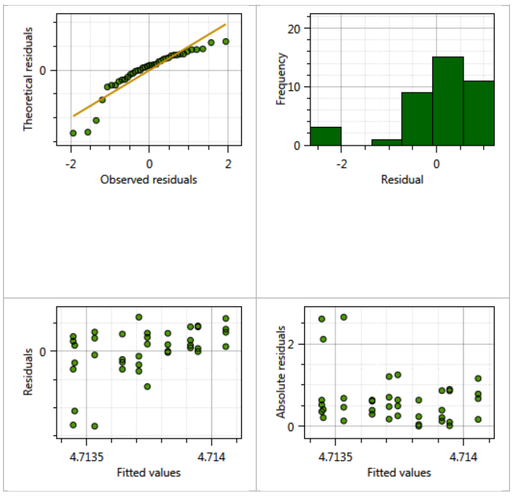

Analysis halt frequency (H4 - Zone 1)

**Analysis model**	Linear mixed model fit by REML: Halt_frequency_H4_Zone_1 ~ 1 + (1|Genotype_Zone_1:Plant_Zone_1) + (1|Genotype_Zone_2:Plant_Zone_2)
**Transformation**	Natural logarithm

Confidence intervals halt frequency (H4 - Zone 1)

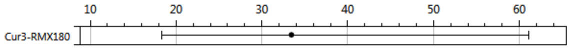

Confidence intervals halt frequency (H4 - Zone 1)

Genotype Zone 1	Genotype Zone 2	Mean	Lower 95% CL	Upper 95% CL	Group
Cur3	RMX180	33.44	18.29	61.13	a

Model summary

```
Linear mixed model fit by REML. t-tests use Satterthwaite's method ['lmerModLmerTest']
```

```
Formula: Halt_frequency_H4_Zone_1 ~ 1 + (1 | Genotype_Zone_1:Plant_Zone_1) +      (1 |
Genotype_Zone_2:Plant_Zone_2)
   Data: data

REML criterion at convergence: 91.3

Scaled residuals:
     Min       1Q   Median       3Q      Max
-2.90185 -0.36488 -0.00732  0.71373  1.54451

Random effects:
 Groups                       Name        Variance Std.Dev.
 Genotype_Zone_1:Plant_Zone_1 (Intercept) 0.2266   0.4761
 Genotype_Zone_2:Plant_Zone_2 (Intercept) 0.0000   0.0000
 Residual                                 1.1792   1.0859
Number of obs: 29, groups:  Genotype_Zone_1:Plant_Zone_1, 10; Genotype_Zone_2:Plant_Zone_2, 10

Fixed effects:
            Estimate Std. Error     df t value Pr(>|t|)
(Intercept)   3.5098     0.2558 7.0903   13.72  2.3e-06 ***
---
Signif. codes:  0 '***' 0.001 '**' 0.01 '*' 0.05 '.' 0.1 ' ' 1
```

Model residuals

Statistic	Value
Sample skewness	-1.12
Sample excess kurtosis	2.116
Passed Shapiro Wilk normality test	No (p-value = 0.03542 < 0.05)

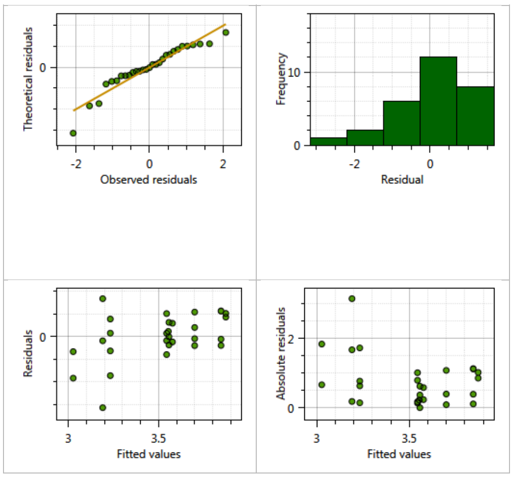

Analysis halt frequency (H4 - Zone 2)

**Analysis model**	Linear mixed model fit by REML: Halt_frequency_H4_Zone_2 ~ 1 + (1|Genotype_Zone_1:Plant_Zone_1) + (1|Genotype_Zone_2:Plant_Zone_2)
**Transformation**	Natural logarithm

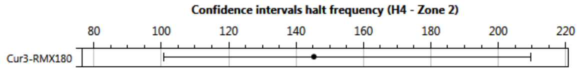

Confidence intervals halt frequency (H4 - Zone 2)

Genotype Zone 1	Genotype Zone 2	Mean	Lower 95% CL	Upper 95% CL	Group
Cur3	RMX180	145.3	100.6	209.7	a

Model summary

```
Linear mixed model fit by REML. t-tests use Satterthwaite's method ['lmerModLmerTest']
Formula: Halt_frequency_H4_Zone_2 ~ 1 + (1 | Genotype_Zone_1:Plant_Zone_1) +      (1 |
Genotype_Zone_2:Plant_Zone_2)
   Data: data

REML criterion at convergence: 87.9

Scaled residuals:
    Min      1Q  Median      3Q     Max
-3.0567 -0.5098  0.3280  0.6061  1.1673

Random effects:
 Groups                       Name        Variance Std.Dev.
 Genotype_Zone_1:Plant_Zone_1 (Intercept) 0.03978  0.1995
 Genotype_Zone_2:Plant_Zone_2 (Intercept) 0.06790  0.2606
 Residual                                 0.52178  0.7223
Number of obs: 37, groups:  Genotype_Zone_1:Plant_Zone_1, 10; Genotype_Zone_2:Plant_Zone_2, 10

Fixed effects:
            Estimate Std. Error     df t value Pr(>|t|)
(Intercept)   4.9787     0.1584 7.7589   31.44 1.84e-09 ***
---
Signif. codes:  0 '***' 0.001 '**' 0.01 '*' 0.05 '.' 0.1 ' ' 1
```

Model residuals

Statistic	Value
Sample skewness	-1.381
Sample excess kurtosis	2.185
Passed Shapiro Wilk normality test	No (p-value = 0.001132 < 0.05)

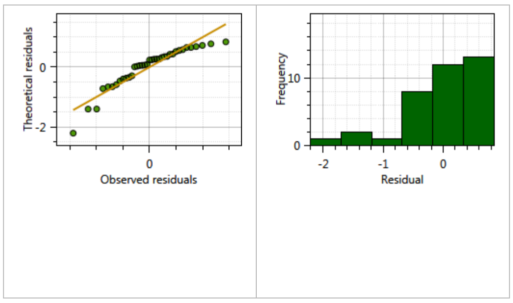

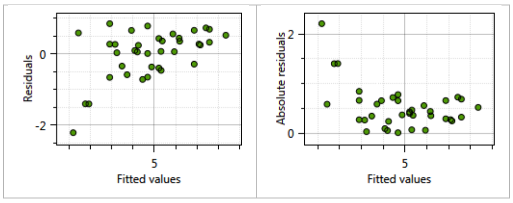

Analysis halt frequency (H5 - Zone 1)

**Analysis model**	Linear mixed model fit by REML: Halt_frequency_H5_Zone_1 ~ 1 + (1|Genotype_Zone_1:Plant_Zone_1) + (1|Genotype_Zone_2:Plant_Zone_2)
**Transformation**	Natural logarithm

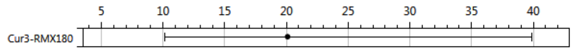

Confidence intervals halt frequency (H5 - Zone 1)

Genotype Zone 1	Genotype Zone 2	Mean	Lower 95% CL	Upper 95% CL	Group
Cur3	RMX180	20.09	10.11	39.9	a

Model summary

```
Linear mixed model fit by REML. t-tests use Satterthwaite's method ['lmerModLmerTest']
Formula: Halt_frequency_H5_Zone_1 ~ 1 + (1 | Genotype_Zone_1:Plant_Zone_1) +      (1 |
Genotype_Zone_2:Plant_Zone_2)
   Data: data

REML criterion at convergence: 113.7

Scaled residuals:
    Min      1Q  Median      3Q     Max
-1.9857 -0.5769  0.2693  0.7196  1.2692

Random effects:
 Groups                       Name        Variance Std.Dev.
 Genotype_Zone_1:Plant_Zone_1 (Intercept) 0.2656   0.5154
 Genotype_Zone_2:Plant_Zone_2 (Intercept) 0.0000   0.0000
 Residual                                 2.0962   1.4478
Number of obs: 31, groups:  Genotype_Zone_1:Plant_Zone_1, 10; Genotype_Zone_2:Plant_Zone_2, 10

Fixed effects:
            Estimate Std. Error      df t value Pr(>|t|)
(Intercept)   3.0001     0.3085 10.1349   9.724 1.85e-06 ***
---
Signif. codes:  0 '***' 0.001 '**' 0.01 '*' 0.05 '.' 0.1 ' ' 1
```

Model residuals

Statistic	Value
Sample skewness	-0.7741
Sample excess kurtosis	-0.5479
Passed Shapiro Wilk normality test	No (p-value = 0.008469 < 0.05)

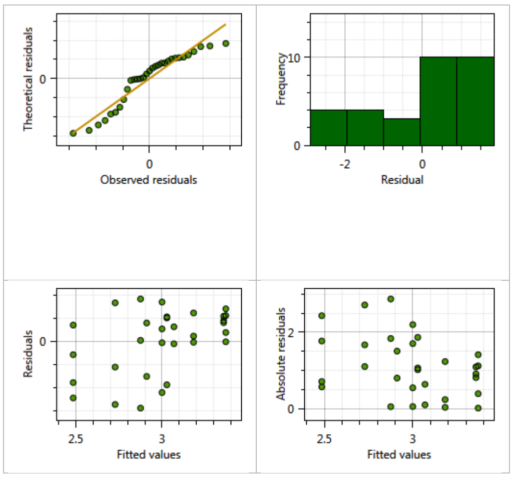

Analysis halt frequency (H5 - Zone 2)

**Analysis model**	Linear mixed model fit by REML: Halt_frequency_H5_Zone_2 ~ 1 + (1|Genotype_Zone_1:Plant_Zone_1) + (1|Genotype_Zone_2:Plant_Zone_2)
**Transformation**	Natural logarithm

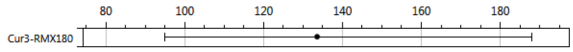

Confidence intervals halt frequency (H5 - Zone 2)

Genotype Zone 1	Genotype Zone 2	Mean	Lower 95% CL	Upper 95% CL	Group
Cur3	RMX180	133.6	94.85	188.1	a

Model summary

```
Linear mixed model fit by REML. t-tests use Satterthwaite's method ['lmerModLmerTest']
Formula: Halt_frequency_H5_Zone_2 ~ 1 + (1 | Genotype_Zone_1:Plant_Zone_1) +      (1 |
Genotype_Zone_2:Plant_Zone_2)
   Data: data

REML criterion at convergence: 95.2

Scaled residuals:
    Min      1Q  Median      3Q     Max
-2.3102 -0.5327  0.1638  0.5980  1.3298

Random effects:
 Groups                       Name        Variance Std.Dev.
 Genotype_Zone_1:Plant_Zone_1 (Intercept) 0.00000  0.0000
 Genotype_Zone_2:Plant_Zone_2 (Intercept) 0.07791  0.2791
 Residual                                 0.59014  0.7682
Number of obs: 39, groups:  Genotype_Zone_1:Plant_Zone_1, 10; Genotype_Zone_2:Plant_Zone_2, 10

Fixed effects:
            Estimate Std. Error     df t value Pr(>|t|)
(Intercept)   4.8946     0.1515 9.0796    32.3  1.1e-10 ***
---
Signif. codes:  0 '***' 0.001 '**' 0.01 '*' 0.05 '.' 0.1 ' ' 1
```

Model residuals

Statistic	Value
Sample skewness	-0.88
Sample excess kurtosis	0.1789
Passed Shapiro Wilk normality test	No (p-value = 0.01112 < 0.05)

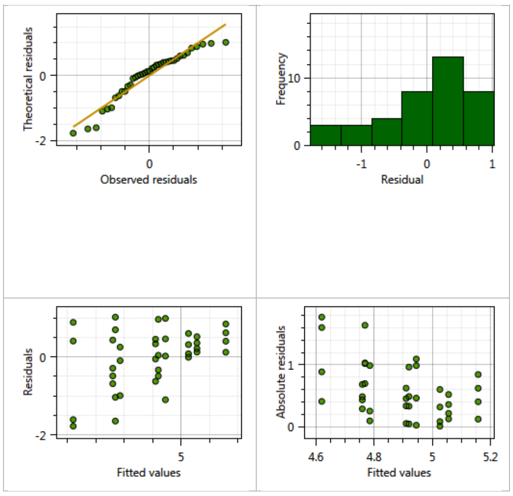

Analysis halt frequency (H6 - Zone 1)

**Analysis model**	Linear mixed model fit by REML: Halt_frequency_H6_Zone_1 ~ 1 + (1|Genotype_Zone_1:Plant_Zone_1) + (1|Genotype_Zone_2:Plant_Zone_2)
**Transformation**	Natural logarithm

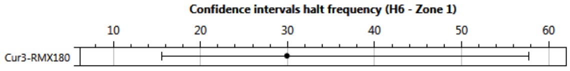

Confidence intervals halt frequency (H6 - Zone 1)

Genotype Zone 1	Genotype Zone 2	Mean	Lower 95% CL	Upper 95% CL	Group
Cur3	RMX180	29.94	15.52	57.75	a

Model summary

```
Linear mixed model fit by REML. t-tests use Satterthwaite's method ['lmerModLmerTest']
Formula: Halt_frequency_H6_Zone_1 ~ 1 + (1 | Genotype_Zone_1:Plant_Zone_1) +      (1 |
Genotype_Zone_2:Plant_Zone_2)
   Data: data

REML criterion at convergence: 83.9

Scaled residuals:
    Min      1Q  Median      3Q     Max
-1.9041 -0.3843  0.2775  0.5110  1.4848

Random effects:
 Groups                       Name        Variance Std.Dev.
 Genotype_Zone_1:Plant_Zone_1 (Intercept) 0.1184   0.3441
 Genotype_Zone_2:Plant_Zone_2 (Intercept) 0.2090   0.4572
 Residual                                 1.0371   1.0184
Number of obs: 27, groups:  Genotype_Zone_1:Plant_Zone_1, 10; Genotype_Zone_2:Plant_Zone_2, 10

Fixed effects:
            Estimate Std. Error     df t value Pr(>|t|)
(Intercept)   3.3991     0.2728 6.4209   12.46 9.77e-06 ***
---
Signif. codes:  0 '***' 0.001 '**' 0.01 '*' 0.05 '.' 0.1 ' ' 1
```

Model residuals

Statistic	Value
Sample skewness	-0.6552
Sample excess kurtosis	0.05336
Passed Shapiro Wilk normality test	Yes (p-value = 0.07888 > 0.05)

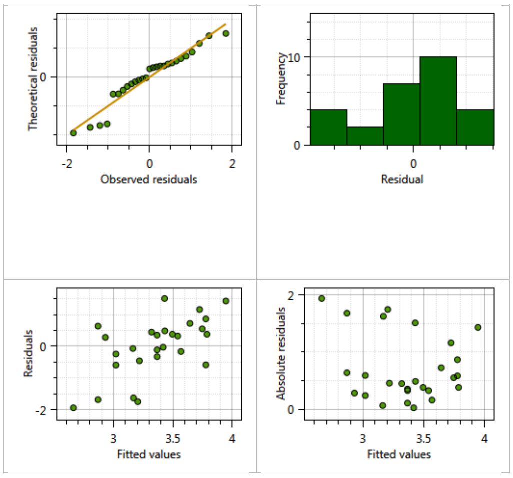

Analysis halt frequency (H6 - Zone 2)

**Analysis model**	Linear mixed model fit by REML: Halt_frequency_H6_Zone_2 ~ 1 + (1|Genotype_Zone_1:Plant_Zone_1) + (1|Genotype_Zone_2:Plant_Zone_2)
**Transformation**	Natural logarithm

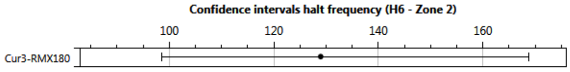

Confidence intervals halt frequency (H6 - Zone 2)

Genotype Zone 1	Genotype Zone 2	Mean	Lower 95% CL	Upper 95% CL	Group
Cur3	RMX180	129	98.5	168.8	a

Model summary

```
Linear mixed model fit by REML. t-tests use Satterthwaite's method ['lmerModLmerTest']
Formula: Halt_frequency_H6_Zone_2 ~ 1 + (1 | Genotype_Zone_1:Plant_Zone_1) +      (1 |
Genotype_Zone_2:Plant_Zone_2)
   Data: data

REML criterion at convergence: 93.9

Scaled residuals:
    Min      1Q  Median      3Q     Max
-3.2038 -0.3594 -0.0172  0.8064  1.3676

Random effects:
 Groups                       Name        Variance Std.Dev.
 Genotype_Zone_1:Plant_Zone_1 (Intercept) 0.000    0.0000
 Genotype_Zone_2:Plant_Zone_2 (Intercept) 0.000    0.0000
 Residual                                 0.672    0.8197
Number of obs: 38, groups:  Genotype_Zone_1:Plant_Zone_1, 10; Genotype_Zone_2:Plant_Zone_2, 10

Fixed effects:
            Estimate Std. Error     df t value Pr(>|t|)
(Intercept)    4.859      0.133 37.000   36.54   <2e-16 ***
---
Signif. codes:  0 '***' 0.001 '**' 0.01 '*' 0.05 '.' 0.1 ' ' 1
```

Model residuals

Statistic	Value
Sample skewness	-1.147
Sample excess kurtosis	2.169
Passed Shapiro Wilk normality test	No (p-value = 0.006195 < 0.05)

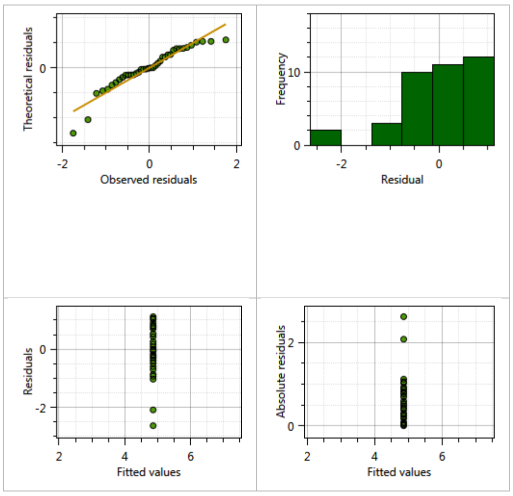

Analysis halt frequency (H7 - Zone 1)

**Analysis model**	Linear mixed model fit by REML: Halt_frequency_H7_Zone_1 ~ 1 + (1|Genotype_Zone_1:Plant_Zone_1) + (1|Genotype_Zone_2:Plant_Zone_2)
**Transformation**	Natural logarithm

Confidence intervals halt frequency (H7 - Zone 1)

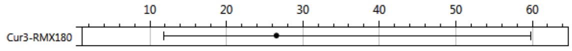

Confidence intervals halt frequency (H7 - Zone 1)

Genotype Zone 1	Genotype Zone 2	Mean	Lower 95% CL	Upper 95% CL	Group
Cur3	RMX180	26.54	11.77	59.83	a

Model summary

```
Linear mixed model fit by REML. t-tests use Satterthwaite's method ['lmerModLmerTest']
Formula: Halt_frequency_H7_Zone_1 ~ 1 + (1 | Genotype_Zone_1:Plant_Zone_1) +      (1 |
Genotype_Zone_2:Plant_Zone_2)
   Data: data

REML criterion at convergence: 92.4

Scaled residuals:
    Min      1Q  Median      3Q     Max
-1.9434 -0.5607  0.1414  0.5312  1.9558

Random effects:
 Groups                       Name        Variance Std.Dev.
 Genotype_Zone_2:Plant_Zone_2 (Intercept) 0.02285  0.1512
 Genotype_Zone_1:Plant_Zone_1 (Intercept) 0.27690  0.5262
 Residual                                 1.57011  1.2530
Number of obs: 27, groups:  Genotype_Zone_2:Plant_Zone_2, 10; Genotype_Zone_1:Plant_Zone_1, 9

Fixed effects:
            Estimate Std. Error     df t value Pr(>|t|)
(Intercept)   3.2785     0.3052 4.4763   10.74 0.000231 ***
---
Signif. codes:  0 '***' 0.001 '**' 0.01 '*' 0.05 '.' 0.1 ' ' 1
```

Model residuals

Statistic	Value
Sample skewness	-0.2435
Sample excess kurtosis	-0.1607
Passed Shapiro Wilk normality test	Yes (p-value = 0.5984 > 0.05)

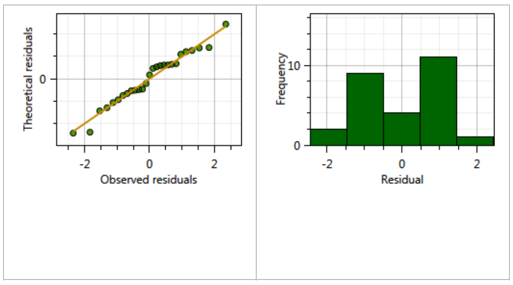

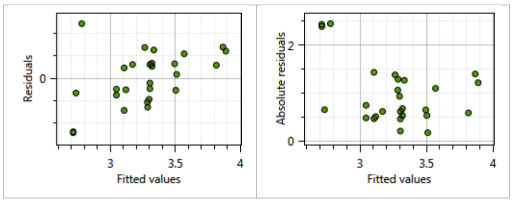

Analysis halt frequency (H7 - Zone 2)

**Analysis model**	Linear mixed model fit by REML: Halt_frequency_H7_Zone_2 ~ 1 + (1|Genotype_Zone_1:Plant_Zone_1) + (1|Genotype_Zone_2:Plant_Zone_2)
**Transformation**	Natural logarithm

Confidence intervals halt frequency (H7 - Zone 2)

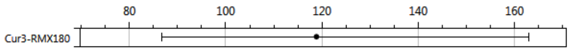

Confidence intervals halt frequency (H7 - Zone 2)

Genotype Zone 1	Genotype Zone 2	Mean	Lower 95% CL	Upper 95% CL	Group
Cur3	RMX180	118.9	86.65	163	a

Model summary

```
Linear mixed model fit by REML. t-tests use Satterthwaite's method ['lmerModLmerTest']
Formula: Halt_frequency_H7_Zone_2 ~ 1 + (1 | Genotype_Zone_1:Plant_Zone_1) +      (1 |
Genotype_Zone_2:Plant_Zone_2)
   Data: data

REML criterion at convergence: 97.8

Scaled residuals:
    Min      1Q  Median      3Q     Max
-2.0358 -0.8320  0.2636  0.7630  1.4173

Random effects:
 Groups                       Name        Variance Std.Dev.
 Genotype_Zone_1:Plant_Zone_1 (Intercept) 0.00000  0.0000
 Genotype_Zone_2:Plant_Zone_2 (Intercept) 0.02265  0.1505
 Residual                                 0.67721  0.8229
Number of obs: 39, groups:  Genotype_Zone_1:Plant_Zone_1, 10; Genotype_Zone_2:Plant_Zone_2, 10

Fixed effects:
            Estimate Std. Error     df t value Pr(>|t|)
(Intercept)   4.7779     0.1401 9.1786   34.09 5.56e-11 ***
---
Signif. codes:  0 '***' 0.001 '**' 0.01 '*' 0.05 '.' 0.1 ' ' 1
```

Model residuals

Statistic	Value
Sample skewness	-0.5205
Sample excess kurtosis	-0.8435
Passed Shapiro Wilk normality test	No (p-value = 0.03017 < 0.05)

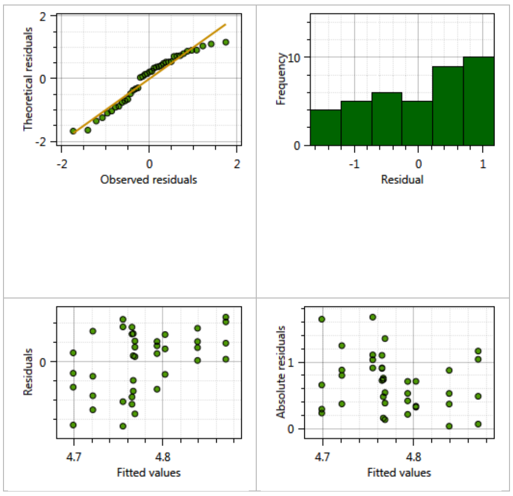

Analysis halt frequency H0 (diff. Zone 1 - Zone 2)

**Analysis model**	Generalized linear mixed model with dispersion factor, formula=cbind(Halt_frequency_H0_Zone_1,Halt_frequency_H0_Zone_2) ~ 1 + (1|Genotype_Zone_1:Plant_Zone_1) + (1|Genotype_Zone_2:Plant_Zone_2)
**Transformation**	Logit

Confidence intervals halt frequency H0 (diff. Zone 1 - Zone 2)

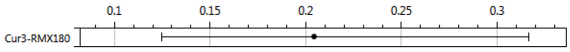

Confidence intervals halt frequency H0 (diff. Zone 1 - Zone 2)

Genotype Zone 1	Genotype Zone 2	Mean	Lower 95% CL	Upper 95% CL	Group
Cur3	RMX180	0.2043	0.1246	0.3166	a

Model summary

```
Linear mixed model fit by REML. t-tests use Satterthwaite's method ['lmerModLmerTest']
Formula: ziFormula
   Data: data
Weights: wi

REML criterion at convergence: 139.4

Scaled residuals:
     Min       1Q   Median       3Q      Max
-1.27002 -0.72518 -0.05042  0.52532  2.83511

Random effects:
 Groups                       Name        Variance Std.Dev.
 Genotype_Zone_1:Plant_Zone_1 (Intercept)  0.0000  0.0000
 Genotype_Zone_2:Plant_Zone_2 (Intercept)  0.2348  0.4846
 Residual                                 50.0923  7.0776
Number of obs: 39, groups:  Genotype_Zone_1:Plant_Zone_1, 10; Genotype_Zone_2:Plant_Zone_2, 10

Fixed effects:
            Estimate Std. Error      df t value Pr(>|t|)
(Intercept)  -1.3595     0.2589  8.5544  -5.252 0.000622 ***
---
```

```
Signif. codes:  0 '***' 0.001 '**' 0.01 '*' 0.05 '.' 0.1 ' ' 1

Dispersion: 7.078
```

Model residuals

Statistic	Value
Sample skewness	1.025
Sample excess kurtosis	0.9694
Passed Shapiro Wilk normality test	No (p-value = 0.01179 < 0.05)

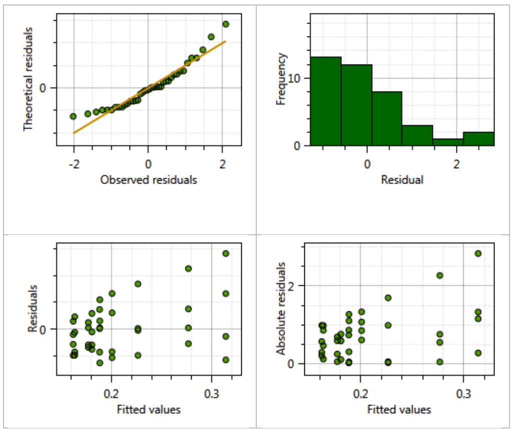

Analysis halt frequency H1 (diff. Zone 1 - Zone 2)

**Analysis model**	Generalized linear mixed model with dispersion factor, formula=cbind(Halt_frequency_H1_Zone_1,Halt_frequency_H1_Zone_2) ~ 1 + (1|Genotype_Zone_1:Plant_Zone_1) + (1|Genotype_Zone_2:Plant_Zone_2)
**Transformation**	Logit

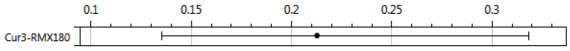

Confidence intervals halt frequency H1 (diff. Zone 1 - Zone 2)

Genotype Zone 1	Genotype Zone 2	Mean	Lower 95% CL	Upper 95% CL	Group
Cur3	RMX180	0.2127	0.1351	0.3185	a

Model summary

```
Linear mixed model fit by REML. t-tests use Satterthwaite's method ['lmerModLmerTest']
Formula: ziFormula
   Data: data
Weights: wi

REML criterion at convergence: 149.3

Scaled residuals: 
     Min       1Q   Median       3Q      Max 
-2.13745 -0.49998 -0.01474  1.02042  1.98771 

Random effects:
 Groups                      Name        Variance  Std.Dev. 
 Genotype_Zone_1:Plant_Zone_1 (Intercept) 6.121e-02 2.474e-01
 Genotype_Zone_2:Plant_Zone_2 (Intercept) 1.833e-16 1.354e-08
 Residual                                8.416e+01 9.174e+00
Number of obs: 39, groups:  Genotype_Zone_1:Plant_Zone_1, 10; Genotype_Zone_2:Plant_Zone_2, 10

Fixed effects:
            Estimate Std. Error      df t value Pr(>|t|)    
(Intercept)  -1.3088     0.2535 12.9413  -5.162 0.000185 ***
---
Signif. codes:  0 '***' 0.001 '**' 0.01 '*' 0.05 '.' 0.1 ' ' 1

Dispersion: 9.174
```

Model residuals

Statistic	Value
Sample skewness	0.1158
Sample excess kurtosis	-0.4971
Passed Shapiro Wilk normality test	Yes (p-value = 0.1552 > 0.05)

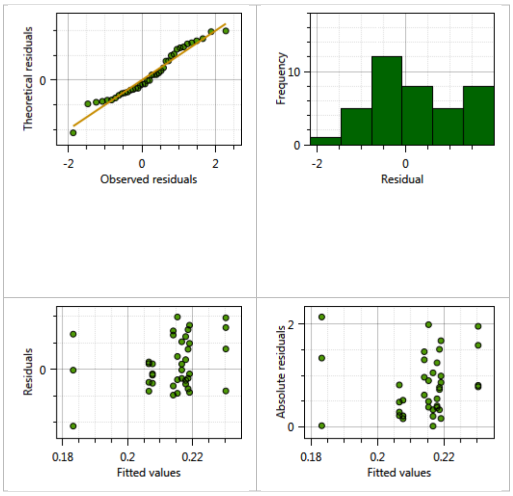

Analysis halt frequency H2 (diff. Zone 1 - Zone 2)

**Analysis model**	Generalized linear mixed model with dispersion factor, formula=cbind(Halt_frequency_H2_Zone_1,Halt_frequency_H2_Zone_2) ~ 1 + (1|Genotype_Zone_1:Plant_Zone_1) + (1|Genotype_Zone_2:Plant_Zone_2)
**Transformation**	Logit

Confidence intervals halt frequency H2 (diff. Zone 1 - Zone 2)

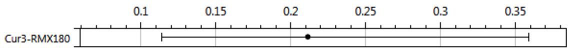

Confidence intervals halt frequency H2 (diff. Zone 1 - Zone 2)

Genotype Zone 1	Genotype Zone 2	Mean	Lower 95% CL	Upper 95% CL	Group
Cur3	RMX180	0.2113	0.1136	0.359	a

Model summary

```
Linear mixed model fit by REML. t-tests use Satterthwaite's method ['lmerModLmerTest']
Formula: ziFormula
   Data: data
Weights: wi

REML criterion at convergence: 147.4

Scaled residuals:
    Min      1Q  Median      3Q     Max
-1.2856 -0.6482 -0.3098  0.5937  2.6035

Random effects:
 Groups                          Name        Variance Std.Dev.
 Genotype_Zone_1:Plant_Zone_1 (Intercept)  0.5276  0.7263
 Genotype_Zone_2:Plant_Zone_2 (Intercept)  0.0000  0.0000
 Residual                                 64.3852  8.0240
Number of obs: 39, groups:  Genotype_Zone_1:Plant_Zone_1, 10; Genotype_Zone_2:Plant_Zone_2, 10

Fixed effects:
            Estimate Std. Error      df t value Pr(>|t|)
(Intercept)  -1.3170     0.3228  8.4640  -4.079  0.00314 **
---
Signif. codes:  0 '***' 0.001 '**' 0.01 '*' 0.05 '.' 0.1 ' ' 1

Dispersion: 8.024
```

Model residuals

Statistic	Value
Sample skewness	0.9917
Sample excess kurtosis	0.4151
Passed Shapiro Wilk normality test	No (p-value = 0.005248 < 0.05)

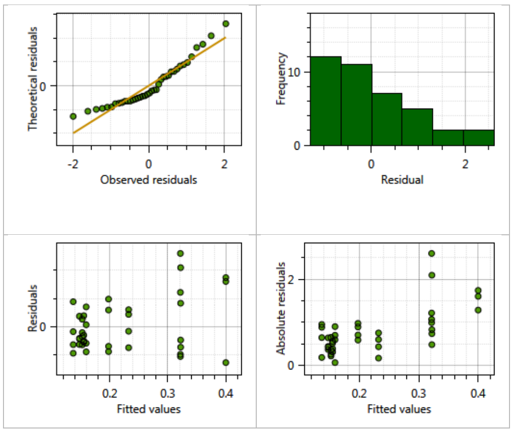

Analysis halt frequency H3 (diff. Zone 1 - Zone 2)

**Analysis model**	Generalized linear mixed model with dispersion factor, formula=cbind(Halt_frequency_H3_Zone_1,Halt_frequency_H3_Zone_2) ~ 1 + (1|Genotype_Zone_1:Plant_Zone_1) + (1|Genotype_Zone_2:Plant_Zone_2)
**Transformation**	Logit

Confidence intervals halt frequency H3 (diff. Zone 1 - Zone 2)

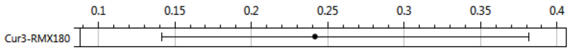

Confidence intervals halt frequency H3 (diff. Zone 1 - Zone 2)

Genotype Zone 1	Genotype Zone 2	Mean	Lower 95% CL	Upper 95% CL	Group
Cur3	RMX180	0.2416	0.1412	0.3817	a

Model summary

```
Linear mixed model fit by REML. t-tests use Satterthwaite's method ['lmerModLmerTest']
Formula: ziFormula
   Data: data
Weights: wi

REML criterion at convergence: 141.6

Scaled residuals:
    Min      1Q  Median      3Q     Max
-1.2755 -0.7261 -0.1318  0.8153  2.1030

Random effects:
 Groups                          Name        Variance Std.Dev.
 Genotype_Zone_1:Plant_Zone_1 (Intercept)  0.0000  0.0000
 Genotype_Zone_2:Plant_Zone_2 (Intercept)  0.3856  0.6209
 Residual                                 61.4165  7.8369
Number of obs: 39, groups:  Genotype_Zone_1:Plant_Zone_1, 10; Genotype_Zone_2:Plant_Zone_2, 10

Fixed effects:
            Estimate Std. Error      df t value Pr(>|t|)
(Intercept)  -1.1440     0.2923  8.9520  -3.914  0.00358 **
---
Signif. codes:  0 '***' 0.001 '**' 0.01 '*' 0.05 '.' 0.1 ' ' 1

Dispersion: 7.837
```

Model residuals

Statistic	Value
Sample skewness	0.4843
Sample excess kurtosis	-0.7841
Passed Shapiro Wilk normality test	No (p-value = 0.03065 < 0.05)

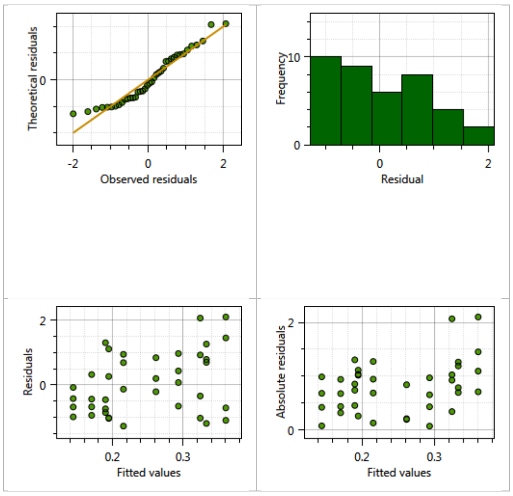

Analysis halt frequency H4 (diff. Zone 1 - Zone 2)

**Analysis model**	Generalized linear mixed model with dispersion factor, formula=cbind(Halt_frequency_H4_Zone_1,Halt_frequency_H4_Zone_2) ~ 1 + (1|Genotype_Zone_1:Plant_Zone_1) + (1|Genotype_Zone_2:Plant_Zone_2)
**Transformation**	Logit

Confidence intervals halt frequency H4 (diff. Zone 1 - Zone 2)

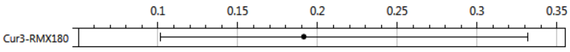

Confidence intervals halt frequency H4 (diff. Zone 1 - Zone 2)

Genotype Zone 1	Genotype Zone 2	Mean	Lower 95% CL	Upper 95% CL	Group
Cur3	RMX180	0.1914	0.1013	0.3319	a

Model summary

```
Linear mixed model fit by REML. t-tests use Satterthwaite's method ['lmerModLmerTest']
Formula: ziFormula
   Data: data
Weights: wi

REML criterion at convergence: 153.6

Scaled residuals: 
    Min      1Q  Median      3Q     Max 
-0.9677 -0.6909 -0.3162  0.6960  2.4941 

Random effects:
 Groups                      Name        Variance Std.Dev.
 Genotype_Zone_1:Plant_Zone_1 (Intercept)  0.2155  0.4642 
 Genotype_Zone_2:Plant_Zone_2 (Intercept)  0.0603  0.2456 
 Residual                              84.0930  9.1702 
Number of obs: 39, groups:  Genotype_Zone_1:Plant_Zone_1, 10; Genotype_Zone_2:Plant_Zone_2, 10

Fixed effects:
            Estimate Std. Error      df t value Pr(>|t|)   
(Intercept)  -1.4411     0.3087  6.4940  -4.669  0.00279 **
---
Signif. codes:  0 '***' 0.001 '**' 0.01 '*' 0.05 '.' 0.1 ' ' 1

Dispersion: 9.17
```

Model residuals

Statistic	Value
Sample skewness	0.9875
Sample excess kurtosis	-0.05196
Passed Shapiro Wilk normality test	No (p-value = 0.0004254 < 0.05)

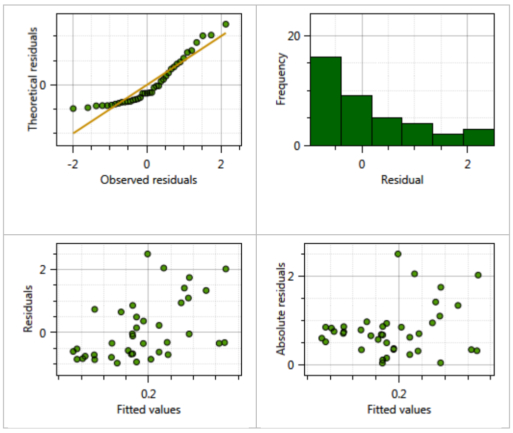

Analysis halt frequency H5 (diff. Zone 1 - Zone 2)

**Analysis model**	Generalized linear mixed model with dispersion factor, formula=cbind(Halt_frequency_H5_Zone_1,Halt_frequency_H5_Zone_2) ~ 1 + (1|Genotype_Zone_1:Plant_Zone_1) + (1|Genotype_Zone_2:Plant_Zone_2)
**Transformation**	Logit

Confidence intervals halt frequency H5 (diff. Zone 1 - Zone 2)

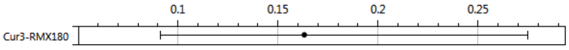

Confidence intervals halt frequency H5 (diff. Zone 1 - Zone 2)

Genotype Zone 1	Genotype Zone 2	Mean	Lower 95% CL	Upper 95% CL	Group
Cur3	RMX180	0.1631	0.09108	0.2749	a

Model summary

```
Linear mixed model fit by REML. t-tests use Satterthwaite's method ['lmerModLmerTest']
Formula: ziFormula
   Data: data
Weights: wi
```

```
REML criterion at convergence: 155

Scaled residuals:
    Min      1Q  Median      3Q     Max
-0.9713 -0.6835 -0.2139  0.6619  2.3546

Random effects:
 Groups                       Name        Variance Std.Dev.
 Genotype_Zone_1:Plant_Zone_1 (Intercept)  0.0000  0.0000
 Genotype_Zone_2:Plant_Zone_2 (Intercept)  0.1388  0.3725
 Residual                              77.8072  8.8208
Number of obs: 39, groups:  Genotype_Zone_1:Plant_Zone_1, 10; Genotype_Zone_2:Plant_Zone_2, 10

Fixed effects:
            Estimate Std. Error      df t value Pr(>|t|)
(Intercept)  -1.6352     0.2946  9.0936  -5.551 0.000343 ***
---
Signif. codes:  0 '***' 0.001 '**' 0.01 '*' 0.05 '.' 0.1 ' ' 1

Dispersion: 8.821
```

Model residuals

Statistic	Value
Sample skewness	0.9078
Sample excess kurtosis	-0.3529
Passed Shapiro Wilk normality test	No (p-value = 0.0003709 < 0.05)

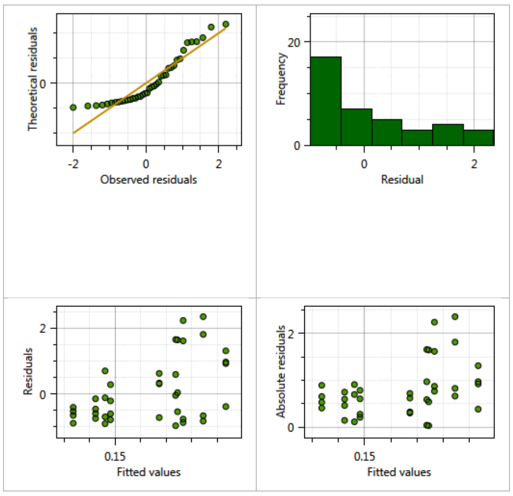

Analysis halt frequency H6 (diff. Zone 1 - Zone 2)

**Analysis model**	Generalized linear mixed model with dispersion factor, formula=cbind(Halt_frequency_H6_Zone_1,Halt_frequency_H6_Zone_2) ~ 1 + (1|Genotype_Zone_1:Plant_Zone_1) + (1|Genotype_Zone_2:Plant_Zone_2)
**Transformation**	Logit

Confidence intervals halt frequency H6 (diff. Zone 1 - Zone 2)

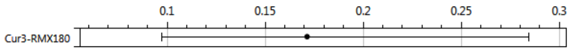

Confidence intervals halt frequency H6 (diff. Zone 1 - Zone 2)

Genotype Zone 1	Genotype Zone 2	Mean	Lower 95% CL	Upper 95% CL	Group
Cur3	RMX180	0.1714	0.0971	0.2845	a

Model summary

```
Linear mixed model fit by REML. t-tests use Satterthwaite's method ['lmerModLmerTest']
Formula: ziFormula
   Data: data
Weights: wi

REML criterion at convergence: 159.2

Scaled residuals:
    Min      1Q  Median      3Q     Max
-1.0286 -0.6773 -0.0583  0.4813  3.2221

Random effects:
 Groups                       Name        Variance Std.Dev.
 Genotype_Zone_1:Plant_Zone_1 (Intercept)  0.00000 0.0000
 Genotype_Zone_2:Plant_Zone_2 (Intercept)  0.09222 0.3037
 Residual                              84.27056 9.1799
Number of obs: 39, groups:  Genotype_Zone_1:Plant_Zone_1, 10; Genotype_Zone_2:Plant_Zone_2, 10

Fixed effects:
            Estimate Std. Error      df t value Pr(>|t|)
(Intercept)  -1.5759     0.2956 10.5643  -5.331 0.000276 ***
---
Signif. codes:  0 '***' 0.001 '**' 0.01 '*' 0.05 '.' 0.1 ' ' 1

Dispersion: 9.18
```

Model residuals

Statistic	Value
Sample skewness	1.32
Sample excess kurtosis	1.82
Passed Shapiro Wilk normality test	No (p-value = 0.000616 < 0.05)

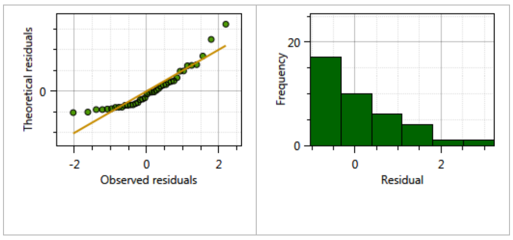

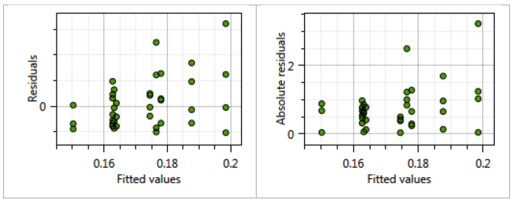

Data points with high residuals

Trial	Arena
Trial 3	11

Analysis halt frequency H7 (diff. Zone 1 - Zone 2)

**Analysis model**	Generalized linear mixed model with dispersion factor, formula=cbind(Halt_frequency_H7_Zone_1,Halt_frequency_H7_Zone_2) ~ 1 + (1|Genotype_Zone_1:Plant_Zone_1) + (1|Genotype_Zone_2:Plant_Zone_2)
**Transformation**	Logit

Confidence intervals halt frequency H7 (diff. Zone 1 - Zone 2)

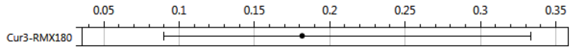

Confidence intervals halt frequency H7 (diff. Zone 1 - Zone 2)

Genotype Zone 1	Genotype Zone 2	Mean	Lower 95% CL	Upper 95% CL	Group
Cur3	RMX180	0.1816	0.08959	0.3336	a

Model summary

```
Linear mixed model fit by REML. t-tests use Satterthwaite's method ['lmerModLmerTest']
Formula: ziFormula
   Data: data
Weights: wi

REML criterion at convergence: 159.7

Scaled residuals:
    Min      1Q  Median      3Q     Max
-1.1553 -0.6093 -0.3529  0.8142  2.4040

Random effects:
 Groups                      Name        Variance Std.Dev.
 Genotype_Zone_1:Plant_Zone_1 (Intercept)  0.33280 0.5769
 Genotype_Zone_2:Plant_Zone_2 (Intercept)  0.01618 0.1272
 Residual                                 80.45962 8.9699
Number of obs: 39, groups:  Genotype_Zone_1:Plant_Zone_1, 10; Genotype_Zone_2:Plant_Zone_2, 10

Fixed effects:
            Estimate Std. Error      df t value Pr(>|t|)
(Intercept)  -1.5054     0.3288  5.7449  -4.578  0.00422 **
---
Signif. codes:  0 '***' 0.001 '**' 0.01 '*' 0.05 '.' 0.1 ' ' 1

Dispersion: 8.97
```

Model residuals

Statistic	Value
Sample skewness	0.9646
Sample excess kurtosis	-0.1876
Passed Shapiro Wilk normality test	No (p-value = 0.000314 < 0.05)

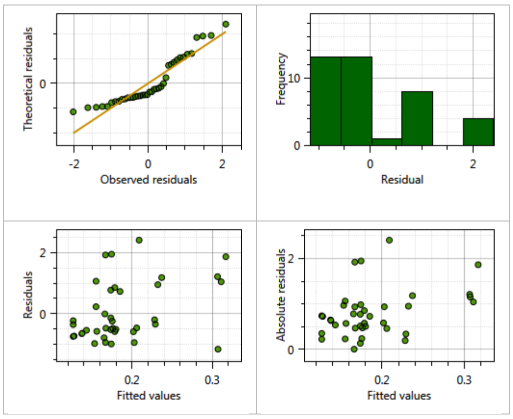

Halt frequency per zone per short/medium/long

Selected zones	Zone 1, Zone 2
Event duration categories	duration < 2, 2 <= duration < 10, duration >= 10
Data transformation	Natural logarithm
Analysis	Zone difference analysis

Model result halt frequency per zone per short/medium/long

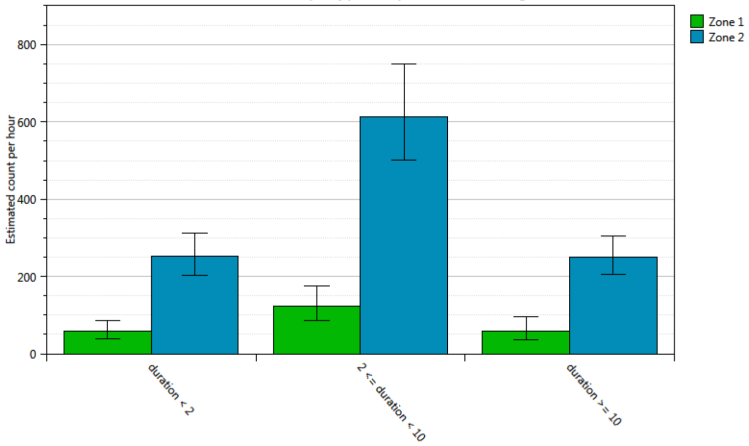

Results difference tests Zone 1 - Zone 2: p values and 95% confidence intervals of the difference on the transformed scale for each statistic.

Behaviour statistic	Cur3-RMX180	Remark
Halt frequency duration < 2 (diff. Zone 1 - Zone 2)	p=8.17E-06****<br>[-1.88, -1.06]	CR
Halt frequency 2 <= duration < 10 (diff. Zone 1 - Zone 2)	p=2.39E-05****<br>[-1.91, -1.06]	
Halt frequency duration >= 10 (diff. Zone 1 - Zone 2)	p=0.00413**<br>[-1.79, -0.544]	

CR = Check residuals

The model predictions and 95% confidence intervals for each statistic.

Statistic	Cur3-RMX180	Remark
Halt frequency (duration < 2 - Zone 1)	57.4<br>[38.7, 85.4]	
Halt frequency (2 <= duration < 10 - Zone 1)	123<br>[85.5, 176]	CR
Halt frequency (duration >= 10 - Zone 1)	58.2<br>[35.1, 96.5]	CR
Halt frequency (duration < 2 - Zone 2)	252<br>[202, 313]	
Halt frequency (2 <= duration < 10 - Zone 2)	614<br>[501, 751]	
Halt frequency (duration >= 10 - Zone 2)	250<br>[205, 305]	CR

CR = Check residuals

Data summary

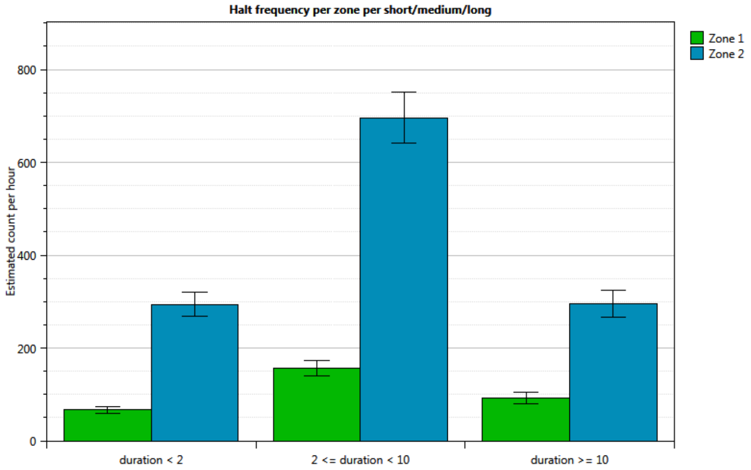

Genotype Zone 1	Genotype Zone 2	Genotype Zone 3	Mean duration < 2 - Zone 1	StdErr duration < 2 - Zone 1	Mean 2 <= duration < 10 - Zone 1	StdErr 2 <= duration < 10 - Zone 1	Mean duration >= 10 - Zone 1	StdErr duration >= 10 - Zone 1	Mean duration < 2 - Zone 2	StdErr duration < 2 - Zone 2	Mean 2 <= duration < 10 - Zone 2	StdErr 2 <= duration < 10 - Zone 2	Mean duration >= 10 - Zone 2	StdErr duration >= 10 - Zone 2
Cur3	RMX180	Neutral	66.64	7.22	156.78	16.48	92.85	11.99	294.09	25.55	696.3	55.25	295.72	29.32

Analysis halt frequency (duration < 2 - Zone 1)

**Analysis model**	Linear mixed model fit by REML: Halt_frequency_duration_2_Zone_1 ~ 1 + (1|Genotype_Zone_1:Plant_Zone_1) + (1|Genotype_Zone_2:Plant_Zone_2)
**Transformation**	Natural logarithm

Confidence intervals halt frequency (duration < 2 - Zone 1)

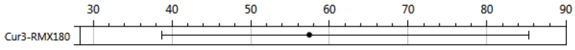

Confidence intervals halt frequency (duration < 2 - Zone 1)

Genotype Zone 1	Genotype Zone 2	Mean	Lower 95% CL	Upper 95% CL	Group
Cur3	RMX180	57.45	38.66	85.36	a

Model summary

```
Linear mixed model fit by REML. t-tests use Satterthwaite's method ['lmerModLmerTest']
Formula: Halt_frequency_duration_2_Zone_1 ~ 1 + (1 | Genotype_Zone_1:Plant_Zone_1) +      (1 |
Genotype_Zone_2:Plant_Zone_2)
   Data: data

REML criterion at convergence: 77.6

Scaled residuals: 
     Min       1Q   Median       3Q      Max 
-2.31843 -0.42933  0.08153  0.44836  1.50659 

Random effects:
 Groups                       Name        Variance Std.Dev.
 Genotype_Zone_1:Plant_Zone_1 (Intercept) 0.1196   0.3458  
 Genotype_Zone_2:Plant_Zone_2 (Intercept) 0.1029   0.3207  
 Residual                                 0.3093   0.5562  
Number of obs: 37, groups:  Genotype_Zone_1:Plant_Zone_1, 10; Genotype_Zone_2:Plant_Zone_2, 10

Fixed effects:
            Estimate Std. Error     df t value Pr(>|t|)    
(Intercept)   4.0508     0.1758 9.2694   23.04 1.69e-09 ***
---
Signif. codes:  0 '***' 0.001 '**' 0.01 '*' 0.05 '.' 0.1 ' ' 1
```

Model residuals

Statistic	Value
Sample skewness	-0.4089
Sample excess kurtosis	0.3046
Passed Shapiro Wilk normality test	Yes (p-value = 0.5546 > 0.05)

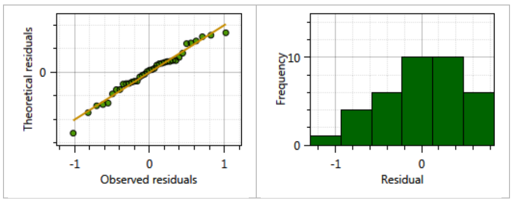

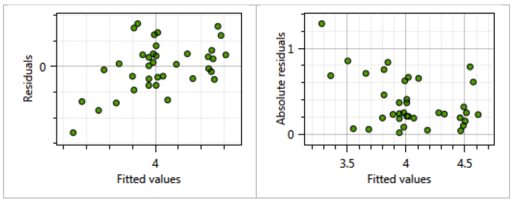

Analysis halt frequency (2 <= duration < 10 - Zone 1)

**Analysis model**	Linear mixed model fit by REML: Halt_frequency_2_duration_10_Zone_1 ~ 1 + (1|Genotype_Zone_1:Plant_Zone_1) + (1|Genotype_Zone_2:Plant_Zone_2)
**Transformation**	Natural logarithm

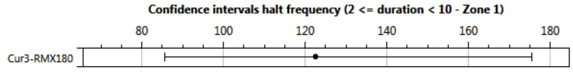

Confidence intervals halt frequency (2 <= duration < 10 - Zone 1)

Genotype Zone 1	Genotype Zone 2	Mean	Lower 95% CL	Upper 95% CL	Group
Cur3	RMX180	122.6	85.55	175.6	a

Model summary

```
Linear mixed model fit by REML. t-tests use Satterthwaite's method ['lmerModLmerTest']
Formula: Halt_frequency_2_duration_10_Zone_1 ~ 1 + (1 | Genotype_Zone_1:Plant_Zone_1) +      (1 |
Genotype_Zone_2:Plant_Zone_2)
   Data: data

REML criterion at convergence: 105.4

Scaled residuals:
    Min      1Q  Median      3Q     Max
-4.4368 -0.2463  0.1444  0.4963  1.3135

Random effects:
 Groups                       Name        Variance  Std.Dev.
 Genotype_Zone_1:Plant_Zone_1 (Intercept) 1.198e-16 1.095e-08
 Genotype_Zone_2:Plant_Zone_2 (Intercept) 1.943e-02 1.394e-01
 Residual                                 8.985e-01 9.479e-01
Number of obs: 38, groups:  Genotype_Zone_1:Plant_Zone_1, 10; Genotype_Zone_2:Plant_Zone_2, 10

Fixed effects:
            Estimate Std. Error    df t value Pr(>|t|)
(Intercept)    4.809      0.160 9.431   30.05  1.1e-10 ***
---
Signif. codes:  0 '***' 0.001 '**' 0.01 '*' 0.05 '.' 0.1 ' ' 1
```

Model residuals

Statistic	Value
Sample skewness	-2.567
Sample excess kurtosis	10.22
Passed Shapiro Wilk normality test	No (p-value = 5.752E-06 < 0.05)

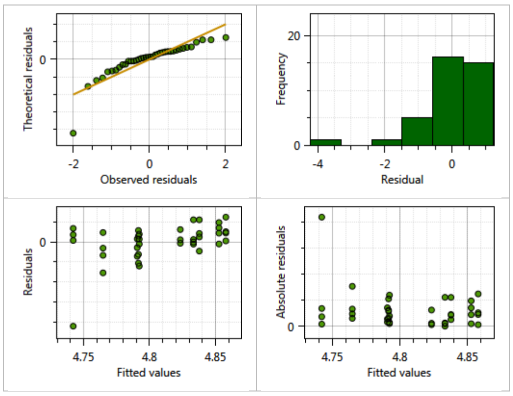

Analysis halt frequency (duration >= 10 - Zone 1)

**Analysis model**	Linear mixed model fit by REML: Halt_frequency_duration_10_Zone_1 ~ 1 + (1|Genotype_Zone_1:Plant_Zone_1) + (1|Genotype_Zone_2:Plant_Zone_2)
**Transformation**	Natural logarithm

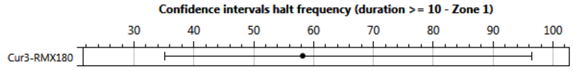

Confidence intervals halt frequency (duration >= 10 - Zone 1)

Genotype Zone 1	Genotype Zone 2	Mean	Lower 95% CL	Upper 95% CL	Group
Cur3	RMX180	58.18	35.08	96.51	a

Model summary

```
Linear mixed model fit by REML. t-tests use Satterthwaite's method ['lmerModLmerTest']
Formula: Halt_frequency_duration_10_Zone_1 ~ 1 + (1 | Genotype_Zone_1:Plant_Zone_1) +      (1 |
Genotype_Zone_2:Plant_Zone_2)
   Data: data

REML criterion at convergence: 126.3

Scaled residuals:
    Min      1Q  Median      3Q     Max
-3.1151 -0.3522  0.2182  0.5227  1.3186

Random effects:
 Groups                       Name        Variance  Std.Dev.
 Genotype_Zone_1:Plant_Zone_1 (Intercept) 4.889e-15 6.992e-08
 Genotype_Zone_2:Plant_Zone_2 (Intercept) 1.567e-01 3.958e-01
 Residual                                 1.352e+00 1.163e+00
Number of obs: 39, groups:  Genotype_Zone_1:Plant_Zone_1, 10; Genotype_Zone_2:Plant_Zone_2, 10

Fixed effects:
            Estimate Std. Error     df t value Pr(>|t|)
(Intercept)   4.0636     0.2245 9.2234    18.1 1.61e-08 ***
---
Signif. codes:  0 '***' 0.001 '**' 0.01 '*' 0.05 '.' 0.1 ' ' 1
```

Model residuals

Statistic	Value
Sample skewness	-1.519
Sample excess kurtosis	2.563
Passed Shapiro Wilk normality test	No (p-value = 0.000259 < 0.05)

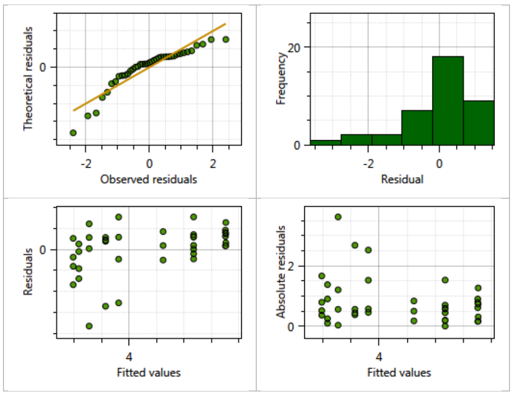

Analysis halt frequency (duration < 2 - Zone 2)

**Analysis model**	Linear mixed model fit by REML: Halt_frequency_duration_2_Zone_2 ~ 1 + (1|Genotype_Zone_1:Plant_Zone_1) + (1|Genotype_Zone_2:Plant_Zone_2)
**Transformation**	Natural logarithm

Confidence intervals halt frequency (duration < 2 - Zone 2)

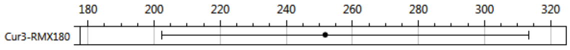

Confidence intervals halt frequency (duration < 2 - Zone 2)

Genotype Zone 1	Genotype Zone 2	Mean	Lower 95% CL	Upper 95% CL	Group
Cur3	RMX180	251.8	202.2	313.5	a

Model summary

```
Linear mixed model fit by REML. t-tests use Satterthwaite's method ['lmerModLmerTest']
Formula: Halt_frequency_duration_2_Zone_2 ~ 1 + (1 | Genotype_Zone_1:Plant_Zone_1) +      (1 |
Genotype_Zone_2:Plant_Zone_2)
   Data: data

REML criterion at convergence: 71.3

Scaled residuals: 
     Min       1Q   Median       3Q      Max 
-2.81733 -0.64063 -0.00936  0.76242  1.64038 

Random effects:
 Groups                       Name        Variance Std.Dev.
 Genotype_Zone_1:Plant_Zone_1 (Intercept) 0.000000 0.00000 
 Genotype_Zone_2:Plant_Zone_2 (Intercept) 0.002417 0.04917 
 Residual                                 0.344596 0.58702 
Number of obs: 39, groups:  Genotype_Zone_1:Plant_Zone_1, 10; Genotype_Zone_2:Plant_Zone_2, 10

Fixed effects:
            Estimate Std. Error      df t value Pr(>|t|)    
(Intercept)  5.52856    0.09528 8.11758   58.02 6.38e-12 ***
---
Signif. codes:  0 '***' 0.001 '**' 0.01 '*' 0.05 '.' 0.1 ' ' 1
```

Model residuals

Statistic	Value
Sample skewness	-0.4416
Sample excess kurtosis	0.1336
Passed Shapiro Wilk normality test	Yes (p-value = 0.3447 > 0.05)

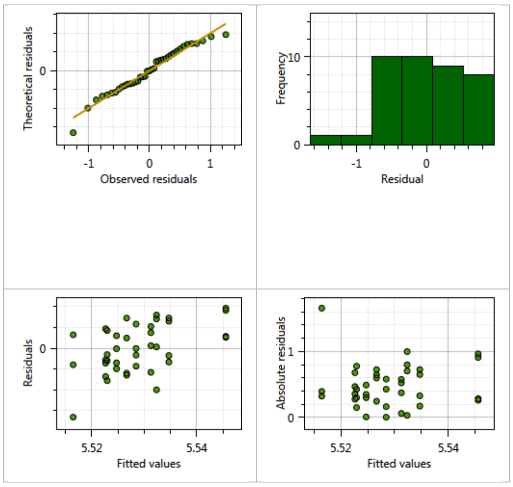

Analysis halt frequency (2 <= duration < 10 - Zone 2)

**Analysis model**	Linear mixed model fit by REML: Halt_frequency_2_duration_10_Zone_2 ~ 1 + (1|Genotype_Zone_1:Plant_Zone_1) + (1|Genotype_Zone_2:Plant_Zone_2)
**Transformation**	Natural logarithm

Confidence intervals halt frequency (2 <= duration < 10 - Zone 2)

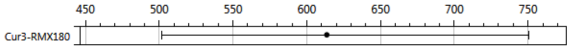

Confidence intervals halt frequency (2 <= duration < 10 - Zone 2)

Genotype Zone 1	Genotype Zone 2	Mean	Lower 95% CL	Upper 95% CL	Group
Cur3	RMX180	613.6	501.5	750.7	a

Model summary

```
Linear mixed model fit by REML. t-tests use Satterthwaite's method ['lmerModLmerTest']
Formula: Halt_frequency_2_duration_10_Zone_2 ~ 1 + (1 | Genotype_Zone_1:Plant_Zone_1) +      (1 |
Genotype_Zone_2:Plant_Zone_2)
   Data: data
```

```
REML criterion at convergence: 63

Scaled residuals:
     Min       1Q   Median       3Q      Max
-2.66734 -0.55529  0.06335  0.74756  1.85903

Random effects:
 Groups                        Name        Variance  Std.Dev.
 Genotype_Zone_1:Plant_Zone_1 (Intercept) 1.661e-18 1.289e-09
 Genotype_Zone_2:Plant_Zone_2 (Intercept) 7.040e-03 8.391e-02
 Residual                                 2.725e-01 5.221e-01
Number of obs: 39, groups:  Genotype_Zone_1:Plant_Zone_1, 10; Genotype_Zone_2:Plant_Zone_2, 10

Fixed effects:
            Estimate Std. Error      df t value Pr(>|t|)
(Intercept)  6.41929    0.08773 8.13895   73.17 9.18e-13 ***
---
Signif. codes:  0 '***' 0.001 '**' 0.01 '*' 0.05 '.' 0.1 ' ' 1
```

Model residuals

Statistic	Value
Sample skewness	-0.5252
Sample excess kurtosis	0.4633
Passed Shapiro Wilk normality test	Yes (p-value = 0.5002 > 0.05)

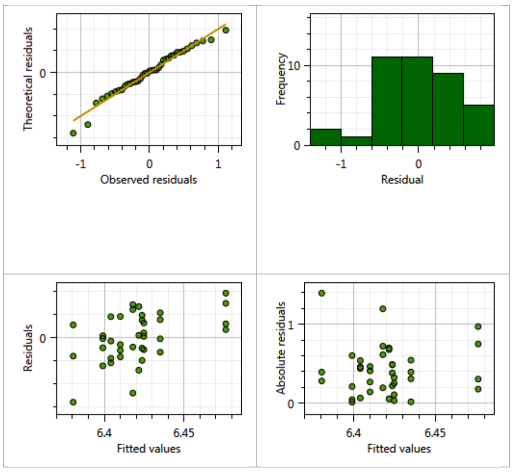

Analysis halt frequency (duration >= 10 - Zone 2)

**Analysis model**	Linear mixed model fit by REML: Halt_frequency_duration_10_Zone_2 ~ 1 + (1|Genotype_Zone_1:Plant_Zone_1) + (1|Genotype_Zone_2:Plant_Zone_2)
**Transformation**	Natural logarithm

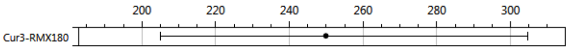

Confidence intervals halt frequency (duration >= 10 - Zone 2)

Genotype Zone 1	Genotype Zone 2	Mean	Lower 95% CL	Upper 95% CL	Group
Cur3	RMX180	249.9	204.9	304.8	a

Model summary

```
Linear mixed model fit by REML. t-tests use Satterthwaite's method ['lmerModLmerTest']
Formula: Halt_frequency_duration_10_Zone_2 ~ 1 + (1 | Genotype_Zone_1:Plant_Zone_1) +      (1 |
Genotype_Zone_2:Plant_Zone_2)
   Data: data

REML criterion at convergence: 74.2

Scaled residuals:
    Min      1Q  Median      3Q     Max
-3.3178 -0.4918  0.0974  0.4433  2.1087

Random effects:
 Groups                        Name        Variance  Std.Dev.
 Genotype_Zone_1:Plant_Zone_1 (Intercept) 0.000e+00 0.000e+00
 Genotype_Zone_2:Plant_Zone_2 (Intercept) 9.727e-17 9.863e-09
 Residual                                 3.746e-01 6.121e-01
Number of obs: 39, groups:  Genotype_Zone_1:Plant_Zone_1, 10; Genotype_Zone_2:Plant_Zone_2, 10

Fixed effects:
            Estimate Std. Error       df t value Pr(>|t|)
(Intercept)  5.52111    0.09801 38.00000   56.33   <2e-16 ***
---
Signif. codes:  0 '***' 0.001 '**' 0.01 '*' 0.05 '.' 0.1 ' ' 1
```

Model residuals

Statistic	Value
Sample skewness	-0.646
Sample excess kurtosis	2.372
Passed Shapiro Wilk normality test	No (p-value = 0.03196 < 0.05)

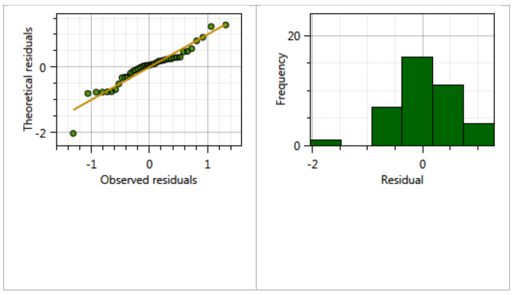

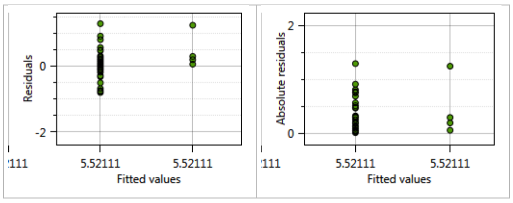

Analysis halt frequency duration < 2 (diff. Zone 1 - Zone 2)

**Analysis model**	Generalized linear mixed model with dispersion factor, formula=cbind(Halt_frequency_duration_2_Zone_1,Halt_frequency_duration_2_Zone_2) ~ 1 + (1|Genotype_Zone_1:Plant_Zone_1) + (1|Genotype_Zone_2:Plant_Zone_2)
**Transformation**	Logit

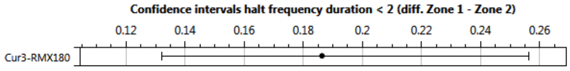

Confidence intervals halt frequency duration < 2 (diff. Zone 1 - Zone 2)

Genotype Zone 1	Genotype Zone 2	Mean	Lower 95% CL	Upper 95% CL	Group
Cur3	RMX180	0.1863	0.132	0.2564	a

Model summary

```
Linear mixed model fit by REML. t-tests use Satterthwaite's method ['lmerModLmerTest']
Formula: ziFormula
   Data: data
Weights: wi

REML criterion at convergence: 106.1

Scaled residuals:
     Min       1Q   Median       3Q      Max
-1.44923 -0.83993 -0.04734  0.80258  1.81012

Random effects:
 Groups                      Name        Variance Std.Dev.
 Genotype_Zone_1:Plant_Zone_1 (Intercept)  0.06534 0.2556
 Genotype_Zone_2:Plant_Zone_2 (Intercept)  0.10567 0.3251
 Residual                                34.83674 5.9023
Number of obs: 39, groups:  Genotype_Zone_1:Plant_Zone_1, 10; Genotype_Zone_2:Plant_Zone_2, 10

Fixed effects:
            Estimate Std. Error      df t value Pr(>|t|)
(Intercept)  -1.4742     0.1854 10.7130   -7.95 8.17e-06 ***
---
Signif. codes:  0 '***' 0.001 '**' 0.01 '*' 0.05 '.' 0.1 ' ' 1

Dispersion: 5.902
```

Model residuals

Statistic	Value
Sample skewness	0.355
Sample excess kurtosis	-1.014
Passed Shapiro Wilk normality test	No (p-value = 0.03409 < 0.05)

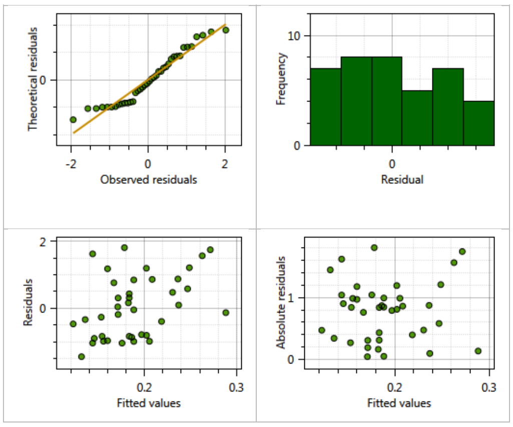

Analysis halt frequency 2 <= duration < 10 (diff. Zone 1 - Zone 2)

**Analysis model**	Generalized linear mixed model with dispersion factor, formula=cbind(Halt_frequency_2_duration_10_Zone_1,Halt_frequency_2_duration_10_Zone_2) ~ 1 + (1|Genotype_Zone_1:Plant_Zone_1) + (1|Genotype_Zone_2:Plant_Zone_2)
**Transformation**	Logit

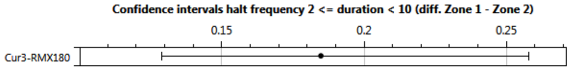

Confidence intervals halt frequency 2 <= duration < 10 (diff. Zone 1 - Zone 2)

Genotype Zone 1	Genotype Zone 2	Mean	Lower 95% CL	Upper 95% CL	Group
Cur3	RMX180	0.1848	0.1288	0.258	a

Model summary

```
Linear mixed model fit by REML. t-tests use Satterthwaite's method ['lmerModLmerTest']
Formula: ziFormula
   Data: data
Weights: wi

REML criterion at convergence: 109.1

Scaled residuals:
     Min       1Q   Median       3Q      Max
-1.49883 -0.67540  0.00213  0.83586  1.64206

Random effects:
 Groups                      Name        Variance Std.Dev.
 Genotype_Zone_1:Plant_Zone_1 (Intercept)  0.07457 0.2731
 Genotype_Zone_2:Plant_Zone_2 (Intercept)  0.08763 0.2960
 Residual                                94.94699 9.7441
Number of obs: 39, groups:  Genotype_Zone_1:Plant_Zone_1, 10; Genotype_Zone_2:Plant_Zone_2, 10

Fixed effects:
            Estimate Std. Error      df t value Pr(>|t|)
(Intercept)  -1.4841     0.1894  9.1429  -7.835 2.39e-05 ***
---
```

```
Signif. codes:  0 '***' 0.001 '**' 0.01 '*' 0.05 '.' 0.1 ' ' 1

Dispersion: 9.744
```

Model residuals

Statistic	Value
Sample skewness	0.2347
Sample excess kurtosis	-1.012
Passed Shapiro Wilk normality test	Yes (p-value = 0.07345 > 0.05)

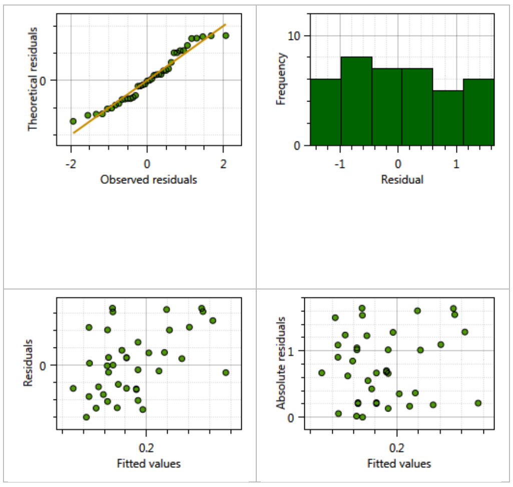

Analysis halt frequency duration >= 10 (diff. Zone 1 - Zone 2)

**Analysis model**	Generalized linear mixed model with dispersion factor, formula=cbind(Halt_frequency_duration_10_Zone_1,Halt_frequency_duration_10_Zone_2) ~ 1 + (1|Genotype_Zone_1:Plant_Zone_1) + (1|Genotype_Zone_2:Plant_Zone_2)
**Transformation**	Logit

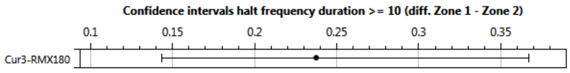

Confidence intervals halt frequency duration >= 10 (diff. Zone 1 - Zone 2)

Genotype Zone 1	Genotype Zone 2	Mean	Lower 95% CL	Upper 95% CL	Group
Cur3	RMX180	0.2374	0.1431	0.3672	a

Model summary

```
Linear mixed model fit by REML. t-tests use Satterthwaite's method ['lmerModLmerTest']
Formula: ziFormula
   Data: data
Weights: wi

REML criterion at convergence: 123.9

Scaled residuals:
     Min       1Q   Median       3Q      Max
-1.61160 -0.52792  0.00661  0.53848  2.50898

Random effects:
 Groups                      Name        Variance Std.Dev.
 Genotype_Zone_1:Plant_Zone_1 (Intercept)  0.1349  0.3673
 Genotype_Zone_2:Plant_Zone_2 (Intercept)  0.2169  0.4657
 Residual                              71.6324  8.4636
Number of obs: 39, groups:  Genotype_Zone_1:Plant_Zone_1, 10; Genotype_Zone_2:Plant_Zone_2, 10

Fixed effects:
            Estimate Std. Error     df t value Pr(>|t|)
(Intercept)   -1.167      0.250  5.592  -4.668  0.00413 **
---
Signif. codes:  0 '***' 0.001 '**' 0.01 '*' 0.05 '.' 0.1 ' ' 1

Dispersion: 8.464
```

Model residuals

Statistic	Value
Sample skewness	0.5131
Sample excess kurtosis	0.3951
Passed Shapiro Wilk normality test	Yes (p-value = 0.6645 > 0.05)

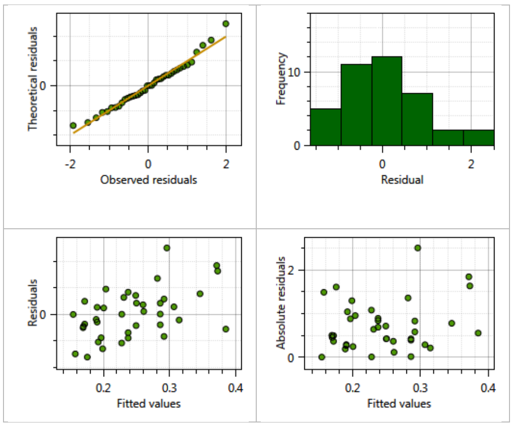

Movement frequency per zone

Selected zones	Zone 1, Zone 2
Data transformation	Natural logarithm
Analysis	Zone difference analysis

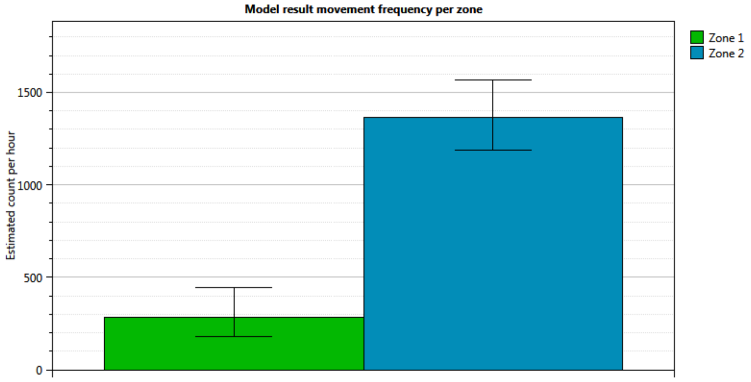

Results difference tests Zone 1 - Zone 2: p values and 95% confidence intervals of the difference on the transformed scale for each statistic.

Behaviour statistic	Cur3-RMX180
Movement frequency (diff. Zone 1 - Zone 2)	p=6E-05****<br>[-1.62, -0.854]

The model predictions and 95% confidence intervals for each statistic.

Statistic	Cur3-RMX180	Remark
Movement frequency (Zone 1)	285<br>[182, 446]	CR
Movement frequency (Zone 2)	1.36E+03<br>[1.19E+03, 1.57E+03]	

CR = Check residuals

Data summary

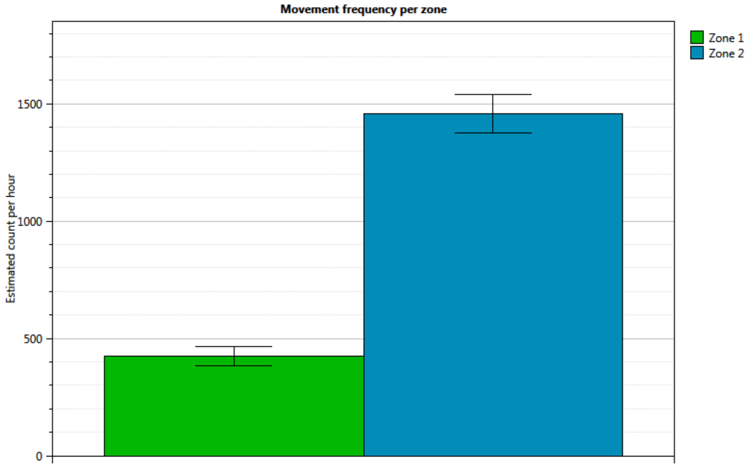

Genotype Zone 1	Genotype Zone 2	Genotype Zone 3	Mean Zone 1	StdErr Zone 1	Mean Zone 2	StdErr Zone 2
Cur3	RMX180	Neutral	424.97	42.26	1457.98	83.57

Analysis movement frequency (Zone 1)

Analysis model	Linear mixed model fit by REML: Movement_frequency_Zone_1 ~ 1 + (1|Genotype_Zone_1:Plant_Zone_1) + (1|Genotype_Zone_2:Plant_Zone_2)
Transformation	Natural logarithm

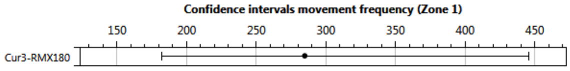

Confidence intervals movement frequency (Zone 1)

Genotype Zone 1	Genotype Zone 2	Mean	Lower 95% CL	Upper 95% CL	Group
Cur3	RMX180	284.7	181.8	445.9	a

Model summary

```
Linear mixed model fit by REML. t-tests use Satterthwaite's method ['lmerModLmerTest']
Formula: Movement_frequency_Zone_1 ~ 1 + (1 | Genotype_Zone_1:Plant_Zone_1) +      (1 |
Genotype_Zone_2:Plant_Zone_2)
   Data: data

REML criterion at convergence: 136.2

Scaled residuals:
    Min      1Q  Median      3Q     Max
-3.9177 -0.0530  0.2426  0.4475  1.0106

Random effects:
 Groups                       Name        Variance Std.Dev.
 Genotype_Zone_1:Plant_Zone_1 (Intercept) 0.000    0.000
 Genotype_Zone_2:Plant_Zone_2 (Intercept) 0.000    0.000
 Residual                                 1.914    1.384
Number of obs: 39, groups:  Genotype_Zone_1:Plant_Zone_1, 10; Genotype_Zone_2:Plant_Zone_2, 10

Fixed effects:
            Estimate Std. Error      df t value Pr(>|t|)
(Intercept)   5.6516     0.2216 38.0000   25.51   <2e-16 ***
---
Signif. codes:  0 '***' 0.001 '**' 0.01 '*' 0.05 '.' 0.1 ' ' 1
```

Model residuals

Statistic	Value
Sample skewness	-2.996
Sample excess kurtosis	9.921
Passed Shapiro Wilk normality test	No (p-value = 1.753E-08 < 0.05)

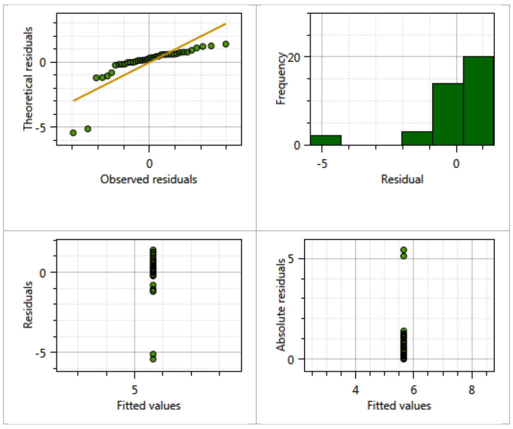

Analysis movement frequency (Zone 2)

**Analysis model**	Linear mixed model fit by REML: Movement_frequency_Zone_2 ~ 1 + (1|Genotype_Zone_1:Plant_Zone_1) + (1|Genotype_Zone_2:Plant_Zone_2)
**Transformation**	Natural logarithm

Confidence intervals movement frequency (Zone 2)

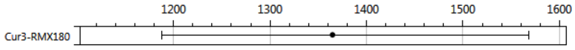

Confidence intervals movement frequency (Zone 2)

Genotype Zone 1	Genotype Zone 2	Mean	Lower 95% CL	Upper 95% CL	Group
Cur3	RMX180	1365	1187	1569	a

Model summary

```
Linear mixed model fit by REML. t-tests use Satterthwaite's method ['lmerModLmerTest']
Formula: Movement_frequency_Zone_2 ~ 1 + (1 | Genotype_Zone_1:Plant_Zone_1) +      (1 |
Genotype_Zone_2:Plant_Zone_2)
   Data: data

REML criterion at convergence: 37.7

Scaled residuals:
    Min      1Q  Median      3Q     Max
-2.9445 -0.5495  0.2641  0.7086  1.8936

Random effects:
 Groups                       Name        Variance  Std.Dev.
 Genotype_Zone_1:Plant_Zone_1 (Intercept) 1.013e-17 3.183e-09
 Genotype_Zone_2:Plant_Zone_2 (Intercept) 9.706e-04 3.115e-02
 Residual                                 1.425e-01 3.775e-01
Number of obs: 39, groups:  Genotype_Zone_1:Plant_Zone_1, 10; Genotype_Zone_2:Plant_Zone_2, 10

Fixed effects:
            Estimate Std. Error      df t value Pr(>|t|)
(Intercept)  7.21871    0.06125 8.73890   117.9 2.69e-15 ***
---
Signif. codes:  0 '***' 0.001 '**' 0.01 '*' 0.05 '.' 0.1 ' ' 1
```

Model residuals

Statistic	Value
Sample skewness	-0.5062
Sample excess kurtosis	0.576
Passed Shapiro Wilk normality test	Yes (p-value = 0.3482 > 0.05)

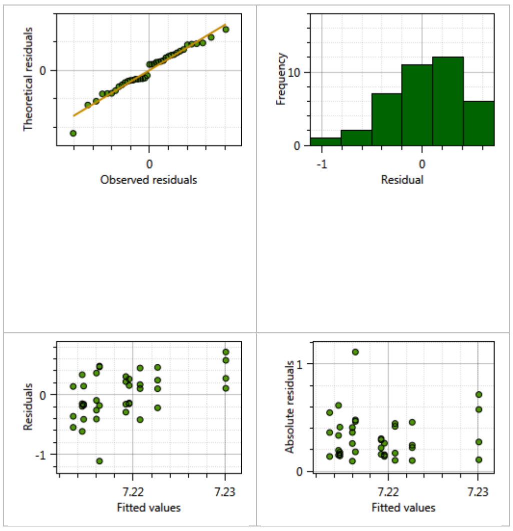

Analysis movement frequency (diff. Zone 1 - Zone 2)

**Analysis model**	Generalized linear mixed model with dispersion factor, formula=cbind(Movement_frequency_Zone_1,Movement_frequency_Zone_2) ~ 1 + (1|Genotype_Zone_1:Plant_Zone_1) + (1|Genotype_Zone_2:Plant_Zone_2)
**Transformation**	Logit

Confidence intervals movement frequency (diff. Zone 1 - Zone 2)

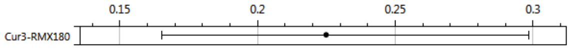

Confidence intervals movement frequency (diff. Zone 1 - Zone 2)

Genotype Zone 1	Genotype Zone 2	Mean	Lower 95% CL	Upper 95% CL	Group
Cur3	RMX180	0.2249	0.1651	0.2985	a

Model summary

```
Linear mixed model fit by REML. t-tests use Satterthwaite's method ['lmerModLmerTest']
Formula: ziFormula
   Data: data
Weights: wi
```

```
REML criterion at convergence: 99.4

Scaled residuals:
    Min      1Q  Median      3Q     Max
-1.4889 -0.7263 -0.1373  0.6020  1.7427

Random effects:
 Groups                        Name        Variance  Std.Dev.
 Genotype_Zone_1:Plant_Zone_1 (Intercept)   0.03525  0.1878
 Genotype_Zone_2:Plant_Zone_2 (Intercept)   0.08651  0.2941
 Residual                                 199.17749 14.1130
Number of obs: 39, groups:  Genotype_Zone_1:Plant_Zone_1, 10; Genotype_Zone_2:Plant_Zone_2, 10

Fixed effects:
            Estimate Std. Error      df t value Pr(>|t|)
(Intercept)  -1.2375     0.1676  8.3984  -7.386    6e-05 ***
---
Signif. codes:  0 '***' 0.001 '**' 0.01 '*' 0.05 '.' 0.1 ' ' 1

Dispersion: 14.11
```

Model residuals

Statistic	Value
Sample skewness	0.2979
Sample excess kurtosis	-0.843
Passed Shapiro Wilk normality test	Yes (p-value = 0.1244 > 0.05)

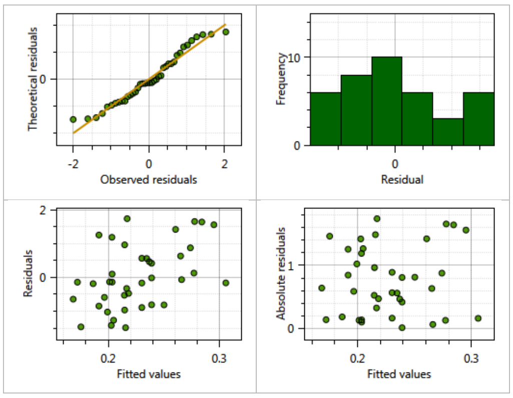

Movement frequency per zone per hour

Selected hours	0, 1, 2, 3, 4, 5, 6, 7
Selected zones	Zone 1, Zone 2
Data transformation	Natural logarithm
Analysis	Zone difference analysis

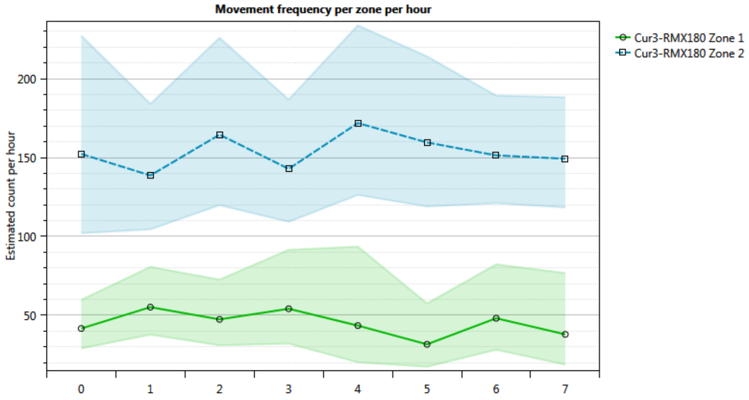

Results difference tests Zone 1 - Zone 2: p values and 95% confidence intervals of the difference on the transformed scale for each statistic.

Behaviour statistic	Cur3-RMX180	Remark
Movement frequency H0 (diff. Zone 1 - Zone 2)	p=0.00131**<br>[-1.79, -0.634]	
Movement frequency H1 (diff. Zone 1 - Zone 2)	p=7.34E-06****<br>[-1.29, -0.569]	
Movement frequency H2 (diff. Zone 1 - Zone 2)	p=0.00204**<br>[-1.73, -0.559]	CR
Movement frequency H3 (diff. Zone 1 - Zone 2)	p=0.00195**<br>[-1.51, -0.468]	
Movement frequency H4 (diff. Zone 1 - Zone 2)	p=0.00322**<br>[-2.01, -0.601]	CR
Movement frequency H5 (diff. Zone 1 - Zone 2)	p=0.000302***<br>[-2.05, -0.871]	CR
Movement frequency H6 (diff. Zone 1 - Zone 2)	p=2.16E-06****<br>[-1.89, -0.885]	CR
Movement frequency H7 (diff. Zone 1 - Zone 2)	p=0.00281**<br>[-2.12, -0.629]	CR

CR = Check residuals

The model predictions and 95% confidence intervals for each statistic.

Statistic	Cur3-RMX180	Remark
Movement frequency (H0 - Zone 1)	41.6<br>[29, 59.8]	CR
Movement frequency (H0 - Zone 2)	152<br>[102, 227]	CR
Movement frequency (H1 - Zone 1)	55.2<br>[37.7, 80.7]	CR
Movement frequency (H1 - Zone 2)	139<br>[104, 184]	CR
Movement frequency (H2 - Zone 1)	47.4<br>[30.9, 72.7]	
Movement frequency (H2 - Zone 2)	165<br>[120, 226]	CR
Movement frequency (H3 - Zone 1)	54.1<br>[32, 91.6]	CR
Movement frequency (H3 - Zone 2)	143<br>[109, 187]	CR
Movement frequency (H4 - Zone 1)	43.5	

Statistic	Cur3-RMX180	Remark
	[20.2, 93.6]	
Movement frequency (H4 - Zone 2)	172	CR
	[126, 234]	
Movement frequency (H5 - Zone 1)	31.6	CR
	[17.3, 57.5]	
Movement frequency (H5 - Zone 2)	160	
	[119, 214]	
Movement frequency (H6 - Zone 1)	48.1	
	[28.1, 82.3]	
Movement frequency (H6 - Zone 2)	151	CR
	[121, 189]	
Movement frequency (H7 - Zone 1)	37.9	CR
	[18.7, 76.8]	
Movement frequency (H7 - Zone 2)	149	
	[118, 188]	

CR = Check residuals

Data summary

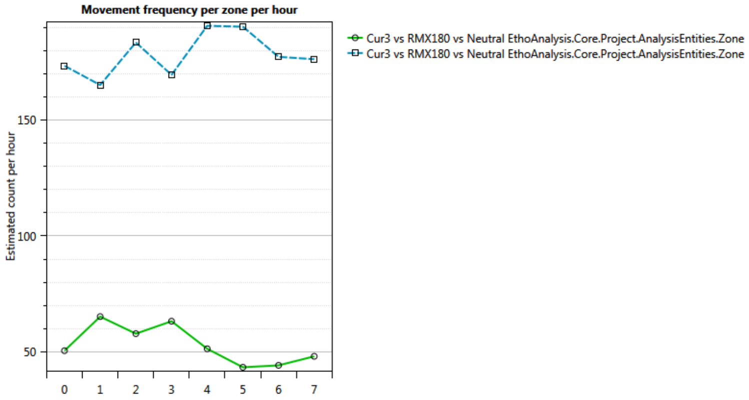

Genotype Zone 1	Genotype Zone 2	Genotype Zone 3	Mean H0 - Zone 1	StdErr H0 - Zone 1	Mean H0 - Zone 2	StdErr H0 - Zone 2	Mean H1 - Zone 1	StdErr H1 - Zone 1	Mean H1 - Zone 2	StdErr H1 - Zone 2	Mean H2 - Zone 1	StdErr H2 - Zone 1	Mean H2 - Zone 2	StdErr H2 - Zone 2
Cur3	RMX180	Neutral	50.54	7.65	173.42	15.49	65.23	6.55	165.11	15.17	57.88	8.64	183.47	14.81

Genotype Zone 1	Mean H3 - Zone 1	StdErr H3 - Zone 1	Mean H3 - Zone 2	StdErr H3 - Zone 2	Mean H4 - Zone 1	StdErr H4 - Zone 1	Mean H4 - Zone 2	StdErr H4 - Zone 2	Mean H5 - Zone 1	StdErr H5 - Zone 1	Mean H5 - Zone 2	StdErr H5 - Zone 2	Mean H6 - Zone 1	StdErr H6 - Zone 1	Mean H6 - Zone 2	StdE H6 - Zon 2
Cur3	63.24	8.37	169.49	13.64	51.36	8.27	190.66	16.91	43.38	7.17	190.29	15.95	44.21	8.27	177.26	16.7[illegible]

Analysis movement frequency (H0 - Zone 1)

**Analysis model**	Linear mixed model fit by REML: Movement_frequency_H0_Zone_1 ~ 1 + (1|Genotype_Zone_1:Plant_Zone_1) + (1|Genotype_Zone_2:Plant_Zone_2)
**Transformation**	Natural logarithm

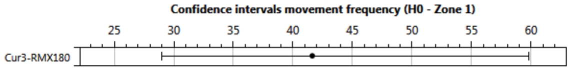

Confidence intervals movement frequency (H0 - Zone 1)

Genotype Zone 1	Genotype Zone 2	Mean	Lower 95% CL	Upper 95% CL	Group
Cur3	RMX180	41.62	28.96	59.83	a

Model summary

```
Linear mixed model fit by REML. t-tests use Satterthwaite's method ['lmerModLmerTest']
Formula: Movement_frequency_H0_Zone_1 ~ 1 + (1 | Genotype_Zone_1:Plant_Zone_1) +      (1 |
Genotype_Zone_2:Plant_Zone_2)
   Data: data

REML criterion at convergence: 95.8

Scaled residuals:
    Min      1Q  Median      3Q     Max
-2.9667 -0.2899  0.1068  0.6292  1.5740

Random effects:
 Groups                       Name        Variance Std.Dev.
 Genotype_Zone_1:Plant_Zone_1 (Intercept) 0.000    0.000
 Genotype_Zone_2:Plant_Zone_2 (Intercept) 0.000    0.000
 Residual                                 1.047    1.023
Number of obs: 33, groups:  Genotype_Zone_1:Plant_Zone_1, 10; Genotype_Zone_2:Plant_Zone_2, 10

Fixed effects:
            Estimate Std. Error      df t value Pr(>|t|)
(Intercept)   3.7287     0.1781 32.0000   20.93   <2e-16 ***
---
Signif. codes:  0 '***' 0.001 '**' 0.01 '*' 0.05 '.' 0.1 ' ' 1
```

Model residuals

Statistic	Value
Sample skewness	-1.375
Sample excess kurtosis	2.758
Passed Shapiro Wilk normality test	No (p-value = 0.003536 < 0.05)

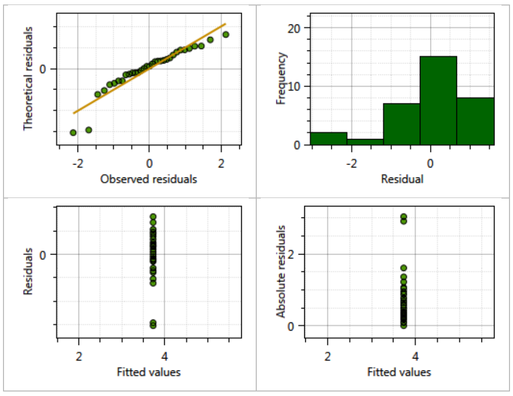

Analysis movement frequency (H0 - Zone 2)

**Analysis model**	Linear mixed model fit by REML: Movement_frequency_H0_Zone_2 ~ 1 + (1|Genotype_Zone_1:Plant_Zone_1) + (1|Genotype_Zone_2:Plant_Zone_2)
**Transformation**	Natural logarithm

Confidence intervals movement frequency (H0 - Zone 2)

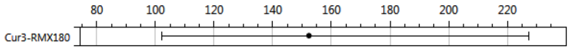

Confidence intervals movement frequency (H0 - Zone 2)

Genotype Zone 1	Genotype Zone 2	Mean	Lower 95% CL	Upper 95% CL	Group
Cur3	RMX180	152.3	102	227.4	a

Model summary

```
Linear mixed model fit by REML. t-tests use Satterthwaite's method ['lmerModLmerTest']
Formula: Movement_frequency_H0_Zone_2 ~ 1 + (1 | Genotype_Zone_1:Plant_Zone_1) +      (1 |
Genotype_Zone_2:Plant_Zone_2)
   Data: data

REML criterion at convergence: 87

Scaled residuals:
    Min      1Q  Median      3Q     Max
-3.6254 -0.1890  0.1479  0.4897  1.1653

Random effects:
 Groups                       Name        Variance  Std.Dev.
 Genotype_Zone_1:Plant_Zone_1 (Intercept) 4.519e-14 2.126e-07
 Genotype_Zone_2:Plant_Zone_2 (Intercept) 1.447e-01 3.804e-01
 Residual                                 5.333e-01 7.303e-01
Number of obs: 36, groups:  Genotype_Zone_1:Plant_Zone_1, 10; Genotype_Zone_2:Plant_Zone_2, 10

Fixed effects:
            Estimate Std. Error    df t value Pr(>|t|)
(Intercept)    5.026      0.172 7.551   29.23 4.81e-09 ***
---
Signif. codes:  0 '***' 0.001 '**' 0.01 '*' 0.05 '.' 0.1 ' ' 1
```

Model residuals

Statistic	Value
Sample skewness	-2.373
Sample excess kurtosis	7.074
Passed Shapiro Wilk normality test	No (p-value = 3.525E-06 < 0.05)

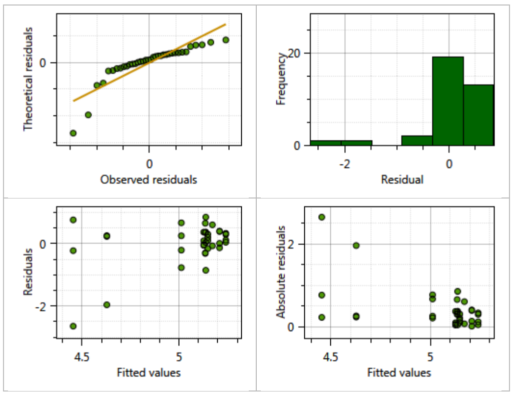

Analysis movement frequency (H1 - Zone 1)

**Analysis model**	Linear mixed model fit by REML: Movement_frequency_H1_Zone_1 ~ 1 + (1|Genotype_Zone_1:Plant_Zone_1) + (1|Genotype_Zone_2:Plant_Zone_2)
**Transformation**	Natural logarithm

Confidence intervals movement frequency (H1 - Zone 1)

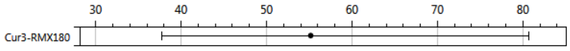

Confidence intervals movement frequency (H1 - Zone 1)

Genotype Zone 1	Genotype Zone 2	Mean	Lower 95% CL	Upper 95% CL	Group
Cur3	RMX180	55.16	37.7	80.73	a

Model summary

```
Linear mixed model fit by REML. t-tests use Satterthwaite's method ['lmerModLmerTest']
Formula: Movement_frequency_H1_Zone_1 ~ 1 + (1 | Genotype_Zone_1:Plant_Zone_1) +      (1 |
Genotype_Zone_2:Plant_Zone_2)
   Data: data

REML criterion at convergence: 96.6

Scaled residuals:
    Min      1Q  Median      3Q     Max
-3.9662 -0.1505  0.2289  0.6409  0.9874

Random effects:
 Groups                       Name        Variance Std.Dev.
 Genotype_Zone_1:Plant_Zone_1 (Intercept) 0.00000  0.0000
 Genotype_Zone_2:Plant_Zone_2 (Intercept) 0.05552  0.2356
 Residual                                 0.78785  0.8876
Number of obs: 36, groups:  Genotype_Zone_1:Plant_Zone_1, 10; Genotype_Zone_2:Plant_Zone_2, 10

Fixed effects:
            Estimate Std. Error     df t value Pr(>|t|)
(Intercept)   4.0103     0.1659 8.2262   24.17 6.21e-09 ***
---
Signif. codes:  0 '***' 0.001 '**' 0.01 '*' 0.05 '.' 0.1 ' ' 1
```

Model residuals

Statistic	Value
Sample skewness	-2.335
Sample excess kurtosis	7.13
Passed Shapiro Wilk normality test	No (p-value = 5.307E-06 < 0.05)

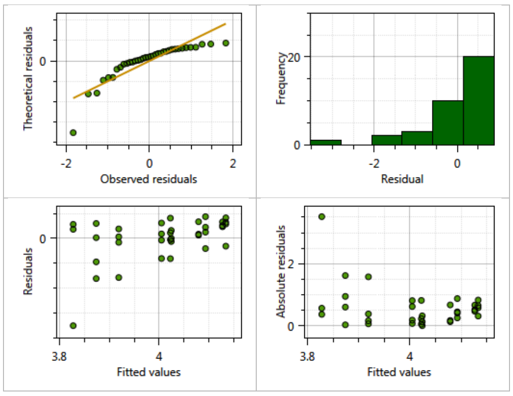

Analysis movement frequency (H1 - Zone 2)

**Analysis model**	Linear mixed model fit by REML: Movement_frequency_H1_Zone_2 ~ 1 + (1|Genotype_Zone_1:Plant_Zone_1) + (1|Genotype_Zone_2:Plant_Zone_2)
**Transformation**	Natural logarithm

Confidence intervals movement frequency (H1 - Zone 2)

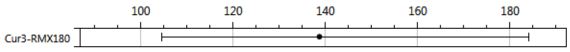

Confidence intervals movement frequency (H1 - Zone 2)

Genotype Zone 1	Genotype Zone 2	Mean	Lower 95% CL	Upper 95% CL	Group
Cur3	RMX180	138.7	104.5	184.1	a

Model summary

```
Linear mixed model fit by REML. t-tests use Satterthwaite's method ['lmerModLmerTest']
Formula: Movement_frequency_H1_Zone_2 ~ 1 + (1 | Genotype_Zone_1:Plant_Zone_1) +      (1 |
Genotype_Zone_2:Plant_Zone_2)
   Data: data

REML criterion at convergence: 94.1

Scaled residuals:
    Min      1Q  Median      3Q     Max
-3.4977 -0.2975  0.3630  0.6762  1.0349

Random effects:
 Groups                       Name        Variance  Std.Dev.
 Genotype_Zone_1:Plant_Zone_1 (Intercept) 1.724e-17 4.152e-09
 Genotype_Zone_2:Plant_Zone_2 (Intercept) 0.000e+00 0.000e+00
 Residual                                 7.221e-01 8.497e-01
Number of obs: 37, groups:  Genotype_Zone_1:Plant_Zone_1, 10; Genotype_Zone_2:Plant_Zone_2, 10

Fixed effects:
            Estimate Std. Error      df t value Pr(>|t|)
(Intercept)   4.9323     0.1397 36.0000   35.31   <2e-16 ***
---
Signif. codes:  0 '***' 0.001 '**' 0.01 '*' 0.05 '.' 0.1 ' ' 1
```

Model residuals

Statistic	Value
Sample skewness	-1.979
Sample excess kurtosis	4.566
Passed Shapiro Wilk normality test	No (p-value = 1.084E-05 < 0.05)

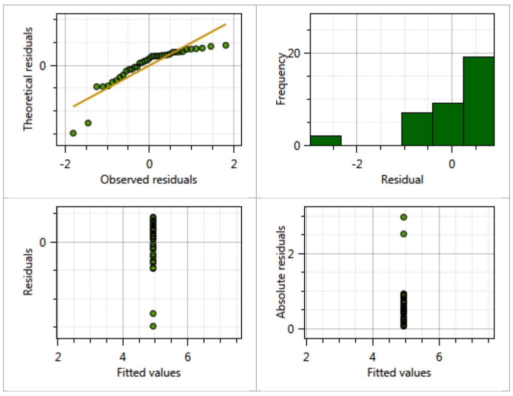

Analysis movement frequency (H2 - Zone 1)

**Analysis model**	Linear mixed model fit by REML: Movement_frequency_H2_Zone_1 ~ 1 + (1|Genotype_Zone_1:Plant_Zone_1) + (1|Genotype_Zone_2:Plant_Zone_2)
**Transformation**	Natural logarithm

Confidence intervals movement frequency (H2 - Zone 1)

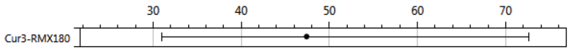

Confidence intervals movement frequency (H2 - Zone 1)

Genotype Zone 1	Genotype Zone 2	Mean	Lower 95% CL	Upper 95% CL	Group
Cur3	RMX180	47.41	30.94	72.66	a

Model summary

```
Linear mixed model fit by REML. t-tests use Satterthwaite's method ['lmerModLmerTest']
Formula: Movement_frequency_H2_Zone_1 ~ 1 + (1 | Genotype_Zone_1:Plant_Zone_1) +      (1 |
Genotype_Zone_2:Plant_Zone_2)
   Data: data

REML criterion at convergence: 84.8

Scaled residuals:
    Min      1Q  Median      3Q     Max
-1.7796 -0.6102 -0.0905  0.7285  1.9394

Random effects:
 Groups                       Name        Variance Std.Dev.
 Genotype_Zone_1:Plant_Zone_1 (Intercept) 0.2053   0.4531
 Genotype_Zone_2:Plant_Zone_2 (Intercept) 0.0000   0.0000
```

```
 Residual                                 0.4679   0.6840
Number of obs: 36, groups:  Genotype_Zone_1:Plant_Zone_1, 10; Genotype_Zone_2:Plant_Zone_2, 10

Fixed effects:
            Estimate Std. Error    df t value Pr(>|t|)
(Intercept)    3.859      0.184 7.735   20.97 4.27e-08 ***
---
Signif. codes:  0 '***' 0.001 '**' 0.01 '*' 0.05 '.' 0.1 ' ' 1
```

Model residuals

Statistic	Value
Sample skewness	-0.1025
Sample excess kurtosis	-0.6266
Passed Shapiro Wilk normality test	Yes (p-value = 0.4444 > 0.05)

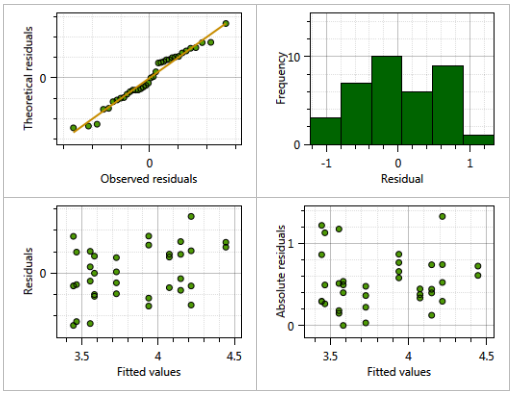

Analysis movement frequency (H2 - Zone 2)

**Analysis model**	Linear mixed model fit by REML: Movement_frequency_H2_Zone_2 ~ 1 + (1|Genotype_Zone_1:Plant_Zone_1) + (1|Genotype_Zone_2:Plant_Zone_2)
**Transformation**	Natural logarithm

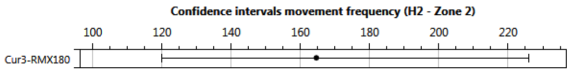

Confidence intervals movement frequency (H2 - Zone 2)

Genotype Zone 1	Genotype Zone 2	Mean	Lower 95% CL	Upper 95% CL	Group
Cur3	RMX180	164.6	119.8	226	a

Model summary

```
Linear mixed model fit by REML. t-tests use Satterthwaite's method ['lmerModLmerTest']
Formula: Movement_frequency_H2_Zone_2 ~ 1 + (1 | Genotype_Zone_1:Plant_Zone_1) +      (1 |
Genotype_Zone_2:Plant_Zone_2)
   Data: data

REML criterion at convergence: 72.1

Scaled residuals:
    Min      1Q  Median      3Q     Max
-3.0816 -0.5016  0.3670  0.5775  1.2583

Random effects:
 Groups                       Name        Variance Std.Dev.
 Genotype_Zone_1:Plant_Zone_1 (Intercept) 0.09494  0.3081
 Genotype_Zone_2:Plant_Zone_2 (Intercept) 0.00000  0.0000
 Residual                                 0.32719  0.5720
Number of obs: 37, groups:  Genotype_Zone_1:Plant_Zone_1, 10; Genotype_Zone_2:Plant_Zone_2, 10

Fixed effects:
            Estimate Std. Error     df t value Pr(>|t|)
(Intercept)   5.1034     0.1357 7.4026   37.61    1e-09 ***
---
Signif. codes:  0 '***' 0.001 '**' 0.01 '*' 0.05 '.' 0.1 ' ' 1
```

Model residuals

Statistic	Value
Sample skewness	-1.292
Sample excess kurtosis	1.905
Passed Shapiro Wilk normality test	No (p-value = 0.001727 < 0.05)

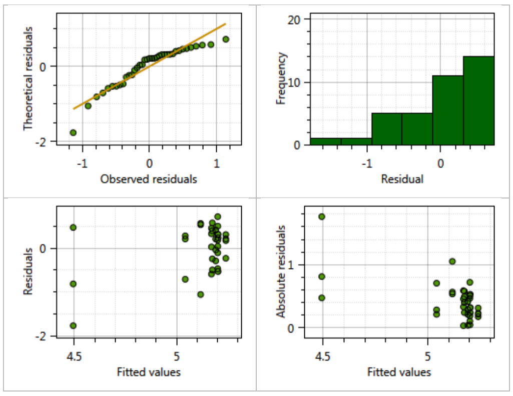

Analysis movement frequency (H3 - Zone 1)

**Analysis model**	Linear mixed model fit by REML: Movement_frequency_H3_Zone_1 ~ 1 + (1|Genotype_Zone_1:Plant_Zone_1) + (1|Genotype_Zone_2:Plant_Zone_2)
**Transformation**	Natural logarithm

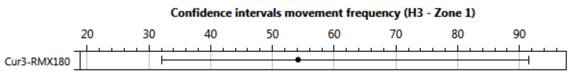

Confidence intervals movement frequency (H3 - Zone 1)

Genotype Zone 1	Genotype Zone 2	Mean	Lower 95% CL	Upper 95% CL	Group
Cur3	RMX180	54.14	32.01	91.56	a

Model summary

```
Linear mixed model fit by REML. t-tests use Satterthwaite's method ['lmerModLmerTest']
Formula: Movement_frequency_H3_Zone_1 ~ 1 + (1 | Genotype_Zone_1:Plant_Zone_1) +      (1 |
Genotype_Zone_2:Plant_Zone_2)
   Data: data

REML criterion at convergence: 94.7

Scaled residuals:
    Min      1Q  Median      3Q     Max
-3.6761 -0.4504  0.1936  0.6224  1.0698

Random effects:
 Groups                       Name        Variance Std.Dev.
 Genotype_Zone_1:Plant_Zone_1 (Intercept) 0.06913  0.2629
 Genotype_Zone_2:Plant_Zone_2 (Intercept) 0.07745  0.2783
 Residual                                 0.88832  0.9425
Number of obs: 33, groups:  Genotype_Zone_1:Plant_Zone_1, 10; Genotype_Zone_2:Plant_Zone_2, 10

Fixed effects:
            Estimate Std. Error     df t value Pr(>|t|)
(Intercept)   3.9916     0.2053 5.0774   19.44  5.8e-06 ***
---
Signif. codes:  0 '***' 0.001 '**' 0.01 '*' 0.05 '.' 0.1 ' ' 1
```

Model residuals

Statistic	Value
Sample skewness	-2.047
Sample excess kurtosis	6.328
Passed Shapiro Wilk normality test	No (p-value = 9.294E-05 < 0.05)

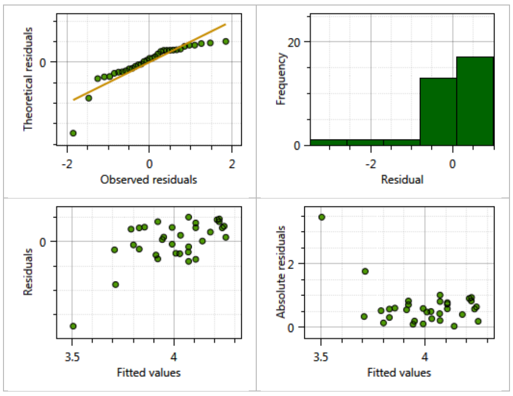

Analysis movement frequency (H3 - Zone 2)

**Analysis model**	Linear mixed model fit by REML: Movement_frequency_H3_Zone_2 ~ 1 + (1|Genotype_Zone_1:Plant_Zone_1) + (1|Genotype_Zone_2:Plant_Zone_2)
**Transformation**	Natural logarithm

Confidence intervals movement frequency (H3 - Zone 2)

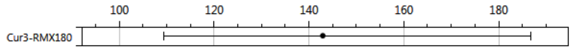

Confidence intervals movement frequency (H3 - Zone 2)

Genotype Zone 1	Genotype Zone 2	Mean	Lower 95% CL	Upper 95% CL	Group
Cur3	RMX180	142.9	109.3	186.8	a

Model summary

```
Linear mixed model fit by REML. t-tests use Satterthwaite's method ['lmerModLmerTest']
Formula: Movement_frequency_H3_Zone_2 ~ 1 + (1 | Genotype_Zone_1:Plant_Zone_1) +      (1 |
Genotype_Zone_2:Plant_Zone_2)
   Data: data

REML criterion at convergence: 81.6

Scaled residuals:
    Min      1Q  Median      3Q     Max
-3.5254 -0.3473  0.0500  0.6880  1.4767

Random effects:
 Groups                       Name        Variance Std.Dev.
 Genotype_Zone_1:Plant_Zone_1 (Intercept) 0.00000  0.0000
 Genotype_Zone_2:Plant_Zone_2 (Intercept) 0.02836  0.1684
 Residual                                 0.43146  0.6569
Number of obs: 39, groups:  Genotype_Zone_1:Plant_Zone_1, 10; Genotype_Zone_2:Plant_Zone_2, 10

Fixed effects:
            Estimate Std. Error    df t value Pr(>|t|)
(Intercept)    4.962      0.118 8.727   42.07 2.21e-11 ***
---
Signif. codes:  0 '***' 0.001 '**' 0.01 '*' 0.05 '.' 0.1 ' ' 1
```

Model residuals

Statistic	Value
Sample skewness	-1.568
Sample excess kurtosis	3.712
Passed Shapiro Wilk normality test	No (p-value = 0.0008123 < 0.05)

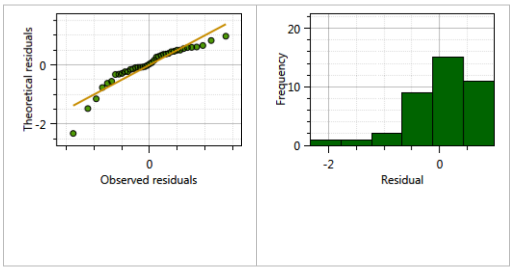

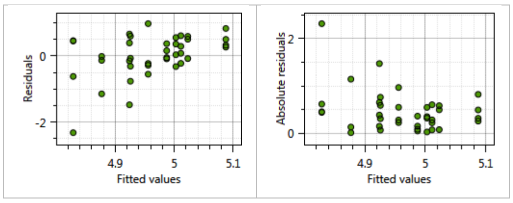

Analysis movement frequency (H4 - Zone 1)

**Analysis model**	Linear mixed model fit by REML: Movement_frequency_H4_Zone_1 ~ 1 + (1|Genotype_Zone_1:Plant_Zone_1) + (1|Genotype_Zone_2:Plant_Zone_2)
**Transformation**	Natural logarithm

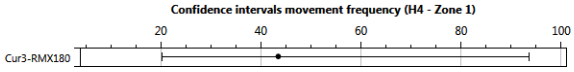

Confidence intervals movement frequency (H4 - Zone 1)

Genotype Zone 1	Genotype Zone 2	Mean	Lower 95% CL	Upper 95% CL	Group
Cur3	RMX180	43.46	20.17	93.62	a

Model summary

```
Linear mixed model fit by REML. t-tests use Satterthwaite's method ['lmerModLmerTest']
Formula: Movement_frequency_H4_Zone_1 ~ 1 + (1 | Genotype_Zone_1:Plant_Zone_1) +      (1 |
Genotype_Zone_2:Plant_Zone_2)
   Data: data

REML criterion at convergence: 84

Scaled residuals:
     Min       1Q   Median       3Q      Max
-1.85283 -0.67229 -0.05976  0.75749  1.39241

Random effects:
 Groups                       Name        Variance Std.Dev.
 Genotype_Zone_1:Plant_Zone_1 (Intercept) 0.0863   0.2938
 Genotype_Zone_2:Plant_Zone_2 (Intercept) 0.3070   0.5541
 Residual                                 0.6666   0.8165
Number of obs: 30, groups:  Genotype_Zone_1:Plant_Zone_1, 10; Genotype_Zone_2:Plant_Zone_2, 10

Fixed effects:
            Estimate Std. Error     df t value Pr(>|t|)
(Intercept)   3.7718     0.2511 3.2334   15.02 0.000422 ***
---
Signif. codes:  0 '***' 0.001 '**' 0.01 '*' 0.05 '.' 0.1 ' ' 1
```

Model residuals

Statistic	Value
Sample skewness	-0.1942
Sample excess kurtosis	-0.8818
Passed Shapiro Wilk normality test	Yes (p-value = 0.3768 > 0.05)

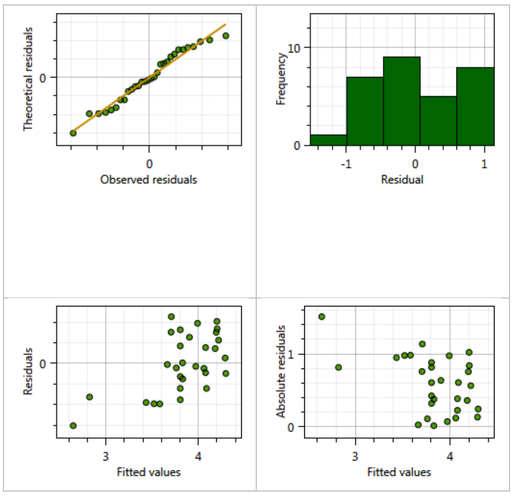

Analysis movement frequency (H4 - Zone 2)

**Analysis model**	Linear mixed model fit by REML: Movement_frequency_H4_Zone_2 ~ 1 + (1|Genotype_Zone_1:Plant_Zone_1) + (1|Genotype_Zone_2:Plant_Zone_2)
**Transformation**	Natural logarithm

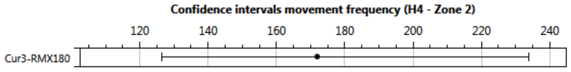

Confidence intervals movement frequency (H4 - Zone 2)

Genotype Zone 1	Genotype Zone 2	Mean	Lower 95% CL	Upper 95% CL	Group
Cur3	RMX180	171.9	126.3	233.9	a

Model summary

```
Linear mixed model fit by REML. t-tests use Satterthwaite's method ['lmerModLmerTest']
Formula: Movement_frequency_H4_Zone_2 ~ 1 + (1 | Genotype_Zone_1:Plant_Zone_1) +      (1 |
Genotype_Zone_2:Plant_Zone_2)
   Data: data

REML criterion at convergence: 71.2

Scaled residuals:
    Min      1Q  Median      3Q     Max
-3.0285 -0.4900  0.1517  0.6561  1.1644

Random effects:
 Groups                       Name        Variance Std.Dev.
 Genotype_Zone_1:Plant_Zone_1 (Intercept) 0.04563  0.2136
 Genotype_Zone_2:Plant_Zone_2 (Intercept) 0.03586  0.1894
 Residual                                 0.31882  0.5646
Number of obs: 37, groups:  Genotype_Zone_1:Plant_Zone_1, 10; Genotype_Zone_2:Plant_Zone_2, 10

Fixed effects:
            Estimate Std. Error    df t value Pr(>|t|)
(Intercept)    5.147      0.130 6.928   39.59 2.01e-09 ***
```

```
---
Signif. codes:  0 '***' 0.001 '**' 0.01 '*' 0.05 '.' 0.1 ' ' 1
```

Model residuals

Statistic	Value
Sample skewness	-1.242
Sample excess kurtosis	1.919
Passed Shapiro Wilk normality test	No (p-value = 0.004964 < 0.05)

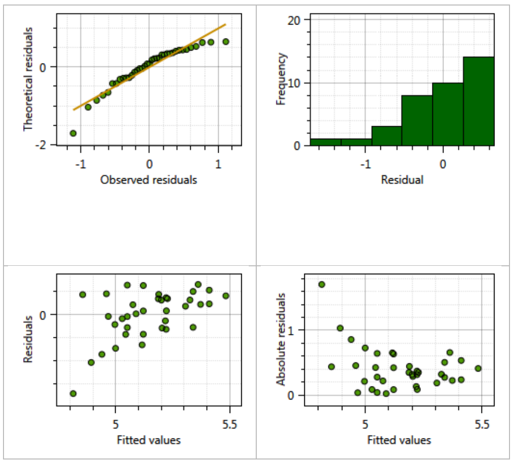

Analysis movement frequency (H5 - Zone 1)

**Analysis model**	Linear mixed model fit by REML: Movement_frequency_H5_Zone_1 ~ 1 + (1|Genotype_Zone_1:Plant_Zone_1) + (1|Genotype_Zone_2:Plant_Zone_2)
**Transformation**	Natural logarithm

Confidence intervals movement frequency (H5 - Zone 1)

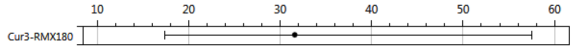

Confidence intervals movement frequency (H5 - Zone 1)

Genotype Zone 1	Genotype Zone 2	Mean	Lower 95% CL	Upper 95% CL	Group
Cur3	RMX180	31.59	17.34	57.54	a

Model summary

```
Linear mixed model fit by REML. t-tests use Satterthwaite's method ['lmerModLmerTest']
Formula: Movement_frequency_H5_Zone_1 ~ 1 + (1 | Genotype_Zone_1:Plant_Zone_1) +      (1 |
Genotype_Zone_2:Plant_Zone_2)
   Data: data

REML criterion at convergence: 107.1

Scaled residuals:
    Min      1Q  Median      3Q     Max
-2.5055 -0.3202  0.2338  0.7579  1.0646

Random effects:
 Groups                       Name        Variance Std.Dev.
 Genotype_Zone_1:Plant_Zone_1 (Intercept) 0.128    0.3578
 Genotype_Zone_2:Plant_Zone_2 (Intercept) 0.000    0.0000
 Residual                                 1.747    1.3219
Number of obs: 31, groups:  Genotype_Zone_1:Plant_Zone_1, 10; Genotype_Zone_2:Plant_Zone_2, 10

Fixed effects:
            Estimate Std. Error     df t value Pr(>|t|)
(Intercept)   3.4529     0.2644 8.8620   13.06 4.34e-07 ***
---
Signif. codes:  0 '***' 0.001 '**' 0.01 '*' 0.05 '.' 0.1 ' ' 1
```

Model residuals

Statistic	Value
Sample skewness	-1.297
Sample excess kurtosis	1.166
Passed Shapiro Wilk normality test	No (p-value = 0.0009972 < 0.05)

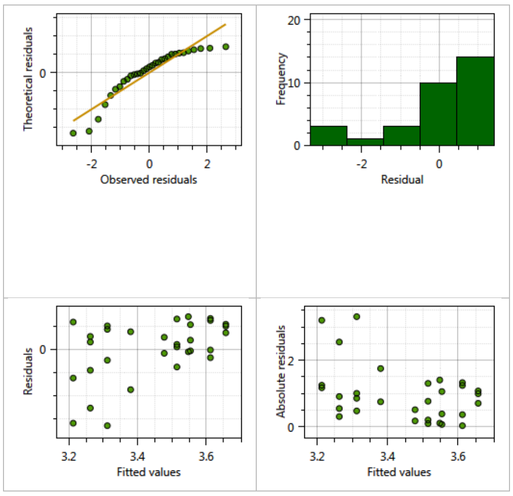

Analysis movement frequency (H5 - Zone 2)

**Analysis model**	Linear mixed model fit by REML: Movement_frequency_H5_Zone_2 ~ 1 + (1|Genotype_Zone_1:Plant_Zone_1) + (1|Genotype_Zone_2:Plant_Zone_2)
**Transformation**	Natural logarithm

Confidence intervals movement frequency (H5 - Zone 2)

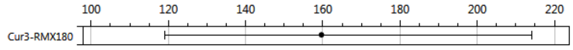

Confidence intervals movement frequency (H5 - Zone 2)

Genotype Zone 1	Genotype Zone 2	Mean	Lower 95% CL	Upper 95% CL	Group
Cur3	RMX180	159.6	119	214.2	a

Model summary

```
Linear mixed model fit by REML. t-tests use Satterthwaite's method ['lmerModLmerTest']
Formula: Movement_frequency_H5_Zone_2 ~ 1 + (1 | Genotype_Zone_1:Plant_Zone_1) +      (1 |
Genotype_Zone_2:Plant_Zone_2)
   Data: data

REML criterion at convergence: 77.6

Scaled residuals:
    Min      1Q  Median      3Q     Max
-2.2698 -0.4637  0.1441  0.5292  1.4058

Random effects:
 Groups                       Name        Variance Std.Dev.
 Genotype_Zone_1:Plant_Zone_1 (Intercept) 0.00000  0.0000
 Genotype_Zone_2:Plant_Zone_2 (Intercept) 0.07788  0.2791
 Residual                                 0.35419  0.5951
Number of obs: 39, groups:  Genotype_Zone_1:Plant_Zone_1, 10; Genotype_Zone_2:Plant_Zone_2, 10

Fixed effects:
            Estimate Std. Error    df t value Pr(>|t|)
(Intercept)    5.073      0.130 9.000   39.03 2.37e-11 ***
---
Signif. codes:  0 '***' 0.001 '**' 0.01 '*' 0.05 '.' 0.1 ' ' 1
```

Model residuals

Statistic	Value
Sample skewness	-0.6952
Sample excess kurtosis	0.09543
Passed Shapiro Wilk normality test	Yes (p-value = 0.07206 > 0.05)

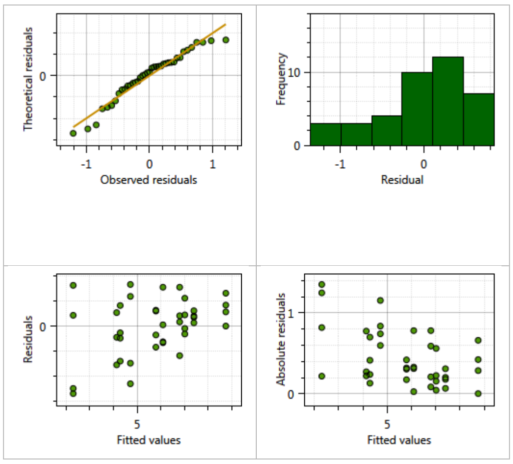

Analysis movement frequency (H6 - Zone 1)

**Analysis model**	Linear mixed model fit by REML: Movement_frequency_H6_Zone_1 ~ 1 + (1|Genotype_Zone_1:Plant_Zone_1) + (1|Genotype_Zone_2:Plant_Zone_2)
**Transformation**	Natural logarithm

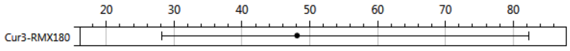

Confidence intervals movement frequency (H6 - Zone 1)

Genotype Zone 1	Genotype Zone 2	Mean	Lower 95% CL	Upper 95% CL	Group
Cur3	RMX180	48.11	28.12	82.34	a

Model summary

```
Linear mixed model fit by REML. t-tests use Satterthwaite's method ['lmerModLmerTest']
Formula: Movement_frequency_H6_Zone_1 ~ 1 + (1 | Genotype_Zone_1:Plant_Zone_1) +      (1 |
Genotype_Zone_2:Plant_Zone_2)
   Data: data

REML criterion at convergence: 69.7

Scaled residuals:
    Min      1Q  Median      3Q     Max
-2.2745 -0.6573  0.1301  0.5425  1.6557

Random effects:
 Groups                       Name        Variance Std.Dev.
 Genotype_Zone_1:Plant_Zone_1 (Intercept) 0.1418   0.3766
 Genotype_Zone_2:Plant_Zone_2 (Intercept) 0.1463   0.3825
 Residual                                 0.5347   0.7312
Number of obs: 27, groups:  Genotype_Zone_1:Plant_Zone_1, 10; Genotype_Zone_2:Plant_Zone_2, 10

Fixed effects:
            Estimate Std. Error     df t value Pr(>|t|)
(Intercept)   3.8736     0.2257 6.7739   17.17 7.79e-07 ***
---
Signif. codes:  0 '***' 0.001 '**' 0.01 '*' 0.05 '.' 0.1 ' ' 1
```

Model residuals

Statistic	Value
Sample skewness	-0.5437
Sample excess kurtosis	0.5821
Passed Shapiro Wilk normality test	Yes (p-value = 0.4612 > 0.05)

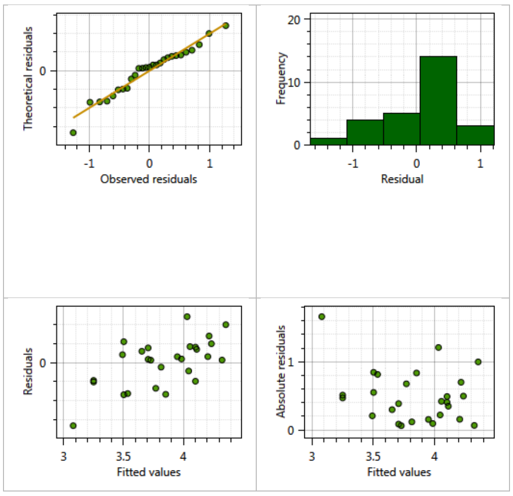

Analysis movement frequency (H6 - Zone 2)

**Analysis model**	Linear mixed model fit by REML: Movement_frequency_H6_Zone_2 ~ 1 + (1|Genotype_Zone_1:Plant_Zone_1) + (1|Genotype_Zone_2:Plant_Zone_2)
**Transformation**	Natural logarithm

Confidence intervals movement frequency (H6 - Zone 2)

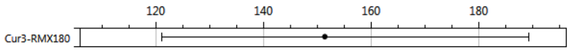

Confidence intervals movement frequency (H6 - Zone 2)

Genotype Zone 1	Genotype Zone 2	Mean	Lower 95% CL	Upper 95% CL	Group
Cur3	RMX180	151.4	121.1	189.4	a

Model summary

```
Linear mixed model fit by REML. t-tests use Satterthwaite's method ['lmerModLmerTest']
Formula: Movement_frequency_H6_Zone_2 ~ 1 + (1 | Genotype_Zone_1:Plant_Zone_1) +      (1 |
Genotype_Zone_2:Plant_Zone_2)
   Data: data

REML criterion at convergence: 80.1

Scaled residuals:
     Min       1Q   Median       3Q      Max
-2.97006 -0.38419 -0.03494  0.75310  1.45397

Random effects:
 Groups                       Name        Variance Std.Dev.
 Genotype_Zone_1:Plant_Zone_1 (Intercept) 0.0000   0.0000
 Genotype_Zone_2:Plant_Zone_2 (Intercept) 0.0000   0.0000
 Residual                                 0.4629   0.6804
Number of obs: 38, groups:  Genotype_Zone_1:Plant_Zone_1, 10; Genotype_Zone_2:Plant_Zone_2, 10

Fixed effects:
            Estimate Std. Error      df t value Pr(>|t|)
(Intercept)   5.0201     0.1104 37.0000   45.48   <2e-16 ***
---
Signif. codes:  0 '***' 0.001 '**' 0.01 '*' 0.05 '.' 0.1 ' ' 1
```

Model residuals

Statistic	Value
Sample skewness	-1.146
Sample excess kurtosis	2.34
Passed Shapiro Wilk normality test	No (p-value = 0.002569 < 0.05)

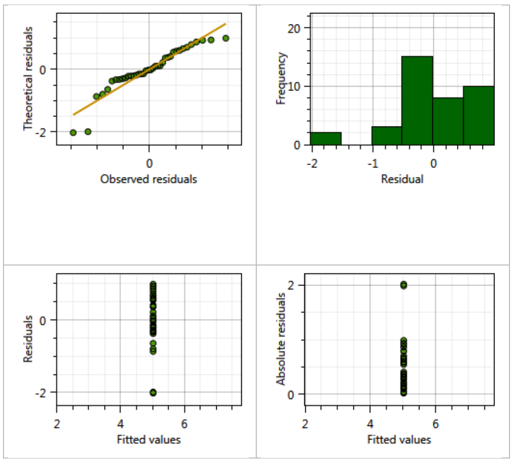

Analysis movement frequency (H7 - Zone 1)

**Analysis model**	Linear mixed model fit by REML: Movement_frequency_H7_Zone_1 ~ 1 + (1|Genotype_Zone_1:Plant_Zone_1) + (1|Genotype_Zone_2:Plant_Zone_2)
**Transformation**	Natural logarithm

Confidence intervals movement frequency (H7 - Zone 1)

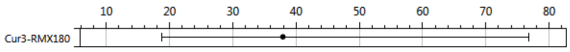

Confidence intervals movement frequency (H7 - Zone 1)

Genotype Zone 1	Genotype Zone 2	Mean	Lower 95% CL	Upper 95% CL	Group
Cur3	RMX180	37.9	18.7	76.82	a

Model summary

```
Linear mixed model fit by REML. t-tests use Satterthwaite's method ['lmerModLmerTest']
Formula: Movement_frequency_H7_Zone_1 ~ 1 + (1 | Genotype_Zone_1:Plant_Zone_1) +      (1 |
Genotype_Zone_2:Plant_Zone_2)
   Data: data
```

```
REML criterion at convergence: 94.2

Scaled residuals:
     Min       1Q   Median       3Q      Max
-2.65520 -0.42229 -0.02987  0.77728  1.33975

Random effects:
 Groups                      Name        Variance Std.Dev.
 Genotype_Zone_2:Plant_Zone_2 (Intercept) 0.10745  0.3278
 Genotype_Zone_1:Plant_Zone_1 (Intercept) 0.03373  0.1837
 Residual                                1.57040  1.2532
Number of obs: 28, groups:  Genotype_Zone_2:Plant_Zone_2, 10; Genotype_Zone_1:Plant_Zone_1, 9

Fixed effects:
            Estimate Std. Error     df t value Pr(>|t|)
(Intercept)   3.6350     0.2687 4.6535   13.53  6.5e-05 ***
---
Signif. codes:  0 '***' 0.001 '**' 0.01 '*' 0.05 '.' 0.1 ' ' 1
```

Model residuals

Statistic	Value
Sample skewness	-1.168
Sample excess kurtosis	2.006
Passed Shapiro Wilk normality test	No (p-value = 0.006614 < 0.05)

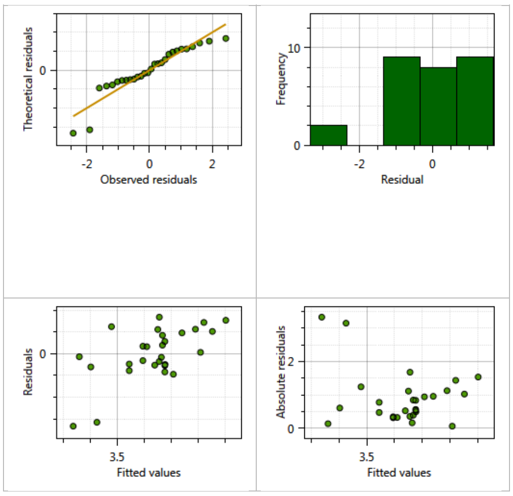

Analysis movement frequency (H7 - Zone 2)

**Analysis model**	Linear mixed model fit by REML: Movement_frequency_H7_Zone_2 ~ 1 + (1|Genotype_Zone_1:Plant_Zone_1) + (1|Genotype_Zone_2:Plant_Zone_2)
**Transformation**	Natural logarithm

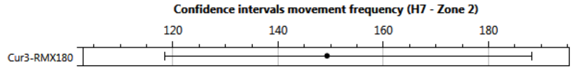

Confidence intervals movement frequency (H7 - Zone 2)

Genotype Zone 1	Genotype Zone 2	Mean	Lower 95% CL	Upper 95% CL	Group
Cur3	RMX180	149.3	118.4	188.3	a

Model summary

```
Linear mixed model fit by REML. t-tests use Satterthwaite's method ['lmerModLmerTest']
Formula: Movement_frequency_H7_Zone_2 ~ 1 + (1 | Genotype_Zone_1:Plant_Zone_1) +      (1 |
Genotype_Zone_2:Plant_Zone_2)
   Data: data

REML criterion at convergence: 74.3

Scaled residuals:
     Min       1Q   Median       3Q      Max
-2.74382 -0.75013 -0.01363  0.74837  1.61592

Random effects:
 Groups                      Name        Variance Std.Dev.
 Genotype_Zone_1:Plant_Zone_1 (Intercept) 0.00000  0.0000
 Genotype_Zone_2:Plant_Zone_2 (Intercept) 0.01267  0.1126
 Residual                                0.36412  0.6034
Number of obs: 39, groups:  Genotype_Zone_1:Plant_Zone_1, 10; Genotype_Zone_2:Plant_Zone_2, 10

Fixed effects:
            Estimate Std. Error    df t value Pr(>|t|)
(Intercept)    5.006      0.103 9.265    48.6 1.79e-12 ***
---
Signif. codes:  0 '***' 0.001 '**' 0.01 '*' 0.05 '.' 0.1 ' ' 1
```

Model residuals

Statistic	Value
Sample skewness	-0.4542
Sample excess kurtosis	-0.02928
Passed Shapiro Wilk normality test	Yes (p-value = 0.2733 > 0.05)

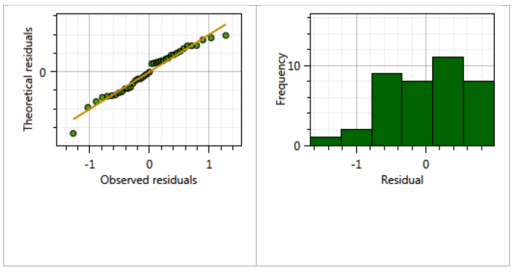

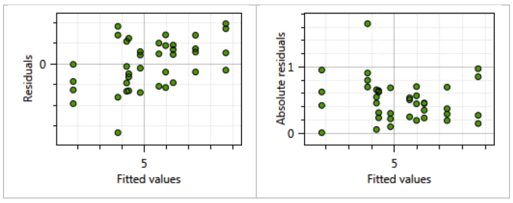

Analysis movement frequency H0 (diff. Zone 1 - Zone 2)

**Analysis model**	Generalized linear mixed model with dispersion factor, formula=cbind(Movement_frequency_H0_Zone_1,Movement_frequency_H0_Zone_2) ~ 1 + (1|Genotype_Zone_1:Plant_Zone_1) + (1|Genotype_Zone_2:Plant_Zone_2)
**Transformation**	Logit

Confidence intervals movement frequency H0 (diff. Zone 1 - Zone 2)

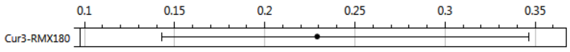

Confidence intervals movement frequency H0 (diff. Zone 1 - Zone 2)

Genotype Zone 1	Genotype Zone 2	Mean	Lower 95% CL	Upper 95% CL	Group
Cur3	RMX180	0.229	0.1427	0.3466	a

Model summary

```
Linear mixed model fit by REML. t-tests use Satterthwaite's method ['lmerModLmerTest']
Formula: ziFormula
   Data: data
Weights: wi

REML criterion at convergence: 123.5

Scaled residuals:
    Min      1Q  Median      3Q     Max
-1.4233 -0.7970 -0.0802  0.5153  2.5229

Random effects:
 Groups                      Name        Variance Std.Dev.
 Genotype_Zone_1:Plant_Zone_1 (Intercept)  0.0000  0.0000
 Genotype_Zone_2:Plant_Zone_2 (Intercept)  0.2933  0.5416
 Residual                                49.3229  7.0230
Number of obs: 37, groups:  Genotype_Zone_1:Plant_Zone_1, 10; Genotype_Zone_2:Plant_Zone_2, 10

Fixed effects:
            Estimate Std. Error      df t value Pr(>|t|)
(Intercept)  -1.2137     0.2512  7.9709  -4.832  0.00131 **
---
Signif. codes:  0 '***' 0.001 '**' 0.01 '*' 0.05 '.' 0.1 ' ' 1

Dispersion: 7.023
```

Model residuals

Statistic	Value
Sample skewness	0.709
Sample excess kurtosis	0.1934
Passed Shapiro Wilk normality test	Yes (p-value = 0.1506 > 0.05)

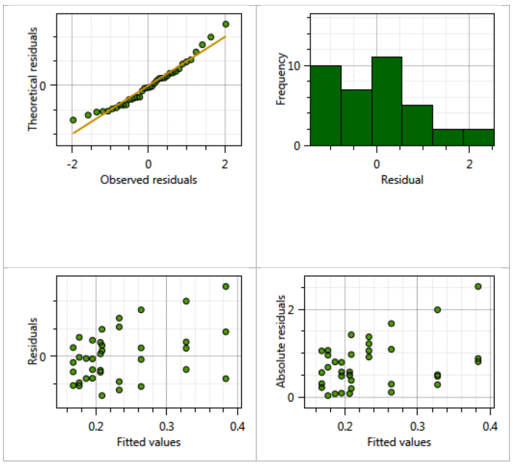

Analysis movement frequency H1 (diff. Zone 1 - Zone 2)

**Analysis model**	Generalized linear mixed model with dispersion factor, formula=cbind(Movement_frequency_H1_Zone_1,Movement_frequency_H1_Zone_2) ~ 1 + (1|Genotype_Zone_1:Plant_Zone_1) + (1|Genotype_Zone_2:Plant_Zone_2)
**Transformation**	Logit

Confidence intervals movement frequency H1 (diff. Zone 1 - Zone 2)

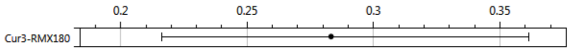

Confidence intervals movement frequency H1 (diff. Zone 1 - Zone 2)

Genotype Zone 1	Genotype Zone 2	Mean	Lower 95% CL	Upper 95% CL	Group
Cur3	RMX180	0.2832	0.2161	0.3615	a

Model summary

```
Linear mixed model fit by REML. t-tests use Satterthwaite's method ['lmerModLmerTest']
Formula: ziFormula
   Data: data
Weights: wi

REML criterion at convergence: 112.8

Scaled residuals:
     Min       1Q   Median       3Q      Max
-1.48203 -0.63845 -0.03252  0.94577  2.13787

Random effects:
 Groups                      Name        Variance Std.Dev.
 Genotype_Zone_1:Plant_Zone_1 (Intercept)  0.00    0.000
 Genotype_Zone_2:Plant_Zone_2 (Intercept)  0.00    0.000
 Residual                                57.41    7.577
Number of obs: 37, groups:  Genotype_Zone_1:Plant_Zone_1, 10; Genotype_Zone_2:Plant_Zone_2, 10

Fixed effects:
            Estimate Std. Error      df t value Pr(>|t|)
(Intercept)  -0.9287     0.1774 36.0000  -5.234 7.34e-06 ***
```

```
---
Signif. codes:  0 '***' 0.001 '**' 0.01 '*' 0.05 '.' 0.1 ' ' 1

Dispersion: 7.577
```

Model residuals

Statistic	Value
Sample skewness	0.4101
Sample excess kurtosis	-0.9063
Passed Shapiro Wilk normality test	Yes (p-value = 0.1155 > 0.05)

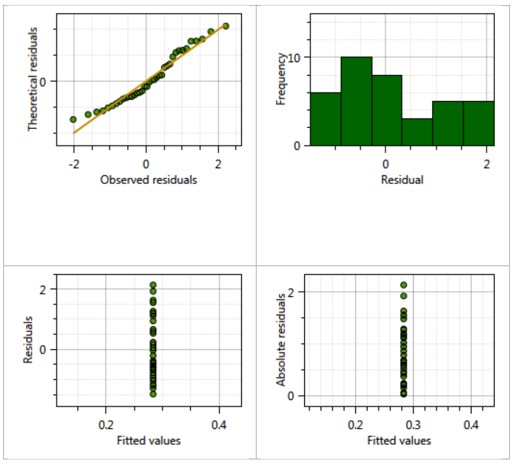

Analysis movement frequency H2 (diff. Zone 1 - Zone 2)

**Analysis model**	Generalized linear mixed model with dispersion factor, formula=cbind(Movement_frequency_H2_Zone_1,Movement_frequency_H2_Zone_2) ~ 1 + (1|Genotype_Zone_1:Plant_Zone_1) + (1|Genotype_Zone_2:Plant_Zone_2)
**Transformation**	Logit

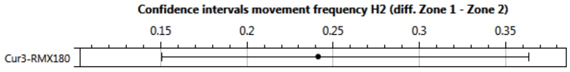

Confidence intervals movement frequency H2 (diff. Zone 1 - Zone 2)

Genotype Zone 1	Genotype Zone 2	Mean	Lower 95% CL	Upper 95% CL	Group
Cur3	RMX180	0.2413	0.1503	0.3637	a

Model summary

```
Linear mixed model fit by REML. t-tests use Satterthwaite's method ['lmerModLmerTest']
Formula: ziFormula
   Data: data
Weights: wi

REML criterion at convergence: 134.9

Scaled residuals:
    Min      1Q  Median      3Q     Max
-1.1195 -0.6358 -0.3006  0.5467  2.8347

Random effects:
 Groups                      Name        Variance Std.Dev.
 Genotype_Zone_1:Plant_Zone_1 (Intercept)  0.228   0.4775
 Genotype_Zone_2:Plant_Zone_2 (Intercept)  0.000   0.0000
 Residual                                 69.492   8.3362
Number of obs: 39, groups:  Genotype_Zone_1:Plant_Zone_1, 10; Genotype_Zone_2:Plant_Zone_2, 10

Fixed effects:
            Estimate Std. Error      df t value Pr(>|t|)
(Intercept)  -1.1456     0.2535  7.8644   -4.52  0.00204 **
---
Signif. codes:  0 '***' 0.001 '**' 0.01 '*' 0.05 '.' 0.1 ' ' 1

Dispersion: 8.336
```

Model residuals

Statistic	Value
Sample skewness	1.177
Sample excess kurtosis	0.9162
Passed Shapiro Wilk normality test	No (p-value = 0.0008287 < 0.05)

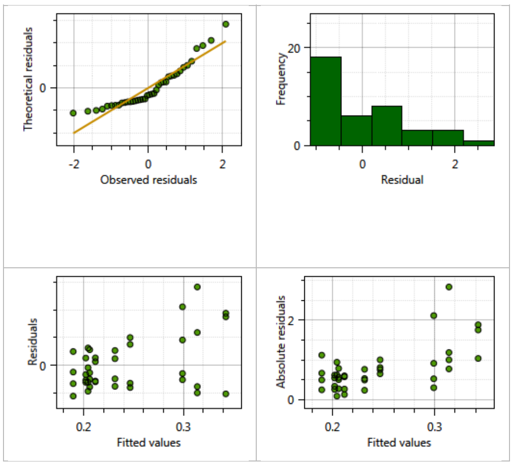

Analysis movement frequency H3 (diff. Zone 1 - Zone 2)

**Analysis model**	Generalized linear mixed model with dispersion factor, formula=cbind(Movement_frequency_H3_Zone_1,Movement_frequency_H3_Zone_2) ~ 1 + (1|Genotype_Zone_1:Plant_Zone_1) + (1|Genotype_Zone_2:Plant_Zone_2)
**Transformation**	Logit

Confidence intervals movement frequency H3 (diff. Zone 1 - Zone 2)

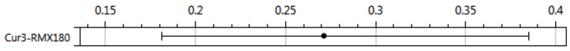

Confidence intervals movement frequency H3 (diff. Zone 1 - Zone 2)

Genotype Zone 1	Genotype Zone 2	Mean	Lower 95% CL	Upper 95% CL	Group
Cur3	RMX180	0.2712	0.181	0.3852	a

Model summary

```
Linear mixed model fit by REML. t-tests use Satterthwaite's method ['lmerModLmerTest']
Formula: ziFormula
   Data: data
Weights: wi

REML criterion at convergence: 127.7

Scaled residuals: 
     Min       1Q   Median       3Q      Max 
-1.44982 -0.80017 -0.04012  0.78323  2.18963 

Random effects:
 Groups                      Name        Variance Std.Dev.
 Genotype_Zone_1:Plant_Zone_1 (Intercept)  0.0000  0.0000 
 Genotype_Zone_2:Plant_Zone_2 (Intercept)  0.1881  0.4337 
 Residual                                60.3729  7.7700 
Number of obs: 39, groups:  Genotype_Zone_1:Plant_Zone_1, 10; Genotype_Zone_2:Plant_Zone_2, 10

Fixed effects:
            Estimate Std. Error      df t value Pr(>|t|)   
(Intercept)  -0.9885     0.2310  9.2096  -4.278  0.00195 **
---
Signif. codes:  0 '***' 0.001 '**' 0.01 '*' 0.05 '.' 0.1 ' ' 1

Dispersion: 7.77
```

Model residuals

Statistic	Value
Sample skewness	0.4272
Sample excess kurtosis	-0.728
Passed Shapiro Wilk normality test	Yes (p-value = 0.1025 > 0.05)

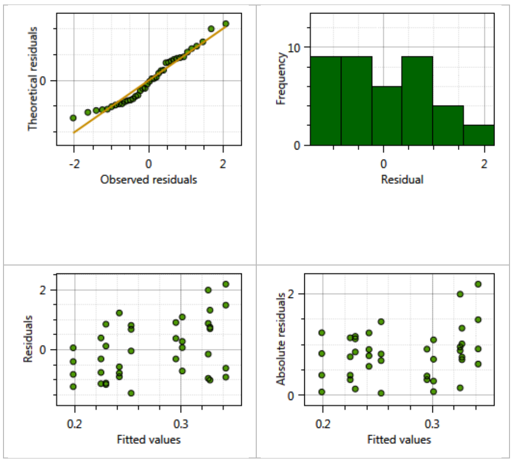

Analysis movement frequency H4 (diff. Zone 1 - Zone 2)

**Analysis model**	Generalized linear mixed model with dispersion factor, formula=cbind(Movement_frequency_H4_Zone_1,Movement_frequency_H4_Zone_2) ~ 1 + (1|Genotype_Zone_1:Plant_Zone_1) + (1|Genotype_Zone_2:Plant_Zone_2)
**Transformation**	Logit

Confidence intervals movement frequency H4 (diff. Zone 1 - Zone 2)

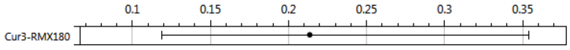

Confidence intervals movement frequency H4 (diff. Zone 1 - Zone 2)

Genotype Zone 1	Genotype Zone 2	Mean	Lower 95% CL	Upper 95% CL	Group
Cur3	RMX180	0.2135	0.1185	0.3541	a

Model summary

```
Linear mixed model fit by REML. t-tests use Satterthwaite's method ['lmerModLmerTest']
Formula: ziFormula
   Data: data
Weights: wi

REML criterion at convergence: 143.7

Scaled residuals: 
    Min      1Q  Median      3Q     Max 
-1.0556 -0.7543 -0.1994  0.6292  2.3748 

Random effects:
 Groups                      Name        Variance Std.Dev.
 Genotype_Zone_1:Plant_Zone_1 (Intercept)  0.2470  0.4970 
 Genotype_Zone_2:Plant_Zone_2 (Intercept)  0.1315  0.3627 
 Residual                                76.0190  8.7189 
Number of obs: 39, groups:  Genotype_Zone_1:Plant_Zone_1, 10; Genotype_Zone_2:Plant_Zone_2, 10

Fixed effects:
            Estimate Std. Error      df t value Pr(>|t|)   
(Intercept)  -1.3037     0.2968  6.9665  -4.392  0.00322 **
---
Signif. codes:  0 '***' 0.001 '**' 0.01 '*' 0.05 '.' 0.1 ' ' 1

Dispersion: 8.719
```

Model residuals

Statistic	Value
Sample skewness	0.8499
Sample excess kurtosis	-0.317
Passed Shapiro Wilk normality test	No (p-value = 0.001467 < 0.05)

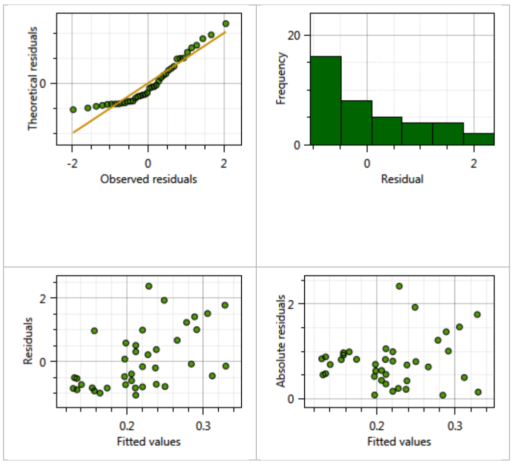

Analysis movement frequency H5 (diff. Zone 1 - Zone 2)

**Analysis model**	Generalized linear mixed model with dispersion factor, formula=cbind(Movement_frequency_H5_Zone_1,Movement_frequency_H5_Zone_2) ~ 1 + (1|Genotype_Zone_1:Plant_Zone_1) + (1|Genotype_Zone_2:Plant_Zone_2)
**Transformation**	Logit

Confidence intervals movement frequency H5 (diff. Zone 1 - Zone 2)

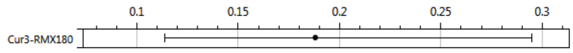

Confidence intervals movement frequency H5 (diff. Zone 1 - Zone 2)

Genotype Zone 1	Genotype Zone 2	Mean	Lower 95% CL	Upper 95% CL	Group
Cur3	RMX180	0.188	0.1136	0.295	a

Model summary

```
Linear mixed model fit by REML. t-tests use Satterthwaite's method ['lmerModLmerTest']
Formula: ziFormula
   Data: data
Weights: wi

REML criterion at convergence: 142.1

Scaled residuals:
    Min      1Q  Median      3Q     Max
-1.1534 -0.7190 -0.1474  0.7145  2.1905

Random effects:
 Groups                        Name        Variance  Std.Dev.
 Genotype_Zone_1:Plant_Zone_1 (Intercept) 1.825e-15 4.272e-08
 Genotype_Zone_2:Plant_Zone_2 (Intercept) 1.775e-01 4.214e-01
 Residual                                 6.999e+01 8.366e+00
Number of obs: 39, groups:  Genotype_Zone_1:Plant_Zone_1, 10; Genotype_Zone_2:Plant_Zone_2, 10

Fixed effects:
            Estimate Std. Error      df t value Pr(>|t|)
(Intercept)  -1.4629     0.2633  9.4043  -5.556 0.000302 ***
---
Signif. codes:  0 '***' 0.001 '**' 0.01 '*' 0.05 '.' 0.1 ' ' 1

Dispersion: 8.366
```

Model residuals

Statistic	Value
Sample skewness	0.7425
Sample excess kurtosis	-0.6345
Passed Shapiro Wilk normality test	No (p-value = 0.002305 < 0.05)

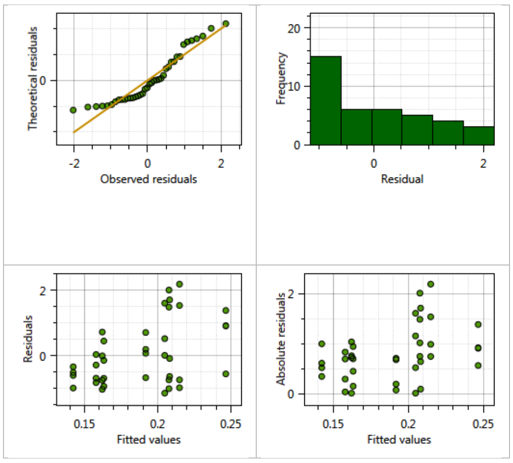

Analysis movement frequency H6 (diff. Zone 1 - Zone 2)

**Analysis model**	Generalized linear mixed model with dispersion factor, formula=cbind(Movement_frequency_H6_Zone_1,Movement_frequency_H6_Zone_2) ~ 1 + (1|Genotype_Zone_1:Plant_Zone_1) + (1|Genotype_Zone_2:Plant_Zone_2)
**Transformation**	Logit

Confidence intervals movement frequency H6 (diff. Zone 1 - Zone 2)

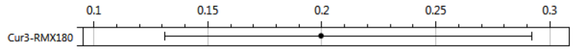

Confidence intervals movement frequency H6 (diff. Zone 1 - Zone 2)

Genotype Zone 1	Genotype Zone 2	Mean	Lower 95% CL	Upper 95% CL	Group
Cur3	RMX180	0.1996	0.1309	0.2922	a

Model summary

```
Linear mixed model fit by REML. t-tests use Satterthwaite's method ['lmerModLmerTest']
Formula: ziFormula
   Data: data
Weights: wi
```

```
REML criterion at convergence: 148

Scaled residuals: 
    Min      1Q  Median      3Q     Max 
-1.0895 -0.7448 -0.1537  0.6849  3.1492 

Random effects:
 Groups                   Name        Variance Std.Dev.
 Genotype_Zone_1:Plant_Zone_1 (Intercept)  0.00    0.00   
 Genotype_Zone_2:Plant_Zone_2 (Intercept)  0.00    0.00   
 Residual                             85.56    9.25   
Number of obs: 39, groups:  Genotype_Zone_1:Plant_Zone_1, 10; Genotype_Zone_2:Plant_Zone_2, 10

Fixed effects:
            Estimate Std. Error     df t value Pr(>|t|)    
(Intercept)   -1.389      0.249 38.000  -5.577 2.16e-06 ***
---
Signif. codes:  0 '***' 0.001 '**' 0.01 '*' 0.05 '.' 0.1 ' ' 1

Dispersion: 9.25
```

Model residuals

Statistic	Value
Sample skewness	1.058
Sample excess kurtosis	1.111
Passed Shapiro Wilk normality test	No (p-value = 0.003135 < 0.05)

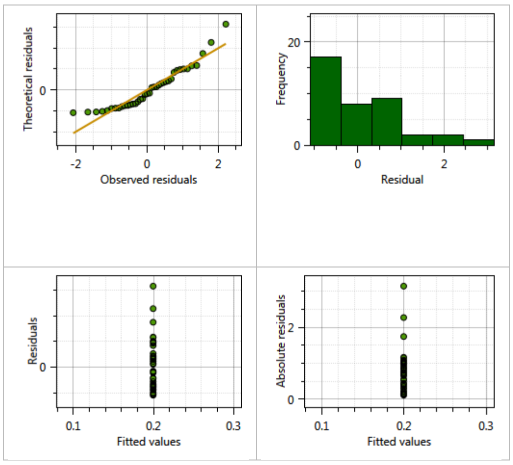

Data points with high residuals

Trial	Arena
Trial 3	11

Analysis movement frequency H7 (diff. Zone 1 - Zone 2)

**Analysis model**	Generalized linear mixed model with dispersion factor, formula=cbind(Movement_frequency_H7_Zone_1,Movement_frequency_H7_Zone_2) ~ 1 + (1|Genotype_Zone_1:Plant_Zone_1) + (1|Genotype_Zone_2:Plant_Zone_2)
**Transformation**	Logit

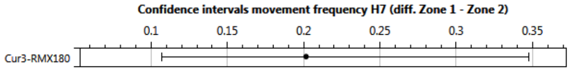

Confidence intervals movement frequency H7 (diff. Zone 1 - Zone 2)

Genotype Zone 1	Genotype Zone 2	Mean	Lower 95% CL	Upper 95% CL	Group
Cur3	RMX180	0.2015	0.1067	0.3477	a

Model summary

```
Linear mixed model fit by REML. t-tests use Satterthwaite's method ['lmerModLmerTest']
Formula: ziFormula
   Data: data
Weights: wi

REML criterion at convergence: 147.1

Scaled residuals: 
    Min      1Q  Median      3Q     Max 
-1.3494 -0.6825 -0.2940  0.7823  2.0353 

Random effects:
 Groups                   Name        Variance Std.Dev.
 Genotype_Zone_1:Plant_Zone_1 (Intercept)  0.4005  0.6328  
 Genotype_Zone_2:Plant_Zone_2 (Intercept)  0.1419  0.3767  
 Residual                             67.8711  8.2384  
Number of obs: 39, groups:  Genotype_Zone_1:Plant_Zone_1, 10; Genotype_Zone_2:Plant_Zone_2, 10

Fixed effects:
            Estimate Std. Error      df t value Pr(>|t|)   
(Intercept)  -1.3769     0.3243  8.0078  -4.245  0.00281 **
---
Signif. codes:  0 '***' 0.001 '**' 0.01 '*' 0.05 '.' 0.1 ' ' 1

Dispersion: 8.238
```

Model residuals

Statistic	Value
Sample skewness	0.8232
Sample excess kurtosis	-0.3231
Passed Shapiro Wilk normality test	No (p-value = 0.003357 < 0.05)

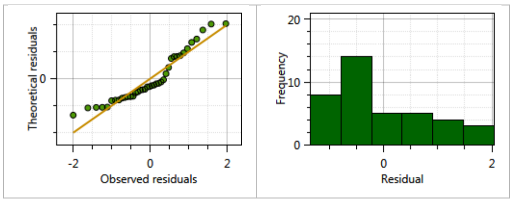

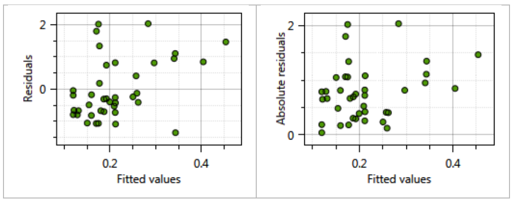

Movement frequency per zone per short/medium/long

Selected zones	Zone 1, Zone 2
Event duration categories	duration < 2, 2 <= duration < 5, duration >= 5
Data transformation	Natural logarithm
Analysis	Zone difference analysis

Model result movement frequency per zone per short/medium/long

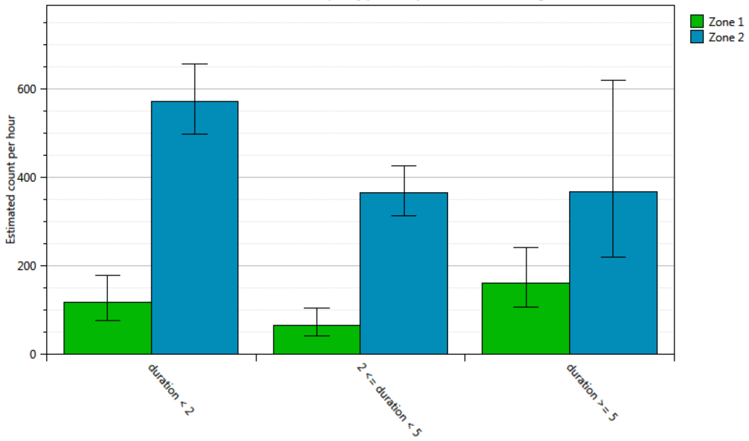

Results difference tests Zone 1 - Zone 2: p values and 95% confidence intervals of the difference on the transformed scale for each statistic.

Behaviour statistic	Cur3-RMX180	Remark
Movement frequency duration < 2 (diff. Zone 1 - Zone 2)	p=0.000342***<br>[-1.86, -0.909]	
Movement frequency 2 <= duration < 5 (diff. Zone 1 - Zone 2)	p=2.7E-05****<br>[-2.01, -1.11]	CR
Movement frequency duration >= 5 (diff. Zone 1 - Zone 2)	p=0.000128***<br>[-1.18, -0.54]	

CR = Check residuals

The model predictions and 95% confidence intervals for each statistic.

Statistic	Cur3-RMX180	Remark
Movement frequency (duration < 2 - Zone 1)	116<br>[75.3, 178]	CR
Movement frequency (2 <= duration < 5 - Zone 1)	64.9<br>[40.4, 104]	CR
Movement frequency (duration >= 5 - Zone 1)	160<br>[107, 241]	CR
Movement frequency (duration < 2 - Zone 2)	572<br>[498, 657]	
Movement frequency (2 <= duration < 5 - Zone 2)	365<br>[313, 426]	
Movement frequency (duration >= 5 - Zone 2)	368<br>[219, 619]	CR

CR = Check residuals

Data summary

Movement frequency per zone per short/medium/long

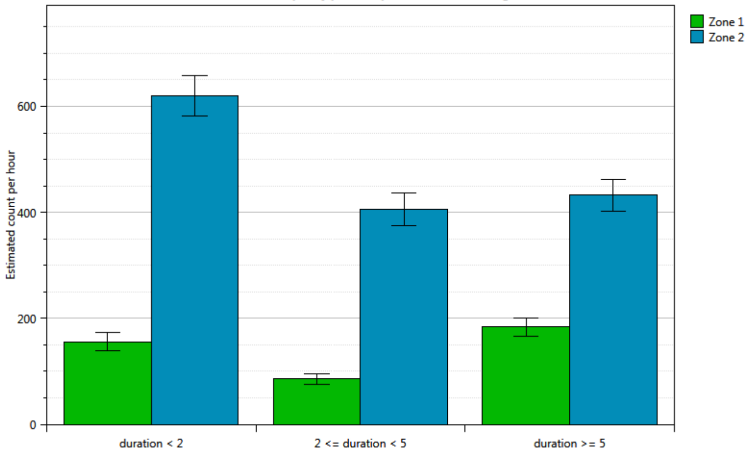

Genotype Zone 1	Genotype Zone 2	Genotype Zone 3	Mean duration < 2 - Zone 1	StdErr duration < 2 - Zone 1	Mean 2 <= duration < 5 - Zone 1	StdErr 2 <= duration < 5 - Zone 1	Mean duration >= 5 - Zone 1	StdErr duration >= 5 - Zone 1	Mean duration < 2 - Zone 2	StdErr duration < 2 - Zone 2	Mean 2 <= duration < 5 - Zone 2	StdErr 2 <= duration < 5 - Zone 2	Mean duration >= 5 - Zone 2	StdErr duration >= 5 - Zone 2
Cur3	RMX180	Neutral	155.92	17.86	85.64	10.2	183.41	17.52	619.92	38.16	405.7	30.02	432.36	30.08

Analysis movement frequency (duration < 2 - Zone 1)

**Analysis model**	Linear mixed model fit by REML: Movement_frequency_duration_2_Zone_1 ~ 1 + (1|Genotype_Zone_1:Plant_Zone_1) + (1|Genotype_Zone_2:Plant_Zone_2)
**Transformation**	Natural logarithm

Confidence intervals movement frequency (duration < 2 - Zone 1)

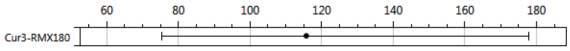

Confidence intervals movement frequency (duration < 2 - Zone 1)

Genotype Zone 1	Genotype Zone 2	Mean	Lower 95% CL	Upper 95% CL	Group
Cur3	RMX180	115.7	75.25	177.9	a

Model summary

```
Linear mixed model fit by REML. t-tests use Satterthwaite's method ['lmerModLmerTest']
Formula: Movement_frequency_duration_2_Zone_1 ~ 1 + (1 | Genotype_Zone_1:Plant_Zone_1) +      (1 |
Genotype_Zone_2:Plant_Zone_2)
   Data: data

REML criterion at convergence: 110.7

Scaled residuals:
    Min      1Q  Median      3Q     Max
-3.9915 -0.2757  0.1980  0.4990  1.2057

Random effects:
 Groups                       Name        Variance  Std.Dev.
 Genotype_Zone_1:Plant_Zone_1 (Intercept) 1.076e-16 1.037e-08
 Genotype_Zone_2:Plant_Zone_2 (Intercept) 1.124e-01 3.353e-01
 Residual                                 9.665e-01 9.831e-01
Number of obs: 38, groups:  Genotype_Zone_1:Plant_Zone_1, 10; Genotype_Zone_2:Plant_Zone_2, 10

Fixed effects:
            Estimate Std. Error     df t value Pr(>|t|)
(Intercept)   4.7511     0.1917 9.5006   24.78 5.93e-10 ***
---
Signif. codes:  0 '***' 0.001 '**' 0.01 '*' 0.05 '.' 0.1 ' ' 1
```

Model residuals

Statistic	Value
Sample skewness	-2.231
Sample excess kurtosis	7.31
Passed Shapiro Wilk normality test	No (p-value = 1.549E-05 < 0.05)

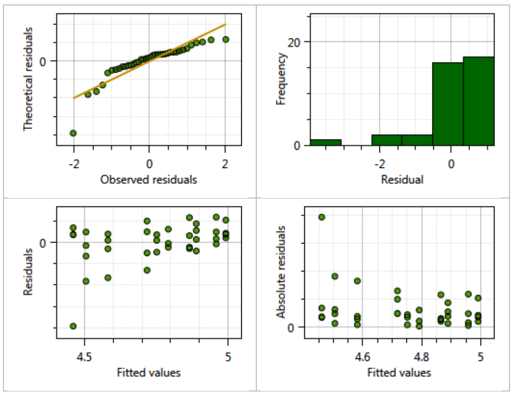

Analysis movement frequency (2 <= duration < 5 - Zone 1)

**Analysis model**	Linear mixed model fit by REML: Movement_frequency_2_duration_5_Zone_1 ~ 1 + (1|Genotype_Zone_1:Plant_Zone_1) + (1|Genotype_Zone_2:Plant_Zone_2)
**Transformation**	Natural logarithm

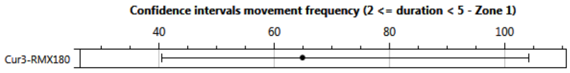

Confidence intervals movement frequency (2 <= duration < 5 - Zone 1)

Genotype Zone 1	Genotype Zone 2	Mean	Lower 95% CL	Upper 95% CL	Group
Cur3	RMX180	64.89	40.4	104.3	a

Model summary

```
Linear mixed model fit by REML. t-tests use Satterthwaite's method ['lmerModLmerTest']
Formula: Movement_frequency_2_duration_5_Zone_1 ~ 1 + (1 | Genotype_Zone_1:Plant_Zone_1) +      (1 |
Genotype_Zone_2:Plant_Zone_2)
   Data: data

REML criterion at convergence: 104.1

Scaled residuals:
    Min      1Q  Median      3Q     Max
-3.8906 -0.2335  0.1984  0.5504  1.3423

Random effects:
 Groups                       Name        Variance Std.Dev.
 Genotype_Zone_1:Plant_Zone_1 (Intercept) 0.14750  0.3841
 Genotype_Zone_2:Plant_Zone_2 (Intercept) 0.05295  0.2301
 Residual                                 0.73172  0.8554
Number of obs: 38, groups:  Genotype_Zone_1:Plant_Zone_1, 10; Genotype_Zone_2:Plant_Zone_2, 10

Fixed effects:
            Estimate Std. Error     df t value Pr(>|t|)
(Intercept)   4.1728     0.1986 6.6984   21.01  2.3e-07 ***
---
Signif. codes:  0 '***' 0.001 '**' 0.01 '*' 0.05 '.' 0.1 ' ' 1
```

Model residuals

Statistic	Value
Sample skewness	-2.177
Sample excess kurtosis	7.597
Passed Shapiro Wilk normality test	No (p-value = 4.491E-05 < 0.05)

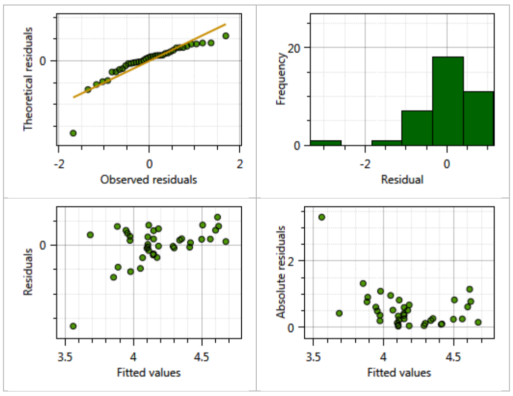

Analysis movement frequency (duration >= 5 - Zone 1)

**Analysis model**	Linear mixed model fit by REML: Movement_frequency_duration_5_Zone_1 ~ 1 + (1|Genotype_Zone_1:Plant_Zone_1) + (1|Genotype_Zone_2:Plant_Zone_2)
**Transformation**	Natural logarithm

Confidence intervals movement frequency (duration >= 5 - Zone 1)

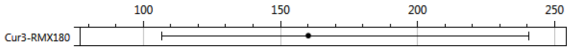

Confidence intervals movement frequency (duration >= 5 - Zone 1)

Genotype Zone 1	Genotype Zone 2	Mean	Lower 95% CL	Upper 95% CL	Group
Cur3	RMX180	160.2	106.5	240.7	a

Model summary

```
Linear mixed model fit by REML. t-tests use Satterthwaite's method ['lmerModLmerTest']
Formula: Movement_frequency_duration_5_Zone_1 ~ 1 + (1 | Genotype_Zone_1:Plant_Zone_1) +      (1 |
Genotype_Zone_2:Plant_Zone_2)
   Data: data

REML criterion at convergence: 81.9

Scaled residuals: 
    Min      1Q  Median      3Q     Max 
-3.5887 -0.3495  0.0277  0.6395  1.1247 

Random effects:
 Groups                       Name        Variance Std.Dev.
 Genotype_Zone_1:Plant_Zone_1 (Intercept) 0.10163  0.3188  
 Genotype_Zone_2:Plant_Zone_2 (Intercept) 0.08903  0.2984  
 Residual                                 0.37968  0.6162  
Number of obs: 37, groups:  Genotype_Zone_1:Plant_Zone_1, 10; Genotype_Zone_2:Plant_Zone_2, 10

Fixed effects:
            Estimate Std. Error     df t value Pr(>|t|)    
(Intercept)   5.0761     0.1721 6.9571   29.49 1.45e-08 ***
---
Signif. codes:  0 '***' 0.001 '**' 0.01 '*' 0.05 '.' 0.1 ' ' 1
```

Model residuals

Statistic	Value
Sample skewness	-1.855
Sample excess kurtosis	6.352
Passed Shapiro Wilk normality test	No (p-value = 0.0002255 < 0.05)

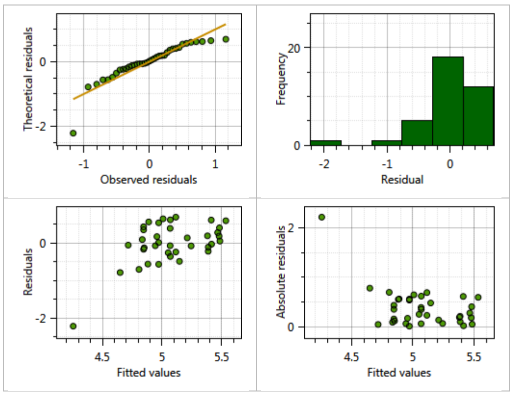

Analysis movement frequency (duration < 2 - Zone 2)

**Analysis model**	Linear mixed model fit by REML: Movement_frequency_duration_2_Zone_2 ~ 1 + (1|Genotype_Zone_1:Plant_Zone_1) + (1|Genotype_Zone_2:Plant_Zone_2)
**Transformation**	Natural logarithm

Confidence intervals movement frequency (duration < 2 - Zone 2)

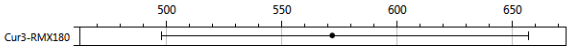

Confidence intervals movement frequency (duration < 2 - Zone 2)

Genotype Zone 1	Genotype Zone 2	Mean	Lower 95% CL	Upper 95% CL	Group
Cur3	RMX180	571.8	497.6	657.1	a

Model summary

```
Linear mixed model fit by REML. t-tests use Satterthwaite's method ['lmerModLmerTest']
Formula: Movement_frequency_duration_2_Zone_2 ~ 1 + (1 | Genotype_Zone_1:Plant_Zone_1) +      (1 |
Genotype_Zone_2:Plant_Zone_2)
   Data: data

REML criterion at convergence: 47.1

Scaled residuals: 
    Min      1Q  Median      3Q     Max 
-3.2251 -0.6806  0.1122  0.7484  1.6316 

Random effects:
 Groups                       Name        Variance Std.Dev.
 Genotype_Zone_1:Plant_Zone_1 (Intercept) 0.0000   0.0000  
 Genotype_Zone_2:Plant_Zone_2 (Intercept) 0.0000   0.0000  
 Residual                                 0.1837   0.4286  
Number of obs: 39, groups:  Genotype_Zone_1:Plant_Zone_1, 10; Genotype_Zone_2:Plant_Zone_2, 10

Fixed effects:
            Estimate Std. Error       df t value Pr(>|t|)    
(Intercept)  6.34882    0.06864 38.00000    92.5   <2e-16 ***
---
Signif. codes:  0 '***' 0.001 '**' 0.01 '*' 0.05 '.' 0.1 ' ' 1
```

Model residuals

Statistic	Value
Sample skewness	-0.8299
Sample excess kurtosis	1.463
Passed Shapiro Wilk normality test	Yes (p-value = 0.0846 > 0.05)

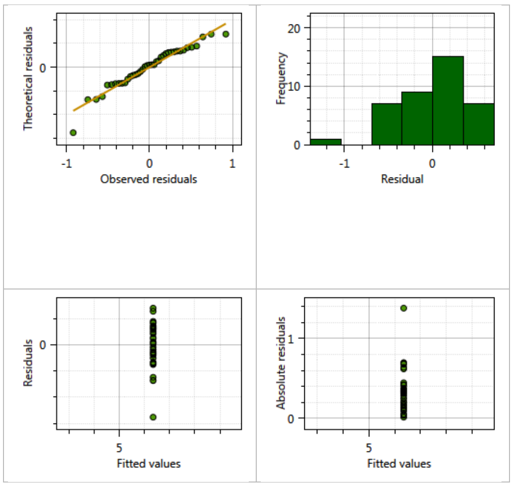

Analysis movement frequency (2 <= duration < 5 - Zone 2)

**Analysis model**	Linear mixed model fit by REML: Movement_frequency_2_duration_5_Zone_2 ~ 1 + (1|Genotype_Zone_1:Plant_Zone_1) + (1|Genotype_Zone_2:Plant_Zone_2)
**Transformation**	Natural logarithm

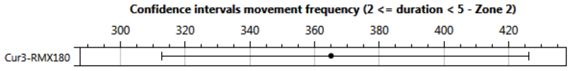

Confidence intervals movement frequency (2 <= duration < 5 - Zone 2)

Genotype Zone 1	Genotype Zone 2	Mean	Lower 95% CL	Upper 95% CL	Group
Cur3	RMX180	365.1	312.7	426.3	a

Model summary

```
Linear mixed model fit by REML. t-tests use Satterthwaite's method ['lmerModLmerTest']
Formula: Movement_frequency_2_duration_5_Zone_2 ~ 1 + (1 | Genotype_Zone_1:Plant_Zone_1) +      (1 |
Genotype_Zone_2:Plant_Zone_2)
   Data: data

REML criterion at convergence: 55.4

Scaled residuals: 
    Min      1Q  Median      3Q     Max 
-2.8532 -0.5796 -0.1040  0.7626  2.0273 

Random effects:
 Groups                       Name        Variance Std.Dev.
 Genotype_Zone_1:Plant_Zone_1 (Intercept) 0.0000   0.0000  
 Genotype_Zone_2:Plant_Zone_2 (Intercept) 0.0000   0.0000  
 Residual                                 0.2287   0.4782  
Number of obs: 39, groups:  Genotype_Zone_1:Plant_Zone_1, 10; Genotype_Zone_2:Plant_Zone_2, 10

Fixed effects:
            Estimate Std. Error       df t value Pr(>|t|)    
(Intercept)  5.90012    0.07658 38.00000   77.05   <2e-16 ***
---
Signif. codes:  0 '***' 0.001 '**' 0.01 '*' 0.05 '.' 0.1 ' ' 1
```

Model residuals

Statistic	Value
Sample skewness	-0.3765
Sample excess kurtosis	0.4729
Passed Shapiro Wilk normality test	Yes (p-value = 0.7817 > 0.05)

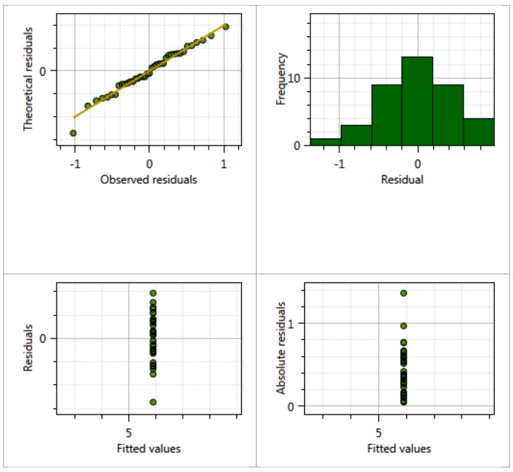

Analysis movement frequency (duration >= 5 - Zone 2)

**Analysis model**	Linear mixed model fit by REML: Movement_frequency_duration_5_Zone_2 ~ 1 + (1|Genotype_Zone_1:Plant_Zone_1) + (1|Genotype_Zone_2:Plant_Zone_2)
**Transformation**	Natural logarithm

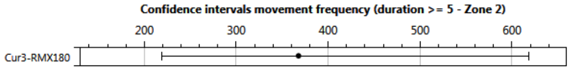

Confidence intervals movement frequency (duration >= 5 - Zone 2)

Genotype Zone 1	Genotype Zone 2	Mean	Lower 95% CL	Upper 95% CL	Group
Cur3	RMX180	367.7	218.6	618.6	a

Model summary

```
Linear mixed model fit by REML. t-tests use Satterthwaite's method ['lmerModLmerTest']
Formula: Movement_frequency_duration_5_Zone_2 ~ 1 + (1 | Genotype_Zone_1:Plant_Zone_1) +      (1 |
Genotype_Zone_2:Plant_Zone_2)
   Data: data
```

```
REML criterion at convergence: 81.8

Scaled residuals:
    Min      1Q  Median      3Q     Max
-3.1962 -0.2123  0.2149  0.6371  0.9041

Random effects:
 Groups                        Name        Variance Std.Dev.
 Genotype_Zone_1:Plant_Zone_1 (Intercept) 0.03361  0.1833
 Genotype_Zone_2:Plant_Zone_2 (Intercept) 0.04472  0.2115
 Residual                                 0.39390  0.6276
Number of obs: 39, groups:  Genotype_Zone_1:Plant_Zone_1, 10; Genotype_Zone_2:Plant_Zone_2, 10

Fixed effects:
            Estimate Std. Error    df t value Pr(>|t|)
(Intercept)    5.907      0.134 2.247   44.07  0.00024 ***
---
Signif. codes:  0 '***' 0.001 '**' 0.01 '*' 0.05 '.' 0.1 ' ' 1
```

Model residuals

Statistic	Value
Sample skewness	-2.094
Sample excess kurtosis	4.613
Passed Shapiro Wilk normality test	No (p-value = 1.497E-06 < 0.05)

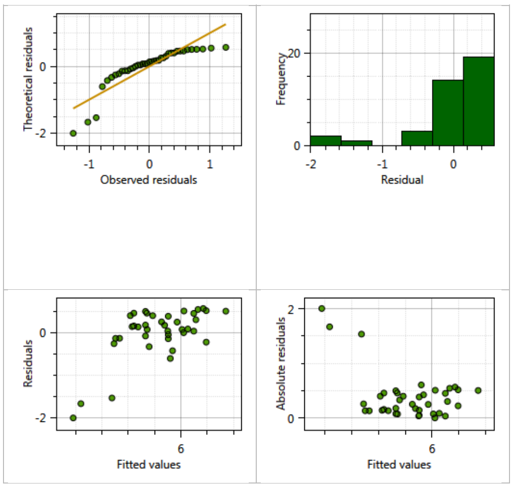

Analysis movement frequency duration < 2 (diff. Zone 1 - Zone 2)

**Analysis model**	Generalized linear mixed model with dispersion factor, formula=cbind(Movement_frequency_duration_2_Zone_1,Movement_frequency_duration_2_Zone_2) ~ 1 + (1|Genotype_Zone_1:Plant_Zone_1) + (1|Genotype_Zone_2:Plant_Zone_2)
**Transformation**	Logit

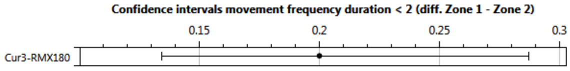

Confidence intervals movement frequency duration < 2 (diff. Zone 1 - Zone 2)

Genotype Zone 1	Genotype Zone 2	Mean	Lower 95% CL	Upper 95% CL	Group
Cur3	RMX180	0.2	0.1342	0.2873	a

Model summary

```
Linear mixed model fit by REML. t-tests use Satterthwaite's method ['lmerModLmerTest']
Formula: ziFormula
   Data: data
Weights: wi

REML criterion at convergence: 112.5

Scaled residuals:
     Min       1Q   Median       3Q      Max
-1.49276 -0.57059  0.00217  0.53921  2.39490

Random effects:
 Groups                        Name        Variance  Std.Dev.
 Genotype_Zone_1:Plant_Zone_1 (Intercept)   0.03599  0.1897
 Genotype_Zone_2:Plant_Zone_2 (Intercept)   0.12768  0.3573
 Residual                                 107.40165 10.3635
Number of obs: 39, groups:  Genotype_Zone_1:Plant_Zone_1, 10; Genotype_Zone_2:Plant_Zone_2, 10

Fixed effects:
            Estimate Std. Error      df t value Pr(>|t|)
(Intercept)  -1.3865     0.1973  6.2677  -7.026 0.000342 ***
---
Signif. codes:  0 '***' 0.001 '**' 0.01 '*' 0.05 '.' 0.1 ' ' 1

Dispersion: 10.36
```

Model residuals

Statistic	Value
Sample skewness	0.6672
Sample excess kurtosis	0.1855
Passed Shapiro Wilk normality test	Yes (p-value = 0.1459 > 0.05)

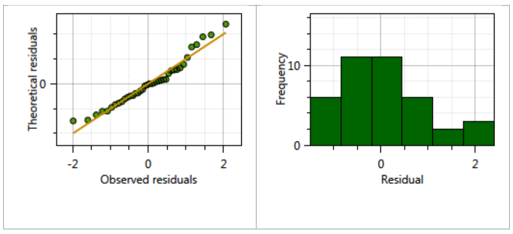

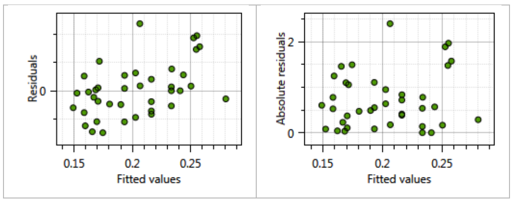

Analysis movement frequency 2 <= duration < 5 (diff. Zone 1 - Zone 2)

**Analysis model**	Generalized linear mixed model with dispersion factor, formula=cbind(Movement_frequency_2_duration_5_Zone_1,Movement_frequency_2_duration_5_Zone_2) ~ 1 + (1|Genotype_Zone_1:Plant_Zone_1) + (1|Genotype_Zone_2:Plant_Zone_2)
**Transformation**	Logit

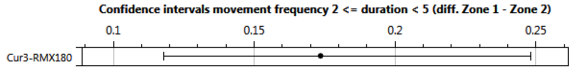

Confidence intervals movement frequency 2 <= duration < 5 (diff. Zone 1 - Zone 2)

Genotype Zone 1	Genotype Zone 2	Mean	Lower 95% CL	Upper 95% CL	Group
Cur3	RMX180	0.1735	0.1178	0.2483	a

Model summary

```
Linear mixed model fit by REML. t-tests use Satterthwaite's method ['lmerModLmerTest']
Formula: ziFormula
   Data: data
Weights: wi

REML criterion at convergence: 109.1

Scaled residuals:
     Min       1Q   Median       3Q      Max
-1.36119 -0.71427 -0.07216  0.55318  1.82377

Random effects:
 Groups                        Name        Variance Std.Dev.
 Genotype_Zone_1:Plant_Zone_1 (Intercept)  0.07562 0.2750
 Genotype_Zone_2:Plant_Zone_2 (Intercept)  0.13474 0.3671
 Residual                                 50.22089 7.0867
Number of obs: 39, groups:  Genotype_Zone_1:Plant_Zone_1, 10; Genotype_Zone_2:Plant_Zone_2, 10

Fixed effects:
            Estimate Std. Error      df t value Pr(>|t|)
(Intercept)  -1.5608     0.2003  9.0150  -7.793  2.7e-05 ***
---
Signif. codes:  0 '***' 0.001 '**' 0.01 '*' 0.05 '.' 0.1 ' ' 1

Dispersion: 7.087
```

Model residuals

Statistic	Value
Sample skewness	0.4924
Sample excess kurtosis	-0.7928
Passed Shapiro Wilk normality test	No (p-value = 0.02897 < 0.05)

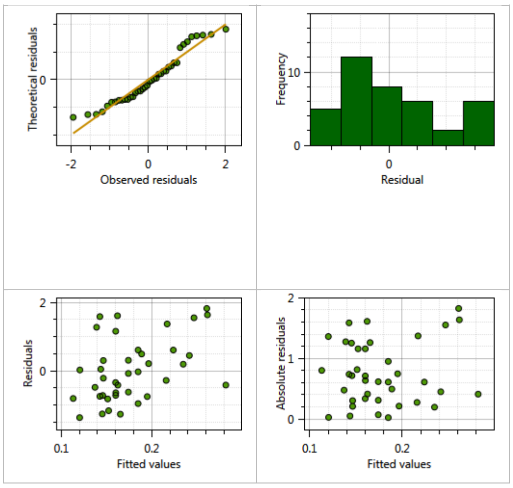

Analysis movement frequency duration >= 5 (diff. Zone 1 - Zone 2)

**Analysis model**	Generalized linear mixed model with dispersion factor, formula=cbind(Movement_frequency_duration_5_Zone_1,Movement_frequency_duration_5_Zone_2) ~ 1 + (1|Genotype_Zone_1:Plant_Zone_1) + (1|Genotype_Zone_2:Plant_Zone_2)
**Transformation**	Logit

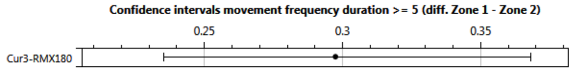

Confidence intervals movement frequency duration >= 5 (diff. Zone 1 - Zone 2)

Genotype Zone 1	Genotype Zone 2	Mean	Lower 95% CL	Upper 95% CL	Group
Cur3	RMX180	0.2974	0.2352	0.3682	a

Model summary

```
Linear mixed model fit by REML. t-tests use Satterthwaite's method ['lmerModLmerTest']
Formula: ziFormula
   Data: data
Weights: wi

REML criterion at convergence: 86.2

Scaled residuals:
    Min      1Q  Median      3Q     Max
-1.6577 -0.8465 -0.1737  0.7169  1.9831

Random effects:
 Groups                        Name        Variance Std.Dev.
 Genotype_Zone_1:Plant_Zone_1 (Intercept)  0.04091 0.2023
 Genotype_Zone_2:Plant_Zone_2 (Intercept)  0.06893 0.2626
 Residual                                 45.88681 6.7740
Number of obs: 39, groups:  Genotype_Zone_1:Plant_Zone_1, 10; Genotype_Zone_2:Plant_Zone_2, 10
```

```
Fixed effects:
            Estimate Std. Error      df t value Pr(>|t|)
(Intercept)  -0.8597     0.1437 10.1336  -5.982 0.000128 ***
---
Signif. codes:  0 '***' 0.001 '**' 0.01 '*' 0.05 '.' 0.1 ' ' 1

Dispersion: 6.774
```

Model residuals

Statistic	Value
Sample skewness	0.1663
Sample excess kurtosis	-0.9196
Passed Shapiro Wilk normality test	Yes (p-value = 0.2829 > 0.05)

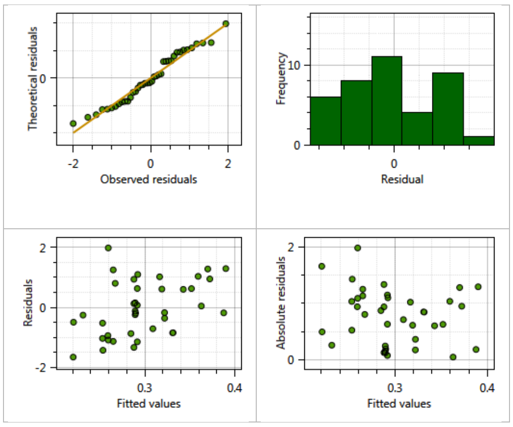

Movement frequency per zone per slow/medium/fast

Selected zones	Zone 1, Zone 2
Velocity categories	velocity < 0.025, 0.025 <= velocity < 0.075, velocity >= 0.075
Data transformation	Natural logarithm
Analysis	Zone difference analysis

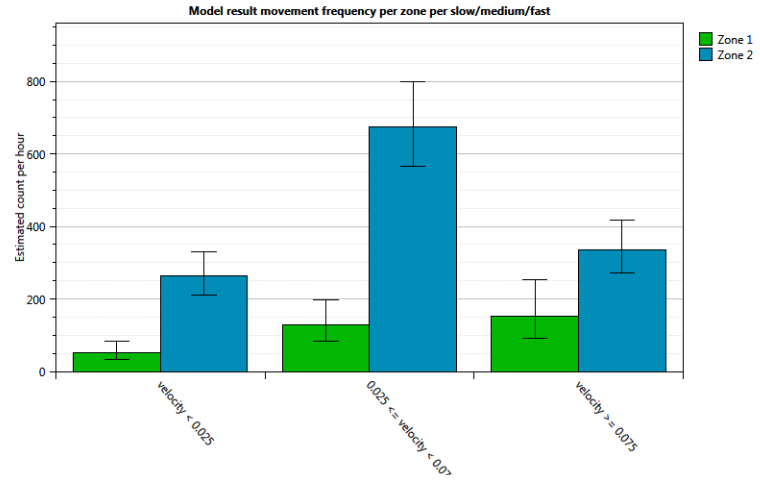

Results difference tests Zone 1 - Zone 2: p values and 95% confidence intervals of the difference on the transformed scale for each statistic.

Behaviour statistic	Cur3-RMX180	Remark
Movement frequency velocity < 0.025 (diff. Zone 1 - Zone 2)	p=5.69E-05****<br>[-1.88, -0.958]	CR
Movement frequency 0.025 <= velocity < 0.075 (diff. Zone 1 - Zone 2)	p=7.51E-05****<br>[-2.1, -1.1]	
Movement frequency velocity >= 0.075 (diff. Zone 1 - Zone 2)	p=3.56E-05****<br>[-0.897, -0.468]	

CR = Check residuals

The model predictions and 95% confidence intervals for each statistic.

Statistic	Cur3-RMX180	Remark
Movement frequency (velocity < 0.025 - Zone 1)	51.7<br>[32.1, 83.4]	CR
Movement frequency (0.025 <= velocity < 0.075 - Zone 1)	129<br>[83.5, 199]	CR
Movement frequency (velocity >= 0.075 - Zone 1)	153<br>[92, 254]	CR
Movement frequency (velocity < 0.025 - Zone 2)	264<br>[211, 330]	
Movement frequency (0.025 <= velocity < 0.075 - Zone 2)	673<br>[567, 800]	
Movement frequency (velocity >= 0.075 - Zone 2)	336<br>[271, 417]	CR

CR = Check residuals

Data summary

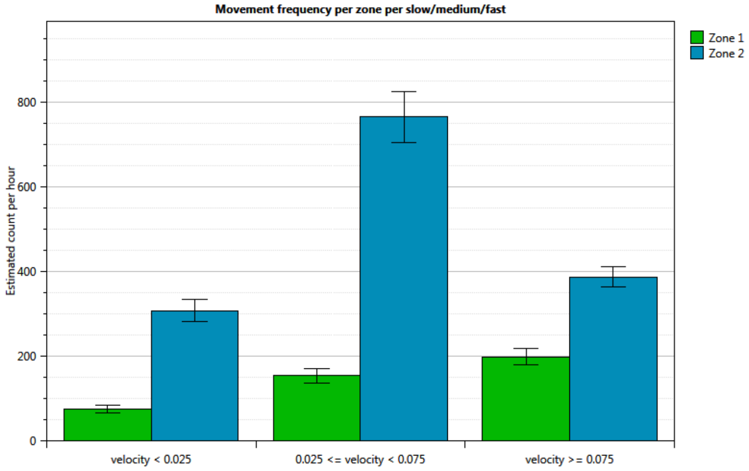

Genotype Zone 1	Genotype Zone 2	Genotype Zone 3	Mean velocity < 0.025 - Zone 1	StdErr velocity < 0.025 - Zone 1	Mean 0.025 <= velocity < 0.075 - Zone 1	StdErr 0.025 <= velocity < 0.075 - Zone 1	Mean velocity >= 0.075 - Zone 1	StdErr velocity >= 0.075 - Zone 1	Mean velocity < 0.025 - Zone 2	StdErr velocity < 0.025 - Zone 2	Mean 0.025 <= velocity < 0.075 - Zone 2	StdErr 0.025 <= velocity < 0.075 - Zone 2	Mean velocity >= 0.075 - Zone 2	StdErr velocity >= 0.075 - Zone 2
Cur3	RMX180	Neutral	73.99	9.33	152.99	17.24	197.99	20.23	307.17	25.3	764.7	60.22	386.11	23.72

Analysis movement frequency (velocity < 0.025 - Zone 1)

**Analysis model**	Linear mixed model fit by REML: Movement_frequency_velocity_0_025_Zone_1 ~ 1 + (1|Genotype_Zone_1:Plant_Zone_1) + (1|Genotype_Zone_2:Plant_Zone_2)
**Transformation**	Natural logarithm

Confidence intervals movement frequency (velocity < 0.025 - Zone 1)

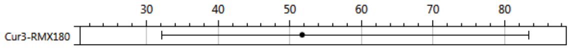

Confidence intervals movement frequency (velocity < 0.025 - Zone 1)

Genotype Zone 1	Genotype Zone 2	Mean	Lower 95% CL	Upper 95% CL	Group
Cur3	RMX180	51.72	32.07	83.41	a

Model summary

```
Linear mixed model fit by REML. t-tests use Satterthwaite's method ['lmerModLmerTest']
Formula: Movement_frequency_velocity_0_025_Zone_1 ~ 1 + (1 | Genotype_Zone_1:Plant_Zone_1) +      (1 |
Genotype_Zone_2:Plant_Zone_2)
   Data: data

REML criterion at convergence: 113.2

Scaled residuals:
    Min      1Q  Median      3Q     Max
-3.1319 -0.2760  0.2319  0.6168  1.2604

Random effects:
 Groups                       Name        Variance Std.Dev.
 Genotype_Zone_1:Plant_Zone_1 (Intercept) 0.0000   0.0000
 Genotype_Zone_2:Plant_Zone_2 (Intercept) 0.1892   0.4349
 Residual                                 0.9903   0.9952
Number of obs: 38, groups:  Genotype_Zone_1:Plant_Zone_1, 10; Genotype_Zone_2:Plant_Zone_2, 10

Fixed effects:
            Estimate Std. Error     df t value Pr(>|t|)
(Intercept)   3.9459     0.2124 9.3310   18.58  1.1e-08 ***
---
Signif. codes:  0 '***' 0.001 '**' 0.01 '*' 0.05 '.' 0.1 ' ' 1
```

Model residuals

Statistic	Value
Sample skewness	-1.586
Sample excess kurtosis	3.284
Passed Shapiro Wilk normality test	No (p-value = 0.0004368 < 0.05)

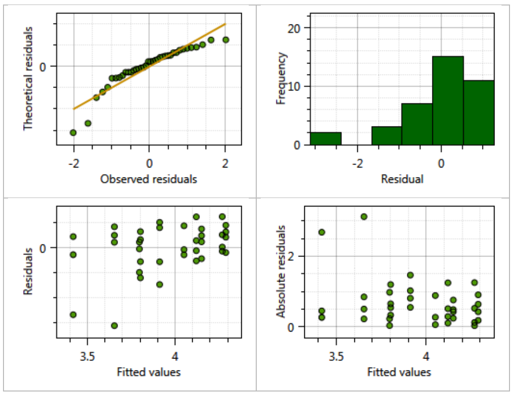

Analysis movement frequency (0.025 <= velocity < 0.075 - Zone 1)

**Analysis model**	Linear mixed model fit by REML: Movement_frequency_0_025_velocity_0_075_Zone_1 ~ 1 + (1|Genotype_Zone_1:Plant_Zone_1) + (1|Genotype_Zone_2:Plant_Zone_2)
**Transformation**	Natural logarithm

Confidence intervals movement frequency (0.025 <= velocity < 0.075 - Zone 1)

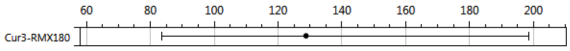

Confidence intervals movement frequency (0.025 <= velocity < 0.075 - Zone 1)

Genotype Zone 1	Genotype Zone 2	Mean	Lower 95% CL	Upper 95% CL	Group
Cur3	RMX180	128.8	83.48	198.6	a

Model summary

```
Linear mixed model fit by REML. t-tests use Satterthwaite's method ['lmerModLmerTest']
```

```
Formula: Movement_frequency_0_025_velocity_0_075_Zone_1 ~ 1 + (1 | Genotype_Zone_1:Plant_Zone_1) +
(1 | Genotype_Zone_2:Plant_Zone_2)
   Data: data

REML criterion at convergence: 85

Scaled residuals:
     Min       1Q   Median       3Q      Max
-2.43866 -0.22146 -0.01825  0.44274  1.75614

Random effects:
 Groups                      Name        Variance Std.Dev.
 Genotype_Zone_1:Plant_Zone_1 (Intercept) 0.1238   0.3519
 Genotype_Zone_2:Plant_Zone_2 (Intercept) 0.1235   0.3515
 Residual                                0.3922   0.6263
Number of obs: 37, groups:  Genotype_Zone_1:Plant_Zone_1, 10; Genotype_Zone_2:Plant_Zone_2, 10

Fixed effects:
            Estimate Std. Error     df t value Pr(>|t|)
(Intercept)   4.8580     0.1889 8.2429   25.71 3.65e-09 ***
---
Signif. codes:  0 '***' 0.001 '**' 0.01 '*' 0.05 '.' 0.1 ' ' 1
```

Model residuals

Statistic	Value
Sample skewness	-0.8413
Sample excess kurtosis	1.34
Passed Shapiro Wilk normality test	No (p-value = 0.04054 < 0.05)

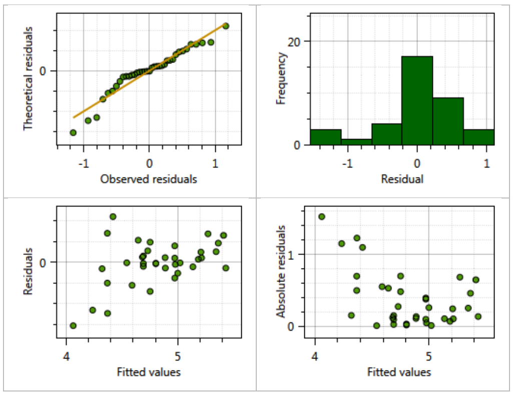

Analysis movement frequency (velocity >= 0.075 - Zone 1)

**Analysis model**	Linear mixed model fit by REML: Movement_frequency_velocity_0_075_Zone_1 ~ 1 + (1|Genotype_Zone_1:Plant_Zone_1) + (1|Genotype_Zone_2:Plant_Zone_2)
**Transformation**	Natural logarithm

Confidence intervals movement frequency (velocity >= 0.075 - Zone 1)

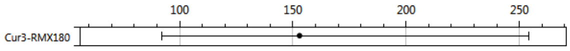

Confidence intervals movement frequency (velocity >= 0.075 - Zone 1)

Genotype Zone 1	Genotype Zone 2	Mean	Lower 95% CL	Upper 95% CL	Group
Cur3	RMX180	152.9	91.97	254.3	a

Model summary

```
Linear mixed model fit by REML. t-tests use Satterthwaite's method ['lmerModLmerTest']
Formula: Movement_frequency_velocity_0_075_Zone_1 ~ 1 + (1 | Genotype_Zone_1:Plant_Zone_1) +      (1 |
Genotype_Zone_2:Plant_Zone_2)
   Data: data

REML criterion at convergence: 108.5

Scaled residuals:
    Min      1Q  Median      3Q     Max
-4.3884 -0.1214  0.0344  0.5302  1.0545

Random effects:
 Groups                      Name        Variance Std.Dev.
 Genotype_Zone_1:Plant_Zone_1 (Intercept) 0.17675  0.4204
 Genotype_Zone_2:Plant_Zone_2 (Intercept) 0.03493  0.1869
 Residual                                0.83815  0.9155
Number of obs: 38, groups:  Genotype_Zone_1:Plant_Zone_1, 10; Genotype_Zone_2:Plant_Zone_2, 10

Fixed effects:
            Estimate Std. Error     df t value Pr(>|t|)
(Intercept)   5.0299     0.2083 6.0609   24.15 2.96e-07 ***
---
Signif. codes:  0 '***' 0.001 '**' 0.01 '*' 0.05 '.' 0.1 ' ' 1
```

Model residuals

Statistic	Value
Sample skewness	-3.016
Sample excess kurtosis	13.09
Passed Shapiro Wilk normality test	No (p-value = 4.391E-07 < 0.05)

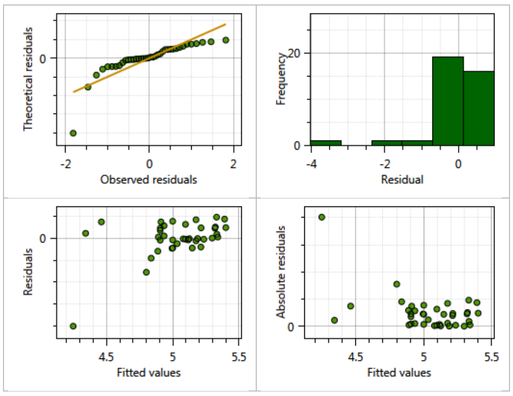

Analysis movement frequency (velocity < 0.025 - Zone 2)

**Analysis model**	Linear mixed model fit by REML: Movement_frequency_velocity_0_025_Zone_2 ~ 1 + (1|Genotype_Zone_1:Plant_Zone_1) + (1|Genotype_Zone_2:Plant_Zone_2)
**Transformation**	Natural logarithm

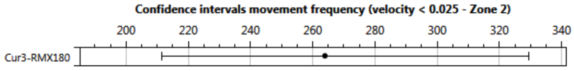

Confidence intervals movement frequency (velocity < 0.025 - Zone 2)

Genotype Zone 1	Genotype Zone 2	Mean	Lower 95% CL	Upper 95% CL	Group
Cur3	RMX180	263.9	211.4	329.5	a

Model summary

```
Linear mixed model fit by REML. t-tests use Satterthwaite's method ['lmerModLmerTest']
Formula: Movement_frequency_velocity_0_025_Zone_2 ~ 1 + (1 | Genotype_Zone_1:Plant_Zone_1) +      (1 |
Genotype_Zone_2:Plant_Zone_2)
   Data: data

REML criterion at convergence: 72.2

Scaled residuals:
    Min      1Q  Median      3Q     Max
-2.7447 -0.6024  0.2925  0.7026  1.7364

Random effects:
 Groups                       Name        Variance Std.Dev.
 Genotype_Zone_1:Plant_Zone_1 (Intercept) 0.000000 0.00000
 Genotype_Zone_2:Plant_Zone_2 (Intercept) 0.004163 0.06452
 Residual                                 0.352148 0.59342
Number of obs: 39, groups:  Genotype_Zone_1:Plant_Zone_1, 10; Genotype_Zone_2:Plant_Zone_2, 10

Fixed effects:
            Estimate Std. Error     df t value Pr(>|t|)
(Intercept)   5.5756     0.0972 8.4512   57.36 2.99e-12 ***
---
Signif. codes:  0 '***' 0.001 '**' 0.01 '*' 0.05 '.' 0.1 ' ' 1
```

Model residuals

Statistic	Value
Sample skewness	-0.7276
Sample excess kurtosis	0.3187
Passed Shapiro Wilk normality test	Yes (p-value = 0.08693 > 0.05)

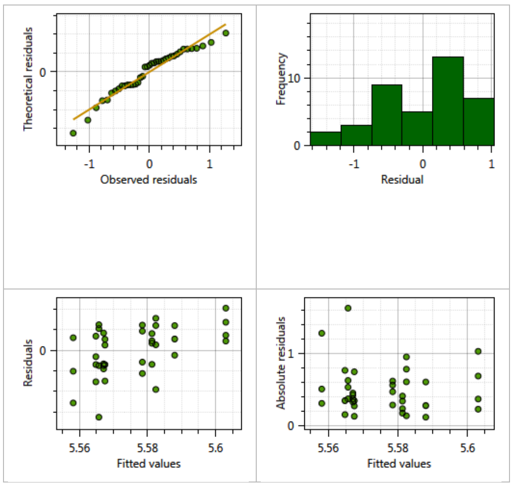

Analysis movement frequency (0.025 <= velocity < 0.075 - Zone 2)

**Analysis model**	Linear mixed model fit by REML: Movement_frequency_0_025_velocity_0_075_Zone_2 ~ 1 + (1|Genotype_Zone_1:Plant_Zone_1) + (1|Genotype_Zone_2:Plant_Zone_2)
**Transformation**	Natural logarithm

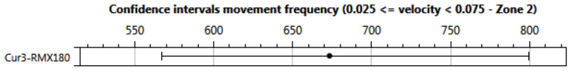

Confidence intervals movement frequency (0.025 <= velocity < 0.075 - Zone 2)

Genotype Zone 1	Genotype Zone 2	Mean	Lower 95% CL	Upper 95% CL	Group
Cur3	RMX180	673.3	566.9	799.7	a

Model summary

```
Linear mixed model fit by REML. t-tests use Satterthwaite's method ['lmerModLmerTest']
Formula: Movement_frequency_0_025_velocity_0_075_Zone_2 ~ 1 + (1 | Genotype_Zone_1:Plant_Zone_1) +
(1 | Genotype_Zone_2:Plant_Zone_2)
   Data: data

REML criterion at convergence: 63.4

Scaled residuals:
     Min       1Q   Median       3Q      Max
-2.64900 -0.76023  0.04744  0.82343  1.83366

Random effects:
 Groups                       Name        Variance  Std.Dev.
 Genotype_Zone_1:Plant_Zone_1 (Intercept) 3.063e-18 1.750e-09
 Genotype_Zone_2:Plant_Zone_2 (Intercept) 1.636e-15 4.045e-08
 Residual                                 2.817e-01 5.308e-01
Number of obs: 39, groups:  Genotype_Zone_1:Plant_Zone_1, 10; Genotype_Zone_2:Plant_Zone_2, 10

Fixed effects:
            Estimate Std. Error       df t value Pr(>|t|)
(Intercept)  6.51218    0.08499 38.00000   76.62   <2e-16 ***
---
Signif. codes:  0 '***' 0.001 '**' 0.01 '*' 0.05 '.' 0.1 ' ' 1
```

Model residuals

Statistic	Value
Sample skewness	-0.3959
Sample excess kurtosis	-0.1856
Passed Shapiro Wilk normality test	Yes (p-value = 0.3295 > 0.05)

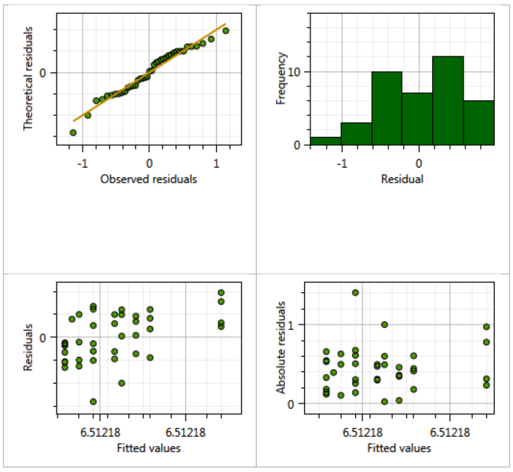

Analysis movement frequency (velocity >= 0.075 - Zone 2)

**Analysis model**	Linear mixed model fit by REML: Movement_frequency_velocity_0_075_Zone_2 ~ 1 + (1|Genotype_Zone_1:Plant_Zone_1) + (1|Genotype_Zone_2:Plant_Zone_2)
**Transformation**	Natural logarithm

Confidence intervals movement frequency (velocity >= 0.075 - Zone 2)

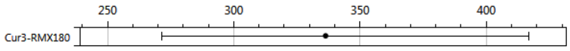

Confidence intervals movement frequency (velocity >= 0.075 - Zone 2)

Genotype Zone 1	Genotype Zone 2	Mean	Lower 95% CL	Upper 95% CL	Group
Cur3	RMX180	336.4	271.3	416.9	a

Model summary

```
Linear mixed model fit by REML. t-tests use Satterthwaite's method ['lmerModLmerTest']
Formula: Movement_frequency_velocity_0_075_Zone_2 ~ 1 + (1 | Genotype_Zone_1:Plant_Zone_1) +      (1 |
Genotype_Zone_2:Plant_Zone_2)
   Data: data

REML criterion at convergence: 80.2

Scaled residuals: 
    Min      1Q  Median      3Q     Max 
-3.6947 -0.1254  0.2652  0.5293  1.0817 

Random effects:
 Groups                       Name        Variance Std.Dev.
 Genotype_Zone_1:Plant_Zone_1 (Intercept) 0.000    0.0000  
 Genotype_Zone_2:Plant_Zone_2 (Intercept) 0.000    0.0000  
 Residual                                 0.439    0.6626  
Number of obs: 39, groups:  Genotype_Zone_1:Plant_Zone_1, 10; Genotype_Zone_2:Plant_Zone_2, 10

Fixed effects:
            Estimate Std. Error      df t value Pr(>|t|)    
(Intercept)   5.8182     0.1061 38.0000   54.84   <2e-16 ***
---
Signif. codes:  0 '***' 0.001 '**' 0.01 '*' 0.05 '.' 0.1 ' ' 1
```

Model residuals

Statistic	Value
Sample skewness	-2.48
Sample excess kurtosis	6.739
Passed Shapiro Wilk normality test	No (p-value = 2.085E-07 < 0.05)

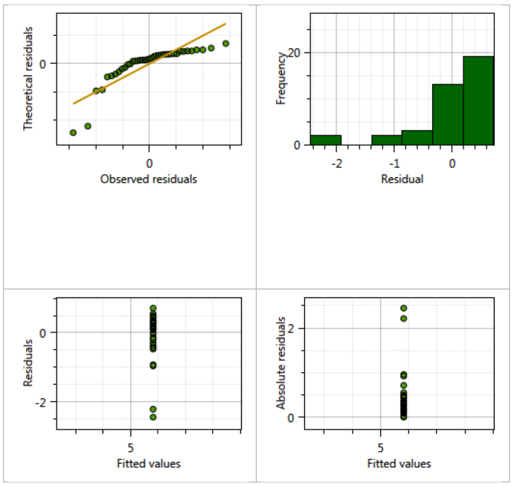

Analysis movement frequency velocity < 0.025 (diff. Zone 1 - Zone 2)

**Analysis model**	Generalized linear mixed model with dispersion factor, formula=cbind(Movement_frequency_velocity_0_025_Zone_1,Movement_frequency_velocity_0_025_Zone_2) ~ 1 + (1|Genotype_Zone_1:Plant_Zone_1) + (1|Genotype_Zone_2:Plant_Zone_2)
**Transformation**	Logit

Confidence intervals movement frequency velocity < 0.025 (diff. Zone 1 - Zone 2)

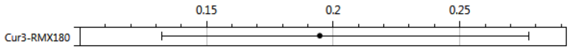

Confidence intervals movement frequency velocity < 0.025 (diff. Zone 1 - Zone 2)

Genotype Zone 1	Genotype Zone 2	Mean	Lower 95% CL	Upper 95% CL	Group
Cur3	RMX180	0.1947	0.1322	0.2773	a

Model summary

```
Linear mixed model fit by REML. t-tests use Satterthwaite's method ['lmerModLmerTest']
Formula: ziFormula
   Data: data
```

```
Weights: wi

REML criterion at convergence: 122.4

Scaled residuals:
     Min       1Q   Median       3Q      Max 
-1.27934 -0.67344 -0.03325  0.56201  2.30716 

Random effects:
 Groups                      Name        Variance Std.Dev.
 Genotype_Zone_1:Plant_Zone_1 (Intercept)  0.000   0.0000 
 Genotype_Zone_2:Plant_Zone_2 (Intercept)  0.125   0.3536 
 Residual                                 67.620   8.2231 
Number of obs: 39, groups:  Genotype_Zone_1:Plant_Zone_1, 10; Genotype_Zone_2:Plant_Zone_2, 10

Fixed effects:
            Estimate Std. Error      df t value Pr(>|t|)    
(Intercept)  -1.4198     0.2056  9.3900  -6.906 5.69e-05 ***
---
Signif. codes:  0 '***' 0.001 '**' 0.01 '*' 0.05 '.' 0.1 ' ' 1

Dispersion: 8.223
```

Model residuals

Statistic	Value
Sample skewness	0.7285
Sample excess kurtosis	-0.09359
Passed Shapiro Wilk normality test	No (p-value = 0.04083 < 0.05)

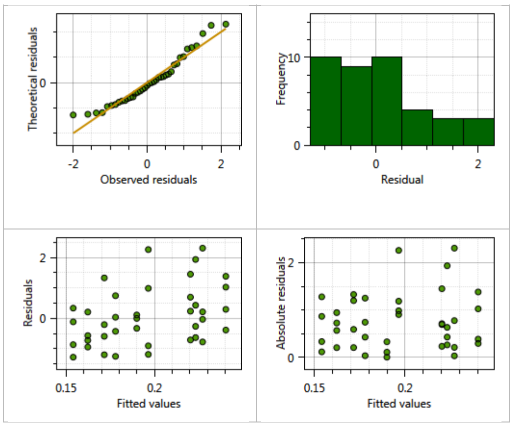

Analysis movement frequency 0.025 <= velocity < 0.075 (diff. Zone 1 - Zone 2)

**Analysis model**	Generalized linear mixed model with dispersion factor, formula=cbind(Movement_frequency_0_025_velocity_0_075_Zone_1,Movement_frequency_0_025_velocity_0_075_Zone_2) ~ 1 + (1|Genotype_Zone_1:Plant_Zone_1) + (1|Genotype_Zone_2:Plant_Zone_2)
**Transformation**	Logit

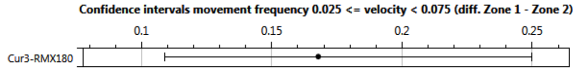

Confidence intervals movement frequency 0.025 <= velocity < 0.075 (diff. Zone 1 - Zone 2)

Genotype Zone 1	Genotype Zone 2	Mean	Lower 95% CL	Upper 95% CL	Group
Cur3	RMX180	0.1678	0.1088	0.2499	a

Model summary

```
Linear mixed model fit by REML. t-tests use Satterthwaite's method ['lmerModLmerTest']
Formula: ziFormula
   Data: data
Weights: wi

REML criterion at convergence: 117.9

Scaled residuals:
     Min       1Q   Median       3Q      Max 
-1.33509 -0.65226  0.03052  0.65600  2.25225 

Random effects:
 Groups                      Name        Variance  Std.Dev.
 Genotype_Zone_1:Plant_Zone_1 (Intercept)   0.07596  0.2756 
 Genotype_Zone_2:Plant_Zone_2 (Intercept)   0.15497  0.3937 
 Residual                                 118.65865 10.8931 
Number of obs: 39, groups:  Genotype_Zone_1:Plant_Zone_1, 10; Genotype_Zone_2:Plant_Zone_2, 10

Fixed effects:
            Estimate Std. Error      df t value Pr(>|t|)    
(Intercept)  -1.6010     0.2183  8.1228  -7.334 7.51e-05 ***
---
Signif. codes:  0 '***' 0.001 '**' 0.01 '*' 0.05 '.' 0.1 ' ' 1

Dispersion: 10.89
```

Model residuals

Statistic	Value
Sample skewness	0.4601
Sample excess kurtosis	-0.4624
Passed Shapiro Wilk normality test	Yes (p-value = 0.2195 > 0.05)

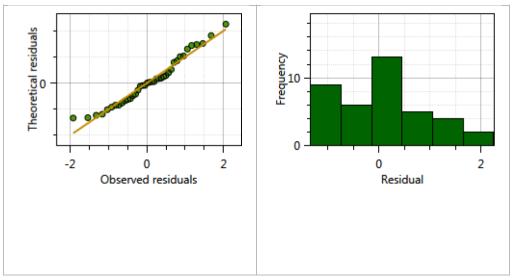

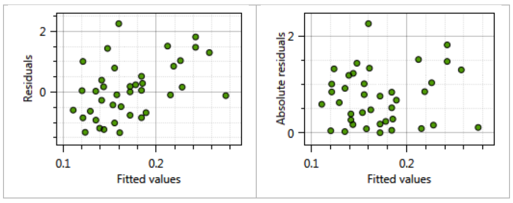

Analysis movement frequency velocity >= 0.075 (diff. Zone 1 - Zone 2)

**Analysis model**	Generalized linear mixed model with dispersion factor, formula=cbind(Movement_frequency_velocity_0_075_Zone_1,Movement_frequency_velocity_0_075_Zone_2) ~ 1 + (1|Genotype_Zone_1:Plant_Zone_1) + (1|Genotype_Zone_2:Plant_Zone_2)
**Transformation**	Logit

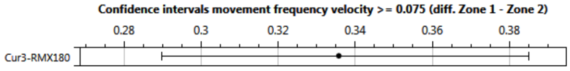

Confidence intervals movement frequency velocity >= 0.075 (diff. Zone 1 - Zone 2)

Genotype Zone 1	Genotype Zone 2	Mean	Lower 95% CL	Upper 95% CL	Group
Cur3	RMX180	0.3357	0.2897	0.3851	a

Model summary

```
Linear mixed model fit by REML. t-tests use Satterthwaite's method ['lmerModLmerTest']
Formula: ziFormula
   Data: data
Weights: wi

REML criterion at convergence: 61.8

Scaled residuals:
     Min       1Q   Median       3Q      Max
-1.92929 -0.77525 -0.07534  0.44550  2.21891

Random effects:
 Groups                      Name        Variance Std.Dev.
 Genotype_Zone_1:Plant_Zone_1 (Intercept)  0.03948 0.1987
 Genotype_Zone_2:Plant_Zone_2 (Intercept)  0.00000 0.0000
 Residual                                 25.93783 5.0929
Number of obs: 39, groups:  Genotype_Zone_1:Plant_Zone_1, 10; Genotype_Zone_2:Plant_Zone_2, 10

Fixed effects:
            Estimate Std. Error       df t value Pr(>|t|)
(Intercept) -0.68238    0.09605  9.84397  -7.105 3.56e-05 ***
---
Signif. codes:  0 '***' 0.001 '**' 0.01 '*' 0.05 '.' 0.1 ' ' 1

Dispersion: 5.093
```

Model residuals

Statistic	Value
Sample skewness	0.5562
Sample excess kurtosis	-0.07423
Passed Shapiro Wilk normality test	Yes (p-value = 0.2294 > 0.05)

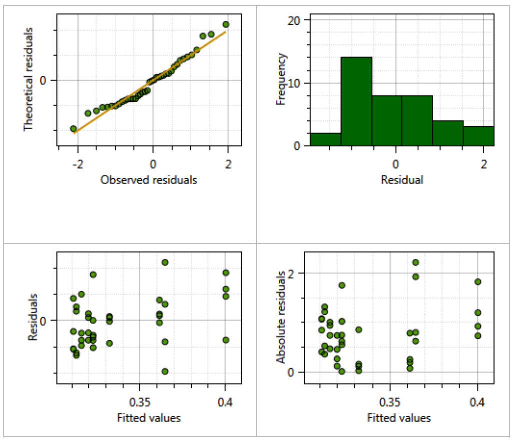

Ratio detection to total trial duration per zone

Selected zones	Zone 1, Zone 2
Data transformation	Natural logarithm
Analysis	Zone difference analysis

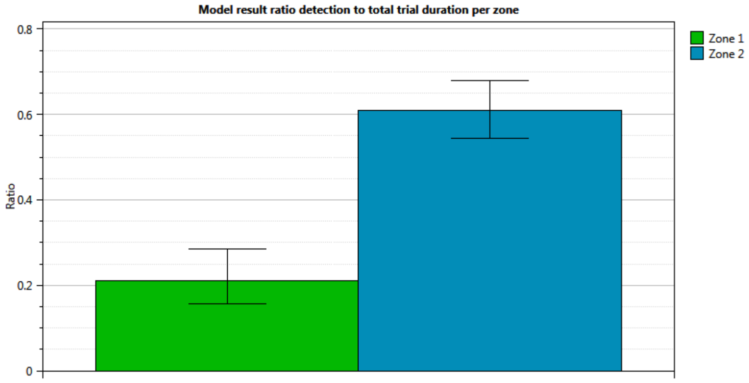

Results difference tests Zone 1 - Zone 2: p values and 95% confidence intervals of the difference on the transformed scale for each statistic.

Behaviour statistic	Cur3-RMX180
Ratio detection to total trial duration (diff. Zone 1 - Zone 2)	p=0.00172**<br>[-1.31, -0.541]

The model predictions and 95% confidence intervals for each statistic.

Statistic	Cur3-RMX180	Remark
Ratio detection to total trial duration (Zone 1)	0.211<br>[0.156, 0.285]	CR
Ratio detection to total trial duration (Zone 2)	0.608<br>[0.544, 0.68]	

CR = Check residuals

Data summary

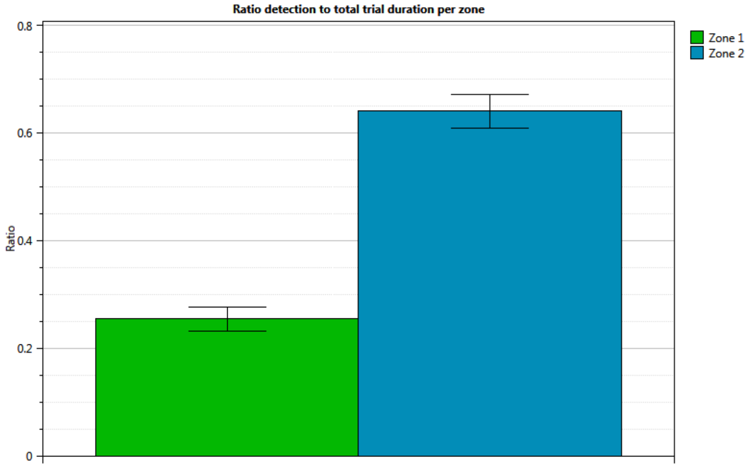

Genotype Zone 1	Genotype Zone 2	Genotype Zone 3	Mean Zone 1	StdErr Zone 1	Mean Zone 2	StdErr Zone 2
Cur3	RMX180	Neutral	0.25	0.02	0.64	0.03

Analysis ratio detection to total trial duration (Zone 1)

**Analysis model**	Linear mixed model fit by REML: Ratio_detection_to_total_trial_duration_Zone_1 ~ 1 + (1|Genotype_Zone_1:Plant_Zone_1) + (1|Genotype_Zone_2:Plant_Zone_2)
**Transformation**	Natural logarithm

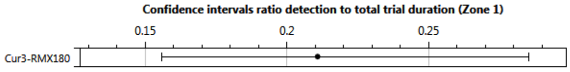

Confidence intervals ratio detection to total trial duration (Zone 1)

Genotype Zone 1	Genotype Zone 2	Mean	Lower 95% CL	Upper 95% CL	Group
Cur3	RMX180	0.2108	0.1557	0.2854	a

Model summary

```
Linear mixed model fit by REML. t-tests use Satterthwaite's method ['lmerModLmerTest']
Formula: Ratio_detection_to_total_trial_duration_Zone_1 ~ 1 + (1 | Genotype_Zone_1:Plant_Zone_1) +
(1 | Genotype_Zone_2:Plant_Zone_2)
   Data: data

REML criterion at convergence: 92

Scaled residuals:
    Min      1Q  Median      3Q     Max
-3.7942 -0.2809  0.2333  0.6354  1.2494

Random effects:
 Groups                       Name        Variance Std.Dev.
 Genotype_Zone_1:Plant_Zone_1 (Intercept) 0.00000  0.0000
 Genotype_Zone_2:Plant_Zone_2 (Intercept) 0.01829  0.1352
 Residual                                 0.62139  0.7883
Number of obs: 38, groups:  Genotype_Zone_1:Plant_Zone_1, 10; Genotype_Zone_2:Plant_Zone_2, 10

Fixed effects:
            Estimate Std. Error      df t value Pr(>|t|)
(Intercept)  -1.5569     0.1349  9.4157  -11.54 7.23e-07 ***
---
Signif. codes:  0 '***' 0.001 '**' 0.01 '*' 0.05 '.' 0.1 ' ' 1
```

Model residuals

Statistic	Value
Sample skewness	-1.877
Sample excess kurtosis	4.855
Passed Shapiro Wilk normality test	No (p-value = 8.164E-05 < 0.05)

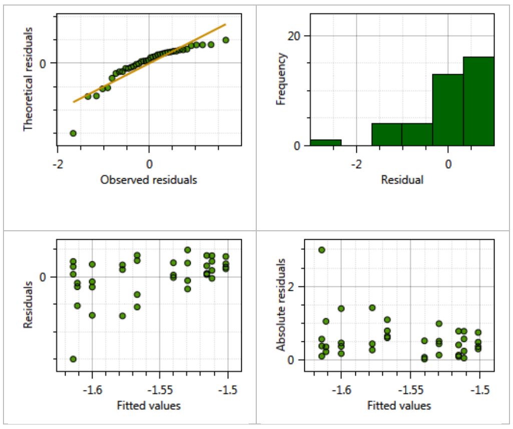

Analysis ratio detection to total trial duration (Zone 2)

**Analysis model**	Linear mixed model fit by REML: Ratio_detection_to_total_trial_duration_Zone_2 ~ 1 + (1|Genotype_Zone_1:Plant_Zone_1) + (1|Genotype_Zone_2:Plant_Zone_2)
**Transformation**	Natural logarithm

Confidence intervals ratio detection to total trial duration (Zone 2)

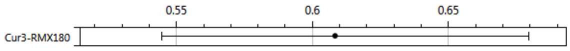

Confidence intervals ratio detection to total trial duration (Zone 2)

Genotype Zone 1	Genotype Zone 2	Mean	Lower 95% CL	Upper 95% CL	Group
Cur3	RMX180	0.6083	0.5445	0.6796	a

Model summary

```
Linear mixed model fit by REML. t-tests use Satterthwaite's method ['lmerModLmerTest']
Formula: Ratio_detection_to_total_trial_duration_Zone_2 ~ 1 + (1 | Genotype_Zone_1:Plant_Zone_1) +
(1 | Genotype_Zone_2:Plant_Zone_2)
   Data: data

REML criterion at convergence: 29.9

Scaled residuals:
    Min      1Q  Median      3Q     Max
-3.1105 -0.6627  0.1485  0.6891  1.4537

Random effects:
 Groups                       Name        Variance  Std.Dev.
 Genotype_Zone_1:Plant_Zone_1 (Intercept) 5.168e-16 2.273e-08
 Genotype_Zone_2:Plant_Zone_2 (Intercept) 2.150e-17 4.637e-09
 Residual                                 1.169e-01 3.419e-01
Number of obs: 39, groups:  Genotype_Zone_1:Plant_Zone_1, 10; Genotype_Zone_2:Plant_Zone_2, 10

Fixed effects:
            Estimate Std. Error       df t value Pr(>|t|)
(Intercept) -0.49706    0.05475 38.00000  -9.078 4.67e-11 ***
---
Signif. codes:  0 '***' 0.001 '**' 0.01 '*' 0.05 '.' 0.1 ' ' 1
```

Model residuals

Statistic	Value
Sample skewness	-0.8693
Sample excess kurtosis	0.95
Passed Shapiro Wilk normality test	Yes (p-value = 0.05879 > 0.05)

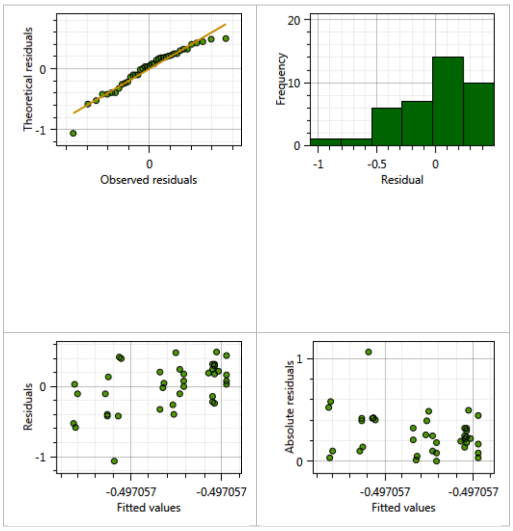

Analysis ratio detection to total trial duration (diff. Zone 1 - Zone 2)

**Analysis model**	Generalized linear mixed model with dispersion factor, formula=cbind(Ratio_detection_to_total_trial_duration_Zone_1,Ratio_detection_to_total_trial_duration_Zone_2) ~ 1 + (1|Genotype_Zone_1:Plant_Zone_1) + (1|Genotype_Zone_2:Plant_Zone_2)
**Transformation**	Logit

Confidence intervals ratio detection to total trial duration (diff. Zone 1 - Zone 2)

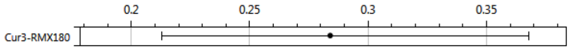

Confidence intervals ratio detection to total trial duration (diff. Zone 1 - Zone 2)

Genotype Zone 1	Genotype Zone 2	Mean	Lower 95% CL	Upper 95% CL	Group
Cur3	RMX180	0.284	0.2127	0.368	a

Model summary

```
Linear mixed model fit by REML. t-tests use Satterthwaite's method ['lmerModLmerTest']
Formula: ziFormula
   Data: data
Weights: wi

REML criterion at convergence: 99.5

Scaled residuals:
    Min      1Q  Median      3Q     Max
-1.7183 -0.6320  0.0032  0.7057  2.0312

Random effects:
 Groups                       Name        Variance Std.Dev.
 Genotype_Zone_1:Plant_Zone_1 (Intercept) 0.009055 0.09516
 Genotype_Zone_2:Plant_Zone_2 (Intercept) 0.032352 0.17987
 Residual                                 0.125427 0.35416
Number of obs: 39, groups:  Genotype_Zone_1:Plant_Zone_1, 10; Genotype_Zone_2:Plant_Zone_2, 10

Fixed effects:
            Estimate Std. Error      df t value Pr(>|t|)
(Intercept)  -0.9248     0.1478  4.8410  -6.256  0.00172 **
---
Signif. codes:  0 '***' 0.001 '**' 0.01 '*' 0.05 '.' 0.1 ' ' 1

Dispersion: 0.3542
```

Model residuals

Statistic	Value
Sample skewness	0.1266
Sample excess kurtosis	-0.6308
Passed Shapiro Wilk normality test	Yes (p-value = 0.7825 > 0.05)

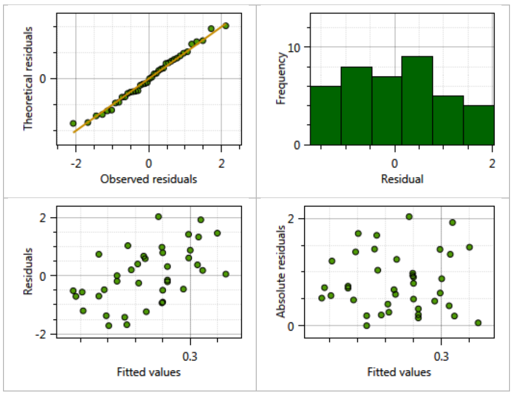

Ratio detection to total trial duration per zone per hour

Selected hours	0, 1, 2, 3, 4, 5, 6, 7
Selected zones	Zone 1, Zone 2
Data transformation	Natural logarithm
Analysis	Zone difference analysis

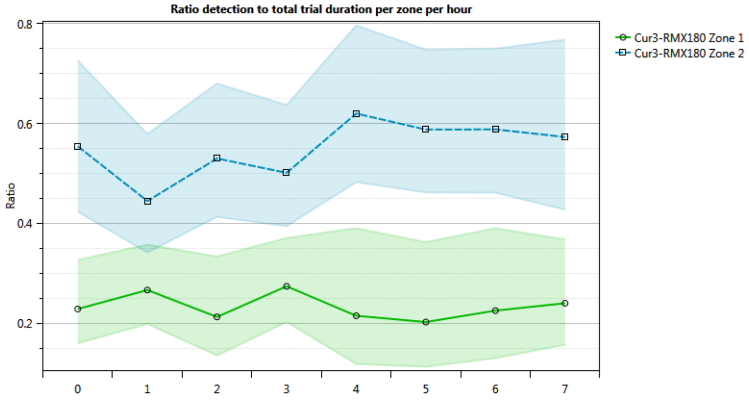

Results difference tests Zone 1 - Zone 2: p values and 95% confidence intervals of the difference on the transformed scale for each statistic.

Behaviour statistic	Cur3-RMX180	Remark
Ratio detection to total trial duration H0 (diff. Zone 1 - Zone 2)	p=0.000125***<br>[-1.28, -0.457]	CR
Ratio detection to total trial duration H1 (diff. Zone 1 - Zone 2)	p=0.0181*<br>[-0.864, -0.0857]	
Ratio detection to total trial duration H2 (diff. Zone 1 - Zone 2)	p=0.0191*<br>[-1.32, -0.152]	CR
Ratio detection to total trial duration H3 (diff. Zone 1 - Zone 2)	p=0.013*<br>[-1.31, -0.202]	CR
Ratio detection to total trial duration H4 (diff. Zone 1 - Zone 2)	p=0.02*<br>[-1.62, -0.2]	CR
Ratio detection to total trial duration H5 (diff. Zone 1 - Zone 2)	p=3.09E-05****<br>[-1.62, -0.648]	CR
Ratio detection to total trial duration H6 (diff. Zone 1 - Zone 2)	p=3.4E-05****<br>[-1.66, -0.661]	CR
Ratio detection to total trial duration H7 (diff. Zone 1 - Zone 2)	p=0.00444**<br>[-1.86, -0.533]	CR

CR = Check residuals

The model predictions and 95% confidence intervals for each statistic.

Statistic	Cur3-RMX180	Remark
Ratio detection to total trial duration (H0 - Zone 1)	0.229<br>[0.16, 0.327]	CR
Ratio detection to total trial duration (H0 - Zone 2)	0.554<br>[0.423, 0.726]	CR
Ratio detection to total trial duration (H1 - Zone 1)	0.267<br>[0.199, 0.358]	CR
Ratio detection to total trial duration (H1 - Zone 2)	0.445<br>[0.342, 0.579]	CR
Ratio detection to total trial duration (H2 - Zone 1)	0.213<br>[0.136, 0.334]	
Ratio detection to total trial duration (H2 - Zone 2)	0.53<br>[0.413, 0.68]	CR
Ratio detection to total trial duration (H3 - Zone 1)	0.274<br>[0.203, 0.371]	
Ratio detection to total trial duration (H3 - Zone 2)	0.501<br>[0.394, 0.637]	CR
Ratio detection to total trial duration (H4 - Zone 1)	0.215<br>[0.119, 0.391]	
Ratio detection to total trial duration (H4 - Zone 2)	0.62<br>[0.483, 0.796]	CR
Ratio detection to total trial duration (H5 - Zone 1)	0.203<br>[0.113, 0.363]	CR
Ratio detection to total trial duration (H5 - Zone 2)	0.588<br>[0.462, 0.748]	CR
Ratio detection to total trial duration (H6 - Zone 1)	0.226<br>[0.13, 0.391]	
Ratio detection to total trial duration (H6 - Zone 2)	0.588<br>[0.461, 0.75]	CR
Ratio detection to total trial duration (H7 - Zone 1)	0.24<br>[0.157, 0.368]	CR
Ratio detection to total trial duration (H7 - Zone 2)	0.573<br>[0.427, 0.768]	CR

CR = Check residuals

Data summary

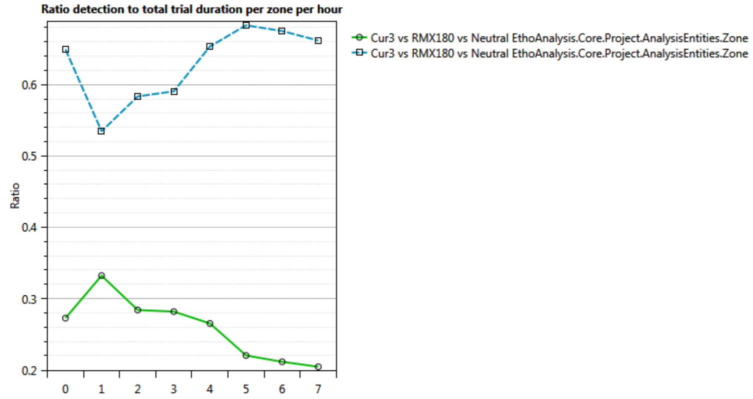

Genotype Zone 1	Genotype Zone 2	Genotype Zone 3	Mean H0 - Zone 1	StdErr H0 - Zone 1	Mean H0 - Zone 2	StdErr H0 - Zone 2	Mean H1 - Zone 1	StdErr H1 - Zone 1	Mean H1 - Zone 2	StdErr H1 - Zone 2	Mean H2 - Zone 1	StdErr H2 - Zone 1	Mean H2 - Zone 2	StdErr H2 - Zone 2	Mean H3 - Zone 1	StdErr H3 - Zone 1	Mean H3 - Zone 2	StdErr H3 - Zone 2	Mean H4 - Zone 1	StdErr H4 - Zone 1	Mean H4 - Zone 2	StdErr H4 - Zone 2	Mean H5 - Zone 1	StdErr H5 - Zone 1	Mean H5 - Zone 2	StdErr H5 - Zone 2	Mean H6 - Zone 1	StdErr H6 - Zone 1	Mean H6 - Zone 2	StdErr H6 - Zone 2
Cur3	RMX180	Neutral	0.27	0.04	0.65	0.04	0.33	0.04	0.53	0.04	0.28	0.04	0.58	0.05	0.28	0.04	0.59	0.05	0.27	0.05	0.65	0.05	0.22	0.04	0.68	0.05	0.21	0.04	0.68	0.05

Analysis ratio detection to total trial duration (H0 - Zone 1)

**Analysis model**	Linear mixed model fit by REML: Ratio_detection_to_total_trial_duration_H0_Zone_1 ~ 1 + (1|Genotype_Zone_1:Plant_Zone_1) + (1|Genotype_Zone_2:Plant_Zone_2)
**Transformation**	Natural logarithm

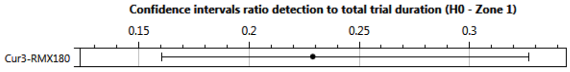

Confidence intervals ratio detection to total trial duration (H0 - Zone 1)

Genotype Zone 1	Genotype Zone 2	Mean	Lower 95% CL	Upper 95% CL	Group
Cur3	RMX180	0.2289	0.1602	0.327	a

Model summary

```
Linear mixed model fit by REML. t-tests use Satterthwaite's method ['lmerModLmerTest']
Formula: Ratio_detection_to_total_trial_duration_H0_Zone_1 ~ 1 + (1 |      Genotype_Zone_1:Plant_Zone_1)
+ (1 | Genotype_Zone_2:Plant_Zone_2)
   Data: data

REML criterion at convergence: 94.7

Scaled residuals:
    Min      1Q  Median      3Q     Max
-3.1115 -0.4201  0.2351  0.7360  1.3933

Random effects:
 Groups                       Name        Variance Std.Dev.
 Genotype_Zone_1:Plant_Zone_1 (Intercept) 0.000    0.000
 Genotype_Zone_2:Plant_Zone_2 (Intercept) 0.000    0.000
 Residual                                 1.012    1.006
Number of obs: 33, groups:  Genotype_Zone_1:Plant_Zone_1, 10; Genotype_Zone_2:Plant_Zone_2, 10

Fixed effects:
            Estimate Std. Error      df t value Pr(>|t|)
(Intercept)  -1.4745     0.1752 32.0000  -8.418 1.28e-09 ***
---
Signif. codes:  0 '***' 0.001 '**' 0.01 '*' 0.05 '.' 0.1 ' ' 1
```

Model residuals

Statistic	Value
Sample skewness	-1.381
Sample excess kurtosis	2.317
Passed Shapiro Wilk normality test	No (p-value = 0.004081 < 0.05)

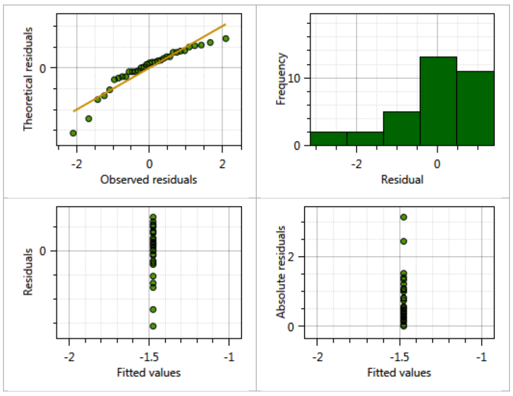

Analysis ratio detection to total trial duration (H0 - Zone 2)

**Analysis model**	Linear mixed model fit by REML: Ratio_detection_to_total_trial_duration_H0_Zone_2 ~ 1 + (1|Genotype_Zone_1:Plant_Zone_1) + (1|Genotype_Zone_2:Plant_Zone_2)
**Transformation**	Natural logarithm

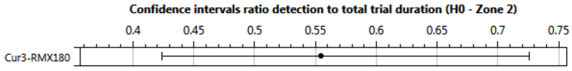

Confidence intervals ratio detection to total trial duration (H0 - Zone 2)

Genotype Zone 1	Genotype Zone 2	Mean	Lower 95% CL	Upper 95% CL	Group
Cur3	RMX180	0.5544	0.4234	0.726	a

Model summary

```
Linear mixed model fit by REML. t-tests use Satterthwaite's method ['lmerModLmerTest']
Formula: Ratio_detection_to_total_trial_duration_H0_Zone_2 ~ 1 + (1 |      Genotype_Zone_1:Plant_Zone_1)
+ (1 | Genotype_Zone_2:Plant_Zone_2)
   Data: data
```

```
REML criterion at convergence: 86.8

Scaled residuals:
    Min      1Q  Median      3Q     Max
-3.9904 -0.3409  0.2473  0.6244  0.9084

Random effects:
 Groups                      Name        Variance Std.Dev.
 Genotype_Zone_1:Plant_Zone_1 (Intercept) 0.00000  0.0000
 Genotype_Zone_2:Plant_Zone_2 (Intercept) 0.01107  0.1052
 Residual                                 0.51238  0.7158
Number of obs: 39, groups:  Genotype_Zone_1:Plant_Zone_1, 10; Genotype_Zone_2:Plant_Zone_2, 10

Fixed effects:
            Estimate Std. Error      df t value Pr(>|t|)
(Intercept)  -0.5898     0.1194  9.0852  -4.941 0.000779 ***
---
Signif. codes:  0 '***' 0.001 '**' 0.01 '*' 0.05 '.' 0.1 ' ' 1
```

Model residuals

Statistic	Value
Sample skewness	-2.55
Sample excess kurtosis	7.848
Passed Shapiro Wilk normality test	No (p-value = 3.993E-07 < 0.05)

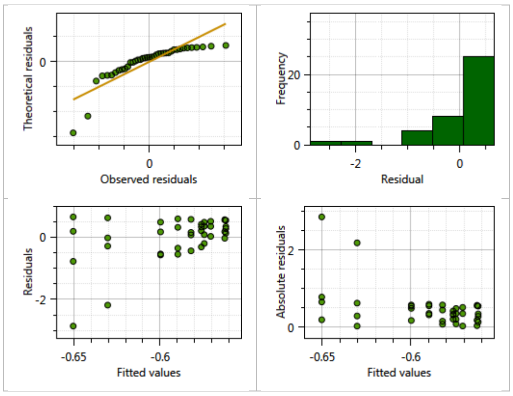

Analysis ratio detection to total trial duration (H1 - Zone 1)

**Analysis model**	Linear mixed model fit by REML: Ratio_detection_to_total_trial_duration_H1_Zone_1 ~ 1 + (1|Genotype_Zone_1:Plant_Zone_1) + (1|Genotype_Zone_2:Plant_Zone_2)
**Transformation**	Natural logarithm

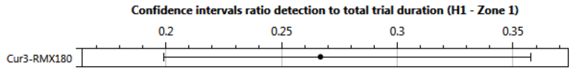

Confidence intervals ratio detection to total trial duration (H1 - Zone 1)

Genotype Zone 1	Genotype Zone 2	Mean	Lower 95% CL	Upper 95% CL	Group
Cur3	RMX180	0.2667	0.1989	0.3578	a

Model summary

```
Linear mixed model fit by REML. t-tests use Satterthwaite's method ['lmerModLmerTest']
Formula: Ratio_detection_to_total_trial_duration_H1_Zone_1 ~ 1 + (1 |      Genotype_Zone_1:Plant_Zone_1)
+ (1 | Genotype_Zone_2:Plant_Zone_2)
   Data: data

REML criterion at convergence: 96.6

Scaled residuals:
    Min      1Q  Median      3Q     Max
-3.7283 -0.3852  0.2067  0.6905  1.5004

Random effects:
 Groups                      Name        Variance Std.Dev.
 Genotype_Zone_1:Plant_Zone_1 (Intercept) 0.0000   0.0000
 Genotype_Zone_2:Plant_Zone_2 (Intercept) 0.0000   0.0000
 Residual                                 0.7757   0.8807
Number of obs: 37, groups:  Genotype_Zone_1:Plant_Zone_1, 10; Genotype_Zone_2:Plant_Zone_2, 10

Fixed effects:
            Estimate Std. Error      df t value Pr(>|t|)
(Intercept)  -1.3215     0.1448 36.0000  -9.127 6.73e-11 ***
---
Signif. codes:  0 '***' 0.001 '**' 0.01 '*' 0.05 '.' 0.1 ' ' 1
```

Model residuals

Statistic	Value
Sample skewness	-1.561
Sample excess kurtosis	4.163
Passed Shapiro Wilk normality test	No (p-value = 0.002193 < 0.05)

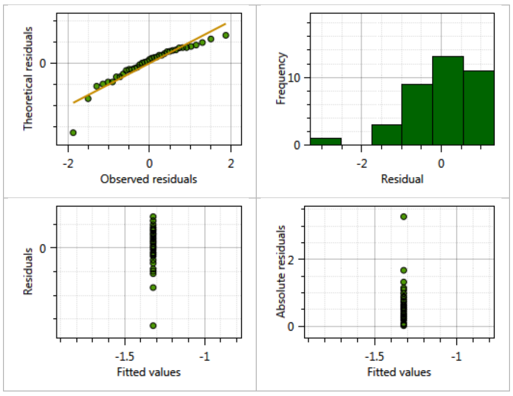

Analysis ratio detection to total trial duration (H1 - Zone 2)

**Analysis model**	Linear mixed model fit by REML: Ratio_detection_to_total_trial_duration_H1_Zone_2 ~ 1 + (1|Genotype_Zone_1:Plant_Zone_1) + (1|Genotype_Zone_2:Plant_Zone_2)
**Transformation**	Natural logarithm

Confidence intervals ratio detection to total trial duration (H1 - Zone 2)

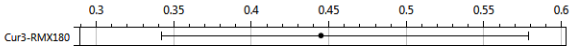

Confidence intervals ratio detection to total trial duration (H1 - Zone 2)

Genotype Zone 1	Genotype Zone 2	Mean	Lower 95% CL	Upper 95% CL	Group
Cur3	RMX180	0.4449	0.3418	0.579	a

Model summary

```
Linear mixed model fit by REML. t-tests use Satterthwaite's method ['lmerModLmerTest']
Formula: Ratio_detection_to_total_trial_duration_H1_Zone_2 ~ 1 + (1 |      Genotype_Zone_1:Plant_Zone_1)
+ (1 | Genotype_Zone_2:Plant_Zone_2)
   Data: data

REML criterion at convergence: 92.3

Scaled residuals:
    Min      1Q  Median      3Q     Max
-3.3629 -0.3870  0.3200  0.6346  1.0101

Random effects:
 Groups                       Name        Variance Std.Dev.
 Genotype_Zone_1:Plant_Zone_1 (Intercept) 0.000    0.0000
 Genotype_Zone_2:Plant_Zone_2 (Intercept) 0.000    0.0000
 Residual                                 0.643    0.8019
Number of obs: 38, groups:  Genotype_Zone_1:Plant_Zone_1, 10; Genotype_Zone_2:Plant_Zone_2, 10

Fixed effects:
            Estimate Std. Error      df t value Pr(>|t|)
(Intercept)  -0.8100     0.1301 37.0000  -6.227  3.1e-07 ***
---
Signif. codes:  0 '***' 0.001 '**' 0.01 '*' 0.05 '.' 0.1 ' ' 1
```

Model residuals

Statistic	Value
Sample skewness	-1.894
Sample excess kurtosis	3.974
Passed Shapiro Wilk normality test	No (p-value = 1.233E-05 < 0.05)

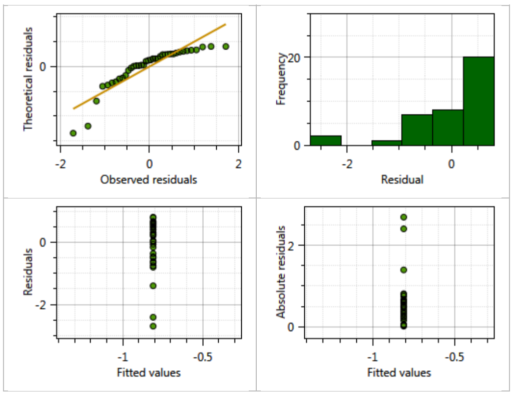

Analysis ratio detection to total trial duration (H2 - Zone 1)

**Analysis model**	Linear mixed model fit by REML: Ratio_detection_to_total_trial_duration_H2_Zone_1 ~ 1 + (1|Genotype_Zone_1:Plant_Zone_1) + (1|Genotype_Zone_2:Plant_Zone_2)
**Transformation**	Natural logarithm

Confidence intervals ratio detection to total trial duration (H2 - Zone 1)

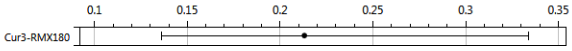

Confidence intervals ratio detection to total trial duration (H2 - Zone 1)

Genotype Zone 1	Genotype Zone 2	Mean	Lower 95% CL	Upper 95% CL	Group
Cur3	RMX180	0.2131	0.1359	0.334	a

Model summary

```
Linear mixed model fit by REML. t-tests use Satterthwaite's method ['lmerModLmerTest']
Formula: Ratio_detection_to_total_trial_duration_H2_Zone_1 ~ 1 + (1 |      Genotype_Zone_1:Plant_Zone_1)
+ (1 | Genotype_Zone_2:Plant_Zone_2)
   Data: data

REML criterion at convergence: 92.9

Scaled residuals:
     Min       1Q   Median       3Q      Max
-1.90355 -0.77323  0.07446  0.59519  1.68757

Random effects:
 Groups                       Name        Variance Std.Dev.
 Genotype_Zone_1:Plant_Zone_1 (Intercept) 0.2239   0.4732
 Genotype_Zone_2:Plant_Zone_2 (Intercept) 0.0000   0.0000
 Residual                                 0.5584   0.7473
Number of obs: 37, groups:  Genotype_Zone_1:Plant_Zone_1, 10; Genotype_Zone_2:Plant_Zone_2, 10

Fixed effects:
            Estimate Std. Error      df t value Pr(>|t|)
(Intercept)  -1.5461     0.1945  7.8868  -7.949 4.95e-05 ***
---
Signif. codes:  0 '***' 0.001 '**' 0.01 '*' 0.05 '.' 0.1 ' ' 1
```

Model residuals

Statistic	Value
Sample skewness	0.03008
Sample excess kurtosis	-0.8086
Passed Shapiro Wilk normality test	Yes (p-value = 0.5394 > 0.05)

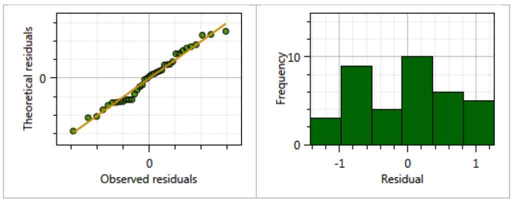

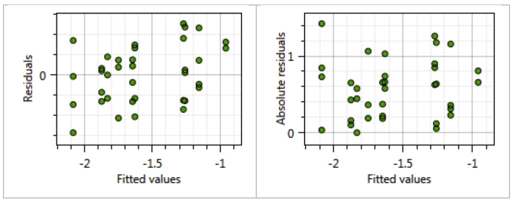

Analysis ratio detection to total trial duration (H2 - Zone 2)

**Analysis model**	Linear mixed model fit by REML: Ratio_detection_to_total_trial_duration_H2_Zone_2 ~ 1 + (1|Genotype_Zone_1:Plant_Zone_1) + (1|Genotype_Zone_2:Plant_Zone_2)
**Transformation**	Natural logarithm

Confidence intervals ratio detection to total trial duration (H2 - Zone 2)

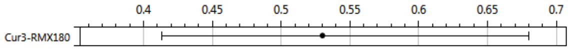

Confidence intervals ratio detection to total trial duration (H2 - Zone 2)

Genotype Zone 1	Genotype Zone 2	Mean	Lower 95% CL	Upper 95% CL	Group
Cur3	RMX180	0.53	0.413	0.6802	a

Model summary

```
Linear mixed model fit by REML. t-tests use Satterthwaite's method ['lmerModLmerTest']
Formula: Ratio_detection_to_total_trial_duration_H2_Zone_2 ~ 1 + (1 |      Genotype_Zone_1:Plant_Zone_1)
+ (1 | Genotype_Zone_2:Plant_Zone_2)
   Data: data

REML criterion at convergence: 74.2

Scaled residuals:
    Min      1Q  Median      3Q     Max
-2.9198 -0.1466  0.3677  0.6390  1.0315

Random effects:
 Groups                       Name        Variance Std.Dev.
 Genotype_Zone_1:Plant_Zone_1 (Intercept) 0.004643 0.06814
 Genotype_Zone_2:Plant_Zone_2 (Intercept) 0.000000 0.00000
 Residual                                 0.412281 0.64209
Number of obs: 37, groups:  Genotype_Zone_1:Plant_Zone_1, 10; Genotype_Zone_2:Plant_Zone_2, 10

Fixed effects:
            Estimate Std. Error      df t value Pr(>|t|)
(Intercept)  -0.6349     0.1078  7.8205  -5.891 0.000398 ***
---
Signif. codes:  0 '***' 0.001 '**' 0.01 '*' 0.05 '.' 0.1 ' ' 1
```

Model residuals

Statistic	Value
Sample skewness	-1.587
Sample excess kurtosis	1.977
Passed Shapiro Wilk normality test	No (p-value = 2.174E-05 < 0.05)

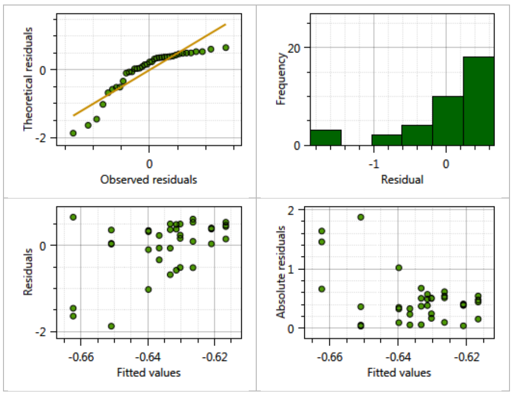

Analysis ratio detection to total trial duration (H3 - Zone 1)

**Analysis model**	Linear mixed model fit by REML: Ratio_detection_to_total_trial_duration_H3_Zone_1 ~ 1 + (1|Genotype_Zone_1:Plant_Zone_1) + (1|Genotype_Zone_2:Plant_Zone_2)
**Transformation**	Natural logarithm

Confidence intervals ratio detection to total trial duration (H3 - Zone 1)

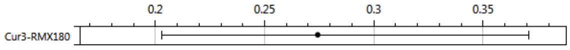

Confidence intervals ratio detection to total trial duration (H3 - Zone 1)

Genotype Zone 1	Genotype Zone 2	Mean	Lower 95% CL	Upper 95% CL	Group
Cur3	RMX180	0.2743	0.2028	0.371	a

Model summary

```
Linear mixed model fit by REML. t-tests use Satterthwaite's method ['lmerModLmerTest']
Formula: Ratio_detection_to_total_trial_duration_H3_Zone_1 ~ 1 + (1 |      Genotype_Zone_1:Plant_Zone_1)
+ (1 | Genotype_Zone_2:Plant_Zone_2)
   Data: data

REML criterion at convergence: 71.3

Scaled residuals:
    Min      1Q  Median      3Q     Max
-2.6093 -0.6155  0.1061  0.6392  1.5419

Random effects:
 Groups                       Name        Variance  Std.Dev.
 Genotype_Zone_1:Plant_Zone_1 (Intercept) 4.599e-18 2.145e-09
 Genotype_Zone_2:Plant_Zone_2 (Intercept) 1.341e-02 1.158e-01
 Residual                                 5.106e-01 7.146e-01
Number of obs: 32, groups:  Genotype_Zone_1:Plant_Zone_1, 10; Genotype_Zone_2:Plant_Zone_2, 10

Fixed effects:
            Estimate Std. Error      df t value Pr(>|t|)
(Intercept)  -1.2936     0.1318  8.2898  -9.816 7.58e-06 ***
---
Signif. codes:  0 '***' 0.001 '**' 0.01 '*' 0.05 '.' 0.1 ' ' 1
```

Model residuals

Statistic	Value
Sample skewness	-0.5762
Sample excess kurtosis	0.2564
Passed Shapiro Wilk normality test	Yes (p-value = 0.3726 > 0.05)

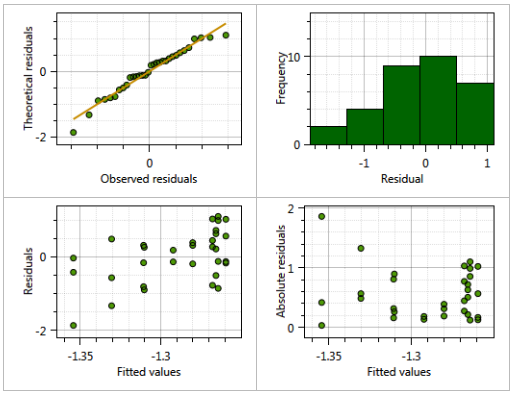

Analysis ratio detection to total trial duration (H3 - Zone 2)

**Analysis model**	Linear mixed model fit by REML: Ratio_detection_to_total_trial_duration_H3_Zone_2 ~ 1 + (1|Genotype_Zone_1:Plant_Zone_1) + (1|Genotype_Zone_2:Plant_Zone_2)
**Transformation**	Natural logarithm

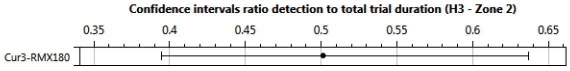

Confidence intervals ratio detection to total trial duration (H3 - Zone 2)

Genotype Zone 1	Genotype Zone 2	Mean	Lower 95% CL	Upper 95% CL	Group
Cur3	RMX180	0.5012	0.3944	0.637	a

Model summary

```
Linear mixed model fit by REML. t-tests use Satterthwaite's method ['lmerModLmerTest']
Formula: Ratio_detection_to_total_trial_duration_H3_Zone_2 ~ 1 + (1 |      Genotype_Zone_1:Plant_Zone_1)
+ (1 | Genotype_Zone_2:Plant_Zone_2)
   Data: data

REML criterion at convergence: 78.6

Scaled residuals:
    Min      1Q  Median      3Q     Max
-2.4777 -0.4240  0.1801  0.7568  1.0724

Random effects:
 Groups                       Name        Variance Std.Dev.
 Genotype_Zone_1:Plant_Zone_1 (Intercept) 0.000000 0.00000
 Genotype_Zone_2:Plant_Zone_2 (Intercept) 0.004121 0.06419
 Residual                                 0.417374 0.64604
Number of obs: 39, groups:  Genotype_Zone_1:Plant_Zone_1, 10; Genotype_Zone_2:Plant_Zone_2, 10

Fixed effects:
            Estimate Std. Error      df t value Pr(>|t|)
(Intercept)  -0.6907     0.1054  8.7070  -6.551 0.000122 ***
---
Signif. codes:  0 '***' 0.001 '**' 0.01 '*' 0.05 '.' 0.1 ' ' 1
```

Model residuals

Statistic	Value
Sample skewness	-1.074
Sample excess kurtosis	0.5468
Passed Shapiro Wilk normality test	No (p-value = 0.0007359 < 0.05)

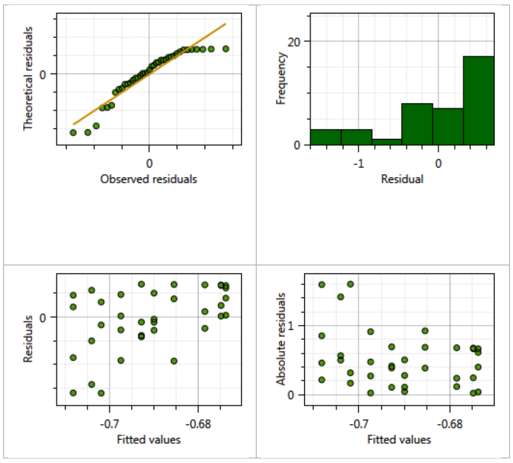

Analysis ratio detection to total trial duration (H4 - Zone 1)

**Analysis model**	Linear mixed model fit by REML: Ratio_detection_to_total_trial_duration_H4_Zone_1 ~ 1 + (1|Genotype_Zone_1:Plant_Zone_1) + (1|Genotype_Zone_2:Plant_Zone_2)
**Transformation**	Natural logarithm

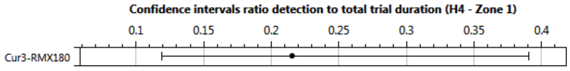

Confidence intervals ratio detection to total trial duration (H4 - Zone 1)

Genotype Zone 1	Genotype Zone 2	Mean	Lower 95% CL	Upper 95% CL	Group
Cur3	RMX180	0.2154	0.1187	0.3906	a

Model summary

```
Linear mixed model fit by REML. t-tests use Satterthwaite's method ['lmerModLmerTest']
Formula: Ratio_detection_to_total_trial_duration_H4_Zone_1 ~ 1 + (1 |      Genotype_Zone_1:Plant_Zone_1)
+ (1 | Genotype_Zone_2:Plant_Zone_2)
   Data: data

REML criterion at convergence: 88.4
```

```
Scaled residuals:
     Min       1Q   Median       3Q      Max 
-1.53383 -0.45489 -0.06424  0.59276  1.67802 

Random effects:
 Groups                     Name        Variance Std.Dev.
 Genotype_Zone_1:Plant_Zone_1 (Intercept) 0.0000   0.0000  
 Genotype_Zone_2:Plant_Zone_2 (Intercept) 0.2857   0.5345  
 Residual                               0.8921   0.9445  
Number of obs: 30, groups:  Genotype_Zone_1:Plant_Zone_1, 10; Genotype_Zone_2:Plant_Zone_2, 10

Fixed effects:
            Estimate Std. Error      df t value Pr(>|t|)    
(Intercept)  -1.5355     0.2436  6.0203  -6.304 0.000733 ***
---
Signif. codes:  0 '***' 0.001 '**' 0.01 '*' 0.05 '.' 0.1 ' ' 1
```

Model residuals

Statistic	Value
Sample skewness	-0.1622
Sample excess kurtosis	-0.9099
Passed Shapiro Wilk normality test	Yes (p-value = 0.1648 > 0.05)

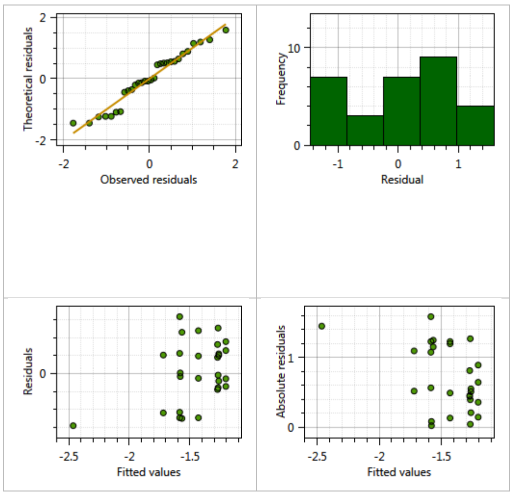

Analysis ratio detection to total trial duration (H4 - Zone 2)

**Analysis model**	Linear mixed model fit by REML: Ratio_detection_to_total_trial_duration_H4_Zone_2 ~ 1 + (1|Genotype_Zone_1:Plant_Zone_1) + (1|Genotype_Zone_2:Plant_Zone_2)
**Transformation**	Natural logarithm

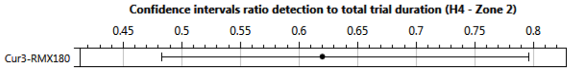

Confidence intervals ratio detection to total trial duration (H4 - Zone 2)

Genotype Zone 1	Genotype Zone 2	Mean	Lower 95% CL	Upper 95% CL	Group
Cur3	RMX180	0.6199	0.4825	0.7964	a

Model summary

```
Linear mixed model fit by REML. t-tests use Satterthwaite's method ['lmerModLmerTest']
Formula: Ratio_detection_to_total_trial_duration_H4_Zone_2 ~ 1 + (1 |      Genotype_Zone_1:Plant_Zone_1)  
+ (1 | Genotype_Zone_2:Plant_Zone_2)
   Data: data

REML criterion at convergence: 59.5

Scaled residuals: 
    Min      1Q  Median      3Q     Max 
-3.1287 -0.4517  0.2667  0.6606  1.0981 

Random effects:
 Groups                     Name        Variance Std.Dev.
 Genotype_Zone_1:Plant_Zone_1 (Intercept) 0.03251  0.1803  
 Genotype_Zone_2:Plant_Zone_2 (Intercept) 0.02458  0.1568  
 Residual                               0.23127  0.4809  
Number of obs: 37, groups:  Genotype_Zone_1:Plant_Zone_1, 10; Genotype_Zone_2:Plant_Zone_2, 10

Fixed effects:
            Estimate Std. Error      df t value Pr(>|t|)   
(Intercept)  -0.4782     0.1098  8.5227  -4.355  0.00209 **
---
Signif. codes:  0 '***' 0.001 '**' 0.01 '*' 0.05 '.' 0.1 ' ' 1
```

Model residuals

Statistic	Value
Sample skewness	-1.482
Sample excess kurtosis	2.477
Passed Shapiro Wilk normality test	No (p-value = 0.0003936 < 0.05)

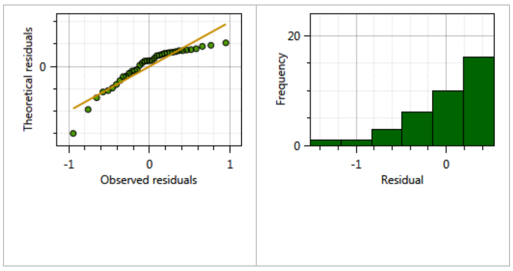

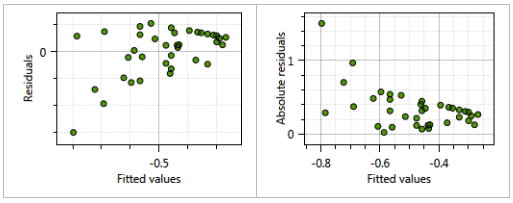

Analysis ratio detection to total trial duration (H5 - Zone 1)

**Analysis model**	Linear mixed model fit by REML: Ratio_detection_to_total_trial_duration_H5_Zone_1 ~ 1 + (1|Genotype_Zone_1:Plant_Zone_1) + (1|Genotype_Zone_2:Plant_Zone_2)
**Transformation**	Natural logarithm

Confidence intervals ratio detection to total trial duration (H5 - Zone 1)

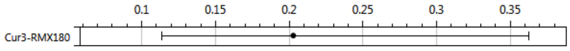

Confidence intervals ratio detection to total trial duration (H5 - Zone 1)

Genotype Zone 1	Genotype Zone 2	Mean	Lower 95% CL	Upper 95% CL	Group
Cur3	RMX180	0.2028	0.1133	0.3628	a

Model summary

```
Linear mixed model fit by REML. t-tests use Satterthwaite's method ['lmerModLmerTest']
Formula: Ratio_detection_to_total_trial_duration_H5_Zone_1 ~ 1 + (1 |      Genotype_Zone_1:Plant_Zone_1)
+ (1 | Genotype_Zone_2:Plant_Zone_2)
   Data: data

REML criterion at convergence: 86.8

Scaled residuals:
    Min      1Q  Median      3Q     Max
-2.4516 -0.4766  0.2782  0.6566  1.3289

Random effects:
 Groups                       Name        Variance Std.Dev.
 Genotype_Zone_1:Plant_Zone_1 (Intercept) 0.0000   0.0000
 Genotype_Zone_2:Plant_Zone_2 (Intercept) 0.3009   0.5485
 Residual                                 0.9346   0.9668
Number of obs: 29, groups:  Genotype_Zone_1:Plant_Zone_1, 10; Genotype_Zone_2:Plant_Zone_2, 10

Fixed effects:
            Estimate Std. Error      df t value Pr(>|t|)
(Intercept)  -1.5957     0.2522  7.9901  -6.326 0.000227 ***
---
Signif. codes:  0 '***' 0.001 '**' 0.01 '*' 0.05 '.' 0.1 ' ' 1
```

Model residuals

Statistic	Value
Sample skewness	-1.063
Sample excess kurtosis	0.6642
Passed Shapiro Wilk normality test	No (p-value = 0.0133 < 0.05)

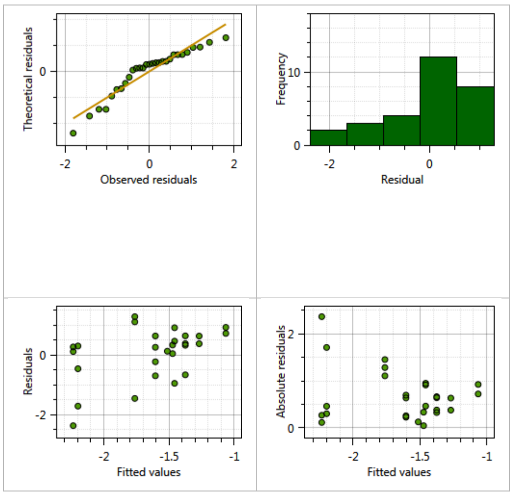

Analysis ratio detection to total trial duration (H5 - Zone 2)

**Analysis model**	Linear mixed model fit by REML: Ratio_detection_to_total_trial_duration_H5_Zone_2 ~ 1 + (1|Genotype_Zone_1:Plant_Zone_1) + (1|Genotype_Zone_2:Plant_Zone_2)
**Transformation**	Natural logarithm

Confidence intervals ratio detection to total trial duration (H5 - Zone 2)

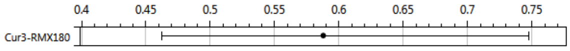

Confidence intervals ratio detection to total trial duration (H5 - Zone 2)

Genotype Zone 1	Genotype Zone 2	Mean	Lower 95% CL	Upper 95% CL	Group
Cur3	RMX180	0.5878	0.462	0.7478	a

Model summary

```
Linear mixed model fit by REML. t-tests use Satterthwaite's method ['lmerModLmerTest']
Formula: Ratio_detection_to_total_trial_duration_H5_Zone_2 ~ 1 + (1 |      Genotype_Zone_1:Plant_Zone_1)
+ (1 | Genotype_Zone_2:Plant_Zone_2)
   Data: data

REML criterion at convergence: 76.5

Scaled residuals:
    Min      1Q  Median      3Q     Max
-2.5891 -0.3794  0.4274  0.7686  0.9352

Random effects:
 Groups                       Name        Variance  Std.Dev.
 Genotype_Zone_1:Plant_Zone_1 (Intercept) 1.156e-16 1.075e-08
 Genotype_Zone_2:Plant_Zone_2 (Intercept) 1.463e-02 1.209e-01
 Residual                                 3.851e-01 6.206e-01
Number of obs: 39, groups:  Genotype_Zone_1:Plant_Zone_1, 10; Genotype_Zone_2:Plant_Zone_2, 10

Fixed effects:
            Estimate Std. Error      df t value Pr(>|t|)
(Intercept)  -0.5314     0.1065  9.0462  -4.989 0.000739 ***
```

```
---
Signif. codes:  0 '***' 0.001 '**' 0.01 '*' 0.05 '.' 0.1 ' ' 1
```

Model residuals

Statistic	Value
Sample skewness	-1.215
Sample excess kurtosis	0.4714
Passed Shapiro Wilk normality test	No (p-value = 4.342E-05 < 0.05)

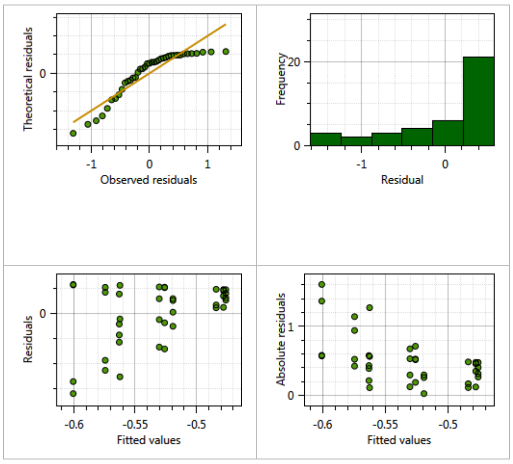

Analysis ratio detection to total trial duration (H6 - Zone 1)

**Analysis model**	Linear mixed model fit by REML: Ratio_detection_to_total_trial_duration_H6_Zone_1 ~ 1 + (1|Genotype_Zone_1:Plant_Zone_1) + (1|Genotype_Zone_2:Plant_Zone_2)
**Transformation**	Natural logarithm

Confidence intervals ratio detection to total trial duration (H6 - Zone 1)

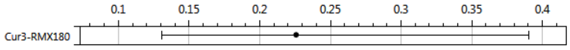

Confidence intervals ratio detection to total trial duration (H6 - Zone 1)

Genotype Zone 1	Genotype Zone 2	Mean	Lower 95% CL	Upper 95% CL	Group
Cur3	RMX180	0.2257	0.1304	0.3905	a

Model summary

```
Linear mixed model fit by REML. t-tests use Satterthwaite's method ['lmerModLmerTest']
Formula: Ratio_detection_to_total_trial_duration_H6_Zone_1 ~ 1 + (1 |      Genotype_Zone_1:Plant_Zone_1)
+ (1 | Genotype_Zone_2:Plant_Zone_2)
   Data: data

REML criterion at convergence: 73.3

Scaled residuals:
    Min      1Q  Median      3Q     Max
-2.0698 -0.5853  0.2072  0.4936  1.2656

Random effects:
 Groups                       Name        Variance Std.Dev.
 Genotype_Zone_1:Plant_Zone_1 (Intercept) 0.08166  0.2858
 Genotype_Zone_2:Plant_Zone_2 (Intercept) 0.17827  0.4222
 Residual                                 0.66201  0.8136
Number of obs: 27, groups:  Genotype_Zone_1:Plant_Zone_1, 10; Genotype_Zone_2:Plant_Zone_2, 10

Fixed effects:
            Estimate Std. Error      df t value Pr(>|t|)
(Intercept)  -1.4886     0.2302  6.7571  -6.466    4e-04 ***
---
Signif. codes:  0 '***' 0.001 '**' 0.01 '*' 0.05 '.' 0.1 ' ' 1
```

Model residuals

Statistic	Value
Sample skewness	-0.705
Sample excess kurtosis	0.04792
Passed Shapiro Wilk normality test	Yes (p-value = 0.1392 > 0.05)

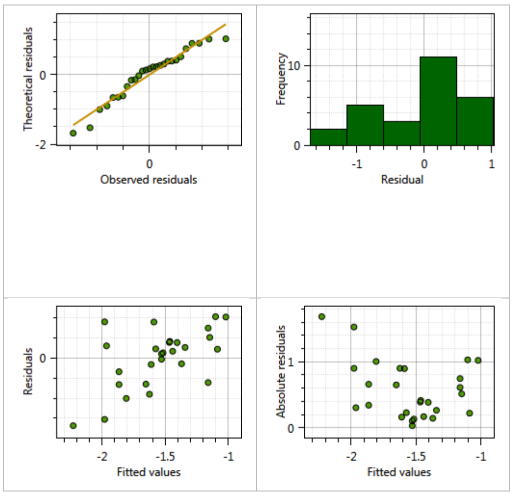

Analysis ratio detection to total trial duration (H6 - Zone 2)

**Analysis model**	Linear mixed model fit by REML: Ratio_detection_to_total_trial_duration_H6_Zone_2 ~ 1 + (1|Genotype_Zone_1:Plant_Zone_1) + (1|Genotype_Zone_2:Plant_Zone_2)
**Transformation**	Natural logarithm

Confidence intervals ratio detection to total trial duration (H6 - Zone 2)

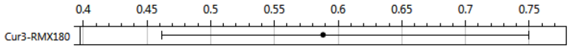

Confidence intervals ratio detection to total trial duration (H6 - Zone 2)

Genotype Zone 1	Genotype Zone 2	Mean	Lower 95% CL	Upper 95% CL	Group
Cur3	RMX180	0.5881	0.4613	0.7498	a

Model summary

```
Linear mixed model fit by REML. t-tests use Satterthwaite's method ['lmerModLmerTest']
Formula: Ratio_detection_to_total_trial_duration_H6_Zone_2 ~ 1 + (1 |      Genotype_Zone_1:Plant_Zone_1)
+ (1 | Genotype_Zone_2:Plant_Zone_2)
   Data: data

REML criterion at convergence: 86.3

Scaled residuals:
    Min      1Q  Median      3Q     Max
-4.0268 -0.3563  0.3108  0.7183  0.7183

Random effects:
 Groups                       Name        Variance Std.Dev.
 Genotype_Zone_1:Plant_Zone_1 (Intercept) 0.0000   0.000
 Genotype_Zone_2:Plant_Zone_2 (Intercept) 0.0000   0.000
 Residual                                 0.5461   0.739
Number of obs: 38, groups:  Genotype_Zone_1:Plant_Zone_1, 10; Genotype_Zone_2:Plant_Zone_2, 10

Fixed effects:
            Estimate Std. Error      df t value Pr(>|t|)
(Intercept)  -0.5308     0.1199 37.0000  -4.428 8.13e-05 ***
---
Signif. codes:  0 '***' 0.001 '**' 0.01 '*' 0.05 '.' 0.1 ' ' 1
```

Model residuals

Statistic	Value
Sample skewness	-2.618
Sample excess kurtosis	8.16
Passed Shapiro Wilk normality test	No (p-value = 1.17E-07 < 0.05)

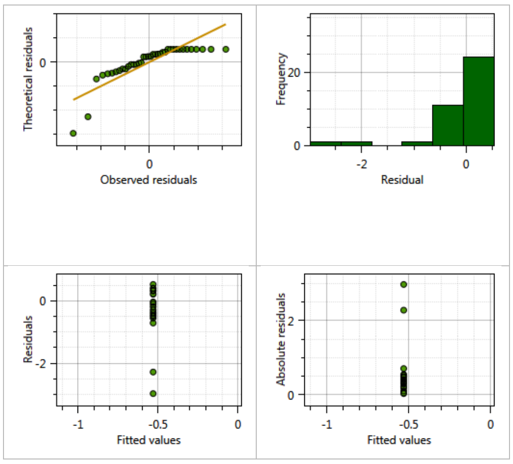

Analysis ratio detection to total trial duration (H7 - Zone 1)

**Analysis model**	Linear mixed model fit by REML: Ratio_detection_to_total_trial_duration_H7_Zone_1 ~ 1 + (1|Genotype_Zone_1:Plant_Zone_1) + (1|Genotype_Zone_2:Plant_Zone_2)
**Transformation**	Natural logarithm

Confidence intervals ratio detection to total trial duration (H7 - Zone 1)

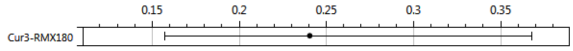

Confidence intervals ratio detection to total trial duration (H7 - Zone 1)

Genotype Zone 1	Genotype Zone 2	Mean	Lower 95% CL	Upper 95% CL	Group
Cur3	RMX180	0.2404	0.1571	0.368	a

Model summary

```
Linear mixed model fit by REML. t-tests use Satterthwaite's method ['lmerModLmerTest']
Formula: Ratio_detection_to_total_trial_duration_H7_Zone_1 ~ 1 + (1 |      Genotype_Zone_1:Plant_Zone_1)
+ (1 | Genotype_Zone_2:Plant_Zone_2)
   Data: data

REML criterion at convergence: 60.2

Scaled residuals:
    Min      1Q  Median      3Q     Max
-1.8253 -0.8450  0.1462  0.8812  1.2143

Random effects:
 Groups                       Name        Variance Std.Dev.
 Genotype_Zone_2:Plant_Zone_2 (Intercept) 0.1338   0.3658
 Genotype_Zone_1:Plant_Zone_1 (Intercept) 0.0000   0.0000
 Residual                                 0.4729   0.6877
Number of obs: 26, groups:  Genotype_Zone_2:Plant_Zone_2, 10; Genotype_Zone_1:Plant_Zone_1, 9

Fixed effects:
            Estimate Std. Error      df t value Pr(>|t|)
(Intercept)  -1.4254     0.1831  7.6338  -7.787 6.84e-05 ***
---
Signif. codes:  0 '***' 0.001 '**' 0.01 '*' 0.05 '.' 0.1 ' ' 1
```

Model residuals

Statistic	Value
Sample skewness	-0.3609
Sample excess kurtosis	-1.277
Passed Shapiro Wilk normality test	No (p-value = 0.01941 < 0.05)

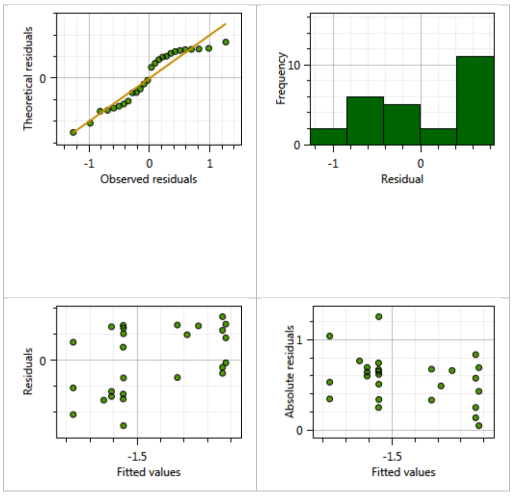

Analysis ratio detection to total trial duration (H7 - Zone 2)

**Analysis model**	Linear mixed model fit by REML: Ratio_detection_to_total_trial_duration_H7_Zone_2 ~ 1 + (1|Genotype_Zone_1:Plant_Zone_1) + (1|Genotype_Zone_2:Plant_Zone_2)
**Transformation**	Natural logarithm

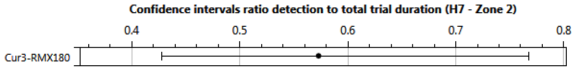

Confidence intervals ratio detection to total trial duration (H7 - Zone 2)

Genotype Zone 1	Genotype Zone 2	Mean	Lower 95% CL	Upper 95% CL	Group
Cur3	RMX180	0.5729	0.4275	0.7678	a

Model summary

```
Linear mixed model fit by REML. t-tests use Satterthwaite's method ['lmerModLmerTest']
Formula: Ratio_detection_to_total_trial_duration_H7_Zone_2 ~ 1 + (1 |      Genotype_Zone_1:Plant_Zone_1)
+ (1 | Genotype_Zone_2:Plant_Zone_2)
   Data: data

REML criterion at convergence: 72.6

Scaled residuals:
    Min      1Q  Median      3Q     Max
-2.4722 -0.5564  0.2865  0.9194  0.9337

Random effects:
 Groups                       Name        Variance  Std.Dev.
 Genotype_Zone_1:Plant_Zone_1 (Intercept) 0.0005236 0.02288
 Genotype_Zone_2:Plant_Zone_2 (Intercept) 0.0001804 0.01343
 Residual                                 0.3586888 0.59891
Number of obs: 39, groups:  Genotype_Zone_1:Plant_Zone_1, 10; Genotype_Zone_2:Plant_Zone_2, 10

Fixed effects:
            Estimate Std. Error       df t value Pr(>|t|)
(Intercept) -0.55704    0.09627  3.26481  -5.786   0.0081 **
---
Signif. codes:  0 '***' 0.001 '**' 0.01 '*' 0.05 '.' 0.1 ' ' 1
```

Model residuals

Statistic	Value
Sample skewness	-0.9717
Sample excess kurtosis	0.0272
Passed Shapiro Wilk normality test	No (p-value = 0.0001446 < 0.05)

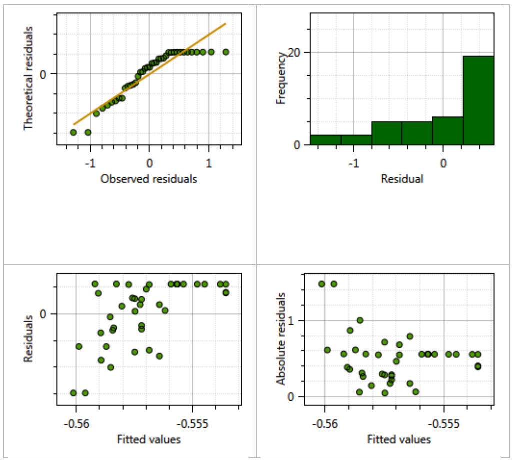

Analysis ratio detection to total trial duration H0 (diff. Zone 1 - Zone 2)

**Analysis model**	Generalized linear mixed model with dispersion factor, formula=cbind(Ratio_detection_to_total_trial_duration_H0_Zone_1,Ratio_detection_to_total_trial_duration_H0_Zone_2) ~ 1 + (1|Genotype_Zone_1:Plant_Zone_1) + (1|Genotype_Zone_2:Plant_Zone_2)
**Transformation**	Logit

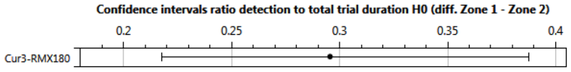

Confidence intervals ratio detection to total trial duration H0 (diff. Zone 1 - Zone 2)

Genotype Zone 1	Genotype Zone 2	Mean	Lower 95% CL	Upper 95% CL	Group
Cur3	RMX180	0.2955	0.2175	0.3877	a

Model summary

```
Linear mixed model fit by REML. t-tests use Satterthwaite's method ['lmerModLmerTest']
Formula: ziFormula
   Data: data
```

```
Weights: wi

REML criterion at convergence: 129.8

Scaled residuals:
    Min      1Q  Median      3Q     Max
-1.1641 -0.8159 -0.1309  0.6177  2.5983

Random effects:
 Groups                      Name        Variance Std.Dev.
 Genotype_Zone_1:Plant_Zone_1 (Intercept) 0.0000   0.0000
 Genotype_Zone_2:Plant_Zone_2 (Intercept) 0.0000   0.0000
 Residual                                 0.3096   0.5564
Number of obs: 39, groups:  Genotype_Zone_1:Plant_Zone_1, 10; Genotype_Zone_2:Plant_Zone_2, 10

Fixed effects:
            Estimate Std. Error      df t value Pr(>|t|)
(Intercept)  -0.8687     0.2033 38.0000  -4.273 0.000125 ***
---
Signif. codes:  0 '***' 0.001 '**' 0.01 '*' 0.05 '.' 0.1 ' ' 1

Dispersion: 0.5564
```

Model residuals

Statistic	Value
Sample skewness	0.7694
Sample excess kurtosis	0.03535
Passed Shapiro Wilk normality test	No (p-value = 0.01346 < 0.05)

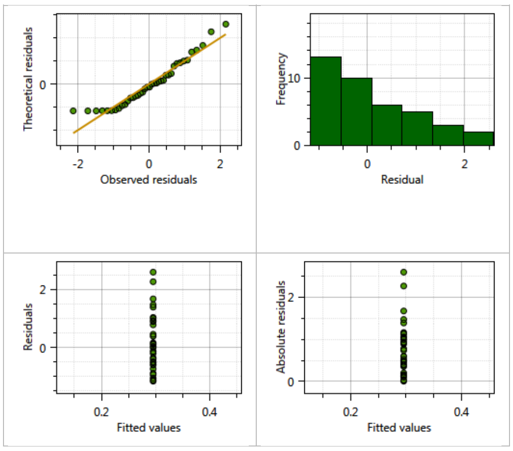

Analysis ratio detection to total trial duration H1 (diff. Zone 1 - Zone 2)

**Analysis model**	Generalized linear mixed model with dispersion factor, formula=cbind(Ratio_detection_to_total_trial_duration_H1_Zone_1,Ratio_detection_to_total_trial_duration_H1_Zone_2) ~ 1 + (1|Genotype_Zone_1:Plant_Zone_1) + (1|Genotype_Zone_2:Plant_Zone_2)
**Transformation**	Logit

Confidence intervals ratio detection to total trial duration H1 (diff. Zone 1 - Zone 2)

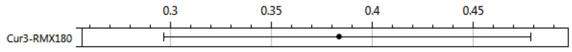

Confidence intervals ratio detection to total trial duration H1 (diff. Zone 1 - Zone 2)

Genotype Zone 1	Genotype Zone 2	Mean	Lower 95% CL	Upper 95% CL	Group
Cur3	RMX180	0.3835	0.2965	0.4786	a

Model summary

```
Linear mixed model fit by REML. t-tests use Satterthwaite's method ['lmerModLmerTest']
Formula: ziFormula
   Data: data
Weights: wi

REML criterion at convergence: 125.6

Scaled residuals:
    Min      1Q  Median      3Q     Max
-1.4522 -0.7141 -0.1003  0.7887  2.3348

Random effects:
 Groups                      Name        Variance  Std.Dev.
 Genotype_Zone_1:Plant_Zone_1 (Intercept) 3.832e-17 6.191e-09
 Genotype_Zone_2:Plant_Zone_2 (Intercept) 0.000e+00 0.000e+00
 Residual                                 2.949e-01 5.431e-01
Number of obs: 39, groups:  Genotype_Zone_1:Plant_Zone_1, 10; Genotype_Zone_2:Plant_Zone_2, 10

Fixed effects:
            Estimate Std. Error      df t value Pr(>|t|)
(Intercept)  -0.4748     0.1922 38.0000   -2.47   0.0181 *
---
Signif. codes:  0 '***' 0.001 '**' 0.01 '*' 0.05 '.' 0.1 ' ' 1

Dispersion: 0.5431
```

Model residuals

Statistic	Value
Sample skewness	0.5024
Sample excess kurtosis	-0.4993
Passed Shapiro Wilk normality test	Yes (p-value = 0.1762 > 0.05)

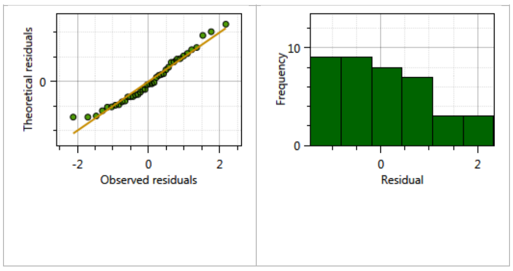

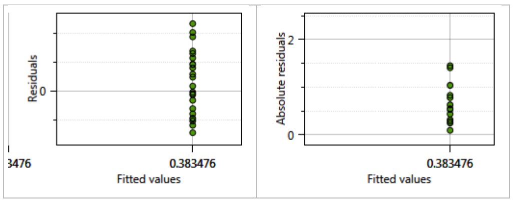

Analysis ratio detection to total trial duration H2 (diff. Zone 1 - Zone 2)

**Analysis model**	Generalized linear mixed model with dispersion factor, formula=cbind(Ratio_detection_to_total_trial_duration_H2_Zone_1,Ratio_detection_to_total_trial_duration_H2_Zone_2) ~ 1 + (1|Genotype_Zone_1:Plant_Zone_1) + (1|Genotype_Zone_2:Plant_Zone_2)
**Transformation**	Logit

Confidence intervals ratio detection to total trial duration H2 (diff. Zone 1 - Zone 2)

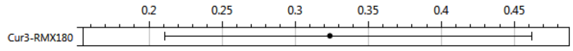

Confidence intervals ratio detection to total trial duration H2 (diff. Zone 1 - Zone 2)

Genotype Zone 1	Genotype Zone 2	Mean	Lower 95% CL	Upper 95% CL	Group
Cur3	RMX180	0.3237	0.2106	0.4621	a

Model summary

```
Linear mixed model fit by REML. t-tests use Satterthwaite's method ['lmerModLmerTest']
Formula: ziFormula
   Data: data
Weights: wi

REML criterion at convergence: 135.1

Scaled residuals: 
    Min      1Q  Median      3Q     Max 
-1.5051 -0.7487 -0.3157  0.4712  2.2142 

Random effects:
 Groups                      Name        Variance  Std.Dev. 
 Genotype_Zone_1:Plant_Zone_1 (Intercept) 2.496e-01 4.996e-01
 Genotype_Zone_2:Plant_Zone_2 (Intercept) 3.337e-15 5.777e-08
 Residual                                 3.049e-01 5.522e-01
Number of obs: 39, groups:  Genotype_Zone_1:Plant_Zone_1, 10; Genotype_Zone_2:Plant_Zone_2, 10

Fixed effects:
            Estimate Std. Error      df t value Pr(>|t|)  
(Intercept)  -0.7366     0.2589  9.0773  -2.846   0.0191 *
---
Signif. codes:  0 '***' 0.001 '**' 0.01 '*' 0.05 '.' 0.1 ' ' 1

Dispersion: 0.5522
```

Model residuals

Statistic	Value
Sample skewness	0.9731
Sample excess kurtosis	0.1738
Passed Shapiro Wilk normality test	No (p-value = 0.002093 < 0.05)

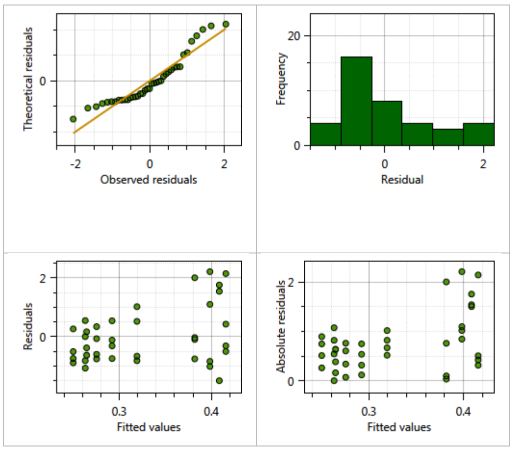

Analysis ratio detection to total trial duration H3 (diff. Zone 1 - Zone 2)

**Analysis model**	Generalized linear mixed model with dispersion factor, formula=cbind(Ratio_detection_to_total_trial_duration_H3_Zone_1,Ratio_detection_to_total_trial_duration_H3_Zone_2) ~ 1 + (1|Genotype_Zone_1:Plant_Zone_1) + (1|Genotype_Zone_2:Plant_Zone_2)
**Transformation**	Logit

Confidence intervals ratio detection to total trial duration H3 (diff. Zone 1 - Zone 2)

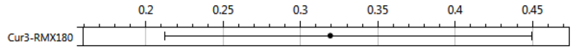

Confidence intervals ratio detection to total trial duration H3 (diff. Zone 1 - Zone 2)

Genotype Zone 1	Genotype Zone 2	Mean	Lower 95% CL	Upper 95% CL	Group
Cur3	RMX180	0.3192	0.212	0.4497	a

Model summary

```
Linear mixed model fit by REML. t-tests use Satterthwaite's method ['lmerModLmerTest']
Formula: ziFormula
   Data: data
Weights: wi

REML criterion at convergence: 130

Scaled residuals: 
     Min       1Q   Median       3Q      Max 
-1.33960 -0.88186  0.05801  0.73774  1.86709 

Random effects:
 Groups                      Name        Variance Std.Dev.
 Genotype_Zone_1:Plant_Zone_1 (Intercept) 0.0000   0.0000  
 Genotype_Zone_2:Plant_Zone_2 (Intercept) 0.2402   0.4902  
 Residual                                 0.2639   0.5137  
Number of obs: 39, groups:  Genotype_Zone_1:Plant_Zone_1, 10; Genotype_Zone_2:Plant_Zone_2, 10

Fixed effects:
            Estimate Std. Error      df t value Pr(>|t|)
```

```
(Intercept)  -0.7575     0.2463  9.1691  -3.075    0.013 *
---
Signif. codes:  0 '***' 0.001 '**' 0.01 '*' 0.05 '.' 0.1 ' ' 1

Dispersion: 0.5137
```

Model residuals

Statistic	Value
Sample skewness	0.3258
Sample excess kurtosis	-1.032
Passed Shapiro Wilk normality test	No (p-value = 0.01985 < 0.05)

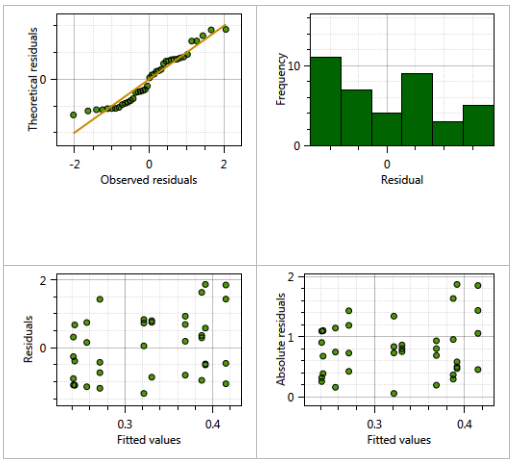

Analysis ratio detection to total trial duration H4 (diff. Zone 1 - Zone 2)

**Analysis model**	Generalized linear mixed model with dispersion factor, formula=cbind(Ratio_detection_to_total_trial_duration_H4_Zone_1,Ratio_detection_to_total_trial_duration_H4_Zone_2) ~ 1 + (1|Genotype_Zone_1:Plant_Zone_1) + (1|Genotype_Zone_2:Plant_Zone_2)
**Transformation**	Logit

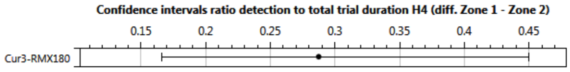

Confidence intervals ratio detection to total trial duration H4 (diff. Zone 1 - Zone 2)

Genotype Zone 1	Genotype Zone 2	Mean	Lower 95% CL	Upper 95% CL	Group
Cur3	RMX180	0.2873	0.1657	0.4501	a

Model summary

```
Linear mixed model fit by REML. t-tests use Satterthwaite's method ['lmerModLmerTest']
Formula: ziFormula
   Data: data
Weights: wi

REML criterion at convergence: 141.9

Scaled residuals:
    Min      1Q  Median      3Q     Max
-1.0600 -0.7917 -0.3571  0.6688  2.2913

Random effects:
 Groups                      Name        Variance Std.Dev.
 Genotype_Zone_1:Plant_Zone_1 (Intercept) 0.09714  0.3117
 Genotype_Zone_2:Plant_Zone_2 (Intercept) 0.25089  0.5009
 Residual                                 0.35597  0.5966
Number of obs: 39, groups:  Genotype_Zone_1:Plant_Zone_1, 10; Genotype_Zone_2:Plant_Zone_2, 10

Fixed effects:
            Estimate Std. Error      df t value Pr(>|t|)
(Intercept)  -0.9083     0.2906  6.1081  -3.125     0.02 *
---
Signif. codes:  0 '***' 0.001 '**' 0.01 '*' 0.05 '.' 0.1 ' ' 1

Dispersion: 0.5966
```

Model residuals

Statistic	Value
Sample skewness	0.9578
Sample excess kurtosis	-0.09638
Passed Shapiro Wilk normality test	No (p-value = 0.0005458 < 0.05)

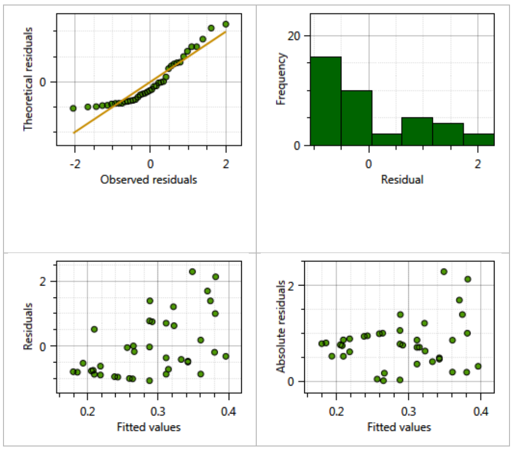

Analysis ratio detection to total trial duration H5 (diff. Zone 1 - Zone 2)

**Analysis model**	Generalized linear mixed model with dispersion factor, formula=cbind(Ratio_detection_to_total_trial_duration_H5_Zone_1,Ratio_detection_to_total_trial_duration_H5_Zone_2) ~ 1 + (1|Genotype_Zone_1:Plant_Zone_1) + (1|Genotype_Zone_2:Plant_Zone_2)
**Transformation**	Logit

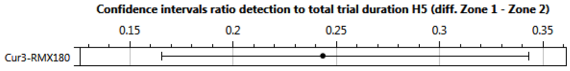

Confidence intervals ratio detection to total trial duration H5 (diff. Zone 1 - Zone 2)

Genotype Zone 1	Genotype Zone 2	Mean	Lower 95% CL	Upper 95% CL	Group
Cur3	RMX180	0.2435	0.1653	0.3434	a

Model summary

```
Linear mixed model fit by REML. t-tests use Satterthwaite's method ['lmerModLmerTest']
Formula: ziFormula
   Data: data
Weights: wi

REML criterion at convergence: 142.5

Scaled residuals: 
    Min      1Q  Median      3Q     Max 
-0.9287 -0.9002 -0.3260  0.6615  2.4329 

Random effects:
 Groups                      Name        Variance Std.Dev.
 Genotype_Zone_1:Plant_Zone_1 (Intercept) 0.0000   0.0000  
 Genotype_Zone_2:Plant_Zone_2 (Intercept) 0.0000   0.0000  
 Residual                                 0.3731   0.6108  
Number of obs: 39, groups:  Genotype_Zone_1:Plant_Zone_1, 10; Genotype_Zone_2:Plant_Zone_2, 10

Fixed effects:
            Estimate Std. Error      df t value Pr(>|t|)    
(Intercept)  -1.1337     0.2398 38.0000  -4.729 3.09e-05 ***
---
Signif. codes:  0 '***' 0.001 '**' 0.01 '*' 0.05 '.' 0.1 ' ' 1

Dispersion: 0.6108
```

Model residuals

Statistic	Value
Sample skewness	0.8257
Sample excess kurtosis	-0.4485
Passed Shapiro Wilk normality test	No (p-value = 0.0002664 < 0.05)

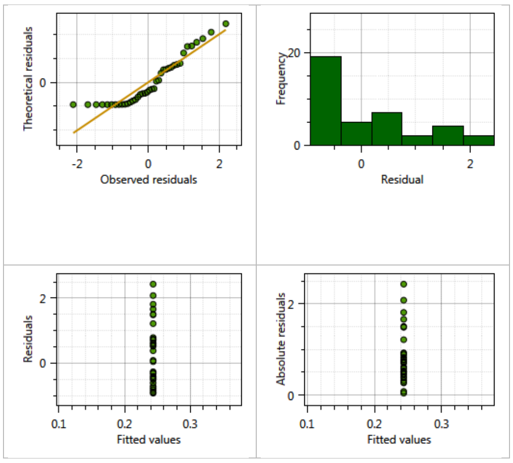

Analysis ratio detection to total trial duration H6 (diff. Zone 1 - Zone 2)

**Analysis model**	Generalized linear mixed model with dispersion factor, formula=cbind(Ratio_detection_to_total_trial_duration_H6_Zone_1,Ratio_detection_to_total_trial_duration_H6_Zone_2) ~ 1 + (1|Genotype_Zone_1:Plant_Zone_1) + (1|Genotype_Zone_2:Plant_Zone_2)
**Transformation**	Logit

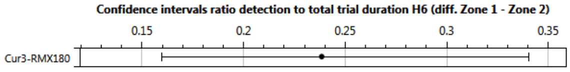

Confidence intervals ratio detection to total trial duration H6 (diff. Zone 1 - Zone 2)

Genotype Zone 1	Genotype Zone 2	Mean	Lower 95% CL	Upper 95% CL	Group
Cur3	RMX180	0.2384	0.1595	0.3405	a

Model summary

```
Linear mixed model fit by REML. t-tests use Satterthwaite's method ['lmerModLmerTest']
Formula: ziFormula
   Data: data
Weights: wi

REML criterion at convergence: 145.5

Scaled residuals: 
    Min      1Q  Median      3Q     Max 
-0.9034 -0.9034 -0.2593  0.5986  2.8858 

Random effects:
 Groups                      Name        Variance  Std.Dev. 
 Genotype_Zone_1:Plant_Zone_1 (Intercept) 1.703e-15 4.126e-08
 Genotype_Zone_2:Plant_Zone_2 (Intercept) 0.000e+00 0.000e+00
 Residual                                 3.836e-01 6.193e-01
Number of obs: 39, groups:  Genotype_Zone_1:Plant_Zone_1, 10; Genotype_Zone_2:Plant_Zone_2, 10

Fixed effects:
            Estimate Std. Error      df t value Pr(>|t|)    
(Intercept)  -1.1613     0.2472 38.0000  -4.697  3.4e-05 ***
---
Signif. codes:  0 '***' 0.001 '**' 0.01 '*' 0.05 '.' 0.1 ' ' 1

Dispersion: 0.6193
```

Model residuals

Statistic	Value
Sample skewness	1.12
Sample excess kurtosis	0.9587
Passed Shapiro Wilk normality test	No (p-value = 0.0001356 < 0.05)

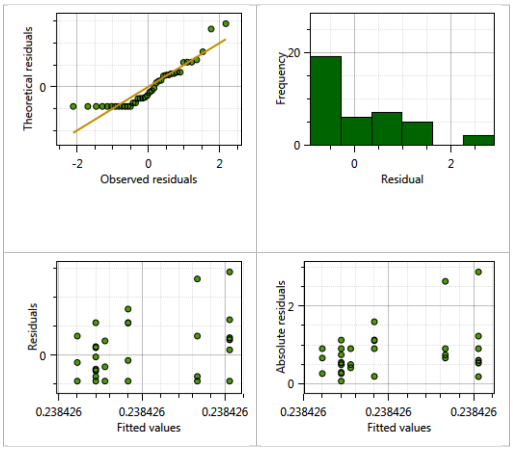

Analysis ratio detection to total trial duration H7 (diff. Zone 1 - Zone 2)

**Analysis model**	Generalized linear mixed model with dispersion factor, formula=cbind(Ratio_detection_to_total_trial_duration_H7_Zone_1,Ratio_detection_to_total_trial_duration_H7_Zone_2) ~ 1 + (1|Genotype_Zone_1:Plant_Zone_1) + (1|Genotype_Zone_2:Plant_Zone_2)
**Transformation**	Logit

Confidence intervals ratio detection to total trial duration H7 (diff. Zone 1 - Zone 2)

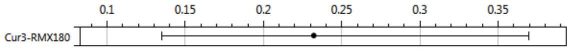

Confidence intervals ratio detection to total trial duration H7 (diff. Zone 1 - Zone 2)

Genotype Zone 1	Genotype Zone 2	Mean	Lower 95% CL	Upper 95% CL	Group
Cur3	RMX180	0.2321	0.1347	0.3698	a

Model summary

```
Linear mixed model fit by REML. t-tests use Satterthwaite's method ['lmerModLmerTest']
Formula: ziFormula
   Data: data
Weights: wi

REML criterion at convergence: 141

Scaled residuals:
    Min      1Q  Median      3Q     Max
-1.1158 -0.8799 -0.3593  0.9196  1.8290

Random effects:
 Groups                        Name        Variance Std.Dev.
 Genotype_Zone_1:Plant_Zone_1 (Intercept) 0.05914  0.2432
 Genotype_Zone_2:Plant_Zone_2 (Intercept) 0.19393  0.4404
 Residual                                 0.28856  0.5372
Number of obs: 39, groups:  Genotype_Zone_1:Plant_Zone_1, 10; Genotype_Zone_2:Plant_Zone_2, 10

Fixed effects:
            Estimate Std. Error      df t value Pr(>|t|)
(Intercept)  -1.1964     0.2718  6.0633  -4.403  0.00444 **
---
Signif. codes:  0 '***' 0.001 '**' 0.01 '*' 0.05 '.' 0.1 ' ' 1

Dispersion: 0.5372
```

Model residuals

Statistic	Value
Sample skewness	0.6683
Sample excess kurtosis	-0.8967
Passed Shapiro Wilk normality test	No (p-value = 0.0006093 < 0.05)

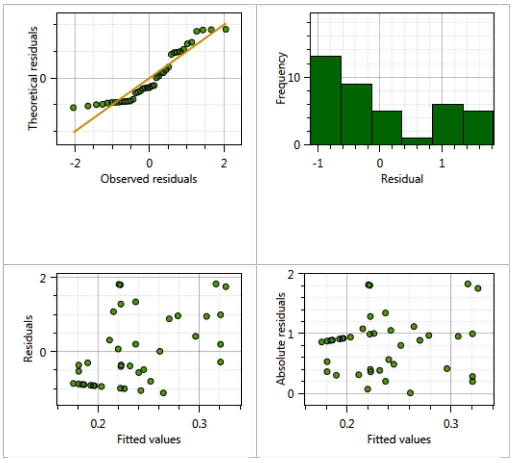

Ratio halting to detection duration per zone

Selected zones	Zone 1, Zone 2
Data transformation	Natural logarithm
Analysis	Zone difference analysis

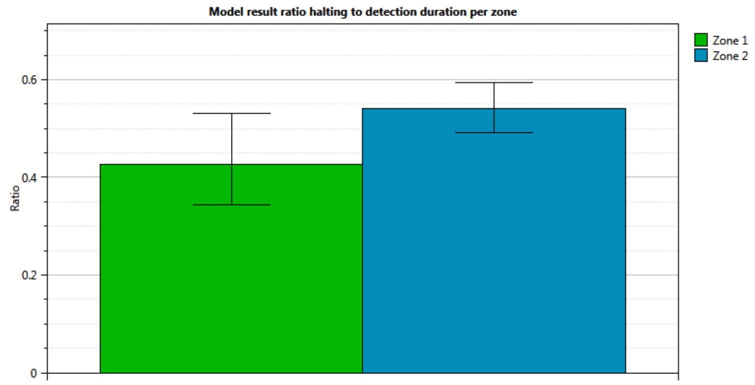

Results difference tests Zone 1 - Zone 2: p values and 95% confidence intervals of the difference on the transformed scale for each statistic.

Behaviour statistic	Cur3-RMX180
Ratio halting to detection duration (diff. Zone 1 - Zone 2)	p=0.0291*
	[-0.341, -0.0247]

The model predictions and 95% confidence intervals for each statistic.

Statistic	Cur3-RMX180
Ratio halting to detection duration (Zone 1)	0.427
	[0.344, 0.531]
Ratio halting to detection duration (Zone 2)	0.541
	[0.492, 0.594]

Data summary

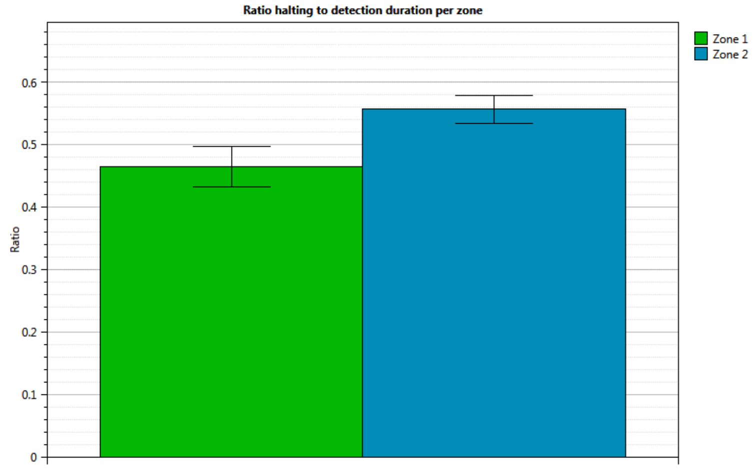

Genotype Zone 1	Genotype Zone 2	Genotype Zone 3	Mean Zone 1	StdErr Zone 1	Mean Zone 2	StdErr Zone 2
Cur3	RMX180	Neutral	0.46	0.03	0.56	0.02

Analysis ratio halting to detection duration (Zone 1)

**Analysis model**	Linear mixed model fit by REML: Ratio_halting_to_detection_duration_Zone_1 ~ 1 + (1|Genotype_Zone_1:Plant_Zone_1) + (1|Genotype_Zone_2:Plant_Zone_2)
**Transformation**	Natural logarithm

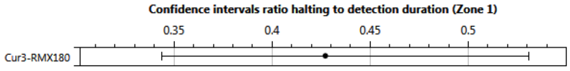

Confidence intervals ratio halting to detection duration (Zone 1)

Genotype Zone 1	Genotype Zone 2	Mean	Lower 95% CL	Upper 95% CL	Group
Cur3	RMX180	0.4271	0.3436	0.531	a

Model summary

```
Linear mixed model fit by REML. t-tests use Satterthwaite's method ['lmerModLmerTest']
Formula: Ratio_halting_to_detection_duration_Zone_1 ~ 1 + (1 | Genotype_Zone_1:Plant_Zone_1) +      (1 |
Genotype_Zone_2:Plant_Zone_2)
   Data: data

REML criterion at convergence: 47.1

Scaled residuals: 
     Min       1Q   Median       3Q      Max 
-2.46050 -0.63800 -0.09042  0.62306  1.89379 

Random effects:
 Groups                       Name        Variance Std.Dev.
 Genotype_Zone_1:Plant_Zone_1 (Intercept) 0.01570  0.1253  
 Genotype_Zone_2:Plant_Zone_2 (Intercept) 0.03653  0.1911  
 Residual                                 0.14558  0.3815  
Number of obs: 39, groups:  Genotype_Zone_1:Plant_Zone_1, 10; Genotype_Zone_2:Plant_Zone_2, 10

Fixed effects:
            Estimate Std. Error       df t value Pr(>|t|)    
(Intercept) -0.85065    0.09474  8.18513  -8.979 1.63e-05 ***
---
Signif. codes:  0 '***' 0.001 '**' 0.01 '*' 0.05 '.' 0.1 ' ' 1
```

Model residuals

Statistic	Value
Sample skewness	-0.05161
Sample excess kurtosis	0.4666
Passed Shapiro Wilk normality test	Yes (p-value = 0.6025 > 0.05)

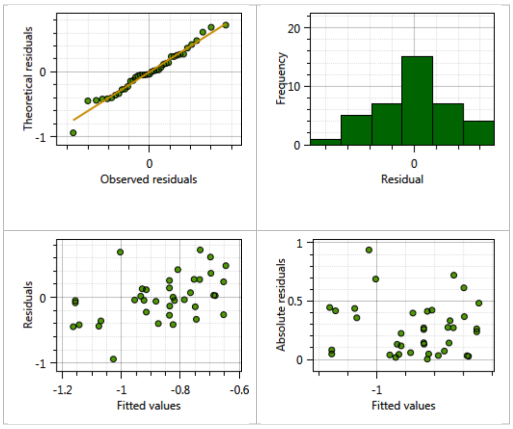

Analysis ratio halting to detection duration (Zone 2)

**Analysis model**	Linear mixed model fit by REML: Ratio_halting_to_detection_duration_Zone_2 ~ 1 + (1|Genotype_Zone_1:Plant_Zone_1) + (1|Genotype_Zone_2:Plant_Zone_2)
**Transformation**	Natural logarithm

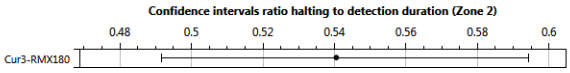

Confidence intervals ratio halting to detection duration (Zone 2)

Genotype Zone 1	Genotype Zone 2	Mean	Lower 95% CL	Upper 95% CL	Group
Cur3	RMX180	0.5405	0.4915	0.5944	a

Model summary

```
Linear mixed model fit by REML. t-tests use Satterthwaite's method ['lmerModLmerTest']
Formula: Ratio_halting_to_detection_duration_Zone_2 ~ 1 + (1 | Genotype_Zone_1:Plant_Zone_1) +      (1 |
Genotype_Zone_2:Plant_Zone_2)
   Data: data

REML criterion at convergence: 6.4

Scaled residuals: 
     Min       1Q   Median       3Q      Max 
-2.55646 -0.49021 -0.00856  0.50148  2.21447 

Random effects:
 Groups                       Name        Variance Std.Dev.
 Genotype_Zone_1:Plant_Zone_1 (Intercept) 0.000000 0.00000 
 Genotype_Zone_2:Plant_Zone_2 (Intercept) 0.001539 0.03923 
 Residual                                 0.061502 0.24800 
Number of obs: 39, groups:  Genotype_Zone_1:Plant_Zone_1, 10; Genotype_Zone_2:Plant_Zone_2, 10

Fixed effects:
            Estimate Std. Error       df t value Pr(>|t|)    
(Intercept) -0.61525    0.04161  8.51374  -14.79 2.29e-07 ***
---
Signif. codes:  0 '***' 0.001 '**' 0.01 '*' 0.05 '.' 0.1 ' ' 1
```

Model residuals

Statistic	Value
Sample skewness	0.02118
Sample excess kurtosis	0.9433
Passed Shapiro Wilk normality test	Yes (p-value = 0.2975 > 0.05)

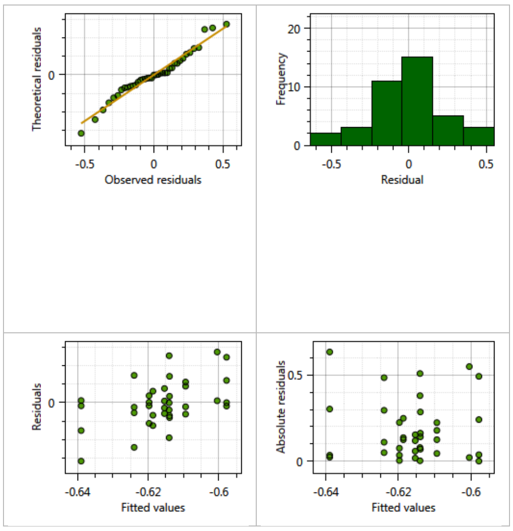

Analysis ratio halting to detection duration (diff. Zone 1 - Zone 2)

**Analysis model**	Generalized linear mixed model with dispersion factor, formula=cbind(Ratio_halting_to_detection_duration_Zone_1,Ratio_halting_to_detection_duration_Zone_2) ~ 1 + (1|Genotype_Zone_1:Plant_Zone_1) + (1|Genotype_Zone_2:Plant_Zone_2)
**Transformation**	Logit

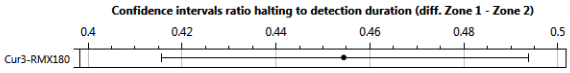

Confidence intervals ratio halting to detection duration (diff. Zone 1 - Zone 2)

Genotype Zone 1	Genotype Zone 2	Mean	Lower 95% CL	Upper 95% CL	Group
Cur3	RMX180	0.4544	0.4155	0.4938	a

Model summary

```
Linear mixed model fit by REML. t-tests use Satterthwaite's method ['lmerModLmerTest']
Formula: ziFormula
   Data: data
```

```
Weights: wi

REML criterion at convergence: 41.4

Scaled residuals:
    Min      1Q  Median      3Q     Max
-2.2198 -0.6027 -0.1352  0.5476  2.7637

Random effects:
 Groups                       Name        Variance  Std.Dev.
 Genotype_Zone_1:Plant_Zone_1 (Intercept) 3.379e-18 1.838e-09
 Genotype_Zone_2:Plant_Zone_2 (Intercept) 7.247e-03 8.513e-02
 Residual                                 3.701e-02 1.924e-01
Number of obs: 39, groups:  Genotype_Zone_1:Plant_Zone_1, 10; Genotype_Zone_2:Plant_Zone_2, 10

Fixed effects:
            Estimate Std. Error       df t value Pr(>|t|)
(Intercept) -0.18291    0.06694  7.02259  -2.733   0.0291 *
---
Signif. codes:  0 '***' 0.001 '**' 0.01 '*' 0.05 '.' 0.1 ' ' 1

Dispersion: 0.1924
```

Model residuals

Statistic	Value
Sample skewness	0.2507
Sample excess kurtosis	1.048
Passed Shapiro Wilk normality test	Yes (p-value = 0.6726 > 0.05)

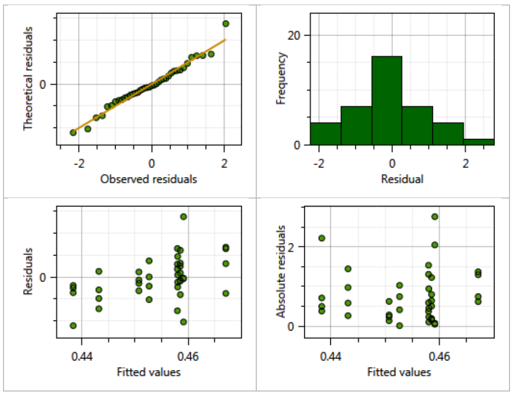

Ratio halting to detection duration per zone per hour

Selected hours	0, 1, 2, 3, 4, 5, 6, 7
Selected zones	Zone 1, Zone 2
Data transformation	Natural logarithm
Analysis	Zone difference analysis

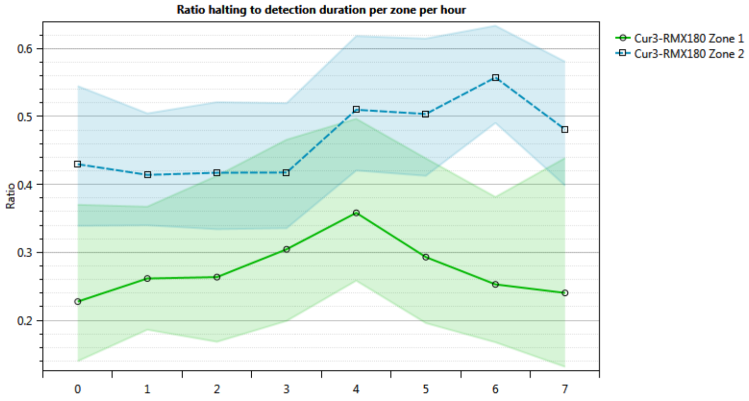

Results difference tests Zone 1 - Zone 2: p values and 95% confidence intervals of the difference on the transformed scale for each statistic.

Behaviour statistic	Cur3-RMX180
Ratio halting to detection duration H0 (diff. Zone 1 - Zone 2)	p=0.0322* [-0.73, -0.0401]
Ratio halting to detection duration H1 (diff. Zone 1 - Zone 2)	p=0.0484* [-0.557, -0.00206]
Ratio halting to detection duration H2 (diff. Zone 1 - Zone 2)	p=0.244 [-0.653, 0.199]
Ratio halting to detection duration H3 (diff. Zone 1 - Zone 2)	p=0.432 [-0.534, 0.259]
Ratio halting to detection duration H4 (diff. Zone 1 - Zone 2)	p=0.197 [-0.51, 0.11]
Ratio halting to detection duration H5 (diff. Zone 1 - Zone 2)	p=0.198 [-0.583, 0.136]
Ratio halting to detection duration H6 (diff. Zone 1 - Zone 2)	p=0.00164** [-0.91, -0.237]
Ratio halting to detection duration H7 (diff. Zone 1 - Zone 2)	p=0.0326* [-1.05, -0.0576]

The model predictions and 95% confidence intervals for each statistic.

Statistic	Cur3-RMX180	Remark
Ratio halting to detection duration (H0 - Zone 1)	0.228 [0.14, 0.37]	CR
Ratio halting to detection duration (H0 - Zone 2)	0.43 [0.339, 0.545]	CR
Ratio halting to detection duration (H1 - Zone 1)	0.262 [0.186, 0.367]	CR
Ratio halting to detection duration (H1 - Zone 2)	0.414 [0.34, 0.505]	CR
Ratio halting to detection duration (H2 - Zone 1)	0.264 [0.168, 0.413]	CR
Ratio halting to detection duration (H2 - Zone 2)	0.417 [0.334, 0.521]	CR
Ratio halting to detection duration (H3 - Zone 1)	0.305 [0.199, 0.466]	CR
Ratio halting to detection duration (H3 - Zone 2)	0.418 [0.336, 0.52]	CR
Ratio halting to detection duration (H4 - Zone 1)	0.358 [0.258, 0.497]	

Statistic	Cur3-RMX180	Remark
Ratio halting to detection duration (H4 - Zone 2)	0.51<br>[0.421, 0.619]	CR
Ratio halting to detection duration (H5 - Zone 1)	0.293<br>[0.196, 0.438]	CR
Ratio halting to detection duration (H5 - Zone 2)	0.504<br>[0.413, 0.615]	CR
Ratio halting to detection duration (H6 - Zone 1)	0.253<br>[0.168, 0.382]	
Ratio halting to detection duration (H6 - Zone 2)	0.558<br>[0.491, 0.634]	
Ratio halting to detection duration (H7 - Zone 1)	0.24<br>[0.132, 0.439]	CR
Ratio halting to detection duration (H7 - Zone 2)	0.481<br>[0.398, 0.581]	CR

CR = Check residuals

Data summary

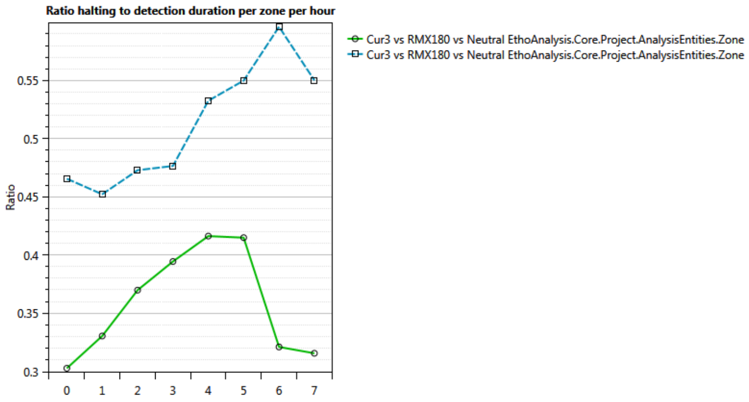

Genotype Zone 1	Genotype Zone 2	Genotype Zone 3	Mean H0 - Zone 1	StdErr H0 - Zone 1	Mean H0 - Zone 2	StdErr H0 - Zone 2	Mean H1 - Zone 1	StdErr H1 - Zone 1	Mean H1 - Zone 2	StdErr H1 - Zone 2	Mean H2 - Zone 1	StdErr H2 - Zone 1	Mean H2 - Zone 2	StdErr H2 - Zone 2	Mean H3 - Zone 1	StdErr H3 - Zone 1	Mean H3 - Zone 2	StdErr H3 - Zone 2	Mean H4 - Zone 1	StdErr H4 - Zone 1	Mean H4 - Zone 2	StdErr H4 - Zone 2	Mean H5 - Zone 1	StdErr H5 - Zone 1	Mean H5 - Zone 2	StdErr H5 - Zone 2	Mean H6 - Zone 1	StdErr H6 - Zone 1	Mean H6 - Zone 2	StdErr H6 - Zone 2
Cur3	RMX180	Neutral	0.3	0.04	0.47	0.04	0.33	0.04	0.45	0.03	0.37	0.05	0.47	0.03	0.39	0.04	0.48	0.03	0.42	0.05	0.53	0.03	0.41	0.05	0.55	0.03	0.32	0.05	0.6	0.03

Analysis ratio halting to detection duration (H0 - Zone 1)

**Analysis model**	Linear mixed model fit by REML: Ratio_halting_to_detection_duration_H0_Zone_1 ~ 1 + (1|Genotype_Zone_1:Plant_Zone_1) + (1|Genotype_Zone_2:Plant_Zone_2)
**Transformation**	Natural logarithm

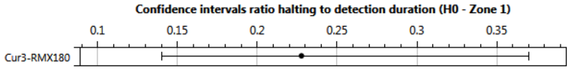

Confidence intervals ratio halting to detection duration (H0 - Zone 1)

Genotype Zone 1	Genotype Zone 2	Mean	Lower 95% CL	Upper 95% CL	Group
Cur3	RMX180	0.2276	0.14	0.3702	a

Model summary

```
Linear mixed model fit by REML. t-tests use Satterthwaite's method ['lmerModLmerTest']
Formula: Ratio_halting_to_detection_duration_H0_Zone_1 ~ 1 + (1 | Genotype_Zone_1:Plant_Zone_1) +      (1
| Genotype_Zone_2:Plant_Zone_2)
   Data: data

REML criterion at convergence: 91.2

Scaled residuals: 
    Min      1Q  Median      3Q     Max 
-2.1410 -0.6733  0.2738  0.7037  1.1571 

Random effects:
 Groups                       Name        Variance Std.Dev.
 Genotype_Zone_1:Plant_Zone_1 (Intercept) 0.2560   0.5059  
 Genotype_Zone_2:Plant_Zone_2 (Intercept) 0.0000   0.0000  
 Residual                                 0.6647   0.8153  
Number of obs: 34, groups:  Genotype_Zone_1:Plant_Zone_1, 10; Genotype_Zone_2:Plant_Zone_2, 10

Fixed effects:
            Estimate Std. Error      df t value Pr(>|t|)    
(Intercept)  -1.4800     0.2136  8.6311  -6.929 8.39e-05 ***
---
Signif. codes:  0 '***' 0.001 '**' 0.01 '*' 0.05 '.' 0.1 ' ' 1
```

Model residuals

Statistic	Value
Sample skewness	-0.711
Sample excess kurtosis	-0.4544
Passed Shapiro Wilk normality test	No (p-value = 0.0225 < 0.05)

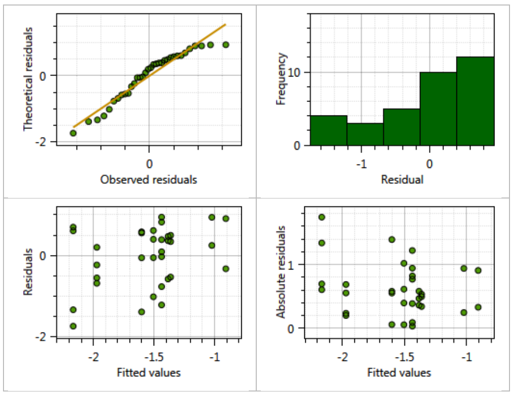

Analysis ratio halting to detection duration (H0 - Zone 2)

**Analysis model**	Linear mixed model fit by REML: Ratio_halting_to_detection_duration_H0_Zone_2 ~ 1 + (1|Genotype_Zone_1:Plant_Zone_1) + (1|Genotype_Zone_2:Plant_Zone_2)
**Transformation**	Natural logarithm

Confidence intervals ratio halting to detection duration (H0 - Zone 2)

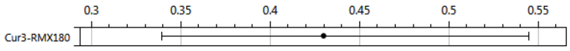

Confidence intervals ratio halting to detection duration (H0 - Zone 2)

Genotype Zone 1	Genotype Zone 2	Mean	Lower 95% CL	Upper 95% CL	Group
Cur3	RMX180	0.4298	0.3391	0.5449	a

Model summary

```
Linear mixed model fit by REML. t-tests use Satterthwaite's method ['lmerModLmerTest']
Formula: Ratio_halting_to_detection_duration_H0_Zone_2 ~ 1 + (1 | Genotype_Zone_1:Plant_Zone_1) +      (1
| Genotype_Zone_2:Plant_Zone_2)
   Data: data

REML criterion at convergence: 59.7

Scaled residuals: 
    Min      1Q  Median      3Q     Max 
-3.7402 -0.3629  0.0645  0.4772  1.5841 

Random effects:
 Groups                       Name        Variance Std.Dev.
 Genotype_Zone_1:Plant_Zone_1 (Intercept) 0.01627  0.12755 
 Genotype_Zone_2:Plant_Zone_2 (Intercept) 0.00127  0.03564 
 Residual                                 0.25117  0.50117 
Number of obs: 38, groups:  Genotype_Zone_1:Plant_Zone_1, 10; Genotype_Zone_2:Plant_Zone_2, 10

Fixed effects:
            Estimate Std. Error       df t value Pr(>|t|)    
(Intercept) -0.84434    0.09154  4.87694  -9.223 0.000287 ***
---
Signif. codes:  0 '***' 0.001 '**' 0.01 '*' 0.05 '.' 0.1 ' ' 1
```

Model residuals

Statistic	Value
Sample skewness	-1.613
Sample excess kurtosis	5.363
Passed Shapiro Wilk normality test	No (p-value = 0.0004495 < 0.05)

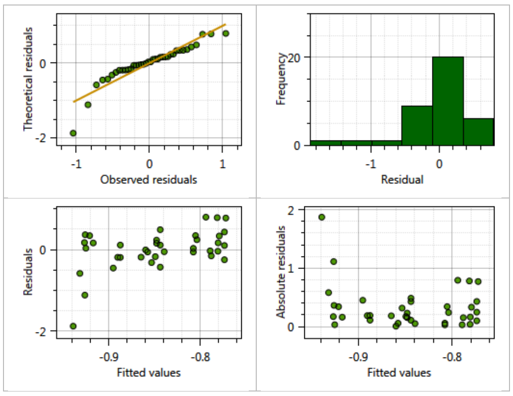

Analysis ratio halting to detection duration (H1 - Zone 1)

**Analysis model**	Linear mixed model fit by REML: Ratio_halting_to_detection_duration_H1_Zone_1 ~ 1 + (1|Genotype_Zone_1:Plant_Zone_1) + (1|Genotype_Zone_2:Plant_Zone_2)
**Transformation**	Natural logarithm

Confidence intervals ratio halting to detection duration (H1 - Zone 1)

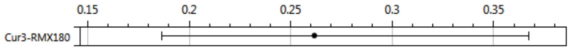

Confidence intervals ratio halting to detection duration (H1 - Zone 1)

Genotype Zone 1	Genotype Zone 2	Mean	Lower 95% CL	Upper 95% CL	Group
Cur3	RMX180	0.2616	0.1863	0.3674	a

Model summary

```
Linear mixed model fit by REML. t-tests use Satterthwaite's method ['lmerModLmerTest']
Formula: Ratio_halting_to_detection_duration_H1_Zone_1 ~ 1 + (1 | Genotype_Zone_1:Plant_Zone_1) +      (1
| Genotype_Zone_2:Plant_Zone_2)
   Data: data

REML criterion at convergence: 92

Scaled residuals: 
    Min      1Q  Median      3Q     Max 
-3.7203 -0.4818  0.0996  0.6134  1.4976 

Random effects:
 Groups                       Name        Variance Std.Dev.
 Genotype_Zone_1:Plant_Zone_1 (Intercept) 0.00000  0.0000  
 Genotype_Zone_2:Plant_Zone_2 (Intercept) 0.03162  0.1778  
 Residual                                 0.70403  0.8391  
Number of obs: 36, groups:  Genotype_Zone_1:Plant_Zone_1, 10; Genotype_Zone_2:Plant_Zone_2, 10

Fixed effects:
            Estimate Std. Error      df t value Pr(>|t|)    
(Intercept)  -1.3408     0.1509  9.3277  -8.886 7.46e-06 ***
---
Signif. codes:  0 '***' 0.001 '**' 0.01 '*' 0.05 '.' 0.1 ' ' 1
```

Model residuals

Statistic	Value
Sample skewness	-1.562
Sample excess kurtosis	4.654
Passed Shapiro Wilk normality test	No (p-value = 0.002481 < 0.05)

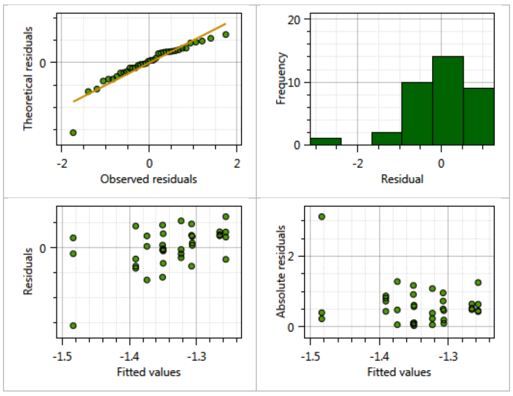

Analysis ratio halting to detection duration (H1 - Zone 2)

**Analysis model**	Linear mixed model fit by REML: Ratio_halting_to_detection_duration_H1_Zone_2 ~ 1 + (1|Genotype_Zone_1:Plant_Zone_1) + (1|Genotype_Zone_2:Plant_Zone_2)
**Transformation**	Natural logarithm

Confidence intervals ratio halting to detection duration (H1 - Zone 2)

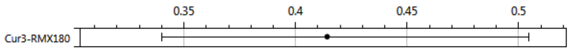

Confidence intervals ratio halting to detection duration (H1 - Zone 2)

Genotype Zone 1	Genotype Zone 2	Mean	Lower 95% CL	Upper 95% CL	Group
Cur3	RMX180	0.4142	0.3399	0.5047	a

Model summary

```
Linear mixed model fit by REML. t-tests use Satterthwaite's method ['lmerModLmerTest']
Formula: Ratio_halting_to_detection_duration_H1_Zone_2 ~ 1 + (1 | Genotype_Zone_1:Plant_Zone_1) +      (1
| Genotype_Zone_2:Plant_Zone_2)
   Data: data

REML criterion at convergence: 71

Scaled residuals: 
    Min      1Q  Median      3Q     Max 
-5.0400 -0.3281  0.1193  0.3704  1.4660 

Random effects:
 Groups                       Name        Variance  Std.Dev. 
 Genotype_Zone_1:Plant_Zone_1 (Intercept) 5.613e-23 7.492e-12
 Genotype_Zone_2:Plant_Zone_2 (Intercept) 0.000e+00 0.000e+00
 Residual                                 3.616e-01 6.013e-01
Number of obs: 38, groups:  Genotype_Zone_1:Plant_Zone_1, 10; Genotype_Zone_2:Plant_Zone_2, 10

Fixed effects:
            Estimate Std. Error       df t value Pr(>|t|)    
(Intercept) -0.88150    0.09754 37.00000  -9.037 6.73e-11 ***
---
Signif. codes:  0 '***' 0.001 '**' 0.01 '*' 0.05 '.' 0.1 ' ' 1
```

Model residuals

Statistic	Value
Sample skewness	-3.397
Sample excess kurtosis	17.71
Passed Shapiro Wilk normality test	No (p-value = 5.268E-08 < 0.05)

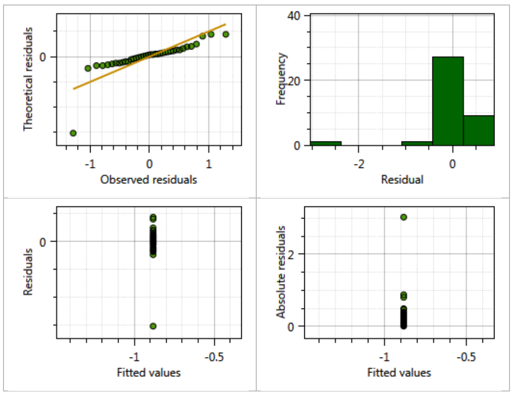

Analysis ratio halting to detection duration (H2 - Zone 1)

**Analysis model**	Linear mixed model fit by REML: Ratio_halting_to_detection_duration_H2_Zone_1 ~ 1 + (1|Genotype_Zone_1:Plant_Zone_1) + (1|Genotype_Zone_2:Plant_Zone_2)
**Transformation**	Natural logarithm

Confidence intervals ratio halting to detection duration (H2 - Zone 1)

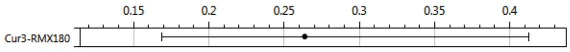

Confidence intervals ratio halting to detection duration (H2 - Zone 1)

Genotype Zone 1	Genotype Zone 2	Mean	Lower 95% CL	Upper 95% CL	Group
Cur3	RMX180	0.2637	0.1684	0.4128	a

Model summary

```
Linear mixed model fit by REML. t-tests use Satterthwaite's method ['lmerModLmerTest']
Formula: Ratio_halting_to_detection_duration_H2_Zone_1 ~ 1 + (1 | Genotype_Zone_1:Plant_Zone_1) +      (1
| Genotype_Zone_2:Plant_Zone_2)
   Data: data

REML criterion at convergence: 99

Scaled residuals: 
     Min       1Q   Median       3Q      Max 
-2.22115 -0.65939  0.05342  0.88391  1.57706 

Random effects:
 Groups                       Name        Variance Std.Dev.
 Genotype_Zone_1:Plant_Zone_1 (Intercept) 0.0000   0.0000  
 Genotype_Zone_2:Plant_Zone_2 (Intercept) 0.1836   0.4285  
```

```
 Residual                                 0.7631   0.8735
Number of obs: 36, groups:  Genotype_Zone_1:Plant_Zone_1, 10; Genotype_Zone_2:Plant_Zone_2, 10

Fixed effects:
            Estimate Std. Error      df t value Pr(>|t|)
(Intercept)  -1.3330     0.1994  9.3607  -6.687 7.47e-05 ***
---
Signif. codes:  0 '***' 0.001 '**' 0.01 '*' 0.05 '.' 0.1 ' ' 1
```

Model residuals

Statistic	Value
Sample skewness	-0.2995
Sample excess kurtosis	-0.7955
Passed Shapiro Wilk normality test	No (p-value = 0.03028 < 0.05)

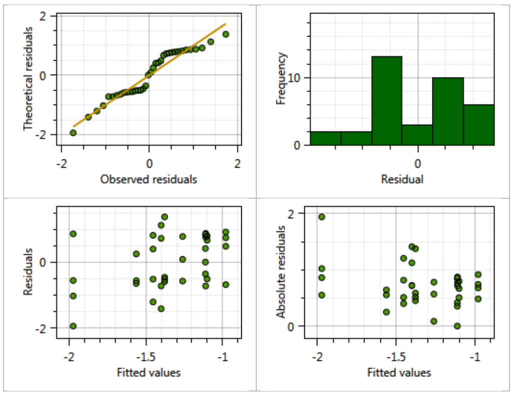

Analysis ratio halting to detection duration (H2 - Zone 2)

**Analysis model**	Linear mixed model fit by REML: Ratio_halting_to_detection_duration_H2_Zone_2 ~ 1 + (1|Genotype_Zone_1:Plant_Zone_1) + (1|Genotype_Zone_2:Plant_Zone_2)
**Transformation**	Natural logarithm

Confidence intervals ratio halting to detection duration (H2 - Zone 2)

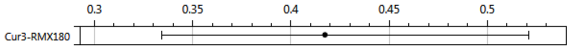

Confidence intervals ratio halting to detection duration (H2 - Zone 2)

Genotype Zone 1	Genotype Zone 2	Mean	Lower 95% CL	Upper 95% CL	Group
Cur3	RMX180	0.4173	0.334	0.5212	a

Model summary

```
Linear mixed model fit by REML. t-tests use Satterthwaite's method ['lmerModLmerTest']
Formula: Ratio_halting_to_detection_duration_H2_Zone_2 ~ 1 + (1 | Genotype_Zone_1:Plant_Zone_1) +      (1
| Genotype_Zone_2:Plant_Zone_2)
   Data: data

REML criterion at convergence: 79.8

Scaled residuals:
    Min      1Q  Median      3Q     Max
-3.4645 -0.1582  0.2203  0.5851  1.1841

Random effects:
 Groups                       Name        Variance Std.Dev.
 Genotype_Zone_1:Plant_Zone_1 (Intercept) 0.0000   0.0000
 Genotype_Zone_2:Plant_Zone_2 (Intercept) 0.0000   0.0000
 Residual                                 0.4581   0.6768
Number of obs: 38, groups:  Genotype_Zone_1:Plant_Zone_1, 10; Genotype_Zone_2:Plant_Zone_2, 10

Fixed effects:
            Estimate Std. Error      df t value Pr(>|t|)
(Intercept)  -0.8740     0.1098 37.0000  -7.961 1.55e-09 ***
---
Signif. codes:  0 '***' 0.001 '**' 0.01 '*' 0.05 '.' 0.1 ' ' 1
```

Model residuals

Statistic	Value
Sample skewness	-2.018
Sample excess kurtosis	4.125
Passed Shapiro Wilk normality test	No (p-value = 2.357E-06 < 0.05)

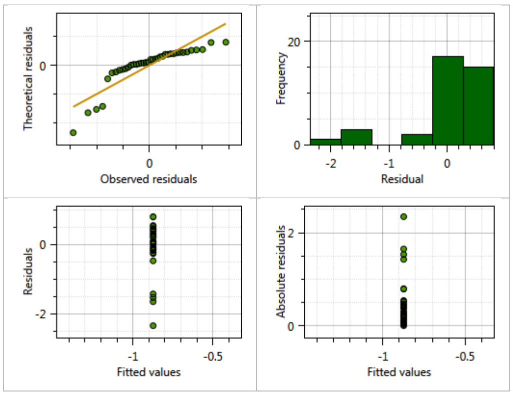

Analysis ratio halting to detection duration (H3 - Zone 1)

**Analysis model**	Linear mixed model fit by REML: Ratio_halting_to_detection_duration_H3_Zone_1 ~ 1 + (1|Genotype_Zone_1:Plant_Zone_1) + (1|Genotype_Zone_2:Plant_Zone_2)
**Transformation**	Natural logarithm

Confidence intervals ratio halting to detection duration (H3 - Zone 1)

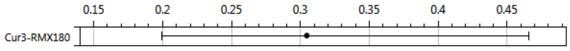

Confidence intervals ratio halting to detection duration (H3 - Zone 1)

Genotype Zone 1	Genotype Zone 2	Mean	Lower 95% CL	Upper 95% CL	Group
Cur3	RMX180	0.3046	0.1991	0.466	a

Model summary

```
Linear mixed model fit by REML. t-tests use Satterthwaite's method ['lmerModLmerTest']
Formula: Ratio_halting_to_detection_duration_H3_Zone_1 ~ 1 + (1 | Genotype_Zone_1:Plant_Zone_1) +      (1
| Genotype_Zone_2:Plant_Zone_2)
   Data: data

REML criterion at convergence: 96.5

Scaled residuals: 
    Min      1Q  Median      3Q     Max 
-3.2794 -0.2611  0.2530  0.6781  0.9783 

Random effects:
 Groups                       Name        Variance Std.Dev.
 Genotype_Zone_1:Plant_Zone_1 (Intercept) 0.000000 0.00000 
 Genotype_Zone_2:Plant_Zone_2 (Intercept) 0.009578 0.09787 
 Residual                                 1.062553 1.03080 
Number of obs: 33, groups:  Genotype_Zone_1:Plant_Zone_1, 10; Genotype_Zone_2:Plant_Zone_2, 10

Fixed effects:
            Estimate Std. Error      df t value Pr(>|t|)    
(Intercept)  -1.1886     0.1822  7.5052  -6.523 0.000243 ***
---
Signif. codes:  0 '***' 0.001 '**' 0.01 '*' 0.05 '.' 0.1 ' ' 1
```

Model residuals

Statistic	Value
Sample skewness	-1.902
Sample excess kurtosis	3.805
Passed Shapiro Wilk normality test	No (p-value = 2.92E-05 < 0.05)

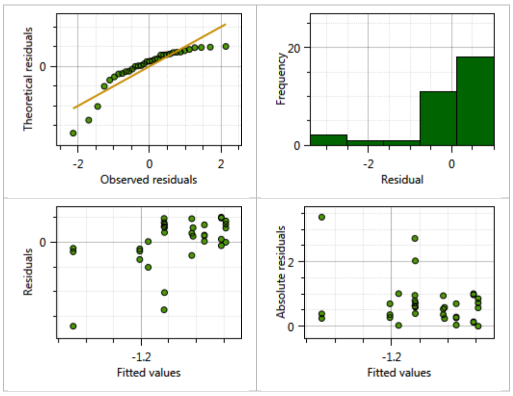

Analysis ratio halting to detection duration (H3 - Zone 2)

**Analysis model**	Linear mixed model fit by REML: Ratio_halting_to_detection_duration_H3_Zone_2 ~ 1 + (1|Genotype_Zone_1:Plant_Zone_1) + (1|Genotype_Zone_2:Plant_Zone_2)
**Transformation**	Natural logarithm

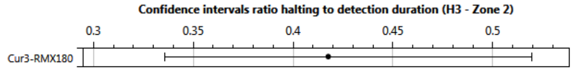

Confidence intervals ratio halting to detection duration (H3 - Zone 2)

Genotype Zone 1	Genotype Zone 2	Mean	Lower 95% CL	Upper 95% CL	Group
Cur3	RMX180	0.4176	0.3355	0.5197	a

Model summary

```
Linear mixed model fit by REML. t-tests use Satterthwaite's method ['lmerModLmerTest']
Formula: Ratio_halting_to_detection_duration_H3_Zone_2 ~ 1 + (1 | Genotype_Zone_1:Plant_Zone_1) +      (1
| Genotype_Zone_2:Plant_Zone_2)
   Data: data

REML criterion at convergence: 70.5

Scaled residuals: 
    Min      1Q  Median      3Q     Max 
-3.2928 -0.3904  0.1841  0.5709  1.3581 

Random effects:
 Groups                       Name        Variance Std.Dev.
 Genotype_Zone_1:Plant_Zone_1 (Intercept) 0.000000 0.00000 
 Genotype_Zone_2:Plant_Zone_2 (Intercept) 0.007702 0.08776 
 Residual                                 0.332865 0.57694 
Number of obs: 39, groups:  Genotype_Zone_1:Plant_Zone_1, 10; Genotype_Zone_2:Plant_Zone_2, 10

Fixed effects:
            Estimate Std. Error       df t value Pr(>|t|)    
(Intercept) -0.87328    0.09649  8.87856  -9.051 8.94e-06 ***
---
Signif. codes:  0 '***' 0.001 '**' 0.01 '*' 0.05 '.' 0.1 ' ' 1
```

Model residuals

Statistic	Value
Sample skewness	-1.461
Sample excess kurtosis	2.925
Passed Shapiro Wilk normality test	No (p-value = 0.0009034 < 0.05)

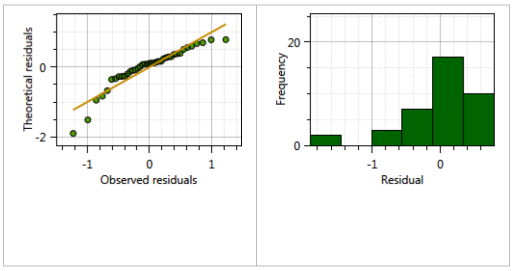

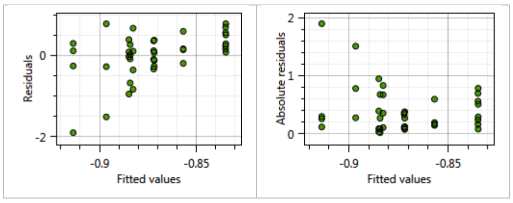

Analysis ratio halting to detection duration (H4 - Zone 1)

**Analysis model**	Linear mixed model fit by REML: Ratio_halting_to_detection_duration_H4_Zone_1 ~ 1 + (1|Genotype_Zone_1:Plant_Zone_1) + (1|Genotype_Zone_2:Plant_Zone_2)
**Transformation**	Natural logarithm

Confidence intervals ratio halting to detection duration (H4 - Zone 1)

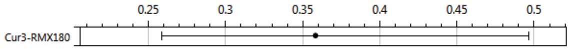

Confidence intervals ratio halting to detection duration (H4 - Zone 1)

Genotype Zone 1	Genotype Zone 2	Mean	Lower 95% CL	Upper 95% CL	Group
Cur3	RMX180	0.3583	0.2584	0.4967	a

Model summary

```
Linear mixed model fit by REML. t-tests use Satterthwaite's method ['lmerModLmerTest']
Formula: Ratio_halting_to_detection_duration_H4_Zone_1 ~ 1 + (1 | Genotype_Zone_1:Plant_Zone_1) +      (1
| Genotype_Zone_2:Plant_Zone_2)
   Data: data

REML criterion at convergence: 66.4

Scaled residuals: 
    Min      1Q  Median      3Q     Max 
-2.9378 -0.4891  0.1697  0.7103  1.2783 

Random effects:
 Groups                       Name        Variance Std.Dev.
 Genotype_Zone_1:Plant_Zone_1 (Intercept) 0.000000 0.00000 
 Genotype_Zone_2:Plant_Zone_2 (Intercept) 0.002378 0.04876 
 Residual                                 0.553081 0.74369 
Number of obs: 29, groups:  Genotype_Zone_1:Plant_Zone_1, 10; Genotype_Zone_2:Plant_Zone_2, 10

Fixed effects:
            Estimate Std. Error     df t value Pr(>|t|)    
(Intercept)   -1.026      0.139  7.229  -7.382 0.000129 ***
---
Signif. codes:  0 '***' 0.001 '**' 0.01 '*' 0.05 '.' 0.1 ' ' 1
```

Model residuals

Statistic	Value
Sample skewness	-1.007
Sample excess kurtosis	1.305
Passed Shapiro Wilk normality test	Yes (p-value = 0.06406 > 0.05)

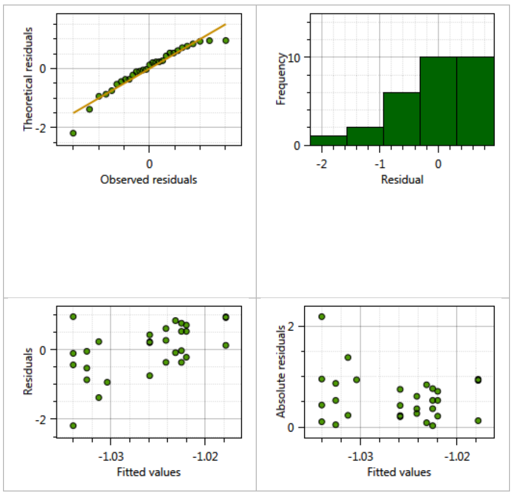

Analysis ratio halting to detection duration (H4 - Zone 2)

**Analysis model**	Linear mixed model fit by REML: Ratio_halting_to_detection_duration_H4_Zone_2 ~ 1 + (1|Genotype_Zone_1:Plant_Zone_1) + (1|Genotype_Zone_2:Plant_Zone_2)
**Transformation**	Natural logarithm

Confidence intervals ratio halting to detection duration (H4 - Zone 2)

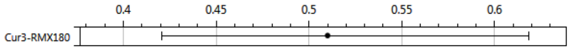

Confidence intervals ratio halting to detection duration (H4 - Zone 2)

Genotype Zone 1	Genotype Zone 2	Mean	Lower 95% CL	Upper 95% CL	Group
Cur3	RMX180	0.51	0.4205	0.6185	a

Model summary

```
Linear mixed model fit by REML. t-tests use Satterthwaite's method ['lmerModLmerTest']
Formula: Ratio_halting_to_detection_duration_H4_Zone_2 ~ 1 + (1 | Genotype_Zone_1:Plant_Zone_1) +      (1
| Genotype_Zone_2:Plant_Zone_2)
   Data: data

REML criterion at convergence: 43.5

Scaled residuals: 
     Min       1Q   Median       3Q      Max 
-2.87100 -0.37801  0.07416  0.49088  1.54358 

Random effects:
 Groups                       Name        Variance Std.Dev.
 Genotype_Zone_1:Plant_Zone_1 (Intercept) 0.00000  0.0000  
 Genotype_Zone_2:Plant_Zone_2 (Intercept) 0.02867  0.1693  
 Residual                                 0.15588  0.3948  
Number of obs: 37, groups:  Genotype_Zone_1:Plant_Zone_1, 10; Genotype_Zone_2:Plant_Zone_2, 10

Fixed effects:
            Estimate Std. Error      df t value Pr(>|t|)    
(Intercept)  -0.6733     0.0845  8.4857  -7.968 3.21e-05 ***
```

```
---
Signif. codes:  0 '***' 0.001 '**' 0.01 '*' 0.05 '.' 0.1 ' ' 1
```

Model residuals

Statistic	Value
Sample skewness	-1.017
Sample excess kurtosis	1.673
Passed Shapiro Wilk normality test	No (p-value = 0.02033 < 0.05)

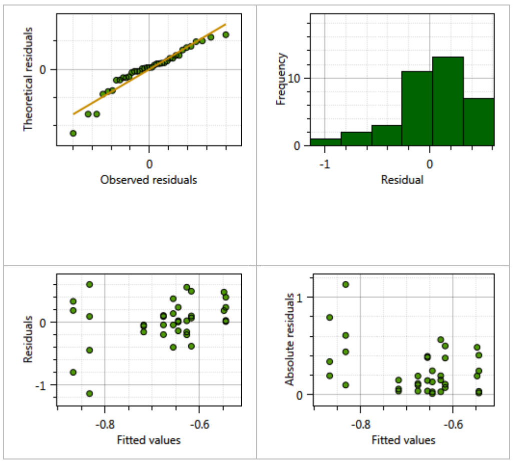

Analysis ratio halting to detection duration (H5 - Zone 1)

**Analysis model**	Linear mixed model fit by REML: Ratio_halting_to_detection_duration_H5_Zone_1 ~ 1 + (1|Genotype_Zone_1:Plant_Zone_1) + (1|Genotype_Zone_2:Plant_Zone_2)
**Transformation**	Natural logarithm

Confidence intervals ratio halting to detection duration (H5 - Zone 1)

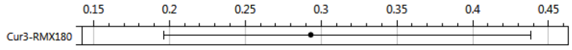

Confidence intervals ratio halting to detection duration (H5 - Zone 1)

Genotype Zone 1	Genotype Zone 2	Mean	Lower 95% CL	Upper 95% CL	Group
Cur3	RMX180	0.2931	0.196	0.4384	a

Model summary

```
Linear mixed model fit by REML. t-tests use Satterthwaite's method ['lmerModLmerTest']
Formula: Ratio_halting_to_detection_duration_H5_Zone_1 ~ 1 + (1 | Genotype_Zone_1:Plant_Zone_1) +      (1
| Genotype_Zone_2:Plant_Zone_2)
   Data: data

REML criterion at convergence: 94.1

Scaled residuals: 
    Min      1Q  Median      3Q     Max 
-2.4465 -0.2210  0.4302  0.7183  1.0520 

Random effects:
 Groups                       Name        Variance Std.Dev.
 Genotype_Zone_1:Plant_Zone_1 (Intercept) 0.000    0.000   
 Genotype_Zone_2:Plant_Zone_2 (Intercept) 0.000    0.000   
 Residual                                 1.204    1.097   
Number of obs: 31, groups:  Genotype_Zone_1:Plant_Zone_1, 10; Genotype_Zone_2:Plant_Zone_2, 10

Fixed effects:
            Estimate Std. Error      df t value Pr(>|t|)    
(Intercept)  -1.2271     0.1971 30.0000  -6.226 7.44e-07 ***
---
Signif. codes:  0 '***' 0.001 '**' 0.01 '*' 0.05 '.' 0.1 ' ' 1
```

Model residuals

Statistic	Value
Sample skewness	-1.209
Sample excess kurtosis	0.311
Passed Shapiro Wilk normality test	No (p-value = 0.0002069 < 0.05)

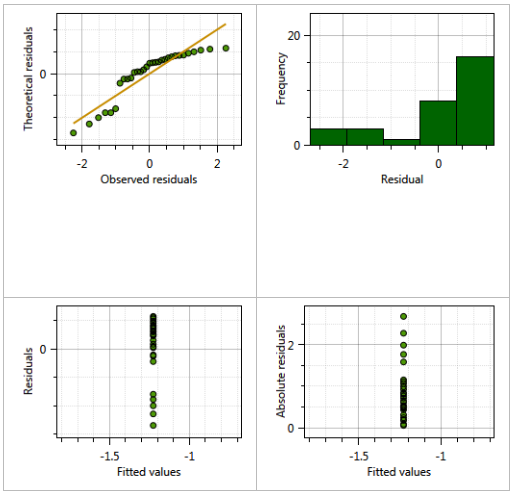

Analysis ratio halting to detection duration (H5 - Zone 2)

**Analysis model**	Linear mixed model fit by REML: Ratio_halting_to_detection_duration_H5_Zone_2 ~ 1 + (1|Genotype_Zone_1:Plant_Zone_1) + (1|Genotype_Zone_2:Plant_Zone_2)
**Transformation**	Natural logarithm

Confidence intervals ratio halting to detection duration (H5 - Zone 2)

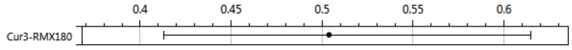

Confidence intervals ratio halting to detection duration (H5 - Zone 2)

Genotype Zone 1	Genotype Zone 2	Mean	Lower 95% CL	Upper 95% CL	Group
Cur3	RMX180	0.5038	0.4127	0.6148	a

Model summary

```
Linear mixed model fit by REML. t-tests use Satterthwaite's method ['lmerModLmerTest']
Formula: Ratio_halting_to_detection_duration_H5_Zone_2 ~ 1 + (1 | Genotype_Zone_1:Plant_Zone_1) +      (1
| Genotype_Zone_2:Plant_Zone_2)
   Data: data

REML criterion at convergence: 50.6

Scaled residuals: 
    Min      1Q  Median      3Q     Max 
-2.5663 -0.5054  0.1217  0.7476  1.3173 

Random effects:
 Groups                       Name        Variance Std.Dev.
 Genotype_Zone_1:Plant_Zone_1 (Intercept) 0.018039 0.1343  
 Genotype_Zone_2:Plant_Zone_2 (Intercept) 0.008557 0.0925  
 Residual                                 0.179245 0.4234  
Number of obs: 39, groups:  Genotype_Zone_1:Plant_Zone_1, 10; Genotype_Zone_2:Plant_Zone_2, 10

Fixed effects:
            Estimate Std. Error       df t value Pr(>|t|)    
(Intercept) -0.68567    0.08526  7.42788  -8.043 6.37e-05 ***
---
Signif. codes:  0 '***' 0.001 '**' 0.01 '*' 0.05 '.' 0.1 ' ' 1
```

Model residuals

Statistic	Value
Sample skewness	-0.7741
Sample excess kurtosis	-0.08685
Passed Shapiro Wilk normality test	No (p-value = 0.01742 < 0.05)

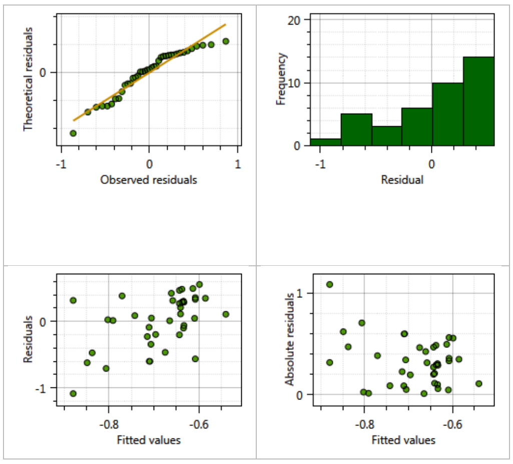

Analysis ratio halting to detection duration (H6 - Zone 1)

**Analysis model**	Linear mixed model fit by REML: Ratio_halting_to_detection_duration_H6_Zone_1 ~ 1 + (1|Genotype_Zone_1:Plant_Zone_1) + (1|Genotype_Zone_2:Plant_Zone_2)
**Transformation**	Natural logarithm

Confidence intervals ratio halting to detection duration (H6 - Zone 1)

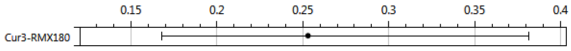

Confidence intervals ratio halting to detection duration (H6 - Zone 1)

Genotype Zone 1	Genotype Zone 2	Mean	Lower 95% CL	Upper 95% CL	Group
Cur3	RMX180	0.253	0.1677	0.3816	a

Model summary

```
Linear mixed model fit by REML. t-tests use Satterthwaite's method ['lmerModLmerTest']
Formula: Ratio_halting_to_detection_duration_H6_Zone_1 ~ 1 + (1 | Genotype_Zone_1:Plant_Zone_1) +      (1
| Genotype_Zone_2:Plant_Zone_2)
   Data: data

REML criterion at convergence: 67.6

Scaled residuals: 
     Min       1Q   Median       3Q      Max 
-1.99228 -0.56855  0.01287  0.81404  1.44288 

Random effects:
 Groups                       Name        Variance  Std.Dev. 
 Genotype_Zone_1:Plant_Zone_1 (Intercept) 4.547e-18 2.132e-09
 Genotype_Zone_2:Plant_Zone_2 (Intercept) 4.794e-02 2.189e-01
 Residual                                 6.542e-01 8.088e-01
Number of obs: 27, groups:  Genotype_Zone_1:Plant_Zone_1, 10; Genotype_Zone_2:Plant_Zone_2, 10

Fixed effects:
            Estimate Std. Error      df t value Pr(>|t|)    
(Intercept)  -1.3745     0.1724  6.7149  -7.972 0.000116 ***
---
Signif. codes:  0 '***' 0.001 '**' 0.01 '*' 0.05 '.' 0.1 ' ' 1
```

Model residuals

Statistic	Value
Sample skewness	-0.2385
Sample excess kurtosis	-0.6454
Passed Shapiro Wilk normality test	Yes (p-value = 0.3345 > 0.05)

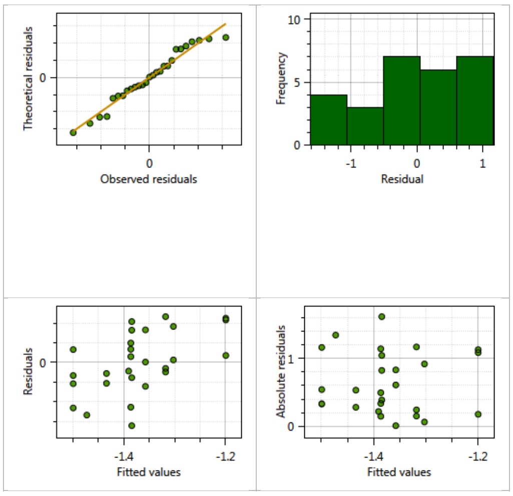

Analysis ratio halting to detection duration (H6 - Zone 2)

**Analysis model**	Linear mixed model fit by REML: Ratio_halting_to_detection_duration_H6_Zone_2 ~ 1 + (1|Genotype_Zone_1:Plant_Zone_1) + (1|Genotype_Zone_2:Plant_Zone_2)
**Transformation**	Natural logarithm

Confidence intervals ratio halting to detection duration (H6 - Zone 2)

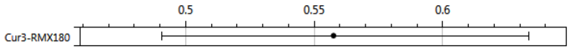

Confidence intervals ratio halting to detection duration (H6 - Zone 2)

Genotype Zone 1	Genotype Zone 2	Mean	Lower 95% CL	Upper 95% CL	Group
Cur3	RMX180	0.5575	0.4906	0.6336	a

Model summary

```
Linear mixed model fit by REML. t-tests use Satterthwaite's method ['lmerModLmerTest']
Formula: Ratio_halting_to_detection_duration_H6_Zone_2 ~ 1 + (1 | Genotype_Zone_1:Plant_Zone_1) +      (1
| Genotype_Zone_2:Plant_Zone_2)
   Data: data

REML criterion at convergence: 38.8

Scaled residuals:
    Min      1Q  Median      3Q     Max
-2.5081 -0.3713  0.1235  0.8543  1.4489

Random effects:
 Groups                       Name        Variance Std.Dev.
 Genotype_Zone_1:Plant_Zone_1 (Intercept) 0.0000   0.0000
 Genotype_Zone_2:Plant_Zone_2 (Intercept) 0.0000   0.0000
 Residual                                 0.1516   0.3893
Number of obs: 38, groups:  Genotype_Zone_1:Plant_Zone_1, 10; Genotype_Zone_2:Plant_Zone_2, 10

Fixed effects:
            Estimate Std. Error       df t value Pr(>|t|)
(Intercept) -0.58426    0.06315 37.00000  -9.252 3.66e-11 ***
---
Signif. codes:  0 '***' 0.001 '**' 0.01 '*' 0.05 '.' 0.1 ' ' 1
```

Model residuals

Statistic	Value
Sample skewness	-0.7234
Sample excess kurtosis	0.07446
Passed Shapiro Wilk normality test	Yes (p-value = 0.05615 > 0.05)

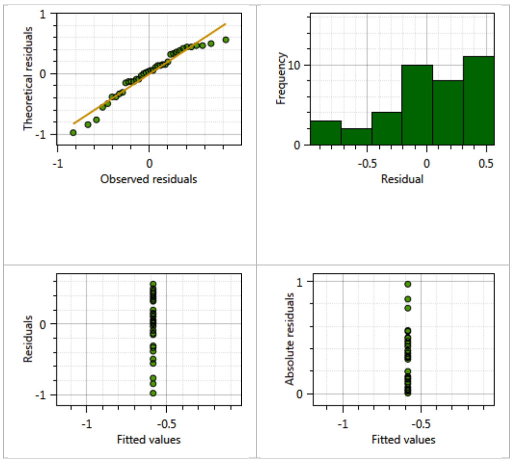

Analysis ratio halting to detection duration (H7 - Zone 1)

**Analysis model**	Linear mixed model fit by REML: Ratio_halting_to_detection_duration_H7_Zone_1 ~ 1 + (1|Genotype_Zone_1:Plant_Zone_1) + (1|Genotype_Zone_2:Plant_Zone_2)
**Transformation**	Natural logarithm

Confidence intervals ratio halting to detection duration (H7 - Zone 1)

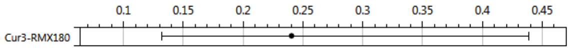

Confidence intervals ratio halting to detection duration (H7 - Zone 1)

Genotype Zone 1	Genotype Zone 2	Mean	Lower 95% CL	Upper 95% CL	Group
Cur3	RMX180	0.2404	0.1316	0.4389	a

Model summary

```
Linear mixed model fit by REML. t-tests use Satterthwaite's method ['lmerModLmerTest']
Formula: Ratio_halting_to_detection_duration_H7_Zone_1 ~ 1 + (1 | Genotype_Zone_1:Plant_Zone_1) +      (1
| Genotype_Zone_2:Plant_Zone_2)
   Data: data
```

```
REML criterion at convergence: 78.4

Scaled residuals:
    Min      1Q  Median      3Q     Max
-2.2721 -0.2587  0.3612  0.6101  1.0102

Random effects:
 Groups                      Name        Variance Std.Dev.
 Genotype_Zone_2:Plant_Zone_2 (Intercept) 0.21809  0.4670
 Genotype_Zone_1:Plant_Zone_1 (Intercept) 0.05644  0.2376
 Residual                                 0.84488  0.9192
Number of obs: 27, groups:  Genotype_Zone_2:Plant_Zone_2, 10; Genotype_Zone_1:Plant_Zone_1, 9

Fixed effects:
            Estimate Std. Error      df t value Pr(>|t|)
(Intercept)  -1.4255     0.2506  6.4918  -5.688  0.00097 ***
---
Signif. codes:  0 '***' 0.001 '**' 0.01 '*' 0.05 '.' 0.1 ' ' 1
```

Model residuals

Statistic	Value
Sample skewness	-1.308
Sample excess kurtosis	0.9146
Passed Shapiro Wilk normality test	No (p-value = 0.0007432 < 0.05)

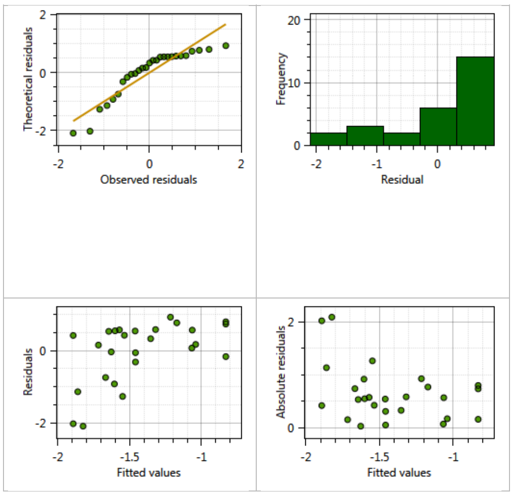

Analysis ratio halting to detection duration (H7 - Zone 2)

**Analysis model**	Linear mixed model fit by REML: Ratio_halting_to_detection_duration_H7_Zone_2 ~ 1 + (1|Genotype_Zone_1:Plant_Zone_1) + (1|Genotype_Zone_2:Plant_Zone_2)
**Transformation**	Natural logarithm

Confidence intervals ratio halting to detection duration (H7 - Zone 2)

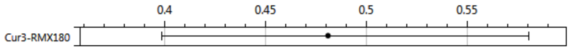

Confidence intervals ratio halting to detection duration (H7 - Zone 2)

Genotype Zone 1	Genotype Zone 2	Mean	Lower 95% CL	Upper 95% CL	Group
Cur3	RMX180	0.481	0.3984	0.5808	a

Model summary

```
Linear mixed model fit by REML. t-tests use Satterthwaite's method ['lmerModLmerTest']
Formula: Ratio_halting_to_detection_duration_H7_Zone_2 ~ 1 + (1 | Genotype_Zone_1:Plant_Zone_1) +      (1
| Genotype_Zone_2:Plant_Zone_2)
   Data: data

REML criterion at convergence: 70.3

Scaled residuals:
    Min      1Q  Median      3Q     Max
-2.3880 -0.4519  0.3801  0.6571  1.2063

Random effects:
 Groups                      Name        Variance Std.Dev.
 Genotype_Zone_1:Plant_Zone_1 (Intercept) 0.0000   0.0000
 Genotype_Zone_2:Plant_Zone_2 (Intercept) 0.0000   0.0000
 Residual                                 0.3381   0.5814
Number of obs: 39, groups:  Genotype_Zone_1:Plant_Zone_1, 10; Genotype_Zone_2:Plant_Zone_2, 10

Fixed effects:
            Estimate Std. Error      df t value Pr(>|t|)
(Intercept)  -0.7318     0.0931 38.0000  -7.861 1.74e-09 ***
---
Signif. codes:  0 '***' 0.001 '**' 0.01 '*' 0.05 '.' 0.1 ' ' 1
```

Model residuals

Statistic	Value
Sample skewness	-1.059
Sample excess kurtosis	0.1837
Passed Shapiro Wilk normality test	No (p-value = 0.0006131 < 0.05)

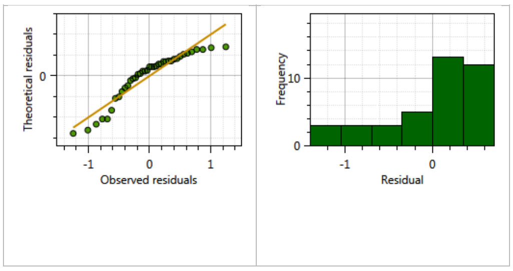

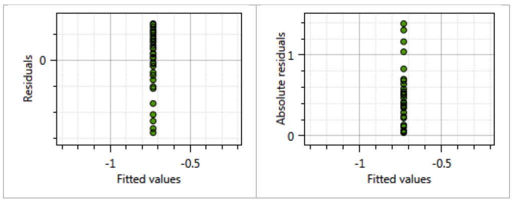

Analysis ratio halting to detection duration H0 (diff. Zone 1 - Zone 2)

**Analysis model**	Generalized linear mixed model with dispersion factor, formula=cbind(Ratio_halting_to_detection_duration_H0_Zone_1,Ratio_halting_to_detection_duration_H0_Zone_2) ~ 1 + (1|Genotype_Zone_1:Plant_Zone_1) + (1|Genotype_Zone_2:Plant_Zone_2)
**Transformation**	Logit

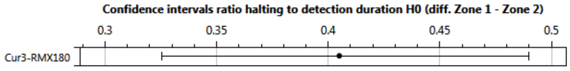

Confidence intervals ratio halting to detection duration H0 (diff. Zone 1 - Zone 2)

Genotype Zone 1	Genotype Zone 2	Mean	Lower 95% CL	Upper 95% CL	Group
Cur3	RMX180	0.4049	0.3253	0.49	a

Model summary

```
Linear mixed model fit by REML. t-tests use Satterthwaite's method ['lmerModLmerTest']
Formula: ziFormula
   Data: data
Weights: wi

REML criterion at convergence: 91.3

Scaled residuals:
    Min      1Q  Median      3Q     Max
-1.7277 -0.8362 -0.2580  0.4439  2.0717

Random effects:
 Groups                      Name        Variance Std.Dev.
 Genotype_Zone_1:Plant_Zone_1 (Intercept) 0.04848  0.2202
 Genotype_Zone_2:Plant_Zone_2 (Intercept) 0.00000  0.0000
 Residual                                0.11612  0.3408
Number of obs: 35, groups:  Genotype_Zone_1:Plant_Zone_1, 10; Genotype_Zone_2:Plant_Zone_2, 10

Fixed effects:
            Estimate Std. Error      df t value Pr(>|t|)
(Intercept)  -0.3849     0.1540  9.6380    -2.5   0.0322 *
---
Signif. codes:  0 '***' 0.001 '**' 0.01 '*' 0.05 '.' 0.1 ' ' 1

Dispersion: 0.3408
```

Model residuals

Statistic	Value
Sample skewness	0.5899
Sample excess kurtosis	-0.09388
Passed Shapiro Wilk normality test	Yes (p-value = 0.14 > 0.05)

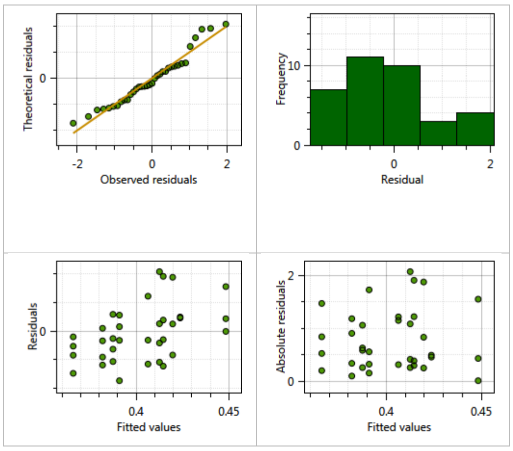

Analysis ratio halting to detection duration H1 (diff. Zone 1 - Zone 2)

**Analysis model**	Generalized linear mixed model with dispersion factor, formula=cbind(Ratio_halting_to_detection_duration_H1_Zone_1,Ratio_halting_to_detection_duration_H1_Zone_2) ~ 1 + (1|Genotype_Zone_1:Plant_Zone_1) + (1|Genotype_Zone_2:Plant_Zone_2)
**Transformation**	Logit

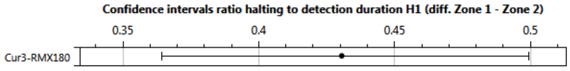

Confidence intervals ratio halting to detection duration H1 (diff. Zone 1 - Zone 2)

Genotype Zone 1	Genotype Zone 2	Mean	Lower 95% CL	Upper 95% CL	Group
Cur3	RMX180	0.4305	0.3642	0.4995	a

Model summary

```
Linear mixed model fit by REML. t-tests use Satterthwaite's method ['lmerModLmerTest']
Formula: ziFormula
   Data: data
Weights: wi

REML criterion at convergence: 94.8

Scaled residuals:
    Min      1Q  Median      3Q     Max
-1.8258 -0.8547 -0.1824  0.4755  2.7434

Random effects:
 Groups                      Name        Variance Std.Dev.
 Genotype_Zone_1:Plant_Zone_1 (Intercept) 0.0000   0.0000
 Genotype_Zone_2:Plant_Zone_2 (Intercept) 0.0000   0.0000
 Residual                                0.1303   0.3609
Number of obs: 37, groups:  Genotype_Zone_1:Plant_Zone_1, 10; Genotype_Zone_2:Plant_Zone_2, 10

Fixed effects:
            Estimate Std. Error      df t value Pr(>|t|)
```

```
(Intercept)  -0.2797     0.1369 36.0000  -2.043   0.0484 *
---
Signif. codes:  0 '***' 0.001 '**' 0.01 '*' 0.05 '.' 0.1 ' ' 1

Dispersion: 0.3609
```

Model residuals

Statistic	Value
Sample skewness	0.8261
Sample excess kurtosis	0.7497
Passed Shapiro Wilk normality test	Yes (p-value = 0.1452 > 0.05)

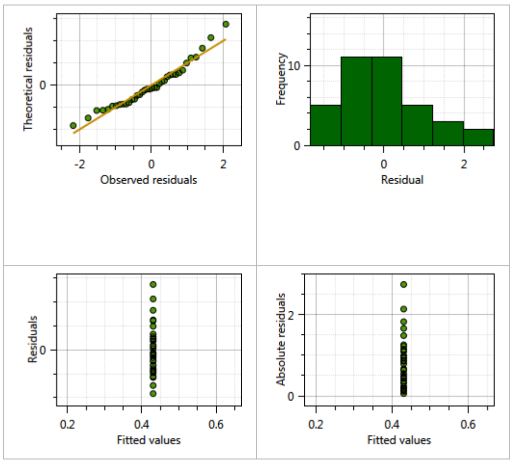

Analysis ratio halting to detection duration H2 (diff. Zone 1 - Zone 2)

**Analysis model**	Generalized linear mixed model with dispersion factor, formula=cbind(Ratio_halting_to_detection_duration_H2_Zone_1,Ratio_halting_to_detection_duration_H2_Zone_2) ~ 1 + (1|Genotype_Zone_1:Plant_Zone_1) + (1|Genotype_Zone_2:Plant_Zone_2)
**Transformation**	Logit

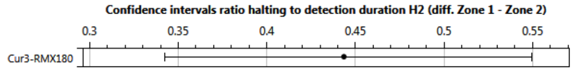

Confidence intervals ratio halting to detection duration H2 (diff. Zone 1 - Zone 2)

Genotype Zone 1	Genotype Zone 2	Mean	Lower 95% CL	Upper 95% CL	Group
Cur3	RMX180	0.4435	0.3422	0.5496	a

Model summary

```
Linear mixed model fit by REML. t-tests use Satterthwaite's method ['lmerModLmerTest']
Formula: ziFormula
   Data: data
Weights: wi

REML criterion at convergence: 99.7

Scaled residuals:
    Min      1Q  Median      3Q     Max
-1.4851 -0.8542 -0.1670  0.4800  2.4158

Random effects:
 Groups                      Name        Variance Std.Dev.
 Genotype_Zone_1:Plant_Zone_1 (Intercept) 0.1100   0.3317
 Genotype_Zone_2:Plant_Zone_2 (Intercept) 0.0277   0.1664
 Residual                                0.1272   0.3566
Number of obs: 37, groups:  Genotype_Zone_1:Plant_Zone_1, 10; Genotype_Zone_2:Plant_Zone_2, 10

Fixed effects:
            Estimate Std. Error      df t value Pr(>|t|)
(Intercept)  -0.2271     0.1769  6.3983  -1.284    0.244

Dispersion: 0.3566
```

Model residuals

Statistic	Value
Sample skewness	0.7077
Sample excess kurtosis	0.3479
Passed Shapiro Wilk normality test	Yes (p-value = 0.1315 > 0.05)

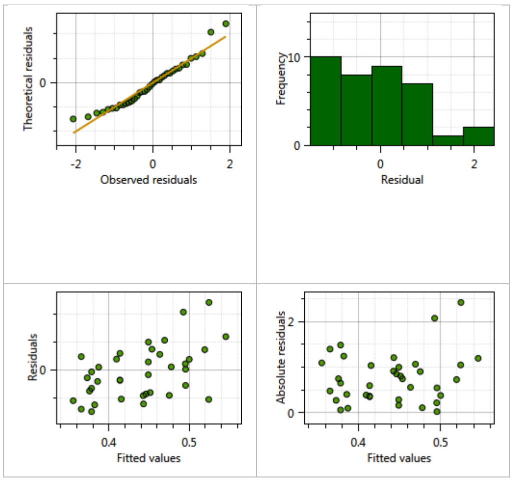

Analysis ratio halting to detection duration H3 (diff. Zone 1 - Zone 2)

**Analysis model**	Generalized linear mixed model with dispersion factor, formula=cbind(Ratio_halting_to_detection_duration_H3_Zone_1,Ratio_halting_to_detection_duration_H3_Zone_2) ~ 1 + (1|Genotype_Zone_1:Plant_Zone_1) + (1|Genotype_Zone_2:Plant_Zone_2)
**Transformation**	Logit

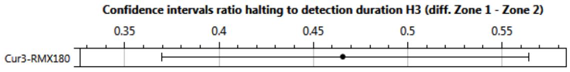

Confidence intervals ratio halting to detection duration H3 (diff. Zone 1 - Zone 2)

Genotype Zone 1	Genotype Zone 2	Mean	Lower 95% CL	Upper 95% CL	Group
Cur3	RMX180	0.4656	0.3695	0.5643	a

Model summary

```
Linear mixed model fit by REML. t-tests use Satterthwaite's method ['lmerModLmerTest']
Formula: ziFormula
   Data: data
Weights: wi

REML criterion at convergence: 87.9

Scaled residuals: 
     Min       1Q   Median       3Q      Max 
-1.94141 -0.43274  0.09906  0.49703  1.96665 

Random effects:
 Groups                        Name        Variance Std.Dev.
 Genotype_Zone_1:Plant_Zone_1 (Intercept) 0.0000   0.0000  
 Genotype_Zone_2:Plant_Zone_2 (Intercept) 0.1014   0.3184  
 Residual                                 0.1165   0.3413  
Number of obs: 35, groups:  Genotype_Zone_1:Plant_Zone_1, 10; Genotype_Zone_2:Plant_Zone_2, 10

Fixed effects:
            Estimate Std. Error      df t value Pr(>|t|)
(Intercept)  -0.1379     0.1645  6.3913  -0.838    0.432

Dispersion: 0.3413
```

Model residuals

Statistic	Value
Sample skewness	-0.2433
Sample excess kurtosis	-0.07021
Passed Shapiro Wilk normality test	Yes (p-value = 0.373 > 0.05)

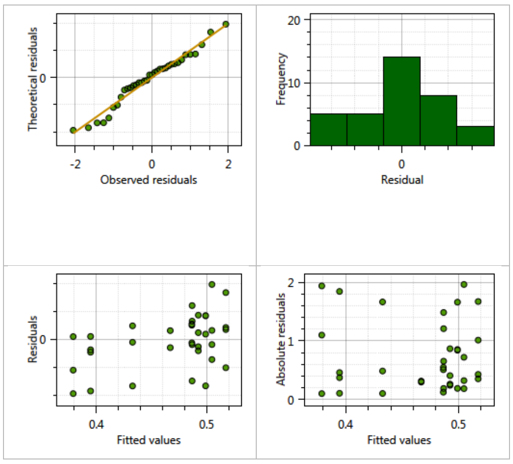

Analysis ratio halting to detection duration H4 (diff. Zone 1 - Zone 2)

**Analysis model**	Generalized linear mixed model with dispersion factor, formula=cbind(Ratio_halting_to_detection_duration_H4_Zone_1,Ratio_halting_to_detection_duration_H4_Zone_2) ~ 1 + (1|Genotype_Zone_1:Plant_Zone_1) + (1|Genotype_Zone_2:Plant_Zone_2)
**Transformation**	Logit

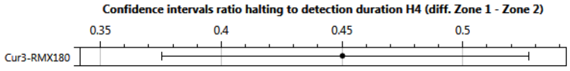

Confidence intervals ratio halting to detection duration H4 (diff. Zone 1 - Zone 2)

Genotype Zone 1	Genotype Zone 2	Mean	Lower 95% CL	Upper 95% CL	Group
Cur3	RMX180	0.4502	0.3752	0.5274	a

Model summary

```
Linear mixed model fit by REML. t-tests use Satterthwaite's method ['lmerModLmerTest']
Formula: ziFormula
   Data: data
Weights: wi

REML criterion at convergence: 76.6

Scaled residuals: 
    Min      1Q  Median      3Q     Max 
-1.9057 -0.5388 -0.2574  0.4896  2.7274 

Random effects:
 Groups                        Name        Variance Std.Dev.
 Genotype_Zone_1:Plant_Zone_1 (Intercept) 0.0000   0.0000  
 Genotype_Zone_2:Plant_Zone_2 (Intercept) 0.0000   0.0000  
 Residual                                 0.1511   0.3887  
Number of obs: 30, groups:  Genotype_Zone_1:Plant_Zone_1, 10; Genotype_Zone_2:Plant_Zone_2, 10

Fixed effects:
            Estimate Std. Error      df t value Pr(>|t|)
(Intercept)  -0.2000     0.1515 29.0000   -1.32    0.197

Dispersion: 0.3887
```

Model residuals

Statistic	Value
Sample skewness	0.6124
Sample excess kurtosis	0.9307
Passed Shapiro Wilk normality test	Yes (p-value = 0.5387 > 0.05)

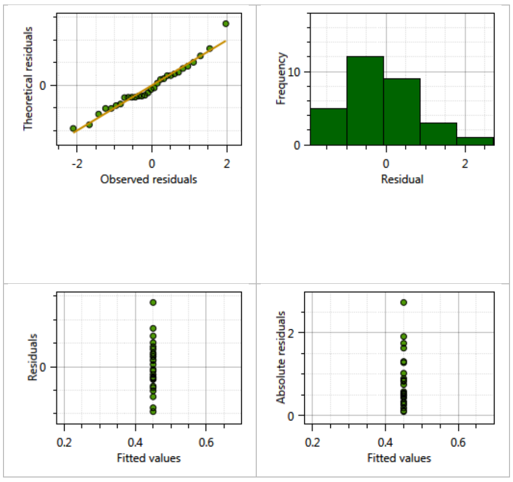

Analysis ratio halting to detection duration H5 (diff. Zone 1 - Zone 2)

**Analysis model**	Generalized linear mixed model with dispersion factor, formula=cbind(Ratio_halting_to_detection_duration_H5_Zone_1,Ratio_halting_to_detection_duration_H5_Zone_2) ~ 1 + (1|Genotype_Zone_1:Plant_Zone_1) + (1|Genotype_Zone_2:Plant_Zone_2)
**Transformation**	Logit

Confidence intervals ratio halting to detection duration H5 (diff. Zone 1 - Zone 2)

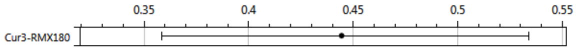

Confidence intervals ratio halting to detection duration H5 (diff. Zone 1 - Zone 2)

Genotype Zone 1	Genotype Zone 2	Mean	Lower 95% CL	Upper 95% CL	Group
Cur3	RMX180	0.4444	0.3583	0.534	a

Model summary

```
Linear mixed model fit by REML. t-tests use Satterthwaite's method ['lmerModLmerTest']
Formula: ziFormula
   Data: data
Weights: wi

REML criterion at convergence: 84.4

Scaled residuals: 
     Min       1Q   Median       3Q      Max 
-1.95774 -0.63602  0.05103  0.62301  1.38105 

Random effects:
 Groups                      Name        Variance  Std.Dev.
 Genotype_Zone_1:Plant_Zone_1 (Intercept) 3.487e-02 1.867e-01
 Genotype_Zone_2:Plant_Zone_2 (Intercept) 1.605e-17 4.006e-09
 Residual                                 1.659e-01 4.073e-01
Number of obs: 32, groups:  Genotype_Zone_1:Plant_Zone_1, 10; Genotype_Zone_2:Plant_Zone_2, 10

Fixed effects:
            Estimate Std. Error      df t value Pr(>|t|)
(Intercept)  -0.2232     0.1623 10.4459  -1.375    0.198

Dispersion: 0.4073
```

Model residuals

Statistic	Value
Sample skewness	-0.3232
Sample excess kurtosis	-0.8033
Passed Shapiro Wilk normality test	Yes (p-value = 0.1941 > 0.05)

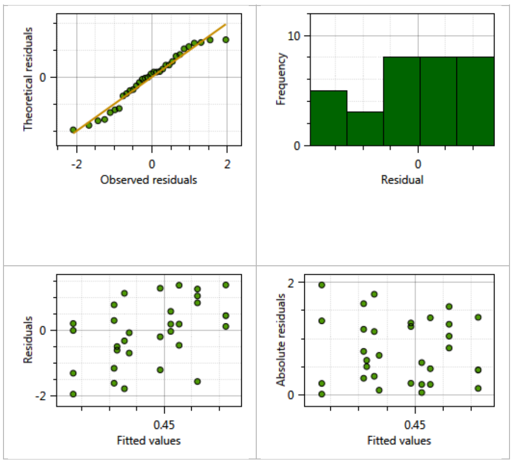

Analysis ratio halting to detection duration H6 (diff. Zone 1 - Zone 2)

**Analysis model**	Generalized linear mixed model with dispersion factor, formula=cbind(Ratio_halting_to_detection_duration_H6_Zone_1,Ratio_halting_to_detection_duration_H6_Zone_2) ~ 1 + (1|Genotype_Zone_1:Plant_Zone_1) + (1|Genotype_Zone_2:Plant_Zone_2)
**Transformation**	Logit

Confidence intervals ratio halting to detection duration H6 (diff. Zone 1 - Zone 2)

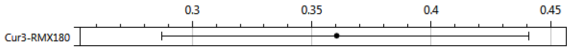

Confidence intervals ratio halting to detection duration H6 (diff. Zone 1 - Zone 2)

Genotype Zone 1	Genotype Zone 2	Mean	Lower 95% CL	Upper 95% CL	Group
Cur3	RMX180	0.3604	0.287	0.441	a

Model summary

```
Linear mixed model fit by REML. t-tests use Satterthwaite's method ['lmerModLmerTest']
Formula: ziFormula
   Data: data
```

```
Weights: wi

REML criterion at convergence: 74

Scaled residuals:
    Min      1Q  Median      3Q     Max
-1.5898 -0.6364 -0.2858  0.8025  2.1887

Random effects:
 Groups                       Name        Variance  Std.Dev.
 Genotype_Zone_1:Plant_Zone_1 (Intercept) 4.017e-16 2.004e-08
 Genotype_Zone_2:Plant_Zone_2 (Intercept) 0.000e+00 0.000e+00
 Residual                                 1.449e-01 3.807e-01
Number of obs: 28, groups:  Genotype_Zone_1:Plant_Zone_1, 10; Genotype_Zone_2:Plant_Zone_2, 10

Fixed effects:
            Estimate Std. Error      df t value Pr(>|t|)
(Intercept)  -0.5736     0.1639 27.0000  -3.499  0.00164 **
---
Signif. codes:  0 '***' 0.001 '**' 0.01 '*' 0.05 '.' 0.1 ' ' 1

Dispersion: 0.3807
```

Model residuals

Statistic	Value
Sample skewness	0.4698
Sample excess kurtosis	-0.3719
Passed Shapiro Wilk normality test	Yes (p-value = 0.3533 > 0.05)

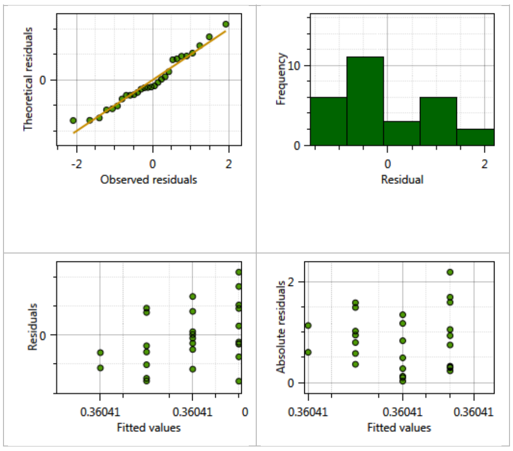

Analysis ratio halting to detection duration H7 (diff. Zone 1 - Zone 2)

**Analysis model**	Generalized linear mixed model with dispersion factor, formula=cbind(Ratio_halting_to_detection_duration_H7_Zone_1,Ratio_halting_to_detection_duration_H7_Zone_2) ~ 1 + (1|Genotype_Zone_1:Plant_Zone_1) + (1|Genotype_Zone_2:Plant_Zone_2)
**Transformation**	Logit

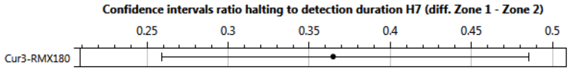

Confidence intervals ratio halting to detection duration H7 (diff. Zone 1 - Zone 2)

Genotype Zone 1	Genotype Zone 2	Mean	Lower 95% CL	Upper 95% CL	Group
Cur3	RMX180	0.3648	0.2588	0.4856	a

Model summary

```
Linear mixed model fit by REML. t-tests use Satterthwaite's method ['lmerModLmerTest']
Formula: ziFormula
   Data: data
Weights: wi

REML criterion at convergence: 85.1

Scaled residuals:
    Min      1Q  Median      3Q     Max
-1.4128 -0.7734 -0.1050  0.6669  1.5803

Random effects:
 Groups                       Name        Variance Std.Dev.
 Genotype_Zone_2:Plant_Zone_2 (Intercept) 0.2225   0.4717
 Genotype_Zone_1:Plant_Zone_1 (Intercept) 0.0000   0.0000
 Residual                                 0.1336   0.3655
Number of obs: 30, groups:  Genotype_Zone_2:Plant_Zone_2, 10; Genotype_Zone_1:Plant_Zone_1, 9

Fixed effects:
            Estimate Std. Error      df t value Pr(>|t|)
(Intercept)  -0.5548     0.2193  8.8791   -2.53   0.0326 *
---
Signif. codes:  0 '***' 0.001 '**' 0.01 '*' 0.05 '.' 0.1 ' ' 1

Dispersion: 0.3655
```

Model residuals

Statistic	Value
Sample skewness	-0.01012
Sample excess kurtosis	-1.262
Passed Shapiro Wilk normality test	Yes (p-value = 0.09701 > 0.05)

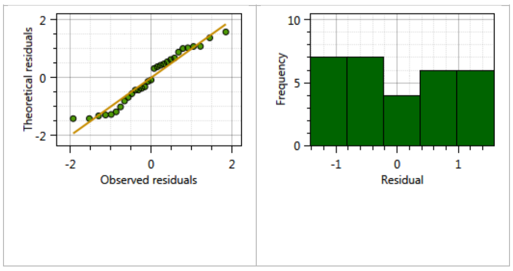

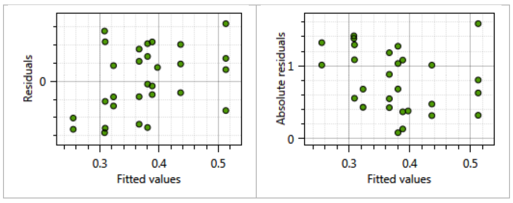

Ratio halting to total trial duration per zone

Selected zones	Zone 1, Zone 2
Data transformation	Natural logarithm
Analysis	Zone difference analysis

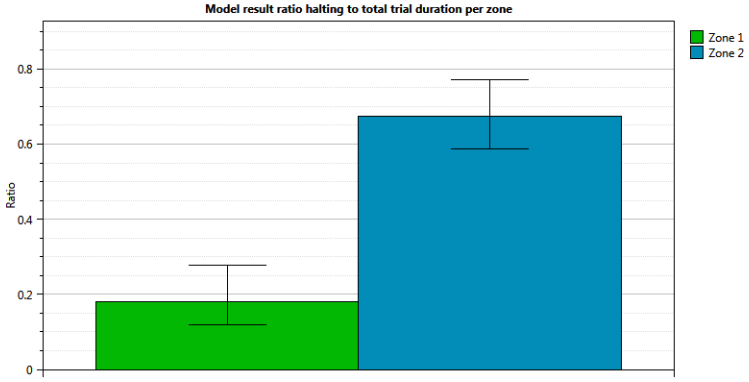

Results difference tests Zone 1 - Zone 2: p values and 95% confidence intervals of the difference on the transformed scale for each statistic.

Behaviour statistic	Cur3-RMX180
Ratio halting to total trial duration (diff. Zone 1 - Zone 2)	p=0.000283*** [-1.45, -0.631]

The model predictions and 95% confidence intervals for each statistic.

Statistic	Cur3-RMX180	Remark
Ratio halting to total trial duration (Zone 1)	0.181 [0.118, 0.278]	CR
Ratio halting to total trial duration (Zone 2)	0.673 [0.587, 0.772]	CR

CR = Check residuals

Data summary

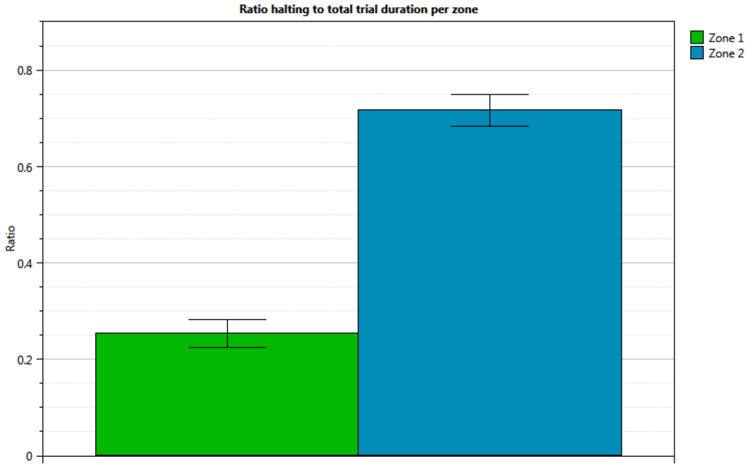

Genotype Zone 1	Genotype Zone 2	Genotype Zone 3	Mean Zone 1	StdErr Zone 1	Mean Zone 2	StdErr Zone 2
Cur3	RMX180	Neutral	0.25	0.03	0.72	0.03

Analysis ratio halting to total trial duration (Zone 1)

**Analysis model**	Linear mixed model fit by REML: Ratio_halting_to_total_trial_duration_Zone_1 ~ 1 + (1|Genotype_Zone_1:Plant_Zone_1) + (1|Genotype_Zone_2:Plant_Zone_2)
**Transformation**	Natural logarithm

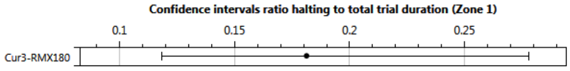

Confidence intervals ratio halting to total trial duration (Zone 1)

Genotype Zone 1	Genotype Zone 2	Mean	Lower 95% CL	Upper 95% CL	Group
Cur3	RMX180	0.1813	0.1182	0.2781	a

Model summary

```
Linear mixed model fit by REML. t-tests use Satterthwaite's method ['lmerModLmerTest']
Formula: Ratio_halting_to_total_trial_duration_Zone_1 ~ 1 + (1 | Genotype_Zone_1:Plant_Zone_1) +      (1
| Genotype_Zone_2:Plant_Zone_2)
   Data: data

REML criterion at convergence: 112.6

Scaled residuals:
    Min      1Q  Median      3Q     Max
-2.7369 -0.3294  0.2530  0.6067  1.2204

Random effects:
 Groups                       Name        Variance Std.Dev.
 Genotype_Zone_1:Plant_Zone_1 (Intercept) 0.00000  0.0000
 Genotype_Zone_2:Plant_Zone_2 (Intercept) 0.08948  0.2991
 Residual                                 1.03961  1.0196
```

```
Number of obs: 38, groups:  Genotype_Zone_1:Plant_Zone_1, 10; Genotype_Zone_2:Plant_Zone_2, 10

Fixed effects:
            Estimate Std. Error      df t value Pr(>|t|)
(Intercept)  -1.7078     0.1907  9.5207  -8.953 6.09e-06 ***
---
Signif. codes:  0 '***' 0.001 '**' 0.01 '*' 0.05 '.' 0.1 ' ' 1
```

Model residuals

Statistic	Value
Sample skewness	-1.408
Sample excess kurtosis	1.798
Passed Shapiro Wilk normality test	No (p-value = 0.0003123 < 0.05)

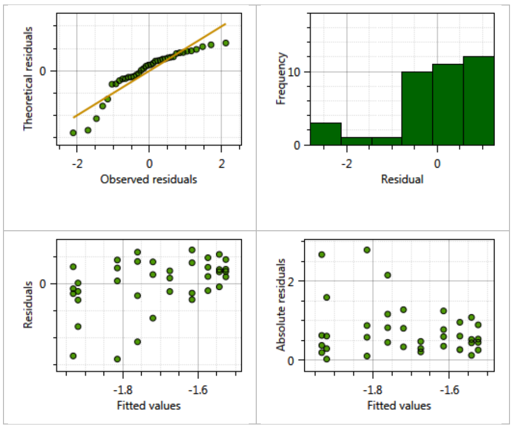

Analysis ratio halting to total trial duration (Zone 2)

**Analysis model**	Linear mixed model fit by REML: Ratio_halting_to_total_trial_duration_Zone_2 ~ 1 + (1|Genotype_Zone_1:Plant_Zone_1) + (1|Genotype_Zone_2:Plant_Zone_2)
**Transformation**	Natural logarithm

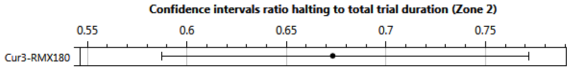

Confidence intervals ratio halting to total trial duration (Zone 2)

Genotype Zone 1	Genotype Zone 2	Mean	Lower 95% CL	Upper 95% CL	Group
Cur3	RMX180	0.6732	0.5872	0.7719	a

Model summary

```
Linear mixed model fit by REML. t-tests use Satterthwaite's method ['lmerModLmerTest']
Formula: Ratio_halting_to_total_trial_duration_Zone_2 ~ 1 + (1 | Genotype_Zone_1:Plant_Zone_1) +      (1
| Genotype_Zone_2:Plant_Zone_2)
   Data: data

REML criterion at convergence: 45.9

Scaled residuals:
    Min      1Q  Median      3Q     Max
-4.5190 -0.2928  0.1591  0.6481  0.9376

Random effects:
 Groups                       Name        Variance Std.Dev.
 Genotype_Zone_1:Plant_Zone_1 (Intercept) 0.0000   0.000
 Genotype_Zone_2:Plant_Zone_2 (Intercept) 0.0000   0.000
 Residual                                 0.1781   0.422
Number of obs: 39, groups:  Genotype_Zone_1:Plant_Zone_1, 10; Genotype_Zone_2:Plant_Zone_2, 10

Fixed effects:
            Estimate Std. Error       df t value Pr(>|t|)
(Intercept) -0.39565    0.06757 38.00000  -5.855 8.99e-07 ***
---
Signif. codes:  0 '***' 0.001 '**' 0.01 '*' 0.05 '.' 0.1 ' ' 1
```

Model residuals

Statistic	Value
Sample skewness	-2.729
Sample excess kurtosis	10.39
Passed Shapiro Wilk normality test	No (p-value = 6.762E-07 < 0.05)

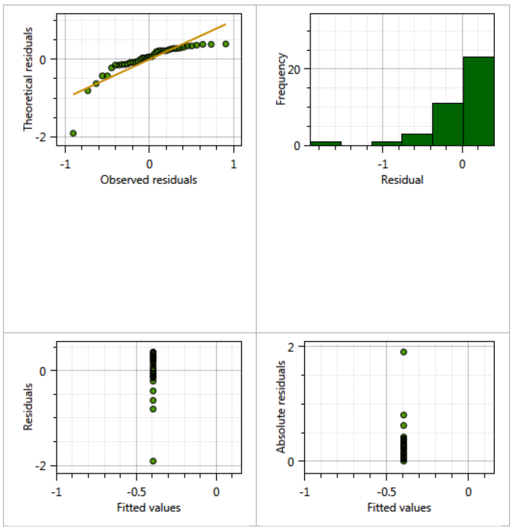

Analysis ratio halting to total trial duration (diff. Zone 1 - Zone 2)

**Analysis model**	Generalized linear mixed model with dispersion factor, formula=cbind(Ratio_halting_to_total_trial_duration_Zone_1,Ratio_halting_to_total_trial_duration_Zone_2) ~ 1 + (1|Genotype_Zone_1:Plant_Zone_1) + (1|Genotype_Zone_2:Plant_Zone_2)
**Transformation**	Logit

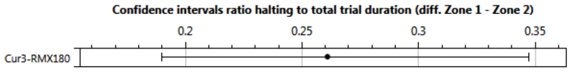

Confidence intervals ratio halting to total trial duration (diff. Zone 1 - Zone 2)

Genotype Zone 1	Genotype Zone 2	Mean	Lower 95% CL	Upper 95% CL	Group
Cur3	RMX180	0.2609	0.1897	0.3474	a

Model summary

```
Linear mixed model fit by REML. t-tests use Satterthwaite's method ['lmerModLmerTest']
Formula: ziFormula
   Data: data
Weights: wi

REML criterion at convergence: 112.1

Scaled residuals: 
     Min       1Q   Median       3Q      Max 
-1.33206 -0.73969 -0.03082  0.50843  2.48961 

Random effects:
 Groups                      Name        Variance Std.Dev.
 Genotype_Zone_1:Plant_Zone_1 (Intercept) 0.00000  0.0000  
 Genotype_Zone_2:Plant_Zone_2 (Intercept) 0.08699  0.2949  
 Residual                                 0.17614  0.4197  
Number of obs: 39, groups:  Genotype_Zone_1:Plant_Zone_1, 10; Genotype_Zone_2:Plant_Zone_2, 10

Fixed effects:
            Estimate Std. Error      df t value Pr(>|t|)    
(Intercept)  -1.0411     0.1814  8.9763  -5.739 0.000283 ***
---
Signif. codes:  0 '***' 0.001 '**' 0.01 '*' 0.05 '.' 0.1 ' ' 1

Dispersion: 0.4197
```

Model residuals

Statistic	Value
Sample skewness	0.7043
Sample excess kurtosis	0.1095
Passed Shapiro Wilk normality test	Yes (p-value = 0.06503 > 0.05)

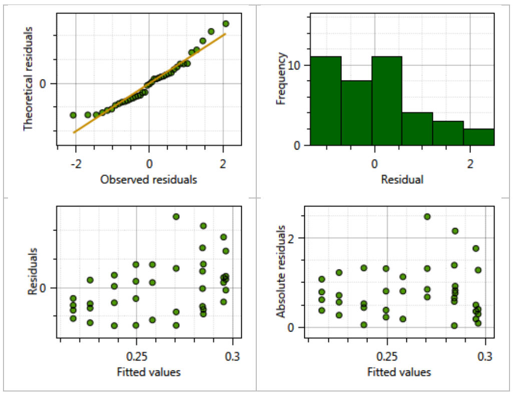

Ratio halting to total trial duration per zone per hour

Selected hours	0, 1, 2, 3, 4, 5, 6, 7
Selected zones	Zone 1, Zone 2
Data transformation	Natural logarithm
Analysis	Zone difference analysis

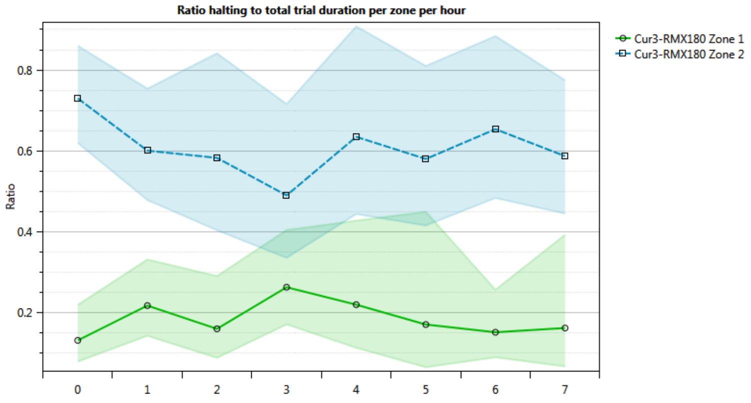

Results difference tests Zone 1 - Zone 2: p values and 95% confidence intervals of the difference on the transformed scale for each statistic.

Behaviour statistic	Cur3-RMX180	Remark
Ratio halting to total trial duration H0 (diff. Zone 1 - Zone 2)	p=5.49E-05****<br>[-1.67, -0.64]	CR
Ratio halting to total trial duration H1 (diff. Zone 1 - Zone 2)	p=0.00827**<br>[-1.12, -0.178]	CR
Ratio halting to total trial duration H2 (diff. Zone 1 - Zone 2)	p=0.0124*<br>[-1.55, -0.251]	CR
Ratio halting to total trial duration H3 (diff. Zone 1 - Zone 2)	p=0.0315*<br>[-1.3, -0.0767]	CR
Ratio halting to total trial duration H4 (diff. Zone 1 - Zone 2)	p=0.00968**<br>[-1.61, -0.298]	CR
Ratio halting to total trial duration H5 (diff. Zone 1 - Zone 2)	p=0.006**<br>[-1.72, -0.386]	CR
Ratio halting to total trial duration H6 (diff. Zone 1 - Zone 2)	p=0.000819***<br>[-2.11, -0.762]	CR
Ratio halting to total trial duration H7 (diff. Zone 1 - Zone 2)	p=0.00404**<br>[-1.86, -0.474]	CR

CR = Check residuals

The model predictions and 95% confidence intervals for each statistic.

Statistic	Cur3-RMX180	Remark
Ratio halting to total trial duration (H0 - Zone 1)	0.131	CR

Statistic	Cur3-RMX180	Remark
	[0.0786, 0.219]	
Ratio halting to total trial duration (H0 - Zone 2)	0.73	CR
	[0.619, 0.86]	
Ratio halting to total trial duration (H1 - Zone 1)	0.217	CR
	[0.142, 0.332]	
Ratio halting to total trial duration (H1 - Zone 2)	0.601	CR
	[0.478, 0.754]	
Ratio halting to total trial duration (H2 - Zone 1)	0.16	
	[0.0879, 0.291]	
Ratio halting to total trial duration (H2 - Zone 2)	0.583	CR
	[0.404, 0.841]	
Ratio halting to total trial duration (H3 - Zone 1)	0.263	CR
	[0.171, 0.404]	
Ratio halting to total trial duration (H3 - Zone 2)	0.49	CR
	[0.335, 0.716]	
Ratio halting to total trial duration (H4 - Zone 1)	0.22	
	[0.113, 0.428]	
Ratio halting to total trial duration (H4 - Zone 2)	0.635	CR
	[0.444, 0.908]	
Ratio halting to total trial duration (H5 - Zone 1)	0.17	
	[0.0646, 0.45]	
Ratio halting to total trial duration (H5 - Zone 2)	0.58	CR
	[0.415, 0.81]	
Ratio halting to total trial duration (H6 - Zone 1)	0.151	
	[0.0892, 0.257]	
Ratio halting to total trial duration (H6 - Zone 2)	0.654	CR
	[0.483, 0.884]	
Ratio halting to total trial duration (H7 - Zone 1)	0.162	CR
	[0.0667, 0.393]	
Ratio halting to total trial duration (H7 - Zone 2)	0.587	CR
	[0.445, 0.775]	

CR = Check residuals

Data summary

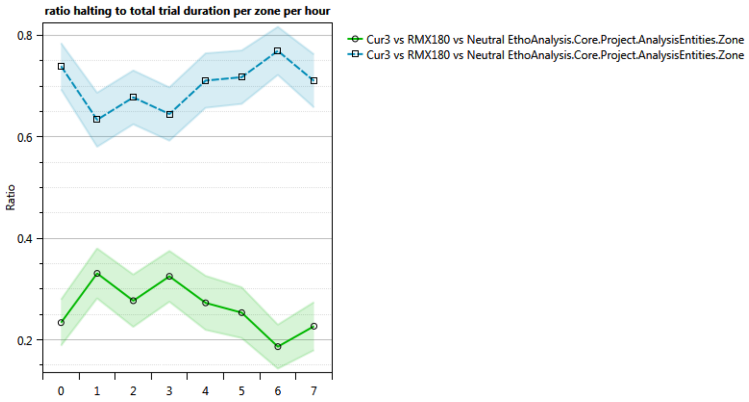

Genotype Zone 1	Genotype Zone 2	Genotype Zone 3	Mean H0 - Zone 1	StdErr H0 - Zone 1	Mean H0 - Zone 2	StdErr H0 - Zone 2	Mean H1 - Zone 1	StdErr H1 - Zone 1	Mean H1 - Zone 2	StdErr H1 - Zone 2	Mean H2 - Zone 1	StdErr H2 - Zone 1	Mean H2 - Zone 2	StdErr H2 - Zone 2	Mean H3 - Zone 1	StdErr H3 - Zone 1	Mean H3 - Zone 2	StdErr H3 - Zone 2	Mean H4 - Zone 1	StdErr H4 - Zone 1	Mean H4 - Zone 2	StdErr H4 - Zone 2	Mean H5 - Zone 1	StdErr H5 - Zone 1	Mean H5 - Zone 2	StdErr H5 - Zone 2	Mean H6 - Zone 1	StdErr H6 - Zone 1	Mean H6 - Zone 2	StdErr H6 - Zone 2
Cur3	RMX180	Neutral	0.23	0.05	0.74	0.05	0.33	0.05	0.63	0.05	0.28	0.05	0.68	0.05	0.33	0.05	0.65	0.05	0.27	0.05	0.71	0.05	0.25	0.05	0.72	0.05	0.19	0.04	0.77	0.05

Analysis ratio halting to total trial duration (H0 - Zone 1)

**Analysis model**	Linear mixed model fit by REML: Ratio_halting_to_total_trial_duration_H0_Zone_1 ~ 1 + (1|Genotype_Zone_1:Plant_Zone_1) + (1|Genotype_Zone_2:Plant_Zone_2)
**Transformation**	Natural logarithm

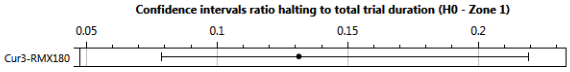

Confidence intervals ratio halting to total trial duration (H0 - Zone 1)

Genotype Zone 1	Genotype Zone 2	Mean	Lower 95% CL	Upper 95% CL	Group
Cur3	RMX180	0.1313	0.07858	0.2193	a

Model summary

```
Linear mixed model fit by REML. t-tests use Satterthwaite's method ['lmerModLmerTest']
Formula: Ratio_halting_to_total_trial_duration_H0_Zone_1 ~ 1 + (1 | Genotype_Zone_1:Plant_Zone_1) +
(1 | Genotype_Zone_2:Plant_Zone_2)
   Data: data

REML criterion at convergence: 118

Scaled residuals:
    Min      1Q  Median      3Q     Max
-1.7789 -0.8211  0.1366  0.6774  1.4027

Random effects:
 Groups                       Name        Variance Std.Dev.
 Genotype_Zone_1:Plant_Zone_1 (Intercept) 0.000    0.000
 Genotype_Zone_2:Plant_Zone_2 (Intercept) 0.000    0.000
 Residual                                 2.095    1.447
Number of obs: 33, groups:  Genotype_Zone_1:Plant_Zone_1, 10; Genotype_Zone_2:Plant_Zone_2, 10

Fixed effects:
            Estimate Std. Error     df t value Pr(>|t|)
(Intercept)   -2.030      0.252 32.000  -8.058 3.36e-09 ***
---
Signif. codes:  0 '***' 0.001 '**' 0.01 '*' 0.05 '.' 0.1 ' ' 1
```

Model residuals

Statistic	Value
Sample skewness	-0.5082
Sample excess kurtosis	-0.8382
Passed Shapiro Wilk normality test	No (p-value = 0.02272 < 0.05)

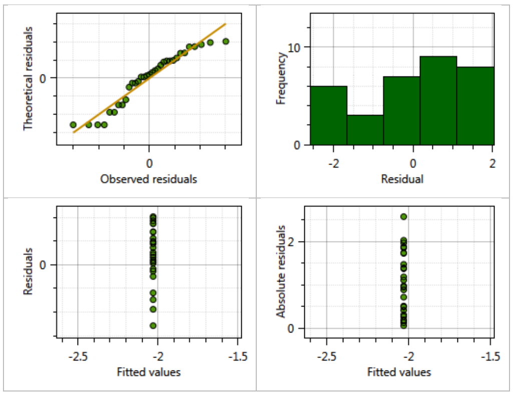

Analysis ratio halting to total trial duration (H0 - Zone 2)

**Analysis model**	Linear mixed model fit by REML: Ratio_halting_to_total_trial_duration_H0_Zone_2 ~ 1 + (1|Genotype_Zone_1:Plant_Zone_1) + (1|Genotype_Zone_2:Plant_Zone_2)
**Transformation**	Natural logarithm

Confidence intervals ratio halting to total trial duration (H0 - Zone 2)

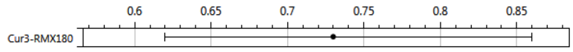

Confidence intervals ratio halting to total trial duration (H0 - Zone 2)

Genotype Zone 1	Genotype Zone 2	Mean	Lower 95% CL	Upper 95% CL	Group
Cur3	RMX180	0.7299	0.6195	0.86	a

Model summary

```
Linear mixed model fit by REML. t-tests use Satterthwaite's method ['lmerModLmerTest']
Formula: Ratio_halting_to_total_trial_duration_H0_Zone_2 ~ 1 + (1 | Genotype_Zone_1:Plant_Zone_1) +
(1 | Genotype_Zone_2:Plant_Zone_2)
   Data: data

REML criterion at convergence: 44.2

Scaled residuals:
    Min      1Q  Median      3Q     Max
-3.4657 -0.2658  0.3535  0.6889  0.8866

Random effects:
 Groups                       Name        Variance Std.Dev.
 Genotype_Zone_1:Plant_Zone_1 (Intercept) 0.006092 0.07805
 Genotype_Zone_2:Plant_Zone_2 (Intercept) 0.000000 0.00000
 Residual                                 0.175355 0.41875
Number of obs: 37, groups:  Genotype_Zone_1:Plant_Zone_1, 10; Genotype_Zone_2:Plant_Zone_2, 10

Fixed effects:
            Estimate Std. Error       df t value Pr(>|t|)
(Intercept) -0.31487    0.07319  9.59662  -4.302  0.00171 **
---
Signif. codes:  0 '***' 0.001 '**' 0.01 '*' 0.05 '.' 0.1 ' ' 1
```

Model residuals

Statistic	Value
Sample skewness	-2.057
Sample excess kurtosis	4.387
Passed Shapiro Wilk normality test	No (p-value = 1.508E-06 < 0.05)

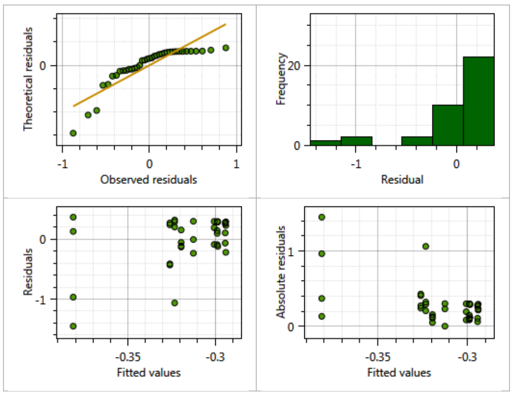

Analysis ratio halting to total trial duration (H1 - Zone 1)

**Analysis model**	Linear mixed model fit by REML: Ratio_halting_to_total_trial_duration_H1_Zone_1 ~ 1 + (1|Genotype_Zone_1:Plant_Zone_1) + (1|Genotype_Zone_2:Plant_Zone_2)
**Transformation**	Natural logarithm

Confidence intervals ratio halting to total trial duration (H1 - Zone 1)

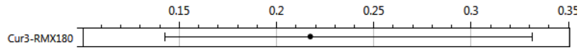

Confidence intervals ratio halting to total trial duration (H1 - Zone 1)

Genotype Zone 1	Genotype Zone 2	Mean	Lower 95% CL	Upper 95% CL	Group
Cur3	RMX180	0.2173	0.1425	0.3315	a

Model summary

```
Linear mixed model fit by REML. t-tests use Satterthwaite's method ['lmerModLmerTest']
Formula: Ratio_halting_to_total_trial_duration_H1_Zone_1 ~ 1 + (1 | Genotype_Zone_1:Plant_Zone_1) +
(1 | Genotype_Zone_2:Plant_Zone_2)
   Data: data

REML criterion at convergence: 114.1

Scaled residuals:
    Min      1Q  Median      3Q     Max
-2.5046 -0.5573  0.3640  0.8124  1.2416

Random effects:
 Groups                       Name        Variance  Std.Dev.
 Genotype_Zone_1:Plant_Zone_1 (Intercept) 0.000e+00 0.000e+00
 Genotype_Zone_2:Plant_Zone_2 (Intercept) 6.165e-17 7.852e-09
```

```
 Residual                                  1.511e+00 1.229e+00
Number of obs: 35, groups:  Genotype_Zone_1:Plant_Zone_1, 10; Genotype_Zone_2:Plant_Zone_2, 10

Fixed effects:
            Estimate Std. Error      df t value Pr(>|t|)
(Intercept)  -1.5263     0.2078 34.0000  -7.346 1.64e-08 ***
---
Signif. codes:  0 '***' 0.001 '**' 0.01 '*' 0.05 '.' 0.1 ' ' 1
```

Model residuals

Statistic	Value
Sample skewness	-0.744
Sample excess kurtosis	-0.3347
Passed Shapiro Wilk normality test	No (p-value = 0.01334 < 0.05)

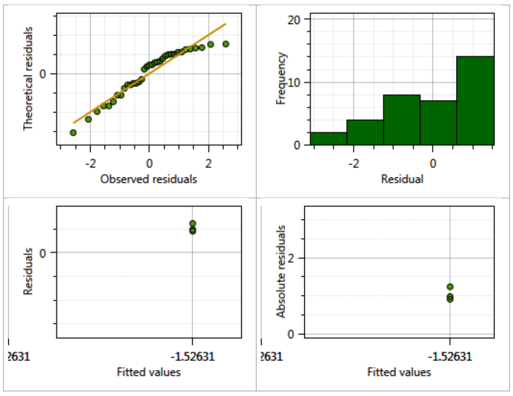

Analysis ratio halting to total trial duration (H1 - Zone 2)

**Analysis model**	Linear mixed model fit by REML: Ratio_halting_to_total_trial_duration_H1_Zone_2 ~ 1 + (1|Genotype_Zone_1:Plant_Zone_1) + (1|Genotype_Zone_2:Plant_Zone_2)
**Transformation**	Natural logarithm

Confidence intervals ratio halting to total trial duration (H1 - Zone 2)

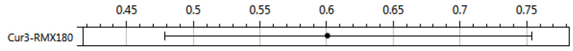

Confidence intervals ratio halting to total trial duration (H1 - Zone 2)

Genotype Zone 1	Genotype Zone 2	Mean	Lower 95% CL	Upper 95% CL	Group
Cur3	RMX180	0.6005	0.4783	0.754	a

Model summary

```
Linear mixed model fit by REML. t-tests use Satterthwaite's method ['lmerModLmerTest']
Formula: Ratio_halting_to_total_trial_duration_H1_Zone_2 ~ 1 + (1 | Genotype_Zone_1:Plant_Zone_1) +
(1 | Genotype_Zone_2:Plant_Zone_2)
   Data: data

REML criterion at convergence: 66.6

Scaled residuals:
    Min      1Q  Median      3Q     Max
-2.4535 -0.5777  0.4095  0.7438  0.9082

Random effects:
 Groups                       Name        Variance Std.Dev.
 Genotype_Zone_1:Plant_Zone_1 (Intercept) 0.000000 0.00000
 Genotype_Zone_2:Plant_Zone_2 (Intercept) 0.005814 0.07625
 Residual                                 0.348829 0.59062
Number of obs: 36, groups:  Genotype_Zone_1:Plant_Zone_1, 10; Genotype_Zone_2:Plant_Zone_2, 10

Fixed effects:
            Estimate Std. Error      df t value Pr(>|t|)
(Intercept)  -0.5099     0.1014  9.4806  -5.029 0.000606 ***
---
Signif. codes:  0 '***' 0.001 '**' 0.01 '*' 0.05 '.' 0.1 ' ' 1
```

Model residuals

Statistic	Value
Sample skewness	-1.303
Sample excess kurtosis	0.7027
Passed Shapiro Wilk normality test	No (p-value = 2.223E-05 < 0.05)

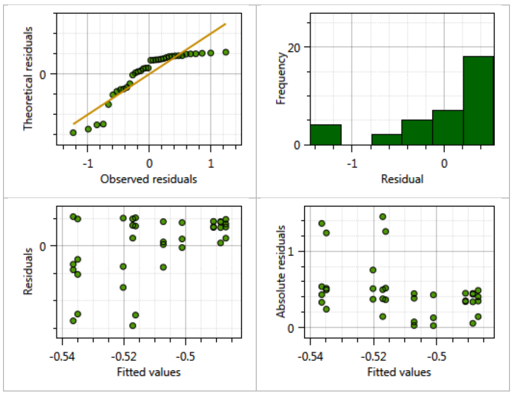

Analysis ratio halting to total trial duration (H2 - Zone 1)

**Analysis model**	Linear mixed model fit by REML: Ratio_halting_to_total_trial_duration_H2_Zone_1 ~ 1 + (1|Genotype_Zone_1:Plant_Zone_1) + (1|Genotype_Zone_2:Plant_Zone_2)
**Transformation**	Natural logarithm

Confidence intervals ratio halting to total trial duration (H2 - Zone 1)

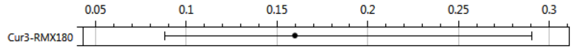

Confidence intervals ratio halting to total trial duration (H2 - Zone 1)

Genotype Zone 1	Genotype Zone 2	Mean	Lower 95% CL	Upper 95% CL	Group
Cur3	RMX180	0.1598	0.08792	0.2905	a

Model summary

```
Linear mixed model fit by REML. t-tests use Satterthwaite's method ['lmerModLmerTest']
Formula: Ratio_halting_to_total_trial_duration_H2_Zone_1 ~ 1 + (1 | Genotype_Zone_1:Plant_Zone_1) +
(1 | Genotype_Zone_2:Plant_Zone_2)
   Data: data

REML criterion at convergence: 116.9

Scaled residuals:
    Min      1Q  Median      3Q     Max
-1.7472 -0.7432  0.1648  0.6700  1.3397

Random effects:
 Groups                       Name        Variance Std.Dev.
 Genotype_Zone_1:Plant_Zone_1 (Intercept) 0.2568   0.5068
 Genotype_Zone_2:Plant_Zone_2 (Intercept) 0.0000   0.0000
 Residual                                 1.4481   1.2034
Number of obs: 35, groups:  Genotype_Zone_1:Plant_Zone_1, 10; Genotype_Zone_2:Plant_Zone_2, 10

Fixed effects:
            Estimate Std. Error      df t value Pr(>|t|)
(Intercept)  -1.8337     0.2602  8.1890  -7.047 9.59e-05 ***
---
Signif. codes:  0 '***' 0.001 '**' 0.01 '*' 0.05 '.' 0.1 ' ' 1
```

Model residuals

Statistic	Value
Sample skewness	-0.2854
Sample excess kurtosis	-1.115
Passed Shapiro Wilk normality test	Yes (p-value = 0.05806 > 0.05)

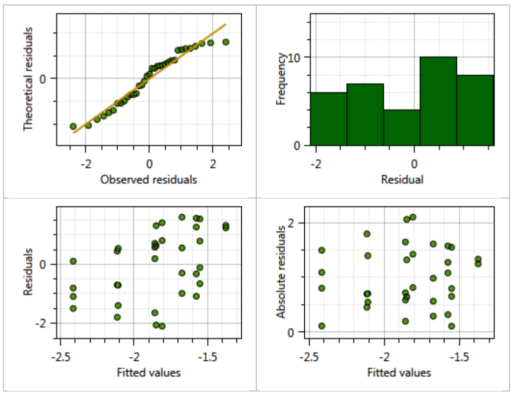

Analysis ratio halting to total trial duration (H2 - Zone 2)

**Analysis model**	Linear mixed model fit by REML: Ratio_halting_to_total_trial_duration_H2_Zone_2 ~ 1 + (1|Genotype_Zone_1:Plant_Zone_1) + (1|Genotype_Zone_2:Plant_Zone_2)
**Transformation**	Natural logarithm

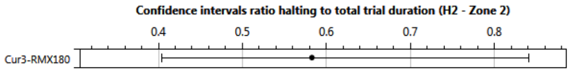

Confidence intervals ratio halting to total trial duration (H2 - Zone 2)

Genotype Zone 1	Genotype Zone 2	Mean	Lower 95% CL	Upper 95% CL	Group
Cur3	RMX180	0.5827	0.4035	0.8413	a

Model summary

```
Linear mixed model fit by REML. t-tests use Satterthwaite's method ['lmerModLmerTest']
Formula: Ratio_halting_to_total_trial_duration_H2_Zone_2 ~ 1 + (1 | Genotype_Zone_1:Plant_Zone_1) +
(1 | Genotype_Zone_2:Plant_Zone_2)
   Data: data

REML criterion at convergence: 89.8

Scaled residuals:
    Min      1Q  Median      3Q     Max
-2.9312 -0.1626  0.3074  0.5389  1.3260

Random effects:
 Groups                       Name        Variance Std.Dev.
 Genotype_Zone_1:Plant_Zone_1 (Intercept) 0.0921   0.3035
 Genotype_Zone_2:Plant_Zone_2 (Intercept) 0.0000   0.0000
 Residual                                 0.5717   0.7561
Number of obs: 37, groups:  Genotype_Zone_1:Plant_Zone_1, 10; Genotype_Zone_2:Plant_Zone_2, 10

Fixed effects:
            Estimate Std. Error      df t value Pr(>|t|)
(Intercept)  -0.5401     0.1573  7.4676  -3.433   0.0099 **
---
Signif. codes:  0 '***' 0.001 '**' 0.01 '*' 0.05 '.' 0.1 ' ' 1
```

Model residuals

Statistic	Value
Sample skewness	-1.888
Sample excess kurtosis	3.425
Passed Shapiro Wilk normality test	No (p-value = 3.341E-06 < 0.05)

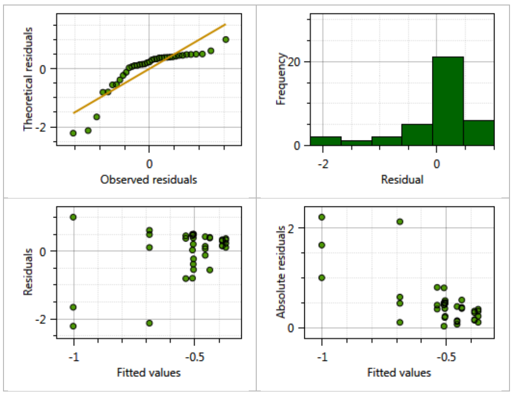

Analysis ratio halting to total trial duration (H3 - Zone 1)

**Analysis model**	Linear mixed model fit by REML: Ratio_halting_to_total_trial_duration_H3_Zone_1 ~ 1 + (1|Genotype_Zone_1:Plant_Zone_1) + (1|Genotype_Zone_2:Plant_Zone_2)
**Transformation**	Natural logarithm

Confidence intervals ratio halting to total trial duration (H3 - Zone 1)

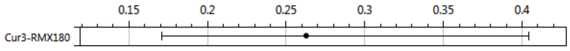

Confidence intervals ratio halting to total trial duration (H3 - Zone 1)

Genotype Zone 1	Genotype Zone 2	Mean	Lower 95% CL	Upper 95% CL	Group
Cur3	RMX180	0.2627	0.1706	0.4044	a

Model summary

```
Linear mixed model fit by REML. t-tests use Satterthwaite's method ['lmerModLmerTest']
Formula: Ratio_halting_to_total_trial_duration_H3_Zone_1 ~ 1 + (1 | Genotype_Zone_1:Plant_Zone_1) +
(1 | Genotype_Zone_2:Plant_Zone_2)
   Data: data

REML criterion at convergence: 98.3

Scaled residuals:
    Min      1Q  Median      3Q     Max
-2.7786 -0.4938  0.1939  0.7501  1.1192

Random effects:
 Groups                       Name        Variance Std.Dev.
 Genotype_Zone_1:Plant_Zone_1 (Intercept) 0.000    0.000
 Genotype_Zone_2:Plant_Zone_2 (Intercept) 0.000    0.000
 Residual                                 1.384    1.176
Number of obs: 31, groups:  Genotype_Zone_1:Plant_Zone_1, 10; Genotype_Zone_2:Plant_Zone_2, 10

Fixed effects:
            Estimate Std. Error      df t value Pr(>|t|)
(Intercept)  -1.3367     0.2113 30.0000  -6.327 5.61e-07 ***
---
Signif. codes:  0 '***' 0.001 '**' 0.01 '*' 0.05 '.' 0.1 ' ' 1
```

Model residuals

Statistic	Value
Sample skewness	-1.176
Sample excess kurtosis	0.8915
Passed Shapiro Wilk normality test	No (p-value = 0.003136 < 0.05)

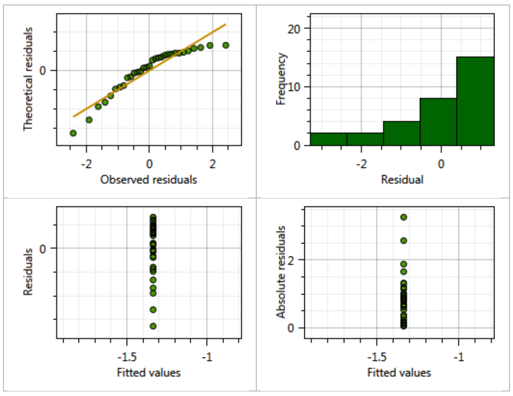

Analysis ratio halting to total trial duration (H3 - Zone 2)

**Analysis model**	Linear mixed model fit by REML: Ratio_halting_to_total_trial_duration_H3_Zone_2 ~ 1 + (1|Genotype_Zone_1:Plant_Zone_1) + (1|Genotype_Zone_2:Plant_Zone_2)
**Transformation**	Natural logarithm

Confidence intervals ratio halting to total trial duration (H3 - Zone 2)

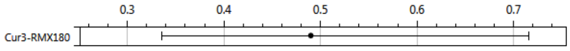

Confidence intervals ratio halting to total trial duration (H3 - Zone 2)

Genotype Zone 1	Genotype Zone 2	Mean	Lower 95% CL	Upper 95% CL	Group
Cur3	RMX180	0.4899	0.3353	0.716	a

Model summary

```
Linear mixed model fit by REML. t-tests use Satterthwaite's method ['lmerModLmerTest']
Formula: Ratio_halting_to_total_trial_duration_H3_Zone_2 ~ 1 + (1 | Genotype_Zone_1:Plant_Zone_1) +
(1 | Genotype_Zone_2:Plant_Zone_2)
   Data: data

REML criterion at convergence: 110.9

Scaled residuals:
    Min      1Q  Median      3Q     Max
-3.8769 -0.3182  0.3461  0.6432  0.7550

Random effects:
 Groups                       Name        Variance  Std.Dev.
 Genotype_Zone_1:Plant_Zone_1 (Intercept) 5.350e-17 7.314e-09
 Genotype_Zone_2:Plant_Zone_2 (Intercept) 3.839e-02 1.959e-01
 Residual                                 9.513e-01 9.753e-01
Number of obs: 39, groups:  Genotype_Zone_1:Plant_Zone_1, 10; Genotype_Zone_2:Plant_Zone_2, 10

Fixed effects:
            Estimate Std. Error      df t value Pr(>|t|)
(Intercept)  -0.7135     0.1681  9.1382  -4.245  0.00209 **
---
Signif. codes:  0 '***' 0.001 '**' 0.01 '*' 0.05 '.' 0.1 ' ' 1
```

Model residuals

Statistic	Value
Sample skewness	-2.335
Sample excess kurtosis	6.409
Passed Shapiro Wilk normality test	No (p-value = 3.04E-07 < 0.05)

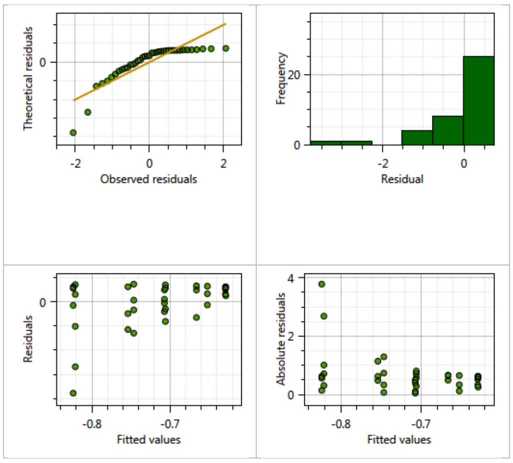

Analysis ratio halting to total trial duration (H4 - Zone 1)

**Analysis model**	Linear mixed model fit by REML: Ratio_halting_to_total_trial_duration_H4_Zone_1 ~ 1 + (1|Genotype_Zone_1:Plant_Zone_1) + (1|Genotype_Zone_2:Plant_Zone_2)
**Transformation**	Natural logarithm

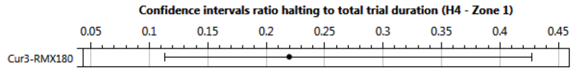

Confidence intervals ratio halting to total trial duration (H4 - Zone 1)

Genotype Zone 1	Genotype Zone 2	Mean	Lower 95% CL	Upper 95% CL	Group
Cur3	RMX180	0.2197	0.1129	0.4275	a

Model summary

```
Linear mixed model fit by REML. t-tests use Satterthwaite's method ['lmerModLmerTest']
Formula: Ratio_halting_to_total_trial_duration_H4_Zone_1 ~ 1 + (1 | Genotype_Zone_1:Plant_Zone_1) +
(1 | Genotype_Zone_2:Plant_Zone_2)
   Data: data

REML criterion at convergence: 92

Scaled residuals:
     Min       1Q   Median       3Q      Max
-2.29100 -0.66085  0.01235  0.81416  1.32386

Random effects:
 Groups                       Name        Variance Std.Dev.
 Genotype_Zone_1:Plant_Zone_1 (Intercept) 0.2587   0.5086
 Genotype_Zone_2:Plant_Zone_2 (Intercept) 0.0000   0.0000
 Residual                                 1.3593   1.1659
Number of obs: 28, groups:  Genotype_Zone_1:Plant_Zone_1, 10; Genotype_Zone_2:Plant_Zone_2, 9

Fixed effects:
            Estimate Std. Error      df t value Pr(>|t|)
(Intercept)  -1.5156     0.2777  6.5463  -5.458  0.00118 **
---
Signif. codes:  0 '***' 0.001 '**' 0.01 '*' 0.05 '.' 0.1 ' ' 1
```

Model residuals

Statistic	Value
Sample skewness	-0.4995
Sample excess kurtosis	-0.4037
Passed Shapiro Wilk normality test	Yes (p-value = 0.2291 > 0.05)

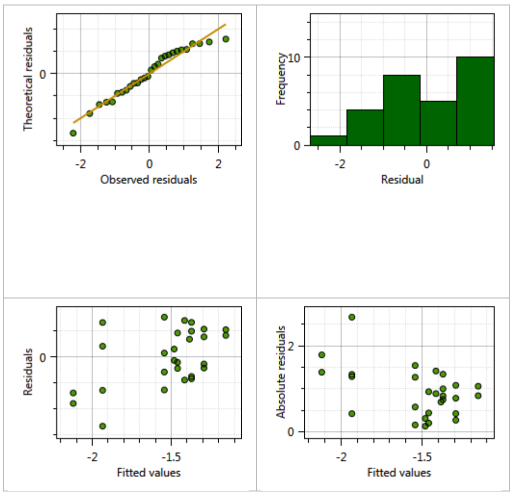

Analysis ratio halting to total trial duration (H4 - Zone 2)

**Analysis model**	Linear mixed model fit by REML: Ratio_halting_to_total_trial_duration_H4_Zone_2 ~ 1 + (1|Genotype_Zone_1:Plant_Zone_1) + (1|Genotype_Zone_2:Plant_Zone_2)
**Transformation**	Natural logarithm

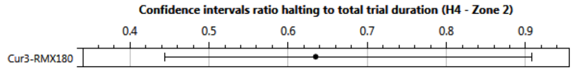

Confidence intervals ratio halting to total trial duration (H4 - Zone 2)

Genotype Zone 1	Genotype Zone 2	Mean	Lower 95% CL	Upper 95% CL	Group
Cur3	RMX180	0.6347	0.4436	0.9082	a

Model summary

```
Linear mixed model fit by REML. t-tests use Satterthwaite's method ['lmerModLmerTest']
Formula: Ratio_halting_to_total_trial_duration_H4_Zone_2 ~ 1 + (1 | Genotype_Zone_1:Plant_Zone_1) +
(1 | Genotype_Zone_2:Plant_Zone_2)
   Data: data
```

```
REML criterion at convergence: 86

Scaled residuals:
    Min      1Q  Median      3Q     Max
-3.7717 -0.0954  0.3617  0.5280  0.9480

Random effects:
 Groups                      Name        Variance Std.Dev.
 Genotype_Zone_1:Plant_Zone_1 (Intercept) 0.03409  0.1846
 Genotype_Zone_2:Plant_Zone_2 (Intercept) 0.05740  0.2396
 Residual                                0.50199  0.7085
Number of obs: 37, groups:  Genotype_Zone_1:Plant_Zone_1, 10; Genotype_Zone_2:Plant_Zone_2, 10

Fixed effects:
            Estimate Std. Error      df t value Pr(>|t|)
(Intercept)  -0.4545     0.1513  6.9543  -3.004     0.02 *
---
Signif. codes:  0 '***' 0.001 '**' 0.01 '*' 0.05 '.' 0.1 ' ' 1
```

Model residuals

Statistic	Value
Sample skewness	-2.448
Sample excess kurtosis	6.968
Passed Shapiro Wilk normality test	No (p-value = 4.295E-07 < 0.05)

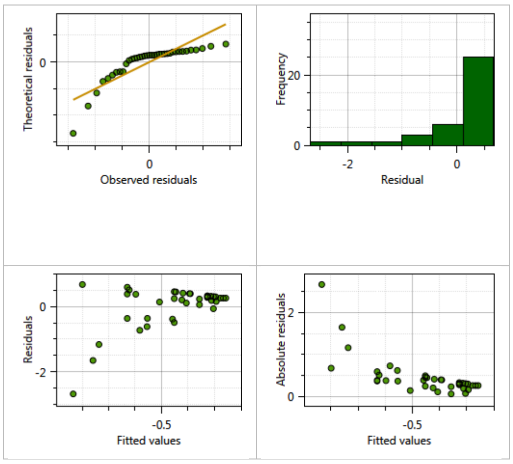

Analysis ratio halting to total trial duration (H5 - Zone 1)

**Analysis model**	Linear mixed model fit by REML: Ratio_halting_to_total_trial_duration_H5_Zone_1 ~ 1 + (1|Genotype_Zone_1:Plant_Zone_1) + (1|Genotype_Zone_2:Plant_Zone_2)
**Transformation**	Natural logarithm

Confidence intervals ratio halting to total trial duration (H5 - Zone 1)

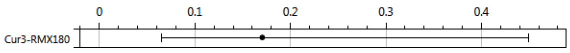

Confidence intervals ratio halting to total trial duration (H5 - Zone 1)

Genotype Zone 1	Genotype Zone 2	Mean	Lower 95% CL	Upper 95% CL	Group
Cur3	RMX180	0.1705	0.06465	0.4496	a

Model summary

```
Linear mixed model fit by REML. t-tests use Satterthwaite's method ['lmerModLmerTest']
Formula: Ratio_halting_to_total_trial_duration_H5_Zone_1 ~ 1 + (1 | Genotype_Zone_1:Plant_Zone_1) +
(1 | Genotype_Zone_2:Plant_Zone_2)
   Data: data

REML criterion at convergence: 101.4

Scaled residuals:
    Min      1Q  Median      3Q     Max
-2.0300 -0.4217  0.1774  0.4584  1.1581

Random effects:
 Groups                      Name        Variance Std.Dev.
 Genotype_Zone_1:Plant_Zone_1 (Intercept) 0.5983   0.7735
 Genotype_Zone_2:Plant_Zone_2 (Intercept) 0.7034   0.8387
 Residual                                1.2972   1.1389
Number of obs: 28, groups:  Genotype_Zone_1:Plant_Zone_1, 10; Genotype_Zone_2:Plant_Zone_2, 10

Fixed effects:
            Estimate Std. Error      df t value Pr(>|t|)
(Intercept)  -1.7691     0.4251  8.5353  -4.162  0.00274 **
---
Signif. codes:  0 '***' 0.001 '**' 0.01 '*' 0.05 '.' 0.1 ' ' 1
```

Model residuals

Statistic	Value
Sample skewness	-0.7206
Sample excess kurtosis	0.1911
Passed Shapiro Wilk normality test	Yes (p-value = 0.1179 > 0.05)

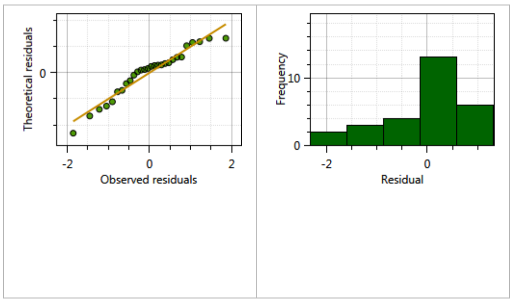

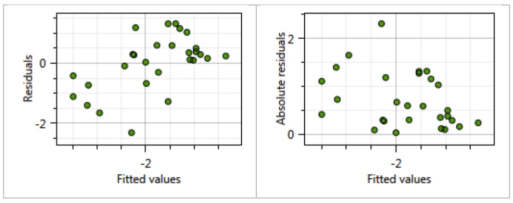

Analysis ratio halting to total trial duration (H5 - Zone 2)

**Analysis model**	Linear mixed model fit by REML: Ratio_halting_to_total_trial_duration_H5_Zone_2 ~ 1 + (1|Genotype_Zone_1:Plant_Zone_1) + (1|Genotype_Zone_2:Plant_Zone_2)
**Transformation**	Natural logarithm

Confidence intervals ratio halting to total trial duration (H5 - Zone 2)

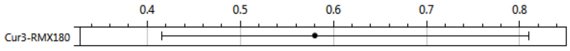

Confidence intervals ratio halting to total trial duration (H5 - Zone 2)

Genotype Zone 1	Genotype Zone 2	Mean	Lower 95% CL	Upper 95% CL	Group
Cur3	RMX180	0.58	0.4153	0.8101	a

Model summary

```
Linear mixed model fit by REML. t-tests use Satterthwaite's method ['lmerModLmerTest']
Formula: Ratio_halting_to_total_trial_duration_H5_Zone_2 ~ 1 + (1 | Genotype_Zone_1:Plant_Zone_1) +
(1 | Genotype_Zone_2:Plant_Zone_2)
   Data: data

REML criterion at convergence: 95.6

Scaled residuals:
    Min      1Q  Median      3Q     Max
-2.5868 -0.2176  0.4130  0.5626  1.0267

Random effects:
 Groups                       Name        Variance  Std.Dev.
 Genotype_Zone_1:Plant_Zone_1 (Intercept) 4.564e-19 6.756e-10
 Genotype_Zone_2:Plant_Zone_2 (Intercept) 6.355e-02 2.521e-01
 Residual                                 6.062e-01 7.786e-01
Number of obs: 39, groups:  Genotype_Zone_1:Plant_Zone_1, 10; Genotype_Zone_2:Plant_Zone_2, 10

Fixed effects:
            Estimate Std. Error      df t value Pr(>|t|)
(Intercept)  -0.5447     0.1481  9.1540  -3.678  0.00494 **
---
Signif. codes:  0 '***' 0.001 '**' 0.01 '*' 0.05 '.' 0.1 ' ' 1
```

Model residuals

Statistic	Value
Sample skewness	-1.548
Sample excess kurtosis	1.684
Passed Shapiro Wilk normality test	No (p-value = 9.356E-06 < 0.05)

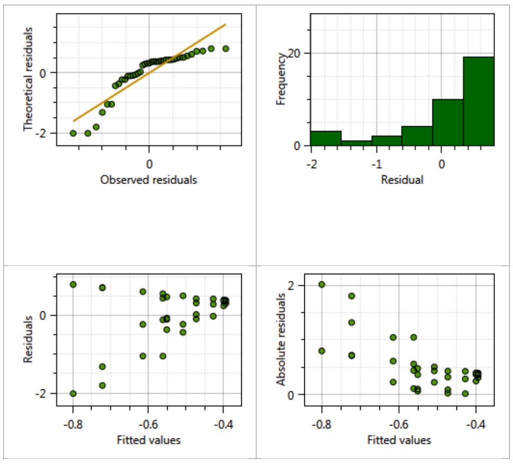

Analysis ratio halting to total trial duration (H6 - Zone 1)

**Analysis model**	Linear mixed model fit by REML: Ratio_halting_to_total_trial_duration_H6_Zone_1 ~ 1 + (1|Genotype_Zone_1:Plant_Zone_1) + (1|Genotype_Zone_2:Plant_Zone_2)
**Transformation**	Natural logarithm

Confidence intervals ratio halting to total trial duration (H6 - Zone 1)

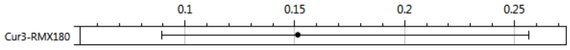

Confidence intervals ratio halting to total trial duration (H6 - Zone 1)

Genotype Zone 1	Genotype Zone 2	Mean	Lower 95% CL	Upper 95% CL	Group
Cur3	RMX180	0.1513	0.0892	0.2567	a

Model summary

```
Linear mixed model fit by REML. t-tests use Satterthwaite's method ['lmerModLmerTest']
Formula: Ratio_halting_to_total_trial_duration_H6_Zone_1 ~ 1 + (1 | Genotype_Zone_1:Plant_Zone_1) +
(1 | Genotype_Zone_2:Plant_Zone_2)
   Data: data

REML criterion at convergence: 87.6

Scaled residuals:
    Min      1Q  Median      3Q     Max
-2.0763 -0.4646  0.1533  0.6384  1.4431

Random effects:
 Groups                       Name        Variance Std.Dev.
 Genotype_Zone_1:Plant_Zone_1 (Intercept) 0.000    0.000
 Genotype_Zone_2:Plant_Zone_2 (Intercept) 0.000    0.000
 Residual                                 1.712    1.309
Number of obs: 26, groups:  Genotype_Zone_1:Plant_Zone_1, 10; Genotype_Zone_2:Plant_Zone_2, 10

Fixed effects:
            Estimate Std. Error      df t value Pr(>|t|)
(Intercept)  -1.8883     0.2566 25.0000  -7.358 1.04e-07 ***
---
Signif. codes:  0 '***' 0.001 '**' 0.01 '*' 0.05 '.' 0.1 ' ' 1
```

Model residuals

Statistic	Value
Sample skewness	-0.7165
Sample excess kurtosis	0.2273
Passed Shapiro Wilk normality test	Yes (p-value = 0.061 > 0.05)

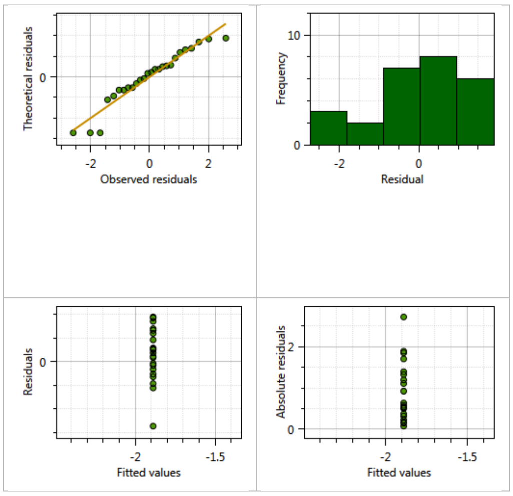

Analysis ratio halting to total trial duration (H6 - Zone 2)

**Analysis model**	Linear mixed model fit by REML: Ratio_halting_to_total_trial_duration_H6_Zone_2 ~ 1 + (1|Genotype_Zone_1:Plant_Zone_1) + (1|Genotype_Zone_2:Plant_Zone_2)
**Transformation**	Natural logarithm

Confidence intervals ratio halting to total trial duration (H6 - Zone 2)

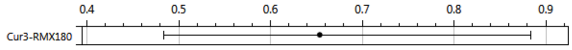

Confidence intervals ratio halting to total trial duration (H6 - Zone 2)

Genotype Zone 1	Genotype Zone 2	Mean	Lower 95% CL	Upper 95% CL	Group
Cur3	RMX180	0.6536	0.4833	0.884	a

Model summary

```
Linear mixed model fit by REML. t-tests use Satterthwaite's method ['lmerModLmerTest']
Formula: Ratio_halting_to_total_trial_duration_H6_Zone_2 ~ 1 + (1 | Genotype_Zone_1:Plant_Zone_1) +
(1 | Genotype_Zone_2:Plant_Zone_2)
   Data: data

REML criterion at convergence: 102.3

Scaled residuals:
    Min      1Q  Median      3Q     Max
-4.5511  0.1424  0.3050  0.4629  0.4629

Random effects:
 Groups                       Name        Variance Std.Dev.
 Genotype_Zone_1:Plant_Zone_1 (Intercept) 0.0000   0.0000
 Genotype_Zone_2:Plant_Zone_2 (Intercept) 0.0000   0.0000
 Residual                                 0.8435   0.9184
Number of obs: 38, groups:  Genotype_Zone_1:Plant_Zone_1, 10; Genotype_Zone_2:Plant_Zone_2, 10

Fixed effects:
            Estimate Std. Error      df t value Pr(>|t|)
(Intercept)  -0.4252     0.1490 37.0000  -2.854  0.00704 **
---
Signif. codes:  0 '***' 0.001 '**' 0.01 '*' 0.05 '.' 0.1 ' ' 1
```

Model residuals

Statistic	Value
Sample skewness	-3.542
Sample excess kurtosis	13.28
Passed Shapiro Wilk normality test	No (p-value = 2.8E-10 < 0.05)

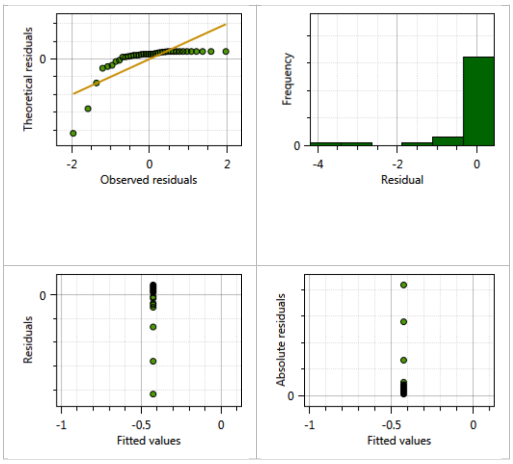

Analysis ratio halting to total trial duration (H7 - Zone 1)

**Analysis model**	Linear mixed model fit by REML: Ratio_halting_to_total_trial_duration_H7_Zone_1 ~ 1 + (1|Genotype_Zone_1:Plant_Zone_1) + (1|Genotype_Zone_2:Plant_Zone_2)
**Transformation**	Natural logarithm

Confidence intervals ratio halting to total trial duration (H7 - Zone 1)

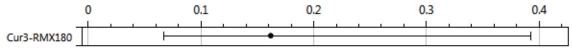

Confidence intervals ratio halting to total trial duration (H7 - Zone 1)

Genotype Zone 1	Genotype Zone 2	Mean	Lower 95% CL	Upper 95% CL	Group
Cur3	RMX180	0.1618	0.06669	0.3927	a

Model summary

```
Linear mixed model fit by REML. t-tests use Satterthwaite's method ['lmerModLmerTest']
Formula: Ratio_halting_to_total_trial_duration_H7_Zone_1 ~ 1 + (1 | Genotype_Zone_1:Plant_Zone_1) +
(1 | Genotype_Zone_2:Plant_Zone_2)
   Data: data

REML criterion at convergence: 94.7

Scaled residuals:
    Min      1Q  Median      3Q     Max
-1.6714 -0.7657  0.3403  0.8563  1.1057

Random effects:
 Groups                       Name        Variance Std.Dev.
 Genotype_Zone_2:Plant_Zone_2 (Intercept) 0.42897  0.6550
 Genotype_Zone_1:Plant_Zone_1 (Intercept) 0.01083  0.1041
 Residual                                 1.93498  1.3910
Number of obs: 26, groups:  Genotype_Zone_2:Plant_Zone_2, 10; Genotype_Zone_1:Plant_Zone_1, 9

Fixed effects:
            Estimate Std. Error      df t value Pr(>|t|)
(Intercept)  -1.8212     0.3539  5.4752  -5.146  0.00279 **
---
Signif. codes:  0 '***' 0.001 '**' 0.01 '*' 0.05 '.' 0.1 ' ' 1
```

Model residuals

Statistic	Value
Sample skewness	-0.5369
Sample excess kurtosis	-1.199
Passed Shapiro Wilk normality test	No (p-value = 0.006784 < 0.05)

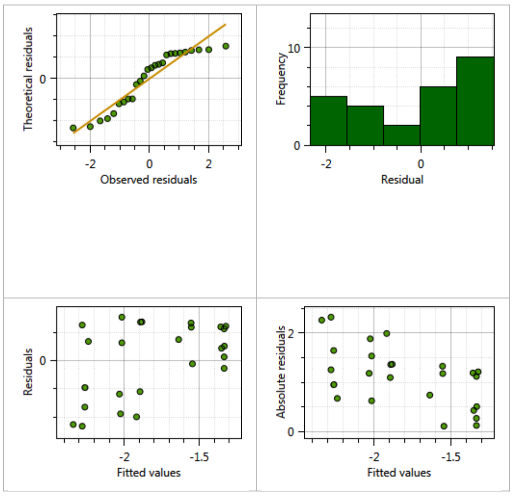

Analysis ratio halting to total trial duration (H7 - Zone 2)

**Analysis model**	Linear mixed model fit by REML: Ratio_halting_to_total_trial_duration_H7_Zone_2 ~ 1 + (1|Genotype_Zone_1:Plant_Zone_1) + (1|Genotype_Zone_2:Plant_Zone_2)
**Transformation**	Natural logarithm

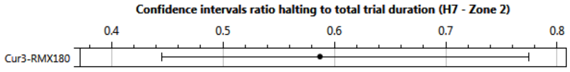

Confidence intervals ratio halting to total trial duration (H7 - Zone 2)

Genotype Zone 1	Genotype Zone 2	Mean	Lower 95% CL	Upper 95% CL	Group
Cur3	RMX180	0.587	0.4447	0.7748	a

Model summary

```
Linear mixed model fit by REML. t-tests use Satterthwaite's method ['lmerModLmerTest']
Formula: Ratio_halting_to_total_trial_duration_H7_Zone_2 ~ 1 + (1 | Genotype_Zone_1:Plant_Zone_1) +
(1 | Genotype_Zone_2:Plant_Zone_2)
   Data: data

REML criterion at convergence: 89.2

Scaled residuals:
    Min      1Q  Median      3Q     Max
-2.8366 -0.4133  0.4918  0.6876  0.7563

Random effects:
 Groups                       Name        Variance  Std.Dev.
 Genotype_Zone_1:Plant_Zone_1 (Intercept) 2.937e-16 1.714e-08
 Genotype_Zone_2:Plant_Zone_2 (Intercept) 8.749e-03 9.354e-02
 Residual                                 5.478e-01 7.401e-01
Number of obs: 39, groups:  Genotype_Zone_1:Plant_Zone_1, 10; Genotype_Zone_2:Plant_Zone_2, 10

Fixed effects:
            Estimate Std. Error      df t value Pr(>|t|)
(Intercept)  -0.5327     0.1222  8.7561  -4.361  0.00194 **
---
Signif. codes:  0 '***' 0.001 '**' 0.01 '*' 0.05 '.' 0.1 ' ' 1
```

Model residuals

Statistic	Value
Sample skewness	-1.507
Sample excess kurtosis	1.405
Passed Shapiro Wilk normality test	No (p-value = 1.095E-06 < 0.05)

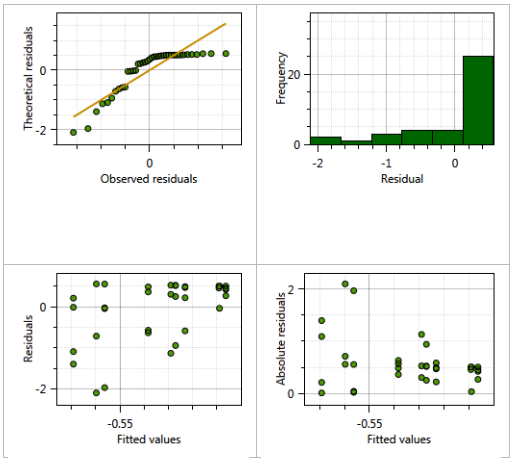

Analysis ratio halting to total trial duration H0 (diff. Zone 1 - Zone 2)

**Analysis model**	Generalized linear mixed model with dispersion factor, formula=cbind(Ratio_halting_to_total_trial_duration_H0_Zone_1,Ratio_halting_to_total_trial_duration_H0_Zone_2) ~ 1 + (1|Genotype_Zone_1:Plant_Zone_1) + (1|Genotype_Zone_2:Plant_Zone_2)
**Transformation**	Logit

Confidence intervals ratio halting to total trial duration H0 (diff. Zone 1 - Zone 2)

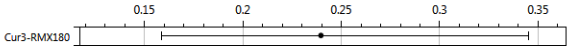

Confidence intervals ratio halting to total trial duration H0 (diff. Zone 1 - Zone 2)

Genotype Zone 1	Genotype Zone 2	Mean	Lower 95% CL	Upper 95% CL	Group
Cur3	RMX180	0.2397	0.1585	0.3453	a

Model summary

```
Linear mixed model fit by REML. t-tests use Satterthwaite's method ['lmerModLmerTest']
Formula: ziFormula
   Data: data
Weights: wi

REML criterion at convergence: 146.7

Scaled residuals:
    Min      1Q  Median      3Q     Max
-0.8397 -0.7848 -0.3492  0.3742  2.6638

Random effects:
 Groups                      Name        Variance  Std.Dev.
 Genotype_Zone_1:Plant_Zone_1 (Intercept) 3.620e-17 6.017e-09
 Genotype_Zone_2:Plant_Zone_2 (Intercept) 0.000e+00 0.000e+00
 Residual                                4.471e-01 6.686e-01
Number of obs: 39, groups:  Genotype_Zone_1:Plant_Zone_1, 10; Genotype_Zone_2:Plant_Zone_2, 10

Fixed effects:
            Estimate Std. Error      df t value Pr(>|t|)
(Intercept)  -1.1545     0.2542 38.0000  -4.542 5.49e-05 ***
---
Signif. codes:  0 '***' 0.001 '**' 0.01 '*' 0.05 '.' 0.1 ' ' 1

Dispersion: 0.6686
```

Model residuals

Statistic	Value
Sample skewness	1.415
Sample excess kurtosis	1.149
Passed Shapiro Wilk normality test	No (p-value = 8.499E-06 < 0.05)

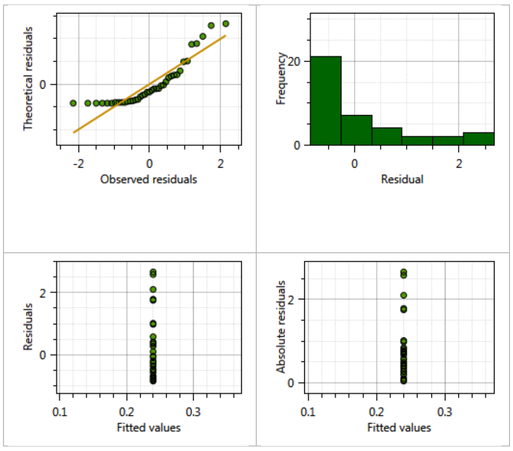

Analysis ratio halting to total trial duration H1 (diff. Zone 1 - Zone 2)

**Analysis model**	Generalized linear mixed model with dispersion factor, formula=cbind(Ratio_halting_to_total_trial_duration_H1_Zone_1,Ratio_halting_to_total_trial_duration_H1_Zone_2) ~ 1 + (1|Genotype_Zone_1:Plant_Zone_1) + (1|Genotype_Zone_2:Plant_Zone_2)
**Transformation**	Logit

Confidence intervals ratio halting to total trial duration H1 (diff. Zone 1 - Zone 2)

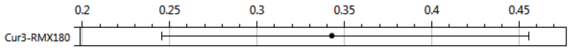

Confidence intervals ratio halting to total trial duration H1 (diff. Zone 1 - Zone 2)

Genotype Zone 1	Genotype Zone 2	Mean	Lower 95% CL	Upper 95% CL	Group
Cur3	RMX180	0.3427	0.2452	0.4556	a

Model summary

```
Linear mixed model fit by REML. t-tests use Satterthwaite's method ['lmerModLmerTest']
Formula: ziFormula
   Data: data
```

```
Weights: wi

REML criterion at convergence: 140.4

Scaled residuals:
    Min      1Q  Median      3Q     Max
-1.0617 -0.8728 -0.2061  0.7639  2.0361

Random effects:
 Groups                      Name        Variance Std.Dev.
 Genotype_Zone_1:Plant_Zone_1 (Intercept) 0.0000   0.0000
 Genotype_Zone_2:Plant_Zone_2 (Intercept) 0.0000   0.0000
 Residual                                0.4626   0.6801
Number of obs: 39, groups:  Genotype_Zone_1:Plant_Zone_1, 10; Genotype_Zone_2:Plant_Zone_2, 10

Fixed effects:
            Estimate Std. Error      df t value Pr(>|t|)
(Intercept)  -0.6512     0.2337 38.0000  -2.787  0.00827 **
---
Signif. codes:  0 '***' 0.001 '**' 0.01 '*' 0.05 '.' 0.1 ' ' 1

Dispersion: 0.6801
```

Model residuals

Statistic	Value
Sample skewness	0.6227
Sample excess kurtosis	-0.9676
Passed Shapiro Wilk normality test	No (p-value = 0.0005821 < 0.05)

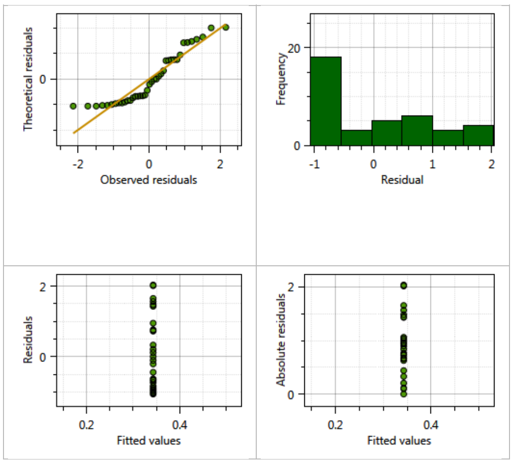

Analysis ratio halting to total trial duration H2 (diff. Zone 1 - Zone 2)

**Analysis model**	Generalized linear mixed model with dispersion factor, formula=cbind(Ratio_halting_to_total_trial_duration_H2_Zone_1,Ratio_halting_to_total_trial_duration_H2_Zone_2) ~ 1 + (1|Genotype_Zone_1:Plant_Zone_1) + (1|Genotype_Zone_2:Plant_Zone_2)
**Transformation**	Logit

Confidence intervals ratio halting to total trial duration H2 (diff. Zone 1 - Zone 2)

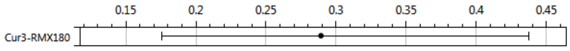

Confidence intervals ratio halting to total trial duration H2 (diff. Zone 1 - Zone 2)

Genotype Zone 1	Genotype Zone 2	Mean	Lower 95% CL	Upper 95% CL	Group
Cur3	RMX180	0.2892	0.1754	0.4376	a

Model summary

```
Linear mixed model fit by REML. t-tests use Satterthwaite's method ['lmerModLmerTest']
Formula: ziFormula
   Data: data
Weights: wi

REML criterion at convergence: 147

Scaled residuals:
    Min      1Q  Median      3Q     Max
-1.1140 -0.7559 -0.3947  0.2989  2.0822

Random effects:
 Groups                      Name        Variance  Std.Dev.
 Genotype_Zone_1:Plant_Zone_1 (Intercept) 1.984e-01 4.454e-01
 Genotype_Zone_2:Plant_Zone_2 (Intercept) 8.423e-16 2.902e-08
 Residual                                4.578e-01 6.766e-01
Number of obs: 39, groups:  Genotype_Zone_1:Plant_Zone_1, 10; Genotype_Zone_2:Plant_Zone_2, 10

Fixed effects:
            Estimate Std. Error     df t value Pr(>|t|)
(Intercept)  -0.8994     0.2833  8.3540  -3.175   0.0124 *
---
Signif. codes:  0 '***' 0.001 '**' 0.01 '*' 0.05 '.' 0.1 ' ' 1

Dispersion: 0.6766
```

Model residuals

Statistic	Value
Sample skewness	1.056
Sample excess kurtosis	-0.1525
Passed Shapiro Wilk normality test	No (p-value = 4.6E-05 < 0.05)

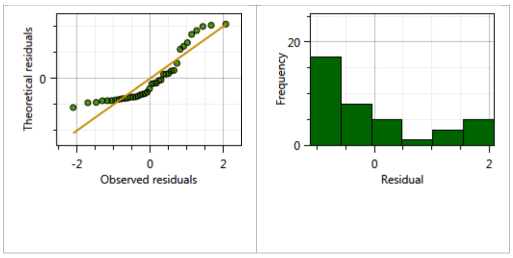

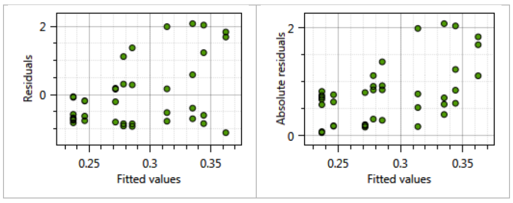

Analysis ratio halting to total trial duration H3 (diff. Zone 1 - Zone 2)

**Analysis model**	Generalized linear mixed model with dispersion factor, formula=cbind(Ratio_halting_to_total_trial_duration_H3_Zone_1,Ratio_halting_to_total_trial_duration_H3_Zone_2) ~ 1 + (1|Genotype_Zone_1:Plant_Zone_1) + (1|Genotype_Zone_2:Plant_Zone_2)
**Transformation**	Logit

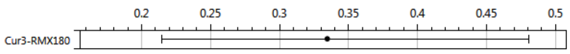

Confidence intervals ratio halting to total trial duration H3 (diff. Zone 1 - Zone 2)

Genotype Zone 1	Genotype Zone 2	Mean	Lower 95% CL	Upper 95% CL	Group
Cur3	RMX180	0.3346	0.2144	0.4808	a

Model summary

```
Linear mixed model fit by REML. t-tests use Satterthwaite's method ['lmerModLmerTest']
Formula: ziFormula
   Data: data
Weights: wi

REML criterion at convergence: 140.3

Scaled residuals:
    Min      1Q  Median      3Q     Max
-1.2113 -0.9132 -0.1675  0.8075  1.8966

Random effects:
 Groups                      Name        Variance  Std.Dev.
 Genotype_Zone_1:Plant_Zone_1 (Intercept) 5.262e-16 2.294e-08
 Genotype_Zone_2:Plant_Zone_2 (Intercept) 2.220e-01 4.711e-01
 Residual                                 4.158e-01 6.448e-01
Number of obs: 39, groups:  Genotype_Zone_1:Plant_Zone_1, 10; Genotype_Zone_2:Plant_Zone_2, 10

Fixed effects:
            Estimate Std. Error      df t value Pr(>|t|)
(Intercept)  -0.6876     0.2691  8.7996  -2.555   0.0315 *
---
Signif. codes:  0 '***' 0.001 '**' 0.01 '*' 0.05 '.' 0.1 ' ' 1

Dispersion: 0.6448
```

Model residuals

Statistic	Value
Sample skewness	0.4867
Sample excess kurtosis	-1.197
Passed Shapiro Wilk normality test	No (p-value = 0.001491 < 0.05)

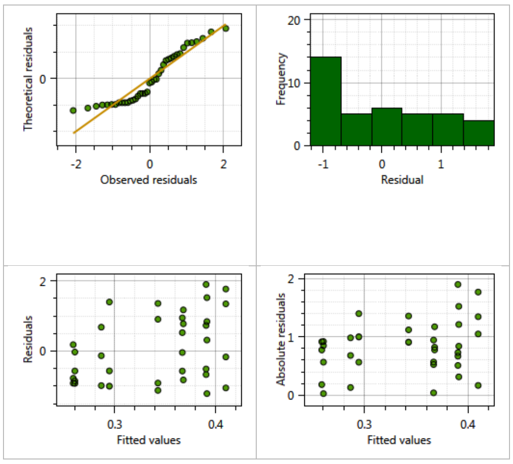

Analysis ratio halting to total trial duration H4 (diff. Zone 1 - Zone 2)

**Analysis model**	Generalized linear mixed model with dispersion factor, formula=cbind(Ratio_halting_to_total_trial_duration_H4_Zone_1,Ratio_halting_to_total_trial_duration_H4_Zone_2) ~ 1 + (1|Genotype_Zone_1:Plant_Zone_1) + (1|Genotype_Zone_2:Plant_Zone_2)
**Transformation**	Logit

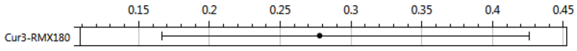

Confidence intervals ratio halting to total trial duration H4 (diff. Zone 1 - Zone 2)

Genotype Zone 1	Genotype Zone 2	Mean	Lower 95% CL	Upper 95% CL	Group
Cur3	RMX180	0.2778	0.1662	0.4261	a

Model summary

```
Linear mixed model fit by REML. t-tests use Satterthwaite's method ['lmerModLmerTest']
Formula: ziFormula
   Data: data
Weights: wi

REML criterion at convergence: 150.4

Scaled residuals:
    Min      1Q  Median      3Q     Max
-0.8852 -0.7531 -0.4046  0.7761  2.1739

Random effects:
 Groups                      Name        Variance  Std.Dev.
 Genotype_Zone_1:Plant_Zone_1 (Intercept) 1.185e-15 3.443e-08
 Genotype_Zone_2:Plant_Zone_2 (Intercept) 1.518e-01 3.896e-01
 Residual                                 5.205e-01 7.215e-01
Number of obs: 39, groups:  Genotype_Zone_1:Plant_Zone_1, 10; Genotype_Zone_2:Plant_Zone_2, 10

Fixed effects:
            Estimate Std. Error      df t value Pr(>|t|)
```

```
(Intercept)  -0.9555     0.2884  8.5621  -3.313  0.00968 **
---
Signif. codes:  0 '***' 0.001 '**' 0.01 '*' 0.05 '.' 0.1 ' ' 1

Dispersion: 0.7215
```

Model residuals

Statistic	Value
Sample skewness	1.072
Sample excess kurtosis	-0.2959
Passed Shapiro Wilk normality test	No (p-value = 6.873E-06 < 0.05)

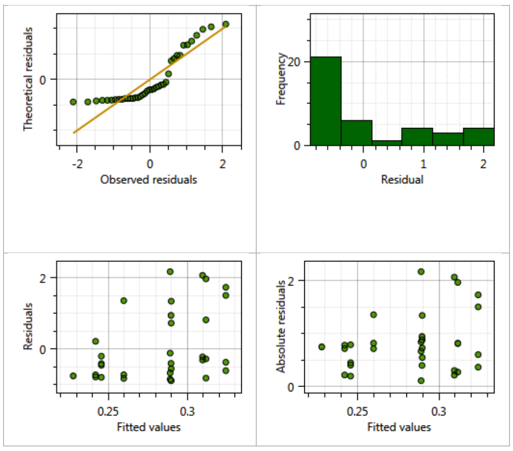

Analysis ratio halting to total trial duration H5 (diff. Zone 1 - Zone 2)

**Analysis model**	Generalized linear mixed model with dispersion factor, formula=cbind(Ratio_halting_to_total_trial_duration_H5_Zone_1,Ratio_halting_to_total_trial_duration_H5_Zone_2) ~ 1 + (1|Genotype_Zone_1:Plant_Zone_1) + (1|Genotype_Zone_2:Plant_Zone_2)
**Transformation**	Logit

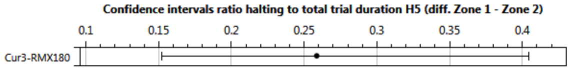

Confidence intervals ratio halting to total trial duration H5 (diff. Zone 1 - Zone 2)

Genotype Zone 1	Genotype Zone 2	Mean	Lower 95% CL	Upper 95% CL	Group
Cur3	RMX180	0.2586	0.1519	0.4046	a

Model summary

```
Linear mixed model fit by REML. t-tests use Satterthwaite's method ['lmerModLmerTest']
Formula: ziFormula
   Data: data
Weights: wi

REML criterion at convergence: 150.7

Scaled residuals:
    Min      1Q  Median      3Q     Max
-0.9826 -0.7569 -0.5539  0.5572  2.2565

Random effects:
 Groups                      Name        Variance Std.Dev.
 Genotype_Zone_1:Plant_Zone_1 (Intercept) 0.1931   0.4394
 Genotype_Zone_2:Plant_Zone_2 (Intercept) 0.0000   0.0000
 Residual                                 0.4885   0.6990
Number of obs: 39, groups:  Genotype_Zone_1:Plant_Zone_1, 10; Genotype_Zone_2:Plant_Zone_2, 10

Fixed effects:
            Estimate Std. Error      df t value Pr(>|t|)
(Intercept)  -1.0533     0.2948  9.0019  -3.573    0.006 **
---
Signif. codes:  0 '***' 0.001 '**' 0.01 '*' 0.05 '.' 0.1 ' ' 1

Dispersion: 0.699
```

Model residuals

Statistic	Value
Sample skewness	0.9853
Sample excess kurtosis	-0.2973
Passed Shapiro Wilk normality test	No (p-value = 2.555E-05 < 0.05)

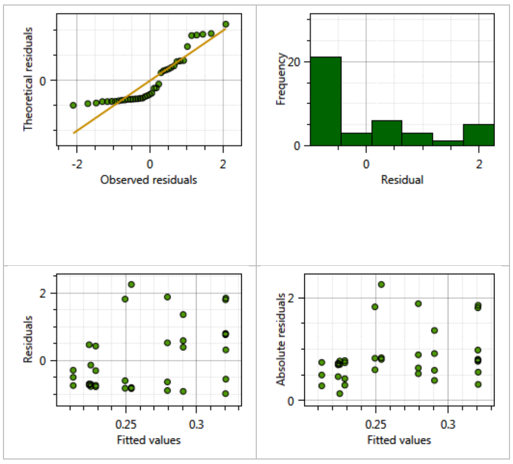

Analysis ratio halting to total trial duration H6 (diff. Zone 1 - Zone 2)

**Analysis model**	Generalized linear mixed model with dispersion factor, formula=cbind(Ratio_halting_to_total_trial_duration_H6_Zone_1,Ratio_halting_to_total_trial_duration_H6_Zone_2) ~ 1 + (1|Genotype_Zone_1:Plant_Zone_1) + (1|Genotype_Zone_2:Plant_Zone_2)
**Transformation**	Logit

Confidence intervals ratio halting to total trial duration H6 (diff. Zone 1 - Zone 2)

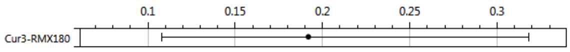

Confidence intervals ratio halting to total trial duration H6 (diff. Zone 1 - Zone 2)

Genotype Zone 1	Genotype Zone 2	Mean	Lower 95% CL	Upper 95% CL	Group
Cur3	RMX180	0.1918	0.1077	0.3183	a

Model summary

```
Linear mixed model fit by REML. t-tests use Satterthwaite's method ['lmerModLmerTest']
Formula: ziFormula
   Data: data
Weights: wi

REML criterion at convergence: 153

Scaled residuals: 
    Min      1Q  Median      3Q     Max 
-0.9864 -0.6962 -0.4308  0.2813  2.6698 

Random effects:
 Groups                      Name        Variance Std.Dev.
 Genotype_Zone_1:Plant_Zone_1 (Intercept) 0.0000   0.0000  
 Genotype_Zone_2:Plant_Zone_2 (Intercept) 0.2309   0.4805  
 Residual                                0.3952   0.6287  
Number of obs: 39, groups:  Genotype_Zone_1:Plant_Zone_1, 10; Genotype_Zone_2:Plant_Zone_2, 10

Fixed effects:
            Estimate Std. Error      df t value Pr(>|t|)    
(Intercept)  -1.4381     0.3028  9.8326  -4.749 0.000819 ***
---
Signif. codes:  0 '***' 0.001 '**' 0.01 '*' 0.05 '.' 0.1 ' ' 1

Dispersion: 0.6287
```

Model residuals

Statistic	Value
Sample skewness	1.431
Sample excess kurtosis	1.315
Passed Shapiro Wilk normality test	No (p-value = 1.59E-05 < 0.05)

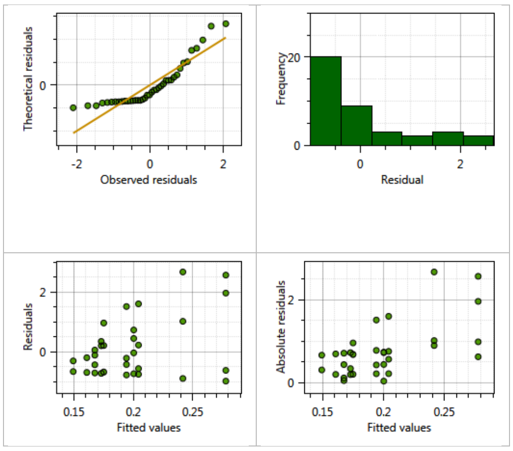

Analysis ratio halting to total trial duration H7 (diff. Zone 1 - Zone 2)

**Analysis model**	Generalized linear mixed model with dispersion factor, formula=cbind(Ratio_halting_to_total_trial_duration_H7_Zone_1,Ratio_halting_to_total_trial_duration_H7_Zone_2) ~ 1 + (1|Genotype_Zone_1:Plant_Zone_1) + (1|Genotype_Zone_2:Plant_Zone_2)
**Transformation**	Logit

Confidence intervals ratio halting to total trial duration H7 (diff. Zone 1 - Zone 2)

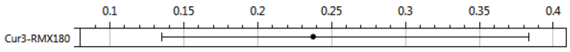

Confidence intervals ratio halting to total trial duration H7 (diff. Zone 1 - Zone 2)

Genotype Zone 1	Genotype Zone 2	Mean	Lower 95% CL	Upper 95% CL	Group
Cur3	RMX180	0.2375	0.1348	0.3836	a

Model summary

```
Linear mixed model fit by REML. t-tests use Satterthwaite's method ['lmerModLmerTest']
Formula: ziFormula
   Data: data
Weights: wi

REML criterion at convergence: 152.5

Scaled residuals: 
    Min      1Q  Median      3Q     Max 
-1.0109 -0.7510 -0.5683  0.6351  2.0049 

Random effects:
 Groups                      Name        Variance Std.Dev.
 Genotype_Zone_1:Plant_Zone_1 (Intercept) 0.0000   0.0000  
 Genotype_Zone_2:Plant_Zone_2 (Intercept) 0.2833   0.5323  
 Residual                                0.4327   0.6578  
Number of obs: 39, groups:  Genotype_Zone_1:Plant_Zone_1, 10; Genotype_Zone_2:Plant_Zone_2, 10

Fixed effects:
            Estimate Std. Error      df t value Pr(>|t|)   
(Intercept)  -1.1665     0.3073  9.2471  -3.796  0.00404 **
---
Signif. codes:  0 '***' 0.001 '**' 0.01 '*' 0.05 '.' 0.1 ' ' 1

Dispersion: 0.6578
```

Model residuals

Statistic	Value
Sample skewness	0.9396
Sample excess kurtosis	-0.6459
Passed Shapiro Wilk normality test	No (p-value = 1.075E-05 < 0.05)

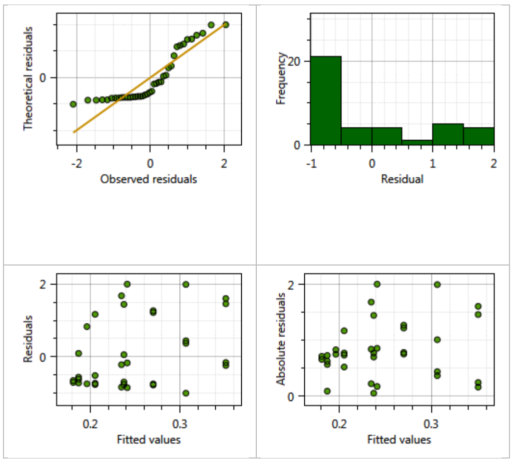

Ratio movement to detection duration per zone

Selected zones	Zone 1, Zone 2
Data transformation	Natural logarithm
Analysis	Zone difference analysis

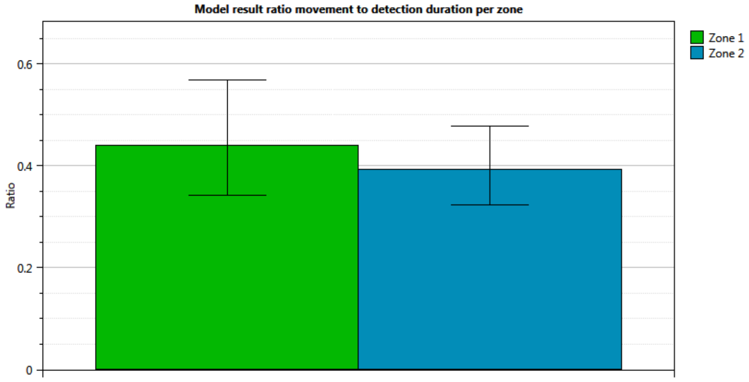

Results difference tests Zone 1 - Zone 2: p values and 95% confidence intervals of the difference on the transformed scale for each statistic.

Behaviour statistic	Cur3-RMX180
Ratio movement to detection duration (diff. Zone 1 - Zone 2)	p=0.00335**
	[0.0655, 0.305]

The model predictions and 95% confidence intervals for each statistic.

Statistic	Cur3-RMX180	Remark
Ratio movement to detection duration (Zone 1)	0.441	CR
	[0.342, 0.569]	
Ratio movement to detection duration (Zone 2)	0.393	CR
	[0.323, 0.479]	

CR = Check residuals

Data summary

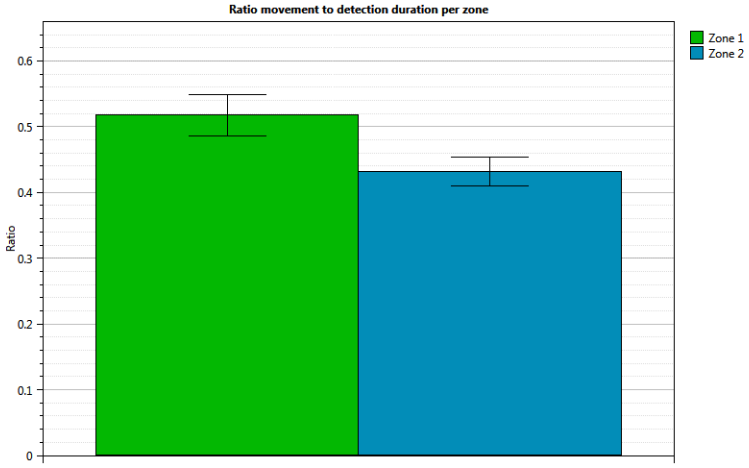

Genotype Zone 1	Genotype Zone 2	Genotype Zone 3	Mean Zone 1	StdErr Zone 1	Mean Zone 2	StdErr Zone 2
Cur3	RMX180	Neutral	0.52	0.03	0.43	0.02

Analysis ratio movement to detection duration (Zone 1)

**Analysis model**	Linear mixed model fit by REML: Ratio_movement_to_detection_duration_Zone_1 ~ 1 + (1|Genotype_Zone_1:Plant_Zone_1) + (1|Genotype_Zone_2:Plant_Zone_2)
**Transformation**	Natural logarithm

Confidence intervals ratio movement to detection duration (Zone 1)

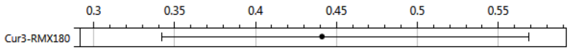

Confidence intervals ratio movement to detection duration (Zone 1)

Genotype Zone 1	Genotype Zone 2	Mean	Lower 95% CL	Upper 95% CL	Group
Cur3	RMX180	0.441	0.3418	0.569	a

Model summary

```
Linear mixed model fit by REML. t-tests use Satterthwaite's method ['lmerModLmerTest']
Formula: Ratio_movement_to_detection_duration_Zone_1 ~ 1 + (1 | Genotype_Zone_1:Plant_Zone_1) +      (1 |
Genotype_Zone_2:Plant_Zone_2)
   Data: data

REML criterion at convergence: 93.2

Scaled residuals: 
    Min      1Q  Median      3Q     Max 
-4.8166 -0.1277  0.3264  0.4928  0.8347 

Random effects:
 Groups                       Name        Variance  Std.Dev. 
 Genotype_Zone_1:Plant_Zone_1 (Intercept) 6.512e-16 2.552e-08
 Genotype_Zone_2:Plant_Zone_2 (Intercept) 0.000e+00 0.000e+00
 Residual                                 6.180e-01 7.861e-01
Number of obs: 39, groups:  Genotype_Zone_1:Plant_Zone_1, 10; Genotype_Zone_2:Plant_Zone_2, 10

Fixed effects:
            Estimate Std. Error      df t value Pr(>|t|)    
(Intercept)  -0.8187     0.1259 38.0000  -6.504 1.16e-07 ***
---
Signif. codes:  0 '***' 0.001 '**' 0.01 '*' 0.05 '.' 0.1 ' ' 1
```

Model residuals

Statistic	Value
Sample skewness	-3.427
Sample excess kurtosis	14.34
Passed Shapiro Wilk normality test	No (p-value = 1.01E-08 < 0.05)

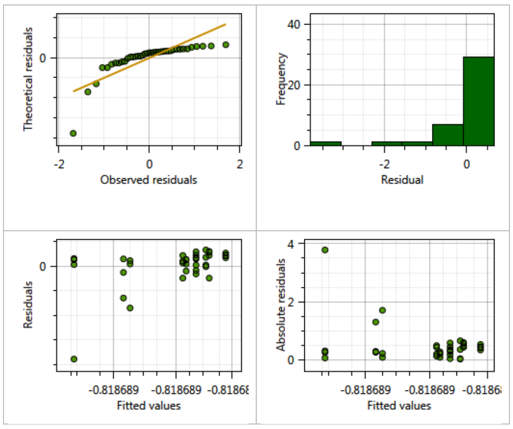

Analysis ratio movement to detection duration (Zone 2)

**Analysis model**	Linear mixed model fit by REML: Ratio_movement_to_detection_duration_Zone_2 ~ 1 + (1|Genotype_Zone_1:Plant_Zone_1) + (1|Genotype_Zone_2:Plant_Zone_2)
**Transformation**	Natural logarithm

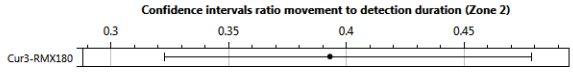

Confidence intervals ratio movement to detection duration (Zone 2)

Genotype Zone 1	Genotype Zone 2	Mean	Lower 95% CL	Upper 95% CL	Group
Cur3	RMX180	0.3931	0.3227	0.4788	a

Model summary

```
Linear mixed model fit by REML. t-tests use Satterthwaite's method ['lmerModLmerTest']
Formula: Ratio_movement_to_detection_duration_Zone_2 ~ 1 + (1 | Genotype_Zone_1:Plant_Zone_1) +      (1 |
Genotype_Zone_2:Plant_Zone_2)
   Data: data

REML criterion at convergence: 61.9

Scaled residuals: 
    Min      1Q  Median      3Q     Max 
-3.9425 -0.0516  0.2745  0.4943  1.0883 

Random effects:
 Groups                       Name        Variance  Std.Dev. 
 Genotype_Zone_1:Plant_Zone_1 (Intercept) 1.940e-16 1.393e-08
 Genotype_Zone_2:Plant_Zone_2 (Intercept) 2.802e-03 5.293e-02
 Residual                                 2.684e-01 5.181e-01
Number of obs: 39, groups:  Genotype_Zone_1:Plant_Zone_1, 10; Genotype_Zone_2:Plant_Zone_2, 10

Fixed effects:
            Estimate Std. Error       df t value Pr(>|t|)    
(Intercept) -0.93379    0.08464  7.51787  -11.03 6.55e-06 ***
---
Signif. codes:  0 '***' 0.001 '**' 0.01 '*' 0.05 '.' 0.1 ' ' 1
```

Model residuals

Statistic	Value
Sample skewness	-2.627
Sample excess kurtosis	7.437
Passed Shapiro Wilk normality test	No (p-value = 6.637E-08 < 0.05)

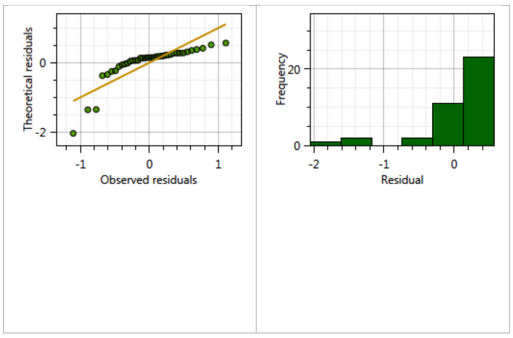

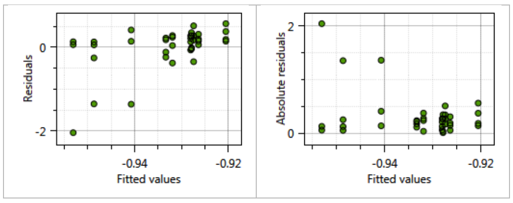

Analysis ratio movement to detection duration (diff. Zone 1 - Zone 2)

**Analysis model**	Generalized linear mixed model with dispersion factor, formula=cbind(Ratio_movement_to_detection_duration_Zone_1,Ratio_movement_to_detection_duration_Zone_2) ~ 1 + (1|Genotype_Zone_1:Plant_Zone_1) + (1|Genotype_Zone_2:Plant_Zone_2)
**Transformation**	Logit

Confidence intervals ratio movement to detection duration (diff. Zone 1 - Zone 2)

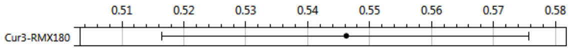

Confidence intervals ratio movement to detection duration (diff. Zone 1 - Zone 2)

Genotype Zone 1	Genotype Zone 2	Mean	Lower 95% CL	Upper 95% CL	Group
Cur3	RMX180	0.5462	0.5164	0.5757	a

Model summary

```
Linear mixed model fit by REML. t-tests use Satterthwaite's method ['lmerModLmerTest']
Formula: ziFormula
   Data: data
Weights: wi

REML criterion at convergence: 39.6

Scaled residuals:
     Min       1Q   Median       3Q      Max
-2.95413 -0.41781 -0.01872  0.56486  2.26367

Random effects:
 Groups                      Name        Variance Std.Dev.
 Genotype_Zone_1:Plant_Zone_1 (Intercept) 0.00000  0.0000
 Genotype_Zone_2:Plant_Zone_2 (Intercept) 0.00000  0.0000
 Residual                                 0.03218  0.1794
Number of obs: 39, groups:  Genotype_Zone_1:Plant_Zone_1, 10; Genotype_Zone_2:Plant_Zone_2, 10

Fixed effects:
            Estimate Std. Error       df t value Pr(>|t|)
(Intercept)  0.18539    0.05923 38.00000    3.13  0.00335 **
---
Signif. codes:  0 '***' 0.001 '**' 0.01 '*' 0.05 '.' 0.1 ' ' 1

Dispersion: 0.1794
```

Model residuals

Statistic	Value
Sample skewness	-0.5259
Sample excess kurtosis	1.154
Passed Shapiro Wilk normality test	Yes (p-value = 0.2331 > 0.05)

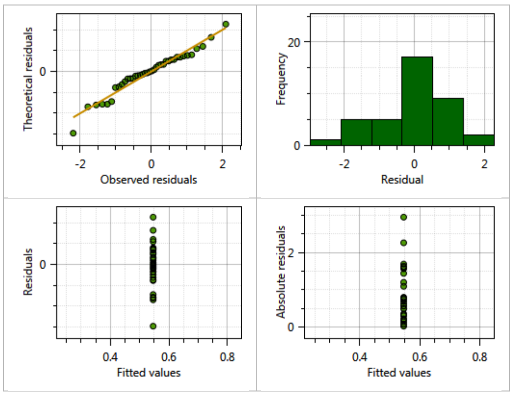

Ratio movement to detection duration per zone per hour

Selected hours	0, 1, 2, 3, 4, 5, 6, 7
Selected zones	Zone 1, Zone 2
Data transformation	Natural logarithm
Analysis	Zone difference analysis

Ratio movement to detection duration per zone per hour

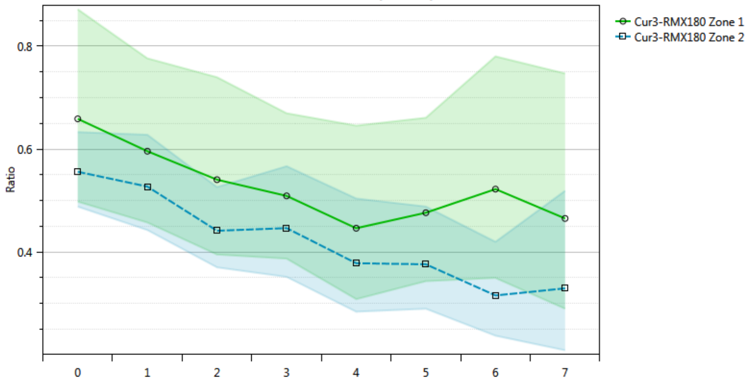

Results difference tests Zone 1 - Zone 2: p values and 95% confidence intervals of the difference on the transformed scale for each statistic.

Behaviour statistic	Cur3-RMX180	Remark
Ratio movement to detection duration H0 (diff. Zone 1 - Zone 2)	p=0.0076** [0.0614, 0.37]	CR

Behaviour statistic	Cur3-RMX180	Remark
Ratio movement to detection duration H1 (diff. Zone 1 - Zone 2)	p=0.0233*<br>[0.0254, 0.327]	
Ratio movement to detection duration H2 (diff. Zone 1 - Zone 2)	p=0.00569**<br>[0.0795, 0.433]	
Ratio movement to detection duration H3 (diff. Zone 1 - Zone 2)	p=0.844<br>[-0.22, 0.181]	
Ratio movement to detection duration H4 (diff. Zone 1 - Zone 2)	p=0.166<br>[-0.0866, 0.429]	
Ratio movement to detection duration H5 (diff. Zone 1 - Zone 2)	p=0.367<br>[-0.19, 0.464]	
Ratio movement to detection duration H6 (diff. Zone 1 - Zone 2)	p=0.0353*<br>[0.0221, 0.576]	
Ratio movement to detection duration H7 (diff. Zone 1 - Zone 2)	p=0.441<br>[-0.251, 0.51]	

CR = Check residuals

The model predictions and 95% confidence intervals for each statistic.

Statistic	Cur3-RMX180	Remark
Ratio movement to detection duration (H0 - Zone 1)	0.659<br>[0.498, 0.872]	CR
Ratio movement to detection duration (H0 - Zone 2)	0.556<br>[0.488, 0.633]	
Ratio movement to detection duration (H1 - Zone 1)	0.596<br>[0.457, 0.776]	CR
Ratio movement to detection duration (H1 - Zone 2)	0.527<br>[0.443, 0.628]	CR
Ratio movement to detection duration (H2 - Zone 1)	0.541<br>[0.395, 0.74]	CR
Ratio movement to detection duration (H2 - Zone 2)	0.441<br>[0.37, 0.526]	CR
Ratio movement to detection duration (H3 - Zone 1)	0.509<br>[0.387, 0.67]	
Ratio movement to detection duration (H3 - Zone 2)	0.446<br>[0.352, 0.567]	CR
Ratio movement to detection duration (H4 - Zone 1)	0.446<br>[0.308, 0.646]	CR
Ratio movement to detection duration (H4 - Zone 2)	0.378<br>[0.284, 0.504]	CR
Ratio movement to detection duration (H5 - Zone 1)	0.476<br>[0.343, 0.661]	CR
Ratio movement to detection duration (H5 - Zone 2)	0.376<br>[0.29, 0.488]	CR
Ratio movement to detection duration (H6 - Zone 1)	0.522<br>[0.35, 0.78]	CR
Ratio movement to detection duration (H6 - Zone 2)	0.316<br>[0.237, 0.42]	CR
Ratio movement to detection duration (H7 - Zone 1)	0.465<br>[0.29, 0.747]	CR
Ratio movement to detection duration (H7 - Zone 2)	0.329<br>[0.209, 0.519]	CR

CR = Check residuals

Data summary

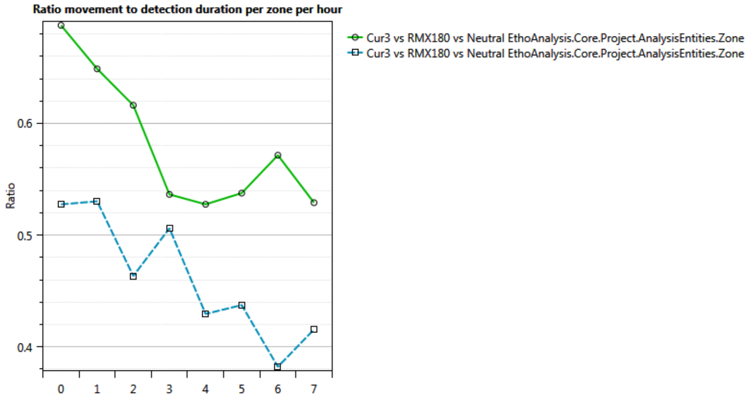

Genotype Zone 1	Genotype Zone 2	Genotype Zone 3	Mean H0 - Zone 1	StdErr H0 - Zone 1	Mean H0 - Zone 2	StdErr H0 - Zone 2	Mean H1 - Zone 1	StdErr H1 - Zone 1	Mean H1 - Zone 2	StdErr H1 - Zone 2	Mean H2 - Zone 1	StdErr H2 - Zone 1	Mean H2 - Zone 2	StdErr H2 - Zone 2	Mean H3 - Zone 1	StdErr H3 - Zone 1	Mean H3 - Zone 2	StdErr H3 - Zone 2	Mean H4 - Zone 1	StdErr H4 - Zone 1	Mean H4 - Zone 2	StdErr H4 - Zone 2	Mean H5 - Zone 1	StdErr H5 - Zone 1	Mean H5 - Zone 2	StdErr H5 - Zone 2	Mean H6 - Zone 1	StdErr H6 - Zone 1	Mean H6 - Zone 2	StdErr H6 - Zone 2
Cur3	RMX180	Neutral	0.69	0.04	0.53	0.03	0.65	0.04	0.53	0.03	0.62	0.05	0.46	0.03	0.54	0.05	0.51	0.03	0.53	0.05	0.43	0.03	0.54	0.05	0.44	0.03	0.57	0.06	0.38	0.03

Analysis ratio movement to detection duration (H0 - Zone 1)

**Analysis model**	Linear mixed model fit by REML: Ratio_movement_to_detection_duration_H0_Zone_1 ~ 1 + (1|Genotype_Zone_1:Plant_Zone_1) + (1|Genotype_Zone_2:Plant_Zone_2)
**Transformation**	Natural logarithm

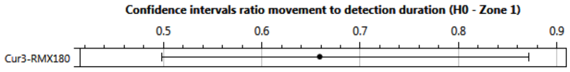

Confidence intervals ratio movement to detection duration (H0 - Zone 1)

Genotype Zone 1	Genotype Zone 2	Mean	Lower 95% CL	Upper 95% CL	Group
Cur3	RMX180	0.6588	0.4979	0.8717	a

Model summary

```
Linear mixed model fit by REML. t-tests use Satterthwaite's method ['lmerModLmerTest']
Formula: Ratio_movement_to_detection_duration_H0_Zone_1 ~ 1 + (1 | Genotype_Zone_1:Plant_Zone_1) +
(1 | Genotype_Zone_2:Plant_Zone_2)
   Data: data

REML criterion at convergence: 40.3

Scaled residuals:
    Min      1Q  Median      3Q     Max
-3.9423 -0.2410  0.1371  0.5034  1.2674

Random effects:
 Groups                       Name        Variance Std.Dev.
 Genotype_Zone_1:Plant_Zone_1 (Intercept) 0.0909   0.3015
 Genotype_Zone_2:Plant_Zone_2 (Intercept) 0.0000   0.0000
 Residual                                 0.1343   0.3665
Number of obs: 33, groups:  Genotype_Zone_1:Plant_Zone_1, 10; Genotype_Zone_2:Plant_Zone_2, 10

Fixed effects:
            Estimate Std. Error      df t value Pr(>|t|)
(Intercept)  -0.4174     0.1165  6.4696  -3.583   0.0102 *
---
Signif. codes:  0 '***' 0.001 '**' 0.01 '*' 0.05 '.' 0.1 ' ' 1
```

Model residuals

Statistic	Value
Sample skewness	-2.727
Sample excess kurtosis	11.1
Passed Shapiro Wilk normality test	No (p-value = 7.09E-06 < 0.05)

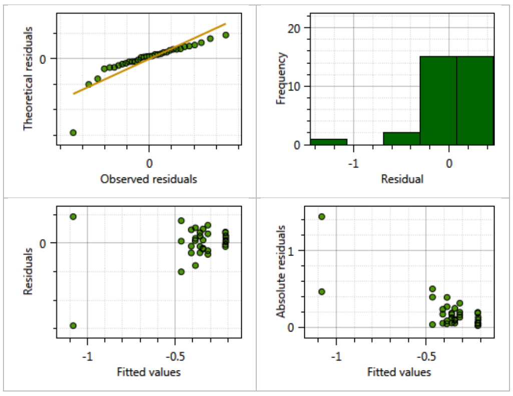

Analysis ratio movement to detection duration (H0 - Zone 2)

Analysis model	Linear mixed model fit by REML: Ratio_movement_to_detection_duration_H0_Zone_2 ~ 1 + (1|Genotype_Zone_1:Plant_Zone_1) + (1|Genotype_Zone_2:Plant_Zone_2)
Transformation	Natural logarithm

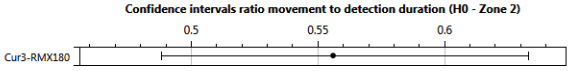

Confidence intervals ratio movement to detection duration (H0 - Zone 2)

Genotype Zone 1	Genotype Zone 2	Mean	Lower 95% CL	Upper 95% CL	Group
Cur3	RMX180	0.5559	0.4881	0.6332	a

Model summary

```
Linear mixed model fit by REML. t-tests use Satterthwaite's method ['lmerModLmerTest']
Formula: Ratio_movement_to_detection_duration_H0_Zone_2 ~ 1 + (1 | Genotype_Zone_1:Plant_Zone_1) +
(1 | Genotype_Zone_2:Plant_Zone_2)
   Data: data

REML criterion at convergence: 10.5

Scaled residuals:
    Min      1Q  Median      3Q     Max
-2.2893 -0.5360  0.1256  0.4088  1.9777

Random effects:
 Groups                       Name        Variance Std.Dev.
 Genotype_Zone_1:Plant_Zone_1 (Intercept) 0.00000  0.0000
 Genotype_Zone_2:Plant_Zone_2 (Intercept) 0.01547  0.1244
 Residual                                 0.06035  0.2457
Number of obs: 36, groups:  Genotype_Zone_1:Plant_Zone_1, 10; Genotype_Zone_2:Plant_Zone_2, 10

Fixed effects:
            Estimate Std. Error       df t value Pr(>|t|)
(Intercept) -0.58711    0.05706  8.55735  -10.29 4.15e-06 ***
---
Signif. codes:  0 '***' 0.001 '**' 0.01 '*' 0.05 '.' 0.1 ' ' 1
```

Model residuals

Statistic	Value
Sample skewness	-0.4822
Sample excess kurtosis	0.8256
Passed Shapiro Wilk normality test	Yes (p-value = 0.2839 > 0.05)

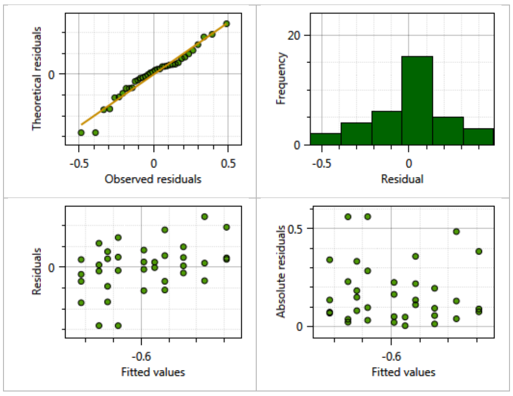

Analysis ratio movement to detection duration (H1 - Zone 1)

Analysis model	Linear mixed model fit by REML: Ratio_movement_to_detection_duration_H1_Zone_1 ~ 1 + (1|Genotype_Zone_1:Plant_Zone_1) + (1|Genotype_Zone_2:Plant_Zone_2)
Transformation	Natural logarithm

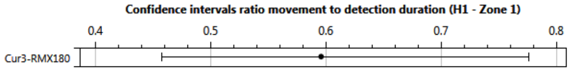

Confidence intervals ratio movement to detection duration (H1 - Zone 1)

Genotype Zone 1	Genotype Zone 2	Mean	Lower 95% CL	Upper 95% CL	Group
Cur3	RMX180	0.5958	0.4572	0.7764	a

Model summary

```
Linear mixed model fit by REML. t-tests use Satterthwaite's method ['lmerModLmerTest']
Formula: Ratio_movement_to_detection_duration_H1_Zone_1 ~ 1 + (1 | Genotype_Zone_1:Plant_Zone_1) +
(1 | Genotype_Zone_2:Plant_Zone_2)
   Data: data

REML criterion at convergence: 64.6

Scaled residuals:
    Min      1Q  Median      3Q     Max
```

```
-4.3455 -0.1795  0.2740  0.5615  0.7718

Random effects:
 Groups                        Name        Variance Std.Dev.
 Genotype_Zone_1:Plant_Zone_1 (Intercept) 0.00000  0.0000
 Genotype_Zone_2:Plant_Zone_2 (Intercept) 0.04514  0.2125
 Residual                                 0.29938  0.5472
Number of obs: 36, groups:  Genotype_Zone_1:Plant_Zone_1, 10; Genotype_Zone_2:Plant_Zone_2, 10

Fixed effects:
            Estimate Std. Error      df t value Pr(>|t|)
(Intercept)  -0.5178     0.1135  7.5171  -4.562  0.00217 **
---
Signif. codes:  0 '***' 0.001 '**' 0.01 '*' 0.05 '.' 0.1 ' ' 1
```

Model residuals

Statistic	Value
Sample skewness	-3.096
Sample excess kurtosis	12.08
Passed Shapiro Wilk normality test	No (p-value = 1.074E-07 < 0.05)

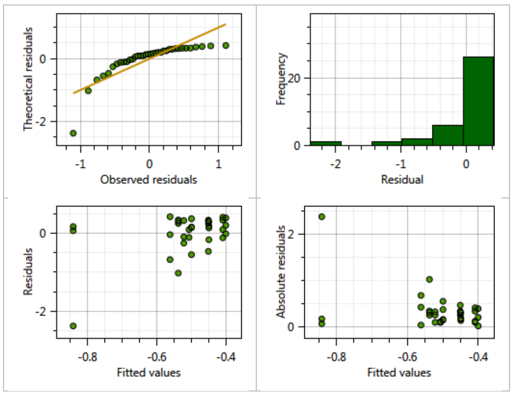

Analysis ratio movement to detection duration (H1 - Zone 2)

**Analysis model**	Linear mixed model fit by REML: Ratio_movement_to_detection_duration_H1_Zone_2 ~ 1 + (1|Genotype_Zone_1:Plant_Zone_1) + (1|Genotype_Zone_2:Plant_Zone_2)
**Transformation**	Natural logarithm

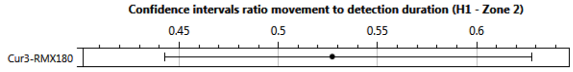

Confidence intervals ratio movement to detection duration (H1 - Zone 2)

Genotype Zone 1	Genotype Zone 2	Mean	Lower 95% CL	Upper 95% CL	Group
Cur3	RMX180	0.5272	0.4426	0.628	a

Model summary

```
Linear mixed model fit by REML. t-tests use Satterthwaite's method ['lmerModLmerTest']
Formula: Ratio_movement_to_detection_duration_H1_Zone_2 ~ 1 + (1 | Genotype_Zone_1:Plant_Zone_1) +
(1 | Genotype_Zone_2:Plant_Zone_2)
   Data: data

REML criterion at convergence: 40.7

Scaled residuals:
    Min      1Q  Median      3Q     Max
-4.8511 -0.1745  0.1093  0.4000  1.5033

Random effects:
 Groups                        Name        Variance  Std.Dev.
 Genotype_Zone_1:Plant_Zone_1 (Intercept) 1.400e-17 3.742e-09
 Genotype_Zone_2:Plant_Zone_2 (Intercept) 8.840e-03 9.402e-02
 Residual                                 1.566e-01 3.957e-01
Number of obs: 37, groups:  Genotype_Zone_1:Plant_Zone_1, 10; Genotype_Zone_2:Plant_Zone_2, 10

Fixed effects:
            Estimate Std. Error       df t value Pr(>|t|)
(Intercept) -0.64023    0.07161  6.03967   -8.94 0.000105 ***
---
Signif. codes:  0 '***' 0.001 '**' 0.01 '*' 0.05 '.' 0.1 ' ' 1
```

Model residuals

Statistic	Value
Sample skewness	-3.451
Sample excess kurtosis	17
Passed Shapiro Wilk normality test	No (p-value = 1.079E-07 < 0.05)

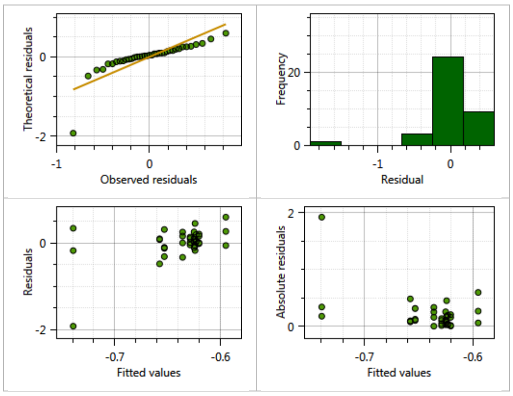

Analysis ratio movement to detection duration (H2 - Zone 1)

**Analysis model**	Linear mixed model fit by REML: Ratio_movement_to_detection_duration_H2_Zone_1 ~ 1 + (1|Genotype_Zone_1:Plant_Zone_1) + (1|Genotype_Zone_2:Plant_Zone_2)
**Transformation**	Natural logarithm

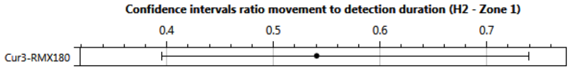

Confidence intervals ratio movement to detection duration (H2 - Zone 1)

Genotype Zone 1	Genotype Zone 2	Mean	Lower 95% CL	Upper 95% CL	Group
Cur3	RMX180	0.5405	0.395	0.7397	a

Model summary

```
Linear mixed model fit by REML. t-tests use Satterthwaite's method ['lmerModLmerTest']
Formula: Ratio_movement_to_detection_duration_H2_Zone_1 ~ 1 + (1 | Genotype_Zone_1:Plant_Zone_1) +
(1 | Genotype_Zone_2:Plant_Zone_2)
   Data: data

REML criterion at convergence: 71

Scaled residuals:
    Min      1Q  Median      3Q     Max
-3.0585 -0.4411  0.3481  0.5708  1.4531

Random effects:
 Groups                       Name        Variance Std.Dev.
 Genotype_Zone_1:Plant_Zone_1 (Intercept) 0.0000   0.0000
 Genotype_Zone_2:Plant_Zone_2 (Intercept) 0.1004   0.3168
 Residual                                 0.3328   0.5769
Number of obs: 36, groups:  Genotype_Zone_1:Plant_Zone_1, 10; Genotype_Zone_2:Plant_Zone_2, 10

Fixed effects:
            Estimate Std. Error      df t value Pr(>|t|)
(Intercept)  -0.6152     0.1392  9.2324   -4.42  0.00157 **
---
Signif. codes:  0 '***' 0.001 '**' 0.01 '*' 0.05 '.' 0.1 ' ' 1
```

Model residuals

Statistic	Value
Sample skewness	-1.342
Sample excess kurtosis	2.099
Passed Shapiro Wilk normality test	No (p-value = 0.001793 < 0.05)

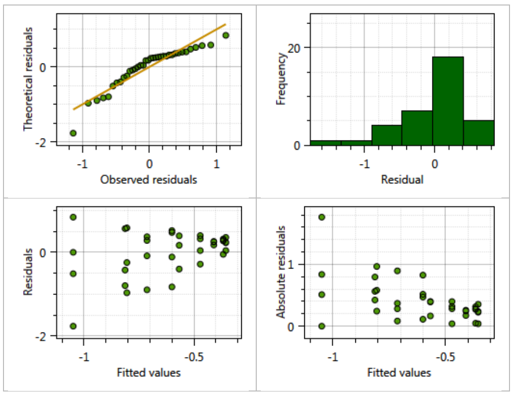

Analysis ratio movement to detection duration (H2 - Zone 2)

**Analysis model**	Linear mixed model fit by REML: Ratio_movement_to_detection_duration_H2_Zone_2 ~ 1 + (1|Genotype_Zone_1:Plant_Zone_1) + (1|Genotype_Zone_2:Plant_Zone_2)
**Transformation**	Natural logarithm

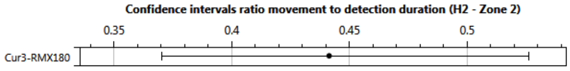

Confidence intervals ratio movement to detection duration (H2 - Zone 2)

Genotype Zone 1	Genotype Zone 2	Mean	Lower 95% CL	Upper 95% CL	Group
Cur3	RMX180	0.4413	0.3701	0.5262	a

Model summary

```
Linear mixed model fit by REML. t-tests use Satterthwaite's method ['lmerModLmerTest']
Formula: Ratio_movement_to_detection_duration_H2_Zone_2 ~ 1 + (1 | Genotype_Zone_1:Plant_Zone_1) +
(1 | Genotype_Zone_2:Plant_Zone_2)
   Data: data

REML criterion at convergence: 59.8

Scaled residuals:
    Min      1Q  Median      3Q     Max
-3.4868 -0.4389  0.1983  0.5176  1.3285

Random effects:
 Groups                       Name        Variance Std.Dev.
 Genotype_Zone_1:Plant_Zone_1 (Intercept) 0.0000   0.0000
 Genotype_Zone_2:Plant_Zone_2 (Intercept) 0.0000   0.0000
 Residual                                 0.2788   0.5281
Number of obs: 37, groups:  Genotype_Zone_1:Plant_Zone_1, 10; Genotype_Zone_2:Plant_Zone_2, 10

Fixed effects:
            Estimate Std. Error       df t value Pr(>|t|)
(Intercept) -0.81805    0.08681 36.00000  -9.423 2.96e-11 ***
---
Signif. codes:  0 '***' 0.001 '**' 0.01 '*' 0.05 '.' 0.1 ' ' 1
```

Model residuals

Statistic	Value
Sample skewness	-2.03
Sample excess kurtosis	5.557
Passed Shapiro Wilk normality test	No (p-value = 1.62E-05 < 0.05)

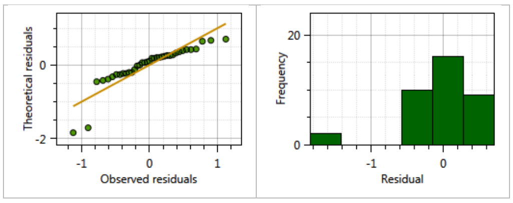

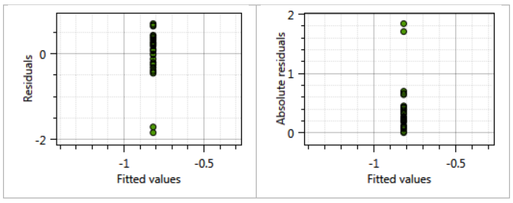

Analysis ratio movement to detection duration (H3 - Zone 1)

**Analysis model**	Linear mixed model fit by REML: Ratio_movement_to_detection_duration_H3_Zone_1 ~ 1 + (1|Genotype_Zone_1:Plant_Zone_1) + (1|Genotype_Zone_2:Plant_Zone_2)
**Transformation**	Natural logarithm

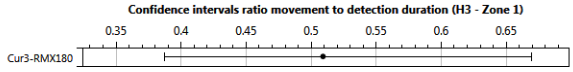

Confidence intervals ratio movement to detection duration (H3 - Zone 1)

Genotype Zone 1	Genotype Zone 2	Mean	Lower 95% CL	Upper 95% CL	Group
Cur3	RMX180	0.5091	0.387	0.6697	a

Model summary

```
Linear mixed model fit by REML. t-tests use Satterthwaite's method ['lmerModLmerTest']
Formula: Ratio_movement_to_detection_duration_H3_Zone_1 ~ 1 + (1 | Genotype_Zone_1:Plant_Zone_1) +
(1 | Genotype_Zone_2:Plant_Zone_2)
   Data: data

REML criterion at convergence: 54.8

Scaled residuals:
    Min      1Q  Median      3Q     Max
-2.3647 -0.5421  0.2401  0.5972  1.6333

Random effects:
 Groups                       Name        Variance Std.Dev.
 Genotype_Zone_1:Plant_Zone_1 (Intercept) 0.01696  0.1302
 Genotype_Zone_2:Plant_Zone_2 (Intercept) 0.04675  0.2162
 Residual                                 0.24138  0.4913
Number of obs: 33, groups:  Genotype_Zone_1:Plant_Zone_1, 10; Genotype_Zone_2:Plant_Zone_2, 10

Fixed effects:
            Estimate Std. Error      df t value Pr(>|t|)
(Intercept)  -0.6752     0.1179  7.6118  -5.728 0.000528 ***
---
Signif. codes:  0 '***' 0.001 '**' 0.01 '*' 0.05 '.' 0.1 ' ' 1
```

Model residuals

Statistic	Value
Sample skewness	-0.8167
Sample excess kurtosis	0.2945
Passed Shapiro Wilk normality test	Yes (p-value = 0.06869 > 0.05)

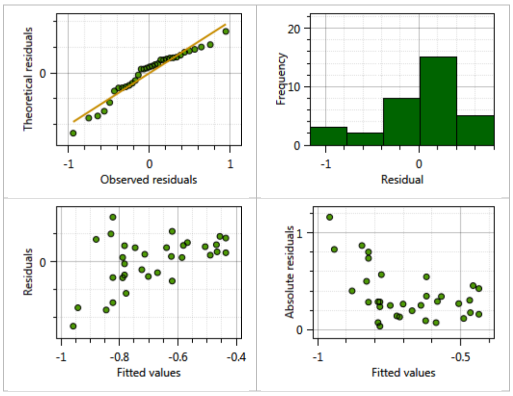

Analysis ratio movement to detection duration (H3 - Zone 2)

**Analysis model**	Linear mixed model fit by REML: Ratio_movement_to_detection_duration_H3_Zone_2 ~ 1 + (1|Genotype_Zone_1:Plant_Zone_1) + (1|Genotype_Zone_2:Plant_Zone_2)
**Transformation**	Natural logarithm

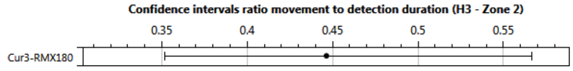

Confidence intervals ratio movement to detection duration (H3 - Zone 2)

Genotype Zone 1	Genotype Zone 2	Mean	Lower 95% CL	Upper 95% CL	Group
Cur3	RMX180	0.4464	0.3515	0.5668	a

Model summary

```
Linear mixed model fit by REML. t-tests use Satterthwaite's method ['lmerModLmerTest']
Formula: Ratio_movement_to_detection_duration_H3_Zone_2 ~ 1 + (1 | Genotype_Zone_1:Plant_Zone_1) +
(1 | Genotype_Zone_2:Plant_Zone_2)
   Data: data

REML criterion at convergence: 72

Scaled residuals:
    Min      1Q  Median      3Q     Max
-3.5281 -0.1882  0.2662  0.5888  1.1442

Random effects:
 Groups                       Name        Variance Std.Dev.
 Genotype_Zone_1:Plant_Zone_1 (Intercept) 0.00000  0.0000
 Genotype_Zone_2:Plant_Zone_2 (Intercept) 0.02689  0.1640
 Residual                                 0.33167  0.5759
Number of obs: 39, groups:  Genotype_Zone_1:Plant_Zone_1, 10; Genotype_Zone_2:Plant_Zone_2, 10

Fixed effects:
            Estimate Std. Error      df t value Pr(>|t|)
(Intercept)  -0.8066     0.1059  9.1706   -7.62 2.94e-05 ***
---
Signif. codes:  0 '***' 0.001 '**' 0.01 '*' 0.05 '.' 0.1 ' ' 1
```

Model residuals

Statistic	Value
Sample skewness	-1.944
Sample excess kurtosis	4.179
Passed Shapiro Wilk normality test	No (p-value = 8.774E-06 < 0.05)

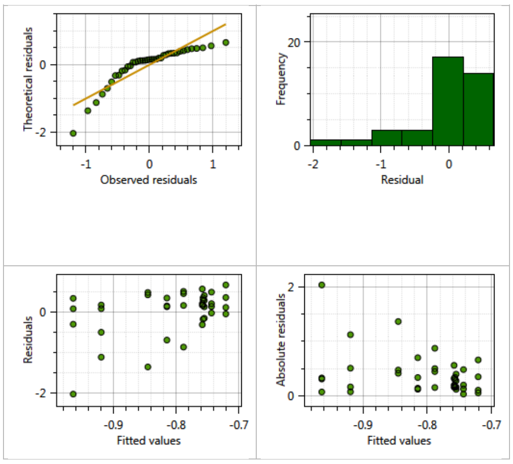

Analysis ratio movement to detection duration (H4 - Zone 1)

**Analysis model**	Linear mixed model fit by REML: Ratio_movement_to_detection_duration_H4_Zone_1 ~ 1 + (1|Genotype_Zone_1:Plant_Zone_1) + (1|Genotype_Zone_2:Plant_Zone_2)
**Transformation**	Natural logarithm

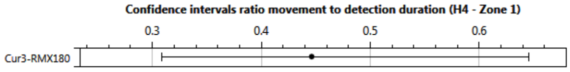

Confidence intervals ratio movement to detection duration (H4 - Zone 1)

Genotype Zone 1	Genotype Zone 2	Mean	Lower 95% CL	Upper 95% CL	Group
Cur3	RMX180	0.4462	0.3083	0.6457	a

Model summary

```
Linear mixed model fit by REML. t-tests use Satterthwaite's method ['lmerModLmerTest']
Formula: Ratio_movement_to_detection_duration_H4_Zone_1 ~ 1 + (1 | Genotype_Zone_1:Plant_Zone_1) +
(1 | Genotype_Zone_2:Plant_Zone_2)
   Data: data

REML criterion at convergence: 69.9

Scaled residuals:
    Min      1Q  Median      3Q     Max
-2.3925 -0.2011  0.3861  0.6667  1.0638

Random effects:
 Groups                       Name        Variance Std.Dev.
 Genotype_Zone_1:Plant_Zone_1 (Intercept) 0.0000   0.0000
 Genotype_Zone_2:Plant_Zone_2 (Intercept) 0.0790   0.2811
 Residual                                 0.5169   0.7189
Number of obs: 30, groups:  Genotype_Zone_1:Plant_Zone_1, 10; Genotype_Zone_2:Plant_Zone_2, 10

Fixed effects:
            Estimate Std. Error      df t value Pr(>|t|)
(Intercept)  -0.8070     0.1597  7.8349  -5.054  0.00105 **
---
Signif. codes:  0 '***' 0.001 '**' 0.01 '*' 0.05 '.' 0.1 ' ' 1
```

Model residuals

Statistic	Value
Sample skewness	-1.322
Sample excess kurtosis	0.7704
Passed Shapiro Wilk normality test	No (p-value = 0.0001739 < 0.05)

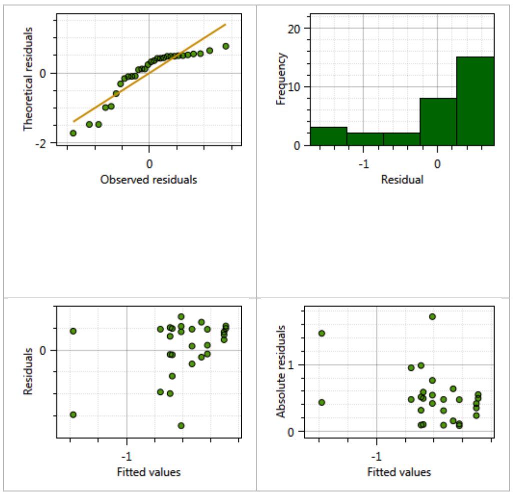

Analysis ratio movement to detection duration (H4 - Zone 2)

**Analysis model**	Linear mixed model fit by REML: Ratio_movement_to_detection_duration_H4_Zone_2 ~ 1 + (1|Genotype_Zone_1:Plant_Zone_1) + (1|Genotype_Zone_2:Plant_Zone_2)
**Transformation**	Natural logarithm

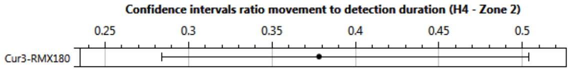

Confidence intervals ratio movement to detection duration (H4 - Zone 2)

Genotype Zone 1	Genotype Zone 2	Mean	Lower 95% CL	Upper 95% CL	Group
Cur3	RMX180	0.3782	0.2838	0.504	a

Model summary

```
Linear mixed model fit by REML. t-tests use Satterthwaite's method ['lmerModLmerTest']
Formula: Ratio_movement_to_detection_duration_H4_Zone_2 ~ 1 + (1 | Genotype_Zone_1:Plant_Zone_1) +
(1 | Genotype_Zone_2:Plant_Zone_2)
   Data: data

REML criterion at convergence: 74.3

Scaled residuals:
    Min      1Q  Median      3Q     Max
-2.9975 -0.0326  0.2569  0.5125  1.2086

Random effects:
 Groups                       Name        Variance Std.Dev.
 Genotype_Zone_1:Plant_Zone_1 (Intercept) 0.00000  0.0000
 Genotype_Zone_2:Plant_Zone_2 (Intercept) 0.02756  0.1660
 Residual                                 0.39365  0.6274
Number of obs: 37, groups:  Genotype_Zone_1:Plant_Zone_1, 10; Genotype_Zone_2:Plant_Zone_2, 10

Fixed effects:
            Estimate Std. Error      df t value Pr(>|t|)
(Intercept)  -0.9724     0.1160  5.7327  -8.379    2e-04 ***
---
Signif. codes:  0 '***' 0.001 '**' 0.01 '*' 0.05 '.' 0.1 ' ' 1
```

Model residuals

Statistic	Value
Sample skewness	-1.792
Sample excess kurtosis	3.104
Passed Shapiro Wilk normality test	No (p-value = 1.287E-05 < 0.05)

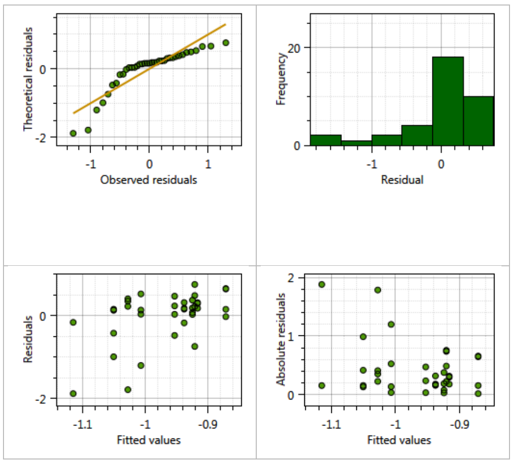

Analysis ratio movement to detection duration (H5 - Zone 1)

**Analysis model**	Linear mixed model fit by REML: Ratio_movement_to_detection_duration_H5_Zone_1 ~ 1 + (1|Genotype_Zone_1:Plant_Zone_1) + (1|Genotype_Zone_2:Plant_Zone_2)
**Transformation**	Natural logarithm

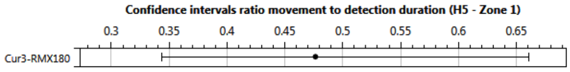

Confidence intervals ratio movement to detection duration (H5 - Zone 1)

Genotype Zone 1	Genotype Zone 2	Mean	Lower 95% CL	Upper 95% CL	Group
Cur3	RMX180	0.4763	0.3433	0.661	a

Model summary

```
Linear mixed model fit by REML. t-tests use Satterthwaite's method ['lmerModLmerTest']
Formula: Ratio_movement_to_detection_duration_H5_Zone_1 ~ 1 + (1 | Genotype_Zone_1:Plant_Zone_1) +
(1 | Genotype_Zone_2:Plant_Zone_2)
   Data: data

REML criterion at convergence: 62.6

Scaled residuals:
    Min      1Q  Median      3Q     Max
-2.4473 -0.4980  0.1956  0.6349  1.1829

Random effects:
 Groups                       Name        Variance Std.Dev.
 Genotype_Zone_1:Plant_Zone_1 (Intercept) 0.080838 0.28432
 Genotype_Zone_2:Plant_Zone_2 (Intercept) 0.003602 0.06001
 Residual                                 0.356889 0.59740
Number of obs: 31, groups:  Genotype_Zone_1:Plant_Zone_1, 10; Genotype_Zone_2:Plant_Zone_2, 10

Fixed effects:
            Estimate Std. Error      df t value Pr(>|t|)
(Intercept)  -0.7416     0.1426  8.1829  -5.201 0.000765 ***
---
Signif. codes:  0 '***' 0.001 '**' 0.01 '*' 0.05 '.' 0.1 ' ' 1
```

Model residuals

Statistic	Value
Sample skewness	-0.9809
Sample excess kurtosis	0.7264
Passed Shapiro Wilk normality test	No (p-value = 0.02248 < 0.05)

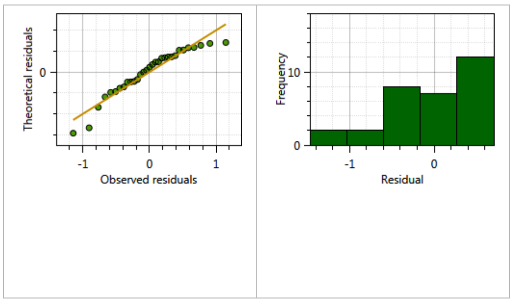

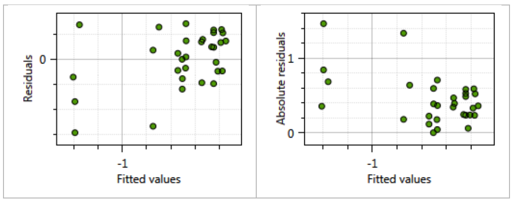

Analysis ratio movement to detection duration (H5 - Zone 2)

**Analysis model**	Linear mixed model fit by REML: Ratio_movement_to_detection_duration_H5_Zone_2 ~ 1 + (1|Genotype_Zone_1:Plant_Zone_1) + (1|Genotype_Zone_2:Plant_Zone_2)
**Transformation**	Natural logarithm

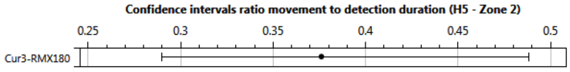

Confidence intervals ratio movement to detection duration (H5 - Zone 2)

Genotype Zone 1	Genotype Zone 2	Mean	Lower 95% CL	Upper 95% CL	Group
Cur3	RMX180	0.3761	0.2896	0.4884	a

Model summary

```
Linear mixed model fit by REML. t-tests use Satterthwaite's method ['lmerModLmerTest']
Formula: Ratio_movement_to_detection_duration_H5_Zone_2 ~ 1 + (1 | Genotype_Zone_1:Plant_Zone_1) +
(1 | Genotype_Zone_2:Plant_Zone_2)
   Data: data

REML criterion at convergence: 76

Scaled residuals:
    Min      1Q  Median      3Q     Max
-3.3769 -0.4693  0.2427  0.6612  1.3713

Random effects:
 Groups                       Name        Variance Std.Dev.
 Genotype_Zone_1:Plant_Zone_1 (Intercept) 0.04202  0.2050
 Genotype_Zone_2:Plant_Zone_2 (Intercept) 0.00000  0.0000
 Residual                                 0.35971  0.5998
Number of obs: 39, groups:  Genotype_Zone_1:Plant_Zone_1, 10; Genotype_Zone_2:Plant_Zone_2, 10

Fixed effects:
            Estimate Std. Error      df t value Pr(>|t|)
(Intercept)  -0.9779     0.1159  9.2385  -8.434 1.23e-05 ***
---
Signif. codes:  0 '***' 0.001 '**' 0.01 '*' 0.05 '.' 0.1 ' ' 1
```

Model residuals

Statistic	Value
Sample skewness	-1.404
Sample excess kurtosis	2.846
Passed Shapiro Wilk normality test	No (p-value = 0.002086 < 0.05)

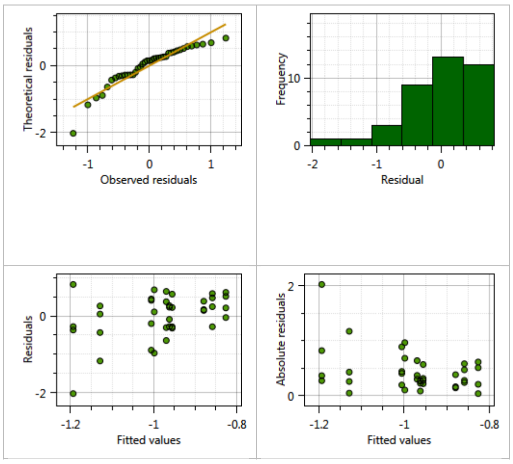

Analysis ratio movement to detection duration (H6 - Zone 1)

**Analysis model**	Linear mixed model fit by REML: Ratio_movement_to_detection_duration_H6_Zone_1 ~ 1 + (1|Genotype_Zone_1:Plant_Zone_1) + (1|Genotype_Zone_2:Plant_Zone_2)
**Transformation**	Natural logarithm

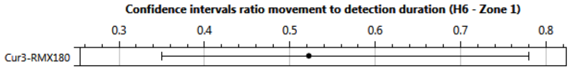

Confidence intervals ratio movement to detection duration (H6 - Zone 1)

Genotype Zone 1	Genotype Zone 2	Mean	Lower 95% CL	Upper 95% CL	Group
Cur3	RMX180	0.5222	0.3495	0.7802	a

Model summary

```
Linear mixed model fit by REML. t-tests use Satterthwaite's method ['lmerModLmerTest']
Formula: Ratio_movement_to_detection_duration_H6_Zone_1 ~ 1 + (1 | Genotype_Zone_1:Plant_Zone_1) +
(1 | Genotype_Zone_2:Plant_Zone_2)
   Data: data

REML criterion at convergence: 58

Scaled residuals:
    Min      1Q  Median      3Q     Max
-2.4588 -0.2706  0.2866  0.4878  1.4006

Random effects:
 Groups                       Name        Variance Std.Dev.
 Genotype_Zone_1:Plant_Zone_1 (Intercept) 0.0000   0.0000
 Genotype_Zone_2:Plant_Zone_2 (Intercept) 0.1608   0.4010
 Residual                                 0.3712   0.6093
Number of obs: 27, groups:  Genotype_Zone_1:Plant_Zone_1, 10; Genotype_Zone_2:Plant_Zone_2, 10

Fixed effects:
            Estimate Std. Error      df t value Pr(>|t|)
(Intercept)  -0.6497     0.1770  8.8496   -3.67  0.00531 **
---
Signif. codes:  0 '***' 0.001 '**' 0.01 '*' 0.05 '.' 0.1 ' ' 1
```

Model residuals

Statistic	Value
Sample skewness	-1.207
Sample excess kurtosis	1.294
Passed Shapiro Wilk normality test	No (p-value = 0.009886 < 0.05)

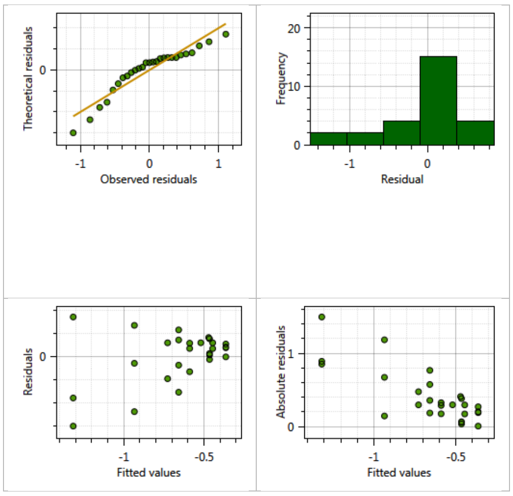

Analysis ratio movement to detection duration (H6 - Zone 2)

**Analysis model**	Linear mixed model fit by REML: Ratio_movement_to_detection_duration_H6_Zone_2 ~ 1 + (1|Genotype_Zone_1:Plant_Zone_1) + (1|Genotype_Zone_2:Plant_Zone_2)
**Transformation**	Natural logarithm

Confidence intervals ratio movement to detection duration (H6 - Zone 2)

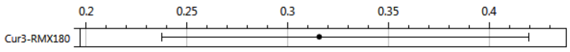

Confidence intervals ratio movement to detection duration (H6 - Zone 2)

Genotype Zone 1	Genotype Zone 2	Mean	Lower 95% CL	Upper 95% CL	Group
Cur3	RMX180	0.3156	0.2373	0.4198	a

Model summary

```
Linear mixed model fit by REML. t-tests use Satterthwaite's method ['lmerModLmerTest']
Formula: Ratio_movement_to_detection_duration_H6_Zone_2 ~ 1 + (1 | Genotype_Zone_1:Plant_Zone_1) +
(1 | Genotype_Zone_2:Plant_Zone_2)
   Data: data

REML criterion at convergence: 86.5

Scaled residuals:
    Min      1Q  Median      3Q     Max
-3.6731 -0.5087  0.2754  0.6331  1.1694

Random effects:
 Groups                       Name        Variance  Std.Dev.
 Genotype_Zone_1:Plant_Zone_1 (Intercept) 1.826e-02 1.351e-01
 Genotype_Zone_2:Plant_Zone_2 (Intercept) 1.558e-15 3.948e-08
 Residual                                 5.342e-01 7.309e-01
Number of obs: 38, groups:  Genotype_Zone_1:Plant_Zone_1, 10; Genotype_Zone_2:Plant_Zone_2, 10

Fixed effects:
            Estimate Std. Error      df t value Pr(>|t|)
(Intercept)  -1.1532     0.1261  8.9886  -9.145 7.56e-06 ***
---
Signif. codes:  0 '***' 0.001 '**' 0.01 '*' 0.05 '.' 0.1 ' ' 1
```

Model residuals

Statistic	Value
Sample skewness	-1.713
Sample excess kurtosis	3.954
Passed Shapiro Wilk normality test	No (p-value = 0.0001193 < 0.05)

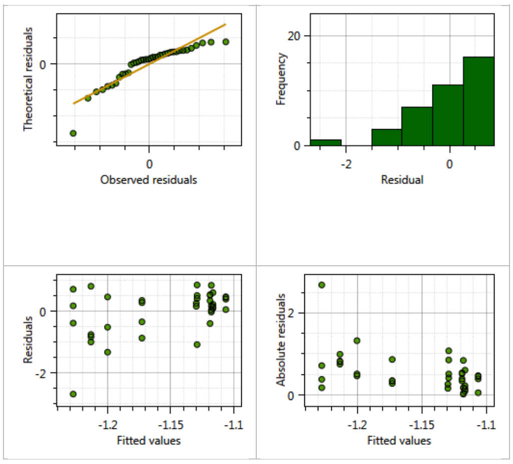

Analysis ratio movement to detection duration (H7 - Zone 1)

**Analysis model**	Linear mixed model fit by REML: Ratio_movement_to_detection_duration_H7_Zone_1 ~ 1 + (1|Genotype_Zone_1:Plant_Zone_1) + (1|Genotype_Zone_2:Plant_Zone_2)
**Transformation**	Natural logarithm

Confidence intervals ratio movement to detection duration (H7 - Zone 1)

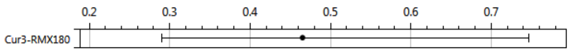

Confidence intervals ratio movement to detection duration (H7 - Zone 1)

Genotype Zone 1	Genotype Zone 2	Mean	Lower 95% CL	Upper 95% CL	Group
Cur3	RMX180	0.4652	0.2897	0.747	a

Model summary

```
Linear mixed model fit by REML. t-tests use Satterthwaite's method ['lmerModLmerTest']
Formula: Ratio_movement_to_detection_duration_H7_Zone_1 ~ 1 + (1 | Genotype_Zone_1:Plant_Zone_1) +
(1 | Genotype_Zone_2:Plant_Zone_2)
   Data: data

REML criterion at convergence: 64.2

Scaled residuals:
    Min      1Q  Median      3Q     Max
-2.8498 -0.2151  0.1491  0.5453  1.8688

Random effects:
 Groups                       Name        Variance Std.Dev.
 Genotype_Zone_2:Plant_Zone_2 (Intercept) 0.03593  0.1896
 Genotype_Zone_1:Plant_Zone_1 (Intercept) 0.19424  0.4407
 Residual                                 0.40307  0.6349
Number of obs: 28, groups:  Genotype_Zone_2:Plant_Zone_2, 10; Genotype_Zone_1:Plant_Zone_1, 9

Fixed effects:
            Estimate Std. Error      df t value Pr(>|t|)
(Intercept)  -0.7653     0.2015  7.2132  -3.798  0.00636 **
---
Signif. codes:  0 '***' 0.001 '**' 0.01 '*' 0.05 '.' 0.1 ' ' 1
```

Model residuals

Statistic	Value
Sample skewness	-1.244
Sample excess kurtosis	3.41
Passed Shapiro Wilk normality test	No (p-value = 0.005849 < 0.05)

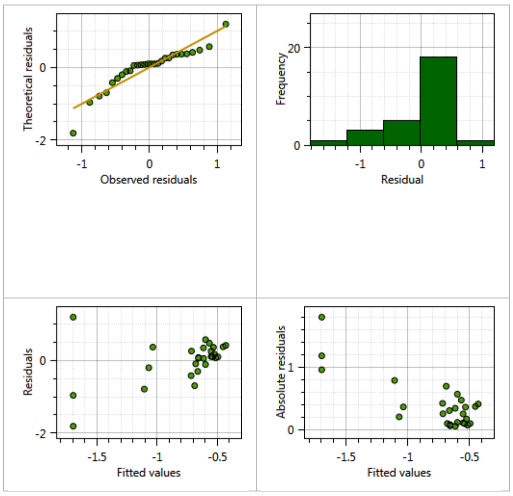

Analysis ratio movement to detection duration (H7 - Zone 2)

**Analysis model**	Linear mixed model fit by REML: Ratio_movement_to_detection_duration_H7_Zone_2 ~ 1 + (1|Genotype_Zone_1:Plant_Zone_1) + (1|Genotype_Zone_2:Plant_Zone_2)
**Transformation**	Natural logarithm

Confidence intervals ratio movement to detection duration (H7 - Zone 2)

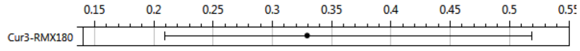

Confidence intervals ratio movement to detection duration (H7 - Zone 2)

Genotype Zone 1	Genotype Zone 2	Mean	Lower 95% CL	Upper 95% CL	Group
Cur3	RMX180	0.3293	0.2089	0.5191	a

Model summary

```
Linear mixed model fit by REML. t-tests use Satterthwaite's method ['lmerModLmerTest']
Formula: Ratio_movement_to_detection_duration_H7_Zone_2 ~ 1 + (1 | Genotype_Zone_1:Plant_Zone_1) +
(1 | Genotype_Zone_2:Plant_Zone_2)
   Data: data

REML criterion at convergence: 85.1

Scaled residuals:
    Min      1Q  Median      3Q     Max
-2.8301 -0.4385  0.1720  0.4554  1.6247

Random effects:
 Groups                       Name        Variance Std.Dev.
 Genotype_Zone_1:Plant_Zone_1 (Intercept) 0.22447  0.4738
 Genotype_Zone_2:Plant_Zone_2 (Intercept) 0.08578  0.2929
 Residual                                 0.31687  0.5629
Number of obs: 39, groups:  Genotype_Zone_1:Plant_Zone_1, 10; Genotype_Zone_2:Plant_Zone_2, 10

Fixed effects:
            Estimate Std. Error      df t value Pr(>|t|)
(Intercept)  -1.1107     0.1981  8.1834  -5.608 0.000466 ***
---
Signif. codes:  0 '***' 0.001 '**' 0.01 '*' 0.05 '.' 0.1 ' ' 1
```

Model residuals

Statistic	Value
Sample skewness	-0.9166
Sample excess kurtosis	2.269
Passed Shapiro Wilk normality test	No (p-value = 0.04354 < 0.05)

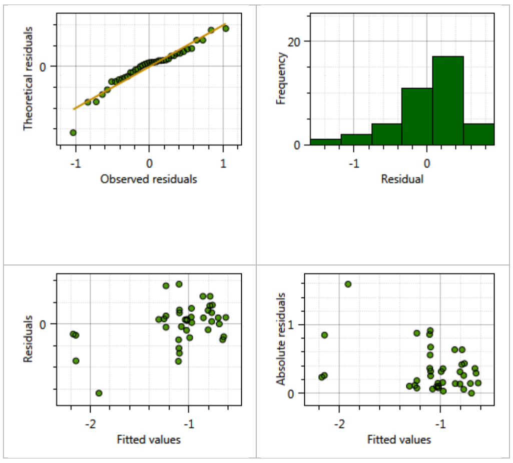

Analysis ratio movement to detection duration H0 (diff. Zone 1 - Zone 2)

**Analysis model**	Generalized linear mixed model with dispersion factor, formula=cbind(Ratio_movement_to_detection_duration_H0_Zone_1,Ratio_movement_to_detection_duration_H0_Zone_2) ~ 1 + (1|Genotype_Zone_1:Plant_Zone_1) + (1|Genotype_Zone_2:Plant_Zone_2)
**Transformation**	Logit

Confidence intervals ratio movement to detection duration H0 (diff. Zone 1 - Zone 2)

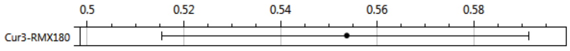

Confidence intervals ratio movement to detection duration H0 (diff. Zone 1 - Zone 2)

Genotype Zone 1	Genotype Zone 2	Mean	Lower 95% CL	Upper 95% CL	Group
Cur3	RMX180	0.5537	0.5153	0.5915	a

Model summary

```
Linear mixed model fit by REML. t-tests use Satterthwaite's method ['lmerModLmerTest']
Formula: ziFormula
   Data: data
Weights: wi

REML criterion at convergence: 45.9

Scaled residuals:
     Min       1Q   Median       3Q      Max
-3.13650 -0.35537  0.08674  0.67271  1.79103

Random effects:
 Groups                      Name        Variance Std.Dev.
 Genotype_Zone_1:Plant_Zone_1 (Intercept) 0.0000   0.0000
 Genotype_Zone_2:Plant_Zone_2 (Intercept) 0.0000   0.0000
 Residual                                 0.0618   0.2486
Number of obs: 34, groups:  Genotype_Zone_1:Plant_Zone_1, 10; Genotype_Zone_2:Plant_Zone_2, 10

Fixed effects:
            Estimate Std. Error       df t value Pr(>|t|)
(Intercept)  0.21569    0.07585 33.00000   2.844   0.0076 **
---
Signif. codes:  0 '***' 0.001 '**' 0.01 '*' 0.05 '.' 0.1 ' ' 1

Dispersion: 0.2486
```

Model residuals

Statistic	Value
Sample skewness	-1.137
Sample excess kurtosis	1.93
Passed Shapiro Wilk normality test	No (p-value = 0.01647 < 0.05)

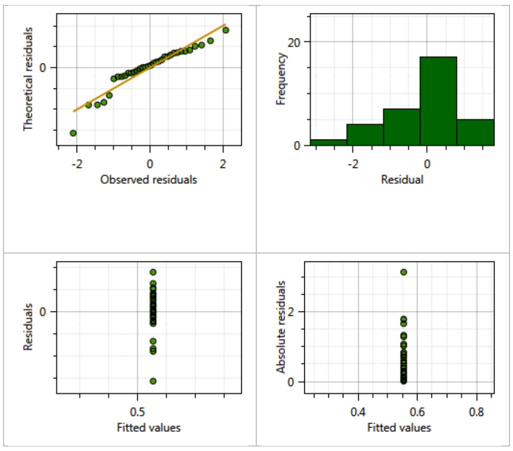

Analysis ratio movement to detection duration H1 (diff. Zone 1 - Zone 2)

**Analysis model**	Generalized linear mixed model with dispersion factor, formula=cbind(Ratio_movement_to_detection_duration_H1_Zone_1,Ratio_movement_to_detection_duration_H1_Zone_2) ~ 1 + (1|Genotype_Zone_1:Plant_Zone_1) + (1|Genotype_Zone_2:Plant_Zone_2)
**Transformation**	Logit

Confidence intervals ratio movement to detection duration H1 (diff. Zone 1 - Zone 2)

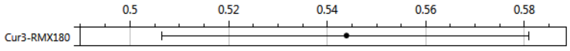

Confidence intervals ratio movement to detection duration H1 (diff. Zone 1 - Zone 2)

Genotype Zone 1	Genotype Zone 2	Mean	Lower 95% CL	Upper 95% CL	Group
Cur3	RMX180	0.5439	0.5063	0.5809	a

Model summary

```
Linear mixed model fit by REML. t-tests use Satterthwaite's method ['lmerModLmerTest']
Formula: ziFormula
   Data: data
```

```
Weights: wi

REML criterion at convergence: 48.2

Scaled residuals: 
    Min      1Q  Median      3Q     Max 
-2.8795 -0.4899  0.1180  0.6690  1.7095 

Random effects:
 Groups                        Name        Variance Std.Dev.
 Genotype_Zone_1:Plant_Zone_1 (Intercept) 0.00000  0.0000  
 Genotype_Zone_2:Plant_Zone_2 (Intercept) 0.00000  0.0000  
 Residual                                 0.06037  0.2457  
Number of obs: 36, groups:  Genotype_Zone_1:Plant_Zone_1, 10; Genotype_Zone_2:Plant_Zone_2, 10

Fixed effects:
            Estimate Std. Error      df t value Pr(>|t|)  
(Intercept)   0.1760     0.0742 35.0000   2.372   0.0233 *
---
Signif. codes:  0 '***' 0.001 '**' 0.01 '*' 0.05 '.' 0.1 ' ' 1

Dispersion: 0.2457
```

Model residuals

Statistic	Value
Sample skewness	-0.9303
Sample excess kurtosis	0.9799
Passed Shapiro Wilk normality test	Yes (p-value = 0.05516 > 0.05)

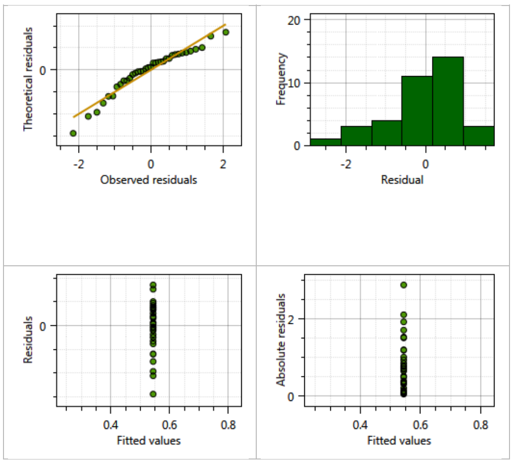

Analysis ratio movement to detection duration H2 (diff. Zone 1 - Zone 2)

**Analysis model**	Generalized linear mixed model with dispersion factor, formula=cbind(Ratio_movement_to_detection_duration_H2_Zone_1,Ratio_movement_to_detection_duration_H2_Zone_2) ~ 1 + (1|Genotype_Zone_1:Plant_Zone_1) + (1|Genotype_Zone_2:Plant_Zone_2)
**Transformation**	Logit

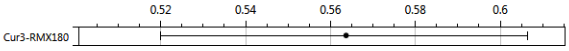

Confidence intervals ratio movement to detection duration H2 (diff. Zone 1 - Zone 2)

Genotype Zone 1	Genotype Zone 2	Mean	Lower 95% CL	Upper 95% CL	Group
Cur3	RMX180	0.5637	0.5199	0.6065	a

Model summary

```
Linear mixed model fit by REML. t-tests use Satterthwaite's method ['lmerModLmerTest']
Formula: ziFormula
   Data: data
Weights: wi

REML criterion at convergence: 65.1

Scaled residuals: 
    Min      1Q  Median      3Q     Max 
-2.2391 -0.5352  0.2299  0.6642  2.1968 

Random effects:
 Groups                        Name        Variance  Std.Dev. 
 Genotype_Zone_1:Plant_Zone_1 (Intercept) 0.000e+00 0.000e+00
 Genotype_Zone_2:Plant_Zone_2 (Intercept) 7.077e-18 2.660e-09
 Residual                                 7.539e-02 2.746e-01
Number of obs: 37, groups:  Genotype_Zone_1:Plant_Zone_1, 10; Genotype_Zone_2:Plant_Zone_2, 10

Fixed effects:
            Estimate Std. Error       df t value Pr(>|t|)   
(Intercept)  0.25602    0.08705 36.00000   2.941  0.00569 **
---
Signif. codes:  0 '***' 0.001 '**' 0.01 '*' 0.05 '.' 0.1 ' ' 1

Dispersion: 0.2746
```

Model residuals

Statistic	Value
Sample skewness	-0.4267
Sample excess kurtosis	0.1046
Passed Shapiro Wilk normality test	Yes (p-value = 0.3861 > 0.05)

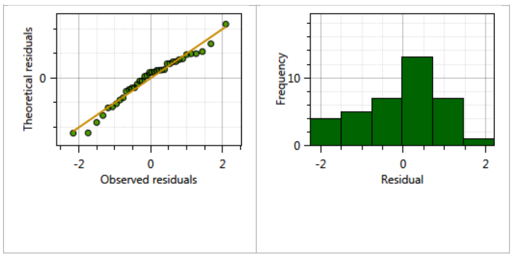

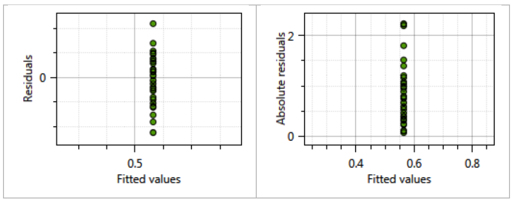

Analysis ratio movement to detection duration H3 (diff. Zone 1 - Zone 2)

**Analysis model**	Generalized linear mixed model with dispersion factor, formula=cbind(Ratio_movement_to_detection_duration_H3_Zone_1,Ratio_movement_to_detection_duration_H3_Zone_2) ~ 1 + (1|Genotype_Zone_1:Plant_Zone_1) + (1|Genotype_Zone_2:Plant_Zone_2)
**Transformation**	Logit

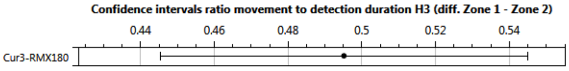

Confidence intervals ratio movement to detection duration H3 (diff. Zone 1 - Zone 2)

Genotype Zone 1	Genotype Zone 2	Mean	Lower 95% CL	Upper 95% CL	Group
Cur3	RMX180	0.4951	0.4453	0.5451	a

Model summary

```
Linear mixed model fit by REML. t-tests use Satterthwaite's method ['lmerModLmerTest']
Formula: ziFormula
   Data: data
Weights: wi

REML criterion at convergence: 66

Scaled residuals: 
     Min       1Q   Median       3Q      Max 
-2.21290 -0.63333  0.06631  0.56370  2.13322 

Random effects:
 Groups                        Name        Variance  Std.Dev.
 Genotype_Zone_1:Plant_Zone_1 (Intercept) 0.000e+00 0.000e+00
 Genotype_Zone_2:Plant_Zone_2 (Intercept) 1.043e-16 1.021e-08
 Residual                                 9.212e-02 3.035e-01
Number of obs: 35, groups:  Genotype_Zone_1:Plant_Zone_1, 10; Genotype_Zone_2:Plant_Zone_2, 10

Fixed effects:
            Estimate Std. Error       df t value Pr(>|t|)
(Intercept) -0.01951    0.09857 34.00000  -0.198    0.844

Dispersion: 0.3035
```

Model residuals

Statistic	Value
Sample skewness	-0.2515
Sample excess kurtosis	-0.141
Passed Shapiro Wilk normality test	Yes (p-value = 0.8446 > 0.05)

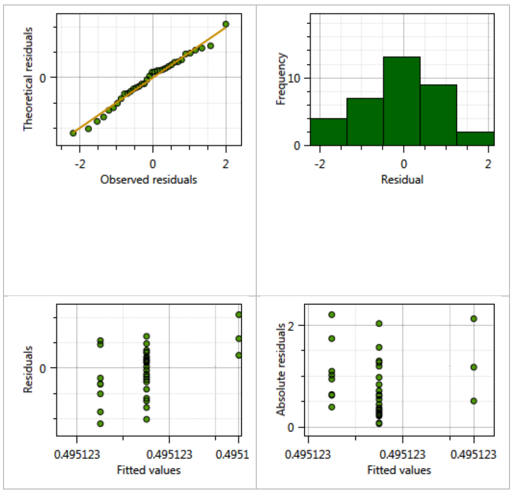

Analysis ratio movement to detection duration H4 (diff. Zone 1 - Zone 2)

**Analysis model**	Generalized linear mixed model with dispersion factor, formula=cbind(Ratio_movement_to_detection_duration_H4_Zone_1,Ratio_movement_to_detection_duration_H4_Zone_2) ~ 1 + (1|Genotype_Zone_1:Plant_Zone_1) + (1|Genotype_Zone_2:Plant_Zone_2)
**Transformation**	Logit

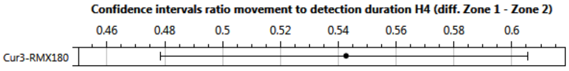

Confidence intervals ratio movement to detection duration H4 (diff. Zone 1 - Zone 2)

Genotype Zone 1	Genotype Zone 2	Mean	Lower 95% CL	Upper 95% CL	Group
Cur3	RMX180	0.5427	0.4784	0.6056	a

Model summary

```
Linear mixed model fit by REML. t-tests use Satterthwaite's method ['lmerModLmerTest']
Formula: ziFormula
   Data: data
Weights: wi

REML criterion at convergence: 52.2

Scaled residuals: 
    Min      1Q  Median      3Q     Max 
-2.6762 -0.6641  0.1961  0.6195  1.3471 

Random effects:
 Groups                        Name        Variance Std.Dev.
 Genotype_Zone_1:Plant_Zone_1 (Intercept) 0.00000  0.0000  
 Genotype_Zone_2:Plant_Zone_2 (Intercept) 0.04440  0.2107  
 Residual                                 0.05852  0.2419  
Number of obs: 30, groups:  Genotype_Zone_1:Plant_Zone_1, 10; Genotype_Zone_2:Plant_Zone_2, 10

Fixed effects:
            Estimate Std. Error     df t value Pr(>|t|)
```

```
(Intercept)   0.1711     0.1129 8.5218   1.515    0.166

Dispersion: 0.2419
```

Model residuals

Statistic	Value
Sample skewness	-0.8523
Sample excess kurtosis	0.7578
Passed Shapiro Wilk normality test	Yes (p-value = 0.1599 > 0.05)

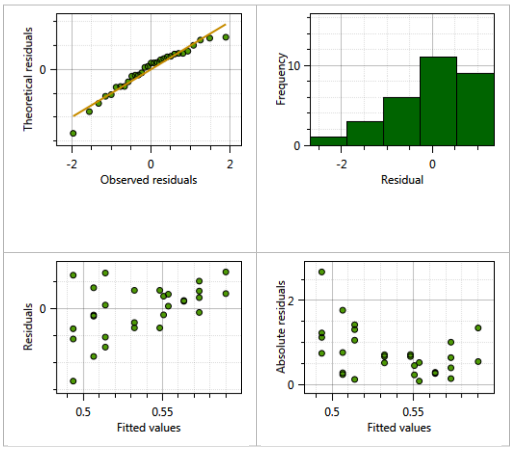

Analysis ratio movement to detection duration H5 (diff. Zone 1 - Zone 2)

Analysis model	Generalized linear mixed model with dispersion factor, formula=cbind(Ratio_movement_to_detection_duration_H5_Zone_1,Ratio_movement_to_detection_duration_H5_Zone_2) ~ 1 + (1|Genotype_Zone_1:Plant_Zone_1) + (1|Genotype_Zone_2:Plant_Zone_2)
Transformation	Logit

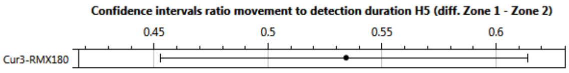

Confidence intervals ratio movement to detection duration H5 (diff. Zone 1 - Zone 2)

Genotype Zone 1	Genotype Zone 2	Mean	Lower 95% CL	Upper 95% CL	Group
Cur3	RMX180	0.5342	0.4526	0.6141	a

Model summary

```
Linear mixed model fit by REML. t-tests use Satterthwaite's method ['lmerModLmerTest']
Formula: ziFormula
   Data: data
Weights: wi

REML criterion at convergence: 71.6

Scaled residuals:
     Min       1Q   Median       3Q      Max
-1.82641 -0.86450 -0.05246  0.57713  1.98275

Random effects:
 Groups                      Name        Variance Std.Dev.
 Genotype_Zone_1:Plant_Zone_1 (Intercept) 0.07043  0.2654
 Genotype_Zone_2:Plant_Zone_2 (Intercept) 0.00000  0.0000
 Residual                                 0.10684  0.3269
Number of obs: 32, groups:  Genotype_Zone_1:Plant_Zone_1, 10; Genotype_Zone_2:Plant_Zone_2, 10

Fixed effects:
            Estimate Std. Error     df t value Pr(>|t|)
(Intercept)   0.1371     0.1443 8.8611    0.95    0.367

Dispersion: 0.3269
```

Model residuals

Statistic	Value
Sample skewness	0.06061
Sample excess kurtosis	-0.6387
Passed Shapiro Wilk normality test	Yes (p-value = 0.6971 > 0.05)

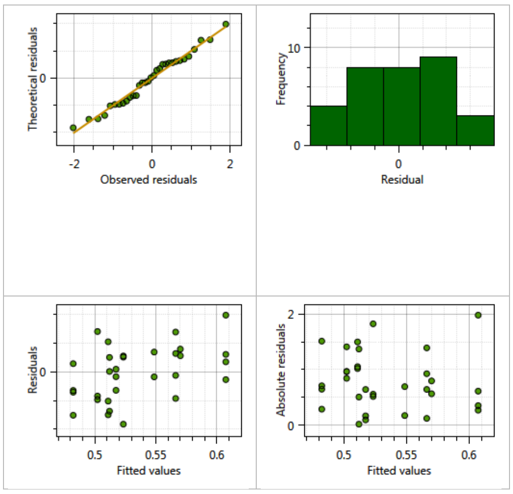

Analysis ratio movement to detection duration H6 (diff. Zone 1 - Zone 2)

Analysis model	Generalized linear mixed model with dispersion factor, formula=cbind(Ratio_movement_to_detection_duration_H6_Zone_1,Ratio_movement_to_detection_duration_H6_Zone_2) ~ 1 + (1|Genotype_Zone_1:Plant_Zone_1) + (1|Genotype_Zone_2:Plant_Zone_2)
Transformation	Logit

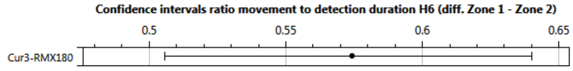

Confidence intervals ratio movement to detection duration H6 (diff. Zone 1 - Zone 2)

Genotype Zone 1	Genotype Zone 2	Mean	Lower 95% CL	Upper 95% CL	Group
Cur3	RMX180	0.5742	0.5055	0.6402	a

Model summary

```
Linear mixed model fit by REML. t-tests use Satterthwaite's method ['lmerModLmerTest']
Formula: ziFormula
   Data: data
Weights: wi

REML criterion at convergence: 63.7

Scaled residuals: 
     Min       1Q   Median       3Q      Max 
-2.22084 -0.67060 -0.02399  0.63923  1.71038 

Random effects:
 Groups                      Name        Variance Std.Dev.
 Genotype_Zone_1:Plant_Zone_1 (Intercept) 0.0000   0.0000  
 Genotype_Zone_2:Plant_Zone_2 (Intercept) 0.0000   0.0000  
 Residual                                0.1278   0.3576  
Number of obs: 28, groups:  Genotype_Zone_1:Plant_Zone_1, 10; Genotype_Zone_2:Plant_Zone_2, 10

Fixed effects:
            Estimate Std. Error      df t value Pr(>|t|)  
(Intercept)   0.2991     0.1350 27.0000   2.216   0.0353 *
---
Signif. codes:  0 '***' 0.001 '**' 0.01 '*' 0.05 '.' 0.1 ' ' 1

Dispersion: 0.3576
```

Model residuals

Statistic	Value
Sample skewness	-0.5219
Sample excess kurtosis	-0.1067
Passed Shapiro Wilk normality test	Yes (p-value = 0.3846 > 0.05)

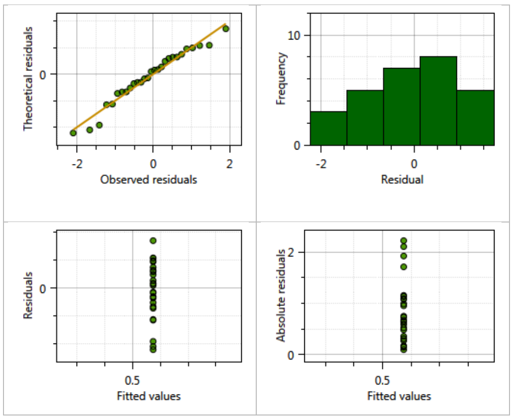

Analysis ratio movement to detection duration H7 (diff. Zone 1 - Zone 2)

**Analysis model**	Generalized linear mixed model with dispersion factor, formula=cbind(Ratio_movement_to_detection_duration_H7_Zone_1,Ratio_movement_to_detection_duration_H7_Zone_2) ~ 1 + (1|Genotype_Zone_1:Plant_Zone_1) + (1|Genotype_Zone_2:Plant_Zone_2)
**Transformation**	Logit

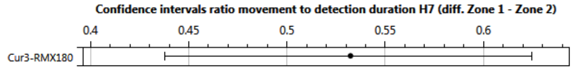

Confidence intervals ratio movement to detection duration H7 (diff. Zone 1 - Zone 2)

Genotype Zone 1	Genotype Zone 2	Mean	Lower 95% CL	Upper 95% CL	Group
Cur3	RMX180	0.5323	0.4376	0.6247	a

Model summary

```
Linear mixed model fit by REML. t-tests use Satterthwaite's method ['lmerModLmerTest']
Formula: ziFormula
   Data: data
Weights: wi

REML criterion at convergence: 66.4

Scaled residuals: 
     Min       1Q   Median       3Q      Max 
-2.28017 -0.54709 -0.07411  0.78402  1.24263 

Random effects:
 Groups                      Name        Variance Std.Dev.
 Genotype_Zone_2:Plant_Zone_2 (Intercept) 0.0991   0.3148  
 Genotype_Zone_1:Plant_Zone_1 (Intercept) 0.0000   0.0000  
 Residual                                0.0977   0.3126  
Number of obs: 30, groups:  Genotype_Zone_2:Plant_Zone_2, 10; Genotype_Zone_1:Plant_Zone_1, 9

Fixed effects:
            Estimate Std. Error     df t value Pr(>|t|)
(Intercept)   0.1293     0.1571 6.2792   0.823    0.441

Dispersion: 0.3126
```

Model residuals

Statistic	Value
Sample skewness	-0.5043
Sample excess kurtosis	-0.1685
Passed Shapiro Wilk normality test	Yes (p-value = 0.1371 > 0.05)

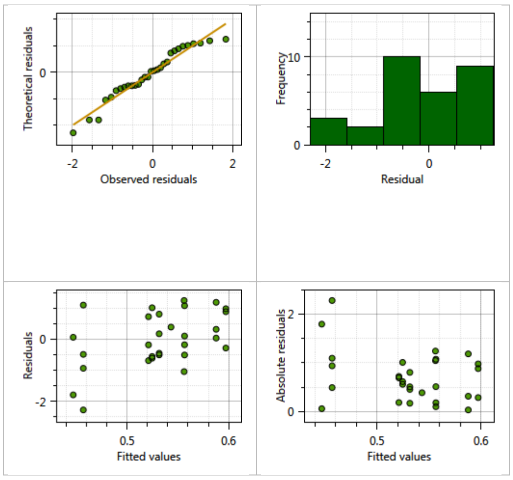

Ratio movement to halting duration per zone

Selected zones	Zone 1, Zone 2
Data transformation	Natural logarithm
Analysis	Zone difference analysis

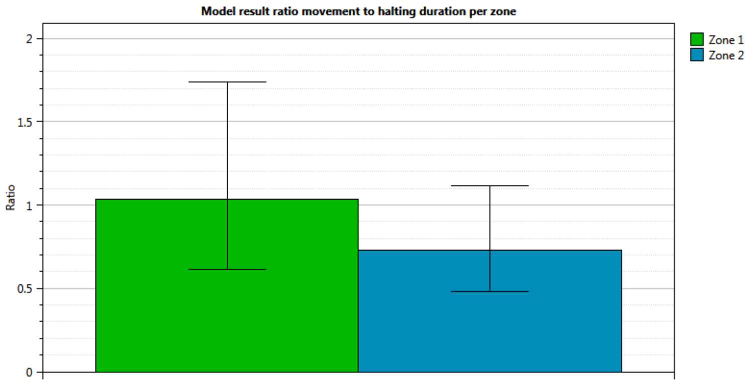

Results difference tests Zone 1 - Zone 2: p values and 95% confidence intervals of the difference on the transformed scale for each statistic.

Behaviour statistic	Cur3-RMX180
Ratio movement to halting duration (diff. Zone 1 - Zone 2)	p=0.00183**
	[0.274, 0.846]

The model predictions and 95% confidence intervals for each statistic.

Statistic	Cur3-RMX180	Remark
Ratio movement to halting duration (Zone 1)	1.03	CR
	[0.614, 1.74]	
Ratio movement to halting duration (Zone 2)	0.73	CR
	[0.479, 1.11]	

CR = Check residuals

Data summary

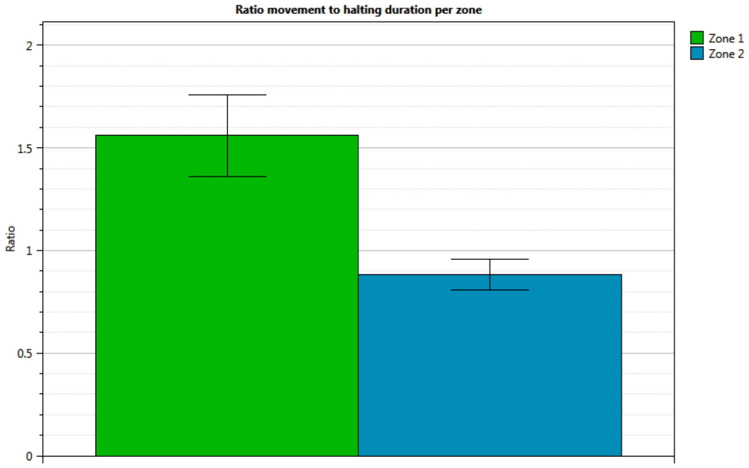

Genotype Zone 1	Genotype Zone 2	Genotype Zone 3	Mean Zone 1	StdErr Zone 1	Mean Zone 2	StdErr Zone 2
Cur3	RMX180	Neutral	1.56	0.2	0.88	0.08

Analysis ratio movement to halting duration (Zone 1)

**Analysis model**	Linear mixed model fit by REML: Ratio_movement_to_halting_duration_Zone_1 ~ 1 + (1|Genotype_Zone_1:Plant_Zone_1) + (1|Genotype_Zone_2:Plant_Zone_2)
**Transformation**	Natural logarithm

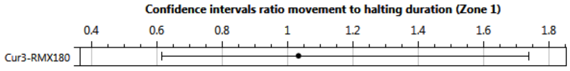

Confidence intervals ratio movement to halting duration (Zone 1)

Genotype Zone 1	Genotype Zone 2	Mean	Lower 95% CL	Upper 95% CL	Group
Cur3	RMX180	1.034	0.6144	1.74	a

Model summary

```
Linear mixed model fit by REML. t-tests use Satterthwaite's method ['lmerModLmerTest']
Formula: Ratio_movement_to_halting_duration_Zone_1 ~ 1 + (1 | Genotype_Zone_1:Plant_Zone_1) +      (1 |
Genotype_Zone_2:Plant_Zone_2)
   Data: data

REML criterion at convergence: 122.1

Scaled residuals:
    Min      1Q  Median      3Q     Max
-3.9030 -0.3888  0.2385  0.5130  1.4739

Random effects:
 Groups                       Name        Variance Std.Dev.
 Genotype_Zone_1:Plant_Zone_1 (Intercept) 0.07718  0.2778
 Genotype_Zone_2:Plant_Zone_2 (Intercept) 0.07456  0.2731
 Residual                                 1.19307  1.0923
Number of obs: 39, groups:  Genotype_Zone_1:Plant_Zone_1, 10; Genotype_Zone_2:Plant_Zone_2, 10

Fixed effects:
            Estimate Std. Error      df t value Pr(>|t|)
(Intercept)  0.03325    0.21411 6.17021   0.155    0.882
```

Model residuals

Statistic	Value
Sample skewness	-2.12
Sample excess kurtosis	6.667
Passed Shapiro Wilk normality test	No (p-value = 3.045E-05 < 0.05)

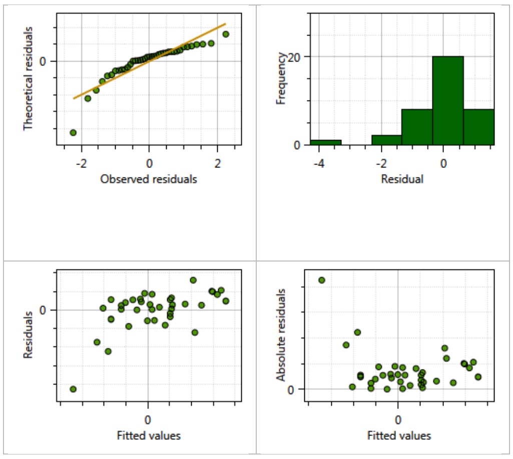

Analysis ratio movement to halting duration (Zone 2)

**Analysis model**	Linear mixed model fit by REML: Ratio_movement_to_halting_duration_Zone_2 ~ 1 + (1|Genotype_Zone_1:Plant_Zone_1) + (1|Genotype_Zone_2:Plant_Zone_2)
**Transformation**	Natural logarithm

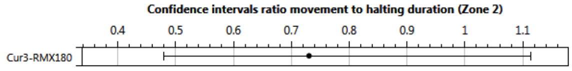

Confidence intervals ratio movement to halting duration (Zone 2)

Genotype Zone 1	Genotype Zone 2	Mean	Lower 95% CL	Upper 95% CL	Group
Cur3	RMX180	0.7305	0.4789	1.114	a

Model summary

```
Linear mixed model fit by REML. t-tests use Satterthwaite's method ['lmerModLmerTest']
Formula: Ratio_movement_to_halting_duration_Zone_2 ~ 1 + (1 | Genotype_Zone_1:Plant_Zone_1) +      (1 |
Genotype_Zone_2:Plant_Zone_2)
   Data: data

REML criterion at convergence: 88.3

Scaled residuals:
    Min      1Q  Median      3Q     Max
-3.3551 -0.2150  0.2033  0.5003  1.6114

Random effects:
 Groups                       Name        Variance  Std.Dev.
 Genotype_Zone_1:Plant_Zone_1 (Intercept) 0.0009767 0.03125
 Genotype_Zone_2:Plant_Zone_2 (Intercept) 0.0105675 0.10280
 Residual                                 0.5332202 0.73022
Number of obs: 39, groups:  Genotype_Zone_1:Plant_Zone_1, 10; Genotype_Zone_2:Plant_Zone_2, 10

Fixed effects:
            Estimate Std. Error      df t value Pr(>|t|)
(Intercept)  -0.3140     0.1218  2.6119  -2.578   0.0942 .
---
Signif. codes:  0 '***' 0.001 '**' 0.01 '*' 0.05 '.' 0.1 ' ' 1
```

Model residuals

Statistic	Value
Sample skewness	-1.78
Sample excess kurtosis	4.001
Passed Shapiro Wilk normality test	No (p-value = 2.426E-05 < 0.05)

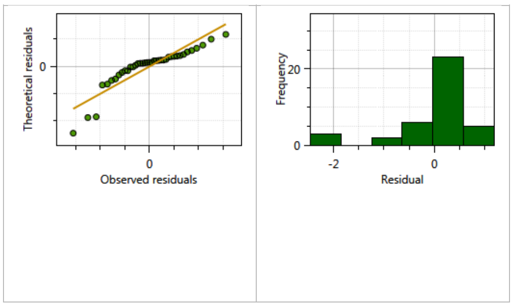

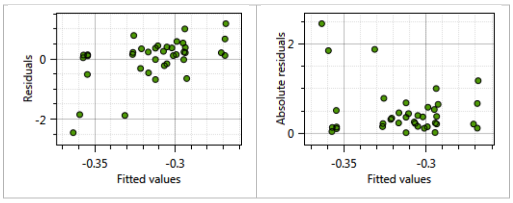

Analysis ratio movement to halting duration (diff. Zone 1 - Zone 2)

**Analysis model**	Generalized linear mixed model with dispersion factor, formula=cbind(Ratio_movement_to_halting_duration_Zone_1,Ratio_movement_to_halting_duration_Zone_2) ~ 1 + (1|Genotype_Zone_1:Plant_Zone_1) + (1|Genotype_Zone_2:Plant_Zone_2)
**Transformation**	Logit

Confidence intervals ratio movement to halting duration (diff. Zone 1 - Zone 2)

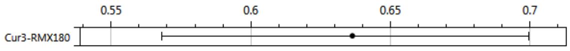

Confidence intervals ratio movement to halting duration (diff. Zone 1 - Zone 2)

Genotype Zone 1	Genotype Zone 2	Mean	Lower 95% CL	Upper 95% CL	Group
Cur3	RMX180	0.6364	0.568	0.6997	a

Model summary

```
Linear mixed model fit by REML. t-tests use Satterthwaite's method ['lmerModLmerTest']
Formula: ziFormula
   Data: data
Weights: wi

REML criterion at convergence: 98.9

Scaled residuals: 
    Min      1Q  Median      3Q     Max 
-3.1693 -0.5568 -0.1979  0.3677  2.2929 

Random effects:
 Groups                      Name        Variance Std.Dev.
 Genotype_Zone_1:Plant_Zone_1 (Intercept) 0.00000  0.0000  
 Genotype_Zone_2:Plant_Zone_2 (Intercept) 0.01201  0.1096  
 Residual                                 0.31357  0.5600  
Number of obs: 39, groups:  Genotype_Zone_1:Plant_Zone_1, 10; Genotype_Zone_2:Plant_Zone_2, 10

Fixed effects:
            Estimate Std. Error     df t value Pr(>|t|)   
(Intercept)   0.5596     0.1251 8.4151   4.474  0.00183 **
---
Signif. codes:  0 '***' 0.001 '**' 0.01 '*' 0.05 '.' 0.1 ' ' 1

Dispersion: 0.56
```

Model residuals

Statistic	Value
Sample skewness	-0.1781
Sample excess kurtosis	2.245
Passed Shapiro Wilk normality test	Yes (p-value = 0.1022 > 0.05)

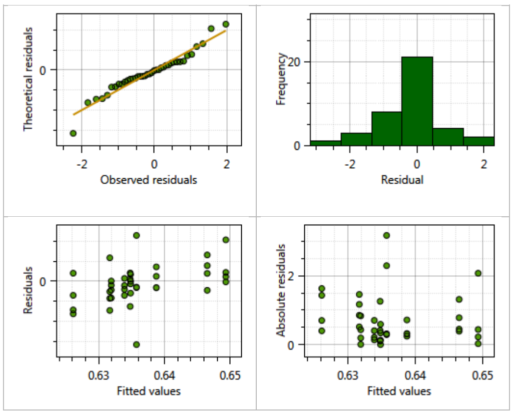

Ratio movement to halting duration per zone per hour

Selected hours	0, 1, 2, 3, 4, 5, 6, 7
Selected zones	Zone 1, Zone 2
Data transformation	Natural logarithm
Analysis	Zone difference analysis

Ratio movement to halting duration per zone per hour

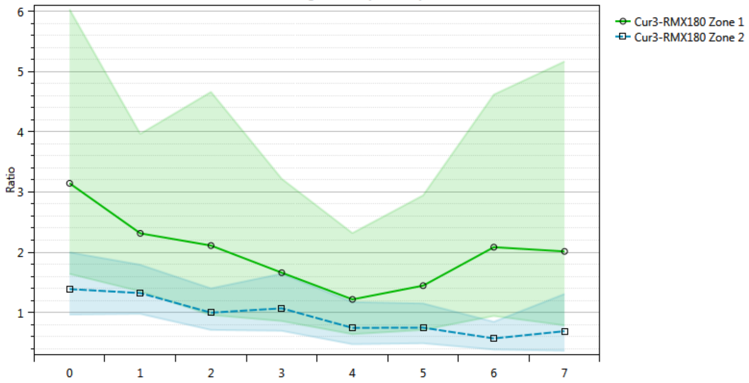

Results difference tests Zone 1 - Zone 2: p values and 95% confidence intervals of the difference on the transformed scale for each statistic.

Behaviour statistic	Cur3-RMX180

Behaviour statistic	Cur3-RMX180
Ratio movement to halting duration H0 (diff. Zone 1 - Zone 2)	p=0.0143* [0.253, 1.85]
Ratio movement to halting duration H1 (diff. Zone 1 - Zone 2)	p=0.142 [-0.311, 1.83]
Ratio movement to halting duration H2 (diff. Zone 1 - Zone 2)	p=0.0535 [-0.0158, 1.67]
Ratio movement to halting duration H3 (diff. Zone 1 - Zone 2)	p=0.196 [-0.389, 1.71]
Ratio movement to halting duration H4 (diff. Zone 1 - Zone 2)	p=0.204 [-0.397, 1.62]
Ratio movement to halting duration H5 (diff. Zone 1 - Zone 2)	p=0.0525 [-0.0149, 1.93]
Ratio movement to halting duration H6 (diff. Zone 1 - Zone 2)	p=1.31E-05**** [0.861, 1.92]
Ratio movement to halting duration H7 (diff. Zone 1 - Zone 2)	p=0.087 [-0.174, 1.98]

The model predictions and 95% confidence intervals for each statistic.

Statistic	Cur3-RMX180	Remark
Ratio movement to halting duration (H0 - Zone 1)	3.15 [1.64, 6.04]	
Ratio movement to halting duration (H0 - Zone 2)	1.39 [0.963, 2]	CR
Ratio movement to halting duration (H1 - Zone 1)	2.31 [1.35, 3.97]	
Ratio movement to halting duration (H1 - Zone 2)	1.32 [0.975, 1.79]	CR
Ratio movement to halting duration (H2 - Zone 1)	2.11 [0.956, 4.66]	
Ratio movement to halting duration (H2 - Zone 2)	0.996 [0.708, 1.4]	CR
Ratio movement to halting duration (H3 - Zone 1)	1.66 [0.856, 3.22]	CR
Ratio movement to halting duration (H3 - Zone 2)	1.07 [0.694, 1.64]	
Ratio movement to halting duration (H4 - Zone 1)	1.22 [0.638, 2.32]	
Ratio movement to halting duration (H4 - Zone 2)	0.743 [0.471, 1.17]	CR
Ratio movement to halting duration (H5 - Zone 1)	1.44 [0.708, 2.95]	
Ratio movement to halting duration (H5 - Zone 2)	0.747 [0.486, 1.15]	
Ratio movement to halting duration (H6 - Zone 1)	2.08 [0.94, 4.62]	
Ratio movement to halting duration (H6 - Zone 2)	0.568 [0.381, 0.845]	
Ratio movement to halting duration (H7 - Zone 1)	2.01 [0.784, 5.17]	
Ratio movement to halting duration (H7 - Zone 2)	0.689 [0.363, 1.31]	

CR = Check residuals

Data summary

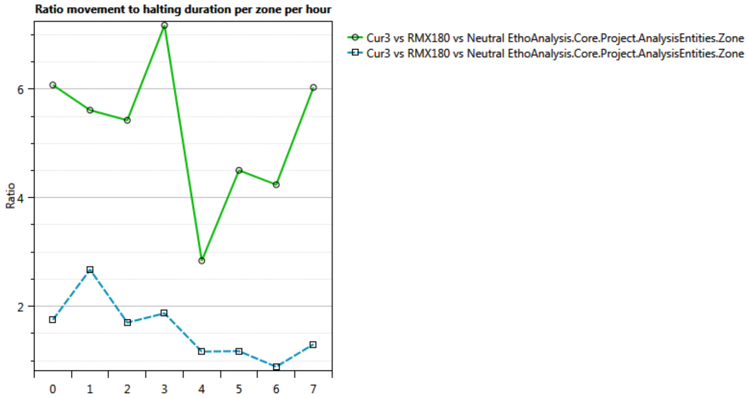

Genotype Zone 1	Genotype Zone 2	Genotype Zone 3	Mean H0 - Zone 1	StdErr H0 - Zone 1	Mean H0 - Zone 2	StdErr H0 - Zone 2	Mean H1 - Zone 1	StdErr H1 - Zone 1	Mean H1 - Zone 2	StdErr H1 - Zone 2	Mean H2 - Zone 1	StdErr H2 - Zone 1	Mean H2 - Zone 2	StdErr H2 - Zone 2
Cur3	RMX180	Neutral	6.08	1.44	1.75	0.41	5.61	2.39	2.68	1.39	5.43	1.58	1.7	0.42

Genotype Zone 1	Mean H3 - Zone 1	StdErr H3 - Zone 1	Mean H3 - Zone 2	StdErr H3 - Zone 2	Mean H4 - Zone 1	StdErr H4 - Zone 1	Mean H4 - Zone 2	StdErr H4 - Zone 2	Mean H5 - Zone 1	StdErr H5 - Zone 1	Mean H5 - Zone 2	StdErr H5 - Zone 2	Mean H6 - Zone 1	StdErr H6 - Zone 1	Mean H6 - Zone 2	StdErr H6 - Zone 2
Cur3	7.18	3.73	1.87	0.42	2.84	0.87	1.17	0.21	4.51	1.43	1.17	0.18	4.24	0.9	0.89	0.13

Analysis ratio movement to halting duration (H0 - Zone 1)

**Analysis model**	Linear mixed model fit by REML: Ratio_movement_to_halting_duration_H0_Zone_1 ~ 1 + (1|Genotype_Zone_1:Plant_Zone_1) + (1|Genotype_Zone_2:Plant_Zone_2)
**Transformation**	Natural logarithm

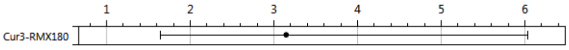

Confidence intervals ratio movement to halting duration (H0 - Zone 1)

Genotype Zone 1	Genotype Zone 2	Mean	Lower 95% CL	Upper 95% CL	Group
Cur3	RMX180	3.145	1.639	6.037	a

Model summary

```
Linear mixed model fit by REML. t-tests use Satterthwaite's method ['lmerModLmerTest']
Formula: Ratio_movement_to_halting_duration_H0_Zone_1 ~ 1 + (1 | Genotype_Zone_1:Plant_Zone_1) +      (1
| Genotype_Zone_2:Plant_Zone_2)
   Data: data

REML criterion at convergence: 101.7

Scaled residuals: 
     Min       1Q   Median       3Q      Max 
-2.45882 -0.59111 -0.09918  0.69006  1.68965 

Random effects:
 Groups                       Name        Variance Std.Dev.
 Genotype_Zone_1:Plant_Zone_1 (Intercept) 0.403    0.6349  
 Genotype_Zone_2:Plant_Zone_2 (Intercept) 0.000    0.0000  
 Residual                                 1.122    1.0591  
Number of obs: 32, groups:  Genotype_Zone_1:Plant_Zone_1, 10; Genotype_Zone_2:Plant_Zone_2, 10

Fixed effects:
            Estimate Std. Error     df t value Pr(>|t|)   
(Intercept)   1.1459     0.2787 7.3961   4.111  0.00401 **
---
Signif. codes:  0 '***' 0.001 '**' 0.01 '*' 0.05 '.' 0.1 ' ' 1
```

Model residuals

Statistic	Value
Sample skewness	-0.1699
Sample excess kurtosis	0.381
Passed Shapiro Wilk normality test	Yes (p-value = 0.3485 > 0.05)

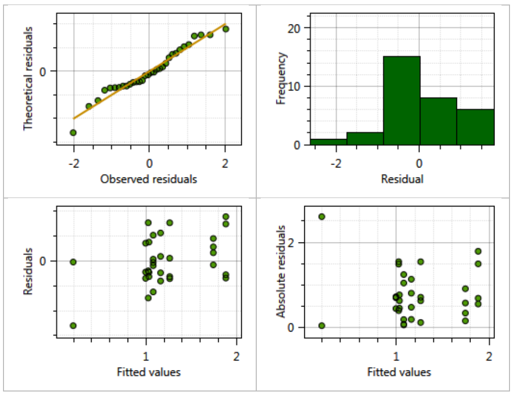

Analysis ratio movement to halting duration (H0 - Zone 2)

Analysis model	Linear mixed model fit by REML: Ratio_movement_to_halting_duration_H0_Zone_2 ~ 1 + (1|Genotype_Zone_1:Plant_Zone_1) + (1|Genotype_Zone_2:Plant_Zone_2)
Transformation	Natural logarithm

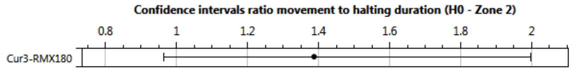

Confidence intervals ratio movement to halting duration (H0 - Zone 2)

Genotype Zone 1	Genotype Zone 2	Mean	Lower 95% CL	Upper 95% CL	Group
Cur3	RMX180	1.387	0.963	1.998	a

Model summary

```
Linear mixed model fit by REML. t-tests use Satterthwaite's method ['lmerModLmerTest']
Formula: Ratio_movement_to_halting_duration_H0_Zone_2 ~ 1 + (1 | Genotype_Zone_1:Plant_Zone_1) +      (1
| Genotype_Zone_2:Plant_Zone_2)
   Data: data

REML criterion at convergence: 73.6

Scaled residuals: 
     Min       1Q   Median       3Q      Max 
-1.67968 -0.42498 -0.03249  0.24118  2.86815 

Random effects:
 Groups                        Name        Variance  Std.Dev.
 Genotype_Zone_1:Plant_Zone_1 (Intercept) 1.860e-16 1.364e-08
 Genotype_Zone_2:Plant_Zone_2 (Intercept) 1.483e-01 3.851e-01
 Residual                                 3.644e-01 6.037e-01
Number of obs: 35, groups:  Genotype_Zone_1:Plant_Zone_1, 10; Genotype_Zone_2:Plant_Zone_2, 10

Fixed effects:
            Estimate Std. Error     df t value Pr(>|t|)  
(Intercept)   0.3273     0.1601 8.5753   2.044   0.0729 .
---
Signif. codes:  0 '***' 0.001 '**' 0.01 '*' 0.05 '.' 0.1 ' ' 1
```

Model residuals

Statistic	Value
Sample skewness	1.008
Sample excess kurtosis	2.551
Passed Shapiro Wilk normality test	No (p-value = 0.01716 < 0.05)

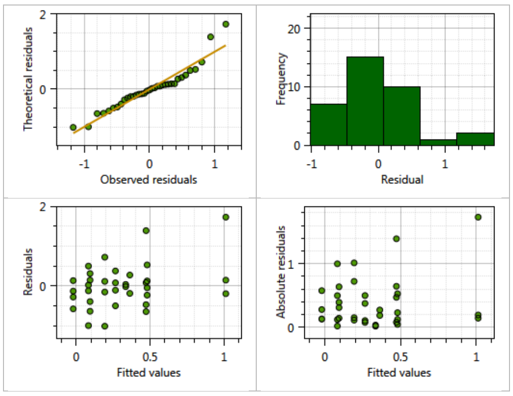

Data points with high residuals

Trial	Arena
Trial 3	37

Analysis ratio movement to halting duration (H1 - Zone 1)

Analysis model	Linear mixed model fit by REML: Ratio_movement_to_halting_duration_H1_Zone_1 ~ 1 + (1|Genotype_Zone_1:Plant_Zone_1) + (1|Genotype_Zone_2:Plant_Zone_2)
Transformation	Natural logarithm

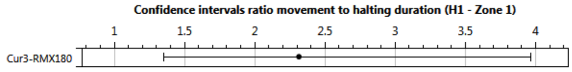

Confidence intervals ratio movement to halting duration (H1 - Zone 1)

Genotype Zone 1	Genotype Zone 2	Mean	Lower 95% CL	Upper 95% CL	Group
Cur3	RMX180	2.314	1.349	3.967	a

Model summary

```
Linear mixed model fit by REML. t-tests use Satterthwaite's method ['lmerModLmerTest']
```

```
Formula: Ratio_movement_to_halting_duration_H1_Zone_1 ~ 1 + (1 | Genotype_Zone_1:Plant_Zone_1) +      (1
| Genotype_Zone_2:Plant_Zone_2)
   Data: data

REML criterion at convergence: 115.7

Scaled residuals: 
     Min       1Q   Median       3Q      Max 
-2.47146 -0.48181  0.05113  0.57796  2.72888 

Random effects:
 Groups                       Name        Variance Std.Dev.
 Genotype_Zone_1:Plant_Zone_1 (Intercept) 0.0000   0.000   
 Genotype_Zone_2:Plant_Zone_2 (Intercept) 0.1318   0.363   
 Residual                                 1.4745   1.214   
Number of obs: 35, groups:  Genotype_Zone_1:Plant_Zone_1, 10; Genotype_Zone_2:Plant_Zone_2, 10

Fixed effects:
            Estimate Std. Error     df t value Pr(>|t|)   
(Intercept)   0.8388     0.2356 8.3533    3.56   0.0069 **
---
Signif. codes:  0 '***' 0.001 '**' 0.01 '*' 0.05 '.' 0.1 ' ' 1
```

Model residuals

Statistic	Value
Sample skewness	0.03686
Sample excess kurtosis	1.498
Passed Shapiro Wilk normality test	Yes (p-value = 0.5835 > 0.05)

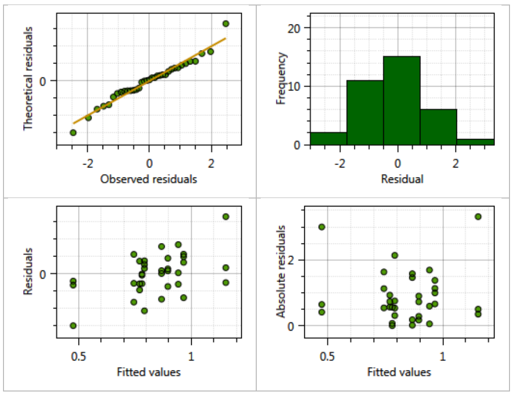

Analysis ratio movement to halting duration (H1 - Zone 2)

**Analysis model**	Linear mixed model fit by REML: Ratio_movement_to_halting_duration_H1_Zone_2 ~ 1 + (1|Genotype_Zone_1:Plant_Zone_1) + (1|Genotype_Zone_2:Plant_Zone_2)
**Transformation**	Natural logarithm

Confidence intervals ratio movement to halting duration (H1 - Zone 2)

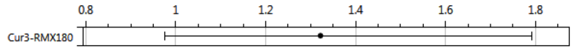

Confidence intervals ratio movement to halting duration (H1 - Zone 2)

Genotype Zone 1	Genotype Zone 2	Mean	Lower 95% CL	Upper 95% CL	Group
Cur3	RMX180	1.322	0.9747	1.792	a

Model summary

```
Linear mixed model fit by REML. t-tests use Satterthwaite's method ['lmerModLmerTest']
Formula: Ratio_movement_to_halting_duration_H1_Zone_2 ~ 1 + (1 | Genotype_Zone_1:Plant_Zone_1) +      (1
| Genotype_Zone_2:Plant_Zone_2)
   Data: data

REML criterion at convergence: 95.5

Scaled residuals: 
    Min      1Q  Median      3Q     Max 
-3.2644 -0.3188 -0.0314  0.3779  4.1197 

Random effects:
 Groups                       Name        Variance Std.Dev.
 Genotype_Zone_1:Plant_Zone_1 (Intercept) 0.0000   0.0000  
 Genotype_Zone_2:Plant_Zone_2 (Intercept) 0.0000   0.0000  
 Residual                                 0.8101   0.9001  
Number of obs: 36, groups:  Genotype_Zone_1:Plant_Zone_1, 10; Genotype_Zone_2:Plant_Zone_2, 10

Fixed effects:
            Estimate Std. Error      df t value Pr(>|t|) 
(Intercept)   0.2789     0.1500 35.0000   1.859   0.0714 .
---
Signif. codes:  0 '***' 0.001 '**' 0.01 '*' 0.05 '.' 0.1 ' ' 1
```

Model residuals

Statistic	Value
Sample skewness	1.008
Sample excess kurtosis	10.49
Passed Shapiro Wilk normality test	No (p-value = 2.146E-06 < 0.05)

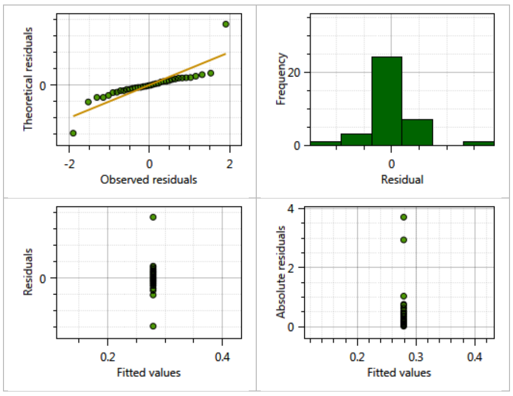

Data points with high residuals

Trial	Arena
Trial 3	14

Analysis ratio movement to halting duration (H2 - Zone 1)

**Analysis model**	Linear mixed model fit by REML: Ratio_movement_to_halting_duration_H2_Zone_1 ~ 1 + (1|Genotype_Zone_1:Plant_Zone_1) + (1|Genotype_Zone_2:Plant_Zone_2)
**Transformation**	Natural logarithm

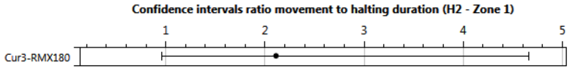

Confidence intervals ratio movement to halting duration (H2 - Zone 1)

Genotype Zone 1	Genotype Zone 2	Mean	Lower 95% CL	Upper 95% CL	Group
Cur3	RMX180	2.111	0.9556	4.664	a

Model summary

```
Linear mixed model fit by REML. t-tests use Satterthwaite's method ['lmerModLmerTest']
Formula: Ratio_movement_to_halting_duration_H2_Zone_1 ~ 1 + (1 | Genotype_Zone_1:Plant_Zone_1) +      (1
| Genotype_Zone_2:Plant_Zone_2)
   Data: data

REML criterion at convergence: 126.9

Scaled residuals: 
    Min      1Q  Median      3Q     Max 
-1.9784 -0.8379  0.2246  0.6729  1.6448 

Random effects:
 Groups                       Name        Variance Std.Dev.
 Genotype_Zone_1:Plant_Zone_1 (Intercept) 0.005956 0.07717 
 Genotype_Zone_2:Plant_Zone_2 (Intercept) 0.634259 0.79640 
 Residual                                 1.774460 1.33209 
Number of obs: 35, groups:  Genotype_Zone_1:Plant_Zone_1, 10; Genotype_Zone_2:Plant_Zone_2, 10

Fixed effects:
            Estimate Std. Error     df t value Pr(>|t|)  
(Intercept)   0.7472     0.3396 7.4801     2.2   0.0613 .
---
Signif. codes:  0 '***' 0.001 '**' 0.01 '*' 0.05 '.' 0.1 ' ' 1
```

Model residuals

Statistic	Value
Sample skewness	-0.2247
Sample excess kurtosis	-0.9037
Passed Shapiro Wilk normality test	Yes (p-value = 0.3448 > 0.05)

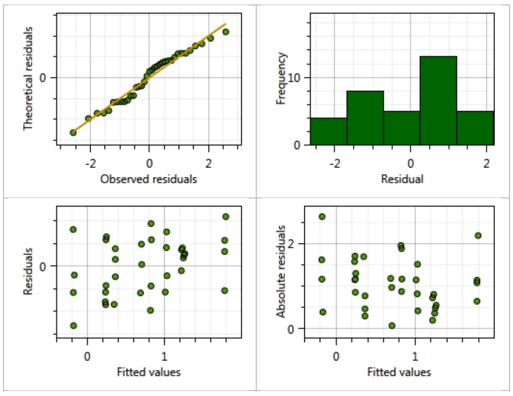

Analysis ratio movement to halting duration (H2 - Zone 2)

**Analysis model**	Linear mixed model fit by REML: Ratio_movement_to_halting_duration_H2_Zone_2 ~ 1 + (1|Genotype_Zone_1:Plant_Zone_1) + (1|Genotype_Zone_2:Plant_Zone_2)
**Transformation**	Natural logarithm

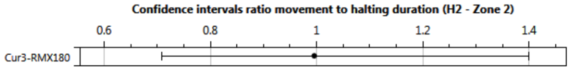

Confidence intervals ratio movement to halting duration (H2 - Zone 2)

Genotype Zone 1	Genotype Zone 2	Mean	Lower 95% CL	Upper 95% CL	Group
Cur3	RMX180	0.9955	0.7079	1.4	a

Model summary

```
Linear mixed model fit by REML. t-tests use Satterthwaite's method ['lmerModLmerTest']
Formula: Ratio_movement_to_halting_duration_H2_Zone_2 ~ 1 + (1 | Genotype_Zone_1:Plant_Zone_1) +      (1
| Genotype_Zone_2:Plant_Zone_2)
   Data: data

REML criterion at convergence: 107.4

Scaled residuals: 
     Min       1Q   Median       3Q      Max 
-2.59637 -0.56268 -0.04578  0.31938  2.41403 

Random effects:
 Groups                       Name        Variance Std.Dev.
 Genotype_Zone_1:Plant_Zone_1 (Intercept) 0.000    0.000   
 Genotype_Zone_2:Plant_Zone_2 (Intercept) 0.000    0.000   
 Residual                                 1.045    1.022   
Number of obs: 37, groups:  Genotype_Zone_1:Plant_Zone_1, 10; Genotype_Zone_2:Plant_Zone_2, 10

Fixed effects:
            Estimate Std. Error       df t value Pr(>|t|)
(Intercept) -0.00448    0.16810 36.00000  -0.027    0.979
```

Model residuals

Statistic	Value
Sample skewness	0.07732
Sample excess kurtosis	1.942
Passed Shapiro Wilk normality test	No (p-value = 0.01317 < 0.05)

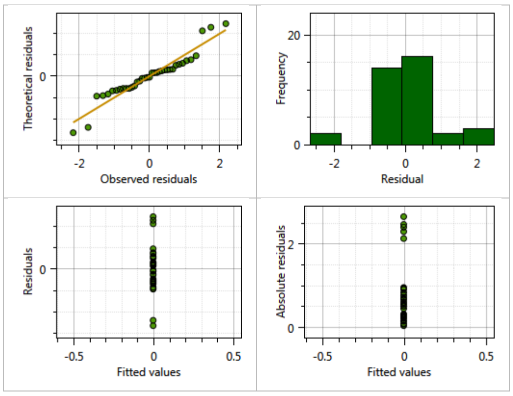

Analysis ratio movement to halting duration (H3 - Zone 1)

**Analysis model**	Linear mixed model fit by REML: Ratio_movement_to_halting_duration_H3_Zone_1 ~ 1 + (1|Genotype_Zone_1:Plant_Zone_1) + (1|Genotype_Zone_2:Plant_Zone_2)
**Transformation**	Natural logarithm

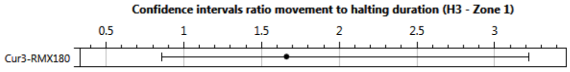

Confidence intervals ratio movement to halting duration (H3 - Zone 1)

Genotype Zone 1	Genotype Zone 2	Mean	Lower 95% CL	Upper 95% CL	Group
Cur3	RMX180	1.66	0.8556	3.221	a

Model summary

```
Linear mixed model fit by REML. t-tests use Satterthwaite's method ['lmerModLmerTest']
Formula: Ratio_movement_to_halting_duration_H3_Zone_1 ~ 1 + (1 | Genotype_Zone_1:Plant_Zone_1) +      (1
| Genotype_Zone_2:Plant_Zone_2)
   Data: data

REML criterion at convergence: 116.6

Scaled residuals:
     Min       1Q   Median       3Q      Max
-1.53588 -0.63676 -0.04659  0.37418  2.66943

Random effects:
 Groups                       Name        Variance Std.Dev.
 Genotype_Zone_1:Plant_Zone_1 (Intercept) 0.0000   0.0000
 Genotype_Zone_2:Plant_Zone_2 (Intercept) 0.1698   0.4121
 Residual                                 2.1080   1.4519
Number of obs: 32, groups:  Genotype_Zone_1:Plant_Zone_1, 10; Genotype_Zone_2:Plant_Zone_2, 10

Fixed effects:
            Estimate Std. Error     df t value Pr(>|t|)
(Intercept)   0.5069     0.2892 8.2862   1.753    0.116
```

Model residuals

Statistic	Value
Sample skewness	0.9606
Sample excess kurtosis	1.304
Passed Shapiro Wilk normality test	No (p-value = 0.036 < 0.05)

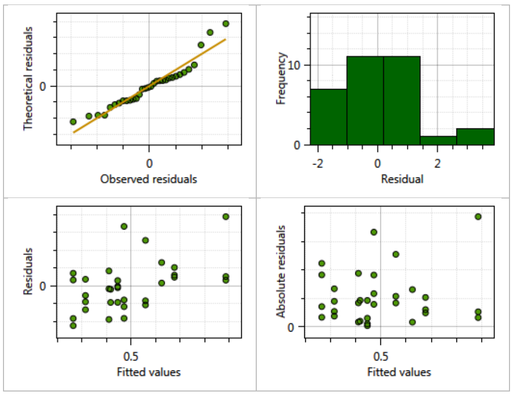

Analysis ratio movement to halting duration (H3 - Zone 2)

**Analysis model**	Linear mixed model fit by REML: Ratio_movement_to_halting_duration_H3_Zone_2 ~ 1 + (1|Genotype_Zone_1:Plant_Zone_1) + (1|Genotype_Zone_2:Plant_Zone_2)
**Transformation**	Natural logarithm

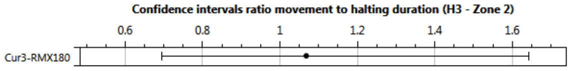

Confidence intervals ratio movement to halting duration (H3 - Zone 2)

Genotype Zone 1	Genotype Zone 2	Mean	Lower 95% CL	Upper 95% CL	Group
Cur3	RMX180	1.068	0.6937	1.644	a

Model summary

```
Linear mixed model fit by REML. t-tests use Satterthwaite's method ['lmerModLmerTest']
Formula: Ratio_movement_to_halting_duration_H3_Zone_2 ~ 1 + (1 | Genotype_Zone_1:Plant_Zone_1) +      (1
| Genotype_Zone_2:Plant_Zone_2)
   Data: data

REML criterion at convergence: 118.9

Scaled residuals:
     Min       1Q   Median       3Q      Max
-2.64838 -0.42157  0.03559  0.52667  2.32545

Random effects:
 Groups                       Name        Variance Std.Dev.
 Genotype_Zone_1:Plant_Zone_1 (Intercept) 0.00000  0.0000
 Genotype_Zone_2:Plant_Zone_2 (Intercept) 0.06999  0.2646
 Residual                                 1.15496  1.0747
Number of obs: 39, groups:  Genotype_Zone_1:Plant_Zone_1, 10; Genotype_Zone_2:Plant_Zone_2, 10
```

```
Fixed effects:
            Estimate Std. Error      df t value Pr(>|t|)
(Intercept)  0.06562    0.19144 9.25215   0.343    0.739
```

Model residuals

Statistic	Value
Sample skewness	-0.3751
Sample excess kurtosis	1.171
Passed Shapiro Wilk normality test	Yes (p-value = 0.2917 > 0.05)

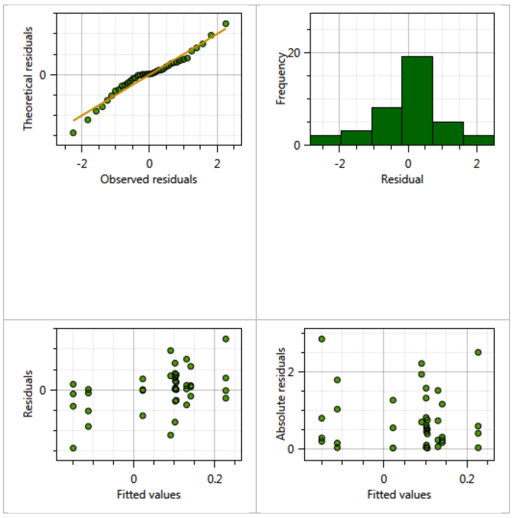

Analysis ratio movement to halting duration (H4 - Zone 1)

**Analysis model**	Linear mixed model fit by REML: Ratio_movement_to_halting_duration_H4_Zone_1 ~ 1 + (1|Genotype_Zone_1:Plant_Zone_1) + (1|Genotype_Zone_2:Plant_Zone_2)
**Transformation**	Natural logarithm

Confidence intervals ratio movement to halting duration (H4 - Zone 1)

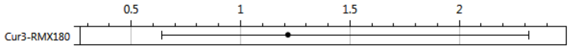

Confidence intervals ratio movement to halting duration (H4 - Zone 1)

Genotype Zone 1	Genotype Zone 2	Mean	Lower 95% CL	Upper 95% CL	Group
Cur3	RMX180	1.216	0.6381	2.319	a

Model summary

```
Linear mixed model fit by REML. t-tests use Satterthwaite's method ['lmerModLmerTest']
Formula: Ratio_movement_to_halting_duration_H4_Zone_1 ~ 1 + (1 | Genotype_Zone_1:Plant_Zone_1) +      (1
| Genotype_Zone_2:Plant_Zone_2)
   Data: data

REML criterion at convergence: 102.5

Scaled residuals:
     Min       1Q   Median       3Q      Max
-1.88135 -0.44860  0.05438  0.63338  2.09220

Random effects:
 Groups                       Name        Variance Std.Dev.
 Genotype_Zone_1:Plant_Zone_1 (Intercept) 0.00000  0.000
 Genotype_Zone_2:Plant_Zone_2 (Intercept) 0.04794  0.219
 Residual                                 1.97950  1.407
Number of obs: 29, groups:  Genotype_Zone_1:Plant_Zone_1, 10; Genotype_Zone_2:Plant_Zone_2, 10

Fixed effects:
            Estimate Std. Error     df t value Pr(>|t|)
(Intercept)   0.1959     0.2712 6.7997   0.722    0.494
```

Model residuals

Statistic	Value
Sample skewness	-0.2951
Sample excess kurtosis	-0.118
Passed Shapiro Wilk normality test	Yes (p-value = 0.487 > 0.05)

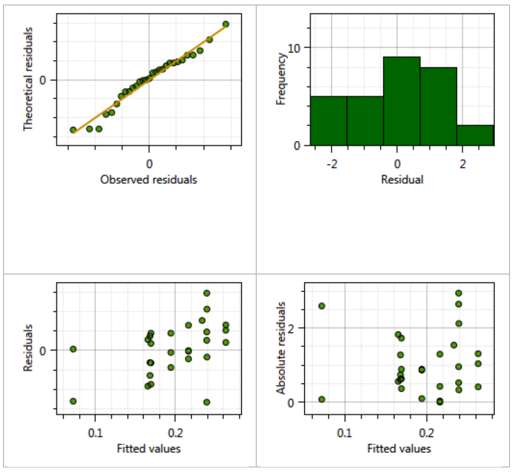

Analysis ratio movement to halting duration (H4 - Zone 2)

**Analysis model**	Linear mixed model fit by REML: Ratio_movement_to_halting_duration_H4_Zone_2 ~ 1 + (1|Genotype_Zone_1:Plant_Zone_1) + (1|Genotype_Zone_2:Plant_Zone_2)
**Transformation**	Natural logarithm

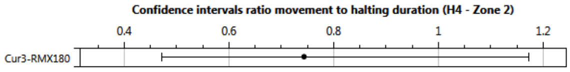

Confidence intervals ratio movement to halting duration (H4 - Zone 2)

Genotype Zone 1	Genotype Zone 2	Mean	Lower 95% CL	Upper 95% CL	Group
Cur3	RMX180	0.7433	0.4709	1.173	a

Model summary

```
Linear mixed model fit by REML. t-tests use Satterthwaite's method ['lmerModLmerTest']
Formula: Ratio_movement_to_halting_duration_H4_Zone_2 ~ 1 + (1 | Genotype_Zone_1:Plant_Zone_1) +      (1
| Genotype_Zone_2:Plant_Zone_2)
   Data: data

REML criterion at convergence: 106

Scaled residuals: 
    Min      1Q  Median      3Q     Max 
-2.3500 -0.2924  0.1325  0.4705  2.0109 

Random effects:
 Groups                       Name        Variance Std.Dev.
 Genotype_Zone_1:Plant_Zone_1 (Intercept) 0.0000   0.0000  
 Genotype_Zone_2:Plant_Zone_2 (Intercept) 0.1262   0.3552  
 Residual                                 0.9083   0.9530  
Number of obs: 37, groups:  Genotype_Zone_1:Plant_Zone_1, 10; Genotype_Zone_2:Plant_Zone_2, 10

Fixed effects:
            Estimate Std. Error      df t value Pr(>|t|)
(Intercept)  -0.2967     0.1935  7.0931  -1.533    0.169
```

Model residuals

Statistic	Value
Sample skewness	-0.6693
Sample excess kurtosis	0.9096
Passed Shapiro Wilk normality test	No (p-value = 0.03411 < 0.05)

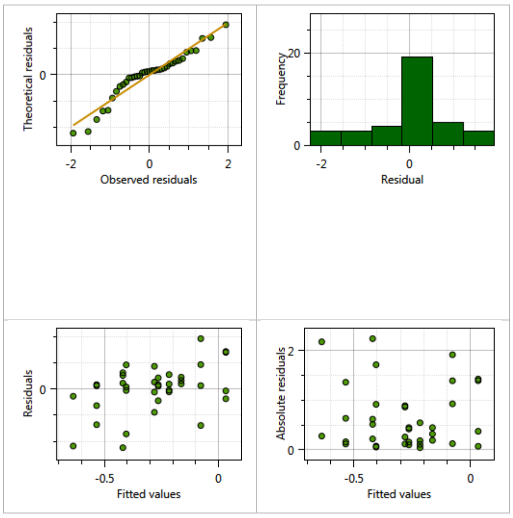

Analysis ratio movement to halting duration (H5 - Zone 1)

**Analysis model**	Linear mixed model fit by REML: Ratio_movement_to_halting_duration_H5_Zone_1 ~ 1 + (1|Genotype_Zone_1:Plant_Zone_1) + (1|Genotype_Zone_2:Plant_Zone_2)
**Transformation**	Natural logarithm

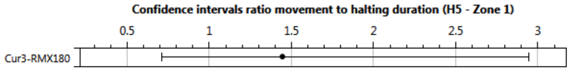

Confidence intervals ratio movement to halting duration (H5 - Zone 1)

Genotype Zone 1	Genotype Zone 2	Mean	Lower 95% CL	Upper 95% CL	Group
Cur3	RMX180	1.444	0.7082	2.946	a

Model summary

```
Linear mixed model fit by REML. t-tests use Satterthwaite's method ['lmerModLmerTest']
Formula: Ratio_movement_to_halting_duration_H5_Zone_1 ~ 1 + (1 | Genotype_Zone_1:Plant_Zone_1) +      (1
| Genotype_Zone_2:Plant_Zone_2)
   Data: data

REML criterion at convergence: 111.1

Scaled residuals: 
    Min      1Q  Median      3Q     Max 
-1.8358 -0.6601 -0.1754  0.5099  1.9986 

Random effects:
 Groups                       Name        Variance Std.Dev.
 Genotype_Zone_1:Plant_Zone_1 (Intercept) 0.02601  0.1613  
 Genotype_Zone_2:Plant_Zone_2 (Intercept) 0.01011  0.1006  
 Residual                                 2.36754  1.5387  
Number of obs: 30, groups:  Genotype_Zone_1:Plant_Zone_1, 10; Genotype_Zone_2:Plant_Zone_2, 10

Fixed effects:
            Estimate Std. Error     df t value Pr(>|t|)
(Intercept)   0.3677     0.2877 5.7100   1.278    0.251
```

Model residuals

Statistic	Value
Sample skewness	0.3805
Sample excess kurtosis	-0.2836
Passed Shapiro Wilk normality test	Yes (p-value = 0.3021 > 0.05)

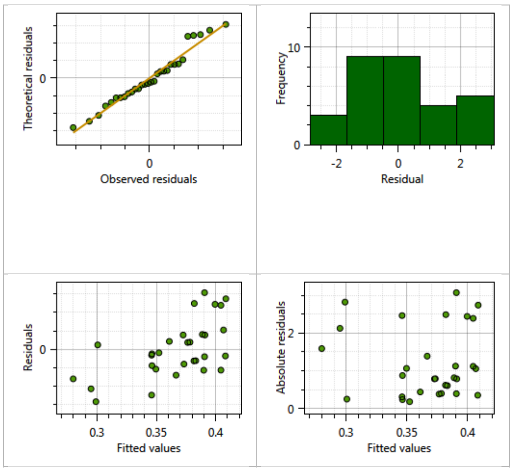

Analysis ratio movement to halting duration (H5 - Zone 2)

**Analysis model**	Linear mixed model fit by REML: Ratio_movement_to_halting_duration_H5_Zone_2 ~ 1 + (1|Genotype_Zone_1:Plant_Zone_1) + (1|Genotype_Zone_2:Plant_Zone_2)
**Transformation**	Natural logarithm

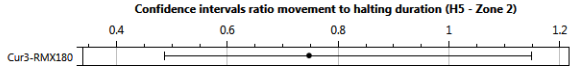

Confidence intervals ratio movement to halting duration (H5 - Zone 2)

Genotype Zone 1	Genotype Zone 2	Mean	Lower 95% CL	Upper 95% CL	Group
Cur3	RMX180	0.7474	0.486	1.149	a

Model summary

```
Linear mixed model fit by REML. t-tests use Satterthwaite's method ['lmerModLmerTest']
Formula: Ratio_movement_to_halting_duration_H5_Zone_2 ~ 1 + (1 | Genotype_Zone_1:Plant_Zone_1) +      (1
| Genotype_Zone_2:Plant_Zone_2)
   Data: data

REML criterion at convergence: 113.6

Scaled residuals:
    Min      1Q  Median      3Q     Max
-2.6723 -0.6405  0.0773  0.6319  1.7448

Random effects:
 Groups                        Name        Variance Std.Dev.
 Genotype_Zone_1:Plant_Zone_1 (Intercept) 0.1173   0.3424
 Genotype_Zone_2:Plant_Zone_2 (Intercept) 0.0000   0.0000
 Residual                                 0.9639   0.9818
Number of obs: 39, groups:  Genotype_Zone_1:Plant_Zone_1, 10; Genotype_Zone_2:Plant_Zone_2, 10

Fixed effects:
            Estimate Std. Error      df t value Pr(>|t|)
(Intercept)  -0.2912     0.1910  9.2404  -1.524    0.161
```

Model residuals

Statistic	Value
Sample skewness	-0.4197
Sample excess kurtosis	0.2561
Passed Shapiro Wilk normality test	Yes (p-value = 0.5416 > 0.05)

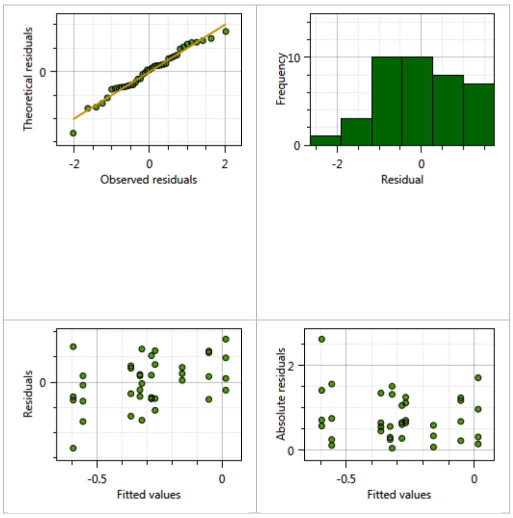

Analysis ratio movement to halting duration (H6 - Zone 1)

**Analysis model**	Linear mixed model fit by REML: Ratio_movement_to_halting_duration_H6_Zone_1 ~ 1 + (1|Genotype_Zone_1:Plant_Zone_1) + (1|Genotype_Zone_2:Plant_Zone_2)
**Transformation**	Natural logarithm

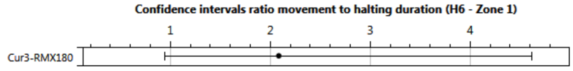

Confidence intervals ratio movement to halting duration (H6 - Zone 1)

Genotype Zone 1	Genotype Zone 2	Mean	Lower 95% CL	Upper 95% CL	Group
Cur3	RMX180	2.084	0.9403	4.62	a

Model summary

```
Linear mixed model fit by REML. t-tests use Satterthwaite's method ['lmerModLmerTest']
Formula: Ratio_movement_to_halting_duration_H6_Zone_1 ~ 1 + (1 | Genotype_Zone_1:Plant_Zone_1) +      (1
| Genotype_Zone_2:Plant_Zone_2)
   Data: data
```

```
REML criterion at convergence: 95.8

Scaled residuals:
    Min      1Q  Median      3Q     Max
-1.8271 -0.5980  0.2741  0.6134  1.6214

Random effects:
 Groups                      Name        Variance Std.Dev.
 Genotype_Zone_1:Plant_Zone_1 (Intercept) 0.0000   0.000
 Genotype_Zone_2:Plant_Zone_2 (Intercept) 0.5112   0.715
 Residual                                 1.6849   1.298
Number of obs: 27, groups:  Genotype_Zone_1:Plant_Zone_1, 10; Genotype_Zone_2:Plant_Zone_2, 10

Fixed effects:
            Estimate Std. Error     df t value Pr(>|t|)
(Intercept)   0.7344     0.3450 7.9792   2.129    0.066 .
---
Signif. codes:  0 '***' 0.001 '**' 0.01 '*' 0.05 '.' 0.1 ' ' 1
```

Model residuals

Statistic	Value
Sample skewness	-0.5154
Sample excess kurtosis	-0.4743
Passed Shapiro Wilk normality test	Yes (p-value = 0.2069 > 0.05)

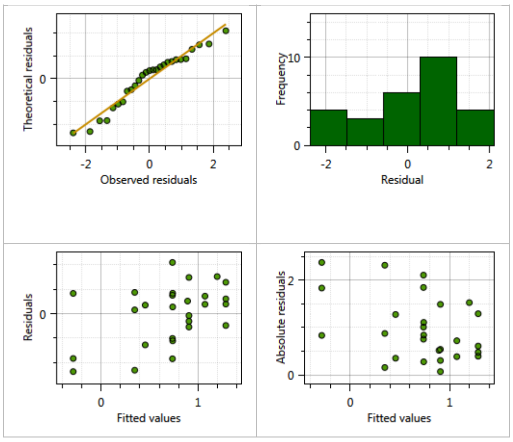

Analysis ratio movement to halting duration (H6 - Zone 2)

**Analysis model**	Linear mixed model fit by REML: Ratio_movement_to_halting_duration_H6_Zone_2 ~ 1 + (1|Genotype_Zone_1:Plant_Zone_1) + (1|Genotype_Zone_2:Plant_Zone_2)
**Transformation**	Natural logarithm

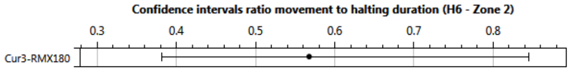

Confidence intervals ratio movement to halting duration (H6 - Zone 2)

Genotype Zone 1	Genotype Zone 2	Mean	Lower 95% CL	Upper 95% CL	Group
Cur3	RMX180	0.5676	0.3811	0.8453	a

Model summary

```
Linear mixed model fit by REML. t-tests use Satterthwaite's method ['lmerModLmerTest']
Formula: Ratio_movement_to_halting_duration_H6_Zone_2 ~ 1 + (1 | Genotype_Zone_1:Plant_Zone_1) +      (1
| Genotype_Zone_2:Plant_Zone_2)
   Data: data

REML criterion at convergence: 114.4

Scaled residuals:
    Min      1Q  Median      3Q     Max
-3.0905 -0.6249  0.2019  0.5668  1.7192

Random effects:
 Groups                      Name        Variance  Std.Dev.
 Genotype_Zone_1:Plant_Zone_1 (Intercept) 3.749e-03 6.123e-02
 Genotype_Zone_2:Plant_Zone_2 (Intercept) 7.444e-17 8.628e-09
 Residual                                 1.165e+00 1.079e+00
Number of obs: 38, groups:  Genotype_Zone_1:Plant_Zone_1, 10; Genotype_Zone_2:Plant_Zone_2, 10

Fixed effects:
            Estimate Std. Error      df t value Pr(>|t|)
(Intercept)  -0.5664     0.1762  9.0210  -3.215   0.0105 *
---
Signif. codes:  0 '***' 0.001 '**' 0.01 '*' 0.05 '.' 0.1 ' ' 1
```

Model residuals

Statistic	Value
Sample skewness	-0.8394
Sample excess kurtosis	1.153
Passed Shapiro Wilk normality test	Yes (p-value = 0.1056 > 0.05)

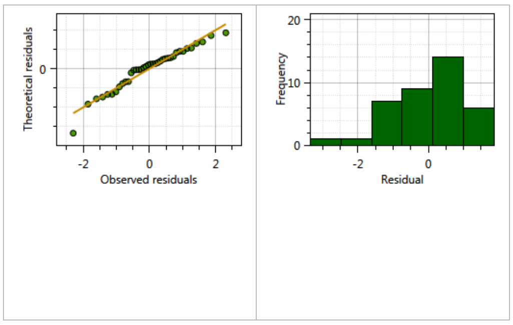

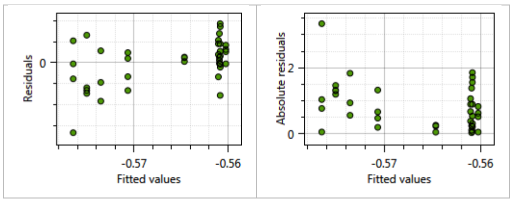

Analysis ratio movement to halting duration (H7 - Zone 1)

**Analysis model**	Linear mixed model fit by REML: Ratio_movement_to_halting_duration_H7_Zone_1 ~ 1 + (1|Genotype_Zone_1:Plant_Zone_1) + (1|Genotype_Zone_2:Plant_Zone_2)
**Transformation**	Natural logarithm

Confidence intervals ratio movement to halting duration (H7 - Zone 1)

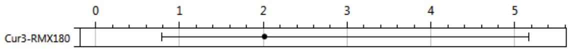

Confidence intervals ratio movement to halting duration (H7 - Zone 1)

Genotype Zone 1	Genotype Zone 2	Mean	Lower 95% CL	Upper 95% CL	Group
Cur3	RMX180	2.014	0.7844	5.17	a

Model summary

```
Linear mixed model fit by REML. t-tests use Satterthwaite's method ['lmerModLmerTest']
Formula: Ratio_movement_to_halting_duration_H7_Zone_1 ~ 1 + (1 | Genotype_Zone_1:Plant_Zone_1) +      (1
| Genotype_Zone_2:Plant_Zone_2)
   Data: data

REML criterion at convergence: 98.2

Scaled residuals:
     Min       1Q   Median       3Q      Max
-1.99007 -0.49441 -0.09405  0.51653  1.74936

Random effects:
 Groups                       Name        Variance Std.Dev.
 Genotype_Zone_2:Plant_Zone_2 (Intercept) 0.8425   0.9179
 Genotype_Zone_1:Plant_Zone_1 (Intercept) 0.1879   0.4335
 Residual                                 1.5661   1.2514
Number of obs: 27, groups:  Genotype_Zone_2:Plant_Zone_2, 10; Genotype_Zone_1:Plant_Zone_1, 9

Fixed effects:
            Estimate Std. Error     df t value Pr(>|t|)
(Intercept)   0.7000     0.4166 8.9663    1.68    0.127
```

Model residuals

Statistic	Value
Sample skewness	0.1457
Sample excess kurtosis	0.2823
Passed Shapiro Wilk normality test	Yes (p-value = 0.7807 > 0.05)

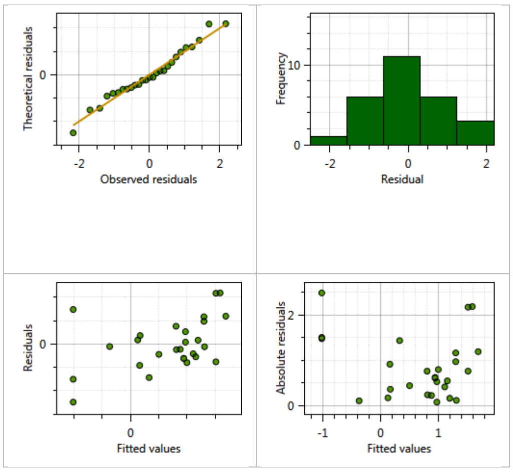

Analysis ratio movement to halting duration (H7 - Zone 2)

**Analysis model**	Linear mixed model fit by REML: Ratio_movement_to_halting_duration_H7_Zone_2 ~ 1 + (1|Genotype_Zone_1:Plant_Zone_1) + (1|Genotype_Zone_2:Plant_Zone_2)
**Transformation**	Natural logarithm

Confidence intervals ratio movement to halting duration (H7 - Zone 2)

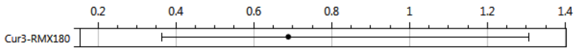

Confidence intervals ratio movement to halting duration (H7 - Zone 2)

Genotype Zone 1	Genotype Zone 2	Mean	Lower 95% CL	Upper 95% CL	Group
Cur3	RMX180	0.6886	0.3628	1.307	a

Model summary

```
Linear mixed model fit by REML. t-tests use Satterthwaite's method ['lmerModLmerTest']
Formula: Ratio_movement_to_halting_duration_H7_Zone_2 ~ 1 + (1 | Genotype_Zone_1:Plant_Zone_1) +      (1
| Genotype_Zone_2:Plant_Zone_2)
   Data: data

REML criterion at convergence: 125.7

Scaled residuals:
     Min       1Q   Median       3Q      Max
-2.32854 -0.53379  0.02204  0.57527  1.68287

Random effects:
 Groups                       Name        Variance Std.Dev.
 Genotype_Zone_1:Plant_Zone_1 (Intercept) 0.27859  0.5278
 Genotype_Zone_2:Plant_Zone_2 (Intercept) 0.09625  0.3102
 Residual                                 1.17487  1.0839
Number of obs: 39, groups:  Genotype_Zone_1:Plant_Zone_1, 10; Genotype_Zone_2:Plant_Zone_2, 10

Fixed effects:
            Estimate Std. Error      df t value Pr(>|t|)
(Intercept)  -0.3731     0.2603  5.8514  -1.433    0.203
```

Model residuals

Statistic	Value
Sample skewness	-0.1273
Sample excess kurtosis	-0.0672
Passed Shapiro Wilk normality test	Yes (p-value = 0.879 > 0.05)

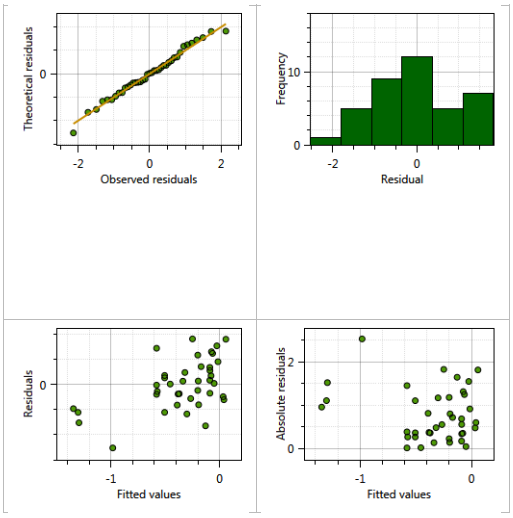

Analysis ratio movement to halting duration H0 (diff. Zone 1 - Zone 2)

Analysis model	Generalized linear mixed model with dispersion factor, formula=cbind(Ratio_movement_to_halting_duration_H0_Zone_1,Ratio_movement_to_halting_duration_H0_Zone_2) ~ 1 + (1|Genotype_Zone_1:Plant_Zone_1) + (1|Genotype_Zone_2:Plant_Zone_2)
**Transformation**	Logit

Confidence intervals ratio movement to halting duration H0 (diff. Zone 1 - Zone 2)

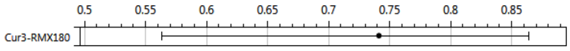

Confidence intervals ratio movement to halting duration H0 (diff. Zone 1 - Zone 2)

Genotype Zone 1	Genotype Zone 2	Mean	Lower 95% CL	Upper 95% CL	Group
Cur3	RMX180	0.7412	0.563	0.8643	a

Model summary

```
Linear mixed model fit by REML. t-tests use Satterthwaite's method ['lmerModLmerTest']
Formula: ziFormula
   Data: data
Weights: wi

REML criterion at convergence: 120.1

Scaled residuals:
     Min       1Q   Median       3Q      Max
-1.90613 -0.80783  0.01609  0.64147  1.04786

Random effects:
 Groups                      Name        Variance Std.Dev.
 Genotype_Zone_1:Plant_Zone_1 (Intercept) 0.2901   0.5386
 Genotype_Zone_2:Plant_Zone_2 (Intercept) 0.5697   0.7548
 Residual                                 1.5142   1.2305
Number of obs: 32, groups:  Genotype_Zone_1:Plant_Zone_1, 10; Genotype_Zone_2:Plant_Zone_2, 10

Fixed effects:
            Estimate Std. Error      df t value Pr(>|t|)
(Intercept)   1.0522     0.3645 11.3777   2.887   0.0143 *
---
Signif. codes:  0 '***' 0.001 '**' 0.01 '*' 0.05 '.' 0.1 ' ' 1

Dispersion: 1.231
```

Model residuals

Statistic	Value
Sample skewness	-0.437
Sample excess kurtosis	-0.7788
Passed Shapiro Wilk normality test	Yes (p-value = 0.117 > 0.05)

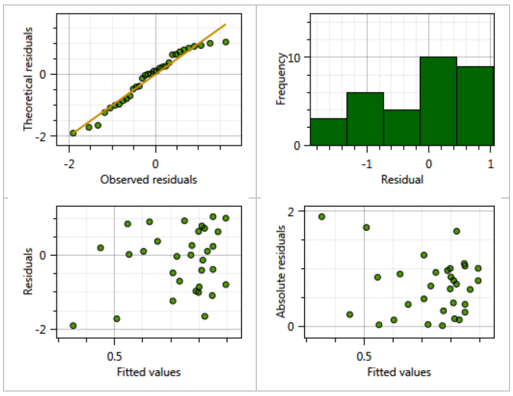

Analysis ratio movement to halting duration H1 (diff. Zone 1 - Zone 2)

Analysis model	Generalized linear mixed model with dispersion factor, formula=cbind(Ratio_movement_to_halting_duration_H1_Zone_1,Ratio_movement_to_halting_duration_H1_Zone_2) ~ 1 + (1|Genotype_Zone_1:Plant_Zone_1) + (1|Genotype_Zone_2:Plant_Zone_2)
**Transformation**	Logit

Confidence intervals ratio movement to halting duration H1 (diff. Zone 1 - Zone 2)

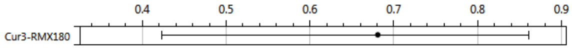

Confidence intervals ratio movement to halting duration H1 (diff. Zone 1 - Zone 2)

Genotype Zone 1	Genotype Zone 2	Mean	Lower 95% CL	Upper 95% CL	Group
Cur3	RMX180	0.6808	0.4228	0.8614	a

Model summary

```
Linear mixed model fit by REML. t-tests use Satterthwaite's method ['lmerModLmerTest']
Formula: ziFormula
   Data: data
Weights: wi

REML criterion at convergence: 147

Scaled residuals: 
    Min      1Q  Median      3Q     Max 
-2.2876 -0.4480 -0.1675  0.5199  1.9181 

Random effects:
 Groups                      Name        Variance Std.Dev.
 Genotype_Zone_1:Plant_Zone_1 (Intercept) 0.06116  0.2473  
 Genotype_Zone_2:Plant_Zone_2 (Intercept) 1.33008  1.1533  
 Residual                                 2.81030  1.6764  
Number of obs: 34, groups:  Genotype_Zone_1:Plant_Zone_1, 10; Genotype_Zone_2:Plant_Zone_2, 10

Fixed effects:
            Estimate Std. Error     df t value Pr(>|t|)
(Intercept)   0.7576     0.4689 8.5641   1.616    0.142

Dispersion: 1.676
```

Model residuals

Statistic	Value
Sample skewness	0.001798
Sample excess kurtosis	0.4979
Passed Shapiro Wilk normality test	Yes (p-value = 0.7137 > 0.05)

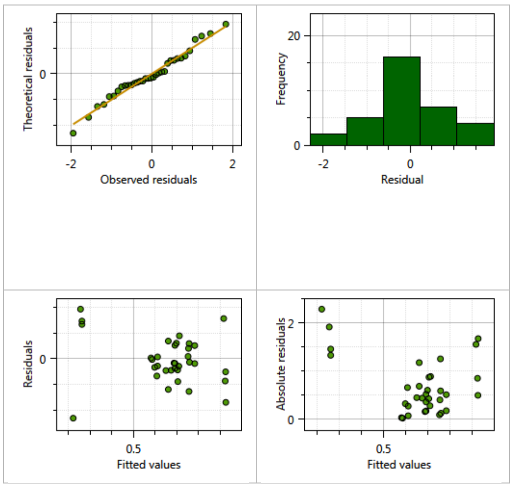

Analysis ratio movement to halting duration H2 (diff. Zone 1 - Zone 2)

**Analysis model**	Generalized linear mixed model with dispersion factor, formula=cbind(Ratio_movement_to_halting_duration_H2_Zone_1,Ratio_movement_to_halting_duration_H2_Zone_2) ~ 1 + (1|Genotype_Zone_1:Plant_Zone_1) + (1|Genotype_Zone_2:Plant_Zone_2)
**Transformation**	Logit

Confidence intervals ratio movement to halting duration H2 (diff. Zone 1 - Zone 2)

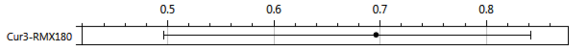

Confidence intervals ratio movement to halting duration H2 (diff. Zone 1 - Zone 2)

Genotype Zone 1	Genotype Zone 2	Mean	Lower 95% CL	Upper 95% CL	Group
Cur3	RMX180	0.6962	0.496	0.8422	a

Model summary

```
Linear mixed model fit by REML. t-tests use Satterthwaite's method ['lmerModLmerTest']
Formula: ziFormula
   Data: data
Weights: wi

REML criterion at convergence: 128.4

Scaled residuals: 
    Min      1Q  Median      3Q     Max 
-1.2775 -0.7335 -0.2226  0.4879  1.6820 

Random effects:
 Groups                      Name        Variance Std.Dev.
 Genotype_Zone_1:Plant_Zone_1 (Intercept) 0.5991   0.7740  
 Genotype_Zone_2:Plant_Zone_2 (Intercept) 0.4913   0.7009  
 Residual                                 0.8185   0.9047  
Number of obs: 35, groups:  Genotype_Zone_1:Plant_Zone_1, 10; Genotype_Zone_2:Plant_Zone_2, 10

Fixed effects:
            Estimate Std. Error     df t value Pr(>|t|)  
(Intercept)   0.8293     0.3710 8.6024   2.236   0.0535 .
---
Signif. codes:  0 '***' 0.001 '**' 0.01 '*' 0.05 '.' 0.1 ' ' 1

Dispersion: 0.9047
```

Model residuals

Statistic	Value
Sample skewness	0.6003
Sample excess kurtosis	-0.6196
Passed Shapiro Wilk normality test	Yes (p-value = 0.05333 > 0.05)

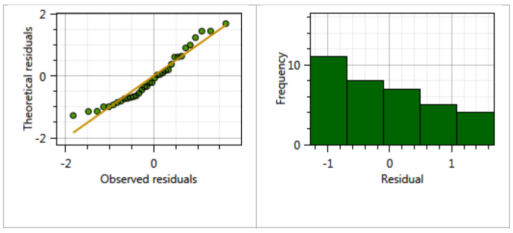

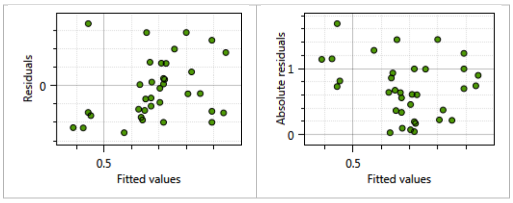

Analysis ratio movement to halting duration H3 (diff. Zone 1 - Zone 2)

**Analysis model**	Generalized linear mixed model with dispersion factor, formula=cbind(Ratio_movement_to_halting_duration_H3_Zone_1,Ratio_movement_to_halting_duration_H3_Zone_2) ~ 1 + (1|Genotype_Zone_1:Plant_Zone_1) + (1|Genotype_Zone_2:Plant_Zone_2)
**Transformation**	Logit

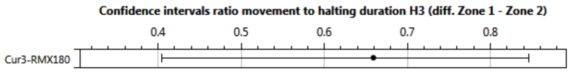

Confidence intervals ratio movement to halting duration H3 (diff. Zone 1 - Zone 2)

Genotype Zone 1	Genotype Zone 2	Mean	Lower 95% CL	Upper 95% CL	Group
Cur3	RMX180	0.6591	0.404	0.8464	a

Model summary

```
Linear mixed model fit by REML. t-tests use Satterthwaite's method ['lmerModLmerTest']
Formula: ziFormula
   Data: data
Weights: wi

REML criterion at convergence: 150.6

Scaled residuals:
    Min      1Q  Median      3Q     Max
-2.8093 -0.7079 -0.2808  0.1691  1.6285

Random effects:
 Groups                      Name        Variance Std.Dev.
 Genotype_Zone_1:Plant_Zone_1 (Intercept) 1.0063   1.0032
 Genotype_Zone_2:Plant_Zone_2 (Intercept) 0.4718   0.6869
 Residual                                2.9240   1.7100
Number of obs: 33, groups:  Genotype_Zone_1:Plant_Zone_1, 10; Genotype_Zone_2:Plant_Zone_2, 10

Fixed effects:
            Estimate Std. Error      df t value Pr(>|t|)
(Intercept)   0.6591     0.4826 12.3970   1.366    0.196

Dispersion: 1.71
```

Model residuals

Statistic	Value
Sample skewness	-0.4035
Sample excess kurtosis	1.746
Passed Shapiro Wilk normality test	Yes (p-value = 0.4531 > 0.05)

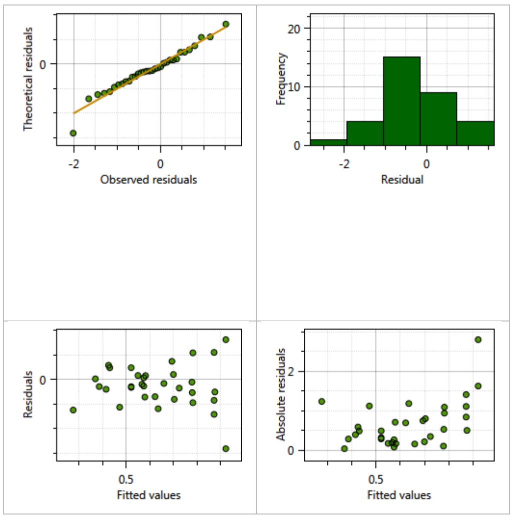

Analysis ratio movement to halting duration H4 (diff. Zone 1 - Zone 2)

**Analysis model**	Generalized linear mixed model with dispersion factor, formula=cbind(Ratio_movement_to_halting_duration_H4_Zone_1,Ratio_movement_to_halting_duration_H4_Zone_2) ~ 1 + (1|Genotype_Zone_1:Plant_Zone_1) + (1|Genotype_Zone_2:Plant_Zone_2)
**Transformation**	Logit

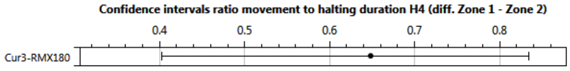

Confidence intervals ratio movement to halting duration H4 (diff. Zone 1 - Zone 2)

Genotype Zone 1	Genotype Zone 2	Mean	Lower 95% CL	Upper 95% CL	Group
Cur3	RMX180	0.6482	0.4021	0.8347	a

Model summary

```
Linear mixed model fit by REML. t-tests use Satterthwaite's method ['lmerModLmerTest']
Formula: ziFormula
   Data: data
Weights: wi

REML criterion at convergence: 93.9

Scaled residuals:
     Min       1Q   Median       3Q      Max
-1.57495 -0.52408  0.00396  0.42340  1.71245

Random effects:
 Groups                      Name        Variance Std.Dev.
 Genotype_Zone_1:Plant_Zone_1 (Intercept) 0.7898   0.8887
 Genotype_Zone_2:Plant_Zone_2 (Intercept) 0.8249   0.9083
 Residual                                0.4623   0.6799
Number of obs: 27, groups:  Genotype_Zone_1:Plant_Zone_1, 10; Genotype_Zone_2:Plant_Zone_2, 10
```

```
Fixed effects:
            Estimate Std. Error     df t value Pr(>|t|)
(Intercept)   0.6113     0.4470 9.1903   1.367    0.204

Dispersion: 0.6799
```

Model residuals

Statistic	Value
Sample skewness	0.1422
Sample excess kurtosis	0.1176
Passed Shapiro Wilk normality test	Yes (p-value = 0.9993 > 0.05)

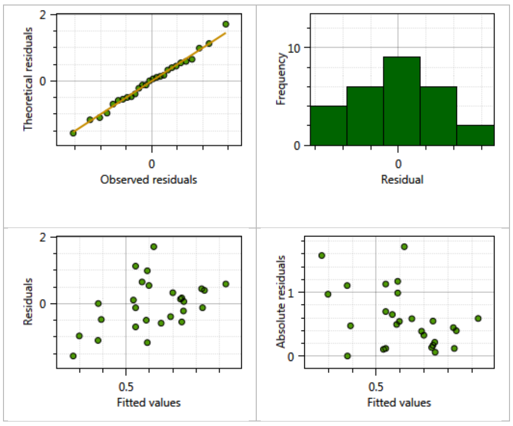

Analysis ratio movement to halting duration H5 (diff. Zone 1 - Zone 2)

**Analysis model**	Generalized linear mixed model with dispersion factor, formula=cbind(Ratio_movement_to_halting_duration_H5_Zone_1,Ratio_movement_to_halting_duration_H5_Zone_2) ~ 1 + (1|Genotype_Zone_1:Plant_Zone_1) + (1|Genotype_Zone_2:Plant_Zone_2)
**Transformation**	Logit

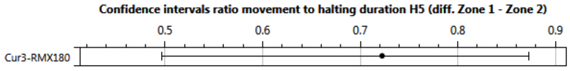

Confidence intervals ratio movement to halting duration H5 (diff. Zone 1 - Zone 2)

Genotype Zone 1	Genotype Zone 2	Mean	Lower 95% CL	Upper 95% CL	Group
Cur3	RMX180	0.7223	0.4963	0.8729	a

Model summary

```
Linear mixed model fit by REML. t-tests use Satterthwaite's method ['lmerModLmerTest']
Formula: ziFormula
   Data: data
Weights: wi

REML criterion at convergence: 135.3

Scaled residuals:
    Min      1Q  Median      3Q     Max
-2.5722 -0.6894 -0.3691  0.1739  1.7732

Random effects:
 Groups                      Name        Variance Std.Dev.
 Genotype_Zone_1:Plant_Zone_1 (Intercept) 0.1973   0.4442
 Genotype_Zone_2:Plant_Zone_2 (Intercept) 0.3120   0.5585
 Residual                                 2.3371   1.5287
Number of obs: 31, groups:  Genotype_Zone_1:Plant_Zone_1, 10; Genotype_Zone_2:Plant_Zone_2, 10

Fixed effects:
            Estimate Std. Error     df t value Pr(>|t|)
(Intercept)   0.9561     0.3803 5.1143   2.514   0.0525 .
---
Signif. codes:  0 '***' 0.001 '**' 0.01 '*' 0.05 '.' 0.1 ' ' 1

Dispersion: 1.529
```

Model residuals

Statistic	Value
Sample skewness	-0.01542
Sample excess kurtosis	0.8422
Passed Shapiro Wilk normality test	Yes (p-value = 0.5659 > 0.05)

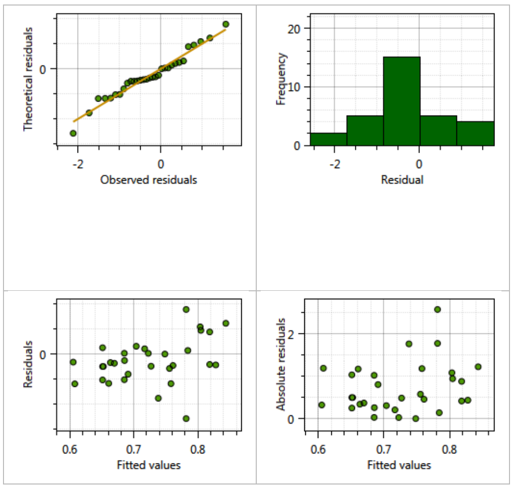

Analysis ratio movement to halting duration H6 (diff. Zone 1 - Zone 2)

**Analysis model**	Generalized linear mixed model with dispersion factor, formula=cbind(Ratio_movement_to_halting_duration_H6_Zone_1,Ratio_movement_to_halting_duration_H6_Zone_2) ~ 1 + (1|Genotype_Zone_1:Plant_Zone_1) + (1|Genotype_Zone_2:Plant_Zone_2)
**Transformation**	Logit

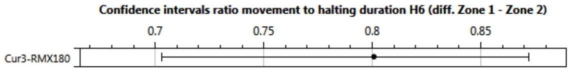

Confidence intervals ratio movement to halting duration H6 (diff. Zone 1 - Zone 2)

Genotype Zone 1	Genotype Zone 2	Mean	Lower 95% CL	Upper 95% CL	Group
Cur3	RMX180	0.8007	0.7029	0.8722	a

Model summary

```
Linear mixed model fit by REML. t-tests use Satterthwaite's method ['lmerModLmerTest']
Formula: ziFormula
   Data: data
Weights: wi

REML criterion at convergence: 96.8

Scaled residuals:
    Min      1Q  Median      3Q     Max
-2.0794 -0.7852 -0.1556  0.3049  1.6056

Random effects:
 Groups                      Name        Variance  Std.Dev.
 Genotype_Zone_1:Plant_Zone_1 (Intercept) 3.317e-14 1.821e-07
 Genotype_Zone_2:Plant_Zone_2 (Intercept) 0.000e+00 0.000e+00
 Residual                                1.509e+00 1.228e+00
Number of obs: 26, groups:  Genotype_Zone_1:Plant_Zone_1, 10; Genotype_Zone_2:Plant_Zone_2, 10

Fixed effects:
            Estimate Std. Error      df t value Pr(>|t|)
(Intercept)   1.3909     0.2573 25.0000   5.405 1.31e-05 ***
---
Signif. codes:  0 '***' 0.001 '**' 0.01 '*' 0.05 '.' 0.1 ' ' 1

Dispersion: 1.228
```

Model residuals

Statistic	Value
Sample skewness	-0.1296
Sample excess kurtosis	-0.413
Passed Shapiro Wilk normality test	Yes (p-value = 0.6303 > 0.05)

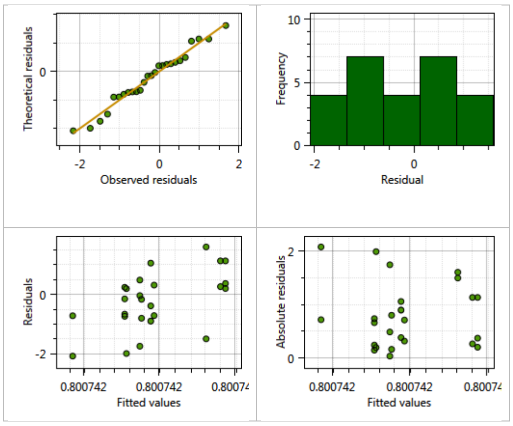

Analysis ratio movement to halting duration H7 (diff. Zone 1 - Zone 2)

**Analysis model**	Generalized linear mixed model with dispersion factor, formula=cbind(Ratio_movement_to_halting_duration_H7_Zone_1,Ratio_movement_to_halting_duration_H7_Zone_2) ~ 1 + (1|Genotype_Zone_1:Plant_Zone_1) + (1|Genotype_Zone_2:Plant_Zone_2)
**Transformation**	Logit

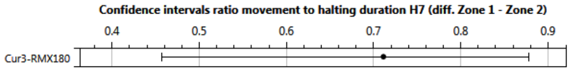

Confidence intervals ratio movement to halting duration H7 (diff. Zone 1 - Zone 2)

Genotype Zone 1	Genotype Zone 2	Mean	Lower 95% CL	Upper 95% CL	Group
Cur3	RMX180	0.7112	0.4566	0.8783	a

Model summary

```
Linear mixed model fit by REML. t-tests use Satterthwaite's method ['lmerModLmerTest']
Formula: ziFormula
   Data: data
Weights: wi

REML criterion at convergence: 112.1

Scaled residuals:
    Min      1Q  Median      3Q     Max
-2.0089 -0.5616 -0.1922  0.3123  1.5092

Random effects:
 Groups                      Name        Variance Std.Dev.
 Genotype_Zone_2:Plant_Zone_2 (Intercept) 0.1438   0.3793
 Genotype_Zone_1:Plant_Zone_1 (Intercept) 0.8932   0.9451
 Residual                                2.0676   1.4379
Number of obs: 27, groups:  Genotype_Zone_2:Plant_Zone_2, 10; Genotype_Zone_1:Plant_Zone_1, 9

Fixed effects:
            Estimate Std. Error     df t value Pr(>|t|)
(Intercept)   0.9012     0.4469 6.4527   2.017    0.087 .
---
Signif. codes:  0 '***' 0.001 '**' 0.01 '*' 0.05 '.' 0.1 ' ' 1

Dispersion: 1.438
```

Model residuals

Statistic	Value
Sample skewness	-0.2606
Sample excess kurtosis	0.0009445
Passed Shapiro Wilk normality test	Yes (p-value = 0.8588 > 0.05)

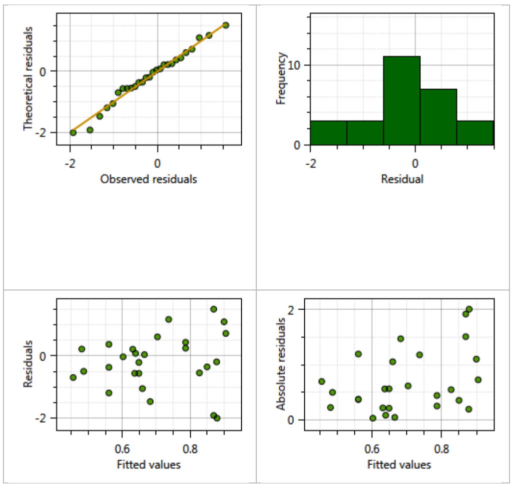

Theoretical residuals
0
-2
-2
0
Observed residuals
Frequency
10
0
-2
0
Residual
Residuals
0
-2
0.6
0.8
Fitted values
Absolute residuals
2
0
0.6
0.8
Fitted values
